# Functionalized azetidines via visible light-enabled aza Paternò-Büchi reactions

**DOI:** 10.1038/s41467-019-13072-x

**Published:** 2019-11-08

**Authors:** Marc R. Becker, Alistair D. Richardson, Corinna S. Schindler

**Affiliations:** Willard Henry Dow Laboratory, Department of Chemistry, University of Michigan, Ann Arbor, MI 48109 USA

**Keywords:** Drug discovery and development, Photocatalysis, Synthetic chemistry methodology

## Abstract

Azetidines are four-membered nitrogen-containing heterocycles that hold great promise in current medicinal chemistry due to their desirable pharmacokinetic effects. However, a lack of efficient synthetic methods to access functionalized azetidines has hampered their incorporation into pharmaceutical lead structures. As a [2+2] cycloaddition reaction between imines and alkenes, the aza Paternò-Büchi reaction arguably represents the most direct approach to functionalized azetidines. Hampered by competing reaction paths accessible upon photochemical excitation of the substrates, the current synthetic utility of these transformations is greatly restricted. We herein report the development of a visible light-enabled aza Paternò-Büchi reaction that surmounts existing limitations and represents a mild solution for the direct formation of functionalized azetidines from imine and alkene containing precursors.

## Abstract (web summary)

The Aza Paternò-Büchi reaction is arguably among the most direct approaches to functionalized azetidines, which are common medicinal scaffolds. Here, the authors report a mild and selective visible light-enabled intramolecular aza Paternò-Büchi reaction yielding bicyclic azetidines in high yields and diastereoselectivity.

## Introduction

**Fig. 1:**
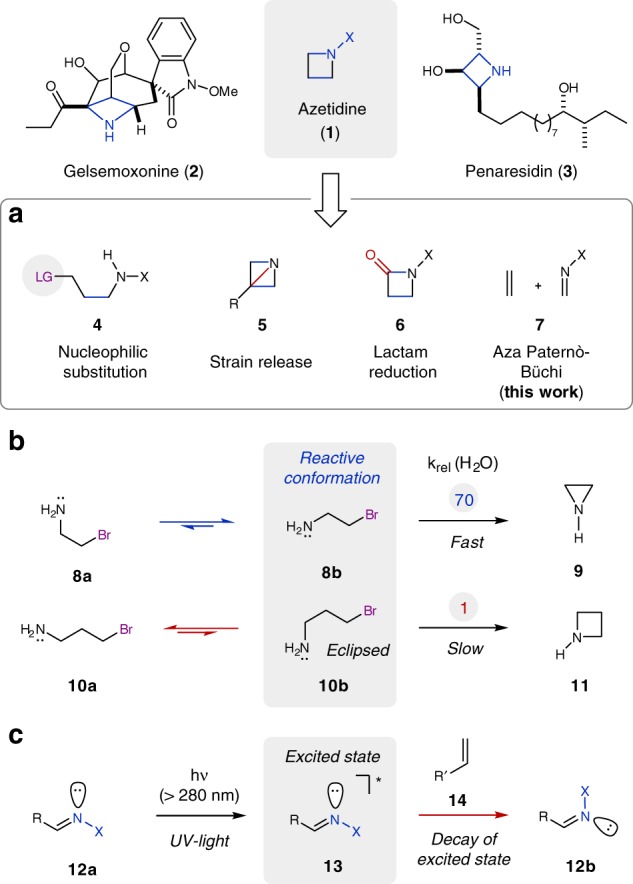
Previous strategies towards azetidines and this approach. **a** Select synthetic strategies. **b** Challenges in nucleophilic substitution reactions. **c** Challenges in aza Paternò–Büchi reactions

Nitrogen-containing heterocycles are essential structural components for drug design and are currently incorporated in more than 59% of all pharmaceuticals approved by the US Food and Drug Administration (FDA)^1^. The majority of these contain five- and six-membered pyrrolidine and pyridine derivatives, regardless of the fact that smaller heterocycles, such as the four-membered azetidines (**1**), are known to display superior physicochemical properties and increased bioavailability, as well as metabolic stability^2–6^. Despite these desirable characteristics, azetidines remain underutilized in current medicinal chemistry, which is a direct result of a lack of efficient synthetic methods for their construction^7–9^. The most important strategy for the synthesis of saturated nitrogen-containing heterocycles relies on unimolecular cyclization reactions via nucleophilic substitution (**4**, Fig. 1a)^10^. While this approach results in the efficient formation of three-, five-, and six-membered heterocycles, it often fails to yield the four-membered azetidines (**11**). In general, four-membered rings are considered the hardest of all to form^11^. The reason for this lack of reactivity lies in the preferred conformation of the acyclic precursors (Fig. 1b). Specifically, the formation of three-membered rings (**9**) is favorable as **8b** represents both the preferred as well as reactive conformation for cyclization. In comparison, formation of the four-membered azetidines is hampered as it requires access to conformation **10b**, which is higher in energy due to unfavorable eclipsing interactions^12^. Thus, strategies that proceed upon strain release of azabicyclobutanes (**5**) as three-membered ring analogs were developed as viable alternatives for the synthesis of azetidines^13–18^. In addition, orthogonal strategies for azetidine synthesis were developed that rely on the reduction of more readily accessible *β*-lactam precursors (**6**)^10,19,20^. Arguably, aza Paternò–Büchi reactions (**7**) represent the most efficient and direct strategy towards azetidines. Analogous to the Paternò–Büchi reaction^21^, in which an excited state carbonyl undergoes a [2+2] cycloaddition with an alkene, aza Paternò–Büchi reactions rely on imines and alkenes. However, the development of these transformations has met challenges associated with the decreased photoreactivity of imine precursors^22,23^. In particular, the excited state of imines (**13**) is known to undergo facile and preferential radiationless decay upon rotation about the C = N π-bond (**12b**)^24–27^. This results in dissipation of electronic energy and a lack of reactivity in [2+2] cycloadditions with alkenes (**14)**^28–42^ (Fig. 1c). Hence, successful reports of aza Paternò–Büchi reactions are rare and limited to rigid imine- and alkene-containing systems predisposed for cycloaddition in addition to the stringent requirement of high energy ultraviolet (UV) light^43–51^. Consequently, the development of a mild and general reaction protocol for aza Paternò–Büchi reactions relying on visible light^52–57^ would be highly desirable.

Here, we report the development of a visible light-mediated aza Paternò–Büchi reaction, which enables the synthesis of highly functionalized azetidines from readily available imine and alkene-containing precursors. Notably, this strategy is characterized by its mild conditions, operational simplicity, and scalability. The accessible azetidine products are readily converted into more valuable azetidine building blocks.

## Results

### Reaction optimization of aza Paternò–Büchi reaction

**Fig. 2:**
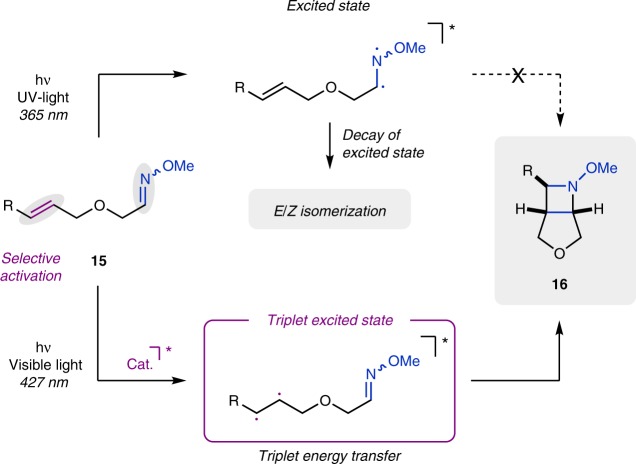
Reaction design of the aza Paternò–Büchi reaction. Direct irradiation leads to unproductive excitation of the oxime moiety, while selective activation of the alkene via triplet energy transfer enables the [2 + 2] photocycloaddition

At the outset of our studies, we envisioned an orthogonal approach for aza Paternò–Büchi reactions that relies on selective activation of the alkene functionality to avoid excitation of the imine and associated competing reaction paths (Fig. 2). Triplet energies of alkenes^58^ (e.g., styrenes, dienes) are known to be lower than those of functionalized imines^59–62^, and could thus engage selectively in an energy transfer process with a suitable excited photocatalyst to reach the triplet state of the alkene. Subsequent [2+2] cycloaddition with the C = N double bond of the imine moiety would then lead to the formation of the desired azetidine product. Importantly, activation of the photocatalyst could be achieved by irradiation with visible light and thus forgo excitation of the imine and associated decay pathways.

Our initial investigations into the development of a mild protocol for an aza Paternò–Büchi reaction centered on oximes such as **15** due to their facile synthesis and superior stability towards hydrolysis compared to imines. Consistent with literature reports, irradiation of oxime **15** with UV light resulted in *E*/*Z* isomerization of the oxime and only trace amounts of azetidine **16** as the desired aza Paternò–Büchi product^63^ (entry 1, Table 1). Conversion of **15** with catalytic amounts of xanthone as photosensitizer and UV light following conditions previously reported by Sivaguru and co-workers^51^ resulted in 43% yield albeit complete conversion of the substrate (entry 2, Table 1). We postulated that substrate decomposition is a direct consequence of the high redox properties of xanthone upon irradiation with UV light^64^. Following our initial hypothesis that selective alkene activation to its corresponding triplet state could lead to a mild reaction protocol for aza Paternò–Büchi reactions, we next evaluated a variety of photocatalysts in combination with lower energy visible light (40 W blue light-emitting diode (LED) light at 427 nm). When oxime **15** was irradiated with visible light in the presence of catalytic amounts of [Ru(bpy)_3_](PF_6_)_2_, no formation of azetidine **16** was observed (entry 3, Table 1). Nevertheless, when Ir(ppy)_3_ was used as photocatalyst under otherwise identical conditions, the desired aza Paternò–Büchi product **16** was formed in 39% yield (entry 4, Table 1). Reaction of oxime **15** with 2.5 mol% of Ir[dF(CF_3_)ppy]_2_(dtbbpy)PF_6_ (**17**•PF_6_) resulted in the formation of azetidine **16** in increased yields of 97% and >20:1 diastereomeric ratios (d.r.) (entry 5, Table 1). The potency of this photocatalyst was previously established by Yoon group^65,66^ in the [2+2] cycloaddition between alkenes for the formation of functionalized cyclobutanes. Further reaction optimization identified THF as optimal solvent resulting in 98% yield of azetidine **16** with catalyst loadings of only 0.5 mol% **17**•PF_6_ (entry 15, Table 1). Finally, control reactions revealed that both light and photocatalyst were necessary for the [2 + 2] photocycloaddition to proceed (entries 16–17, Table 1)^67–71^.

**Table 1:** Reaction optimization


Entry	Catalyst (mol%)	Wavelength (nm)	Solvent	Concentration (M)	Yield (%)^a^
1^b^	–	365	CH_2_Cl_2_	0.01	6
2^c^	Xanthone (30)	365	MeCN	0.01	43
3	[Ru(bpy)_3_](PF_6_)_2_ (2.5)	427	THF	0.01	–
4	Ir(ppy)_3_ (2.5)	427	THF	0.01	39
5	**17**•PF_6_ (2.5)	427	THF	0.01	97
6	**17**•PF_6_ (2.5)	427	CH_2_Cl_2_	0.025	72
7	**17**•PF_6_ (2.5)	427	MeOH	0.025	87
8	**17**•PF_6_ (2.5)	427	EtOAc	0.025	87
9	**17**•PF_6_ (2.5)	427	acetone	0.025	86
10	**17**•PF_6_ (2.5)	427	MeCN	0.025	88
11	**17**•PF_6_ (2.5)	427	THF	0.025	93
12	**17**•PF_6_ (2.5)	427	THF	0.05	88
13	**17**•PF_6_ (2.5)	427	THF	0.10	90
14	**17**•PF_6_ (1.0)	427	THF	0.01	96
15	**17**•PF_6_ (0.5)	427	THF	0.01	98
16	–	427	THF	0.01	–
17^d^	**17**•PF_6_ (0.5)	–	THF	0.01	–

Conditions: Reactions performed on 0.1 mmol scale under irradiation with a blue LED light (427 nm) for 0.5 h at ambient temperature (fan cooling)

^a^Yield determined by quantitative ^1^H NMR analysis from the crude mixture using an internal standard

^b^For 24 h

^c^For 12 h

^d^Run in the dark

### Substrate scope of aza Paternò–Büchi reaction

With optimized reaction conditions established, we next evaluated different oximes and hydrazones for their ability to undergo the [2 + 2] cycloaddition (Table 2). It is important to note that all substrates were prepared and used as mixtures of *E*/*Z* oxime or hydrazone isomers. However, the observed diastereoselectivity of the azetidine products formed was found to be independent of the *E*/*Z* ratio of the substrate. *O*-Benzyl oxime **18a** converted smoothly to **19a** in 96% yield and very good diastereoselectivity of >20:1 d.r., providing similar results as the *O*-methyl oxime **15** (entries 1–2, Table 2a). Notably, the reaction can be performed on gram scale at slightly higher concentration with no significant decrease in yield. Free oxime **18b** and *N*-Boc hydrazone **18c** were reacted under the optimized conditions, and the corresponding azetidines **19b** and **19c** were isolated in 54 and 62% yield, respectively (entries 3–4, Table 2a). Additionally, the structure of **19c** was subsequently verified by X-ray analysis (Table 2b). Interestingly, no reaction was observed with *N*,*N*-dimethyl hydrazone **18d** and only unreacted starting material was recovered from the reaction mixture (entry 5, Table 2a). While other substituted imines (e.g., *N*-tosyl imines) successfully underwent the aza Paternò–Büchi reaction, the poor stability towards hydrolysis of the corresponding substrates prohibited sufficient purification and led to reproducibility issues.

**Table 2:**
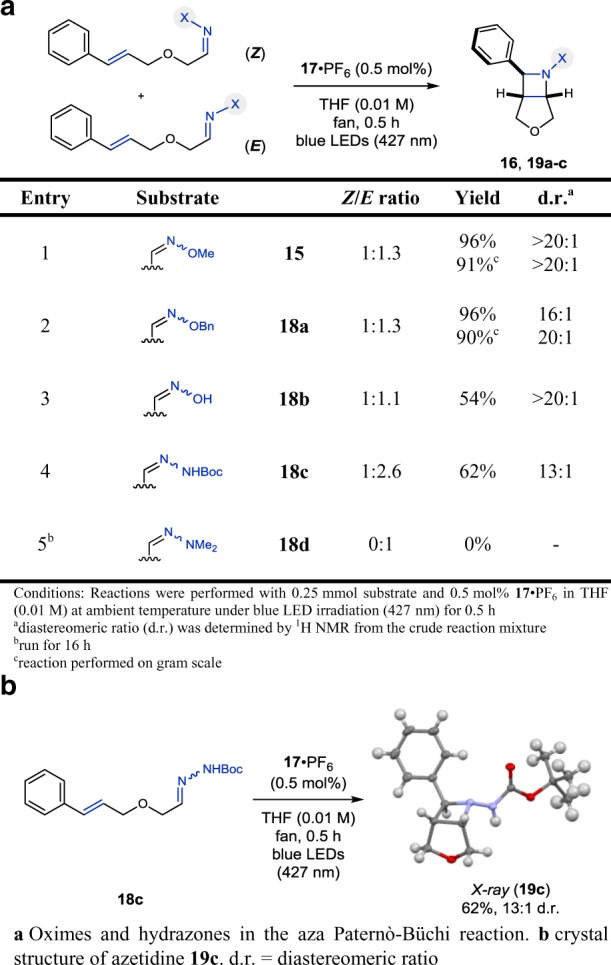
Evaluation of oxime and hydrazone substrates

**Fig. 3:**
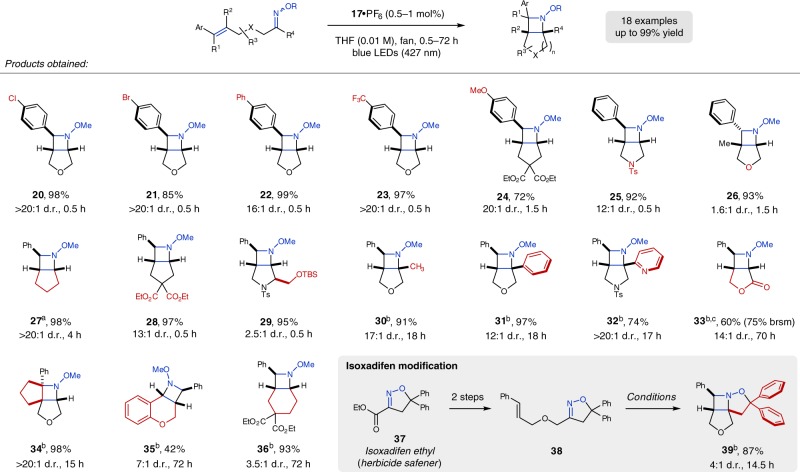
Scope of the [2 + 2] cycloaddition reaction. Reactions were performed with 0.25 mmol substrate (mixture *E*/*Z* oxime isomers) and 0.5–1.0 mol% **17**•PF_6_ in THF (0.01 M) at ambient temperature (fan cooling) under blue LED irradiation (427 nm) unless noted; diastereomeric ratios (d.r.) were determined by ^1^H NMR from the crude reaction mixture; isolated yields refer to the mixture of diastereomers (major diastereomer is given); ^a^on 500 mg scale; ^b^under N_2_ atmosphere; ^c^run at 82 °C

We subsequently investigated the scope of the aza Paternò–Büchi reaction with a particular emphasis on functional group tolerance and the ability to rapidly construct functionalized azetidines (Fig. 3). The optimized conditions proved efficient for a variety of electronically diverse styrenes including both electron-rich and electron-deficient systems, affording the corresponding azetidines in excellent yields and diastereoselectivities (**20**–**24**). Furthermore, different substitution patterns on the styrene moiety as well as the substrate backbone including esters and sulfonamides were well tolerated, providing a set of densely substituted azetidines (**25**–**29**). While the developed transformation could be readily conducted under ambient atmosphere in short reaction times of 30 min, substrates bearing increased steric constraints were found to require extended reaction times (**30**–**36**). It was beneficial to conduct these transformations in degassed solvent to minimize undesired reactivity with atmospheric oxygen to ultimately obtain the desired azetidine products in high yields of up to 98%. Specifically, oximes derived from methyl and phenyl ketones were converted to the corresponding azetidines **30** and **31** in excellent yield. The reaction is also amenable to heteroaromatic ketones—2-pyridyl azetidine **32** was afforded in 74% yield and excellent diastereoselectivity. Additionally, azetidine **33** bearing a lactone tether was formed in 60% (75% brsm) after irradiation for 70 h at elevated temperatures. Importantly, substrates containing internal esters are often found less reactive in many ring-closing transformations, considering that the reaction can only proceed through the less favored *s*-*trans* ester conformation^72^. Furthermore, cyclic tetrasubstituted styrenes are compatible with the reaction conditions, providing tricyclic azetidine **34** in 98% yield. We also evaluated the feasibility of substrates to form azetidines fused to six-membered rings upon [2 + 2] cycloaddition. The corresponding substrates were found to be significantly less reactive; nevertheless, chromane **35** and cyclohexane **36** were obtained after irradiation for 72 h in 42 and 93% yield, respectively. Based on the fact that many pharmaceutical or agrochemical products contain cyclic oxime or hydrazone motifs, we were interested in evaluating whether the aza Paternò–Büchi reaction protocol developed herein would allow for their late-stage modification. Cyclic oxime **38** derived from the herbicide safener isoxadifen ethyl (**37**) provided highly functionalized azetidine **39** in 87% yield, thus enabling late-stage modification of an industrially important oxime.

**Fig. 4:**
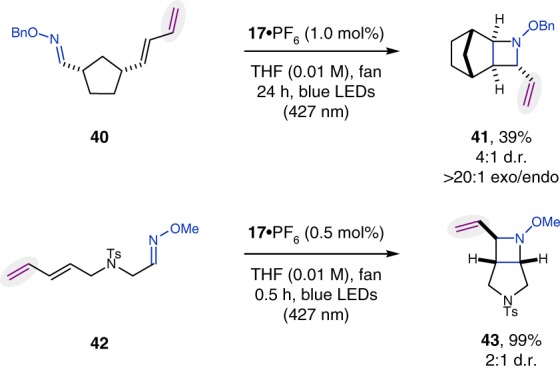
Aza Paternò–Büchi reaction utilizing dienes. Reactions were performed with 0.25 mmol substrate; diastereomeric ratios (d.r.) were determined by ^1^H NMR from the crude reaction mixture; isolated yields refer to the mixture of diastereomers (major diastereomer is given)

Subsequent investigations focused on the compatibility of dienes as alkene equivalent with the developed procedure for visible light-mediated aza Paternò–Büchi reactions (Fig. 4)^66^. After reacting the corresponding diene **40** under the optimized conditions, strained bicycle **41** was isolated as the product of the reaction in 39% yield. Similarly, azetidine **43** was obtained in 99% yield. Notably, no products resulting from competing [4 + 2] cycloadditions were observed in either one of these reactions.

### Mechanistic investigations

**Fig. 5:**
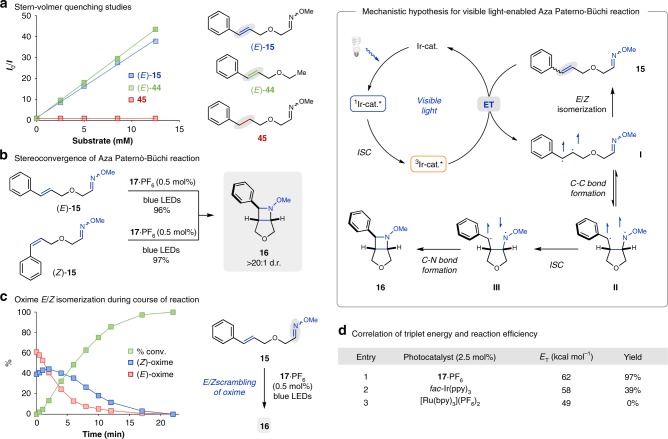
Mechanistic investigations of the title reaction. **a** Stern–Volmer quenching studies. **b** Stereoconvergence of the aza Paternò–Büchi reaction. **c** Oxime *E*/*Z* isomerization during the course of the reaction. **d** Correlation of triplet energy and reaction efficiency (see Supplementary Methods for details). *E*_T_ = triplet energy; ET = energy transfer; ISC = intersystem crossing

Ensuing efforts focused on gaining additional insights into the controlling features of the visible light-mediated aza Paternò–Büchi reaction. A Stern–Volmer quenching study unambiguously showed that the styrene moiety is necessary for productive quenching of the photocatalyst, while the corresponding oxime **45** lacking a styrene moiety does not result in significant quenching of **17**•PF_6_ (Fig. 5a). We postulate that a photoredox process is unlikely under the optimized reaction conditions as the excited state redox potentials of **17**•PF_6_ (*E*_1/2_^III*/II^ = +1.21 V vs. SCE; *E*_1/2_^IV/III*^ = –0.89 V vs. SCE)^73^ are not sufficient for an effective oxidation or reduction of substrate **15** (see Supplementary Methods for additional details). To further confirm this hypothesis, a series of control experiments was conducted, which imply that a triplet energy transfer mechanism from the photocatalyst to the styrene moiety of the substrate is operative. Styrenes possess a triplet energy (*E*_T_) of ~60 kcal mol^−1^, which suggest that **17**•PF_6_ (*E*_T_ = 62 kcal mol^−1^) is capable of sensitizing substrate **15**^58^. In comparison, the efficiency of this transformation significantly decreases with photocatalysts that have a triplet energy below 60 kcal mol^−1^. While *fac*-Ir(ppy)_3_ (*E*_T_ = 58 kcal mol^−1^) is still able to mediate product formation, albeit less efficiently, Ru(bpy)_3_^2+^ (*E*_T_ = 49 kcal mol^−1^) was found incapable of catalyzing the desired aza Paternò–Büchi reaction (Fig. 5d)^74^. Consistent with this hypothesis, substrates bearing terminal alkenes with significantly higher triplet energy (~76–84 kcal mol^−1^)^58^ were not found to undergo the desired transformation, but instead resulted in exclusive isolation of starting material (see Supplementary Methods for additional details). Additionally, the aza Paternò–Büchi reaction developed herein was found to be stereoconvergent, as both (*E*)- and (*Z*)-**15** gave identical results under standard conditions (Fig. 5b). The interconversion of both styrene isomers upon photosensitization is fast and occurs at a rate similar to product formation (see Supplementary Fig. 4). Interestingly, monitoring the oxime isomer ratio of **15** over the course of the reaction revealed that oxime *E*/*Z* scrambling occurs at low conversion (Fig. 5c). In particular, within the first 2 min of the reaction an increase in (*Z*)-oxime concentration was observed along with a change in *E*/*Z* ratio from 1.6:1 to 1:1. This observation cannot be accounted for based on the faster conversion of (*E*)-oxime to azetidine **16** in comparison to (*Z*)-oxime. In contrast, no change in *E*/*Z* ratio was observed for compound **45** lacking the styrene moiety. Based on the results obtained in these investigations, we propose a reaction mechanism that relies on a styrene triplet manifold (intermediate I, Fig. 5). Efficient triplet energy transfer from photoexcited **17**•PF_6_ accessible upon irradiation with visible light results in a triplet styrene (intermediate I) that undergoes subsequent reversible *C*–*C* bond formation to result in a 1,4-biradical (intermediate II). This intermediate allows for free rotation around the *C*–*N* bond that ultimately leads to the observed *E*/*Z* scrambling of the oxime after ring-opening (intermediate I) and relaxation back to the ground state. Alternatively, intermediate II can undergo intersystem crossing (ISC) (intermediate III), and deliver the azetidine product (**16**) after the final *C*–*N* bond-forming step. Importantly, the biradical nature of the involved triplet intermediates leads to complete loss of stereoinformation, which results in the formation of the same diastereomer independent of the oxime or alkene isomer ratios of the starting material.

### Synthetic applications

**Fig. 6:**
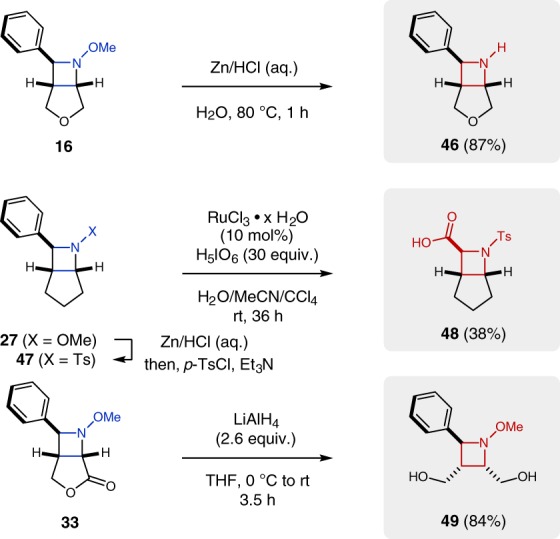
Synthetic modifications of azetidine products. The accessed azetidines can be converted to the corresponding unprotected azetidine (**46**), azetidine-2-carboxylic acid (**48**), or monocyclic azetidine (**49**)

The developed [2+2] cycloaddition protocol enables rapid access to highly functionalized azetidines under mild conditions that can function as versatile building blocks to undergo further diversification (Fig. 6). The azetidine *N*–*O* bond can be readily cleaved with zinc metal under acidic conditions providing free azetidine **46** in 87% yield. To demonstrate the utility of the 2-phenylazetidine motif accessible by this method, azetidine **27** was converted to the corresponding *N*-Ts azetidine **47** (70% yield over 2 steps), which is amenable to phenyl ring oxidation utilizing RuCl_3_/H_5_IO_6_ to provide carboxylic acid **48** in 38% yield. Notably, **48** represents a previously inaccessible analog of the non-proteinogenic amino acid azetidine-2-carboxylic acid (Aze). Finally, the lactone tether of azetidine **33** is readily cleaved with LiAlH_4_ to provide **49** in 84% yield, resembling the product of a net intermolecular [2 + 2] cycloaddition reaction.

## Discussion

We herein report the development of a visible light-mediated aza Paternò–Büchi reaction between alkene and oxime moieties that results in the direct formation of functionalized azetidines, with yields of up to 99% and >20:1 d.r. The approach described relies on the selective activation of the alkene functionality upon energy transfer from a suitable photocatalyst to its corresponding triplet state. As a result, the aza Paternò–Büchi reaction developed herein overcomes previous challenges associated with the excitation of functionalized imines and resulting undesired competing reaction paths. We expect that this strategy will provide a new platform for the facile synthesis of azetidines and will enable further advancements in developing new enabling [2 + 2] cycloadditions involving carbon–nitrogen double bonds.

## Methods

### Representative procedure

A test tube was charged with **15** (0.25 mmol), **17**•PF_6_ (0.5 mol%), and tetrahydrofuran (25 mL), sealed with a rubber septum and placed in front of a 40 W PR160-427 nm Kessil light (5 cm distance; 100% intensity). Upon completion as judged by thin layer chromatography analysis, the reaction mixture was concentrated in vacuum. The diastereomeric ratio was determined by ^1^H NMR (proton nuclear magnetic resonance) analysis from the crude reaction mixture, before purification by flash column chromatography (10–20% EtOAc/hexanes) to afford pure **16**.

## Supplementary information

Supplementary Information

## Data Availability

Experimental data as well as ^1^H and ^13^C NMR spectra for all new compounds prepared in the course of these studies are provided in the supplementary information of this manuscript. The X-ray crystallographic coordinates for compound **19c** have been deposited at the Cambridge Crystallographic Data Center (CCDC) with the accession code 1873931 (10.5517/ccdc.csd.cc20wzd9). These data can be obtained free of charge from The Cambridge Crystallographic Data Center via www.ccdc.cam.ac.uk/data_request/cif. All other data including synthetic procedures are available in the supplementary information files.
