# Supplementary material for: Functionalized azetidines via visible light-enabled aza Paternò-Büchi reactions
Source: Nat Commun. 2019 Nov 8;10:5095. doi: 10.1038/s41467-019-13072-x (PMC6841681; doi:10.1038/s41467-019-13072-x)
Supplement: Supplementary file 1 — Supplementary Information [file 41467_2019_13072_MOESM1_ESM.pdf]

Supplementary Information

Functionalized Azetidines via Visible-Light Enabled Aza Paternò-Büchi Reactions

Becker et al.

Supplementary Methods

General Laboratory Procedures. All air- or moisture-sensitive reaction were carried out in flame-dried glassware under an atmosphere of nitrogen. Thin-layer chromatography (TLC) was performed on *Merck* silica gel 60 F_{254} plates using UV light (254 or 366 nm), $KMnO_4$ or CAM stain for visualization. Flash chromatography was performed using silica gel Silia Flash® 40-63 micron (230-400 mesh) from Silicycle.

Materials and Instrumentation. All chemicals were purchased from Sigma-Aldrich, Alfa Aesar, Acros Organics, Oakwood, TCI America, Frontier Scientific, Matrix Scientific, Ark Pharm, Strem and Chem Impex International, and were used as received unless otherwise stated. THF, CH_2Cl_2, Et_2O, MeOH, MeCN and DMF were dried by being passed through a column of activated alumina under argon using a JC-Meyer Solvent Systems. Triethylamine was freshly distilled prior to use over CaH. [Ir(dF(CF$_3$)ppy)$_2$(dtbbpy)]PF$_6$ (**17**•PF$_6$) was prepared according to the procedure described by Stephenson[1]. 2-Iodoxybenzoic acid (IBX) was prepared as described by Santagostino[2]. Proton nuclear magnetic resonance (^{1}H NMR) spectra were recorded on Varian MR400, Varian vnmrs 500, Varian Inova 500, and Varian vnmrs 700 spectrometers and are referenced to residual protic NMR solvent ($CDCl_3$: δ 7.26 ppm, CD_2Cl_2: δ 5.32 ppm). Data for ^{1}H NMR are reported as follows: chemical shift (δ ppm), multiplicity (s = singlet, d = doublet, t = triplet, q = quartet, m = multiplet, b = broad), coupling constant (Hz), integration. Carbon nuclear magnetic resonance (^{13}C NMR) spectra were recorded on Varian vnmrs 500 and Varian vnmrs 700 spectrometers and are referenced to the carbon resonances of the NMR solvent ($CDCl_3$: δ 77.16 ppm, CD_2Cl_2: δ 54.00 ppm). High-resolution mass spectrometry (MS) data was recorded at the Mass Spectrometry Facility at the Department of Chemistry of the University of Michigan in Ann Arbor, MI on an Agilent 6230 TOF HPLC-MS (ESI) or Micromass AutoSpec Ultima Magnetic Sector mass spectrometer (ESI, EI). Infrared (IR) spectra were obtained using a Thermo-Nicolet IS-50 spectrometer. IR data are represented as frequency of absorption (cm^{-1}). Stereochemistry indicators with asterisk (*R**, *S**) were used to indicate relative stereochemistry of diastereomers.

Abbreviations used: AcOH = acetic acid, Ag/AgCl = silver/silver chloride, aq. = aqueous, brsm = based on recovered starting material, CaH = calcium hydride, CAM = cerium ammonium molybdate, CD_2Cl_2 = deuterated dichloromethane, CCl_4 = carbon tetrachloride, $CDCl_3$ = deuterated chloroform, CH_2Cl_2 = dichloromethane, CuBr = copper(I) bromide, DIBAL-H = diisobutylaluminium hydride, d.r. = diastereomeric ratio, DMF = *N*,*N*-dimethylformamide, DMSO = dimethylsulfoxide, EI = electron ionization, ESI = electrospray ionization, E_T = triplet energy, Et_2O = diethyl ether, Et_3N = triethylamine, EtOAc = ethyl acetate, EtOH = ethanol, HCl = hydrochloric acid, IBX = 2-iodoxybenzoic acid, IR = infrared, K_2CO_3 = potassium carbonate, KCl = potassium chloride, KI = potassium iodide, $KMnO_4$ = potassium permanganate, $LiAlH_4$ = lithium aluminum hydride, MeCN = acetonitrile, MeOH = methanol, $MgSO_4$ = magnesium

sulfate, MS = mass spectrometry, n-Bu_4NPF_6 = tetrabutylammonium hexafluorophosphate, Na_2SO_4 = sodium sulfate, NaH = sodium hydride, $NaHCO_3$ = sodium bicarbonate, $NaHSO_3$ = sodium bisulfite, $NaIO_4$ = sodium periodate, NaOAc = sodium acetate, NaOH = sodium hydroxide, NH_4Cl = ammonium chloride, NMR = nuclear magnetic resonance, *p*-TsOH = *p*-toluenesulfonic acid monohydrate, *p*-TsCl = *p*-toluenesulfonyl chloride, rt = room temperature, $RuCl_3$ = ruthenium(III) chloride, sat. = saturated, SCE = saturated calomel electrode, TBSCl = *tert*-butyldimethylsilyl chloride, THF = tetrahydrofuran, TLC = thin-layer chromatography, UV = ultraviolet.

Reaction optimization

Supplementary Table 1. Reaction optimization. [a]for 24 h; [b]for 12 h; [c]run in the dark.

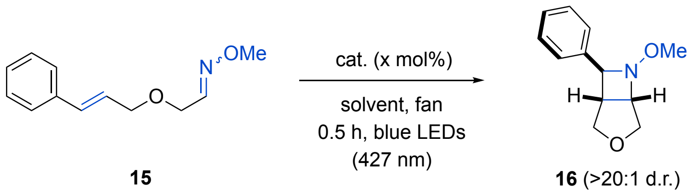

15

16 (>20:1 d.r.)

entry	catalyst (mol%)	ν (nm)	solvent	conc. [M]	yield
1[a]	-	365 (UV)	CH_2Cl_2	0.01	6%
2[b]	Xanthone (30)	365 (UV)	MeCN	0.01	43%
3	$[Ru(bpy)_3](PF_6)_2$ (2.5)	427	THF	0.01	-
4	*fac*-$Ir(Fppy)_3$ (2.5)	427	THF	0.01	30%
5	*fac*-$Ir(ppy)_3$ (2.5)	427	THF	0.01	39%
6	*fac*-$Ir(dFppy)_3$ (2.5)	427	THF	0.01	52%
7	**17**•PF_6 (2.5)	427	THF	0.01	97%
8	**17**•PF_6 (2.5)	427	CH_2Cl_2	0.025	72%
9	**17**•PF_6 (2.5)	427	MeOH	0.025	87%
10	**17**•PF_6 (2.5)	427	EtOAc	0.025	87%
11	**17**•PF_6 (2.5)	427	acetone	0.025	86%
12	**17**•PF_6 (2.5)	427	MeCN	0.025	88%
13	**17**•PF_6 (2.5)	427	THF	0.025	93%
14	**17**•PF_6 (2.5)	427	THF	0.05	88%
15	**17**•PF_6 (2.5)	427	THF	0.10	90%
16	**17**•PF_6 (1.0)	427	THF	0.01	96%
**17**	**17•PF_6 (0.5)**	**427**	**THF**	**0.01**	**98%**
18	-	427	THF	0.01	-
19[c]	**17**•PF_6 (0.5)	-	THF	0.01	-

A test tube was charged with **15** (21 mg, 0.1 mmol, 1.0 equiv.), photocatalyst and solvent, then sealed with a rubber septum and placed in front of a 40 W PR160-427 nm Kessil light at a distance of approximately 5 cm, which was set to 100% intensity (reactions involving UV light were carried out in a Luzchem LZG-ORG photoreactor). After stirring for 0.5 h, the reaction mixture was transferred to a 50-mL round-bottom flask and the solvent removed *in vacuo*. The crude reaction mixture was analyzed by ^{1}H NMR to determine the yield of **16** using mesitylene as internal standard.

Electrochemical Measurements

Cyclic voltammetry was performed on a CHI620E electrochemical analyzer (CH instruments) using a 3-mL five-necked electrochemical cell equipped with a carbon working electrode, a platinum counter or auxiliary electrode, an Ag/AgCl (3 M KCl) reference electrode and a scan rate of 100 mV/s. The experimental setup was calibrated using ferrocene (Fc^+/Fc) prior to each experiment. Samples were prepared with 0.03 mmol substrate in 3 mL *n*-Bu_4NPF_6 electrolyte (0.1 M in MeCN) and degassed by sparging with argon gas for 10 min prior to use. The potential ($E_{p/2}$) was determined and converted to SCE as described by Nicewicz.[3]

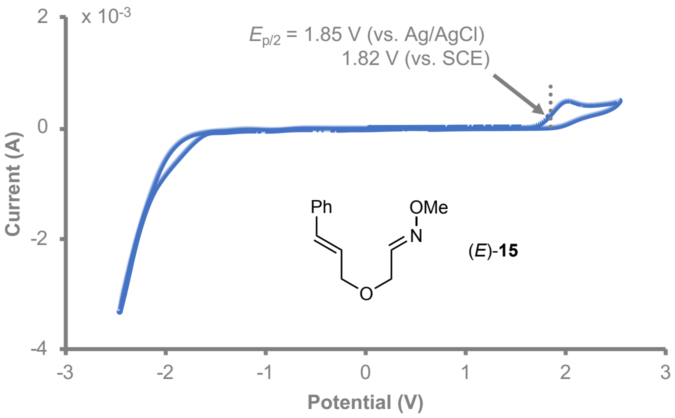

Supplementary Figure 1. Cyclic voltammogram of compound (*E*)-**15**.

The cyclic voltammogram shows an irreversible oxidation process with $E_{p/2}$ = +1.82 V (vs. SCE). [Ir(dF(CF_3)ppy)$_2$(dtbbpy)]PF_6 (**17**•PF_6) ($E_{1/2}^{III*/II}$ = +1.21 V vs. SCE)[4] does not possess an excited state oxidation potential sufficient to oxidize (*E*)-**15**, thus, a photoredox process is unlikely.

UV/Vis Absorption Spectra

UV/Vis absorption spectra were recorded on a Shimadzu UV-1601 UV/Vis spectrometer. Samples were prepared in THF with substrate (*E*)-**15** (10 mM) and photocatalyst **17**•PF_6 (0.05 mM). The photocatalyst is the only species absorbing at 427 nm.

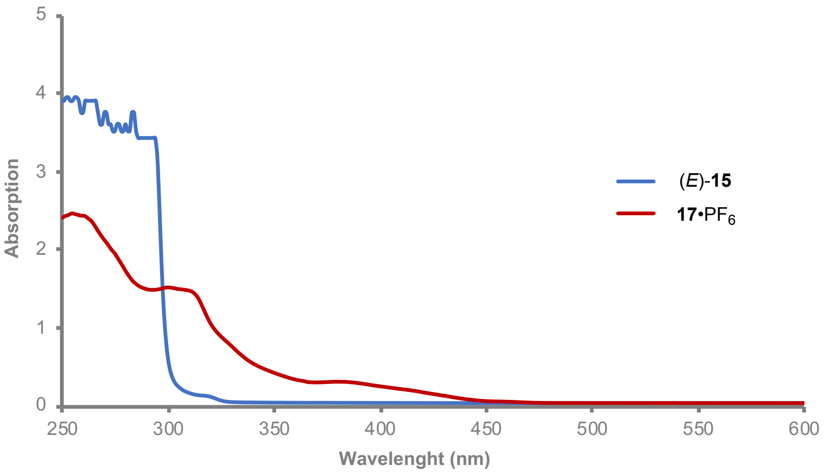

Supplementary Figure 2. UV/Vis spectra of (*E*)-**15** and **17•**PF_6; blue line = (*E*)-**15**; red line = **17•**PF_6.

Stern-Volmer Quenching Studies

All samples were prepared using stock solutions of **17•**PF_6 (0.11 mM), (*E*)-**15** (102.9 mM), (*E*)-**44** (144.8 mM) or **45** (102.4 mM) in dry MeCN. To a volumetric flask was added **17•**PF_6 (190 µL) and the respective amount of quencher and the volume adjusted to 4 mL with dry MeCN. The solution was transferred to a 1-cm quartz cuvette and degassed by sparging with nitrogen gas for 15 min. Emission spectra were recorded using a PTI QuantaMaster fluorimeter (Horiba) with an excitation wavelength of 420 nm. The emission intensities for the Stern-Volmer analysis were observed at 471 nm. The ratio of I_0/I was plotted as a function of the quencher concentration (I_0: emission intensity of **17•**PF_6 without quencher; I: emission intensity of **17•**PF_6 in the presence of quencher). The Stern-Volmer analysis shows that **17•**PF_6 is only efficiently quenched by the styrene moiety in (*E*)-**15** or **44**, while the oxime moiety (**45**) does not quench the photocatalyst. Although the triplet state energies of some aromatic oximes are close to the triplet energy of **17•**PF_6, we attribute the lack of quenching of **45** to the fact that the oxime moiety lacks an aromatic substituent.

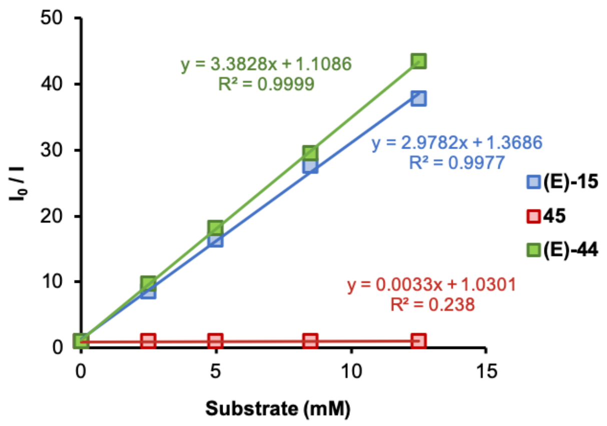

Supplementary Figure 3. Stern-Volmer quenching study of **17•**PF_6. The data was fitted through linear regression: (*E*)-**15** ($y = 2.9782x + 1.3686$; $R^2 = 0.9977$); **45** ($y = 0.0033x + 1.0301$; $R^2 = 0.238$); (*E*)-**44** ($y = 3.2828x + 1.1086$; $R^2 = 0.9999$); blue squares = (*E*)-**15**; red squares = **45**; green squares = (*E*)-**44**.

NMR Time Study

A 1-dram vial was charged with (*E*)-**15** (6.2 mg, 0.03 mmol, 1.0 equiv.), **17•**PF_6 (0.2 mg, 0.5 mol%), dimethyl terephthalate (4.7 mg) and d_3-MeCN (3 mL). 1 mL of the resulting solution was transferred to a NMR tube, which was placed in front of a 40 W PR160-427 nm Kessil light (~5 cm distance; 50% intensity) and the solution irradiated under ambient atmosphere. Conversion and yield were determined at several time points by quantitative ^{1}H NMR using dimethyl terephthalate as internal standard. The time study shows that styrene *E*/*Z* isomerization occurs at a similar rate as productive formation of **16**, however, both (*E*)- and (*Z*)-**15** eventually are converted to **16** (Fig. 4A). Additionally, the observed oxime *E*/*Z* isomerization at low conversion indicates reversible *C*–*C* bond formation from the triplet styrene, which generates a 1,4-biradical that can freely rotate around the *C*–*N* bond. In contrast, no oxime *E*/*Z* isomerization was observed for compound **45** lacking the styrene moiety.

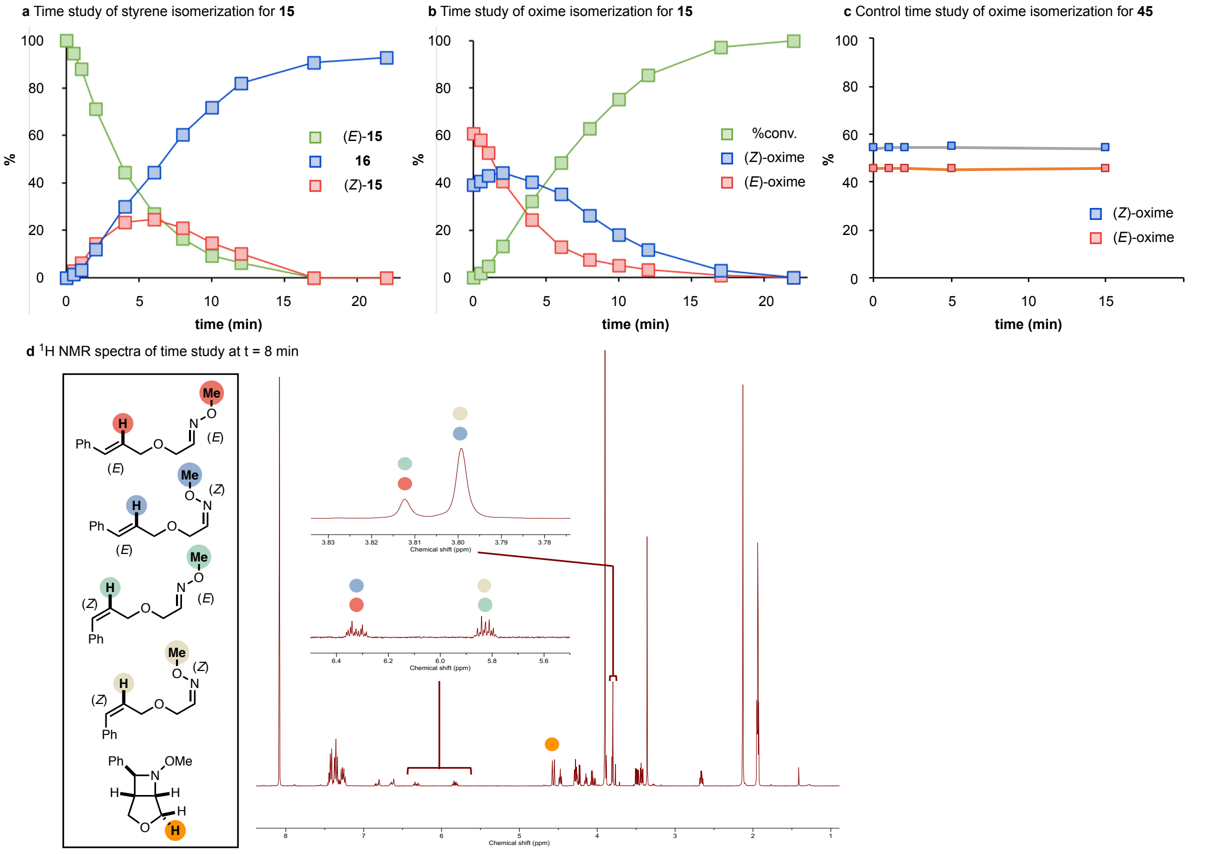

Supplementary Figure 4. Time study of the [2+2] cycloaddition of **15** monitored by ^{1}H NMR. **a** time study of styrene isomerization (sum of oxime isomers was used for integration); green squares = (*E*)-**15**; blue squares = **16**; red squares = (*Z*)-**15**; **b** time study of oxime isomerization (sum of styrene isomers was used for integration); green squares = %conversion of **15**.; blue squares = (*Z*)-oxime isomer; red squares = (*E*)-oxime isomer; **c** control time study of oxime isomerization of compound **45**; blue squares = (*Z*)-oxime isomer; red squares = (*E*)-oxime isomer; **d** ^{1}H NMR spectrum of time point t = 8 min highlighting the signals utilized for the time study.

Control Reactions

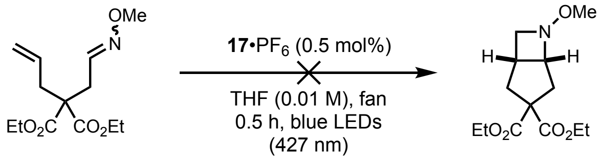

50 **51**, not observed

Reaction was carried out according to GP-6a on 0.25 mmol scale. No formation of azetidine **51** was observed, and only unreacted starting material (**50**) was isolated from the reaction mixture. For an overview of additional alkenes that would result in an endergonic triplet energy transfer process (ΔE_T >0), see references 5 and 6.

Rationale for Observed Diastereoselectivity

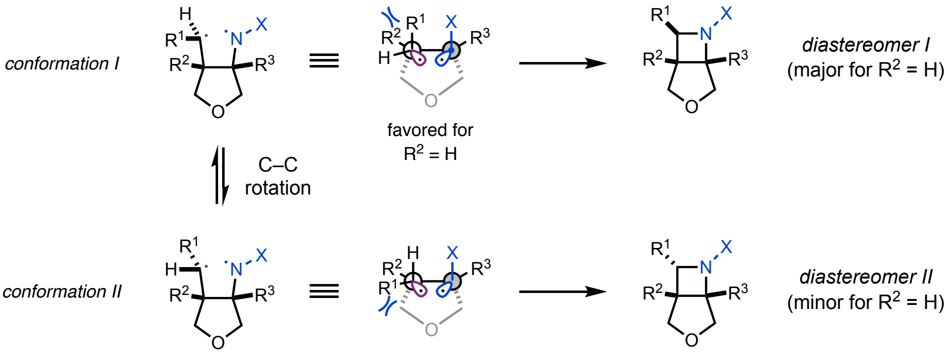

The observed relative stereoselectivity in the developed aza Paternò-Büchi reaction can be rationalized through analysis of the conformation of the biradical intermediate prior final C–C bond formation. Generally, the conformation leading to the major diastereomer (conformation I) avoids steric interactions between the alkene residue (R^1) and cyclic backbone of the substrate. In particular, small substituents on the alkene moiety (R^2 = H) achieve excellent diastereoselectivity, while larger substituents (R^2 = Me) lead to an erosion of selectivity due to additional steric interactions between R^1 and R^2. This effect is smaller for remote substituents (R^3), for which we observed a correlation between the selectivity and size of the substituent (R^3 = H > Me > Ph). Finally, larger alkene residues (R^1 = Ph) provide greater levels of stereoselectivity, which can be rationalized through increased steric interactions between R^1 and the backbone of the substrate, further disfavoring conformation II. In contrast, substrates bearing a diene (R^2 = vinyl) proceed with lower diastereoselectivity as a result of the smaller size of the vinyl group.

General Procedure for Alkylation of Cinnamyl Alcohols (GP-1)

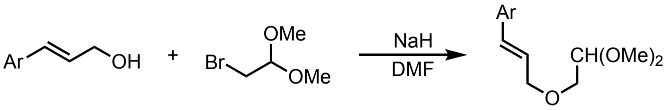

A round-bottom flask equipped with a magnetic stir bar was charged with NaH (60% dispersion in mineral oil; 1.5 equiv.) and dry DMF (0.5 M). The mixture was cooled to 0 °C and the corresponding cinnamyl alcohol was added slowly and the solution stirred

for 1 h. Next, 2-bromo-1,1-dimethoxyethane (2.0 equiv.) was added and the reaction heated at 110°C for 24 h. NH_4Cl (aq., sat.) and water were sequentially added, the organic layer was separated and the aqueous layer extracted with EtOAc (3x). The combined organic layers were washed with water (2x) and brine (2x), dried over Na_2SO_4, filtered and concentrated *in vacuo*. The crude product was purified by flash column chromatography (EtOAc/hexanes) to afford the corresponding pure alkylated cinnamyl alcohol.

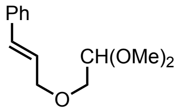

(*E*)-(3-(2,2-Dimethoxyethoxy)prop-1-en-1-yl)benzene (SM-a): Prepared according to GP-1 from (*E*)-cinnamyl alcohol (37.3 mmol). Purification by flash column chromatography (5-15% EtOAc/hexanes) afforded the pure title compound as yellow oil (2.90 g, 35%). **^{1}H NMR** (700 MHz, $CDCl_3$): δ 7.39 (d, *J* = 7.4 Hz, 2H), 7.32 (t, *J* = 7.6 Hz, 2H), 7.24 (t, *J* = 7.3 Hz, 1H), 6.61 (d, *J* = 15.9 Hz, 1H), 6.29 (dt, *J* = 15.9, 6.2 Hz, 1H), 4.56 (t, *J* = 5.2 Hz, 1H), 4.21 (dd, *J* = 6.2, 1.2 Hz, 2H), 3.54 (d, *J* = 5.2 Hz, 2H), 3.41 (s, 6H); **^{13}C NMR** (176 MHz, $CDCl_3$): δ 136.7, 133.0, 128.7, 127.9, 126.7, 125.9, 102.9, 72.3, 69.8, 54.1; **IR** (cm^{-1}): 2936, 2834, 1724, 1450, 1366, 1312, 1194, 1111, 1067, 966, 841, 750, 697; **HRMS**: *m*/*z* calculated for $C_{13}H_{18}O_3Na^+$ $[M+Na]^+$: 245.1148; found: 245.1163.

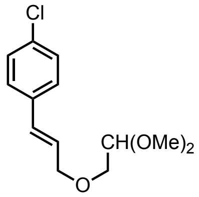

(*E*)-1-Chloro-4-(3-(2,2-dimethoxyethoxy)prop-1-en-1-yl)benzene (A20): Prepared according to GP-1 from (*E*)-4-chlorocinnamyl alcohol[7] (5.7 mmol). Purification by flash column chromatography (5-20% EtOAc/hexanes) afforded the pure title compound as yellow oil (482 mg, 33%). **^{1}H NMR** (500 MHz, $CDCl_3$): δ 7.31 (d, *J* = 8.7 Hz, 2H), 7.28 (d, *J* = 8.8 Hz, 2H), 6.56 (d, *J* = 15.9 Hz, 1H), 6.26 (dt, *J* = 15.9, 6.0 Hz, 1H), 4.55 (t, *J* = 5.2 Hz, 1H), 4.19 (dd, *J* = 6.0, 1.3 Hz, 2H), 3.54 (d, *J* = 5.2 Hz, 2H), 3.41 (s, 6H); **^{13}C NMR** (126 MHz, $CDCl_3$): δ 135.3, 133.5, 131.6, 128.9, 127.8, 126.6, 102.9, 72.1, 70.0, 54.1; **IR** (cm^{-1}): 2909, 2831, 1491, 1447, 1193, 1112, 1090, 1012, 967, 849, 797; **HRMS**: *m*/*z* calculated for $C_{13}H_{17}ClO_3Na^+$ $[M+Na]^+$: 279.0758; found: 279.0760.

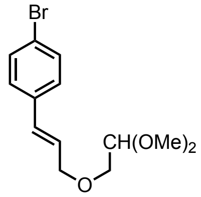

(*E*)-1-Bromo-4-(3-(2,2-dimethoxyethoxy)prop-1-en-1-yl)benzene (A21): Prepared according to GP-1 from (*E*)-4-bromocinnamyl alcohol[8] (4.7 mmol). Purification by flash column chromatography (5-20% EtOAc/hexanes) afforded the pure title compound as yellow oil (433 mg, 31%). **^{1}H NMR** (400 MHz, $CDCl_3$): δ 7.43 (d, *J* = 8.4 Hz, 2H), 7.24 (d, *J* = 8.5 Hz, 2H), 6.55 (d, *J* = 15.9 Hz, 1H), 6.28 (dt, *J* = 15.9, 6.0 Hz, 1H), 4.55 (t, *J* = 5.2 Hz, 1H), 4.19 (dd, *J* = 6.0, 1.2 Hz, 2H), 3.54 (d, *J* = 5.2 Hz, 2H), 3.41 (s, 6H); **^{13}C NMR** (176 MHz MHz, $CDCl_3$): δ 135.7, 131.8, 131.7, 128.2, 126.8, 121.7, 102.9, 72.1, 70.0, 54.1; **IR** (cm^{-1}): 2928, 2831, 1488, 1401, 1323, 1201, 1114, 1072, 1009, 969, 848; **HRMS**: *m*/*z* calculated for $C_{13}H_{17}BrO_3Na^+$ $[M+Na]^+$: 323.0253; found: 323.0252.

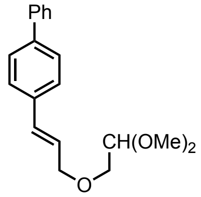

(*E*)-4-(3-(2,2-Dimethoxyethoxy)prop-1-en-1-yl)-1,1'-biphenyl (A22): Prepared according to GP-1 from (*E*)-4-phenylcinnamyl alcohol[8] (5.6 mmol). Purification by flash column chromatography (5-20% EtOAc/hexanes) afforded the pure title compound as pale-yellow foam (624 mg, 38%). **^{1}H NMR** (700 MHz, $CDCl_3$): δ 7.60 (d, *J* = 7.3 Hz, 2H), 7.56 (d, *J* = 8.2 Hz, 2H), 7.48 – 7.42 (m, 4H), 7.34 (t, *J* = 7.4 Hz, 1H), 6.65 (d, *J* = 15.9 Hz, 1H), 6.34 (dt, *J* = 15.9, 6.2 Hz, 1H), 4.57 (t, *J* = 5.2 Hz, 1H), 4.23 (dd, *J* = 6.1, 1.0 Hz, 2H), 3.56 (d, *J* = 5.2 Hz, 2H), 3.42 (s, 6H); **^{13}C NMR** (176 MHz, $CDCl_3$): δ 140.7, 140.6, 135.7, 132.5, 128.9, 127.4, 127.4, 127.1, 127.0, 125.9, 102.9, 72.3, 69.8, 54.1; **IR** (cm^{-1}): 2915, 2832, 1487, 1449, 1408, 1364, 1193, 1109, 1077, 971, 911, 853, 756, 731, 695; **HRMS**: *m*/*z* calculated for $C_{19}H_{22}O_3Na^+$ $[M+Na]^+$: 321.1461; found: 321.1466.

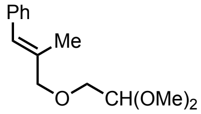

(*E*)-(3-(2,2-dimethoxyethoxy)-2-methylprop-1-en-1-yl)benzene (A26): Prepared according to GP-1 from (*E*)-2-methyl-3-phenylprop-2-en-1-ol (6.8 mmol). Purification by flash column chromatography (5-20% EtOAc/hexanes) afforded the pure title compound as yellow oil (530 mg, 33%). **^{1}H NMR** (500 MHz, $CDCl_3$): δ 7.33 (t, *J* = 7.6 Hz, 2H), 7.28 (d, *J* = 7.5 Hz, 2H), 7.22 (t, *J* = 7.2 Hz, 1H), 6.50 (s, 1H), 4.56 (t, *J* = 5.2 Hz, 1H), 4.09 (s, 2H), 3.52 (d, *J* = 5.2 Hz, 2H), 3.42 (s, 6H), 1.90 (s, 3H); **^{13}C NMR**

(126 MHz, $CDCl_3$): δ 137.6, 135.0, 129.0, 128.2, 127.5, 126.6, 103.0, 77.8, 69.5, 54.0, 15.5; **IR** (cm^{-1}): 2911, 2831, 1445, 1358, 1110, 1072, 964, 918, 855, 746, 699; **HRMS**: *m/z* calculated for $C_{14}H_{20}O_3Na^+$ $[M+Na]^+$: 259.1305; found: 259.1308.

Miscellaneous Procedures

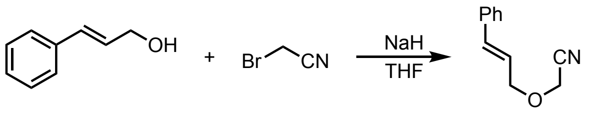

2-(Cinnamyloxy)acetonitrile (SM-b): A 100-mL round-bottom flask equipped with a stir bar was charged with NaH (60% dispersion in mineral oil; 1.79 g, 44.7 mmol, 1.2 equiv.) and THF (35 mL). The mixture was cooled to 0 °C and a solution of cinnamyl alcohol (5.00 g, 37.3 mmol, 1.0 equiv.) in THF (5 mL) was added dropwise. After stirring for 30 min at 0 °C, bromoacetonitrile (3.1 mL, 44.7 mmol, 1.2 equiv.) was added and the reaction mixture allowed to warm up to rt and stirred overnight. Then, NH_4Cl (aq., sat.) was added and the biphasic mixture partitioned between water and EtOAc. The organic layer was separated and the aqueous layer extracted with EtOAc (3x). The combined organic layers were washed with brine, dried over Na_2SO_4 filtered and concentrated *in vacuo*. Purification by flash column chromatography (5-10% EtOAc/hexanes) afforded the pure title compound as pale-yellow oil (3.23 g, 50%). **^{1}H NMR** (700 MHz, $CDCl_3$): δ 7.41 (d, J = 7.4 Hz, 2H), 7.34 (t, J = 7.6 Hz, 2H), 7.28 (t, J = 7.3 Hz, 1H), 6.70 (d, J = 15.9 Hz, 1H), 6.23 (dt, J = 15.9, 6.4 Hz, 1H), 4.31 (dd, J = 6.5, 1.2 Hz, 2H), 4.29 (s, 2H); **^{13}C NMR** (176 MHz, $CDCl_3$): δ 136.0, 135.4, 128.8, 128.4, 126.8, 123.2, 116.1, 71.8, 54.8; **IR** (cm^{-1}): 3027, 2861, 1494, 1450, 1352, 1091, 967, 883, 744, 692; **HRMS**: *m/z* calculated for $C_{11}H_{11}NONa^+$ $[M+Na]^+$: 196.0733; found: 196.0730.

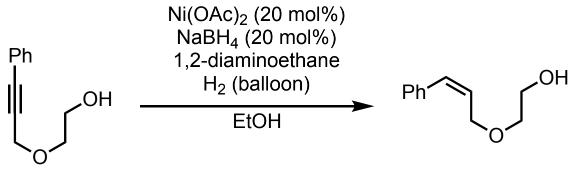

(*Z*)-2-((3-Phenylallyl)oxy)ethan-1-ol (A15): A 25-mL round-bottom flask equipped with a stir bar was charged with nickel(II) acetate tetrahydrate (71 mg, 0.28 mmol, 0.2 equiv.), sodium borohydride (11 mg, 0.28 mmol, 0.2 equiv.) and EtOH (4 mL). The mixture was sparged with hydrogen gas from a balloon and stirred for 1 h at rt. A solution of 2-((3-phenylprop-2-yn-1-yl)oxy)ethan-1-ol[9] (250 mg, 1.4 mmol, 1.0 equiv.) and 1,2-diaminoethane (38 µL, 0.57 mmol, 0.4 equiv.) in EtOH (1 mL) was added and the reaction stirred for 5.5 h at rt under an atmosphere of hydrogen. The mixture was filtered through celite and the filtrate concentrated *in vacuo*. Purification by flash column chromatography (30% EtOAc/hexanes) afforded the pure title compound as

clear oil (207 mg, 82%). Spectroscopic data were consistent with those reported in the literature.[10]

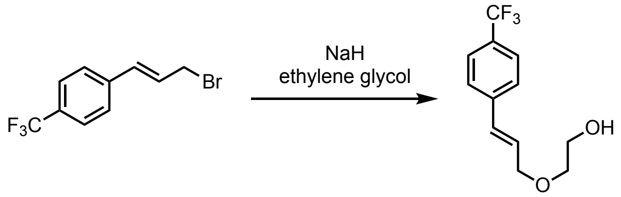

(*E*)-2-((3-(4-(Trifluoromethyl)phenyl)allyl)oxy)ethan-1-ol (A23): In a 25-mL round-bottom flask equipped with a magnetic stir bar, NaH (60% dispersion in mineral oil; 148 mg, 3.7 mmol, 1.2 equiv.) was added to a solution of ethylene glycol (0.31 mL, 5.6 mmol, 1.9 equiv.) in THF (8 mL) at 0 °C and the mixture stirred for 0.5 h at that temperature, before adding 4-(trifluoromethyl)cinnamyl bromide[11] (800 mg, 3.0 mmol, 1.0 equiv.) as solution in THF (2 mL) dropwise. Then, the reaction mixture was allowed to warm up to rt and heated at reflux overnight. After cooling down to rt, NH_4Cl (aq., sat.) was added and the biphasic mixture partitioned between water and EtOAc. The organic layer was separated and the aqueous layer extracted with EtOAc (3x). The combined organic layers were washed with brine, dried over Na_2SO_4, filtered and concentrated *in vacuo*. Purification by flash column chromatography (5-50% EtOAc/hexanes) afforded the pure title compound as clear oil (470 mg, 64%). **^{1}H NMR** (700 MHz, $CDCl_3$): δ 7.57 (d, *J* = 8.2 Hz, 2H), 7.48 (d, *J* = 8.2 Hz, 2H), 6.66 (d, *J* = 16.0 Hz, 1H), 6.39 (dt, *J* = 16.0, 5.8 Hz, 1H), 4.23 (dd, *J* = 5.8, 1.4 Hz, 2H), 3.80 (t, *J* = 4.6 Hz, 2H), 3.64 (t, *J* = 4.6 Hz, 2H); **^{13}C NMR** (176 MHz, $CDCl_3$): δ 140.2, 131.0, 129.7 (q, *J* = 32.4 Hz), 128.7, 126.8, 125.7 (q, *J* =3.8 Hz), 124.3 (q, *J* = 271.4 Hz), 71.7, 71.6, 62.0; **IR** (cm^{-1}): 3396, 2860, 1615, 1322, 1162, 1106, 1065, 1016, 968, 852, 730; **HRMS**: *m/z* calculated for $C_{12}H_{13}F_3O_2Na^+$ $[M+Na]^+$: 269.0760; found: 269.0762.

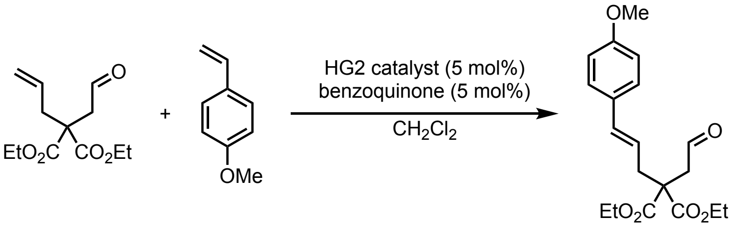

Diethyl (*E*)-2-(3-(4-methoxyphenyl)allyl)-2-(2-oxoethyl)malonate (A24): A 25-mL round-bottom flask equipped with a magnetic stir bar was charged with diethyl 2-allyl-2-(2-oxoethyl)malonate[12] (300 mg, 1.2 mmol, 1.0 equiv.) and CH_2Cl_2 (2.5 mL). Hoveyda-Grubbs catalyst (2nd generation) (39 mg, 0.06 mmol, 5 mol%), 4-methoxystyrene (332 mg, 2.5 mmol, 2.0 eq.), and benzoquinone (7 mg, 0.06 mmol, 5 mol%) were added sequentially and the reaction was heated at reflux for 16 h. After cooling to room temperature, the solvent was removed *in vacuo* and the crude product purified by flash column chromatography (2-30% EtOAc/hexanes) to afford the pure title compound as a yellow oil (220 mg, 55%). **^{1}H NMR** (500 MHz, CD_2Cl_2): δ 9.70 (s,

1H), 7.26 (d, J = 8.6 Hz, 2H), 6.83 (d, J = 8.7 Hz, 2H), 6.37 (d, J = 15.7 Hz, 1H), 5.90 (dt, J = 15.5, 7.6 Hz, 1H), 4.27 – 4.12 (m, 4H), 3.78 (s, 3H), 2.96 (s, 2H), 2.86 (d, J = 7.6 Hz, 2H), 1.25 (t, J = 7.1 Hz, 6H); **^{13}C NMR** (126 MHz, CD_2Cl_2): δ 199.6, 170.8, 159.9, 134.6, 130.2, 127.9, 121.8, 114.4, 62.5, 55.8, 55.7, 46.8, 38.2, 14.4; **IR** (cm^{-1}): 2981, 2839, 1723, 1607, 1511, 1247, 1190, 1176, 1094, 1031, 972, 843; **HRMS**: *m*/*z* calculated for $C_{19}H_{24}O_6Na^+$ $[M+Na]^+$: 371.1465; found: 371.1465.

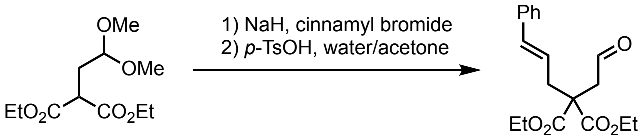

Diethyl 2-cinnamyl-2-(2-oxoethyl)malonate (A28): A 25-mL round-bottom flask equipped with a magnetic stir bar was charged with NaH (60% dispersion in mineral oil; 193 mg, 4.8 mmol, 1.2 equiv.) and THF (13 mL). After cooling the mixture to 0 °C, diethyl 2-(2,2-dimethoxyethyl)malonate[13] (1.00 g, 4.0 mmol, 1.0 equiv.) was added dropwise and the solution allowed to warm up to rt and stirred for 1 h. The solution was cooled to 0 °C, cinnamyl bromide (1.19 g, 6.0 mmol, 1.5 equiv.) was added subsequently and the solution allowed to warm up to rt and stirred overnight. Then, NH_4Cl (aq., sat.) was added and the biphasic mixture partitioned between water and Et_2O. The organic layer was separated and the aqueous layer extracted with Et_2O (3x). The combined organic layers were dried over $MgSO_4$, filtered, concentrated *in vacuo* and dried using high-vac.

The crude acetal was dissolved in a 1:1 mixture (*v*/*v*) of water/acetone (40 mL). *p*-TsOH (153 mg, 0.8 mmol, 0.2 equiv.) was added and the reaction heated at 85 °C for 2 h. After cooling down to rt, $NaHCO_3$ (aq., sat.) was added and the mixture extracted with EtOAc (3x). The combined organic layers were dried over Na_2SO_4, filtered and concentrated *in vacuo*. Purification by flash column chromatography (20% EtOAc/hexanes) afforded the pure title compound as colorless oil (950 mg, 74%). **^{1}H NMR** (500 MHz, $CDCl_3$): δ 9.73 (s, 1H), 7.34 – 7.27 (m, 4H), 7.25 – 7.21 (m, 1H), 6.43 (d, J = 15.7 Hz, 1H), 6.05 (dt, J = 15.5, 7.6 Hz, 1H), 4.24 (q, J = 7.1 Hz, 4H), 3.01 (s, 2H), 2.92 (d, J = 7.6 Hz, 2H), 1.27 (t, J = 7.1 Hz, 6H); **^{13}C NMR** (176 MHz, $CDCl_3$): δ 199.0, 170.1, 136.8, 134.9, 128.7, 127.8, 126.4, 123.6, 62.1, 55.2, 46.4, 37.7, 14.1; **IR** (cm^{-1}): 2982, 1721, 1446, 1367, 1188, 1093, 1020, 969, 860, 741, 693; **HRMS**: *m*/*z* calculated for $C_{18}H_{22}O_5Na^+$ $[M+Na]^+$: 341.1359; found: 341.1362.

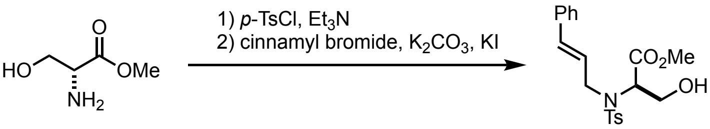

Methyl *N*-cinnamyl-*N*-tosyl-*D*-serinate (A1-29): A 100-mL round-bottom flask equipped with a magnetic stir bar was charged with *D*-serine methyl ester

hydrochloride (1.00 g, 6.43 mmol, 1.0 equiv.) and CH_2Cl_2 (30 mL), before adding tosyl chloride (1.76 g, 9.23 mmol, 1.4 equiv.) and triethylamine (2.93 mL, 21.0 mmol, 3.3 equiv.). After stirring at rt for 16 h, $NaHCO_3$ (aq., sat.) was added, the organic layer separated, and the aqueous layer extracted with CH_2Cl_2 (3x). The combined organic layers were washed with brine, dried over Na_2SO_4, filtered and concentrated *in vacuo*, then dried using high-vac.

The crude *N*-tosyl amine was dissolved in acetone (30 mL), before adding K_2CO_3 (1.74 g, 12.6 mmol, 2.0 equiv.), cinnamyl bromide (2.49 g, 12.6 mmol, 2.0 equiv.), and KI (140 mg, 0.84 mmol, 0.1 equiv.). The resulting mixture was stirred at 60 °C for 3 h. $NaHCO_3$ (aq., sat.) was added, the organic layer separated, and the aqueous layer extracted with Et_2O (3x). The combined organic layers were washed with brine, dried over $MgSO_4$, filtered and concentrated *in vacuo*. Purification by flash column chromatography (5-65% EtOAc/hexanes) afforded the pure title compound as pale-yellow oil (1.17 g, 47%). **^{1}H NMR** (700 MHz, $CDCl_3$): δ 7.73 (d, *J* = 8.2 Hz, 2H), 7.26 (m, 6H), 7.21 (m, 1H), 6.48 – 6.43 (m, 1H), 6.10 (dt, *J* = 15.9, 6.7 Hz, 1H), 4.62 (t, *J* = 6.4 Hz, 1H), 4.10 (dd, *J* = 16.1, 6.2 Hz, 1H), 4.06 (dd, *J* = 11.8, 6.1 Hz, 1H), 3.94 (dd, *J* = 16.0, 7.2 Hz, 1H), 3.86 (dd, *J* = 11.8, 6.8 Hz, 1H), 3.55 (s, 3H), 2.39 (s, 3H); **^{13}C NMR** (176 MHz, $CDCl_3$): δ 170.4, 143.8, 137.1, 136.1, 133.8, 129.7, 128.7, 128.2, 127.7, 126.6, 125.2, 61.5, 60.9, 52.5, 49.1, 21.7; **IR** (cm^{-1}): 3522, 2953, 1739,1336, 1290, 1246, 1155, 1089, 1038, 970, 731, 660; **HRMS**: *m*/*z* calculated for $C_{20}H_{23}NO_5SNH_4^+$ $[M+NH_4]^+$: 407.1635; found: 407.1635.

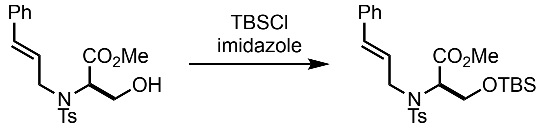

Methyl *O*-(*tert*-butyldimethylsilyl)-*N*-cinnamyl-*N*-tosyl-*D*-serinate (A2-29): A 100-mL round-bottom flask equipped with a magnetic stir bar was charged with **A1-29** (1.17 g, 3.00 mmol, 1.0 equiv.), imidazole (205 mg, 3.00 mmol, 1.0 equiv.), TBSCl (543 mg, 3.60 mmol, 1.2 equiv.) and CH_2Cl_2 (30.0 mL). After stirring for 16 h at rt, the $NaHCO_3$ (aq., sat.) was added and the organic layer separated. Then, the aqueous layer was extracted with CH_2Cl_2 (3x) and the combined organic layers washed with brine, dried over Na_2SO_4, filtered and concentrated *in vacuo*. Purification by flash chromatography (2-20% EtOAc/hexanes) afforded the pure title compound as clear oil (1.30 g, 86%). **^{1}H NMR** (400 MHz, $CDCl_3$): δ 7.72 (d, *J* = 8.2 Hz, 2H), 7.31 – 7.18 (m, 7H), 6.46 (d, *J* = 15.9 Hz, 1H), 6.22 – 6.07 (m, 1H), 4.71 (t, *J* = 5.5 Hz, 1H), 4.23 – 4.08 (m, 2H), 4.04 (d, *J* = 5.5 Hz, 2H), 3.57 (s, 3H), 2.37 (s, 3H), 0.82 (s, 9H), 0.00 (d, *J* = 3.9 Hz, 6H); **^{13}C NMR** (100 MHz, CD_2Cl_2): δ 170.3, 144.1, 138.2, 137.3, 132.6, 130.0, 129.0, 128.1, 128.0, 127.5, 126.9, 63.2, 61.9, 52.5, 49.1, 26.0, 21.8, 18.6, –5.3, –5.5; **IR** (cm^{-1}): 2952, 2929, 2884, 2856, 1743, 1342, 1254, 1157, 1093, 837, 813, 779,

753, 693, 657; **HRMS**: *m/z* calculated for $C_{26}H_{37}NO_5SSiNH_4^+$ $[M+NH_4]^+$: 521.2500; found: 521.2501.

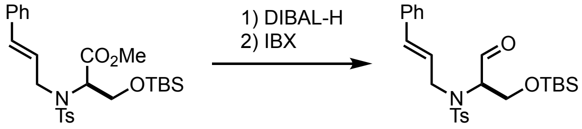

(*R*)-*N*-(1-((*tert*-butyldimethylsilyl)oxy)-3-oxopropan-2-yl)-*N*-cinnamyl-4-methylbenzenesulfonamide (A3-29): A 100-mL round-bottom flask equipped with a magnetic stir bar was charged with **A2-29** (1.17 g, 2.32 mmol, 1.0 equiv.) and CH_2Cl_2 (25 mL). After cooling the solution to –78 °C, a freshly prepared 1 M solution of DIBAL-H (0.91 mL, 5.11 mmol, 2.2 equiv.) in CH_2Cl_2 was added slowly over 12 min. After stirring for 0.5 h at –78 °C, the flask was transferred to an ice bath and allowed to warm to 0 °C. The mixture was diluted with diethyl ether (25 mL), then, water (0.2 mL) was added dropwise, followed by the sequential addition of 15% NaOH (aq., 0.2 mL) and water (0.5 mL). The mixture was allowed to warm to rt and stirred for 15 min. $MgSO_4$ was added and the mixture stirred for an additional 15 min. Solids were filtered off and the filtrate concentrated *in vacuo* to afford the corresponding crude alcohol.

The crude alcohol was dissolved in DMSO (20 mL), before adding IBX (971 mg, 3.47 mmol, 1.5 equiv.). The mixture was stirred at rt for 12 h, after which Et_2O (20 mL) and water (20 mL) were added. The mixture was filtered through celite, and the organic layer was separated from the biphasic filtrate. The aqueous layer was extracted with Et_2O (3x), and the combined organic layers were washed with brine, dried over $MgSO_4$, filtered and concentrated *in vacuo*. Purification by flash chromatography (2-20% EtOAc/hexanes) afforded the pure title compound as clear oil (343 mg, 31%). **^{1}H NMR** (500 MHz, $CDCl_3$): δ 9.68 (s, 1H), 7.77 (d, *J* = 8.3 Hz, 2H), 7.34 – 7.21 (m, 7H), 6.47 (d, *J* = 15.9 Hz, 1H), 6.12 (dt, *J* = 15.8, 6.8 Hz, 1H), 4.34 (dd, *J* = 7.3, 5.0 Hz, 1H), 4.20 – 4.05 (m, 3H), 4.02 (dd, *J* = 11.0, 7.4 Hz, 1H), 2.41 (s, 3H), 0.82 (s, 9H), 0.00 (d, *J* = 10.6 Hz, 6H); **^{13}C NMR** (100 MHz, $CDCl_3$): δ 198.8, 143.7, 137.5, 136.1, 134.4, 129.8, 128.7, 128.2, 127.6, 126.6, 125.0, 67.5, 61.0, 49.7, 25.8, 21.6, 18.2, –5.5, –5.6; **IR** (cm^{-1}): 2954, 2928, 2856, 1734, 1471, 1338, 1256, 1157, 1092, 837, 781, 751; **HRMS**: *m/z* calculated for $C_{25}H_{35}NO_4SSiNH_4^+$ $[M+NH_4]^+$: 491.2394; found: 491.2400.

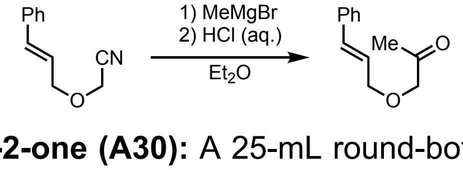

1-(Cinnamyloxy)propan-2-one (A30): A 25-mL round-bottom flask equipped with a magnetic stir bar was charged with **SM-b** (500 mg, 2.9 mmol, 1.0 equiv.) and THF (10 mL). After cooling to 0 °C, methylmagnesium bromide (3 M solution in Et_2O, 1.2 mL, 3.6 mmol, 1.2 equiv.) was added dropwise and the reaction mixture allowed

to warm up to rt and stirred for 2 h. Then, a 2 M aqueous solution of HCl (5 mL) was added at 0 °C, the mixture allowed to warm up to rt and stirred vigorously for 0.5 h. The biphasic mixture was diluted with water and EtOAc, the organic layer separated and the aqueous layer extracted with EtOAc (3x). The combined organic layers were washed with $NaHCO_3$ (aq., sat.), brine, dried over $MgSO_4$, filtered and concentrated *in vacuo*. Purification by flash column chromatography (5-15% EtOAc/hexanes) afforded the pure title compound as clear oil (328 mg, 60%). **^{1}H NMR** (700 MHz, $CDCl_3$): δ 7.39 (d, *J* = 7.5 Hz, 2H), 7.33 (t, *J* = 7.6 Hz, 2H), 7.26 (t, *J* = 7.8 Hz, 1H), 6.63 (d, *J* = 15.9 Hz, 1H), 6.29 (dt, *J* = 15.9, 6.2 Hz, 1H), 4.23 (dd, *J* = 6.2, 1.1 Hz, 2H), 4.11 (s, 2H), 2.18 (s, 3H); **^{13}C NMR** (176 MHz, $CDCl_3$): δ 206.8, 136.5, 133.7, 128.8, 128.1, 126.7, 125.1, 75.4, 72.1, 26.6; **IR** (cm^{-1}): 3027, 2852, 1729, 1716, 1495, 1449, 1354, 1119, 966, 733, 692; **HRMS**: *m*/*z* calculated for $C_{12}H_{14}O_2Na^+$ $[M+Na]^+$: 213.0886; found: 213.0893.

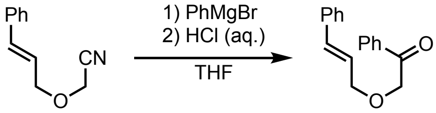

2-(Cinnamyloxy)-1-phenylethan-1-one (A31): A 50-mL round-bottom flask equipped with a magnetic stir bar was charged with freshly grinded magnesium turnings (105 mg, 4.3 mmol, 1.5 equiv.). THF (4 mL) and bromobenzene (0.48 mL, 4.6 mmol, 1.6 equiv.) were added sequentially and the mixture brought to reflux with a heat gun, then allowed to stir at rt for 2 h. After diluting the solution with THF (4 mL), CuBr (8 mg, 0.06 mmol, 0.02 equiv.) and **SM-b** (500 mg, 2.9 mmol, 1.0 equiv.) were added and the reaction heated at reflux for 0.5 h. Then, the mixture was cooled to 0 °C and 2 M aqueous HCl (5 mL) was added followed by vigorous stirring for 0.5 h. The biphasic mixture was partitioned between water and Et_2O, the organic layer separated and the aqueous layer extracted with Et_2O (3x). The combined organic layers were washed with $NaHCO_3$ (aq., sat.) and brine, dried over $MgSO_4$, filtered and concentrated *in vacuo*, then dried using high-vac. The crude phenyl ketone was used for the synthesis of **S31** without further purification.

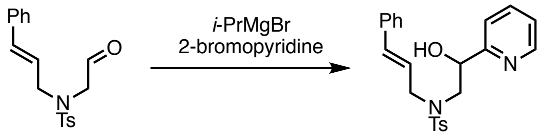

***N*-Cinnamyl-*N*-(2-hydroxy-2-(pyridin-2-yl)ethyl)-4-methylbenzenesulfonamide (A32):** A 25-mL round-bottom flask with a magnetic stir bar was charged with 2-bromopyridine (0.16 mL, 1.61 mmol, 1.0 equiv.) and THF (1 mL), and a 1 M solution of isopropylmagnesium bromide in THF (1.6 mL, 1.0 equiv.) was added over 5 minutes. After stirring for 2 h at rt, a solution of *N*-cinnamyl-*N*-(2-hydroxyethyl)-4-methylbenzenesulfonamide[14] (530 mg, 1.61 mmol, 1.0 equiv.) in THF (1 mL) was added dropwise. The mixture was stirred for 12 h at rt followed by addition of NH_4Cl

(aq., sat.). The organic layer was separated, and the aqueous layer extracted with diethyl ether (3x). The combined organic layers were washed with brine, dried over $MgSO_4$, filtered and concentrated *in vacuo*. Purification by flash column chromatography (10-70% EtOAc/hexanes) afforded the pure title compound as yellow oil (225 mg, 34%). **^{1}H NMR** (500 MHz, $CDCl_3$): δ 8.47 (d, *J* = 4.7 Hz, 1H), 7.74 (d, *J* = 8.3 Hz, 2H), 7.70 (td, *J* = 7.7, 1.7 Hz, 1H), 7.48 (d, *J* = 7.8 Hz, 1H), 7.32 – 7.27 (m, 4H), 7.25 – 7.13 (m, 4H), 6.37 (d, *J* = 15.9 Hz, 1H), 5.80 (dt, *J* = 15.8, 6.8 Hz, 1H), 5.02 (q, *J* = 5.0 Hz, 1H), 4.27 (d, *J* = 5.4 Hz, 1H), 4.03 (dd, *J* = 15.5, 5.8 Hz, 1H), 3.92 (dd, *J* = 15.5, 7.3 Hz, 1H), 3.51 (dd, *J* = 14.7, 4.5 Hz, 1H), 3.44 (dd, *J* = 14.8, 7.4 Hz, 1H), 2.41 (s, 3H); **^{13}C NMR** (126 MHz, $CDCl_3$): δ 159.5, 148.6, 143.7, 136.9, 136.8, 136.3, 134.4, 129.9, 128.6, 128.0, 127.6, 126.6, 123.7, 123.0, 121.7, 72.4, 54.2, 52.0, 21.6; **IR** (cm^{-1}): 3026, 2924, 1596, 1438, 1335, 1305, 1155, 1089, 969, 925, 815, 732, 693, 662; **HRMS**: *m/z* calculated for $C_{23}H_{24}N_2O_4SH^+$ $[M+H]^+$: 409.1580; found: 409.1578.

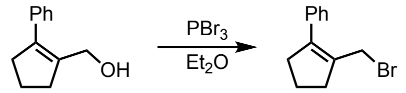

(2-(Bromomethyl)cyclopent-1-en-1-yl)benzene (A1-34): A 25-mL round-bottom flask equipped with a magnetic stir bar was charged with (2-phenylcyclopent-1-en-1-yl)methanol[15] (940 mg, 5.4 mmol, 1.0 equiv.) and Et_2O (6 mL). PBr_3 (0.21 mL, 2.2 mmol, 0.4 equiv.) was added dropwise at 0 °C and the reaction stirred for 1 h at that temperature. Then, brine (1 mL) was added and the aqueous layer separated. The organic layer was washed sequentially with $NaHCO_3$ (aq., sat., 3x) and brine, dried over $MgSO_4$, filtered and concentrated *in vacuo* and dried using high-vac to afford the crude title compound as clear oil (1.18 g, 92%), which was directly used for the next step without further purification.

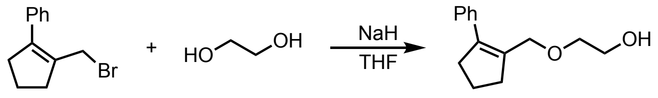

2-((2-Phenylcyclopent-1-en-1-yl)methoxy)ethan-1-ol (A2-34): NaH (60% dispersion in mineral oil; 498 mg, 12.4 mmol, 2.5 equiv.) was carefully added to a solution of ethylene glycol (2.8 mL, 49.8 mmol, 10.0 equiv.) in THF (10 mL) at 0 °C. After stirring for 15 min, the mixture was transferred to a 50-mL round-bottom flask equipped with a magnetic stir bar containing a solution of **A1-34** (1.18 g, 5.0 mmol, 1.0 equiv.) in THF (10 mL) and the resulting mixture refluxed for 4 h. After cooling down to rt, NH_4Cl (aq., sat.) was added and the biphasic mixture extracted with EtOAc (3x). The combined organic layers were washed with brine, dried over Na_2SO_4, filtered and concentrated *in vacuo*. Purification by flash column chromatography (5-50% EtOAc/hexanes) afforded the pure title compound as clear oil (869 mg, 80%). **^{1}H NMR** (700 MHz, $CDCl_3$): δ 7.34 (t, *J* = 7.6 Hz, 2H), 7.27 – 7.25 (m, 1H), 7.25 – 7.22 (m, 2H),

4.17 (s, 2H), 3.74 – 3.70 (m, 2H), 3.52 – 3.48 (m, 2H), 2.79 (t, J = 7.5 Hz, 2H), 2.65 (t, J = 7.5 Hz, 2H), 1.97 (p, J = 7.5 Hz, 2H), 1.91 (t, J = 6.2 Hz, 1H); **^{13}C NMR** (176 MHz, $CDCl_3$): δ 140.9, 137.8, 135.51, 128.3, 127.9, 127.1, 71.4, 68.1, 62.1, 37.9, 36.0, 22.1; **IR** (cm^{-1}): 3386, 2843, 1493, 1442, 1355, 1253, 1207, 1105, 1054, 1035, 1005, 889, 760, 732, 698, 655; **HRMS**: *m*/*z* calculated for $C_{14}H_{18}O_2^+$ [M]$^+$: 218.1307; found: 218.1312.

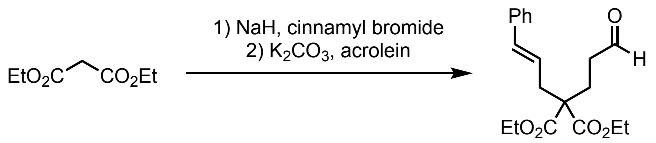

Diethyl 2-cinnamyl-2-(3-oxopropyl)malonate (A36): A round-bottom flask equipped with a magnetic stir bar was charged with NaH (60% dispersion in mineral oil; 457 mg, 11.4 mmol, 1.5 equiv.) and THF (15 mL). After cooling to 0 °C, diethyl malonate (1.7 mL, 11.4 mmol, 1.5 equiv.) was added dropwise and the reaction mixture stirred for 1 h at that temperature. Next, a solution of cinnamyl bromide (1.50 g, 7.6 mmol, 1.0 equiv.) in THF (2 mL) was added and the reaction mixture allowed to warm up to rt and stirred overnight. NH_4Cl (aq., sat.) and water were sequentially added, the organic layer separated and the aqueous layer extracted with EtOAc (3x). The combined organic layers were dried over Na_2SO_4, filtered and concentrated *in vacuo*. Excess diethyl malonate was removed under high-vac at 70 °C and the crude product was taken to the next step without further purification.

A 100-mL round-bottom flask equipped with a magnetic stir bar was charged with crude diethyl 2-cinnamylmalonate, K_2CO_3 (1.16 g, 8.4 mmol, 1.1 equiv.), acrolein (0.85 mL, 11.4 mmol, 1.5 equiv.) and CH_2Cl_2 (20 mL) and the mixture stirred at rt overnight. Water and EtOAc were added, the organic layer separated and the aqueous layer extracted with EtOAc (3x). The combined organic layers were washed with brine, dried over Na_2SO_4, filtered and concentrated *in vacuo*. Purification by flash column chromatography (20% EtOAc/hexanes) afforded the pure title compound as pale-yellow oil (914 mg, 36%). **^{1}H NMR** (500 MHz, $CDCl_3$): δ 9.74 (s, 1H), 7.33 – 7.27 (m, 4H), 7.24 – 7.20 (m, 1H), 6.45 (d, J = 15.7 Hz, 1H), 6.04 (dt, J = 15.5, 7.5 Hz, 1H), 4.26 – 4.15 (m, 4H), 2.80 (d, J = 7.5 Hz, 2H), 2.53 (t, J = 7.8 Hz, 2H), 2.24 (t, J = 8.1 Hz, 2H), 1.26 (t, J = 7.1 Hz, 6H); **^{13}C NMR** (176 MHz, $CDCl_3$): δ 200.9, 170.9, 137.0, 134.3, 128.6, 127.6, 126.3, 123.6, 61.6, 57.0, 39.3, 37.5, 25.4, 14.2; **IR** (cm^{-1}): 2981, 1721, 1446, 1367, 1233, 1184, 1095, 1025, 968, 858, 739, 693; **HRMS**: *m*/*z* calculated for $C_{19}H_{24}O_5Na^+$ [M+Na]$^+$: 355.1516; found: 355.1518.

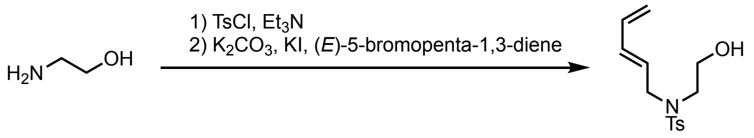

(*E*)-*N*-(2-Hydroxyethyl)-4-methyl-*N*-(penta-2,4-dien-1-yl)benzenesulfonamide (A42): Ethanolamine (0.25 mL, 4.1 mmol, 1.0 equiv.) was converted to *N*-(2-hydroxyethyl)-4-toluenesulfonamide following a literature procedure[14] and the crude product used for the next step without further purification.

A 25-mL round-bottom flask equipped with a magnetic stir bar was charged with crude *N*-(2-hydroxyethyl)-4-toluenesulfonamide and acetone (10 mL), followed by the sequential addition of K_2CO_3 (859 mg, 6.2 mmol, 1.5 equiv.), KI (69 mg, 0.41 mmol, 0.1 equiv.) and freshly prepared (*E*)-5-bromopenta-1,3-diene[16] (913 mg, 6.2 mmol, 1.5 equiv.). The reaction was set to reflux and stirred overnight. Then, the reaction mixture was diluted with water and EtOAc, the organic layer separated and the aqueous layer extracted with EtOAc (3x). The combined organic layers were washed with brine, dried over Na_2SO_4, filtered and concentrated *in vacuo*. Purification by flash column chromatography (30-50% EtOAc/hexanes) afforded the pure title compound as pale-yellow oil (994 mg, 85%; *E*/*Z* (diene) = 15:1). **^{1}H NMR** (700 MHz, $CDCl_3$): δ 7.71 (d, *J* = 8.3 Hz, 2H), 7.32 (d, *J* = 7.9 Hz, 2H), 6.25 (dt, *J* = 17.0, 10.3 Hz, 1H), 6.11 (dd, *J* = 15.2, 10.5 Hz, 1H), 5.51 (dt, *J* = 15.0, 6.8 Hz, 1H), 5.18 (d, *J* = 17.0 Hz, 1H), 5.11 (d, *J* = 10.1 Hz, 1H), 3.89 (d, *J* = 6.8 Hz, 2H), 3.73 (t, *J* = 5.3 Hz, 2H), 3.24 (t, *J* = 5.3 Hz, 2H), 2.44 (s, 3H); **^{13}C NMR** (176 MHz, $CDCl_3$): δ 143.7, 136.4, 135.7, 135.1, 129.9, 127.9, 127.4, 118.6, 61.2, 51.3, 49.8, 21.6; **IR** (cm^{-1}): 3510, 2925, 1598, 1447, 1329, 1152, 1047, 1003, 950, 909, 814, 752, 726, 657; **HRMS**: *m*/*z* calculated for $C_{14}H_{19}NO_3SNa^+$ $[M+Na]^+$: 304.0978; found: 304.0982.

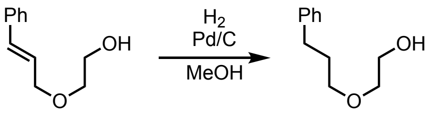

2-(3-Phenylpropoxy)ethan-1-ol (A45): A 100-mL round-bottom flask equipped with a magnetic stir bar was charged with 2-(cinnamyloxy)ethan-1-ol[17] (1.00 g, 5.6 mmol, 1.0 equiv.), palladium on activated carbon (10 wt%; 299 mg, 5 mol%) and MeOH (30 mL). The mixture was sparged for 20 min with hydrogen gas and stirred at rt for 2 h under a hydrogen atmosphere. Then, the reaction mixture was passed through a pad of celite. After washing the pad with EtOAc, the filtrate was concentrated *in vacuo* and dried using high-vac to obtain the pure title compound as clear oil (1.00 g, 99%). **^{1}H NMR** (700 MHz, $CDCl_3$): δ 7.29 (t, *J* = 7.6 Hz, 2H), 7.21 – 7.18 (m, 3H), 3.73 (q, *J* = 5.4 Hz, 2H), 3.53 (t, *J* = 4.5 Hz, 2H), 3.49 (t, *J* = 6.4 Hz, 2H), 2.70 (t, *J*= 7.8 Hz, 2H), 1.96 – 1.90 (m, 3H); **^{13}C NMR** (176 MHz MHz, $CDCl_3$): δ 141.9, 128.54, 128.46, 125.9, 71.9, 70.5, 62.0, 32.4, 31.3; **IR** (cm^{-1}): 3418, 2927, 2862, 1496, 1453, 1361, 1118,

1044, 891, 745, 698; **HRMS**: *m/z* calculated for $C_{11}H_{16}O_2Na^+$ $[M+Na]^+$: 203.1043; found: 203.1037.

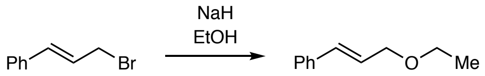

(*E*)-(3-ethoxyprop-1-en-1-yl)benzene (44): A 50-mL round-bottom flask equipped with a magnetic stir bar was charged with NaH (60% dispersion in mineral oil; 507 mg, 12.7 mmol, 2.5 equiv.) and THF (15 mL). After cooling to 0 ºC, EtOH (0.74 mL, 12.7 mmol, 2.5 equiv.) was added dropwise and the mixture stirred for 0.5 h. Next, a solution of cinnamyl bromide (1.00 g, 5.1 mmol, 1.0 equiv.) in THF (2.5 mL) was added at 0 ºC, and the mixture heated at reflux for 2 h. After cooling down to rt, NH_4Cl (aq., sat.) was added and the resulting mixture partitioned between water and EtOAc. The organic layer was separated and the aqueous layer extracted with EtOAc (3x). The combined organic layers were washed with brine, dried over MgSO4, filtered and concentrated *in vacuo*. Purification by flash column chromatography (2.5% Et_2O/pentane) provided the pure title compound as clear oil (452 mg, 52%). Spectroscopic data were consistent with those reported in the literature.[18]

General Procedure for Oxime Synthesis from Acetals (GP-2)

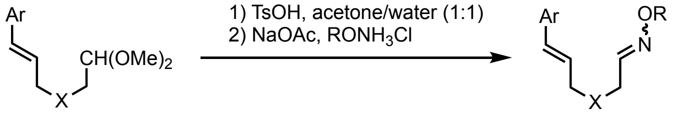

A 100-mL round-bottom flask equipped with a magnetic stir bar was charged with the corresponding acetal (1.0 equiv.) and a 1:1 mixture (*v*/*v*) of water/acetone (0.1 M). *p*-TsOH (0.2 equiv.) was added and the reaction heated at 85 °C until complete as determined by TLC analysis (4-8 h). After cooling down to rt, $NaHCO_3$ (aq., sat.) was added and the mixture extracted with EtOAc (3x). The combined organic layers were dried over Na_2SO_4, filtered, concentrated *in vacuo* and dried using high-vac.

The crude aldehyde was dissolved in CH_2Cl_2 (0.1 M), before adding NaOAc (4.0 equiv.) and the corresponding hydroxylamine hydrochloride (2.0 equiv.). The mixture was stirred at rt until complete as judged by TLC analysis (4-18 h). $NaHCO_3$ (aq., sat.) was added, the organic layer separated and the aqueous layer extracted with EtOAc (3x). The combined organic layers were washed with brine, dried over Na_2SO_4, filtered and concentrated *in vacuo*. Purification by flash column chromatography (EtOAc/hexanes) afforded the corresponding pure oxime as mixture of *E*/*Z* oxime isomers.

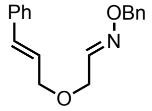

2-(Cinnamyloxy)acetaldehyde *O*-benzyl oxime (18a): Prepared according to GP-2 from **SM-a** (500 mg, 2.3 mmol, 1.0 equiv.) and *O*-benzylhydroxylamine hydrochloride (718 mg, 4.5 mmol, 2.0 equiv.). Purification by flash column chromatography (5-10% EtOAc/hexanes) afforded the pure title compound as pale yellow oil (444 mg, 70%; *E*/*Z* (oxime) = 1.3:1). **^{1}H NMR** (700 MHz, $CDCl_3$): δ 7.56 (t, *J* = 5.7 Hz, 1.3H; major), 7.41 – 7.30 (m, 20.7H; major+minor), 7.28 – 7.24 (m, 2.3H; major+minor), 6.94 (t, *J* = 3.6 Hz, 1H; minor), 6.61 (dd, *J* = 18.9, 16.1 Hz, 2.3H; major+minor), 6.27 (dq, *J* = 15.9, 6.1 Hz, 2.3H; major+minor), 5.12 (d, *J* = 3.3 Hz, 4.6H; major+minor), 4.37 (d, *J* = 3.6 Hz, 2H; minor), 4.17 (ddd, *J* = 12.0, 6.1, 1.3 Hz, 4.6H; major+minor), 4.14 (d, *J* = 5.7 Hz, 2.6H; major); **^{13}C NMR** (176 MHz, $CDCl_3$): δ 150.9, 147.7, 137.7, 137.5, 136.7, 136.6, 133.4, 133.3, 128.72, 128.70, 128.57, 128.56, 128.4, 128.2, 128.10, 128.08, 127.99, 127.95, 126.68, 126.67, 125.3, 125.2, 76.4, 76.2, 71.9, 71.2, 66.8, 64.6; **IR** (cm^{-1}): 3028, 2851, 1495, 1453, 1365, 1107, 1013, 966, 914, 842, 733, 691; **HRMS**: *m*/*z* calculated for $C_{18}H_{19}NO_2Na^+$ $[M+Na]^+$: 304.1308; found: 304.1312.

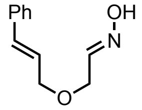

2-(Cinnamyloxy)acetaldehyde oxime (18b): Prepared according to GP-2 from **SM-a** (500 mg, 2.3 mmol, 1.0 equiv.) and hydroxylamine hydrochloride (313 mg, 4.5 mmol, 2.0 equiv.). Purification by flash column chromatography (10-30% EtOAc/hexanes) afforded the pure title compound as pale-yellow oil (287 mg, 67%; *E*/*Z* (oxime) = 1.1:1). **^{1}H NMR** (700 MHz, $CDCl_3$): δ 8.13 (b, 1H; minor), 7.85 (b, 1.1H; major), 7.54 (t, *J* = 5.6 Hz, 1.1H; major), 7.41 – 7.38 (m, 4.2H; major+minor), 7.34 – 7.30 (m, 4.2H; major+minor), 7.27 – 7.23 (m, 2.1H; major+minor), 6.96 (t, *J* = 3.7 Hz, 1H; minor), 6.63 (dd, *J* = 15.9, 9.8 Hz, 2.1H; major+minor), 6.28 (dq, *J* = 15.9, 6.2 Hz, 2.1H; major+minor), 4.40 (d, *J* = 3.7 Hz, 2H; minor), 4.19 (ddd, *J* = 9.7, 6.1, 1.3 Hz, 4.2H; major+minor), 4.15 (d, *J* = 5.6 Hz, 2.2H; major); **^{13}C NMR** (176 MHz MHz, $CDCl_3$): δ 151.5, 148.7, 136.61, 136.57, 133.5, 133.4, 128.73, 128.71, 128.02, 127.99, 126.69, 126.68, 125.24, 125.15, 72.0, 71.3, 66.7, 64.0; **IR** (cm^{-1}): 3203, 3027, 2868, 1448, 1395, 1348, 1284, 1116, 967, 920, 829, 731, 689; **HRMS**: *m*/*z* calculated for $C_{11}H_{13}NO_2H^+$ $[M+H]^+$: 192.1019; found: 192.1026.

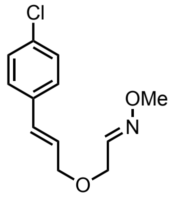

2-(((*E*)-3-(4-Chlorophenyl)allyl)oxy)acetaldehyde *O*-methyl oxime (S20): Prepared according to GP-2 from **A20** (438 mg, 1.7 mmol, 1.0 equiv.) and *O*-methylhydroxylamine hydrochloride (285 mg, 3.4 mmol, 2.0 equiv.). Purification by flash column chromatography (5-10% EtOAc/hexanes) afforded the pure title compound as clear oil (312 mg, 76%; *E*/*Z* (oxime) = 1.5:1). **^{1}H NMR** (500 MHz, $CDCl_3$): δ 7.46 (t, *J* = 5.7 Hz, 1.5H; major), 7.33 – 7.27 (m, 10H; major+minor), 6.86 (t, *J* = 3.9 Hz, 1H; minor), 6.58 (d, *J* = 15.9 Hz, 2.5H; major+minor), 6.25 (dt, *J* = 15.9, 6.0 Hz, 2.5H; major+minor), 4.31 (d, *J* = 3.7 Hz, 2H; minor), 4.16 (d, *J* = 6.0 Hz, 5H; major+minor), 4.12 (d, *J* = 5.7 Hz, 3H; major), 3.89 – 3.86 (m, 7.5H; major+minor); **^{13}C NMR** (126 MHz, $CDCl_3$): δ 150.0, 146.9, 135.13, 135.07, 133.6, 133.5, 131.82, 131.80, 128.9, 128.8, 127.82, 127.81, 126.1, 126.0, 71.6, 71.0, 66.9, 64.5, 62.2, 61.8; **IR** (cm^{-1}): 2938, 2899, 1491, 1464, 1359, 1089, 1040, 1012, 967, 846, 796; **HRMS**: *m*/*z* calculated for $C_{12}H_{14}ClNO_2Na^+$ [M+Na]$^+$: 262.0605; found: 262.0603.

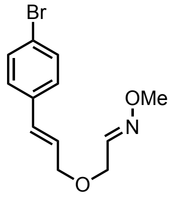

2-(((*E*)-3-(4-Bromophenyl)allyl)oxy)acetaldehyde *O*-methyl oxime (S21): Prepared according to GP-2 from **A21** (434 mg, 1.4 mmol, 1.0 equiv.) and *O*-methylhydroxylamine hydrochloride (241 mg, 2.9 mmol, 2.0 equiv.). Purification by flash column chromatography (5-10% EtOAc/hexanes) afforded the pure title compound as colorless foam (308 mg, 75%; *E*/*Z* (oxime) = 1.3:1). **^{1}H NMR** (700 MHz, $CDCl_3$): δ 7.46 (t, *J* = 5.7 Hz, 1.3H; major), 7.45 – 7.42 (m, 4.6H; major+minor), 7.27 – 7.23 (m, 4.6H; major+minor), 6.86 (t, *J* = 3.7 Hz, 1H; minor), 6.56 (dd, *J* = 15.9, 3.8 Hz, 2.3H; major+minor), 6.26 (dt, *J* = 15.9, 6.0 Hz, 2.3H; major+minor), 4.31 (d, *J* = 3.7 Hz, 2H; minor), 4.17 – 4.15 (m, 4.6H; major+minor), 4.12 (d, *J* = 5.7 Hz, 2.6H; major), 3.87 (d, *J* = 0.7 Hz, 6.9H; major+minor); **^{13}C NMR** (176 MHz, $CDCl_3$): δ 150.0, 146.9, 135.6, 135.5, 131.9, 131.83, 131.80, 131.79, 128.14, 128.13, 126.2, 126.1, 121.74, 121.70, 71.6, 71.0, 66.9, 64.5, 62.2, 61.8; **IR** (cm^{-1}): 2937, 2851, 1487, 1401, 1358, 1109, 1072, 1040, 1008, 966, 844, 793; **HRMS**: *m*/*z* calculated for $C_{12}H_{14}BrNO_2Na^+$ [M+Na]$^+$: 306.0100; found: 306.0107.

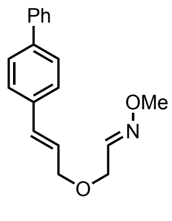

2-(((*E*)-3-([1,1'-Biphenyl]-4-yl)allyl)oxy)acetaldehyde *O*-methyl oxime (S22): Prepared according to GP-2 from **A22** (497 mg, 1.7 mmol, 1.0 equiv.) and *O*-methylhydroxylamine hydrochloride (278 mg, 3.3 mmol, 2.0 equiv.). Purification by flash column chromatography (5-10% EtOAc/hexanes) afforded the pure title compound as colorless solid (364 mg, 78%; *E*/*Z* (oxime) = 1.1:1). **^{1}H NMR** (700 MHz, $CDCl_3$): δ 7.60 (d, *J* = 7.4 Hz, 4.2H; major+minor), 7.57 (dd, *J* = 8.3, 2.8 Hz, 4.2H; major+minor), 7.50 – 7.42 (m, 9.5H; major+minor), 7.36 – 7.33 (m, 2.1H; major+minor), 6.88 (t, *J* = 3.7 Hz, 1H; minor), 6.69 – 6.64 (m, 2.1H; major+minor), 6.32 (dtd, *J* = 15.9, 6.1, 1.4 Hz, 2.1H; major+minor), 4.33 (d, *J* = 3.7 Hz, 2H; minor), 4.22 – 4.19 (m, 4.2H; major+minor), 4.14 (d, *J* = 5.7 Hz, 2.2H; major), 3.88 (d, *J* = 1.0 Hz, 6.3H; major+minor); **^{13}C NMR** (176 MHz, $CDCl_3$): δ 150.2, 147.0, 140.74 (2C), 140.72, 140.69, 135.7, 135.6, 132.84, 132.82, 128.9 (2C), 127.48, 127.46, 127.39, 127.37, 127.09, 127.08, 127.05 (2C), 125.4, 125.3, 71.9, 71.3, 66.8, 64.4, 62.2, 61.9; **IR** (cm^{-1}): 2940, 2848, 1486, 1448, 1349, 1264, 1104, 1042, 967, 851, 754, 689; **HRMS**: *m*/*z* calculated for $C_{18}H_{19}NO_2Na^+$ $[M+Na]^+$: 304.1308; found: 304.1301.

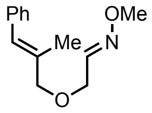

2-(((*E*)-2-Methyl-3-phenylallyl)oxy)acetaldehyde *O*-methyl oxime (S26): Prepared according to GP-2 from **A26** (499 mg, 2.1 mmol, 1.0 equiv.) and *O*-methylhydroxylamine hydrochloride (353 mg, 4.2 mmol, 2.0 equiv.). Purification by flash column chromatography (5-10% EtOAc/hexanes) afforded the pure title compound as pale-yellow oil (407 mg, 88%; *E*/*Z* (oxime) = 1.4:1). **^{1}H NMR** (500 MHz, $CDCl_3$): δ 7.48 (t, *J* = 5.7 Hz, 1.4H; major), 7.34 (td, *J* = 7.7, 2.0 Hz, 4.8H; major+minor), 7.28 (dd, *J* = 6.7, 3.8 Hz, 4.8H; major+minor), 7.23 (dt, *J* = 8.7, 3.8 Hz, 2.4H, major+minor), 6.88 (t, *J* = 3.6 Hz, 1H; minor), 6.52 (s, 2.4H; major+minor), 4.29 (d, *J* = 3.7 Hz, 2H; minor), 4.11 (d, *J* = 5.7 Hz, 2.8H; major), 4.06 (s, 4.8H; major+minor), 3.88 (d, *J* = 1.6 Hz, 7.2H; major+minor), 1.92 – 1.87 (m, 7.2H; major+minor); **^{13}C NMR** (176 MHz, $CDCl_3$): δ 150.2, 147.1, 137.42, 137.36, 134.5, 134.4, 129.00, 128.99, 128.22, 128.20, 127.8, 127.7, 126.70, 126.65, 77.4, 76.9, 66.6, 64.1, 62.1, 61.8, 15.6, 15.5; **IR** (cm^{-1}): 2938, 2900, 144, 1351, 1091, 1039, 918, 848, 744, 697; **HRMS**: *m*/*z* calculated for $C_{13}H_{17}NO_2H^+$ $[M+H]^+$: 220.1332; found: 220.1333.

General Procedure for Oxime Synthesis from Alcohols (GP-3)

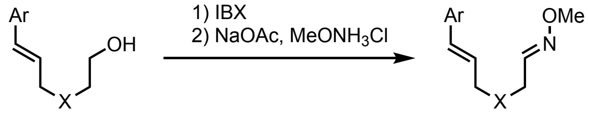

A 25-mL round-bottom flask equipped with a magnetic stir bar was charged with the corresponding alcohol (1.0 equiv.) and DMSO (0.3 M). IBX (1.5 equiv.) was added and the mixture stirred at rt until complete as judged by TLC analysis (4-18 h). Water was added to the reaction and the mixture filtered through a pad of celite. After washing the pad with Et_2O, the filtrate was collected, the organic layer separated and the aqueous layer extracted with Et_2O (3x). The combined organic layers were washed with water (2x) and brine (2x), dried over $MgSO_4$, filtered, concentrated *in vacuo* and dried using high-vac.

The crude aldehyde was dissolved in CH_2Cl_2 (0.1 M), before adding NaOAc (4.0 equiv.) and the respective hydroxylamine hydrochloride (2.0 equiv.). The mixture was stirred at rt until complete as judged by TLC analysis (4-18 h). $NaHCO_3$ (aq., sat.) was added, the organic layer separated and the aqueous layer extracted with EtOAc (3x). The combined organic layers were washed with brine, dried over Na_2SO_4, filtered and concentrated *in vacuo*. Purification by flash column chromatography (EtOAc/hexanes) afforded the corresponding pure oxime as mixture of *E*/*Z* oxime isomers.

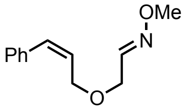

2-(((*Z*)-3-Phenylallyl)oxy)acetaldehyde *O*-methyl oxime ((*Z*)-15): Prepared according to GP-3 from **A15** (207 mg, 1.2 mmol, 1.0 equiv.) and *O*-methylhydroxylamine hydrochloride (194 mg, 2.3 mmol, 2.0 equiv.). Purification by flash column chromatography (20-30% EtOAc/hexanes) afforded the pure title compound as clear oil (84 mg, 35%; *E*/*Z* (oxime) =1.3:1). **^{1}H NMR** (700 MHz, $CDCl_3$): δ 7.43 (t, *J* = 5.7 Hz, 1.3H; major), 7.35 (t, *J* = 7.6 Hz, 4.6H; major+minor), 7.29 – 7.26 (m, 2.3H; major+minor), 7.20 (d, *J* = 7.4 Hz, 4.6H; major+minor), 6.84 (t, *J* = 3.7 Hz, 1H; minor), 6.64 (dd, *J* = 11.8, 4.9 Hz, 2.3H; major+minor), 5.86 – 5.81 (m, 2.3H; major+minor), 4.29 – 4.27 (m, 6.6H; major+minor), 4.09 (d, *J* = 5.7 Hz, 2.6H; major), 3.86 (s, 3H; minor), 3.84 (s, 3.9H; major); **^{13}C NMR** (176 MHz, $CDCl_3$): δ 150.2, 147.0, 136.59, 136.55, 132.5, 132.4, 128.89, 128.87, 128.41, 128.40, 128.3, 128.2, 127.5, 127.4, 68.0, 67.4, 67.1, 64.7, 62.2, 61.8; **IR** (cm^{-1}): 2938, 1494, 1447, 1340, 1098, 1041, 866, 851, 772, 698; **HRMS**: *m*/*z* calculated for $C_{12}H_{15}NO_2Na^+$ [M+Na]$^+$: 228.0995; found: 228.0995.

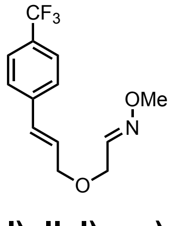

2-(((*E*)-3-(4-(Trifluoromethyl)phenyl)allyl)oxy)acetaldehyde *O*-methyl oxime (S23): Prepared according to GP-3 from **A23** (437 mg, 1.8 mmol, 1.0 equiv.) and *O*-methylhydroxylamine hydrochloride (296 mg, 3.6 mmol, 2.0 equiv.). Purification by flash column chromatography (5-10% EtOAc/hexanes) afforded the pure title compound as clear solid (343 mg, 71%; *E*/*Z* (oxime) = 1.4:1). **^{1}H NMR** (700 MHz, $CDCl_3$): δ 7.57 (dd, *J* = 8.2, 2.8 Hz, 4.8H; major+minor), 7.49 – 7.45 (m, 6.2H; major+minor), 6.87 (t, *J* = 3.7 Hz, 1H; minor), 6.66 (dd, *J* = 16.0, 4.1 Hz, 2.4H; major+minor), 6.36 (dt, *J* = 16.0, 5.8 Hz, 2.4H; major+minor), 4.32 (d, *J* = 3.7 Hz, 2H; minor), 4.20 (d, *J* = 5.8 Hz, 4.8H; major+minor), 4.14 (d, *J* = 5.7 Hz, 2.8H; major), 3.88 (s, 7.2H; major+minor); **^{13}C NMR** (176 MHz, $CDCl_3$): δ 149.9, 146.8, 140.2, 140.1, 131.3, 131.2, 129.7 (q, *J* = 32.4 Hz), 129.6 (q, *J* = 32.4 Hz), 128.3, 128.1, 126.73, 126.72, 125.7 – 125.5 (m, 2C), 124.3 (q, *J* = 271.9 Hz, 2C), 71.4, 70.8, 67.0, 64.6, 62.1, 61.8; **IR** (cm^{-1}): 2943, 1615, 1323, 1106, 1066, 1040, 1016, 967, 854, 821, 728, 651; **HRMS**: *m*/*z* calculated for $C_{13}H_{14}F_3NO_2H^+$ $[M+H]^+$: 274.1049; found: 274.1044.

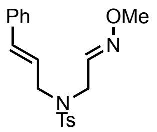

***N*-Cinnamyl-*N*-(2-(methoxyimino)ethyl)-4-methylbenzenesulfonamide (S25):** Prepared according to GP-3 from *N*-cinnamyl-*N*-(2-hydroxyethyl)-4-methylbenzenesulfonamide[14] (700 mg, 2.1 mmol, 1.0 equiv.) and *O*-methylhydroxylamine hydrochloride (353 mg, 4.2 mmol, 2.0 equiv.). Purification by flash column chromatography (10-30% EtOAc/hexanes) afforded the pure title compound as clear oil (371 mg, 49%; *E*/*Z* (oxime) = 1.2:1). **^{1}H NMR** (700 MHz, $CDCl_3$): δ 7.73 (d, *J* = 7.6 Hz, 4.4H; major+minor), 7.34 – 7.28 (m, 8.8H; major+minor), 7.27 (d, *J* = 7.4 Hz, 4.4H; major+minor), 7.26 – 7.23 (m, 2.2H; major+minor), 7.21 (t, *J* = 5.9 Hz, 1.2H; major), 6.65 (t, *J* = 4.3 Hz, 1H; minor), 6.45 (d, *J* = 15.7 Hz, 2.2H; major+minor), 6.01 – 5.93 (m, 2.2H; major+minor), 4.04 (d, *J* = 4.3 Hz, 2H; minor), 3.96 (dd, *J* = 9.6, 7.0 Hz, 4.4H; major+minor), 3.93 (d, *J* = 5.9 Hz, 2.4H; major), 3.79 (s, 3H; minor), 3.77 (s, 3.6H; major), 2.44 (s, 3H; minor), 2.43 (s, 3.6H; major); **^{13}C NMR** (176 MHz, $CDCl_3$): δ 147.9, 145.6, 143.9, 143.8, 137.0, 136.5, 136.3, 136.1, 135.0, 134.9, 130.1, 130.0, 128.74, 128.69, 128.2, 128.1, 127.43, 127.43, 126.7, 126.6, 123.3, 123.0, 62.2, 61.9, 51.5, 50.0, 45.9, 42.9, 21.7, 21.6; **IR** (cm^{-1}): 2937, 1597, 1495, 1448, 1337, 1156, 1089, 1030, 968, 897, 814, 731, 654; **HRMS**: *m*/*z* calculated for $C_{19}H_{22}N_2O_3SNa^+$ $[M+Na]^+$: 381.1243; found: 381.1245.

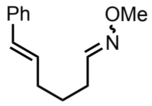

(5*E*)-6-Phenylhex-5-enal *O*-methyl oxime (S27): text. Prepared according to GP-3 from 6-phenylhex-5-en-1-ol[19] (763 mg, 4.3 mmol, 1.0 equiv.; *E*/*Z* = 2.6:1) and *O*-methylhydroxylamine hydrochloride (723 mg, 8.7 mmol, 2.0 equiv.) Purification by flash column chromatography (2-10% EtOAc/hexanes) afforded the pure title compound as clear oil (797 mg, 91%; *E*/*Z* (styrene) = 2.6:1; *E*/*Z* (oxime) = 1.5:1). Characterization data is provided for the two major (*E*)-styrene isomers: **^{1}H NMR** (700 MHz, $CDCl_3$): δ 7.39 (t, *J* = 6.2 Hz, 1.5H; major), 7.36 – 7.30 (m, 5H; major+minor), 7.31 – 7.27 (m, 5H; major+minor), 7.24 – 7.18 (m, 2.5H; major+minor), 6.66 (t, *J* = 5.5 Hz, 1H; minor), 6.40 (d, *J* = 15.8 Hz, 2.5H; major+minor), 6.19 (dtd, *J* = 15.6, 6.9, 1.5 Hz, 2.5H; major+minor), 3.87 (s, 3H; minor), 3.82 (s, 4.5H; major), 2.41 – 2.32 (m, 4H; minor), 2.29 – 2.19 (m, 6H; major), 1.71 – 1.60 (m, 5H; major+minor)17; **^{13}C NMR** (176 MHz, $CDCl_3$): δ 151.5, 151.4, 150.6, 150.5, 137.74, 137.73, 137.6 (2C), 131.9 (2C), 130.80, 130.79, 129.84 (2C), 129.81, 129.76, 128.8 (2C), 128.62, 128.62, 128.3 (2C), 127.1 (2C), 126.74, 126.73, 126.1 (2C), 61.71, 61.69, 61.4, 61.3, 32.7, 32.5, 29.2, 29.1, 28.4, 28.1, 27.1, 26.64, 26.56, 26.1, 25.4, 25.2; **IR** (cm^{-1}): 3024, 2936, 1493, 1448, 1047, 964, 917, 883, 847, 803, 741, 692; **HRMS**: *m*/*z* calculated for $C_{13}H_{17}NOH^+$ $[M+H]^+$: 204.1383; found: 204.1376.

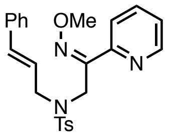

***N*-Cinnamyl-*N*-((*Z*)-2-(methoxyimino)-2-(pyridin-2-yl)ethyl)-4-methylbenzenesulfonamide (S32):** Prepared according to GP-3 from **A32** (225 mg, 0.55 mmol, 1.0 equiv.) and *O*-methylhydroxylamine hydrochloride (92 mg, 1.1 mmol, 2.0 equiv.). The oxime formation was carried out in methanol (5 mL) at reflux for 16 h. Purification by flash column chromatography (10-60% EtOAc/hexanes) afforded the pure title compound as clear oil (602 mg, 64%; 1:1.3 mixture of oxime isomers). **^{1}H NMR** (500 MHz, $CDCl_3$): δ 8.58 (d, *J* = 4.8 Hz, 1H; minor), 8.54 (d, *J* = 4.3 Hz, 1.3H; major), 7.74 – 7.60 (m, 8.2H; major+minor), 7.57 (d, *J* = 7.9 Hz, 1H; minor), 7.32 – 7.14 (m, 18.4H; major+minor), 6.43 (d, *J* = 15.8 Hz, 1H; minor), 6.32 (d, *J* = 15.9 Hz, 1.3H; major), 5.94 (ddt, *J* = 15.9, 11.2, 6.7 Hz, 2.3H; major+minor), 4.62 (s, 2.6H; major), 4.47 (s, 2H; minor), 3.99 – 3.96 (m, 8.5H; major+minor), 3.83 (s, 3H; minor), 2.41 (s, 6.9H; major+minor); **^{13}C NMR** (176 MHz, $CDCl_3$): δ 154.2, 152.9, 151.7, 149.7, 149.2, 148.8, 143.3, 143.1, 137.05, 137.03, 136.53, 136.46, 136.4, 135.7, 134.2, 133.7, 129.52, 129.51, 128.6, 128.5, 127.9, 127.8, 127.6, 127.5, 126.51, 126.45, 126.21, 124.2, 124.1, 123.9, 123.8, 122.0, 62.7, 62.5, 51.4, 50.4, 49.1, 40.4, 21.6, 21.5; **IR** (cm^{-1}): 2936, 2821, 1598, 1566, 1582, 1495, 1435, 1339, 1156, 1091,

1044, 994, 997, 906, 814, 729, 692, 667, 650; **HRMS**: *m/z* calculated for $C_{24}H_{25}N_3O_3SH^+$ [M+H]$^+$: 436.1689; found: 436.1687.

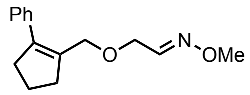

(2-((2-Phenylcyclopent-1-en-1-yl)methoxy)acetaldehyde *O*-methyl oxime (S34): Prepared according to GP-3 from **A34** (834 mg, 3.8 mmol, 1.0 equiv.) and *O*-methylhydroxylamine hydrochloride (638 mg, 7.6 mmol, 2.0 equiv.) Purification by flash column chromatography (0-10% EtOAc/hexanes) afforded the pure title compound as clear oil (602 mg, 64%; *E*/*Z* (oxime) = 1:1.6). **^{1}H NMR** (700 MHz, $CDCl_3$): δ 7.42 (t, *J* = 5.8 Hz, 1H; minor), 7.34 (t, *J* = 7.6 Hz, 5.2H; major+minor), 7.27 – 7.25 (m, 2.6H; major+minor), 7.24 – 7.21 (m, 5.2H; major+minor), 6.83 (t, *J* = 3.6 Hz, 1.6H; major), 4.22 (d, *J* = 3.6 Hz, 3.2H; major), 4.14 (d, *J* = 5.2 Hz, 5.2H; major+minor), 4.02 (d, *J* = 5.8 Hz, 2H; minor), 3.85 (s, 4.8H; major), 3.83 (s, 3H; minor), 2.81 – 2.77 (m, 5.2H; major+minor), 2.66 – 2.61 (m, 5.2H; major+minor), 2.00 – 1.94 (m, 5.2H; major+minor); **^{13}C NMR** (176 MHz, $CDCl_3$): δ 150.5, 147.2, 141.51, 141.49, 137.64, 137.60, 135.03, 134.96, 128.31, 128.29, 127.88, 127.86, 127.14, 127.09, 68.2, 67.6, 67.0, 64.6, 62.2, 61.8, 37.9 (2C), 35.9 (2C), 22.12, 22.10; **IR** (cm^{-1}): 2937, 2843, 1493, 1442, 1341, 1250, 1100, 1042, 850, 761, 698, 655; **HRMS**: *m/z* calculated for $C_{15}H_{19}NO_2Na^+$ [M+Na]$^+$: 268.1308; found: 268.1307.

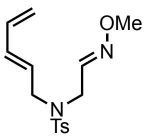

***N*-(2-(Methoxyimino)ethyl)-4-methyl-*N*-((*E*)-penta-2,4-dien-1-yl)benzenesulfonamide (42):** Prepared according to GP-3 from **A42** (500 mg, 1.8 mmol, 1.0 equiv.) and *O*-methylhydroxylamine hydrochloride (297 mg, 3.6 mmol, 2.0 equiv.). Purification by flash column chromatography (20% EtOAc/hexanes) afforded the pure title compound as clear oil (337 mg, 62%; *E*/*Z* (oxime) = 1.3:1; *E*/*Z* (diene) = 8:1). **^{1}H NMR** (700 MHz, $CDCl_3$): δ 7.69 (dd, *J* = 8.3, 2.2 Hz, 4.6H; major+minor), 7.31 (t, *J* = 8.4 Hz, 4.6H; major+minor), 7.17 (t, *J* = 5.9 Hz, 1.3H; major), 6.61 (t, *J* = 4.3 Hz, 1H; minor), 6.29 – 6.22 (m, 2.3H; major+minor), 6.10 (dd, *J* = 15.2, 10.7 Hz, 2.3H; major+minor), 5.54 – 5.43 (m, 2.3H; major+minor), 5.18 (d, *J* = 17.0 Hz, 2.3H; major+minor), 5.11 (dd, *J* = 10.0, 7.8 Hz, 2.3H; major+minor), 3.98 (d, *J* = 4.3 Hz, 2H; minor), 3.88 (d, *J* = 5.9 Hz, 2.6H; major), 3.84 – 3.81 (m, 7.6H; major+minor), 3.79 (s, 3.9H; major+minor), 2.43 (d, *J* = 2.2 Hz, 6.9H; major+minor); **^{13}C NMR** (176 MHz, $CDCl_3$): δ 147.9, 145.5, 143.9, 143.7, 136.8, 136.3, 135.8, 135.7, 135.64, 135.56, 130.0, 129.9, 127.38, 127.37, 127.2, 127.0, 118.6, 118.4, 62.2, 61.9, 51.0, 49.9, 45.9, 42.8, 21.64, 21.63; **IR** (cm^{-1}): 2938, 1598, 1441, 1337, 1156, 1090,

1030, 1005, 911, 854, 814, 743, 658; **HRMS**: *m*/*z* calculated for $C_{15}H_{20}N_2O_3SNa^+$ $[M+Na]^+$: 331.1087; found: 331.1088.

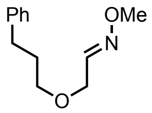

2-(3-Phenylpropoxy)acetaldehyde *O*-methyl oxime (45): Prepared according to GP-3 from **A45** (926 mg, 5.1 mmol, 1.0 equiv.) and *O*-methylhydroxylamine hydrochloride (858 mg, 10.3 mmol, 2.0 equiv.). Purification by flash column chromatography (2-10% EtOAc/hexanes) afforded the pure title compound as clear oil (338 mg, 32%; *E*/*Z* (oxime) = 1:1.1). **^{1}H NMR** (700 MHz, $CDCl_3$): δ 7.44 (t, *J* = 5.8 Hz, 1H; minor), 7.28 (td, *J* = 7.9, 2.2 Hz, 4.2H; major+minor), 7.21 – 7.17 (m, 6.3H; major+minor), 6.83 (t, *J* = 3.7 Hz, 1.1H; major), 4.24 (d, *J* = 3.7 Hz, 2.2H; major), 4.06 (d, *J* = 5.8 Hz, 2H; minor), 3.87 (s, 3.3H; major), 3.86 (s, 3H; minor), 3.46 (q, *J* = 6.2 Hz, 4.2H; major+minor), 2.70 (q, *J* = 7.7 Hz, 4.2H; major+minor), 1.95 – 1.88 (m, 4.2H; major+minor); **^{13}C NMR** (176 MHz, $CDCl_3$): δ 150.6, 147.3, 141.9, 141.8, 128.6 (2C), 128.49, 128.47, 125.99, 125.96, 70.6, 70.0, 67.5, 65.1, 62.2, 61.8, 32.34, 32.33, 31.3, 31.2; **IR** (cm^{-1}): 2938, 2861, 1496, 1454, 1114, 1040, 911, 850, 744, 698; **HRMS**: *m*/*z* calculated for $C_{12}H_{17}NO_2H^+$ $[M+H]^+$: 208.1332; found: 208.1328.

General Procedure for Oxime Synthesis from Carbonyl Compounds (GP-4)

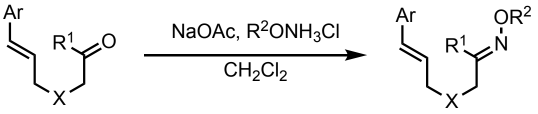

A 50-mL round-bottom flask equipped with a magnetic stir bar was charged with the corresponding aldehyde (1.0 equiv.) and CH_2Cl_2 (0.1 M). Next, NaOAc (4.0 equiv.) and the corresponding hydroxylamine hydrochloride (2.0 equiv.) were added sequentially and the mixture stirred at rt until complete as judged by TLC analysis (4-18 h). $NaHCO_3$ (aq., sat.) was added and the organic layer separated. Then, the aqueous layer was extracted with EtOAc (3x) and the combined organic layers washed with brine, dried over Na_2SO_4, filtered and concentrated in vacuo. Purification by flash column chromatography (EtOAc/hexanes) afforded the pure oxime as mixture of *E*/*Z* oxime isomers.

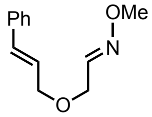

2-(Cinnamyloxy)acetaldehyde *O*-methyl oxime (*E*-15): Prepared according to GP-4 from 2-(cinnamyloxy)acetaldehyde[20] (1.33 g, 7.6 mmol, 1.0 equiv.) and *O*-methylhydroxylamine hydrochloride (1.26 g, 15.1 mmol, 2.0 equiv.). Purification by flash column chromatography (10-20% EtOAc/hexanes) afforded the pure title

compound as clear oil (1.05 g, 68%; *E*/*Z* (oxime) = 1.4:1). **^{1}H NMR** (700 MHz, $CDCl_3$): δ 7.47 (t, *J* = 5.7 Hz, 1.4H; major), 7.41 – 7.37 (m, 4.8H; major+minor), 7.34 – 7.30 (m, 4.8H; major+minor), 7.27 – 7.23 (m, 2.4H; major+minor), 6.87 (t, *J* = 3.6 Hz, 1H; minor), 6.62 (d, *J* = 15.9 Hz, 2.4H; major+minor), 6.27 (dtd, *J* = 15.9, 6.1, 1.0 Hz, 2.4H; major+minor), 4.31 (d, *J* = 3.7 Hz, 2H; minor), 4.20 – 4.16 (m, 4.8H; major+minor), 4.13 (d, *J* = 5.7 Hz, 2.8H; major), 3.87 (s, 7.2H; major+minor); **^{13}C NMR** (176 MHz, $CDCl_3$): δ 150.2, 147.0, 136.7, 136.6, 133.37, 133.35, 128.73, 128.71, 128.01, 127.97, 126.69, 126.68, 125.4, 125.3, 71.9, 71.3, 66.8, 64.4, 62.2, 61.9; **IR** (cm^{-1}): 2938, 2850, 1449, 1358, 1108, 1040, 965, 851, 735, 691; **HRMS**: *m*/*z* calculated for $C_{12}H_{15}NO_2Na^+$ $[M+Na]^+$: 228.0995; found: 228.0999.

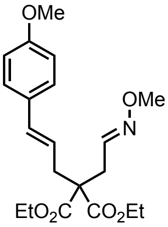

Diethyl 2-(2-(methoxyimino)ethyl)-2-((*E*)-3-(4-methoxyphenyl)allyl)malonate (S24): Prepared according to GP-4 from **A24** (190 mg, 0.6 mmol, 1.0 equiv.) and *O*-methylhydroxylamine hydrochloride (91 mg, 1.1 mmol, 2.0 equiv.). Purification by flash column chromatography (2-20% EtOAc/hexanes) afforded the pure title compound as yellow oil (150 mg, 73%; *E*/*Z* (oxime) = 2:1). **^{1}H NMR** (500 MHz, CD_2Cl_2): δ 7.38 – 7.31 (m, 2H; major), 7.26 (dd, *J* = 8.8, 3.0 Hz, 6H), 6.85 – 6.81 (m, 6H), 6.70 (t, *J* = 5.4 Hz, 1H), 6.40 (dd, *J* = 15.7, 7.3 Hz, 3H), 5.91 (dt, *J* = 15.5, 7.6 Hz, 3H), 4.19 (q, *J* = 7.1 Hz, 12H), 3.83 (s, 3H), 3.78 (s, 15H), 2.87 (d, *J* = 5.4 Hz, 2H), 2.77 (dd, *J* = 7.5, 3.8 Hz, 6H), 2.73 (d, *J* = 6.4 Hz, 4H), 1.24 (t, *J* = 7.1 Hz, 18H); **^{13}C NMR** (126 MHz, CD_2Cl_2): δ 170.9, 170.7, 159.8 (2C), 147.1, 147.0, 134.44 (2C), 134.3 (2C), 130.3, 127.9 (2C), 121.62, 121.60, 114.4, 62.22, 62.15, 62.1, 61.9, 57.4, 56.5, 55.8 (2C), 38.0, 37.5, 33.4, 29.6, 14.5, 14.4; **IR** (cm^{-1}): 2980, 2936, 1729, 1608, 1512, 1301, 1250, 1205, 1032, 971, 841; **HRMS**: *m*/*z* calculated for $C_{20}H_{27}NO_6H^+$ $[M+H]^+$: 378.1911; found: 378.1910.

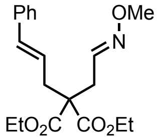

Diethyl 2-cinnamyl-2-(2-(methoxyimino)ethyl)malonate (S28): Prepared according to GP-4 from **A28** (350 mg, 1.1 mmol, 1.0 equiv.) and *O*-methylhydroxylamine hydrochloride (184 mg, 2.2 mmol, 2.0 equiv.). Purification by flash column chromatography (20% EtOAc/hexanes) afforded the pure title compound as pale-yellow oil (366 mg, 96%; *E*/*Z* (oxime) = 1.3:1). **^{1}H NMR** (500 MHz, $CDCl_3$): δ 7.37 (t, *J* = 6.4 Hz, 1.3H; major), 7.33 – 7.27 (m, 9.2H; major+minor), 7.22 (t, *J* = 6.5 Hz, 2.3H;

major+minor), 6.73 (t, *J* = 5.4 Hz, 1H; minor), 6.46 (dd, *J* = 15.7, 5.2 Hz, 2.3H; major+minor), 6.10 – 6.02 (m, 2.3H; major+minor), 4.22 (q, *J* = 7.1 Hz, 9.2H; major+minor), 3.86 (s, 3H; minor), 3.81 (s, 3.9H; major), 2.93 (d, *J* = 5.4 Hz, 2H; minor), 2.86 – 2.80 (m, 4.6H; major+minor), 2.78 (d, *J* = 6.4 Hz, 2.6H; major), 1.26 (t, *J* = 7.1 Hz, 13.8H; major+minor); **^{13}C NMR** (176 MHz, $CDCl_3$): δ 170.5, 170.3, 146.62, 146.57, 137.1 (2C), 134.74, 134.73, 128.6 (2C), 127.6 (2C), 126.4 (2C), 123.5, 123.4, 61.84, 61.83, 61.76, 61.6, 56.9, 56.1, 37.7, 37.3, 33.2, 29.4, 14.22, 14.21; **IR** (cm^{-1}): 2981, 2938, 1727, 1445, 1367, 1185, 1095, 1041, 968, 851, 741, 693; **HRMS**: *m*/*z* calculated for $C_{19}H_{25}NO_5Na^+$ $[M+Na]^+$: 370.1625; found: 370.1630.

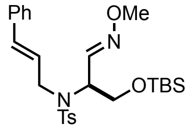

***N*-Cinnamyl-4-methyl-*N*-((*S*,*E*)-8,8,9,9-tetramethyl-2,7-dioxa-3-aza-8-siladec-3-en-5-yl)benzenesulfonamide (S29):** Prepared according to GP-4 from crude **A3-29** (343 mg, 0.72 mmol, 1.0 equiv.) and *O*-methylhydroxylamine hydrochloride (121 mg, 1.45 mmol, 2.0 equiv.). Purification by flash column chromatography (2-18% EtOAc/hexanes) afforded the pure title compound as clear oil (326 mg, 90%; *E*/*Z* (oxime) = 1.1:1). **^{1}H NMR** (500 MHz, $CDCl_3$): δ 7.73 (dd, *J* = 10.2, 8.3 Hz, 4.2H; major+minor), 7.36 – 7.18 (m, 15.8H; major+minor), 6.75 (d, *J* = 5.6 Hz, 1H; minor), 6.46 (dd, *J* = 20.2, 15.9 Hz, 2.1H; major+minor), 6.09 (ddt, *J* = 32.6, 15.9, 6.7 Hz, 2.1H; major+minor), 4.83 (q, *J* = 6.1 Hz, 1H; minor), 4.61 (q, *J* = 6.3 Hz, 1.1H; major), 4.17 – 3.91 (m, 6.3H; major+minor), 3.88 (ddd, *J* = 10.6, 6.7, 1.7 Hz, 2.1H; major+minor), 3.74 (s, 3.3H; major), 3.67 (s, 3H; minor), 2.40 (s, 3H; minor), 2.39 (s, 3.3H; major), 0.87 – 0.78 (m, 18.9H; major+minor), 0.00 (d, *J* = 3.2 Hz, 12.6H; major+minor); **^{13}C NMR** (176 MHz, $CDCl_3$): δ 147.5, 147.0, 143.32, 143.26, 138.1, 137.7, 136.5, 136.4, 133.5, 133.0, 129.6, 129.5, 128.7, 128.6, 128.0, 127.9, 127.68, 127.65, 126.59, 126.57, 126.3, 126.0, 63.4, 62.7, 62.0, 61.9, 58.6, 54.8, 49.3, 48.1, 25.9 (2C), 21.6 (2C), 18.34, 18.30, -5.35 (3C), -5.41; **IR** (cm^{-1}): 2928.4, 2855.9, 1462.9, 13339.7, 1253.1, 1156.5, 1090.2, 1043.9, 967.1, 900.1, 834.0, 813.1, 776.5, 727.4, 691.6, 657.8.; **HRMS**: *m*/*z* calculated $C_{26}H_{38}N_2O_4SSiH^+$ for $[M+H]^+$: 503.2394; found: 503.2393.

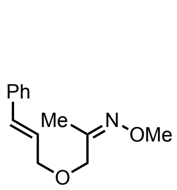

1-(Cinnamyloxy)propan-2-one *O*-methyl oxime (S30): Prepared according to GP-4 from **A30** (295 mg, 1.6 mmol, 1.0 equiv.) and *O*-methylhydroxylamine hydrochloride (259 mg, 3.1 mmol, 2.0 equiv.). Purification by flash column chromatography (5-15% EtOAc/hexanes) afforded the pure title compound as clear oil (316 mg, 93%; *E*/*Z* (oxime) = 1:3.3). **^{1}H NMR** (700 MHz, $CDCl_3$): δ 7.41 – 7.37 (m, 8.6H;

major+minor), 7.34 – 7.29 (m, 8.6H; major+minor), 7.24 (t, *J* = 7.3 Hz, 4.3H; major+minor), 6.62 (dd, *J* = 15.7, 3.7 Hz, 4.3H; major+minor), 6.31 – 6.25 (m, 4.3H; major+minor), 4.32 (s, 2H; minor), 4.14 (td, *J* = 6.5, 1.4 Hz, 8.6H; major+minor), 4.04 (s, 6.6H; major), 3.88 (s, 9.9H; major), 3.81 (s, 3H; minor), 1.97 (s, 3H; minor), 1.91 (s, 9.9H; major); **^{13}C NMR** (176 MHz, $CDCl_3$): δ 157.2, 154.9, 136.72, 136.67, 133.0, 132.9, 128.69, 128.67, 127.90, 127.86, 126.6 (2C), 125.59, 125.55, 71.9, 71.5, 70.9, 65.6, 61.7, 61.6, 16.7, 12.2; **IR** (cm^{-1}): 2937, 2852, 1495, 1448, 1366, 1115, 1045, 965, 888, 831, 735, 691; **HRMS**: *m*/*z* calculated for $C_{13}H_{17}NO_2Na^+$ $[M+Na]^+$: 242.1151; found: 242.1155.

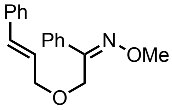

(*Z*)-2-(Cinnamyloxy)-1-phenylethan-1-one *O*-methyl oxime (S31): Prepared according to GP-4 from crude **A31** (stoichiometry based on 2.9 mmol **A31**) and *O*-methylhydroxylamine hydrochloride (482 mg, 5.8 mmol, 2.0 equiv.). Purification by flash column chromatography (5-15% EtOAc/hexanes) afforded the pure title compound as clear oil (255 mg, 31%; *Z* only). **^{1}H NMR** (700 MHz, $CDCl_3$): δ 7.71 (dd, *J* = 6.5, 2.9 Hz, 2H), 7.39 – 7.34 (m, 5H), 7.31 (t, *J* = 7.6 Hz, 2H), 7.24 (t, *J* = 7.3 Hz, 1H), 6.55 (d, *J* = 15.9 Hz, 1H), 6.22 (dt, *J* = 15.9, 6.1 Hz, 1H), 4.69 (s, 2H), 4.13 (d, *J* = 6.1 Hz, 2H), 4.00 (s, 3H); **^{13}C NMR** (176 MHz, $CDCl_3$): δ 155.5, 136.7, 134.4, 133.1, 129.3, 128.7, 128.4, 127.9, 127.2, 126.6, 125.6, 71.5, 62.4, 62.0; **IR** (cm^{-1}): 2936, 1724, 1494, 1445, 1327, 1184, 1115, 1041, 965, 885, 763, 743, 690; **HRMS**: *m*/*z* calculated for $C_{18}H_{19}NO_2H^+$ $[M+H]^+$: 282.1489; found: 282.1494.

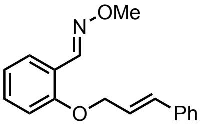

(*E*)-2-(Cinnamyloxy)benzaldehyde *O*-methyl oxime (S35): Prepared according to GP-4 from 2-(cinnamyloxy)benzaldehyde[21] (508 mg, 2.1 mmol, 1.0 equiv.) and *O*-methylhydroxylamine hydrochloride (356 mg, 4.3 mmol, 2.0 equiv.). Purification by flash column chromatography (10% EtOAc/hexanes) afforded the pure title compound as clear oil (459 mg, 81%; *E* only). **^{1}H NMR** (700 MHz, $CDCl_3$): δ 8.53 (s, 1H), 7.81 (dd, *J* = 7.7, 1.6 Hz, 1H), 7.41 (d, *J* = 7.6 Hz, 2H), 7.34 (t, *J* = 7.6 Hz, 3H), 7.29 – 7.26 (m, 1H), 6.96 (t, *J* = 7.5 Hz, 1H), 6.94 (d, *J* = 8.3 Hz, 1H), 6.72 (d, *J* = 16.0 Hz, 1H), 6.40 (dt, *J* = 16.0, 5.7 Hz, 1H), 4.73 (dd, *J* = 5.7, 1.4 Hz, 2H), 3.98 (s, 3H); **^{13}C NMR** (176 MHz, $CDCl_3$): δ 156.8, 145.0, 136.5, 133.2, 131.2, 128.8, 128.1, 126.7, 126.6, 124.2, 121.3, 121.2, 112.7, 69.3, 62.0; **IR** (cm^{-1}): 2934, 1598, 1486, 1449, 1340, 1241, 1108, 1051, 1005, 963, 917, 743, 690; **HRMS**: *m*/*z* calculated for $C_{17}H_{17}NO_2Na^+$ $[M+Na]^+$: 290.1151; found: 290.1147.

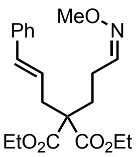

Diethyl 2-cinnamyl-2-(3-(methoxyimino)propyl)malonate (S36): Prepared according to GP-4 from **A36** (500 mg, 1.5 mmol, 1.0 equiv.) and *O*-methylhydroxylamine hydrochloride (251 mg, 3.0 , 2.0 equiv.). Purification by flash column chromatography (20% EtOAc/hexanes) afforded the pure title compound as pale-yellow oil (476 mg, 88%; *E*/*Z* (oxime) = 1.2:1). **^{1}H NMR** (500 MHz, $CDCl_3$): δ 7.35 – 7.27 (m, 10H; major+minor), 7.21 (t, *J* = 6.8 Hz, 2.2H; major+minor), 6.62 (t, *J* = 5.3 Hz, 1H; minor), 6.45 (d, *J* = 15.7 Hz, 2.2H; major+minor), 6.03 (dtd, *J* = 15.4, 7.5, 2.6 Hz, 2.2H; major+minor), 4.25 – 4.17 (m, 8.8H; major+minor), 3.84 (s, 3H; minor), 3.80 (s, 3.6H; major), 2.82 (d, *J* = 7.5 Hz, 4.4H; major+minor), 2.34 – 2.27 (m, 2H; minor), 2.24 – 2.17 (m, 2.4H; major), 2.14 – 2.06 (m, 4.4H; major+minor), 1.25 (t, *J* = 7.1 Hz, 13.2H; major+minor); **^{13}C NMR** (176 MHz, $CDCl_3$): δ 170.9 (2C), 150.2, 149.5, 137.12, 137.10, 134.2 (2C), 128.6 (2C), 127.6 (2C), 126.34, 126.33, 123.81, 123.79, 61.8, 61.6 (2C), 61.4, 57.5, 57.4, 36.8, 36.4, 29.7, 29.1, 24.8, 20.8, 14.2 (2C); **IR** (cm^{-1}): 2980, 1725, 1446, 1367, 1263, 1180, 1094, 1049, 1028, 967, 858, 741, 693; **HRMS**: *m*/*z* calculated for $C_{20}H_{27}NO_5Na^+$ $[M+Na]^+$: 384.1781; found: 384.1783.

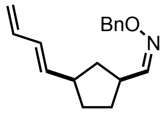

3-(Buta-1,3-dien-1-yl)cyclopentane-1-carbaldehyde *O*-benzyl oxime (40): Prepared according to GP-4 from (1*S**,3*R**)-3-(buta-1,3-dien-1-yl)cyclopentane-1-carbaldehyde[22] (172 mg, 1.2 mmol, 1.0 equiv.; *E*/*Z* (diene) = 4:1) and *O*-benzylhydroxylamine hydrochloride (366 mg, 2.3 mmol, 2.0 equiv.). Purification by flash column chromatography (0-10% EtOAc/hexanes) afforded the pure title compound as clear oil (205 mg, 70%; *E*/*Z* (oxime) = 2:1; *E*/*Z* (diene) = 10:1). Characterization data is provided for the two major (*E*)-diene isomers: **^{1}H NMR** (700 MHz, $CDCl_3$): δ 7.38 (d, *J* = 7.4 Hz, 2H; major), 7.37 – 7.33 (m, 12H; major+minor), 7.31 – 7.28 (m, 3H; major+minor), 6.65 (d, *J* = 6.9 Hz, 1H, minor), 6.29 (dt, *J* = 17.2, 10.3 Hz, 3H; major+minor), 6.05 (dd, *J* = 15.2, 10.6 Hz, 3H; major+minor), 5.69 – 5.63 (m, 3H; major+minor), 5.10 (d, *J* = 18.0 Hz, 5H; major+minor), 5.04 (s, 4H; major), 4.98 (d, *J* = 10.1 Hz, 3H; major+minor), 3.38 – 3.31 (m, 1H; minor), 2.80 – 2.71 (m, 2H; major), 2.66 – 2.52 (m, 3H; major+minor), 2.17 – 2.10 (m, 1H; minor), 2.06 – 2.00 (m, 2H; major), 1.99 – 1.83 (m, 6H; major+minor), 1.69 – 1.60 (m, 3H; major+minor), 1.49 – 1.41 (m, 3H; major+minor), 1.33 (dt, *J* = 12.6, 10.3 Hz, 2H; major), 1.21 (dt, *J* = 12.5, 10.3 Hz, 1H; minor); **^{13}C NMR** (176 MHz, $CDCl_3$): δ 156.9, 156.5, 154.9, 154.8, 138.7, 138.6, 137.24, 137.22, 129.89, 129.86, 128.53, 128.50, 128.44, 128.43, 127.98, 127.98, 115.41, 115.39, 75.8, 75.7, 43.34,

43.33, 39.90, 39.90, 38.28, 38.26, 32.3, 32.1, 30.0, 29.8; **IR** (cm^{-1}): 2950, 2866, 1496, 1453, 1366, 1040, 1003, 899, 732, 696; **HRMS**: *m*/*z* calculated for $C_{17}H_{21}NOH^+$ $[M+H]^+$: 256.1696; found: 256.1701.

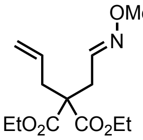

Diethyl 2-allyl-2-(2-(methoxyimino)ethyl)malonate (50): Prepared according to GP-4 from diethyl 2-allyl-2-(2-oxoethyl)malonate[12] (271 mg, 1.0 mmol, 1.0 equiv.) and *O*-methylhydroxylamine hydrochloride (167 mg, 2.0 mmol, 2.0 equiv.). Purification by flash column chromatography (10% EtOAc/hexanes) afforded the pure title compound as clear oil (233 mg, 86%; *E*/*Z* (oxime) = 1.3:1). Spectroscopic data were consistent with those reported in the literature.[23]

General Procedure for Hydrazone Synthesis (GP-5)

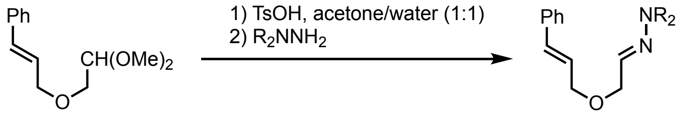

A 100-mL round-bottom flask equipped with a magnetic stir bar was charged with acetal (1.0 equiv.) and a 1:1 mixture (*v*/*v*) of water/acetone (0.1 M). *p*-TsOH (0.2 equiv.) was added and the reaction heated at 85 °C until complete as determined by TLC analysis (4-8 h). After cooling down to rt, $NaHCO_3$ (aq., sat.) was added and the mixture extracted with EtOAc (3x). The combined organic layers were dried over Na_2SO_4, filtered, concentrated *in vacuo* and dried using high-vac.

The crude aldehyde was dissolved in MeOH (0.1 M), the corresponding hydrazide/hydrazine (1.5 equiv.) added and the reaction stirred overnight at rt. Solvent was removed *in vacuo* and the crude product purified by flash column chromatography (EtOAc/hexanes) to afford the corresponding pure hydrazone.

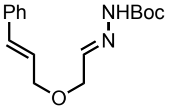

***Tert*-butyl 2-(2-(cinnamyloxy)ethylidene)hydrazine-1-carboxylate (18c):** Prepared according to GP-5 from **SM-a** (500 mg, 2.3 mmol, 1.0 equiv.) and *tert*-butyl carbazate (446 mg, 3.4 mmol, 1.5 equiv.). Purification by flash column chromatography (20-40% EtOAc/hexanes) afforded the pure title compound as pale-yellow solid (402 mg, 62%; *E*/*Z* (hydrazone) = 3.9:1). **^{1}H NMR** (700 MHz, $CDCl_3$): δ 7.86 (s, 3.9H; major), 7.41 – 7.36 (m, 9.8H; major+minor), 7.35 – 7.29 (m, 9.8H; major+minor), 7.24 (t, *J* = 7.3 Hz, 5.9H; major+minor), 6.65 (d, *J* = 7.8 Hz, 1H; minor), 6.61 (d, *J* = 15.8 Hz, 3.9H; major),

6.26 (dt, J = 15.9, 6.1 Hz, 4.9H; major+minor), 4.24 – 4.19 (m, 9.8H; major+minor), 4.19 – 4.15 (m, 9.8H; major+minor), 1.50 (s, 44.1H; major+minor); **^{13}C NMR** (176 MHz, $CDCl_3$): δ 152.8, 152.5, 143.1, 139.4, 136.6, 136.2, 134.2, 133.1, 128.7, 128.6, 128.2, 127.8, 126.7, 126.6, 125.4, 124.2, 81.4, 81.2, 71.8, 71.3, 69.3, 66.7, 28.3 (2C); **IR** (cm^{-1}): 3232, 2978, 2931, 1706, 1533, 1449, 1366, 1269, 1247, 1164, 1133, 1042, 1015, 966, 859, 723, 691; **HRMS**: *m*/*z* calculated for $C_{16}H_{22}N_2O_3Na^+$ $[M+Na]^+$: 313.1523; found: 313.1530.

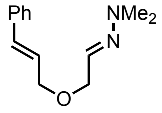

(*E*)-2-(2-(Cinnamyloxy)ethylidene)-1,1-dimethylhydrazine (18d): Prepared according to GP-5 from **SM-a** (300 mg, 1.4 mmol, 1.0 equiv.) and *N*,*N*-dimethylhydrazine (0.15 mL, 2.0 mmol, 1.5 equiv.). Purification by flash column chromatography (5-10% EtOAc/hexanes) afforded the pure title compound as pale-yellow oil (116 mg, 39%; *E* only). **^{1}H NMR** (700 MHz, $CDCl_3$): δ 7.38 (d, J = 8.0 Hz, 2H), 7.31 (t, J = 7.2 Hz, 2H), 7.23 (t, J = 7.3 Hz, 1H), 6.65 – 6.59 (m, 2H), 6.31 (dt, J = 15.9, 6.1 Hz, 1H), 4.18 (d, J = 6.1 Hz, 2H), 4.15 (d, J = 5.3 Hz, 2H), 2.83 (s, 6H); **^{13}C NMR** (176 MHz, $CDCl_3$): δ 136.9, 132.9, 131.8, 128.7, 127.8, 126.6, 126.1, 71.0, 70.7, 42.8; **IR** (cm^{-1}): 2854, 1598, 1496, 1447, 1262, 1116, 1036, 968, 817, 745, 693; **HRMS**: *m*/*z* calculated for $C_{13}H_{18}N_2OH^+$ $[M+H]^+$: 219.1492; found: 219.1498.

Miscellaneous Procedures

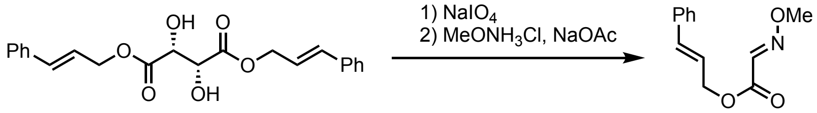

Cinnamyl 2-(methoxyimino)acetate (S33): A 25-mL round-bottom flask equipped with a magnetic stir bar was charged with dicinnamyl (2*R*,3*R*)-2,3-dihydroxysuccinate[24] (1.00 g, 2.6 mmol, 1.0 equiv.) and a 2:1 THF/water mixture. $NaIO_4$ (1.12 g, 5.2 mmol, 2.0 equiv.) was added and the reaction stirred overnight at rt. Then, solids were removed by filtration through a pad of celite and $NaHSO_3$ (aq., sat.) was added to the filtrate. The resulting mixture was extracted with Et_2O (3x) and the combined organic layers washed with brine, dried over $MgSO_4$, filtered and concentrated *in vacuo* and dried using high-vac. The crude oxoacetate was used for the next step without further purification.

To a 50-mL round-bottom flask equipped with magnetic stir bar containing crude oxoacetate and CH_2Cl_2 (25 mL) was sequentially added NaOAc (1.72 g, 20.9 mmol, 8.0 equiv.) and *O*-methylhydroxylamine hydrochloride (874 mg, 10.5 mmol, 4.0 equiv.). After stirring the resulting mixture overnight at rt, $NaHCO_3$ (aq., sat.) was added, the organic layer separated and the aqueous layer extracted with EtOAc (3x).

The combined organic layers were washed with brine, dried over Na_2SO_4, filtered and concentrated *in vacuo*. Purification by flash column chromatography (5-10% EtOAc/hexanes) afforded the pure title compound as clear oil (453 mg, 40%; *E*/*Z* (oxime) = 6.7:1). **^{1}H NMR** (500 MHz, $CDCl_3$): δ 7.52 (s, 6.7H; major), 7.40 (d, *J* = 7.3 Hz, 15.4H; major+minor), 7.33 (t, *J* = 7.4 Hz, 15.4H; major+minor), 7.29 – 7.25 (m, 7.7H; major+minor), 6.99 (s, 1H; minor), 6.71 (d, *J* = 15.9 Hz, 7.7H; major+minor), 6.39 – 6.26 (m, 7.7H; major+minor), 4.92 (dd, *J* = 6.6, 1.3 Hz, 13.4H; major), 4.86 (dd, *J* = 6.6, 1.3 Hz, 2H; minor), 4.07 (s, 20.1H; major), 4.05 (s, 3H; minor); **^{13}C NMR** (176 MHz, $CDCl_3$): δ 161.9, 158.8, 140.7, 136.9, 136.1 (2C), 135.5, 135.3, 128.77, 128.75, 128.40, 128.39, 126.84, 126.82, 122.3, 122.2, 66.3, 65.9, 64.0, 63.7; **IR** (cm^{-1}): 2941, 1719, 1598, 1494, 1449, 1382, 1318, 1265, 1198, 1171, 1047, 962, 917, 735, 691; **HRMS**: *m*/*z* calculated for $C_{12}H_{13}NO_3Na^+$ $[M+Na]^+$: 242.0788; found: 242.0794.

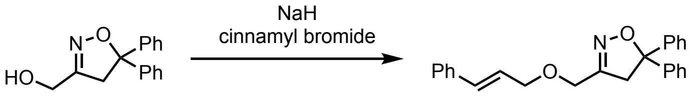

3-((Cinnamyloxy)methyl)-5,5-diphenyl-4,5-dihydroisoxazole (38): A 25-mL round-bottom flask equipped with a magnetic stir bar was charged with NaH (60% dispersion in mineral oil; 71 mg, 1.8 mmol, 1.5 equiv.) and THF (10 mL). Next, a solution of (5,5-diphenyl-4,5-dihydroisoxazol-3-yl)methanol[25] (300 mg, 1.2 mmol, 1.0 equiv.) in THF (2 mL) was added dropwise at 0 °C and then, after stirring for 15 min, a solution of cinnamyl bromide (257 mg, 1.3 mmol, 1.1 equiv.) in THF (2 mL) and the reaction allowed to warm up to rt and stirred overnight. NH_4Cl (aq., sat.) was added and the mixture extracted with EtOAc (3x). The combined organic layers were washed with brine, dried over Na_2SO_4, filtered and concentrated *in vacuo*. Purification by flash column chromatography (2-10% EtOAc/hexanes) afforded the pure title compound as pale-yellow oil (384 mg, 88%). **^{1}H NMR** (700 MHz, $CDCl_3$): δ 7.44 – 7.41 (m, 4H), 7.36 – 7.30 (m, 8H), 7.29 – 7.26 (m, 2H), 7.27 – 7.23 (m, 1H), 6.50 (d, *J* = 15.9 Hz, 1H), 6.18 (dt, *J* = 15.9, 6.2 Hz, 1H), 4.32 (s, 2H), 4.03 (dd, *J* = 6.2, 1.4 Hz, 2H), 3.70 (s, 2H); **^{13}C NMR** (176 MHz, $CDCl_3$): δ 156.7, 144.0, 136.5, 133.6, 128.7, 128.5, 128.00, 127.8, 126.7, 126.2, 124.9, 91.8, 71.0, 64.7, 48.4; **IR** (cm^{-1}): 3025, 2852, 1598, 1492, 1447, 1363, 1328, 1221, 1109, 1058, 966, 894, 865, 747, 692; **HRMS**: *m*/*z* calculated for $C_{25}H_{23}NO_2Na^+$ $[M+Na]^+$: 392.1621; found: 392.1619.

[2+2] Cycloaddition

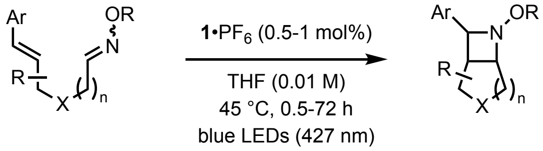

General Procedure (GP-6a) An oven-dried test tube (25x150 mm) equipped with a magnetic stir bar was charged with substrate (0.25 mmol, 1.0 equiv.),

[Ir(dF(CF$_3$)ppy)$_2$(dtbbpy)](PF$_6$) (**17**•PF$_6$) (1.4 mg or 2.8 mg, 0.5 or 1 mol%) and THF (25 mL). When a substrate required extended reaction time (>2 h), the reaction mixture was degassed by sparging with nitrogen gas for 30 min prior to irradiation and the reaction conducted under a nitrogen atmosphere. The test tube was sealed with a rubber septum and placed in front of a 40 W PR160-427 nm Kessil light at a distance of approximately 5 cm. The light was set to 100% intensity and the reaction stirred until complete as judged by TLC analysis (0.5-72 h). The internal temperature of the photoreactor was maintained below 45 °C by a fan. Upon completion, the reaction mixture was transferred to a 100-mL round-bottom flask and the solvent removed *in vacuo*. The diastereomeric ratio was determined by ^{1}H NMR analysis from the crude mixture, before purifying the crude product by flash column chromatography (EtOAc/hexanes) to afford the corresponding pure azetidine.
Note: The reaction can be alternatively run utilizing a 23W CFL lamp, although extended reaction times are necessary (~24 h for (*E*)-**15**).

General Procedure for Gram-Scale Reaction (GP-6b): A 500-mL round-bottom flask equipped with a magnetic stir bar was charged with substrate (1.0 equiv.), [Ir(dF(CF$_3$)ppy)$_2$(dtbbpy)](PF$_6$) (**17**•PF$_6$) (0.5 mol%) and THF (0.025 M). The flask was placed in between a 40 W PR160-427 nm Kessil light (100% intensity) and a 34 W H150-BLUE Kessil light at a distance of approximately 5 cm and the reaction stirred under ambient atmosphere until complete as judged by TLC analysis. Solvent was removed *in vacuo* and the diastereomeric ratio determined by ^{1}H NMR analysis from the crude mixture. Purification of the crude product by flash column chromatography (EtOAc/hexanes) afforded the corresponding pure azetidine.

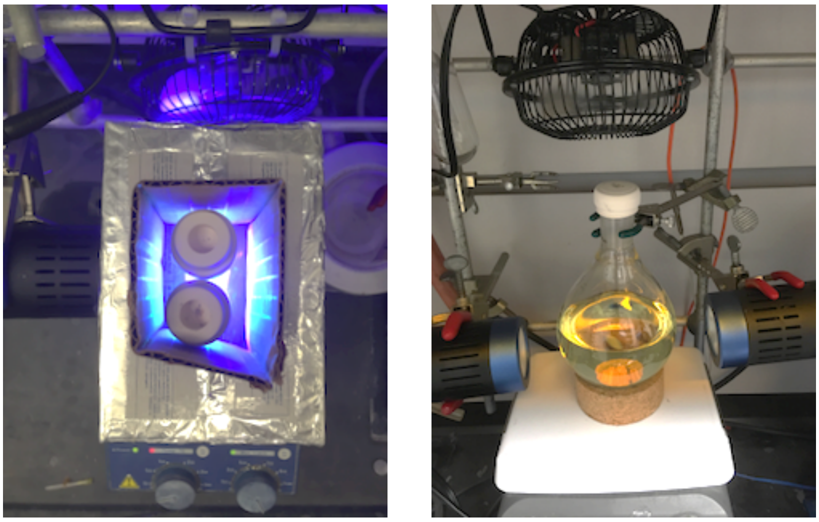

Supplementary Figure 5. Reaction setup for [2+2] cycloadditions; left: setup for 0.25 mmol scale reaction; right: setup for gram-scale reaction.

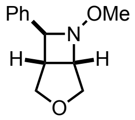

(1*R,5*S**,7*S**)-6-Methoxy-7-phenyl-3-oxa-6-azabicyclo[3.2.0]heptane (16):** Prepared according to GP-6a using (*E*)-**15** (51 mg, 0.25 mmol, 1.0 equiv.), **17**•PF_6 (1.4 mg, 0.5 mol%) and THF (25 mL) with a reaction time of 0.5 h. The diastereomeric ratio was determined to be >20:1 by ^{1}H NMR analysis of the crude mixture. Purification by flash column chromatography (10-20% EtOAc/hexanes) afforded the pure title compound as clear oil (49 mg, 96%; combined yield). **^{1}H NMR** (500 MHz, $CDCl_3$): δ 7.43 (d, *J* = 7.5 Hz, 2H), 7.37 (t, *J* = 7.6 Hz, 2H), 7.29 (t, *J* = 7.2 Hz, 1H), 4.76 (d, *J* = 10.6 Hz, 1H), 4.54 (t, *J* = 5.5 Hz, 1H), 4.42 (d, *J* = 5.8 Hz, 1H), 3.98 (d, *J* = 9.4 Hz, 1H), 3.58 (dd, *J* = 10.6, 5.3 Hz, 1H), 3.52 (dd, *J* = 9.4, 3.6 Hz, 1H), 3.45 (s, 3H), 2.72 (td, *J* = 5.8, 3.9 Hz, 1H); **^{13}C NMR** (126 MHz, $CDCl_3$): δ 141.9, 128.6, 127.6, 126.4, 73.9, 70.8, 67.5, 67.2, 60.6, 42.1; **IR** (cm^{-1}): 2949, 2852, 1466, 1162, 1084, 1059, 1019, 909, 730, 697; **HRMS**: *m*/*z* calculated for $C_{12}H_{15}NO_2H^+$ $[M+H]^+$: 206.1176; found: 206.1175.

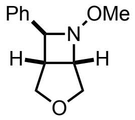

(1*R,5*S**,7*S**)-6-Methoxy-7-phenyl-3-oxa-6-azabicyclo[3.2.0]heptane (16):** Prepared according to GP-6a using (*Z*)-**15** (51 mg, 0.25 mmol, 1.0 equiv.), **17**•PF_6 (1.4 mg, 0.5 mol%) and THF (25 mL) with a reaction time of 0.5 h. The diastereomeric ratio was determined to be >20:1 by ^{1}H NMR analysis of the crude mixture. Purification by flash column chromatography (10-20% EtOAc/hexanes) afforded the pure title compound as clear oil (50 mg, 97%; combined yield). Spectroscopic data was found consistent with those obtained when (*E*)-**13** was used.

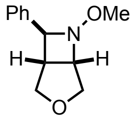

(1*R,5*S**,7*S**)-6-Methoxy-7-phenyl-3-oxa-6-azabicyclo[3.2.0]heptane (16):** Prepared according to GP-6b using (*E*)-**15** (1.40 g, 6.8 mmol, 1.0 equiv.), **17**•PF_6 (38 mg, 0.5 mol%) and THF (300 mL) with a reaction time of 1 h. The diastereomeric ratio was determined to be >20:1 by ^{1}H NMR analysis of the crude mixture. Purification by flash column chromatography (5-20% EtOAc/hexanes) afforded the pure title compound as clear oil (1.27 g, 91%; combined yield). Spectroscopic data was found consistent with those obtained when the reaction was conducted on 0.25 mmol scale.

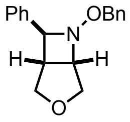

(1*R,5*S**,7*S**)-6-(Benzyloxy)-7-phenyl-3-oxa-6-azabicyclo[3.2.0]heptane (19a):** Prepared according to GP-6a using **18a** (70 mg, 0.25 mmol, 1.0 equiv.), **17•**PF_6 (1.4 mg, 0.5 mol%) and THF (25 mL) with a reaction time of 0.5 h. The diastereomeric ratio was determined to be 16:1 by ^{1}H NMR analysis of the crude mixture. Purification by flash column chromatography (5-10% EtOAc/hexanes) afforded the pure title compound as clear oil (67 mg, 96%; combined yield). **^{1}H NMR** (700 MHz, $CDCl_3$): δ 7.37 – 7.35 (m, 2H), 7.33 (t, *J* = 7.6 Hz, 2H), 7.29 – 7.23 (m, 6H), 4.76 (d, *J* = 10.5 Hz, 1H), 4.62 (s, 2H), 4.48 (d, *J* = 5.8 Hz, 1H), 4.30 (t, *J* = 5.6 Hz, 1H), 3.95 (d, *J* = 9.4 Hz, 1H), 3.50 – 3.45 (m, 2H), 2.65 (td, *J* = 5.9, 3.6 Hz, 1H) **^{13}C NMR** (176 MHz, $CDCl_3$): δ 141.9, 138.4, 128.6, 128.5, 128.3, 127.7, 127.5, 126.5, 75.6, 74.1, 70.8, 68.5, 67.6, 42.6; **IR** (cm^{-1}): 2850, 1495, 1453, 1366, 1204, 1084, 1061, 977, 909, 742, 695; **HRMS**: *m*/*z* calculated for $C_{18}H_{19}NO_2H^+$ $[M+H]^+$: 282.1489; found: 282.1491.

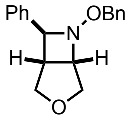

(1*R,5*S**,7*S**)-6-(Benzyloxy)-7-phenyl-3-oxa-6-azabicyclo[3.2.0]heptane (19a):** Prepared according to GP-6b using **18a** (1.20 g, 4.3 mmol, 1.0 equiv.), **17•**PF_6 (24 mg, 0.5 mol%) and THF (300 mL) with a reaction time of 1 h. The diastereomeric ratio was determined to be 20:1 by ^{1}H NMR analysis of the crude mixture. Purification by flash column chromatography (5-20% EtOAc/hexanes) afforded the pure title compound as clear oil (1.08 g, 90%; combined yield). Spectroscopic data was found consistent with those obtained when the reaction was conducted on 0.25 mmol scale.

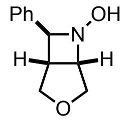

(1*R,5*S**,7*S**)-7-Phenyl-3-oxa-6-azabicyclo[3.2.0]heptan-6-ol (19b):** Prepared according to GP-6a using **18b** (48 mg, 0.25 mmol, 1.0 equiv.), **17•**PF_6 (1.4 mg, 0.5 mol%) and THF (25 mL) with a reaction time of 0.5 h. The diastereomeric ratio was determined to be >20:1 by ^{1}H NMR analysis of the crude mixture. Purification by flash column chromatography (20-40% EtOAc/hexanes) afforded the pure title compound as off-white solid (48 mg, 54%; combined yield). **^{1}H NMR** (700 MHz, $CDCl_3$): δ 7.35 – 7.30 (m, 4H), 7.29 – 7.26 (m, 1H), 4.79 (d, *J* = 10.7 Hz, 1H), 4.36 (d, *J* = 5.9 Hz, 1H), 4.32 (t, *J* = 5.6 Hz, 1H), 3.92 (d, *J* = 9.4 Hz, 1H), 3.51 – 3.45 (m, 2H), 2.74 (td, *J* = 5.9, 3.6 Hz, 1H); **^{13}C NMR** (176 MHz, $CDCl_3$): δ 140.8, 128.6, 127.9, 126.9, 75.5, 70.7, 69.1, 66.8, 41.8; **IR** (cm^{-1}): 3243, 2853, 1454, 1353, 1266, 1162, 1084, 1055, 954, 815,

741, 697; **HRMS**: *m*/*z* calculated for $C_{11}H_{13}NO_2Na^+$ $[M+Na]^+$: 214.0838; found: 214.0838.

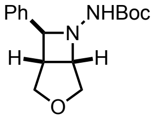

***Tert*-butyl ((1*R**,5*S**,7*S**)-7-phenyl-3-oxa-6-azabicyclo[3.2.0]heptan-6-yl)carbamate (19c):** Prepared according to GP-6a using **18c** (73 mg, 0.25 mmol, 1.0 equiv.), **17•**PF_6 (1.4 mg, 0.5 mol%) and THF (25 mL) with a reaction time of 0.5 h. The diastereomeric ratio was determined to be 13:1 by ^{1}H NMR analysis of the crude mixture. Purification by flash column chromatography (30-40% EtOAc/hexanes) afforded the pure title compound as pale-yellow solid (45 mg, 62%; combined yield). **^{1}H NMR** (700 MHz, $CDCl_3$): δ 7.50 (d, *J* = 7.4 Hz, 2H), 7.36 (t, *J* = 7.7 Hz, 2H), 7.26 (t, *J* = 7.4 Hz, 1H), 5.91 (b, 1H), 4.60 (t, *J* = 4.8 Hz, 1H), 4.49 (d, *J* = 11.5 Hz, 1H), 4.22 (d, *J* = 4.8 Hz, 1H), 4.03 (d, *J* = 9.5 Hz, 1H), 3.50 (dd, *J* = 9.5, 3.9 Hz, 1H), 3.47 (dd, *J* = 11.5, 4.1 Hz, 1H), 2.75 (q, *J* = 4.9 Hz, 1H), 1.41 (s, 9H); **^{13}C NMR** (176 MHz, $CDCl_3$): δ 154.9, 141.6, 128.6, 127.5, 126.0, 80.3, 77.2, 71.9, 68.7, 67.5, 43.2, 28.4; **IR** (cm^{-1}): 3262, 2975, 2857, 1734, 1695, 1522, 1455, 1366, 1247, 1161, 1049, 993, 906, 743, 670; **HRMS**: *m*/*z* calculated for $C_{16}H_{22}N_2O_3Na^+$ $[M+Na]^+$: 313.1523; found: 313.1528.

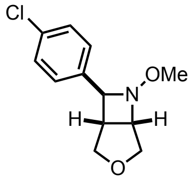

(1*R,5*S**,7*S**)-7-(4-Chlorophenyl)-6-methoxy-3-oxa-6-azabicyclo[3.2.0]heptane (20):** Prepared according to GP-6a using **S20** (62 mg, 0.26 mmol, 1.0 equiv.), **17•**PF_6 (1.4 mg, 0.5 mol%) and THF (25 mL) with a reaction time of 0.5 h. The diastereomeric ratio was determined to be >20:1 by ^{1}H NMR analysis of the crude mixture. Purification by flash column chromatography (2-20% EtOAc/hexanes) afforded the pure title compound as clear oil (61 mg, 98%; combined yield). **^{1}H NMR** (500 MHz, $CDCl_3$): δ 7.35 (d, *J* = 8.6 Hz, 2H), 7.32 (d, *J* = 8.6 Hz, 2H), 4.73 (d, *J* = 10.7 Hz, 1H), 4.52 (t, *J* = 5.6 Hz, 1H), 4.36 (d, *J* = 5.8 Hz, 1H), 3.94 (d, *J* = 9.5 Hz, 1H), 3.55 (dd, *J* = 10.7, 5.3 Hz, 1H), 3.49 (dd, *J* = 9.5, 3.6 Hz, 1H), 3.42 (s, 3H), 2.66 (td, *J* = 5.9, 3.5 Hz, 1H); **^{13}C NMR** (100 MHz, $CDCl_3$): δ 140.4, 133.3, 128.7, 127.8, 73.1, 70.7, 67.5, 67.1, 60.6, 42.2; **IR** (cm^{-1}): 2951, 2895, 2852, 1491, 1466, 1081, 1064, 1050, 1023, 1014, 979, 913, 820, 803, 725; **HRMS**: *m*/*z* calculated for $C_{12}H_{14}ClNO_2H^+$ $[M+H]^+$: 240.0786; found: 240.0786.

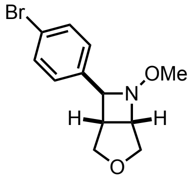

(1*R,5*S**,7*S**)-7-(4-Bromophenyl)-6-methoxy-3-oxa-6-azabicyclo[3.2.0]heptane (21):** Prepared according to GP-6a using **S21** (71 mg, 0.25 mmol, 1.0 equiv.), **17•**PF_6 (1.4 mg, 0.5 mol%) and THF (25 mL) with a reaction time of 0.5 h. The diastereomeric ratio was determined to be >20:1 by ^{1}H NMR analysis of the crude mixture. Purification by flash column chromatography (10-20% EtOAc/hexanes) afforded the pure title compound as white solid (60 mg, 85%; combined yield). **^{1}H NMR** (500 MHz, $CDCl_3$): δ 7.48 (d, *J* = 8.3 Hz, 2H), 7.29 (d, *J* = 8.3 Hz, 2H), 4.73 (d, *J* = 10.7 Hz, 1H), 4.51 (t, *J* = 5.5 Hz, 1H), 4.34 (d, *J* = 5.8 Hz, 1H), 3.94 (d, *J* = 9.5 Hz, 1H), 3.55 (dd, *J* = 10.7, 5.3 Hz, 1H), 3.49 (dd, *J* = 9.5, 3.6 Hz, 1H), 3.41 (s, 3H), 2.65 (td, *J* = 5.8, 3.7 Hz, 1H); **^{13}C NMR** (126 MHz, $CDCl_3$): δ 141.0, 131.7, 128.2, 121.4, 73.2, 70.7, 67.5, 67.1, 60.7, 42.2; **IR** (cm^{-1}): 2929, 2843, 1485, 1161, 1059, 1007, 937, 903, 858, 814, 792, 718; **HRMS**: *m*/*z* calculated for $C_{12}H_{14}BrNO_2H^+$ $[M+H]^+$: 284.0281; found: 284.0279.

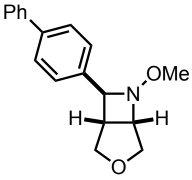

(1*R,5*S**,7*S**)-7-([1,1'-Biphenyl]-4-yl)-6-methoxy-3-oxa-6-azabicyclo[3.2.0]heptane (22):** Prepared according to GP-6a using **S22** (70 mg, 0.25 mmol, 1.0 equiv.), **17•**PF_6 (1.4 mg, 0.5 mol%) and THF (25 mL) with a reaction time of 0.5 h. The diastereomeric ratio was determined to be 16:1 by ^{1}H NMR analysis of the crude mixture. Purification by flash column chromatography (10-20% EtOAc/hexanes) afforded the pure title compound as clear oil (70 mg, 99%; combined yield). **^{1}H NMR** (500 MHz, $CDCl_3$): δ 7.60 (d, *J* = 8.1 Hz, 4H), 7.49 (d, *J* = 8.1 Hz, 2H), 7.44 (t, *J* = 7.6 Hz, 2H), 7.35 (t, *J* = 7.4 Hz, 1H), 4.77 (d, *J* = 10.6 Hz, 1H), 4.56 (t, *J* = 5.5 Hz, 1H), 4.45 (d, *J* = 5.8 Hz, 1H), 3.99 (d, *J* = 9.4 Hz, 1H), 3.58 (dd, *J* = 10.6, 5.3 Hz, 1H), 3.53 (dd, *J* = 9.3, 3.6 Hz, 1H), 3.46 (s, 3H), 2.75 (td, *J* = 5.8, 3.8 Hz, 1H); **^{13}C NMR** (126 MHz, $CDCl_3$): δ 141.1, 141.0, 140.6, 128.9, 127.4 (2C), 127.2, 126.9, 73.7, 70.8, 67.5, 67.2, 60.7, 42.2; **IR** (cm^{-1}): 2930, 2849, 1486, 1098, 1079, 1008, 978, 911, 832, 760, 734, 695; **HRMS**: *m*/*z* calculated for $C_{18}H_{19}NO_2Na^+$ $[M+Na]^+$: 304.1308; found: 304.1311.

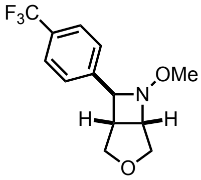

(1*R,5*S**,7*S**)-6-Methoxy-7-(4-(trifluoromethyl)phenyl)-3-oxa-6-azabicyclo[3.2.0]heptane (23):** Prepared according to GP-6a using **S23** (68 mg, 0.25 mmol, 1.0 equiv.), **17**•PF_6 (1.4 mg, 0.5 mol%) and THF (25 mL) with a reaction time of 0.5 h. The diastereomeric ratio was determined to be >20:1 by ^{1}H NMR analysis of the crude mixture. Purification by flash column chromatography (5-20% EtOAc/hexanes) afforded the pure title compound as clear oil (66 mg, 97%; combined yield). **^{1}H NMR** (700 MHz, $CDCl_3$): δ 7.61 (d, *J* = 8.1 Hz, 2H), 7.53 (d, *J* = 8.1 Hz, 2H), 4.75 (d, *J* = 10.6 Hz, 1H), 4.54 (t, *J* = 5.5 Hz, 1H), 4.45 (d, *J* = 5.7 Hz, 1H), 3.97 (d, *J* = 9.5 Hz, 1H), 3.57 (dd, *J* = 10.6, 5.2 Hz, 1H), 3.52 (dd, *J* = 9.5, 3.5 Hz, 1H), 3.44 (s, 3H), 2.68 (td, *J* = 5.8, 3.6 Hz, 1H); **^{13}C NMR** (176 MHz, $CDCl_3$): δ 145.9, 129.8 (q, *J* = 32.3 Hz), 126.6, 125.5 (q, *J* = 3.8 Hz), 124.3 (q, *J* = 272 Hz), 73.1, 70.7, 67.6, 67.1, 60.6, 42.2; **IR** (cm^{-1}): 2940, 2854, 1620, 1417, 1322, 1161, 1118, 1064, 1017, 913, 832, 807, 712, 639; **HRMS**: *m*/*z* calculated for $C_{13}H_{14}F_3NO_2H^+$ $[M+H]^+$: 274.1049; found: 274.1044.

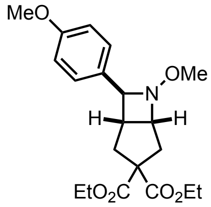

Diethyl (1*S,5*R**,7*S**)-6-methoxy-7-(4-methoxyphenyl)-6-azabicyclo[3.2.0]heptane-3,3-dicarboxylate (24):** Prepared according to GP-6a using **S24** (94 mg, 0.25 mmol, 1.0 equiv.), **17**•PF_6 (1.4 mg, 0.5 mol%) and THF (25 mL) with a reaction time of 1.5 h. The diastereomeric ratio was determined to be 20:1 by ^{1}H NMR analysis of the crude mixture. Purification by flash column chromatography (2-30% EtOAc/hexanes) afforded the pure title compound as clear oil (68 mg, 72%; combined yield). **^{1}H NMR** (400 MHz, C_6D_6): δ 7.37 (d, *J* = 8.6 Hz, 2H), 6.83 (d, *J* = 8.6 Hz, 2H), 4.57 (d, *J* = 6.0 Hz, 1H), 4.52 (q, *J* = 6.9 Hz, 1H), 4.13 – 4.02 (m, 2H), 3.98 – 3.87 (m, 2H), 3.41 (dd, *J* = 14.7, 4.6 Hz, 1H), 3.32 (s, 3H), 3.31 (s, 3H), 2.89 – 2.79 (m, 2H), 2.39 (qd, *J* = 7.1, 6.2, 1.8 Hz, 1H), 2.29 (dd, *J* = 14.0, 7.9 Hz, 1H), 0.99 (t, *J* = 7.1 Hz, 3H), 0.89 (t, *J* = 7.1 Hz, 3H); **^{13}C NMR** (126 MHz, C_6D_6): δ 172.3, 171.7, 159.8, 134.2, 128.5, 114.2, 76.6, 69.9, 63.9, 61.52, 61.49, 60.5, 54.8, 42.6, 38.5, 34.7, 14.1, 14.0; **IR** (cm^{-1}): 2937, 1725, 1611, 1513, 1464, 1443, 1300, 1244, 1206, 1178, 1094, 1062, 1034, 859, 809; **HRMS**: *m*/*z* calculated for $C_{20}H_{27}NO_6H^+$ $[M+H]^+$: 378.1911; found: 378.1917.

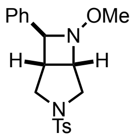

(1*R,5*S**,7*S**)-6-Methoxy-7-phenyl-3-tosyl-3,6-diazabicyclo[3.2.0]heptane (25):** Prepared according to GP-6a using **S25** (90 mg, 0.25 mmol, 1.0 equiv.), **17•**PF_6 (1.4 mg, 0.5 mol%) and THF (25 mL) with a reaction time of 0.5 h. The diastereomeric ratio was determined to be 12:1 by ^{1}H NMR analysis of the crude mixture. Purification by flash column chromatography (10-30% EtOAc/hexanes) afforded the pure title compound as white solid (83 mg, 92%; combined yield). **^{1}H NMR** (700 MHz, $CDCl_3$): δ 7.77 (d, *J* = 8.2 Hz, 2H), 7.39 – 7.32 (m, 6H), 7.30 – 7.26 (m, 1H), 4.51 (d, *J* = 6.0 Hz, 1H), 4.40 – 4.33 (m, 2H), 3.61 (d, *J* = 10.0 Hz, 1H), 3.40 (s, 3H), 2.82 – 2.75 (m, 2H), 2.57 (q, *J* = 5.8 Hz, 1H), 2.45 (s, 3H); **^{13}C NMR** (176 MHz, $CDCl_3$): δ 143.9, 141.3, 132.5, 129.8, 128.6, 128.1, 127.8, 126.5, 73.8, 66.2, 60.8, 51.5, 46.4, 40.5, 21.7; **IR** (cm^{-1}): 2936, 1597, 1466, 1343, 1158, 1093, 1055, 1023, 813, 735, 699, 665; **HRMS**: *m*/*z* calculated for $C_{19}H_{22}N_2O_3SNa^+$ [M+Na]$^+$: 381.1243; found: 381.1245.

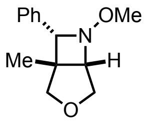

(1*R,5*S**,7*S**)-6-Methoxy-1-methyl-7-phenyl-3-oxa-6-azabicyclo[3.2.0]heptane (26):** Prepared according to GP-6a using **S26** (55 mg, 0.25 mmol, 1.0 equiv.), **17•**PF_6 (1.4 mg, 0.5 mol%) and THF (25 mL) with a reaction time of 1.5 h. The diastereomeric ratio was determined to be 1.6:1 by ^{1}H NMR analysis of the crude mixture. Purification by flash column chromatography (1-5% EtOAc/hexanes) afforded the pure title compound as clear oil (51 mg, 93%; combined yield). **(1*R**,5*S**,7*S**) Diastereomer (major): ^{1}H NMR** (700 MHz, $CDCl_3$): δ 7.41 (d, *J* = 7.5 Hz, 2H), 7.36 (t, *J* = 7.6 Hz, 2H), 7.27 (t, *J* = 7.4 Hz, 1H), 4.48 (s, 1H), 4.12 (d, *J* = 9.8 Hz, 1H), 3.83 (d, *J* = 2.8 Hz, 1H), 3.54 – 3.50 (m, 5H), 2.98 (d, *J* = 9.8 Hz, 1H), 1.44 (s, 3H); **^{13}C NMR** (176 MHz, $CDCl_3$): δ 138.7, 128.5, 127.5, 126.8, 77.6, 77.2, 73.0, 72.5, 61.8, 45.0, 22.7; **IR** (cm^{-1}): 2952, 2894, 2844, 1495, 1466, 1452, 1062, 1032, 1023, 911, 744, 721; **HRMS**: *m*/*z* calculated for $C_{13}H_{17}NO_2H^+$ [M+H]$^+$: 220.1332; found: 220.1333; **(1*R**,5*S**,7*R**) Diastereomer (minor): ^{1}H NMR** (500 MHz, $CDCl_3$): δ 7.39 (d, *J* = 7.1 Hz, 2H), 7.35 (t, *J* = 7.6 Hz, 2H), 7.27 (t, *J* = 7.1 Hz, 1H), 4.72 (d, *J* = 10.7 Hz, 1H), 4.58 (s, 1H), 4.05 (d, *J* = 5.1 Hz, 1H), 3.83 (d, *J* = 9.1 Hz, 1H), 3.68 (dd, *J* = 10.7, 5.1 Hz, 1H), 3.48 (s, 3H), 3.29 (d, *J* = 9.1 Hz, 1H), 0.86 (s, 3H); **^{13}C NMR** (176 MHz, $CDCl_3$): δ 139.5, 128.3, 127.3, 126.8, 76.8, 75.3, 72.3, 67.8, 60.8, 46.2, 14.3; **IR** (cm^{-1}): 2935, 2849, 1495, 1467, 1451, 1056, 1032, 1008, 943, 911, 734, 701; **HRMS**: *m*/*z* calculated for $C_{13}H_{17}NO_2H^+$ [M+H]$^+$: 220.1332; found: 220.1331.

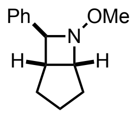

(1*S,5*R**,7*S**)-6-Methoxy-7-phenyl-6-azabicyclo[3.2.0]heptane (27):** Prepared according to GP-6b using **S27** (0.50 g, 2.50 mmol, 1.0 equiv.), **17•**PF_6 (14 mg, 0.5 mol%) and THF (250 mL) with a reaction time of 4 h. The diastereomeric ratio was determined to be >20:1 by ^{1}H NMR analysis of the crude mixture. Purification by flash column chromatography (1-5% EtOAc/hexanes) afforded the pure title compound as pale-yellow oil (0.49 g, 98%; combined yield). **^{1}H NMR** (700 MHz, $CDCl_3$): δ 7.39 (d, *J* = 7.3 Hz, 2H), 7.34 (t, *J* = 7.7 Hz, 2H), 7.25 (t, *J* = 7.3 Hz, 1H), 4.40 (t, *J* = 6.5 Hz, 1H), 4.15 (d, *J* = 5.8 Hz, 1H), 3.44 (s, 3H), 2.54 (q, *J* = 5.8 Hz, 1H), 2.48 – 2.43 (m, 1H), 2.00 – 1.93 (m, 2H), 1.82 – 1.77 (m, 1H), 1.62 – 1.50 (m, 2H); **^{13}C NMR** (176 MHz, $CDCl_3$): δ 143.1, 128.4, 127.1, 126.2, 74.0, 68.4, 60.5, 42.5, 30.8, 25.99, 25.95; **IR** (cm^{-1}): 2934, 1853, 1494, 1465, 1451, 1325, 1267, 1179, 1058, 1022, 970, 944, 907, 833, 751, 734, 697; **HRMS**: *m*/*z* calculated for $C_{13}H_{17}NOH^+$ $[M+H]^+$: 204.1383; found: 204.1376.

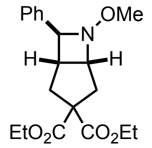

Diethyl (1*S,5*R**,7*S**)-6-Methoxy-7-phenyl-6-azabicyclo[3.2.0]heptane-3,3-dicarboxylate (28):** Prepared according to GP-6a using **S28** (87 mg, 0.25 mmol, 1.0 equiv.), **17•**PF_6 (1.4 mg, 0.5 mol%) and THF (25 mL) with a reaction time of 0.5 h. The diastereomeric ratio was determined to be 13:1 by ^{1}H NMR analysis of the crude mixture. Purification by flash column chromatography (5-10% EtOAc/hexanes) afforded the pure title compound as clear oil (84 mg, 97%; combined yield). **^{1}H NMR** (500 MHz, $CDCl_3$): δ 7.39 (d, *J* = 7.5 Hz, 2H), 7.34 (t, *J* = 7.5 Hz, 2H), 7.29 – 7.24 (m, 1H), 4.50 (q, *J* = 6.6 Hz, 1H), 4.34 (d, *J* = 6.0 Hz, 1H), 4.30 – 4.23 (m, 2H), 4.23 – 4.14 (m, 2H), 3.38 (s, 3H), 3.04 (dd, *J* = 14.7, 4.8 Hz, 1H), 2.62 (d, *J* = 14.4 Hz, 1H), 2.55 (q, *J* = 7.4 Hz, 2H), 2.33 (dd, *J* = 14.2, 7.9 Hz, 1H), 1.30 (t, *J* = 7.1 Hz, 3H), 1.25 (t, *J* = 7.1 Hz, 3H); **^{13}C NMR** (126 MHz, $CDCl_3$): δ 172.5, 171.7, 141.3, 128.5, 127.6, 126.7, 76.6, 69.5, 63.6, 61.9, 61.8, 60.5, 41.5, 38.8, 34.2, 14.3, 14.2; **IR** (cm^{-1}): 2980, 2938, 1726, 1445, 1366, 1252, 1180, 1094, 1061, 1039, 934, 746, 698; **HRMS**: *m*/*z* calculated for $C_{19}H_{25}NO_5Na^+$ $[M+Na]^+$: 370.1625; found: 370.1629.

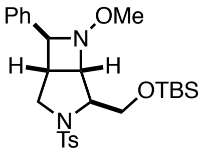

(1*R,4*S**,5*S**,7*S**)-4-(((*Tert*-butyldimethylsilyl)oxy)methyl)-6-methoxy-7-phenyl-3-tosyl-3,6-diazabicyclo[3.2.0]heptane (29):** Prepared according to GP-6a using

S29 (126 mg, 0.25 mmol, 1.0 equiv.), **17**•PF_6 (1.4 mg, 0.5 mol%) and THF (25 mL) with a reaction time of 0.5 h. The diastereomeric ratio was determined to be 2.5:1 by ^{1}H NMR analysis of the crude mixture. Purification by flash column chromatography (2-20% diethyl ether/pentane) afforded the pure title compound as clear oil (120 mg, 95%; combined yield). **^{1}H NMR** (500 MHz, $CDCl_3$): δ 7.87 (d, *J* = 8.2 Hz, 2H), 7.35 (d, *J* = 8.2 Hz, 2H), 7.28 (m, 2H), 7.24 (m, 1H), 7.06 (d, *J* = 7.0 Hz, 2H), 4.79 (t, *J* = 4.0 Hz, 1H), 4.46 (d, *J* = 6.1 Hz, 1H), 3.85 (d, *J* = 4.0 Hz, 2H), 3.53 – 3.44 (m, 3H), 3.33 (s, 3H), 2.50 (tt, *J* = 6.0, 2.4 Hz, 1H), 2.43 (s, 3H), 0.86 (s, 9H), 0.06 (d, *J* = 15.9 Hz, 6H); **^{13}C NMR** (176 MHz, $CDCl_3$): δ 143.5, 140.8, 137.5, 129.9, 128.5, 127.8, 127.6, 126.5, 73.1, 71.2, 66.2, 60.4, 58.8, 51.8, 41.2, 25.9, 21.7, 18.2, -5.36, -5.43; **IR** (cm^{-1}): 2929, 2883, 2856, 1463, 1345, 1251, 1187, 1156, 1091, 1033, 1005, 956, 869, 831, 813, 776, 751, 699, 666; **HRMS**: *m*/*z* calculated for $C_{26}H_{38}N_2O_4SSiH^+$ [M+H]$^+$: 503.2394; found: 503.2394.

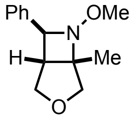

(1*R,5*S**,7*S**)-6-Methoxy-5-methyl-7-phenyl-3-oxa-6-azabicyclo[3.2.0]heptane (30):** Prepared according to GP-6a using **S30** (55 mg, 0.25 mmol, 1.0 equiv.), **17**•PF_6 (2.8 mg, 1 mol%) and degassed THF (25 mL) with a reaction time of 18 h. The diastereomeric ratio was determined to be 17:1 by ^{1}H NMR analysis of the crude mixture. Purification by flash column chromatography (5-15% EtOAc/hexanes) afforded the pure title compound as clear oil (50 mg, 91%; combined yield). **^{1}H NMR** (700 MHz, $CDCl_3$): δ 7.43 (d, *J* = 7.3 Hz, 2H), 7.36 (t, *J* = 7.6 Hz, 2H), 7.28 (t, *J* = 7.3 Hz, 1H), 4.73 (d, *J* = 10.1 Hz, 1H), 4.28 (d, *J* = 5.8 Hz, 1H), 3.93 (d, *J* = 9.4 Hz, 1H), 3.63 (dd, *J* = 9.4, 3.5 Hz, 1H), 3.49 (s, 3H), 3.37 (d, *J* = 10.1 Hz, 1H), 2.23 (dd, *J* = 5.7, 3.5 Hz, 1H), 1.51 (s, 3H); **^{13}C NMR** (126 MHz, $CDCl_3$): δ 142.4, 128.5, 127.4, 126.6, 74.3, 71.8, 70.9, 70.7, 62.2, 48.4, 23.5; **IR** (cm^{-1}): 2935, 2851, 1452, 1375, 1192, 1130, 1061, 1045, 939, 913, 792, 746, 697; **HRMS**: *m*/*z* calculated for $C_{13}H_{17}NO_2H^+$ [M+H]$^+$: 220.1332; found: 220.1329.

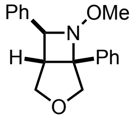

(1*R,5*R**,7*S**)-6-Methoxy-5,7-diphenyl-3-oxa-6-azabicyclo[3.2.0]heptane (31):** Prepared according to GP-6a using **S31** (70 mg, 0.25 mmol, 1.0 equiv.), **17**•PF_6 (2.8 mg, 1 mol%) and degassed THF (25 mL) with a reaction time of 18 h. The diastereomeric ratio was determined to be 12:1 by ^{1}H NMR analysis of the crude mixture. Purification by flash column chromatography (1-10% EtOAc/hexanes) afforded the pure title compound as clear oil (68 mg, 97%; combined yield). **^{1}H NMR** (700 MHz, $CDCl_3$): δ 7.51 (d, *J* = 7.1 Hz, 2H), 7.42 – 7.38 (m, 4H), 7.33 (t, *J* = 7.6 Hz,

2H), 7.29 (t, *J* = 7.4 Hz, 1H), 7.28 – 7.24 (m, 1H), 5.03 (d, *J* = 10.5 Hz, 1H), 4.47 (d, *J* = 5.7 Hz, 1H), 4.12 (d, *J* = 9.6 Hz, 1H), 3.91 (dd, *J* = 9.6, 3.5 Hz, 1H), 3.79 (d, *J* = 10.5 Hz, 1H), 3.59 (s, 3H), 2.60 (dd, *J* = 5.6, 3.5 Hz, 1H); **^{13}C NMR** (176 MHz, $CDCl_3$): δ 143.0, 142.2, 128.56, 128.55, 127.6, 127.1, 126.9, 125.5, 79.3, 73.6, 71.9, 71.6, 61.6, 51.0; **IR** (cm^{-1}): 2933, 2851, 1602, 1492, 1446, 1262, 1059, 1043, 1022, 964, 914, 840, 748, 696; **HRMS**: *m*/*z* calculated for $C_{18}H_{19}NO_2H^+$ $[M+H]^+$: 282.1489; found: 282.1491.

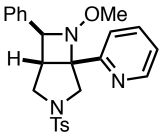

(1*R,5*S**,7*S**)-6-Methoxy-7-phenyl-5-(pyridin-2-yl)-3-tosyl-3,6-diazabicyclo[3.2.0]heptane (32):** Prepared according to GP-6a using **S32** (109 mg, 0.25 mmol, 1.0 equiv.), **17**•PF_6 (1.4 mg, 0.5 mol%) and degassed THF (25 mL) with a reaction time of 17 h. The diastereomeric ratio was determined to be >20:1 by ^{1}H NMR analysis of the crude mixture. Purification by flash column chromatography (10-60% EtOAc/hexanes) afforded the pure title compound as clear oil (80 mg, 74%; combined yield). **^{1}H NMR** (500 MHz, $CDCl_3$): δ 8.39 (d, *J* = 4.7 Hz, 1H), 7.81 (d, *J* = 7.9 Hz, 3H), 7.70 (t, *J* = 7.7 Hz, 1H), 7.35 (dt, *J* = 15.5, 7.5 Hz, 6H), 7.31 – 7.23 (m, 1H), 7.17 – 7.10 (m, 1H), 4.61 (d, *J* = 10.8 Hz, 1H), 4.56 (d, *J* = 6.0 Hz, 1H), 3.73 (d, *J* = 10.0 Hz, 1H), 3.58 (d, *J* = 10.8 Hz, 1H), 3.54 (s, 3H), 3.20 (dd, *J* = 10.0, 4.5 Hz, 1H), 2.55 – 2.49 (m, 1H), 2.46 (s, 3H); **^{13}C NMR** (176 MHz, $CDCl_3$): δ 161.6, 149.2, 143.7, 141.5, 136.7, 133.4, 129.8, 128.6, 128.1, 127.8, 126.9, 122.2, 120.8, 79.3, 71.2, 61.8, 52.0, 50.9, 48.4, 21.7; **IR** (cm^{-1}): 2934, 1590, 1465, 1161, 1091, 1027, 1012, 911, 815, 784, 749, 731, 698, 666; **HRMS**: *m*/*z* calculated for $C_{24}H_{25}N_3O_3SNa^+$ $[M+Na]^+$: 458.1509; found: 458.1507.

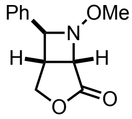

(1*R,5*S**,7*S**)-6-methoxy-7-phenyl-3-oxa-6-azabicyclo[3.2.0]heptan-4-one (33):** Prepared according to GP6-a, the reaction was conducted in a sealed 30-mL microwave vial using **S33** (55 mg, 0.25 mmol, 1.0 equiv.), **17**•PF_6 (2.8 mg, 1 mol%) and degassed MeCN (25 mL) at 82 °C with a reaction time of 70 h. The diastereomeric ratio was determined to be 14:1 by ^{1}H NMR analysis of the crude mixture. Purification by flash column chromatography (5-40% EtOAc/hexanes) afforded the pure title compound as yellow oil (33 mg, 60%; combined yield; 75% brsm). **^{1}H NMR** (500 MHz, $CDCl_3$): δ 7.43 – 7.37 (m, 4H), 7.36 – 7.32 (m, 1H), 4.57 (ddd, *J* = 12.8, 6.1, 1.2 Hz, 2H), 4.40 (d, *J* = 9.7 Hz, 1H), 4.34 (dd, *J* = 9.8, 5.0 Hz, 1H), 3.54 (s, 3H), 2.87 (q, *J* = 5.7 Hz, 1H); **^{13}C NMR** (126 MHz, $CDCl_3$): δ 171.9, 139.3, 128.9, 128.6, 126.7, 75.6, 70.5, 63.2, 61.2, 37.1; **IR** (cm^{-1}): 2934, 1774, 1456, 1371, 1266, 1160, 1046, 993, 974,

943, 731, 698; **HRMS**: *m*/*z* calculated for $C_{12}H_{13}NO_3Na^+$ $[M+Na]^+$: 242.0788; found: 242.0787.

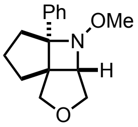

(3a*S,4a*S**,7a*S**)-4-methoxy-4a-phenylhexahydro-1*H*,3*H*-cyclopenta[*b*]furo[3,4-*c*]azete (34):** Prepared according to GP-6a using **S34** (61 mg, 0.25 mmol, 1.0 equiv.), **17•**PF_6 (2.8 mg, 1 mol%) and degassed THF (25 mL) with a reaction time of 15 h. The diastereomeric ratio was determined to be >20:1 by ^{1}H NMR analysis of the crude mixture. Purification by flash column chromatography (2-10% EtOAc/hexanes) afforded the pure title compound as white solid (60 mg, 98%; combined yield). **^{1}H NMR** (700 MHz, $CDCl_3$): δ 7.43 (dd, *J* = 8.2, 1.1 Hz, 2H), 7.35 (t, *J* = 7.8 Hz, 2H), 7.20 (tt, *J* = 7.2, 1.3 Hz, 1H), 4.02 (d, *J* = 10.0 Hz, 1H), 3.76 (d, *J* = 3.7 Hz, 1H), 3.61 (s, 3H), 3.48 (d, *J* = 10.0 Hz, 1H), 3.36 (dd, *J* = 10.0, 3.7 Hz, 1H), 3.18 (d, *J* = 10.0 Hz, 1H), 2.82 (dd, *J* = 14.8, 6.8 Hz, 1H), 2.11 – 2.02 (m, 2H), 1.94 – 1.87 (m, 1H), 1.87 – 1.81 (m, 2H); **^{13}C NMR** (176 MHz, $CDCl_3$): δ 144.2, 128.2, 126.3, 126.2, 80.9, 73.5, 72.8, 69.3, 61.4, 59.0, 35.6, 32.6, 25.7; **IR** (cm^{-1}): 2959, 2941, 2841, 1492, 1461, 1446, 1061, 1043, 992, 902, 786, 757, 736, 717, 704, 653; **HRMS**: *m*/*z* calculated for $C_{15}H_{19}NO_2Na^+$ $[M+Na]^+$: 268.1308; found: 268.1314.

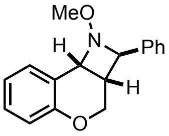

(2*R,2a*S**,8b*R**)-1-Methoxy-2-phenyl-1,2a,3,8b-tetrahydro-2*H*-chromeno[4,3-*b*]azete (35):** Prepared according to GP-6a using **S35** (67 mg, 0.25 mmol, 1.0 equiv.), **17•**PF_6 (2.8 mg, 1 mol%) and degassed THF (25 mL) with a reaction time of 72 h. The diastereomeric ratio was determined to be 7:1 by ^{1}H NMR analysis of the crude mixture. Purification by flash column chromatography (1-10% EtOAc/hexanes) afforded the pure title compound as clear oil (28 mg, 42%). **^{1}H NMR** (700 MHz, $CDCl_3$): δ 7.51 (d, *J* = 7.3 Hz, 2H), 7.39 (t, *J* = 7.6 Hz, 2H), 7.34 – 7.27 (m, 2H), 7.24 (dd, *J* = 7.8, 1.4 Hz, 1H), 7.06 – 7.02 (m, 2H), 4.97 (d, *J* = 7.6 Hz, 1H), 4.61 (d, *J* = 8.4 Hz, 1H), 4.17 (dd, *J* = 11.9, 1.5 Hz, 1H), 3.90 (dd, *J* = 11.9, 1.9 Hz, 1H), 3.48 (s, 3H), 2.68 – 2.63 (m, 1H); **^{13}C NMR** (176 MHz, $CDCl_3$): δ 155.4, 141.0, 133.4, 129.4, 128.6, 127.8, 126.9, 121.2, 118.9, 117.8, 70.5, 63.4, 61.2, 59.9, 36.8; **IR** (cm^{-1}): 2823, 1581, 1486, 1447, 1220, 1209, 1079, 1051, 1030, 1001, 940, 929, 747, 699; **HRMS**: *m*/*z* calculated for $C_{17}H_{17}NO_2Na^+$ $[M+Na]^+$: 290.1151; found: 290.1150.

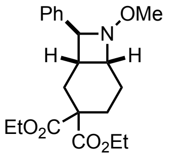

Diethyl (1*S,6*R**,8*S**)-7-Methoxy-8-phenyl-7-azabicyclo[4.2.0]octane-3,3-dicarboxylate (36):** Prepared according to GP-6a using **S36** (90 mg, 0.25 mmol, 1.0 equiv.), **17**•PF_6 (2.8 mg, 1 mol%) and degassed THF (25 mL) with a reaction time of 72 h. The diastereomeric ratio was determined to be 3.5:1 by ^{1}H NMR analysis of the crude mixture. Purification by flash column chromatography (5-10% EtOAc/hexanes) afforded the pure title compound as clear oil (84 mg, 93%; combined yield). Characterization data was obtained for a 5:1 mixture of (1*S**,6*R**,8*S**) diastereomer (major) and (1*S**,6*R**,8*R**) diastereomer (minor). **^{1}H NMR** (700 MHz, $CDCl_3$): δ 7.48 (d, *J* = 6.1 Hz, 9H; major+minor), 7.39 – 7.31 (m, 15H; major+minor), 7.29 – 7.24 (m, 6H; major+minor), 4.47 (d, *J* = 8.0 Hz, 5H; major), 4.29 (q, *J* = 7.2 Hz, 10H; major), 4.21 – 4.11 (m, 15H; major), 4.04 (d, *J* = 9.4 Hz, 1H; minor), 3.98 (q, *J* = 7.6 Hz, 5H; minor), 3.49 (s, 3H; minor), 3.44 (s, 15H; major), 2.80 (td, *J* = 11.2, 3.2 Hz, 1H; minor), 2.64 (dd, *J* = 12.5, 3.1 Hz, 1H; minor), 2.59 – 2.54 (m, 1H; minor), 2.52 – 2.34 (m, 10H; major+minor), 2.21 – 2.01 (m, 13H; major+minor), 1.97 – 1.73 (m, 15H; major+minor), 1.44 – 1.36 (m, 1H minor), 1.30 (t, *J* = 7.1 Hz, 16H; major+minor), 1.23 (td, *J* = 7.1, 1.6 Hz, 20H; major+minor); **^{13}C NMR** (176 MHz, $CDCl_3$): δ 172.5, 172.0, 171.7, 170.9, 141.6, 140.1, 128.5, 128.4, 127.7, 127.4, 126.9, 126.5, 78.8, 74.0, 72.8, 61.73, 61.66, 61.59, 61.56, 60.9, 60.8 (2C), 56.0, 53.4, 36.5, 34.9, 32.9, 30.7, 28.6, 27.6, 26.2, 17.8, 14.21, 14.15, 14.13, 14.11; **IR** (cm^{-1}): 2938, 1726, 1449, 1367, 1228, 1175, 1111, 1024, 952, 860, 733, 699; **HRMS**: *m*/*z* calculated for $C_{20}H_{27}NO_5Na^+$ $[M+Na]^+$: 384.1781; found: 384.1784.

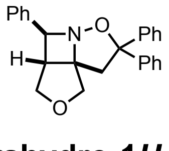

(3a*R,4*S**,8a*S**)-4,7,7-Triphenyltetrahydro-1*H*,3*H*-furo[3',4':2,3]azeto[1,2-*b*]isoxazole (39):** Prepared according to GP-6a using **38** (94 mg, 0.25 mmol, 1.0 equiv.), **17**•PF_6 (2.8 mg, 1 mol%) and degassed THF (25 mL) with a reaction time of 14.5 h. The diastereomeric ratio was determined to be 4:1 by ^{1}H NMR analysis of the crude mixture. Purification by flash column chromatography (10-60% EtOAc/hexanes) afforded the pure title compound as off-white solid (82 mg, 87%; combined yield). **^{1}H NMR** (700 MHz, $CDCl_3$): δ 7.45 – 7.42 (m, 4H), 7.39 – 7.33 (m, 3H), 7.30 (t, *J* = 7.8 Hz, 2H), 7.21 (t, *J* = 7.3 Hz, 1H), 7.14 – 7.10 (m, 3H), 7.02 – 6.99 (m, 2H), 4.84 (d, *J* = 6.4 Hz, 1H), 4.04 (d, *J* = 9.8 Hz, 1H), 3.71 (dd, *J* = 9.9, 5.1 Hz, 1H), 3.69 (d, *J* = 10.2 Hz, 1H), 3.54 (t, *J* = 5.6 Hz, 1H), 3.37 (d, *J* = 10.2 Hz, 1H), 3.18 (s, 2H); **^{13}C NMR** (176 MHz, $CDCl_3$): δ 144.3, 143.2, 135.7, 129.6, 128.5, 128.14, 128.10, 127.9, 127.3, 127.2, 126.06, 126.06, 89.4, 78.3, 74.7, 71.5, 68.2, 48.0, 47.8;

IR (cm^{-1}): 3058, 2968, 2850, 1598, 1492, 1448, 1265, 1189, 1125, 1043, 1032, 985, 912, 866, 731, 694; **HRMS**: *m*/*z* calculated for $C_{25}H_{23}NO_2H^+$ [M+H]$^+$: 370.1802; found: 370.1799.

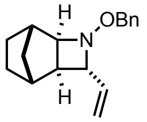

(1*S,2*R**,4*R**,5*R**,6*R**)-3-(Benzyloxy)-4-vinyl-3-azatricyclo[4.2.1.0^{2,5}]nonane (41):** Prepared according to GP-6a using **40** (64 mg, 0.25 mmol, 1.0 equiv.), **17•**PF_6 (2.8 mg, 1 mol%) and degassed THF (25 mL) with a reaction time of 24 h. The diastereomeric ratio was determined to be 4:1 (exo/endo >20:1) by ^{1}H NMR analysis of the crude mixture. Purification by flash column chromatography (0-10% EtOAc/hexanes) afforded the pure title compound as clear oil (25 mg, 39%; combined yield). Characterization data was obtained for the (1*S**,2*R**,4*R**,5*R**,6*R**) diastereomer (major). **^{1}H NMR** (700 MHz, $CDCl_3$): δ 7.34 – 7.30 (m, 4H), 7.28 – 7.24 (m, 1H), 5.96 (ddd, *J* = 17.1, 10.2, 6.8 Hz, 1H), 5.11 (d, *J* = 17.1 Hz, 1H), 5.01 (d, *J* = 10.3 Hz, 1H), 4.60 (d, *J* = 11.7 Hz, 1H), 4.56 (d, *J* = 11.7 Hz, 1H), 3.90 (t, *J* = 5.4 Hz, 1H), 3.70 (d, *J* = 5.6 Hz, 1H), 2.46 (s, 1H), 2.17 (d, *J* = 10.6 Hz, 2H), 1.98 (t, *J* = 4.9 Hz, 1H), 1.49 – 1.42 (m, 2H), 1.30 (d, *J* = 10.5 Hz, 1H), 1.02 – 0.91 (m, 2H); **^{13}C NMR** (176 MHz, $CDCl_3$): δ 139.1, 138.8, 128.4, 128.3, 127.6, 114.6, 75.8, 73.4, 71.5, 43.2, 37.9, 37.8, 34.1, 27.6, 25.2; **IR** (cm^{-1}): 2952, 1871, 1453, 1364, 1023, 987, 916, 846, 733, 695; **HRMS**: *m*/*z* calculated for $C_{17}H_{21}NOH^+$ [M+H]$^+$: 256.1696; found: 256.1700.

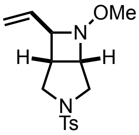

(1*R,5*S**,7*R**)-6-Methoxy-3-tosyl-7-vinyl-3,6-diazabicyclo[3.2.0]heptane (43):** Prepared according to GP-6a using **42** (77 mg, 0.25 mmol, 1.0 equiv.), **17•**PF_6 (1.4 mg, 0.5 mol%) and THF (25 mL) with a reaction time of 0.5 h. The diastereomeric ratio was determined to be 2:1 by ^{1}H NMR analysis of the crude mixture. Purification by flash column chromatography (25% EtOAc/hexanes) afforded the pure title compound as pale-yellow solid (76 mg, 99%; combined yield). **(1*R**,5*S**,7*R**) Diastereomer (major): ^{1}H NMR** (500 MHz, $CDCl_3$): δ 7.73 (d, *J* = 8.2 Hz, 2H), 7.34 (d, *J* = 8.0 Hz, 2H), 5.94 (ddd, *J* = 17.3, 10.3, 7.0 Hz, 1H), 5.23 (d, *J* = 17.2 Hz, 1H), 5.12 (d, *J* = 10.3 Hz, 1H), 4.29 – 4.21 (m, 2H), 3.97 (t, *J* = 6.4 Hz, 1H), 3.49 (d, *J* = 10.0 Hz, 1H), 3.42 (s, 3H), 2.72 (td, *J* = 11.6, 10.6, 5.8 Hz, 2H), 2.48 (q, *J* = 5.7 Hz, 1H), 2.44 (s, 3H); **^{13}C NMR** (126 MHz, $CDCl_3$): δ 143.9, 137.8, 132.6, 129.8, 128.1, 116.8, 73.2, 66.7, 60.8, 51.2, 46.6, 38.2, 21.7; **IR** (cm^{-1}): 2936, 2890, 1598, 1472, 1338, 1156, 1125, 1053, 1013, 928, 809, 708, 667; **HRMS**: *m*/*z* calculated for $C_{15}H_{20}N_2O_3SNa^+$ [M+Na]$^+$: 331.1087; found: 331.1089; **(1*R**,5*S**,7*S**) Diastereomer (minor): ^{1}H NMR** (500 MHz, $CDCl_3$): δ 7.73 (d, *J* = 8.2 Hz, 2H), 7.32 (d, *J* = 8.0 Hz,

2H), 5.76 (ddd, J = 16.9, 10.5, 6.1 Hz, 1H), 5.31 (d, J = 17.2 Hz, 1H), 5.23 (d, J = 10.5 Hz, 1H), 4.02 – 3.93 (m, 2H), 3.66 (d, J = 10.6 Hz, 1H), 3.51 (dd, J = 10.6, 2.5 Hz, 1H), 3.43 (s, 3H), 2.99 (qd, J = 8.7, 2.3 Hz, 1H), 2.89 – 2.82 (m, 2H), 2.43 (s, 3H); **^{13}C NMR** (126 MHz, $CDCl_3$): δ 143.6, 133.9, 133.6, 129.6, 128.0, 118.8, 70.1, 69.0, 61.9, 53.8, 46.9, 35.6, 21.8; **IR** (cm^{-1}): 2939, 2889, 1464, 1334, 1176, 1155, 1093, 1045, 1030, 988, 923, 812, 737, 665; **HRMS**: *m/z* calculated for $C_{15}H_{20}N_2O_3SNa^+$ $[M+Na]^+$: 331.1087; found: 331.1090.

Synthetic Modifications of Azetidine Products

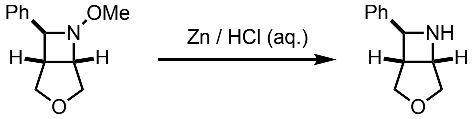

(1*R,5*S**,7*S**)-7-Phenyl-3-oxa-6-azabicyclo[3.2.0]heptane (46):** A 25-mL round-bottom flask equipped with a magnetic stir bar was charged with azetidine **16** (51 mg, 0.25 mmol, 1.0 equiv.). Then, 2 M aqueous HCl (5 mL) and zinc powder (82 mg, 1.25 mmol, 5 equiv.) were added sequentially and the resulting mixture stirred at 80 °C for 1 h. The reaction was cooled to 0 °C and 2 M aqueous NaOH was added dropwise until pH 10-12 was reached. The mixture was subsequently extracted with CH_2Cl_2 (3x) and the combined organic layers dried over Na_2SO_4, filtered and concentrated *in vacuo*. Purification by flash column chromatography (1-10% MeOH/CH_2Cl_2) afforded the pure title compound as yellow oil (38 mg, 87%). **^{1}H NMR** (500 MHz, $CDCl_3$): δ 7.43 (d, J = 7.3 Hz, 2H), 7.37 (t, J = 7.6 Hz, 2H), 7.28 – 7.24 (m, 1H), 4.60 (d, J = 4.5 Hz, 1H), 4.38 (dd, J = 6.3, 3.6 Hz, 1H), 4.16 (d, J = 9.7 Hz, 1H), 4.08 (d, J = 10.4 Hz, 1H), 3.57 (dd, J = 10.4, 3.6 Hz, 1H), 3.54 (dd, J = 9.7, 4.6 Hz, 1H), 3.07 (dt, J = 6.3, 4.6 Hz, 1H); **^{13}C NMR** (126 MHz, $CDCl_3$): δ 145.1, 128.7, 127.2, 125.9, 76.0, 72.9, 64.0, 60.4, 48.1; **IR** (cm^{-1}): 3314, 3025, 2929, 2844, 1603, 1491, 1452, 1338, 1165, 1094, 1070, 977, 909, 881, 735, 697; **HRMS**: *m/z* calculated for $C_{11}H_{13}NOH^+$ $[M+H]^+$: 176.1070; found: 176.1067.

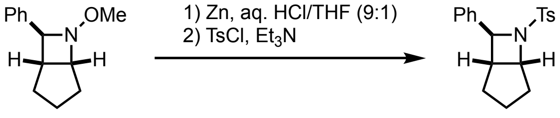

(1*S,5*R**,7*S**)-7-Phenyl-6-tosyl-6-azabicyclo[3.2.0]heptane (47):** A 100-mL round-bottom flask equipped with a magnetic stir bar was charged with **27** (178 mg, 0.88 mmol, 1.0 equiv.) and a 9:1 mixture (*v/v*) of 2 M HCl (aq.) and THF (20 mL). The mixture was heated to reflux until the substrate completely dissolved, then, Zn (286 mg, 4.38 mmol, 5.0 equiv.) was added and the mixture continued to reflux for 1.5 h. After cooling to 0 °C, 2 M NaOH (aq.) was added until pH 12 and the mixture subsequently extracted with CH_2Cl_2 (3x) and EtOAc (3x). The combined organic layers were dried over Na_2SO_4, filtered and concentrated *in vacuo*, then dried using high-vac

to afford the crude azetidine (quant. yield), which was used in the next step without further purification.

The crude azetidine was dissolved in CH_2Cl_2 (10 mL) and the solution cooled to 0 °C. Et_3N (0.13 mL, 0.96 mmol, 1.1 equiv.) and *p*-TsCl (184 mg, 0.96 mmol, 1.1 equiv.) were added sequentially and the solution allowed to warm up to rt and stirred for 1.5 h. Then, water was added, the organic layer separated and the aqueous layer extracted with CH_2Cl_2 (3x). The combined organic layers were dried over Na_2SO_4, filtered and concentrated *in vacuo*. Purification by flash column chromatography (5-15% EtOAc/hexanes) afforded the pure title compound as white solid (200 mg, 70%). **^{1}H NMR** (700 MHz, $CDCl_3$): δ 7.43 (d, *J* = 8.2 Hz, 2H), 7.22 (s, 5H), 7.10 (d, *J* = 8.0 Hz, 2H), 4.85 (t, *J* = 5.5 Hz, 1H), 4.65 (d, *J* = 4.1 Hz, 1H), 2.76 (q, *J* = 6.3 Hz, 1H), 2.57 (dd, *J* = 14.1, 5.6 Hz, 1H), 2.36 (s, 3H), 1.89 – 1.79 (m, 2H), 1.80 – 1.71 (m, 1H), 1.52 – 1.39 (m, 2H); **^{13}C NMR** (176 MHz, $CDCl_3$): δ 142.8, 140.2, 137.9, 129.3, 128.5, 127.9, 127.2, 126.9, 70.9, 68.9, 44.6, 32.2, 30.7, 24.1, 21.6; **IR** (cm^{-1}): 3030, 2952, 1598, 1495, 1455, 1338, 1186, 1152, 1121, 1090, 1059, 1028, 997, 951, 815, 754, 698, 665; **HRMS**: *m*/*z* calculated for $C_{19}H_{21}NO_2SNa^+$ $[M+Na]^+$: 350.1185; found: 350.1185.

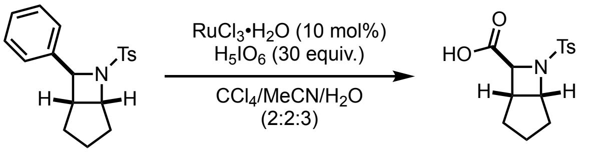

(1*S,5*R**,7*S**)-6-Tosyl-6-azabicyclo[3.2.0]heptane-7-carboxylic acid (48):** According to a procedure by Enders with minor modifications.[26] A 10-mL microwave vial equipped with a magnetic stir bar was charged with **47** (100 mg, 0.31 mmol, 1.0 equiv.). CCl_4 (0.7 mL), MeCN (0.7 mL) and water (1.0 mL) were added and the mixture stirred until all solids were dissolved. Next, periodic acid (879 mg, 4.6 mmol, 15.0 equiv.) and $RuCl_3$ hydrate (3.4 mg, 0.015 mmol, 0.05 equiv.) were added sequentially and the vial sealed with a rubber septa pierced with a needle to maintain an open atmosphere. The biphasic mixture was vigorously stirred for 24 h, before adding additional periodic acid (879 mg, 4.6 mmol, 15.0 equiv.) and $RuCl_3$ hydrate (3.4 mg, 0.015 mmol, 0.05 equiv.), and the reaction was continued to stir for 12 h. Et_2O was added and the mixture stirred for 0.5 h, before the addition of water. The organic layer was separated and the aqueous layer extracted with Et_2O (3x) and EtOAc (3x). The combined organic layers were dried over $MgSO_4$, filtered and concentrated *in vacuo*. Purification by flash column chromatography (94:5:1 CH_2Cl_2/MeOH/AcOH) afforded the pure title compound as pale-yellow oil (34 mg, 38%). **^{1}H NMR** (700 MHz, $CDCl_3$): δ 7.75 (d, *J* = 8.0 Hz, 2H), 7.35 (d, *J* = 7.9 Hz, 2H), 4.74 (t, *J* = 5.4 Hz, 1H), 4.19 (d, *J* = 4.1 Hz, 1H), 3.06 (q, *J* = 5.6 Hz, 1H), 2.46 (s, 3H), 2.31 (dd, *J* = 14.7, 5.9 Hz, 1H), 1.81 (dd, *J* = 13.7, 6.3 Hz, 1H), 1.71 (dt, *J* = 13.1, 6.6 Hz, 1H), 1.50 (h,

J = 13.5, 6.8 Hz, 1H), 1.33 (h, J = 19.6, 12.5, 5.7 Hz, 1H), 1.20 – 1.11 (m, 1H); **^{13}C NMR** (176 MHz, $CDCl_3$): δ 174.2, 144.2, 136.5, 129.9, 127.5, 69.5, 65.7, 39.9, 31.2, 30.6, 23.6, 21.8; **IR** (cm^{-1}): 2960, 1716, 1598, 1434, 1335, 1289, 1241, 1150, 1090, 1060, 1001, 907, 815, 727, 708, 674, 648; **HRMS**: *m*/*z* calculated for $C_{14}H_{17}NO_4SNa^+$ [M+Na]$^+$: 318.0770; found: 318.0769.

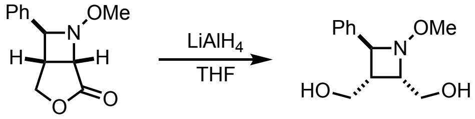

((2*S,3*R**,4*S**)-1-Methoxy-4-phenylazetidine-2,3-diyl)dimethanol (49):** A 10-mL round-bottom flask equipped with a magnetic stir bar was charged with $LiAlH_4$ (12 mg, 0.32 mmol, 2.6 equiv.) and THF (1.5 mL). After cooling to 0 °C, a solution of **33** (27 mg, 0.12 mmol, 1.0 equiv.) in THF (0.5 mL) was added dropwise. Then, the reaction mixture was allowed to warm up to rt and stirred for 3.5 h. Water was carefully added at 0 °C, followed by Rochelle salt solution (aq., sat.) and the resulting mixture allowed to gradually warm up to rt and stirred for 3 h. The resulting biphasic mixture was extracted with Et_2O (3x) and the combined organic layers dried over $MgSO_4$, filtered and concentrated *in vacuo*. Purification by flash column chromatography (30-95% EtOAc/hexanes) afforded the pure title compound as white solid (23 mg, 84%). **^{1}H NMR** (700 MHz, $CDCl_3$): δ 7.43 (d, J = 7.3 Hz, 2H), 7.36 (t, J = 7.6 Hz, 2H), 7.28 (t, J = 7.3 Hz, 1H), 4.76 (d, J = 9.0 Hz, 1H), 4.31 – 4.24 (m, 1H), 4.17 – 4.10 (m, 2H), 3.91 – 3.83 (m, 2H), 3.52 (s, 3H), 3.03 (t, J = 5.2 Hz, 1H), 2.90 (dd, J = 7.6, 4.4 Hz, 1H), 2.50 (bs, 1H); **^{13}C NMR** (176 MHz, $CDCl_3$): δ 141.2, 128.6, 127.7, 126.6, 73.4, 66.6, 62.1, 59.8, 59.6, 40.7; **IR** (cm^{-1}): 3319, 2935, 1596, 1495, 1453, 1370, 1266, 1155, 1091, 1020, 736, 698; **HRMS**: *m*/*z* calculated for $C_{12}H_{17}NO_3Na^+$ [M+Na]$^+$: 246.1101; found: 246.1105.

Representative NOE Data

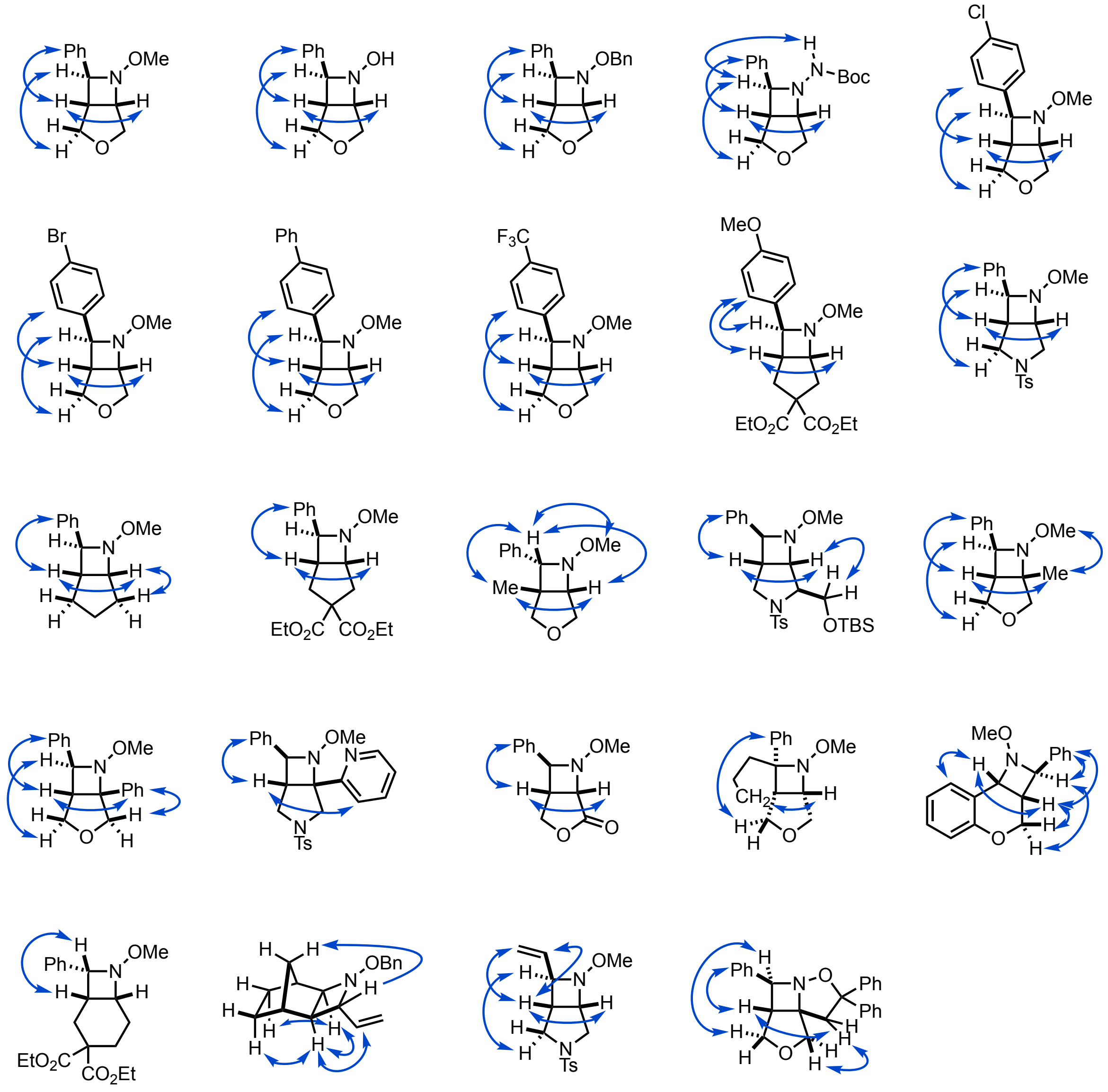

Supplementary Figure 6. Representative NOE data for the synthesized azetidine products

X-Ray Crystallographic Data

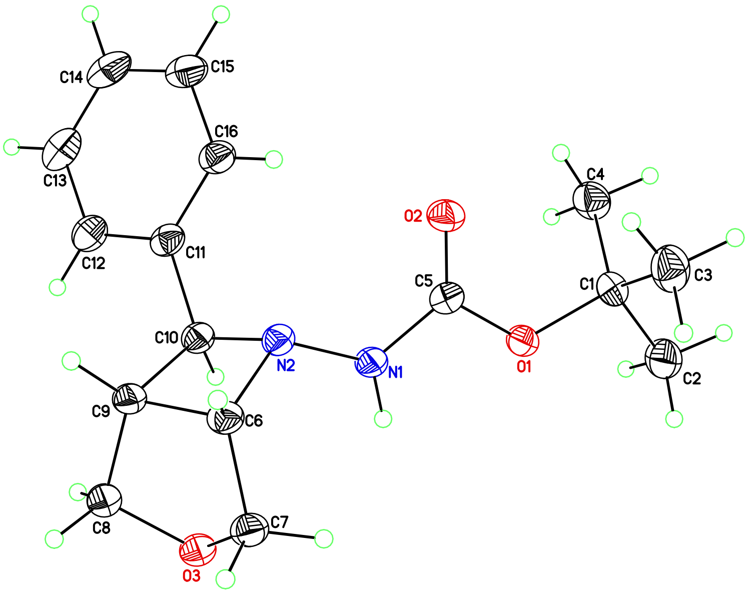

Supplementary Figure 7. Crystal structure of compound **19c**. X-ray crystallographic coordinates have been deposited at the Cambridge Crystallographic Data Centre (CCDC) with the accession code 1873931 [https://doi.org/10.5517/ccdc.csd.cc20wzd9]

Colorless blocks of *tert*-butyl ((1*R**,5*S**,7*S**)-7-phenyl-3-oxa-6-azabicyclo[3.2.0]heptan-6-yl)carbamate (**19c**) were grown via vapor diffusion (pentane/ethyl acetate) of the compound at ambient temperature. A crystal of dimensions 0.18 x 0.14 x 0.10 mm was mounted on a Rigaku AFC10K Saturn 944+ CCD-based X-ray diffractometer equipped with a low temperature device and Micromax-007HF Cu-target micro-focus rotating anode (λ = 1.54187 A) operated at 1.2 kW power (40 kV, 30 mA). The X-ray intensities were measured at 85(1) K with the detector placed at a distance 42.00 mm from the crystal. A total of 2028 images were collected with an oscillation width of 1.0° in ω. The exposure times were 1 sec. for the low angle images, 2 sec. for high angle. Rigaku d*trek images were exported to CrysAlisPro for processing and corrected for absorption. The integration of the data yielded a total of 23789 reflections to a maximum 2θ value of 138.67° of which 2901 were independent and 2802 were greater than 2σ(I). The final cell constants (Table 2) were based on the xyz centroids of 17770 reflections above 10σ(I). Analysis of the data showed negligible decay during data collection. The structure was solved and refined with the Bruker SHELXTL (version 2016/6) software package, using the space group P2(1)/c with Z = 4 for the formula $C_{16}H_{22}N_2O_3$. All non-hydrogen atoms were refined anisotropically with the hydrogen atoms placed in a combination of idealized and refined positions. Full matrix least-squares refinement based on F^2 converged at R1 = 0.0505 and wR2 = 0.1183 [based on I > 2sigma(I)], R1 = 0.0512 and wR2 = 0.1191 for all data. Additional details are presented in Table 2 and are given as

Supporting Information in a CIF file. Acknowledgement is made for funding from NSF grant CHE-0840456 for X-ray instrumentation.

G.M. Sheldrick (2015) "Crystal structure refinement with SHELXL", Acta Cryst., C71, 3-8 (Open Access).

CrystalClear Expert 2.0 r16, Rigaku Americas and Rigaku Corporation (2014), Rigaku Americas, 9009, TX, USA 77381-5209, Rigaku Tokyo, 196-8666, Japan.

CrysAlisPro 1.171.38.41 (Rigaku Oxford Diffraction, 2015).

Supplementary Table 2. Crystal data and structure refinement for *tert*-butyl ((1*R**,5*S**,7*S**)-7-phenyl-3-oxa-6-azabicyclo[3.2.0]heptan-6-yl)carbamate (**19c**).

Identification code	*tert*-butyl ((1*R**,5*S**,7*S**)-7-phenyl-3-oxa-6-azabicyclo[3.2.0]heptan-6-yl)carbamate
Empirical formula	C16 H22 N2 O3
Formula weight	290.35
Temperature	85(2) K
Wavelength	1.54184 A
Crystal system, space group	Monoclinic, P2(1)/c
Unit cell dimensions	a = 9.81308(10) A alpha = 90 deg.
	b = 16.02162(18) A beta = 96.5969(9) deg.
	c = 10.02249(9) A gamma = 90 deg.
Volume	1565.32(3) A^3
Z, Calculated density	4, 1.232 Mg/m^3
Absorption coefficient	0.693 mm^-1
F(000)	624
Crystal size	0.180 x 0.140 x 0.100 mm
Theta range for data collection	4.536 to 69.335 deg.
Limiting indices	-11<=h<=11, -19<=k<=18, -11<=l<=12
Reflections collected / unique	23789 / 2901 [R(int) = 0.0747]
Completeness to theta = 67.684	99.70%
Absorption correction	Semi-empirical from equivalents
Max. and min. transmission	1.00000 and 0.76509
Refinement method	Full-matrix least-squares on F^2
Data / restraints / parameters	2901 / 0 / 198
Goodness-of-fit on F^2	1.105
Final R indices [I>2sigma(I)]	R1 = 0.0505, wR2 = 0.1183
R indices (all data)	R1 = 0.0512, wR2 = 0.1191

Extinction coefficient	0.041(2)
Largest diff. peak and hole	0.283 and -0.352 e.A^-3

NMR Spectra

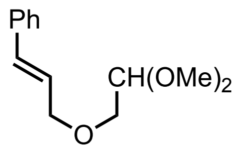

SM-a

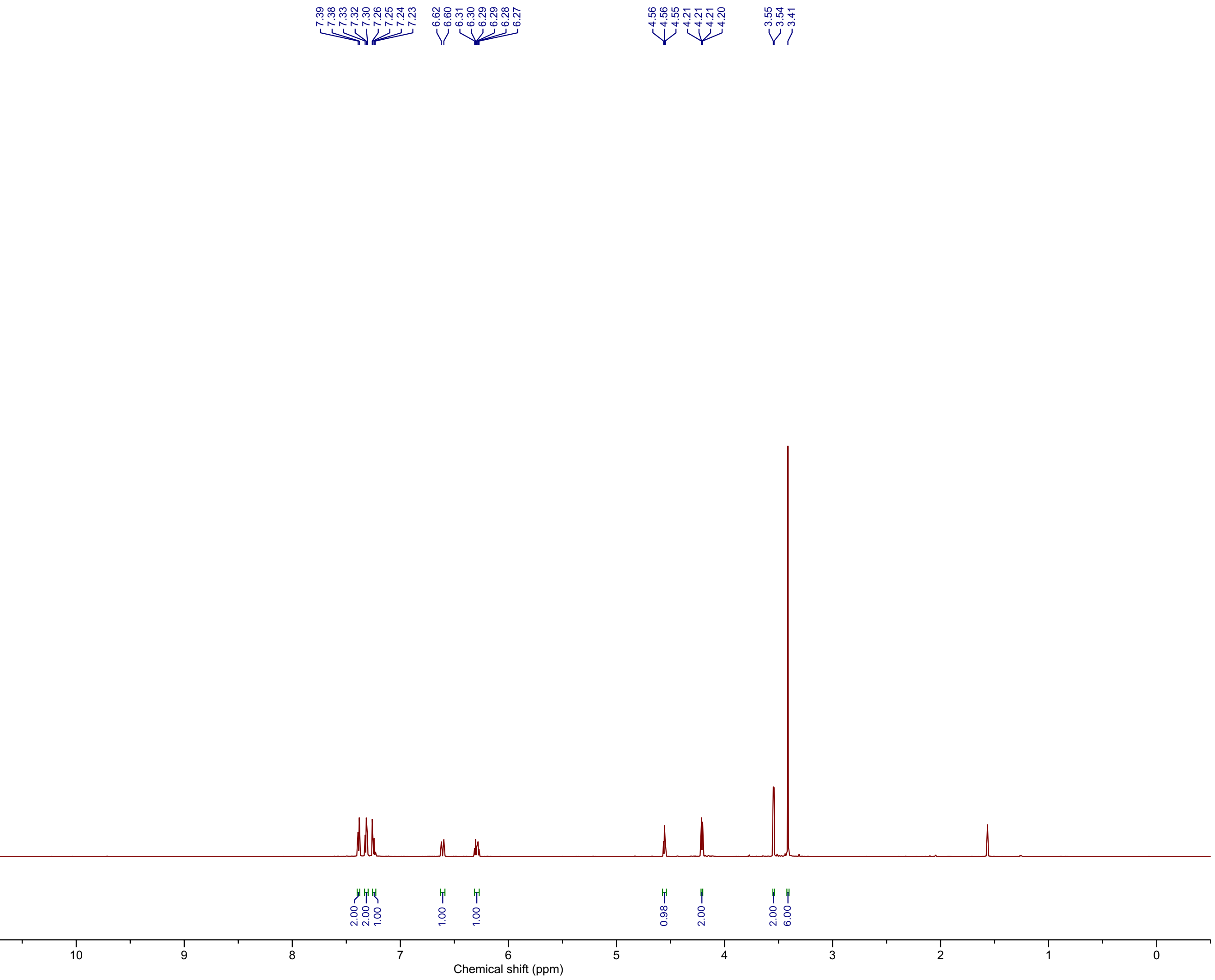

Supplementary Figure 8. ^{1}H NMR (700 MHz, $CDCl_3$) of **SM-a**.

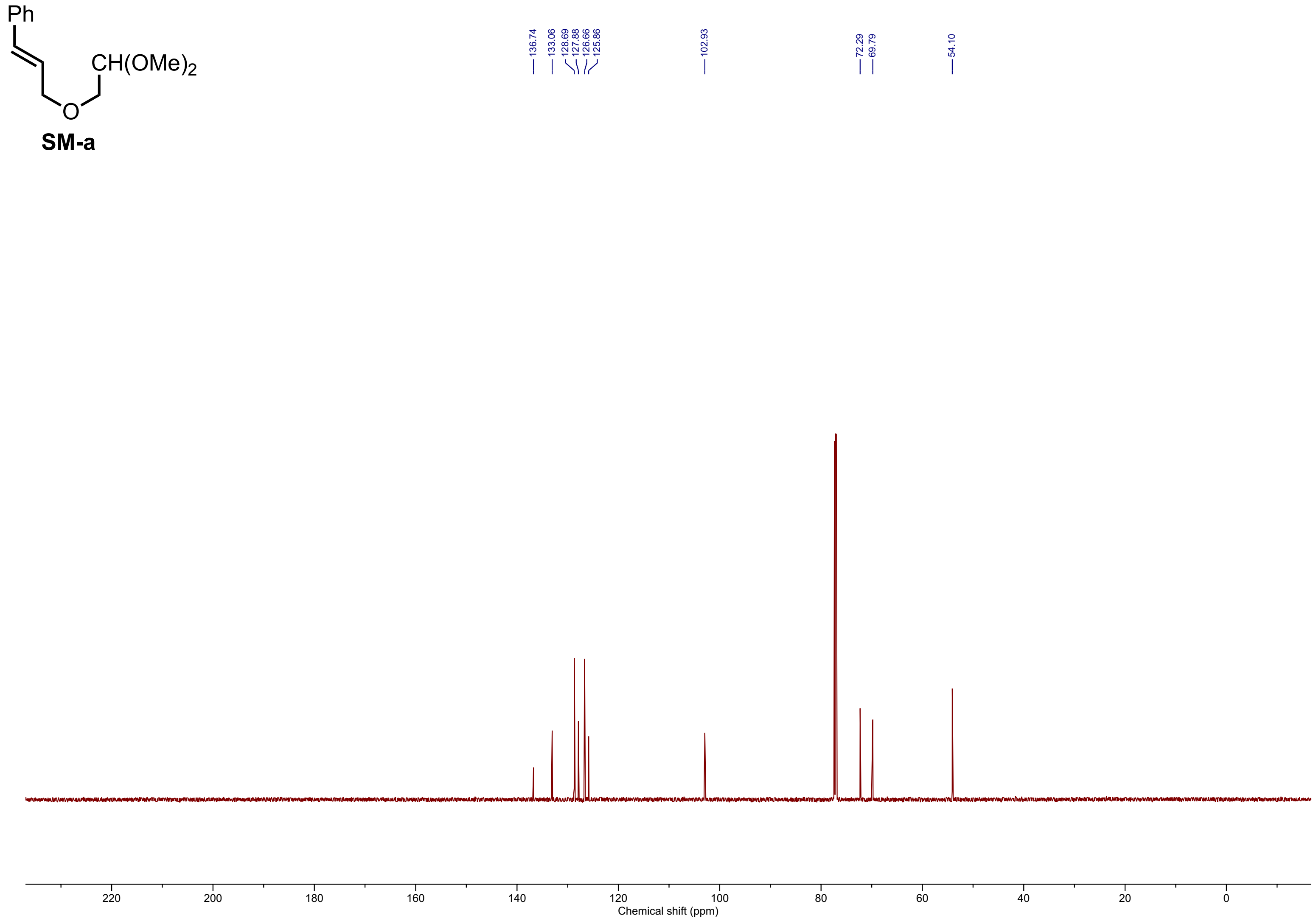

Supplementary Figure 9. ^{13}C NMR (176 MHz, $CDCl_3$) of **SM-a**.

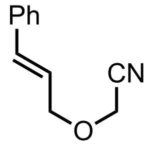

SM-b

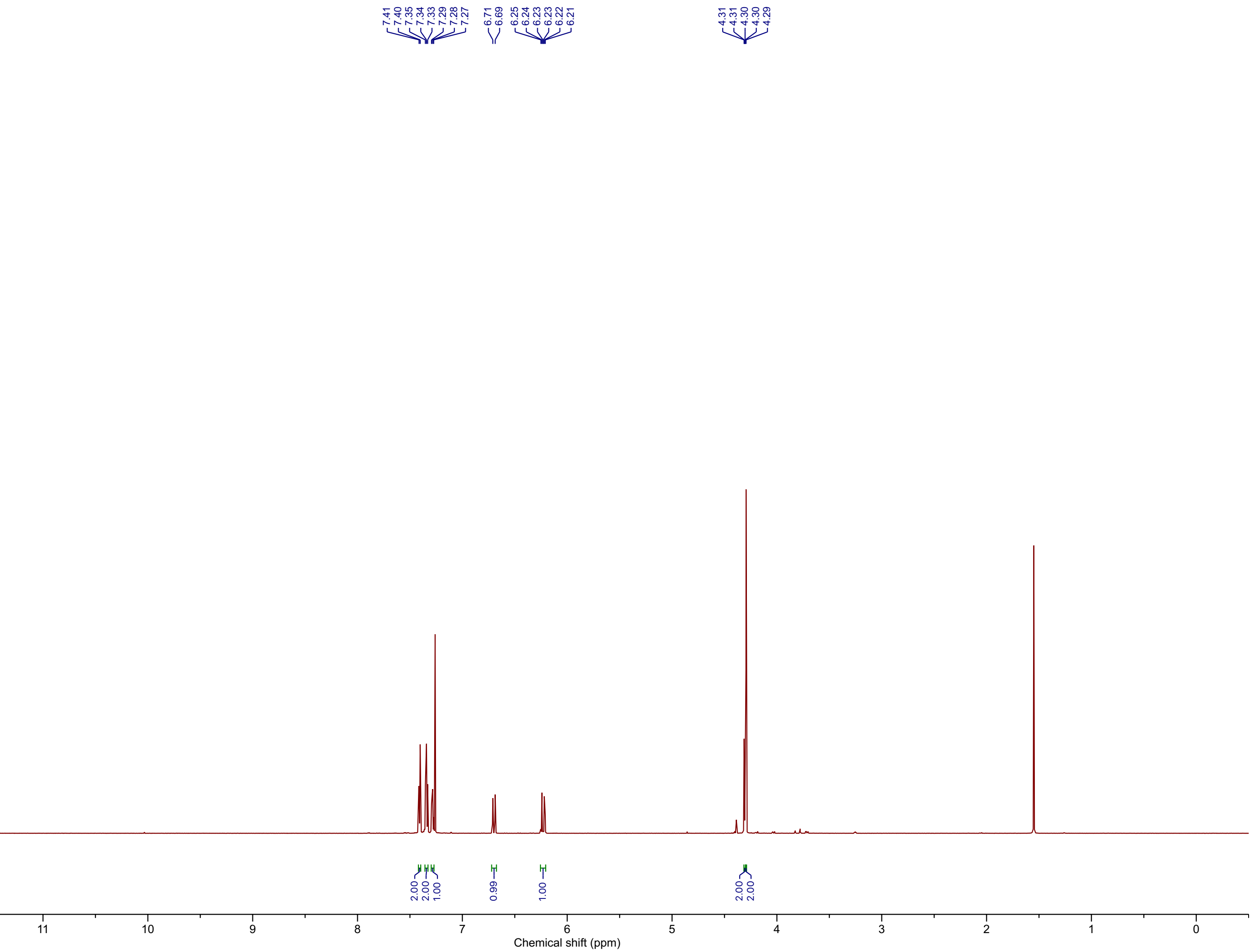

Supplementary Figure 10. ^{1}H NMR (700 MHz, $CDCl_3$) of **SM-b**.

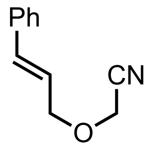

SM-b

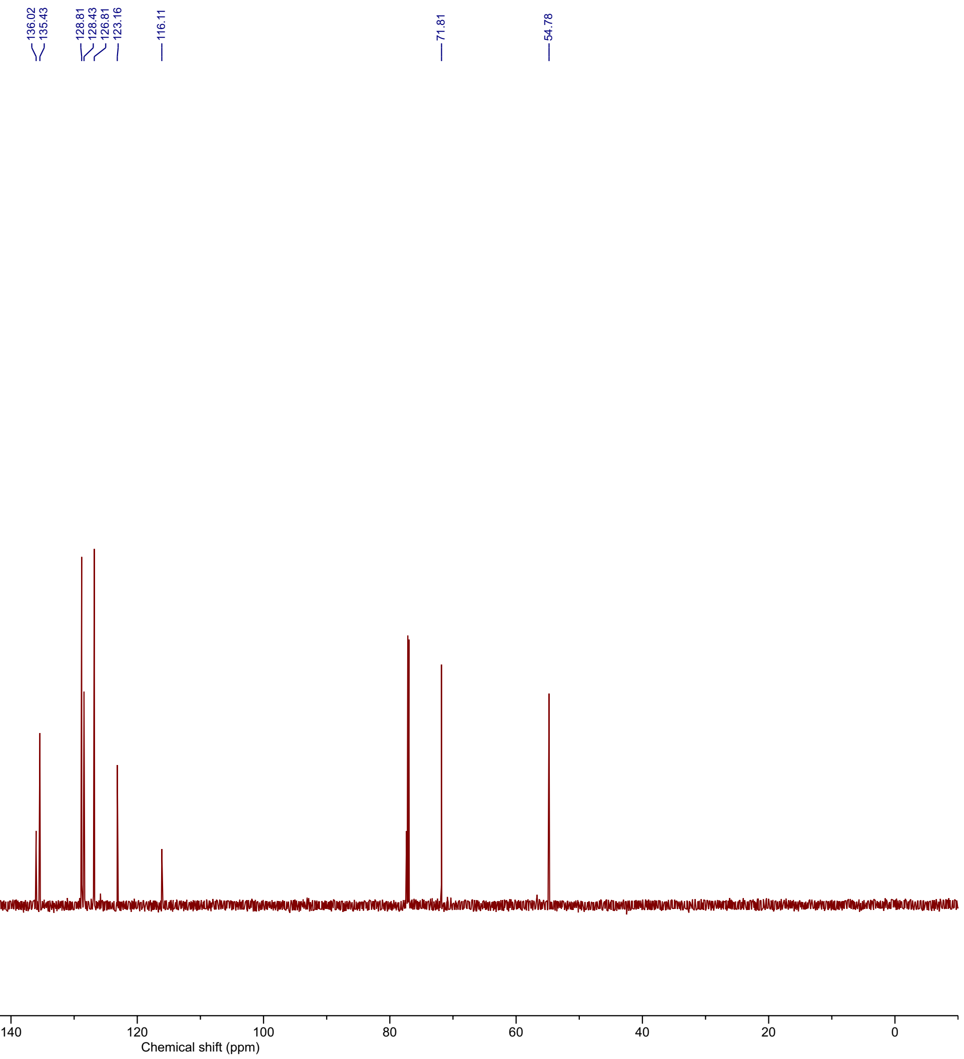

Supplementary Figure 11. ^{13}C NMR (176 MHz, $CDCl_3$) of **SM-b**.

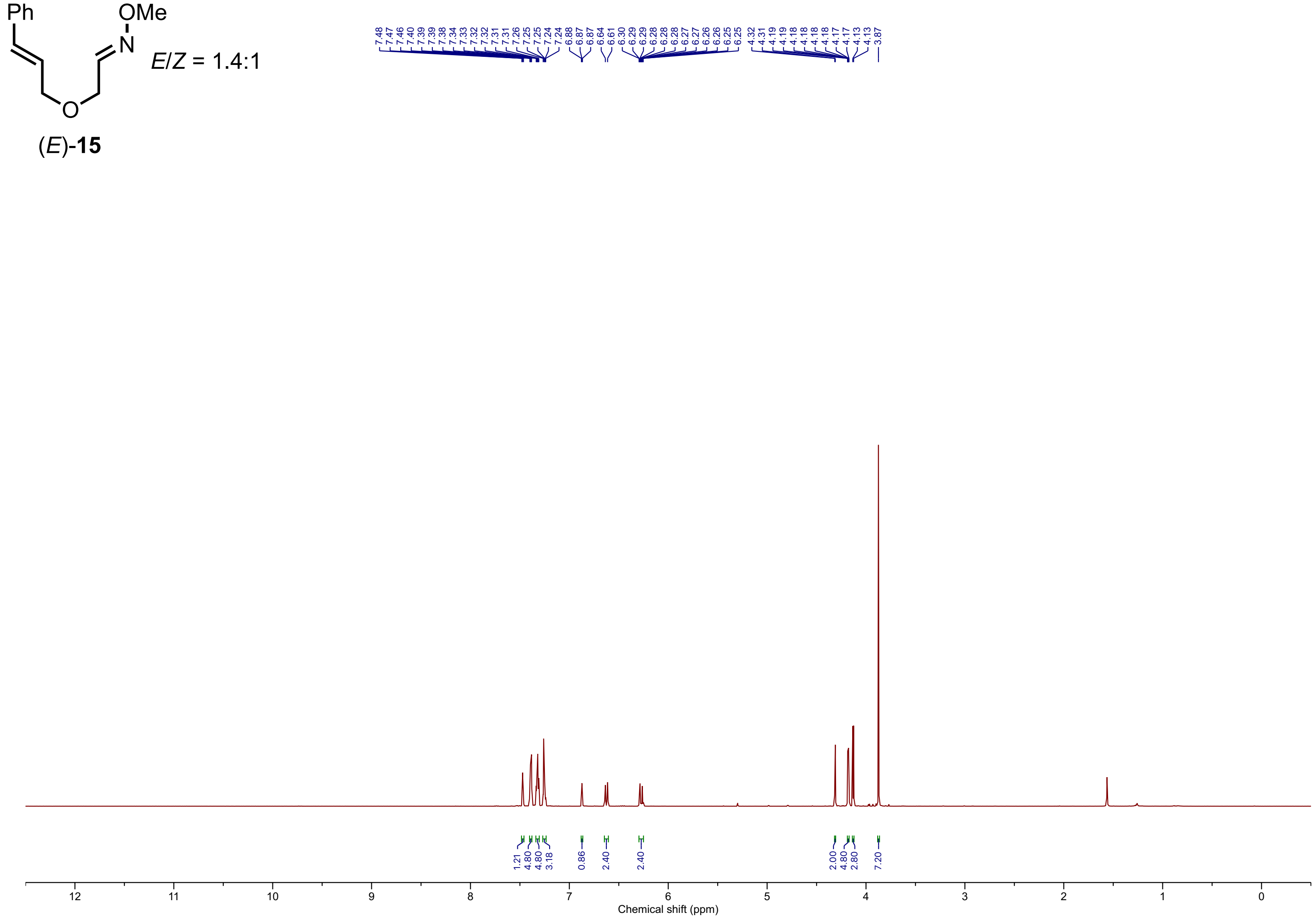

Supplementary Figure 12. ^{1}H NMR (700 MHz, $CDCl_3$) of (*E*)-**15**.

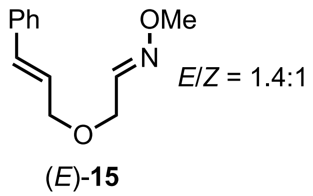

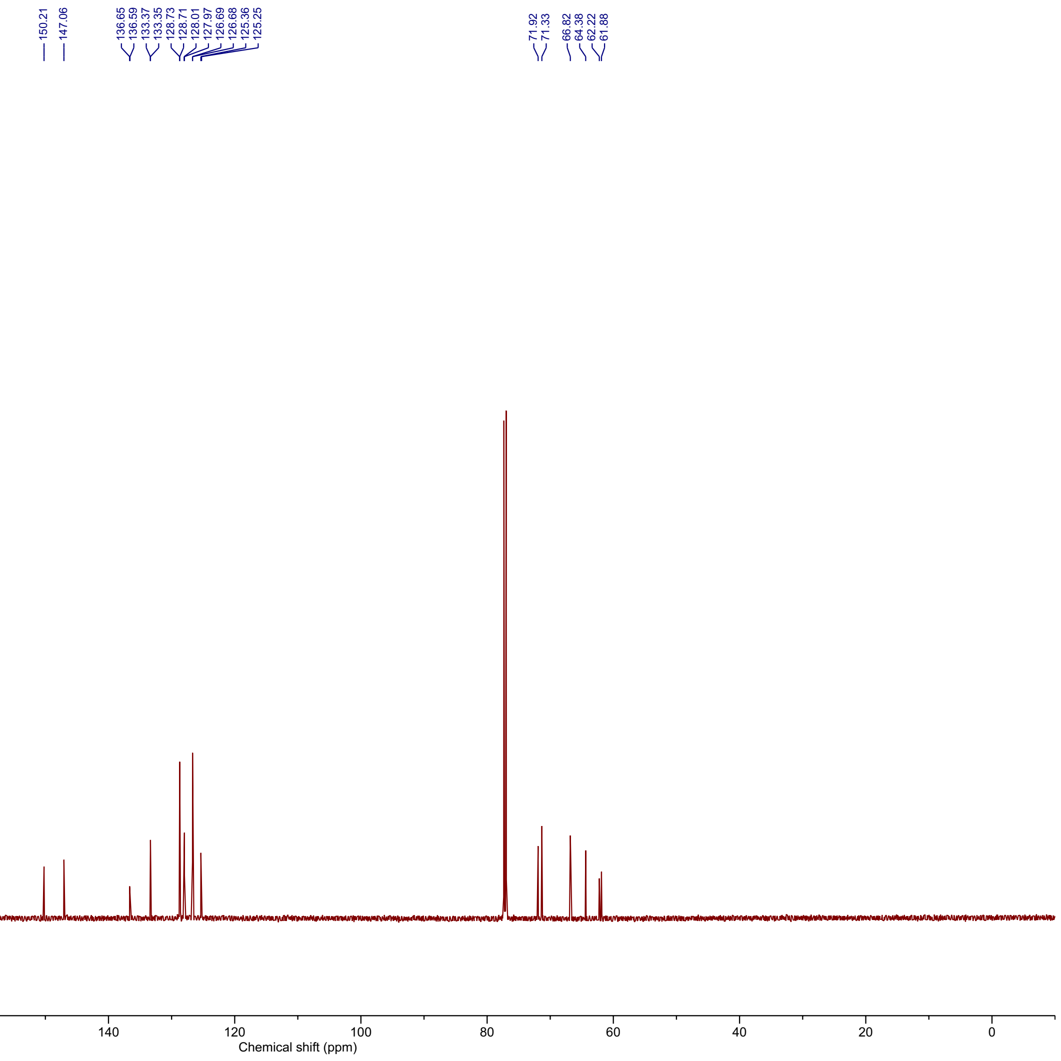

Supplementary Figure 13. ^{13}C NMR (176 MHz, $CDCl_3$) of (*E*)-**15**.

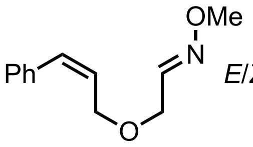

E/*Z* = 1.3:1

(*Z*)-**15**

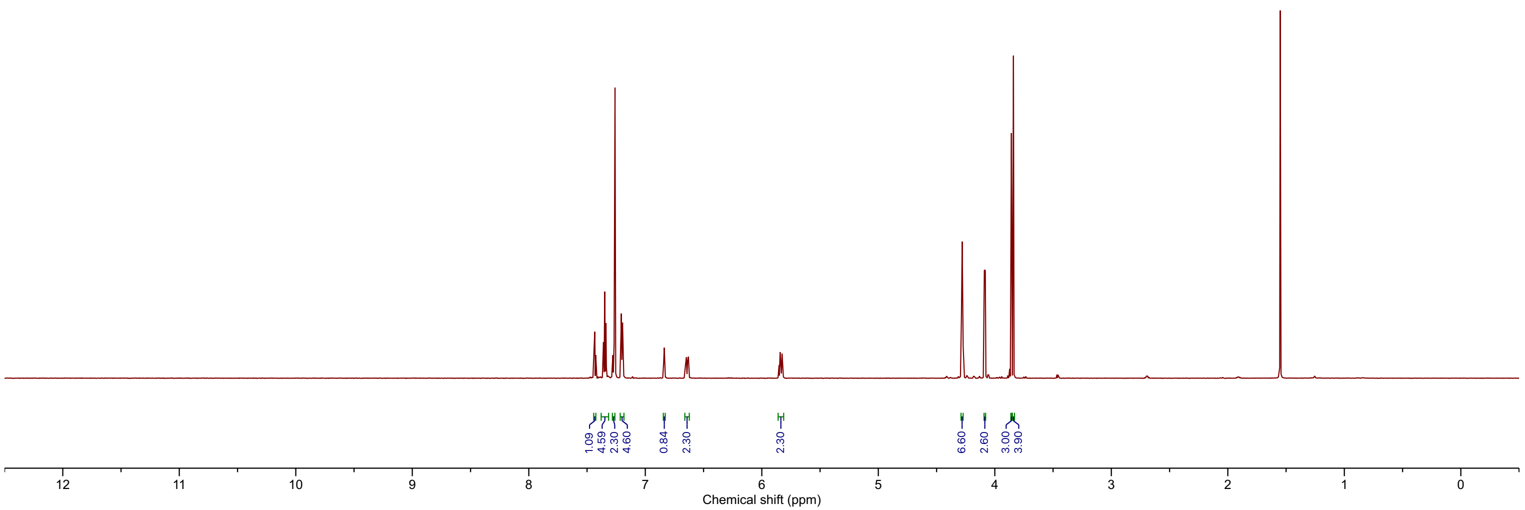

Supplementary Figure 14. ^{1}H NMR (700 MHz, $CDCl_3$) of (*Z*)-**15**.

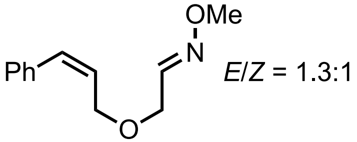

(*Z*)-**15**

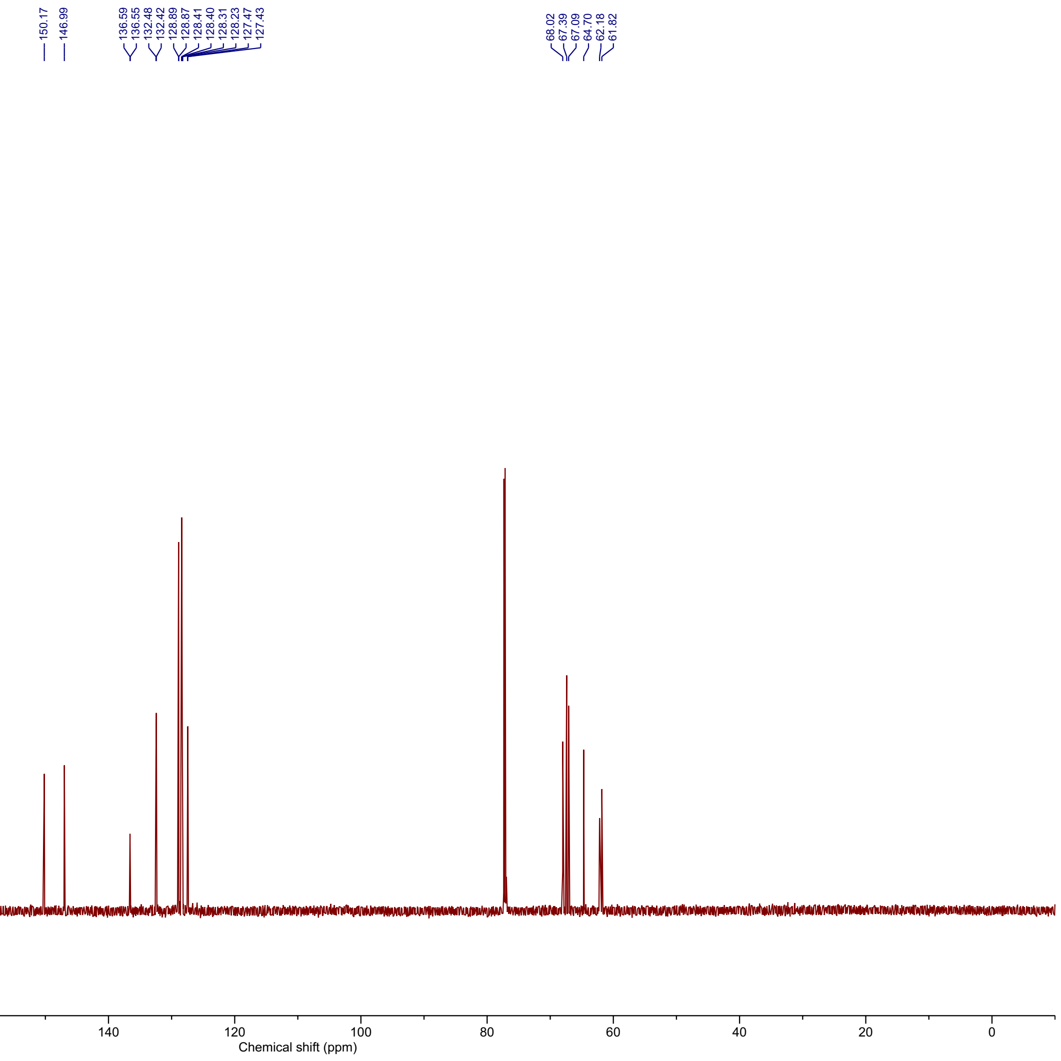

Supplementary Figure 15. ^{13}C NMR (176 MHz, $CDCl_3$) of (*Z*)-**15**.

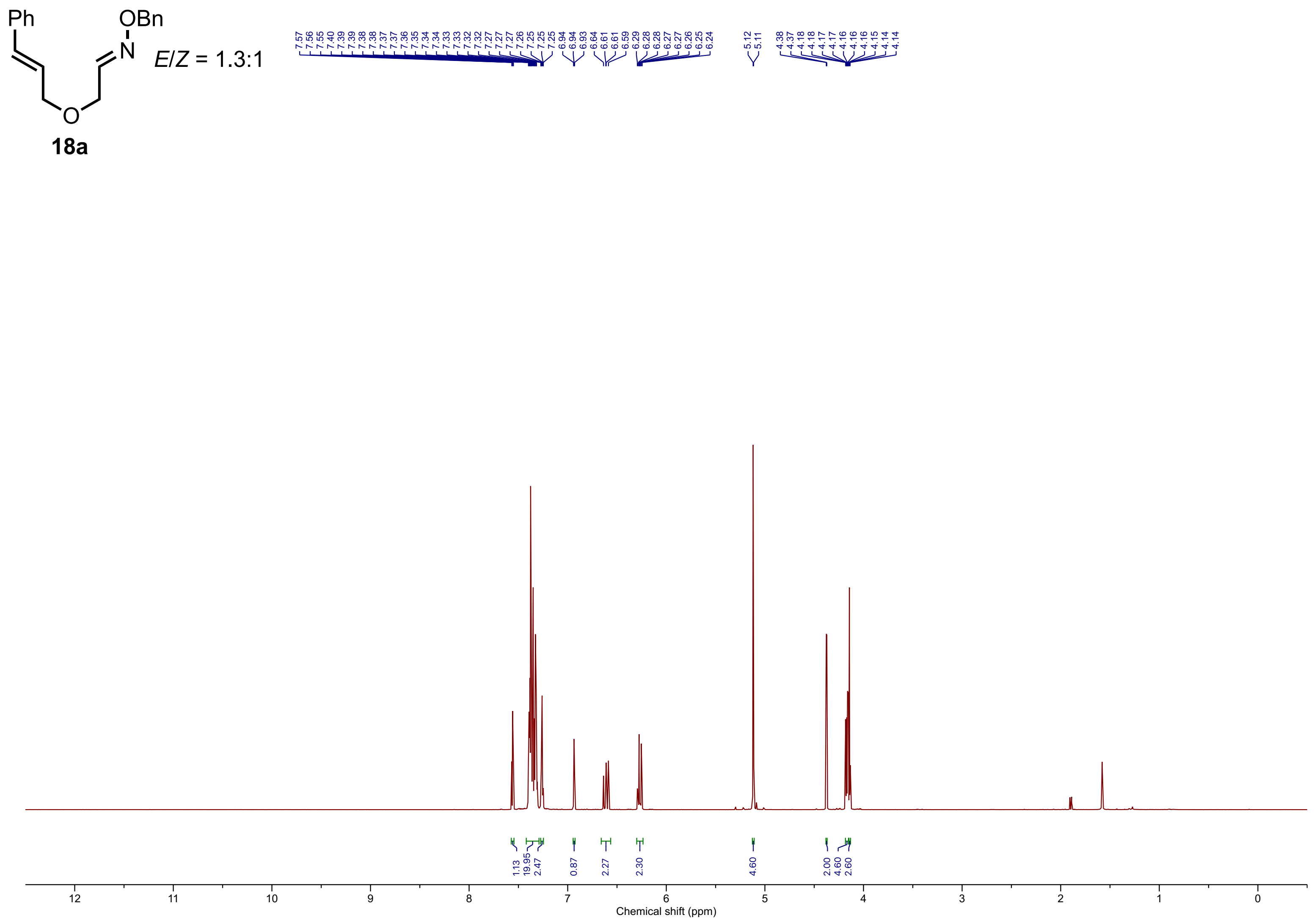

Supplementary Figure 16. ^{1}H NMR (700 MHz, $CDCl_3$) of **18a**.

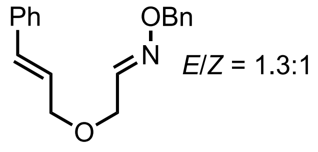

18a

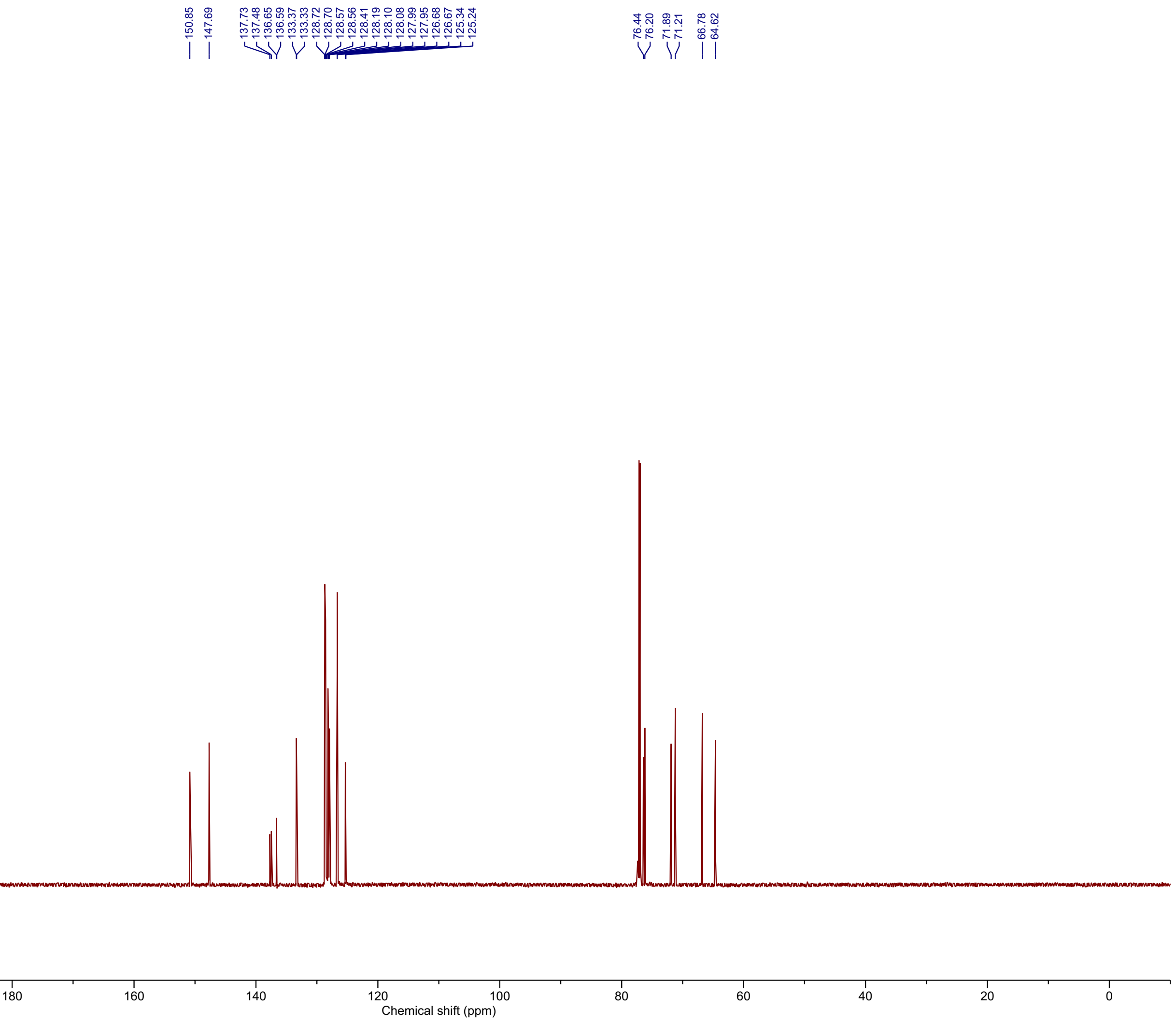

Supplementary Figure 17. ^{13}C NMR (176 MHz, $CDCl_3$) of **18a**.

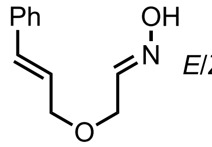

E/*Z* = 1.1:1

18b

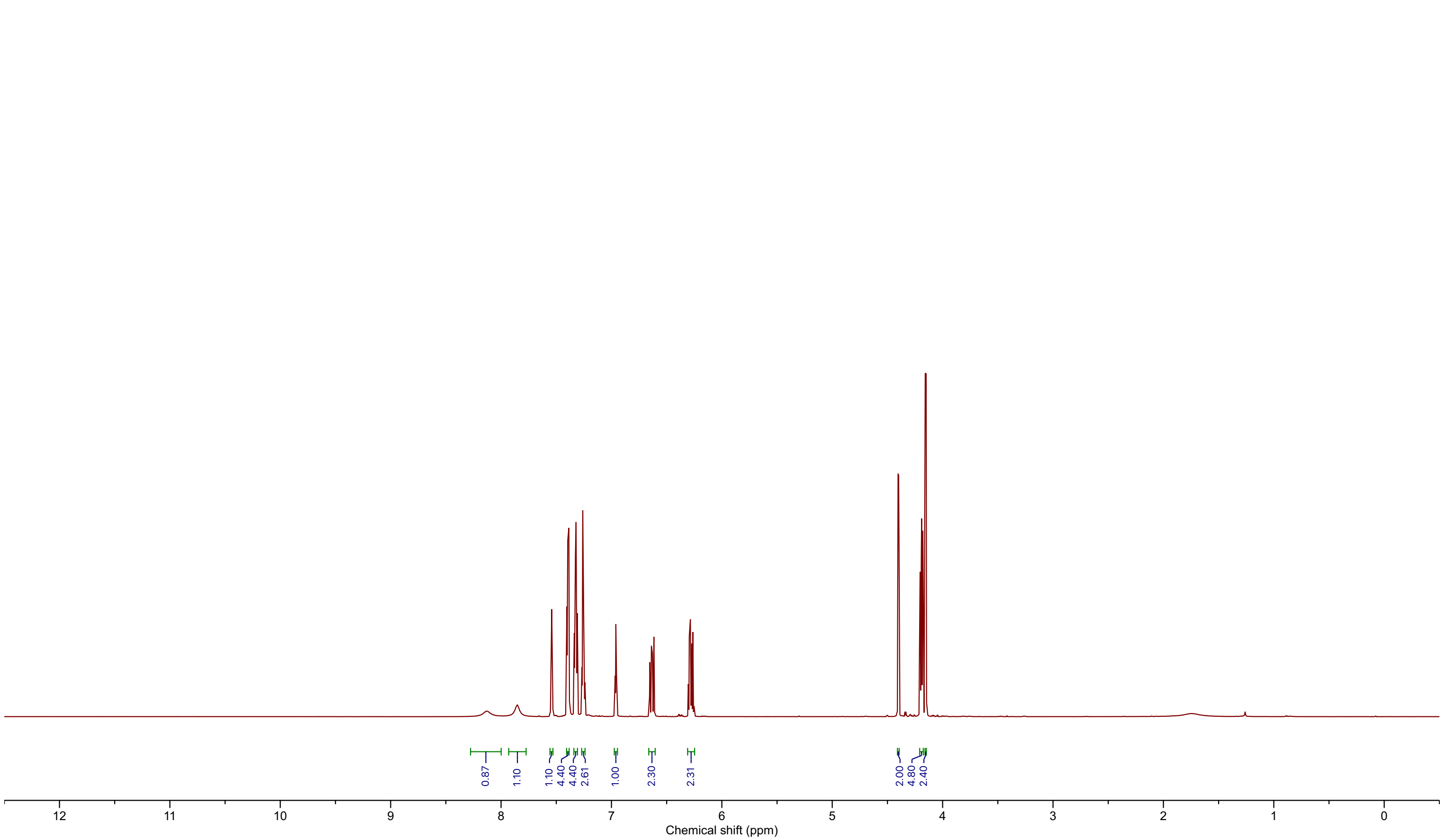

Supplementary Figure 18. ^{1}H NMR (700 MHz, $CDCl_3$) of **18b**.

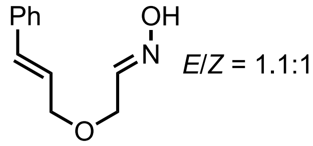

E/*Z* = 1.1:1

18b

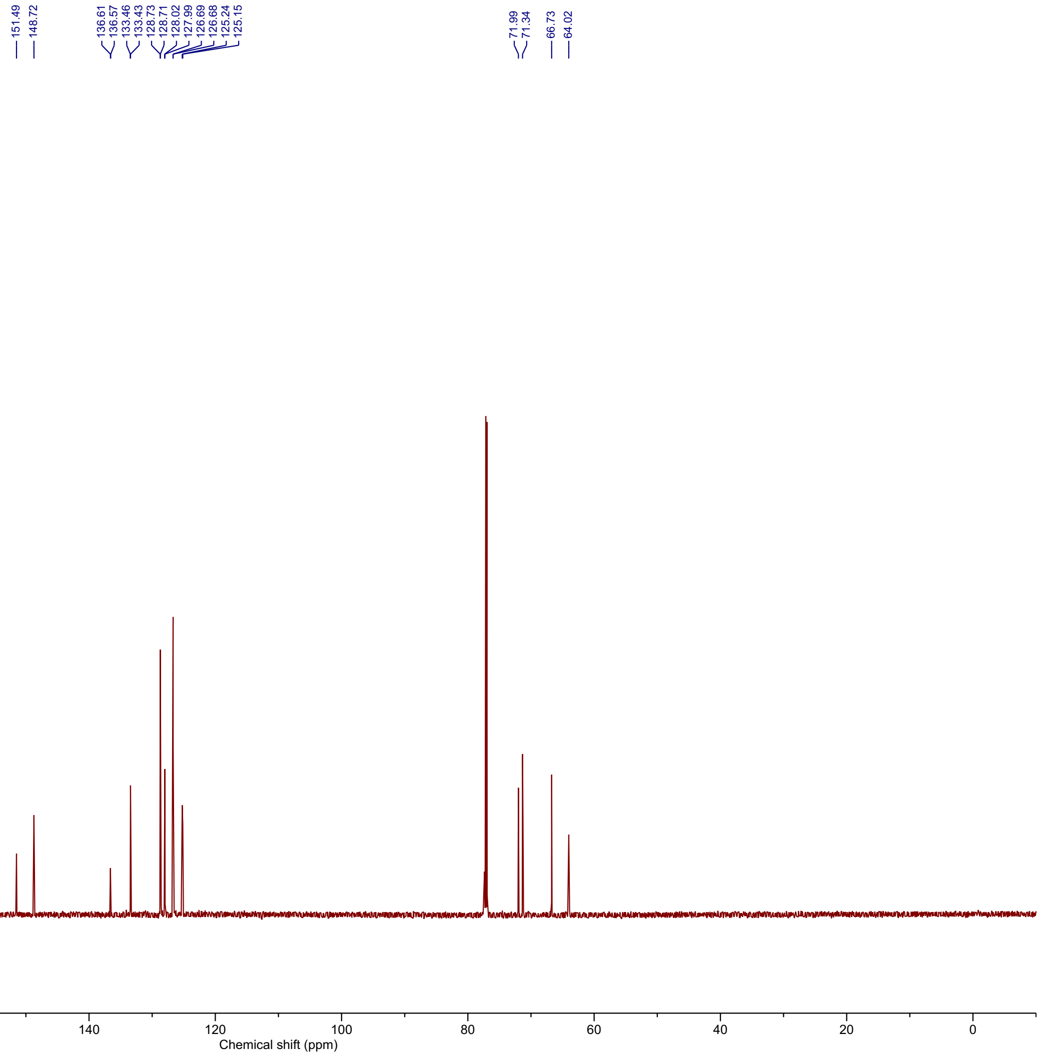

Supplementary Figure 19. ^{13}C NMR (176 MHz, $CDCl_3$) of **18b**.

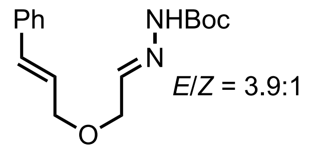

18c

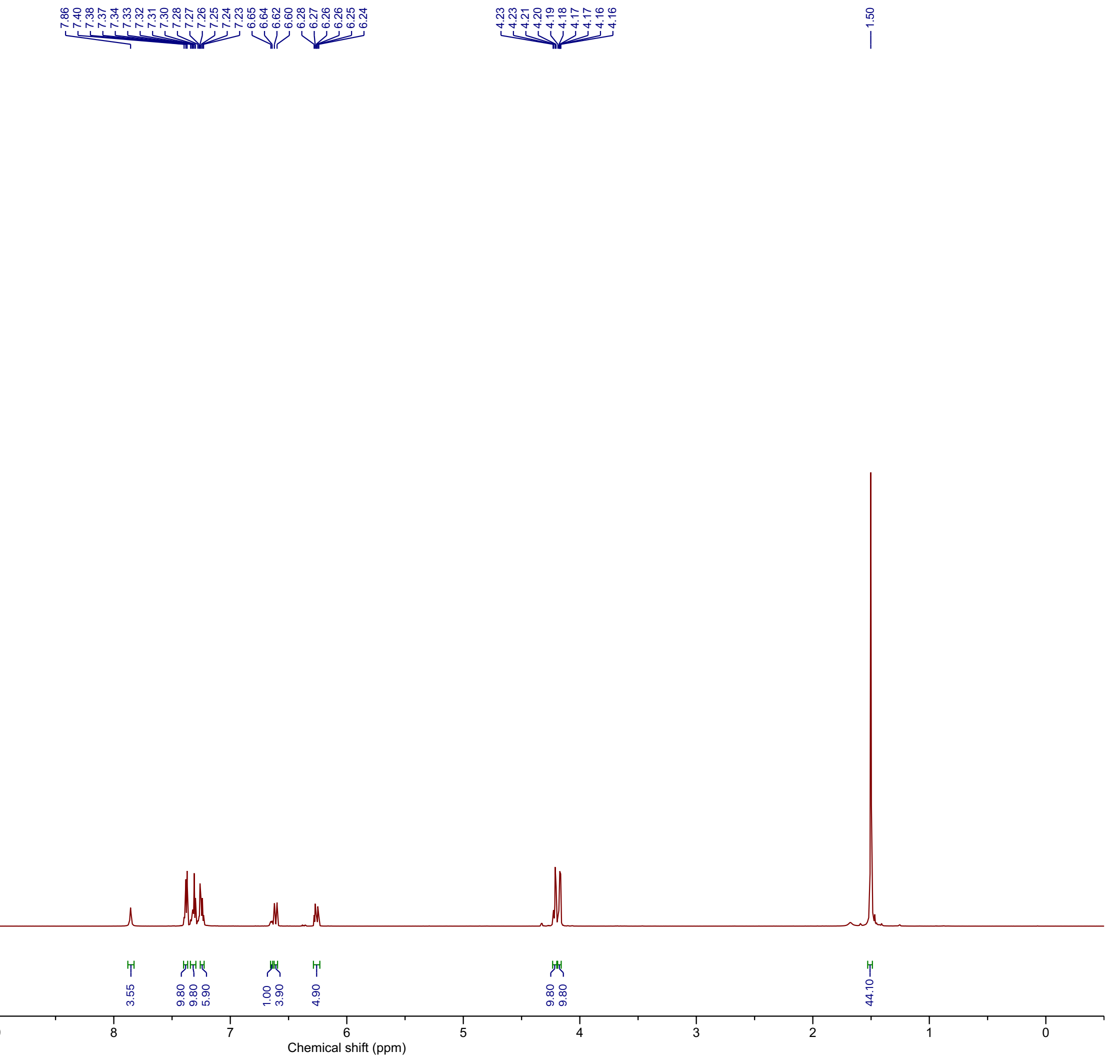

Supplementary Figure 20. ^{1}H NMR (700 MHz, $CDCl_3$) of **18c**.

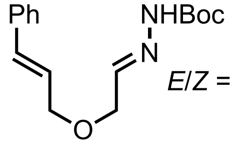

E/*Z* = 3.9:1

18c

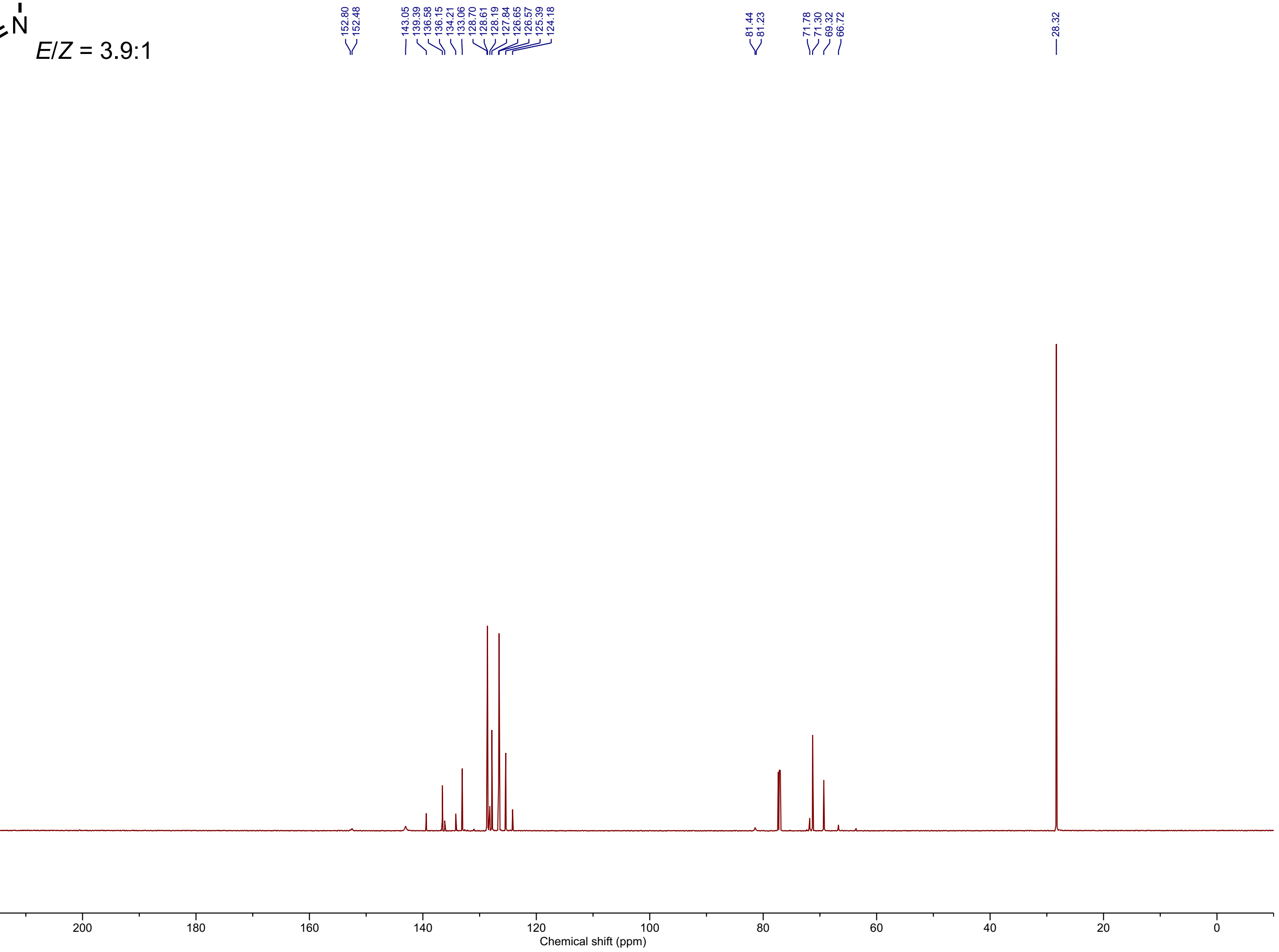

Supplementary Figure 21. ^{13}C NMR (176 MHz, $CDCl_3$) of **18c**.

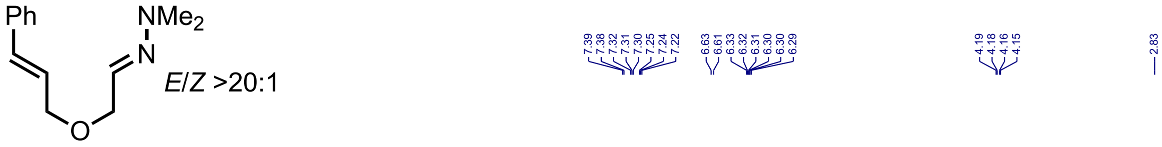

E/*Z* >20:1

18d

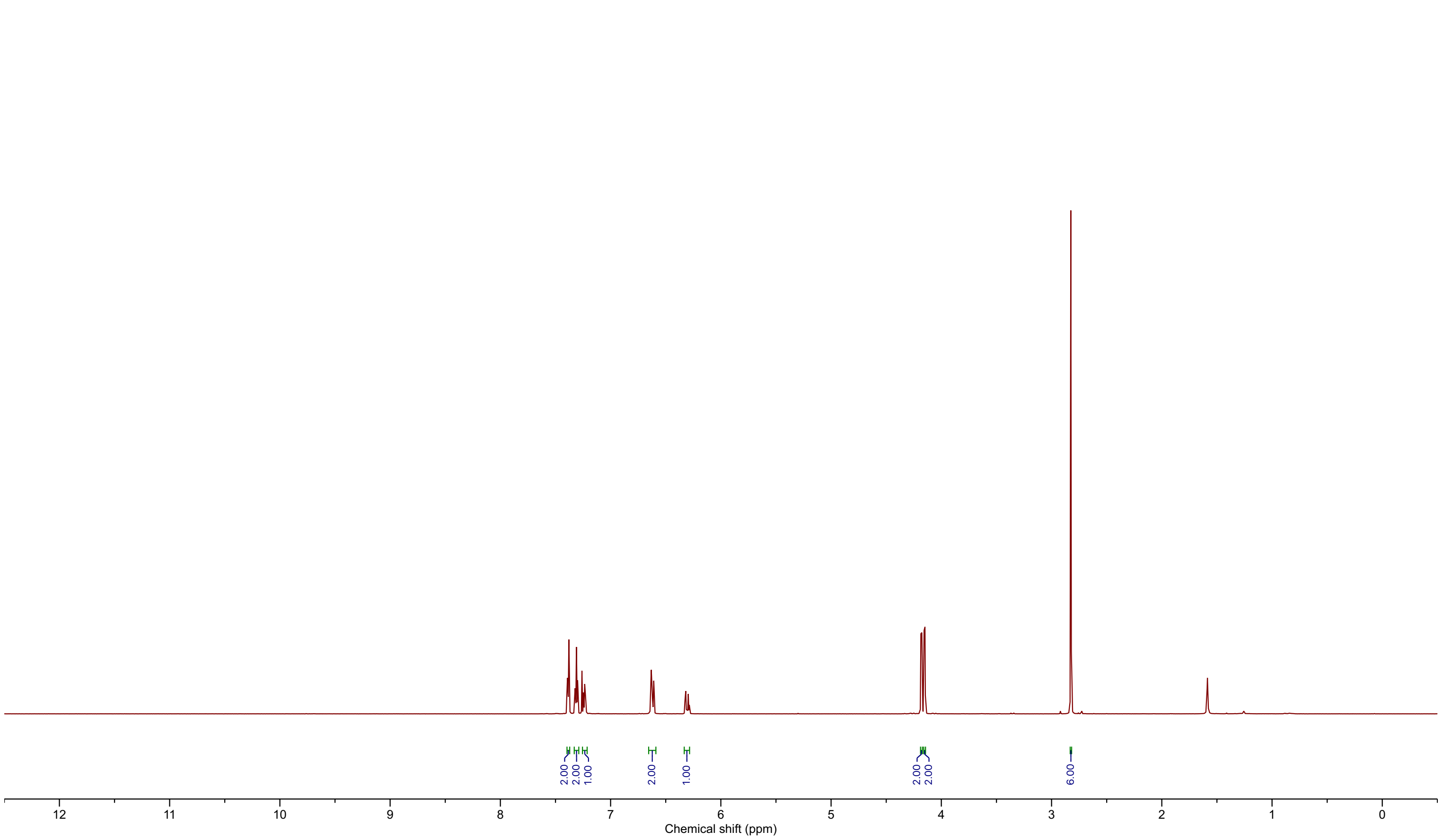

Supplementary Figure 22. ^{1}H NMR (700 MHz, $CDCl_3$) of **18d**.

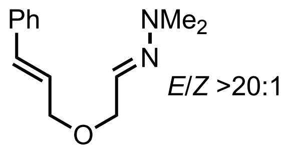

18d

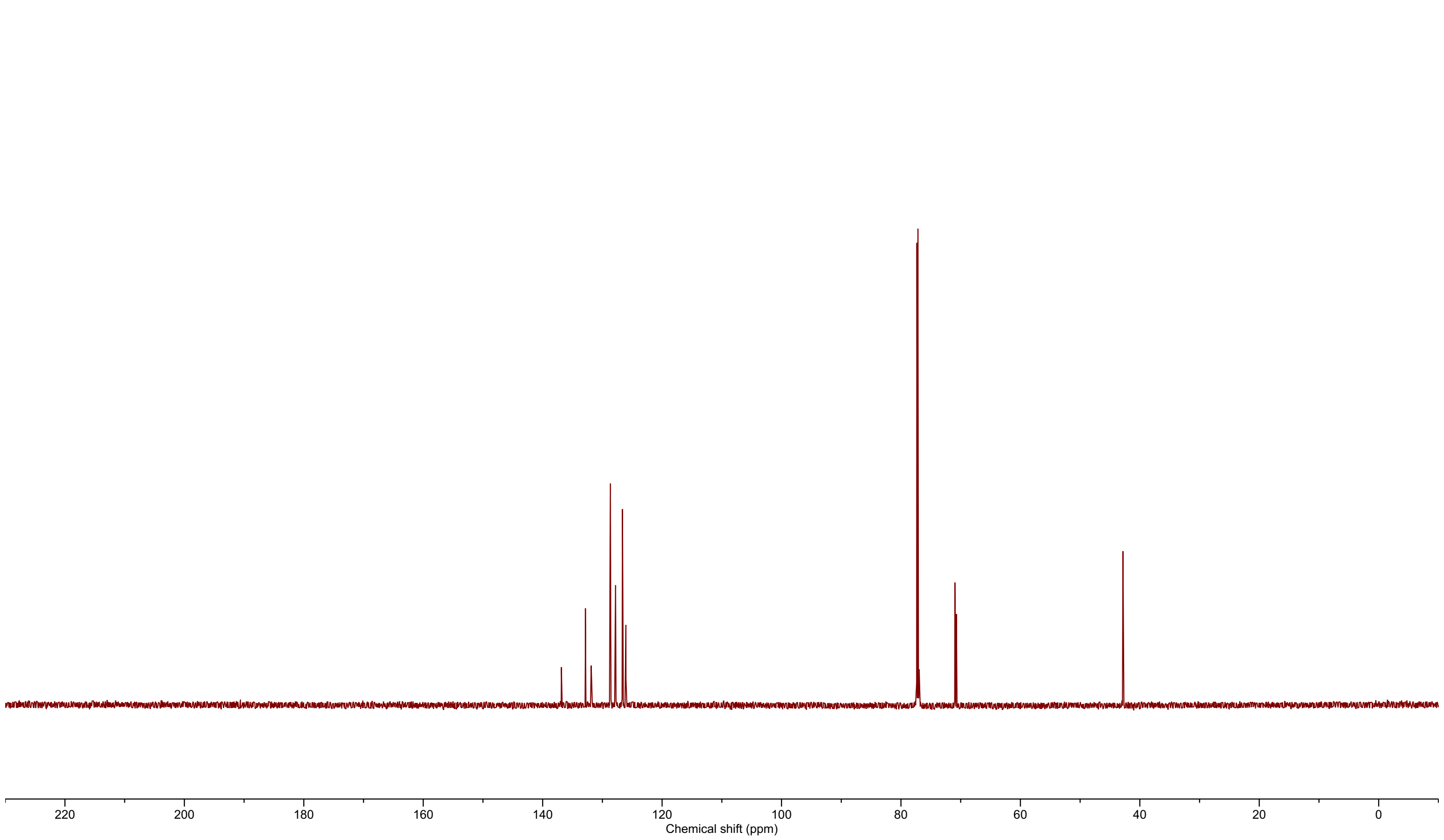

Supplementary Figure 23. ^{13}C NMR (176 MHz, $CDCl_3$) of **18d**.

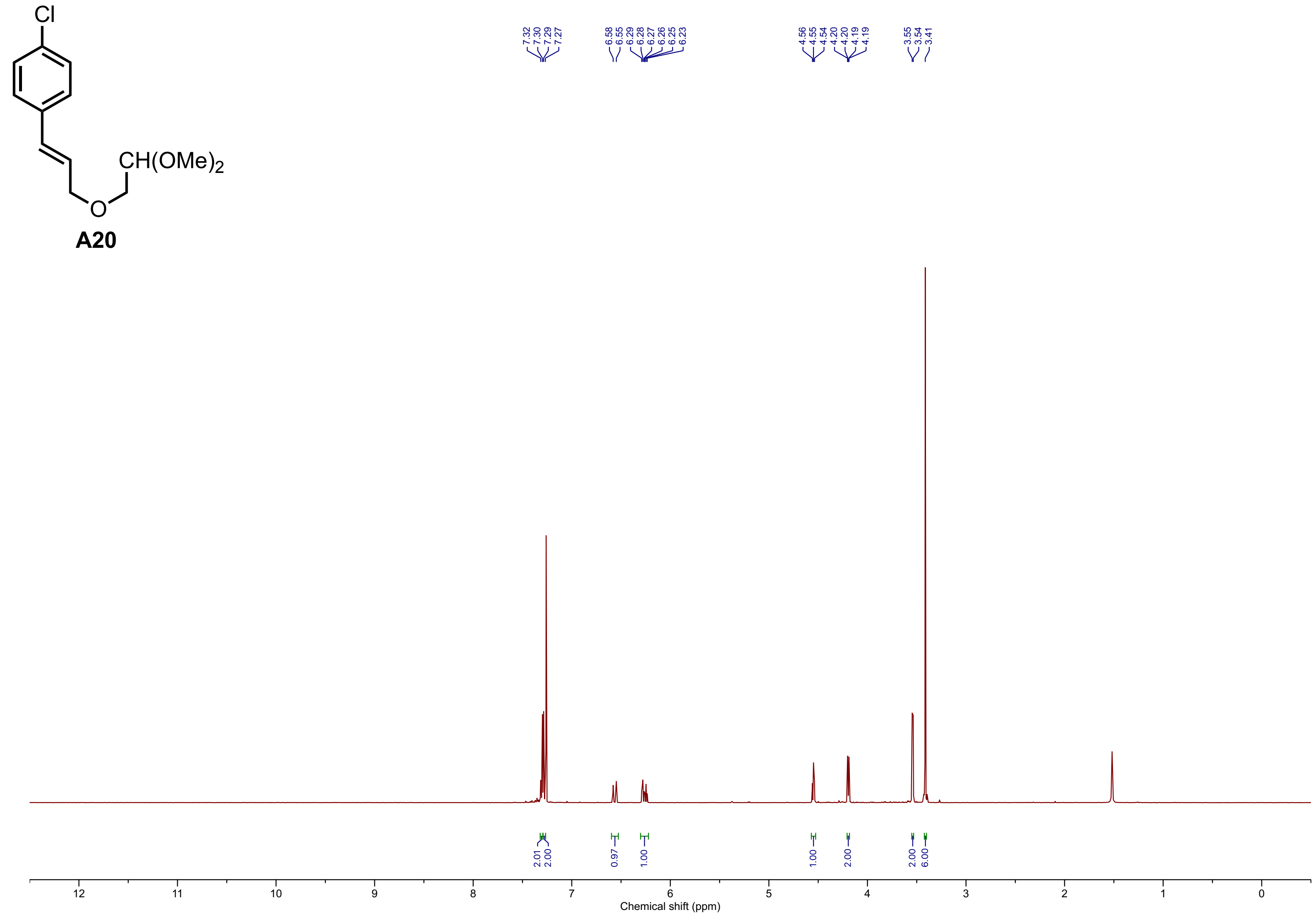

Supplementary Figure 24. ^{1}H NMR (500 MHz, $CDCl_3$) of **A20**.

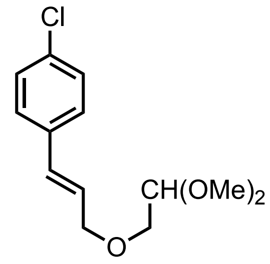

A20

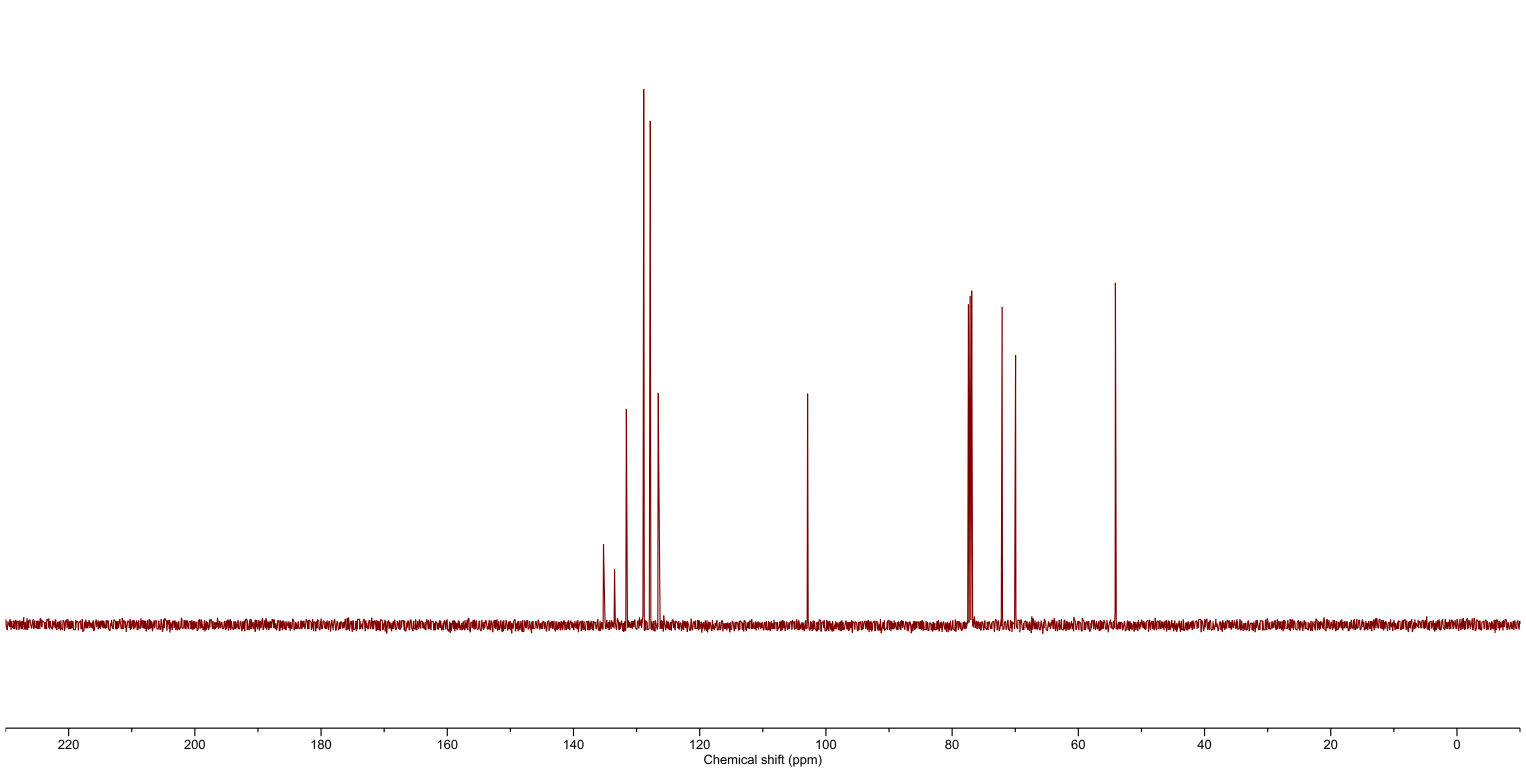

Supplementary Figure 25. ^{13}C NMR (126 MHz, $CDCl_3$) of **A20**.

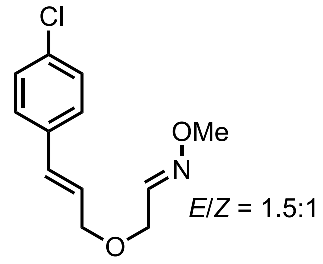

S20

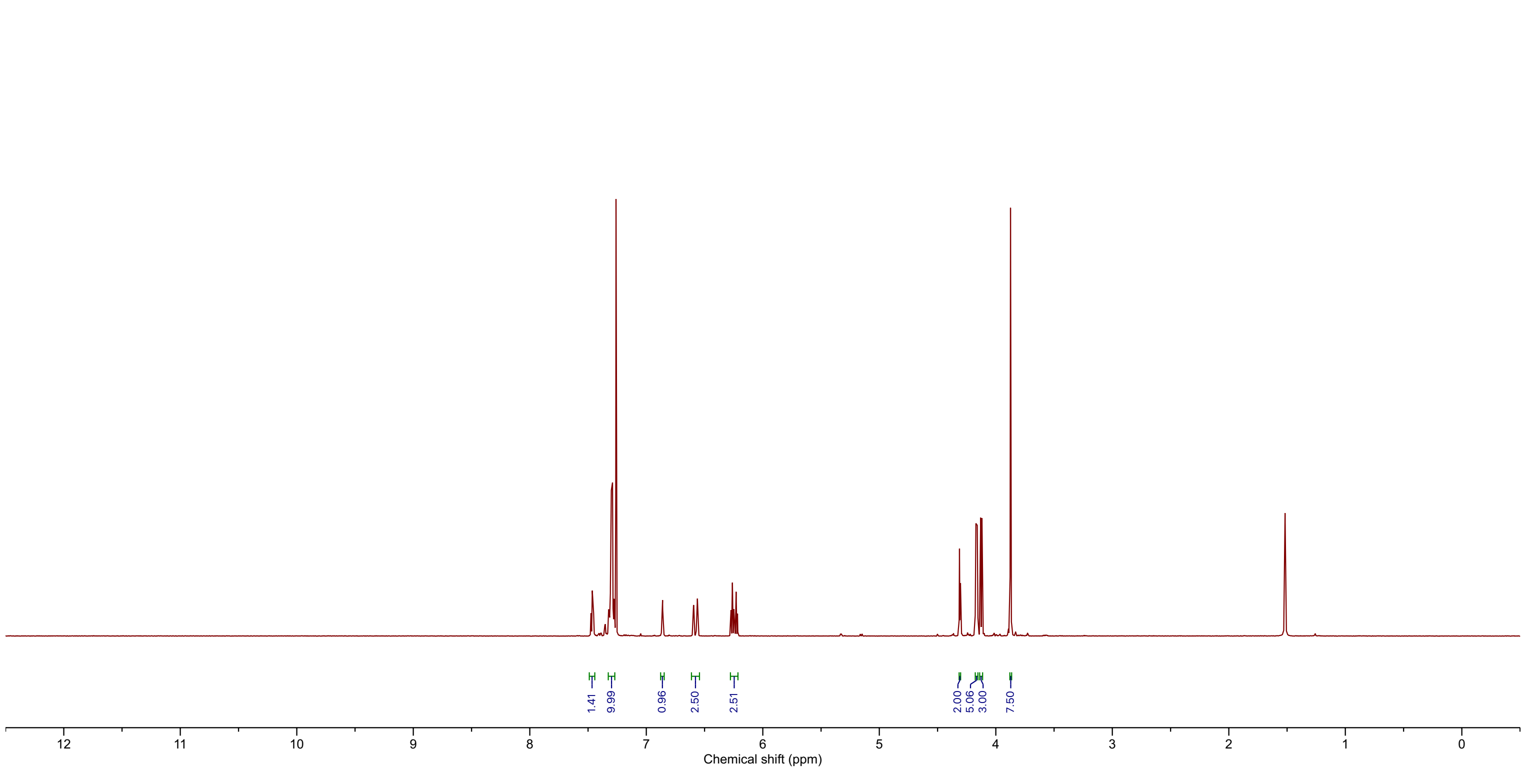

Supplementary Figure 26. ^{1}H NMR (500 MHz, $CDCl_3$) of **S20**.

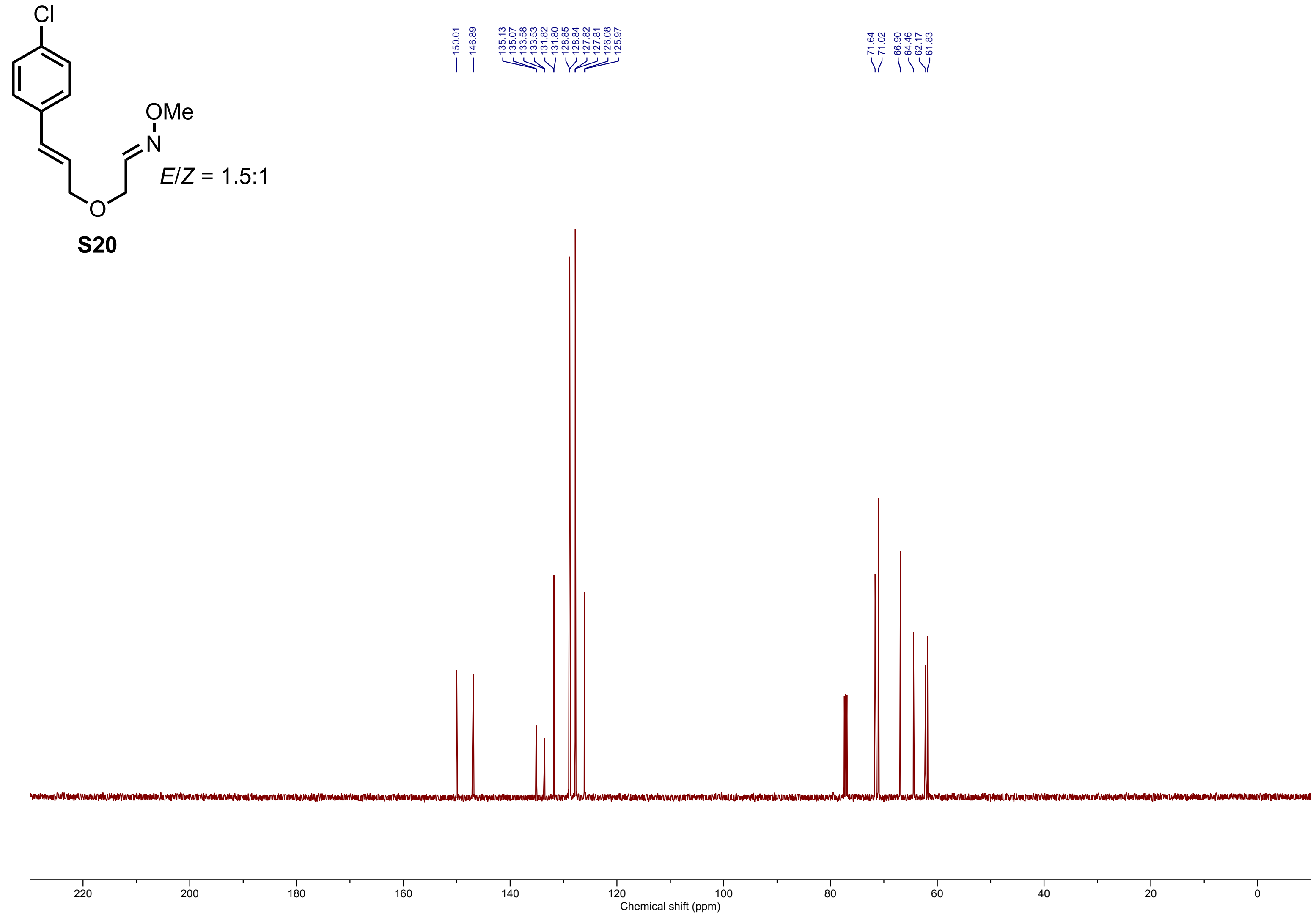

Supplementary Figure 27. ^{13}C NMR (500 MHz, $CDCl_3$) of **S20**.

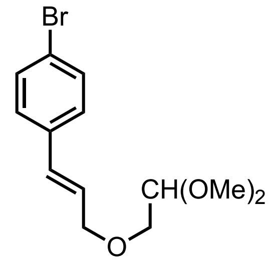

A21

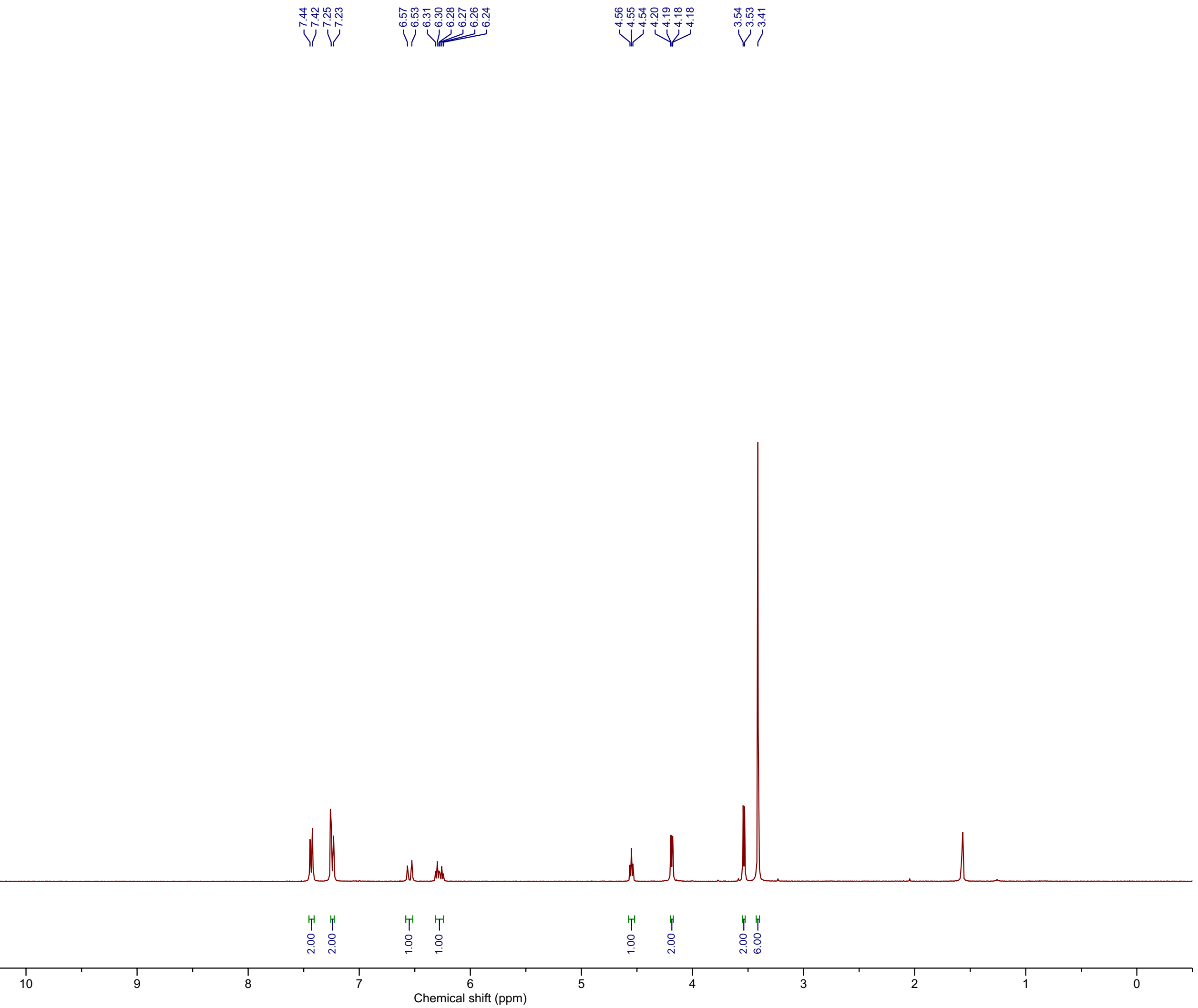

Supplementary Figure 28. ^{1}H NMR (400 MHz, $CDCl_3$) of **A21**.

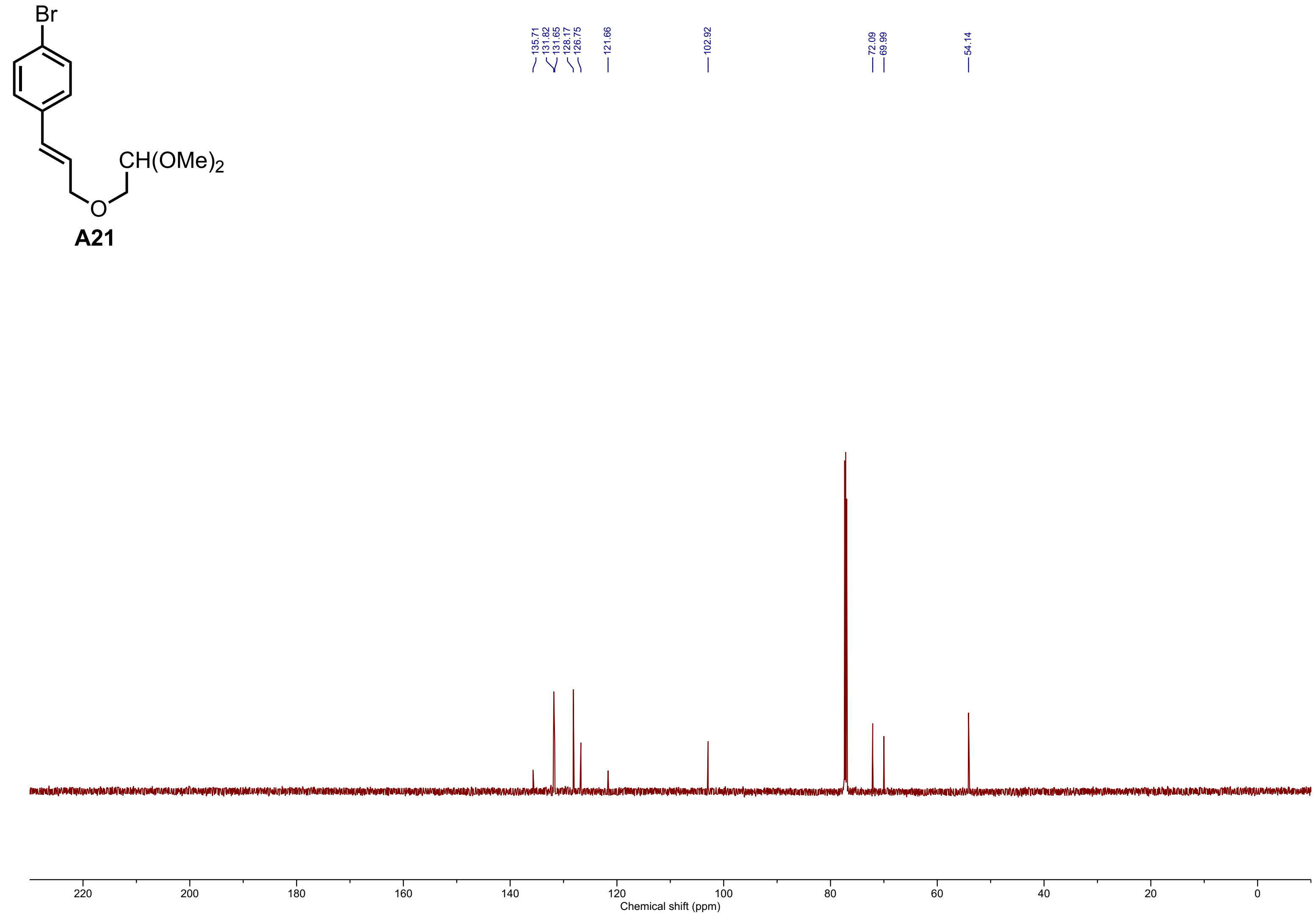

Supplementary Figure 29. ^{13}C NMR (176 MHz, $CDCl_3$) of **A21**.

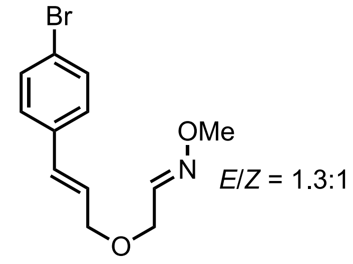

S21

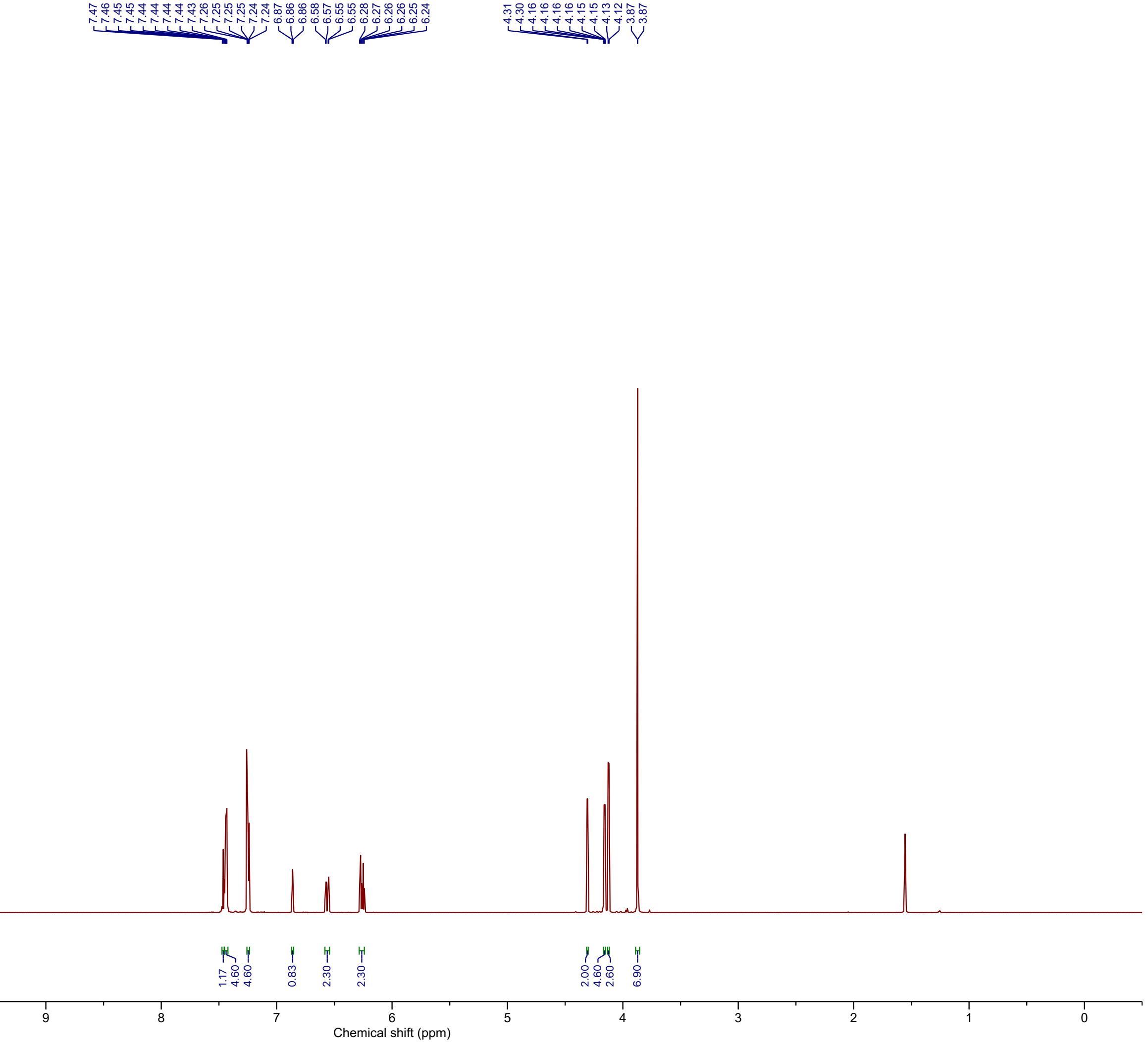

Supplementary Figure 30. ^{1}H NMR (700 MHz, $CDCl_3$) of **S21**.

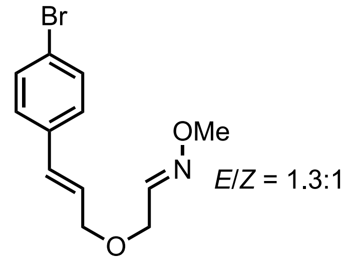

S21

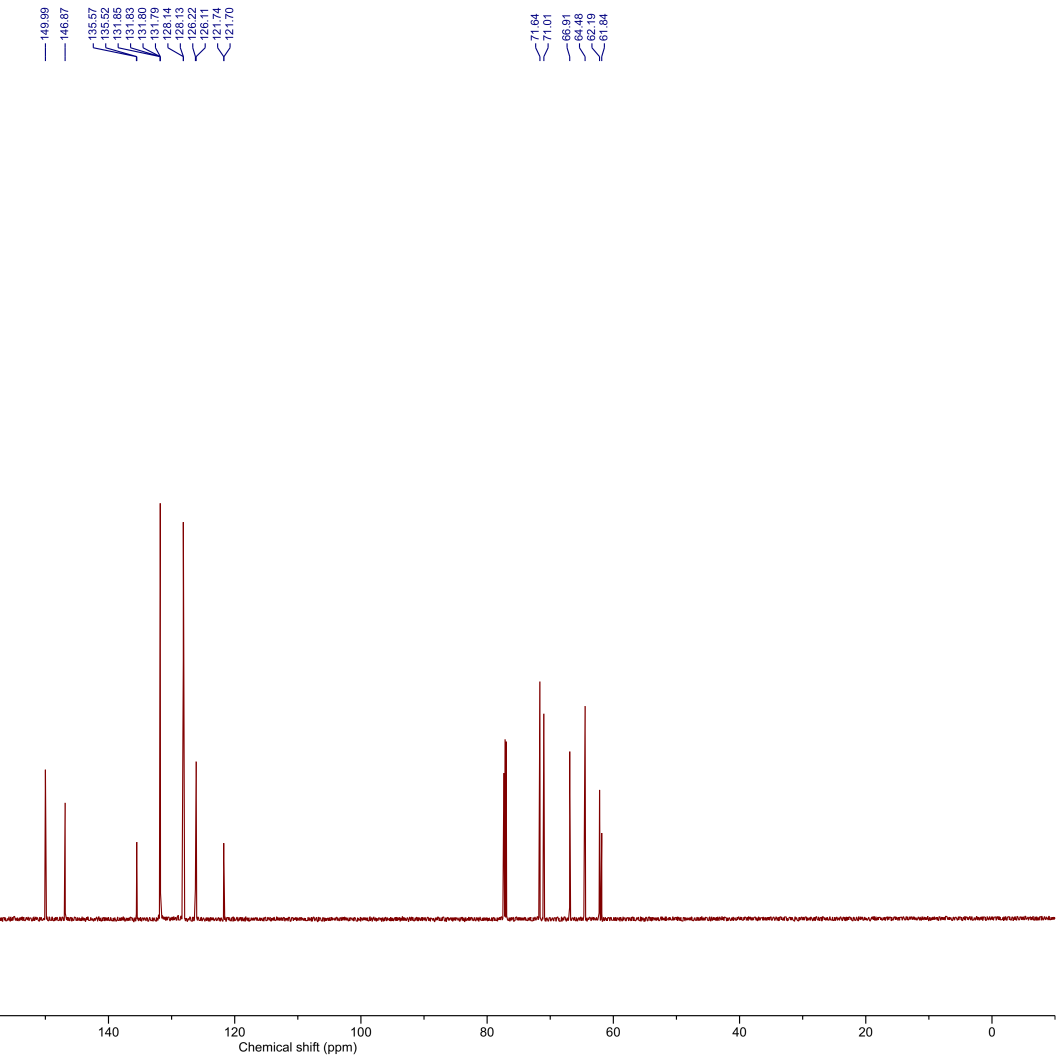

Supplementary Figure 31. ^{13}C NMR (176 MHz, $CDCl_3$) of **S21**.

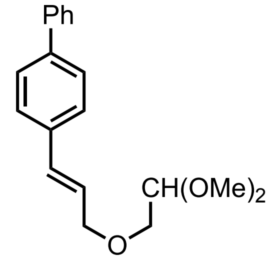

A22

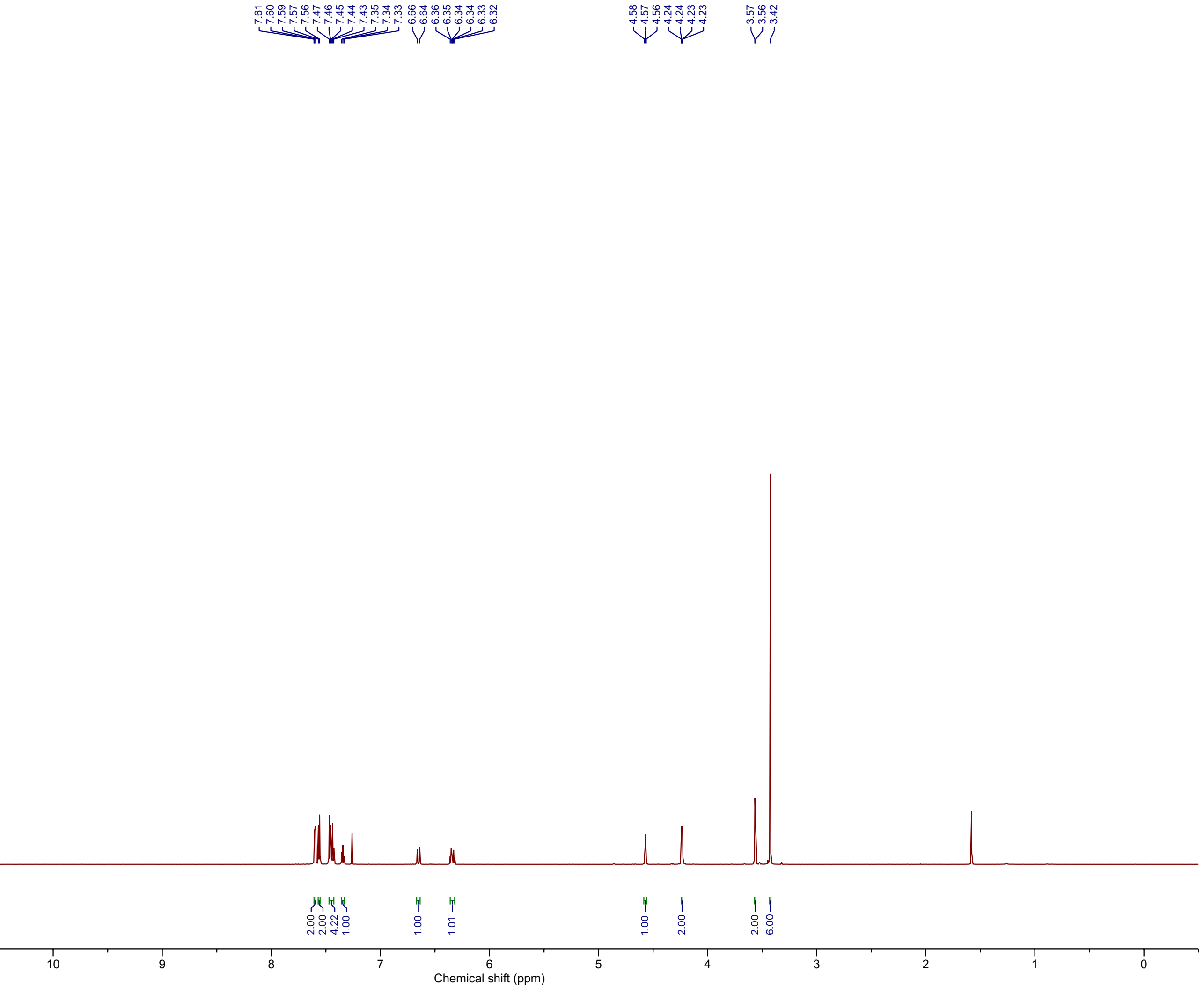

Supplementary Figure 32. ^{1}H NMR (700 MHz, $CDCl_3$) of **A22**.

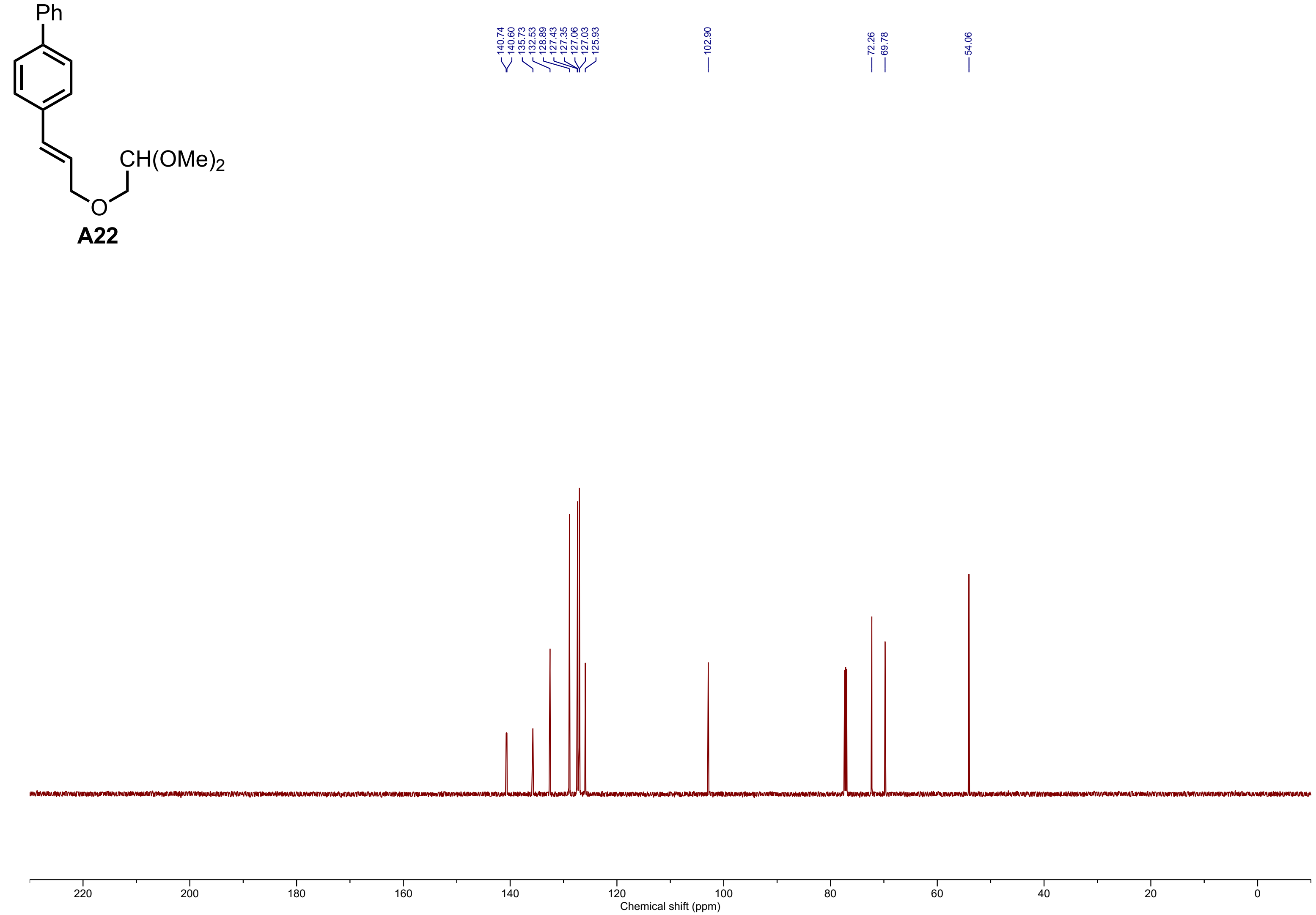

Supplementary Figure 33. ^{13}C NMR (176 MHz, $CDCl_3$) of **A22**.

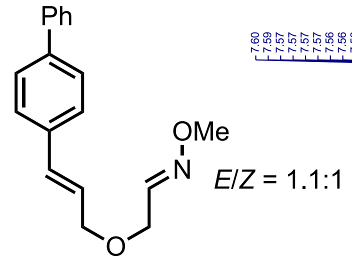

S22

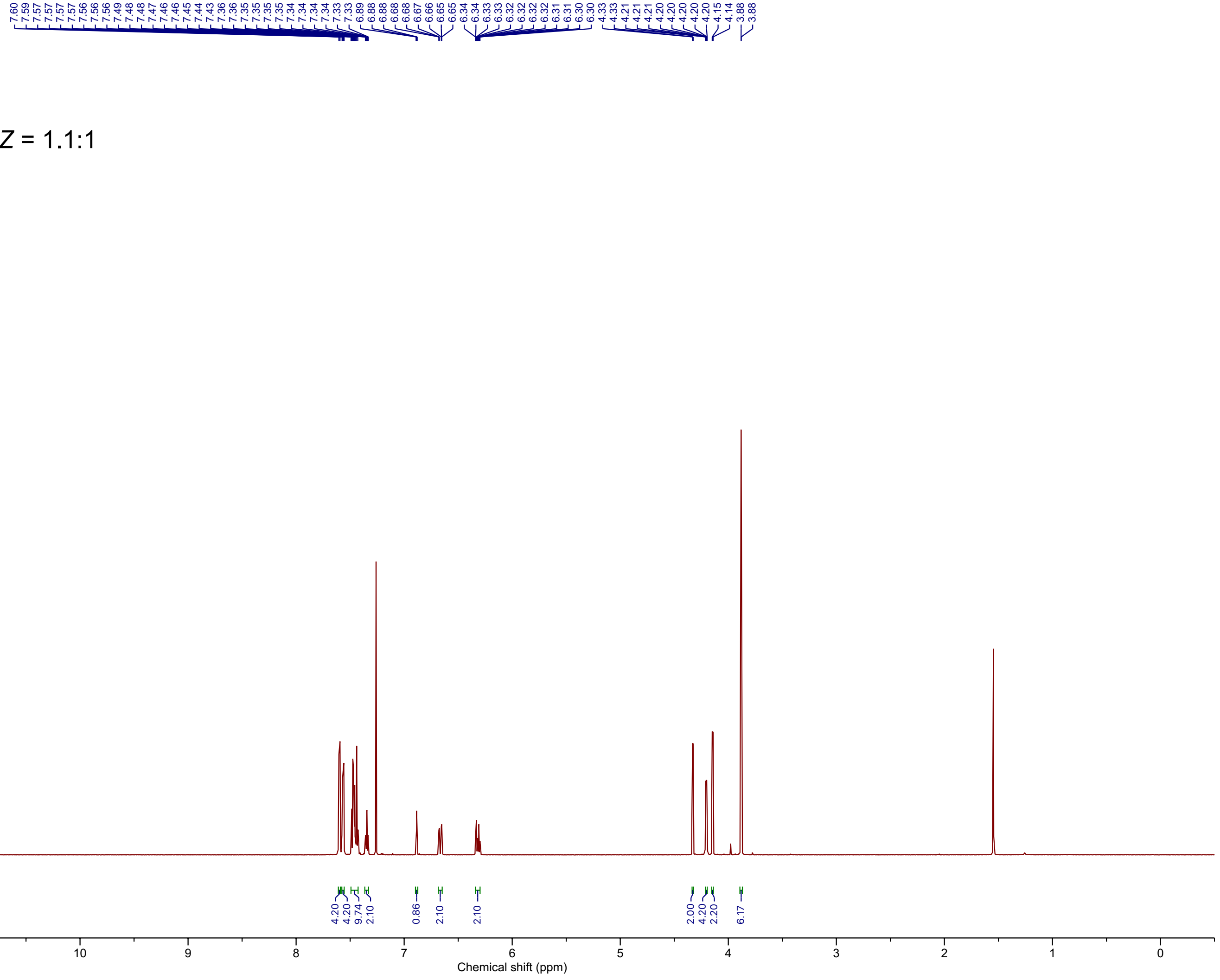

Supplementary Figure 34. ^{1}H NMR (700 MHz, $CDCl_3$) of **S22**.

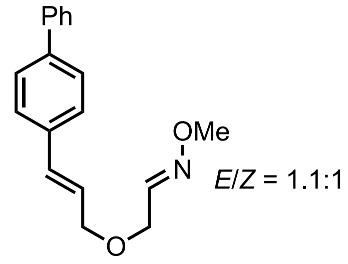

S22

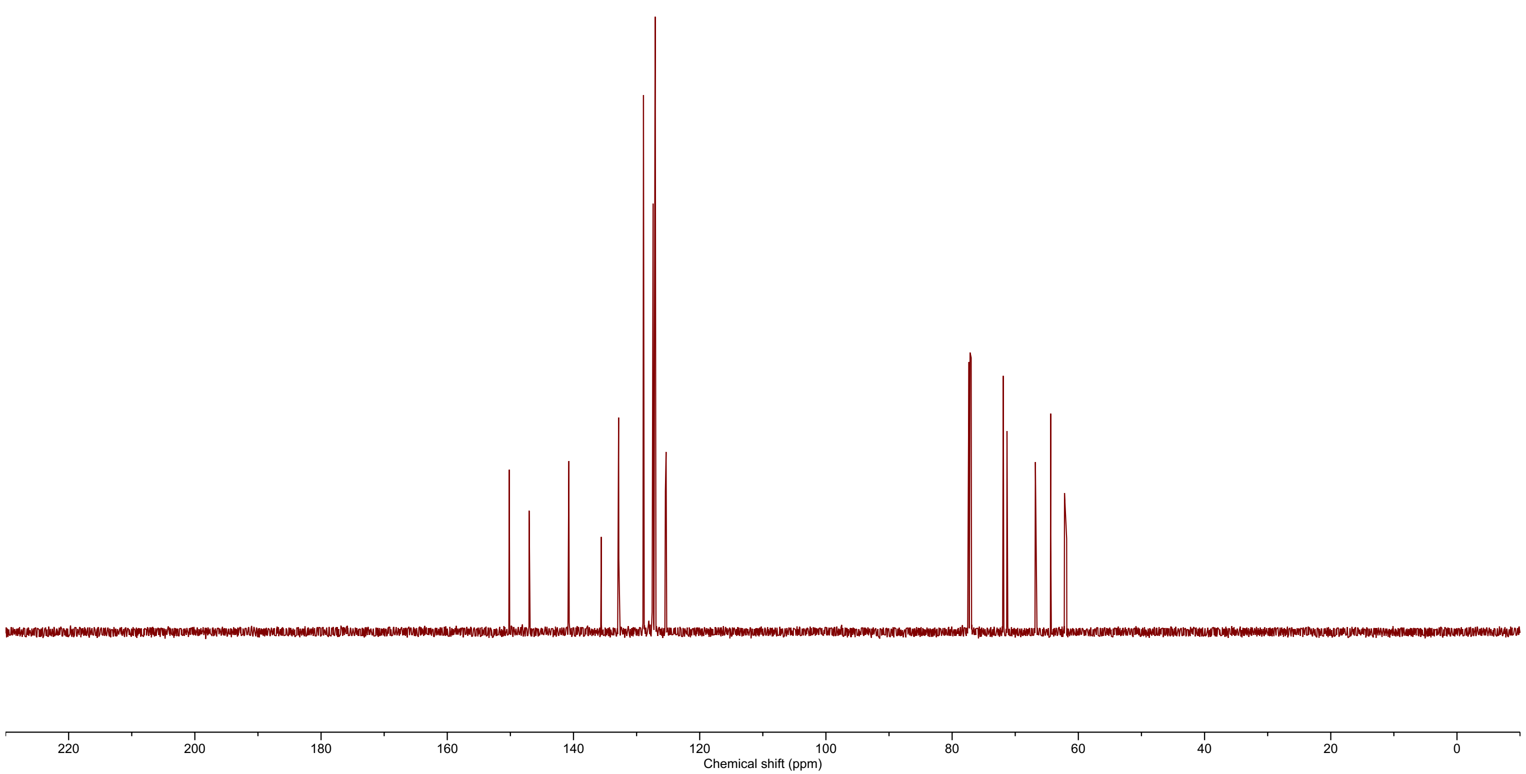

Supplementary Figure 35. ^{13}C NMR (176 MHz, $CDCl_3$) of **S22**.

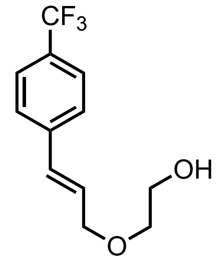

A23

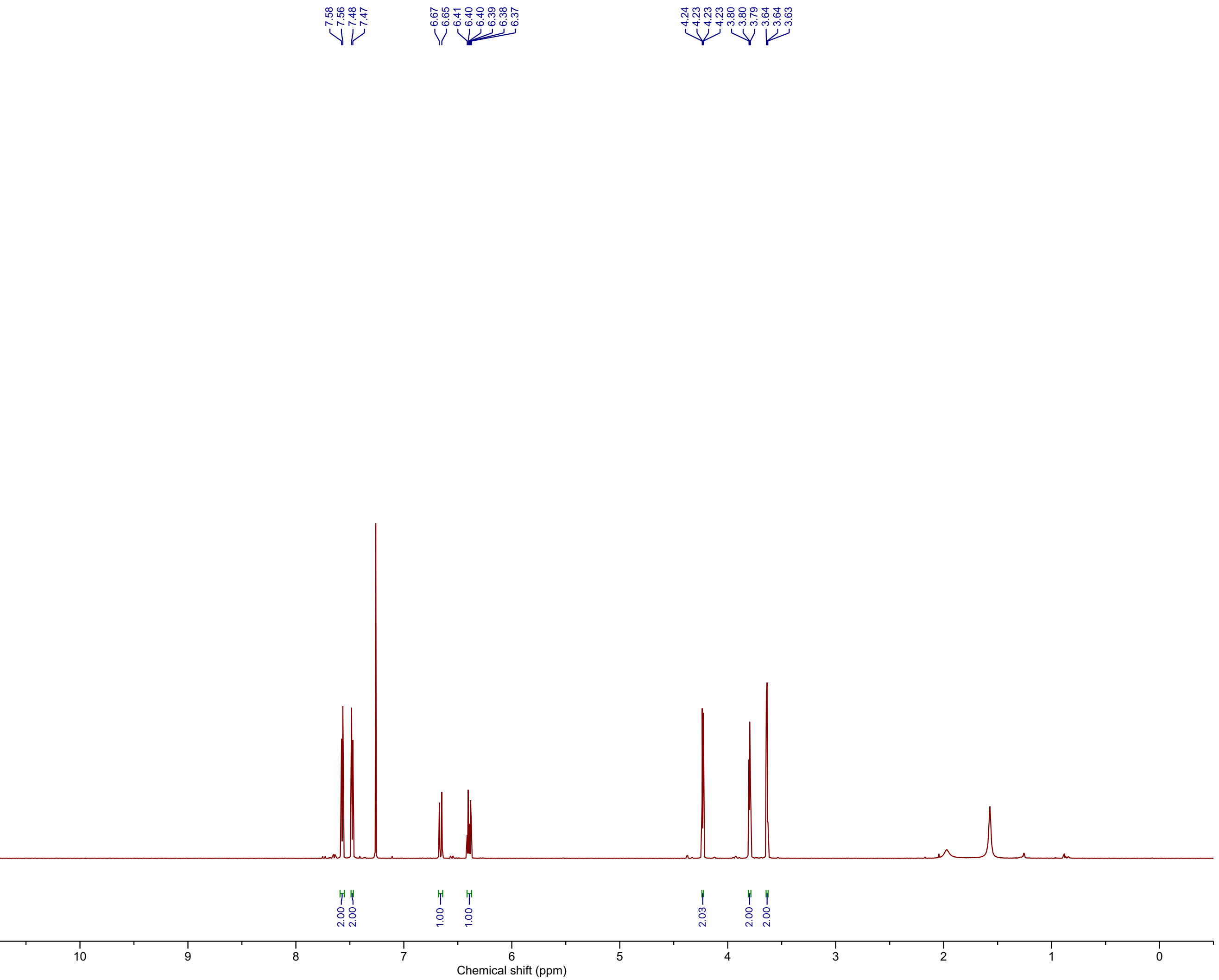

Supplementary Figure 36. ^{1}H NMR (700 MHz, $CDCl_3$) of **A23**.

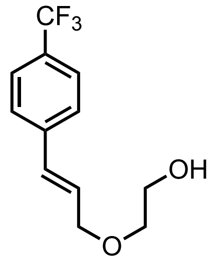

A23

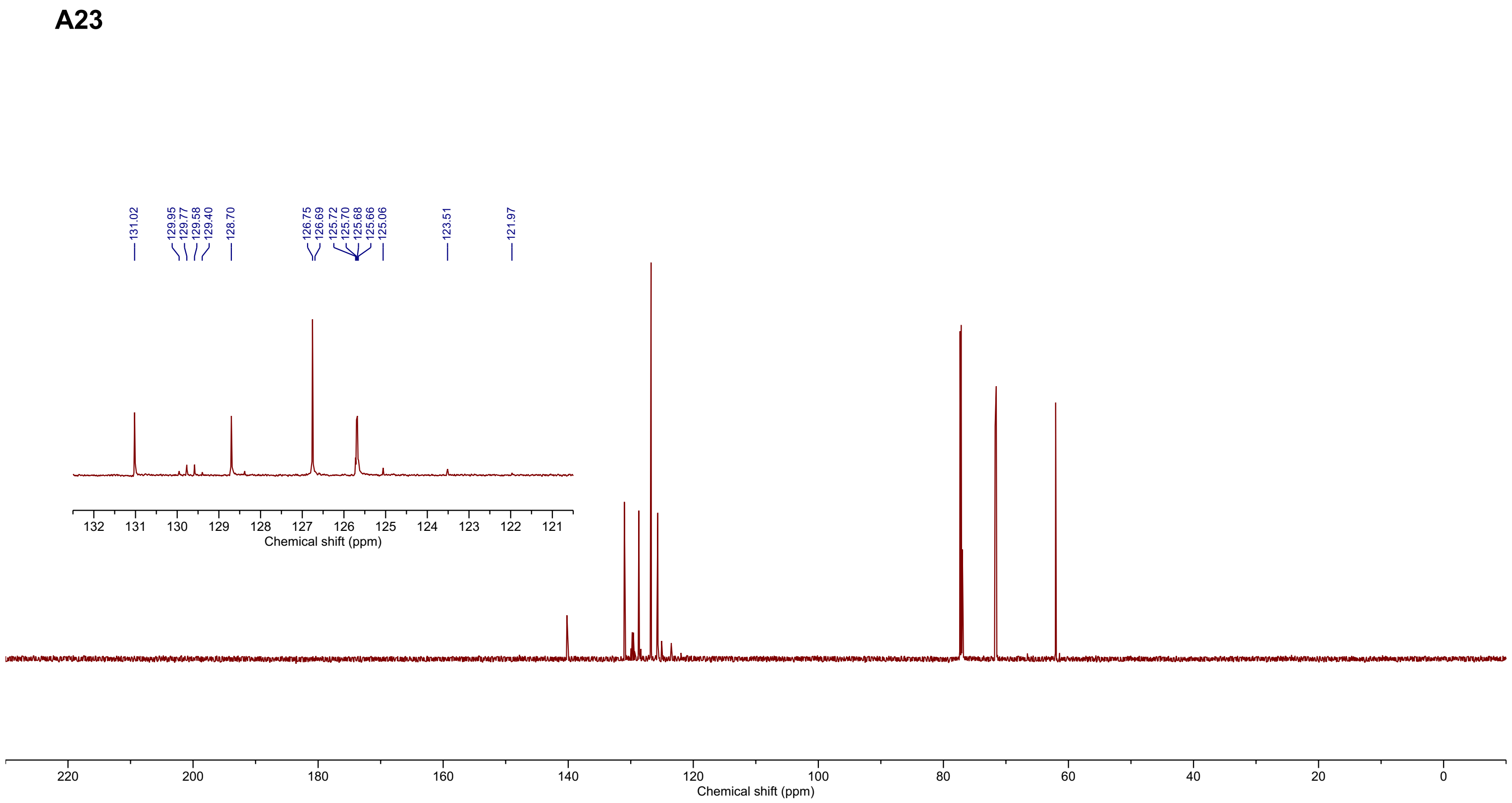

Supplementary Figure 37. ^{13}C NMR (176 MHz, $CDCl_3$) of **A23**.

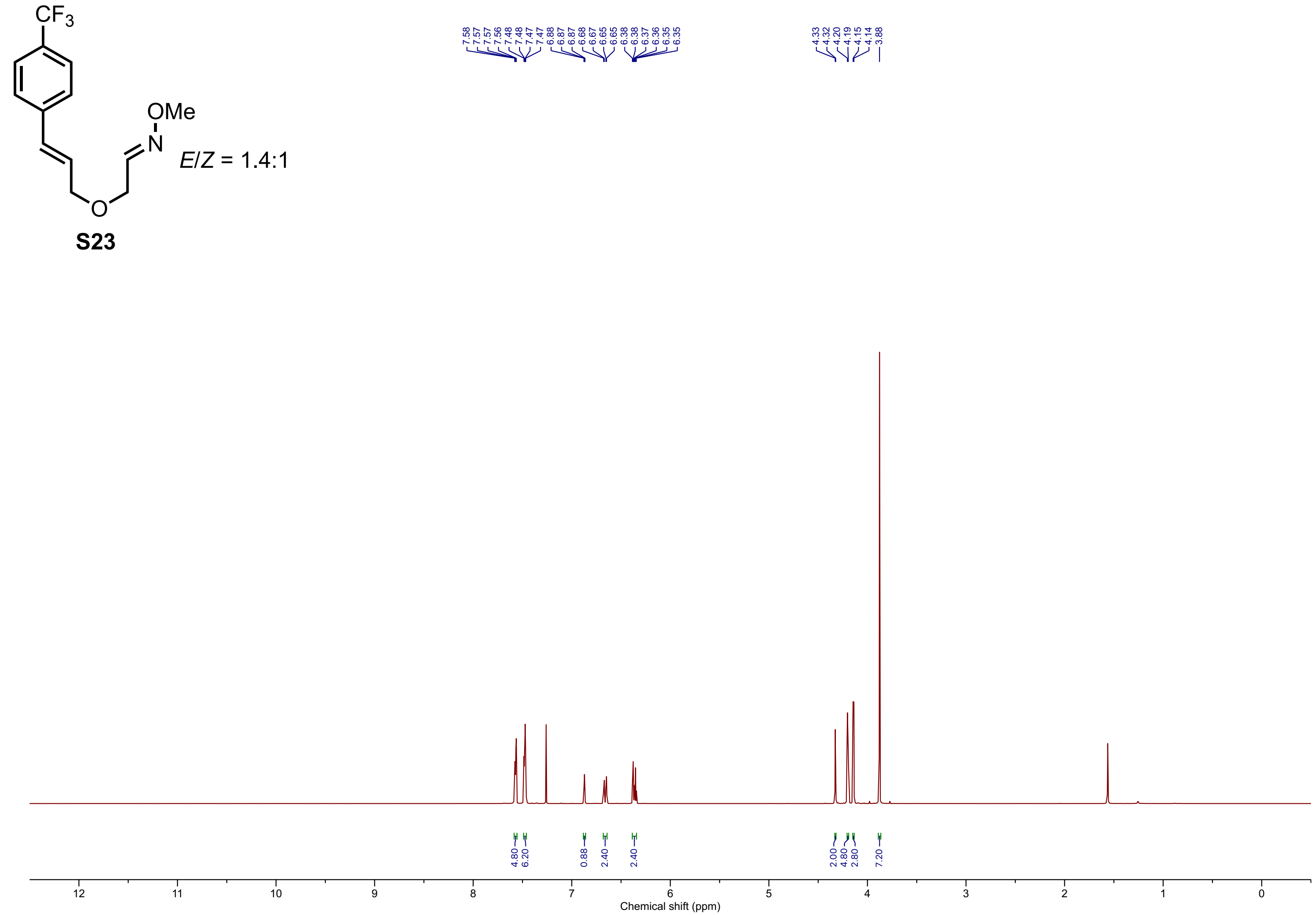

Supplementary Figure 38. ^{1}H NMR (700 MHz, $CDCl_3$) of **S23**.

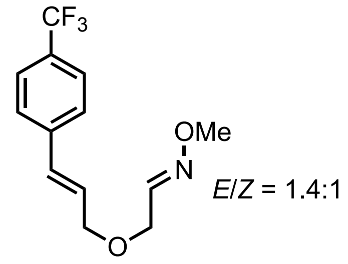

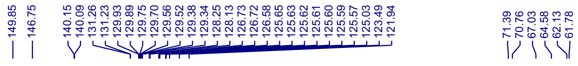

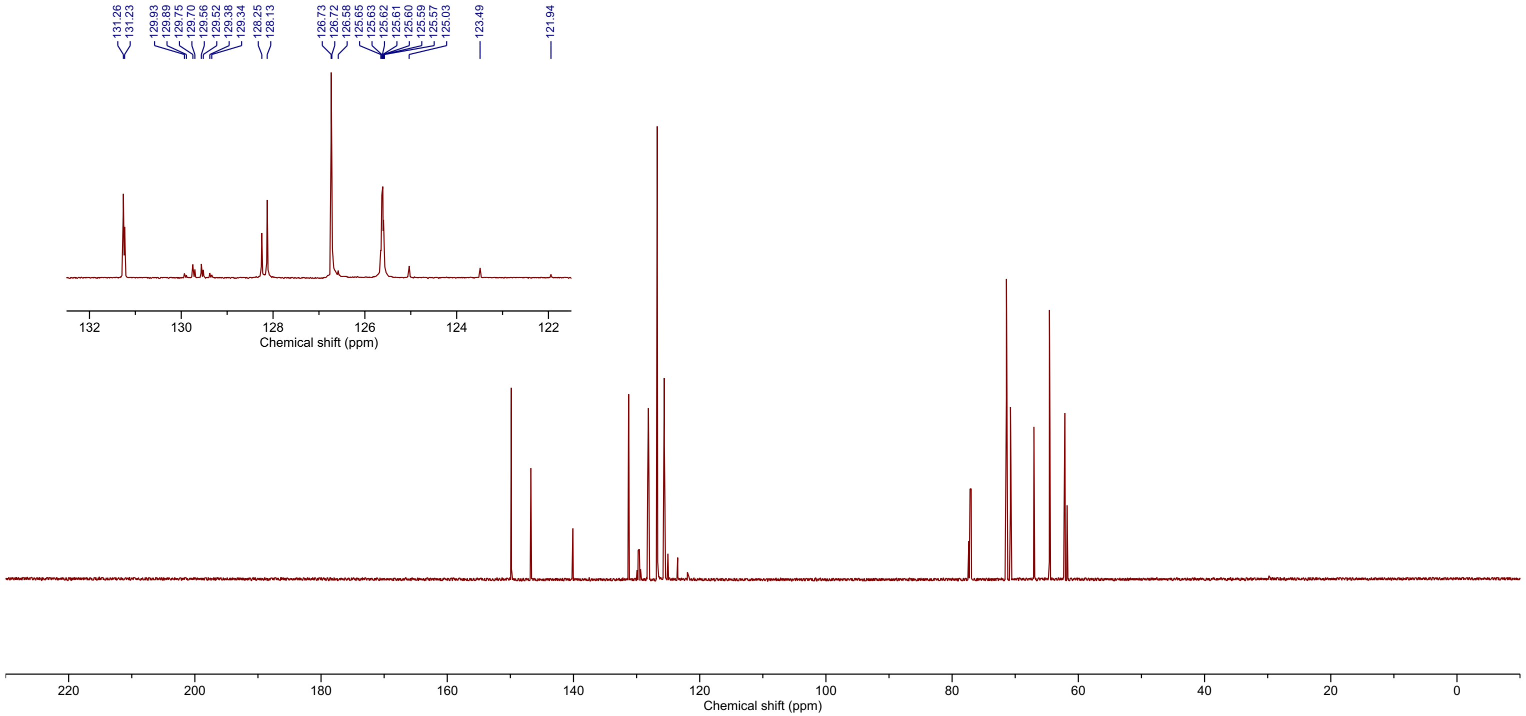

Supplementary Figure 39. ^{13}C NMR (176 MHz, $CDCl_3$) of **S23**.

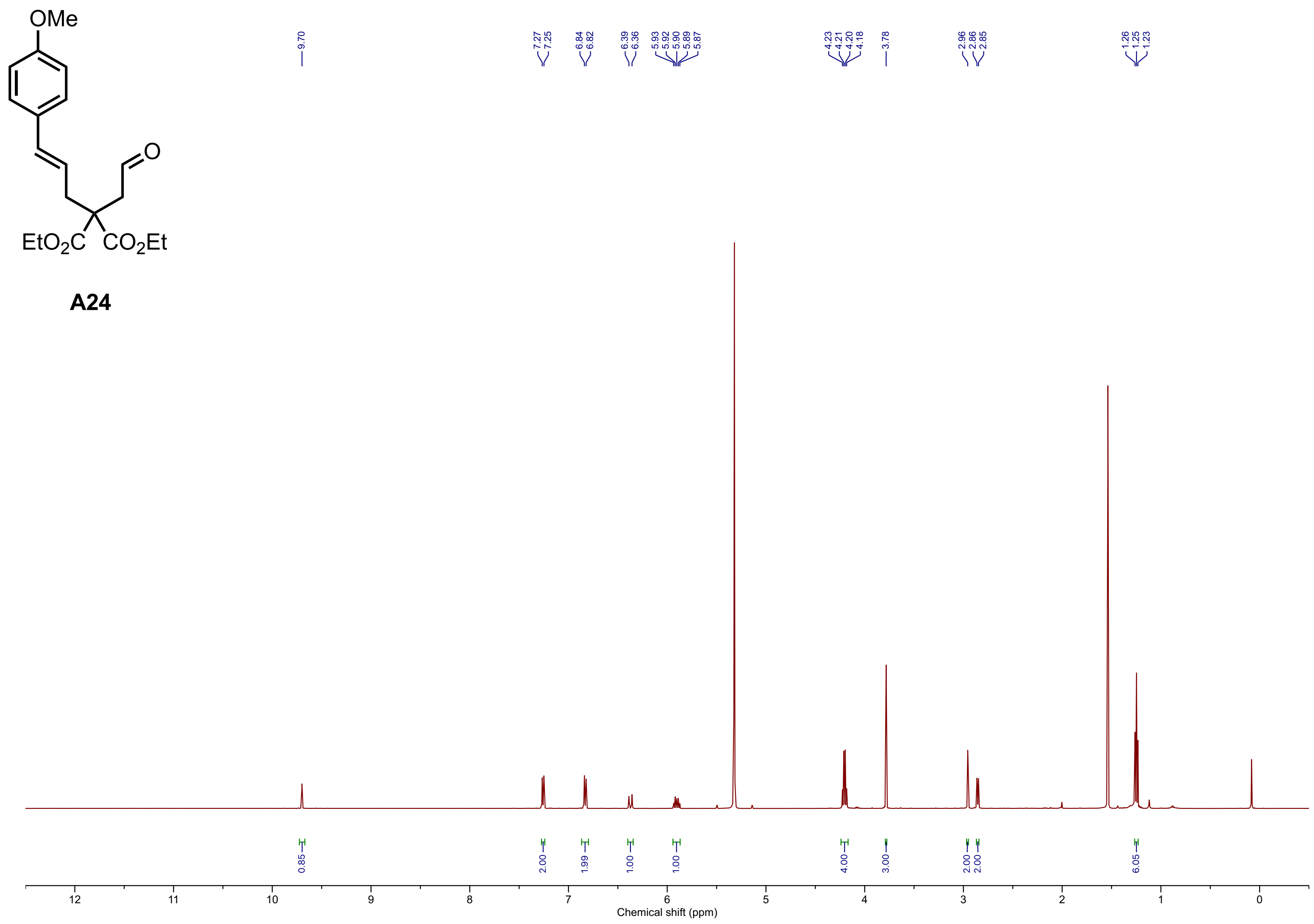

A24

Supplementary Figure 40. ^{1}H NMR (500 MHz, CD_2Cl_2) of **A24**.

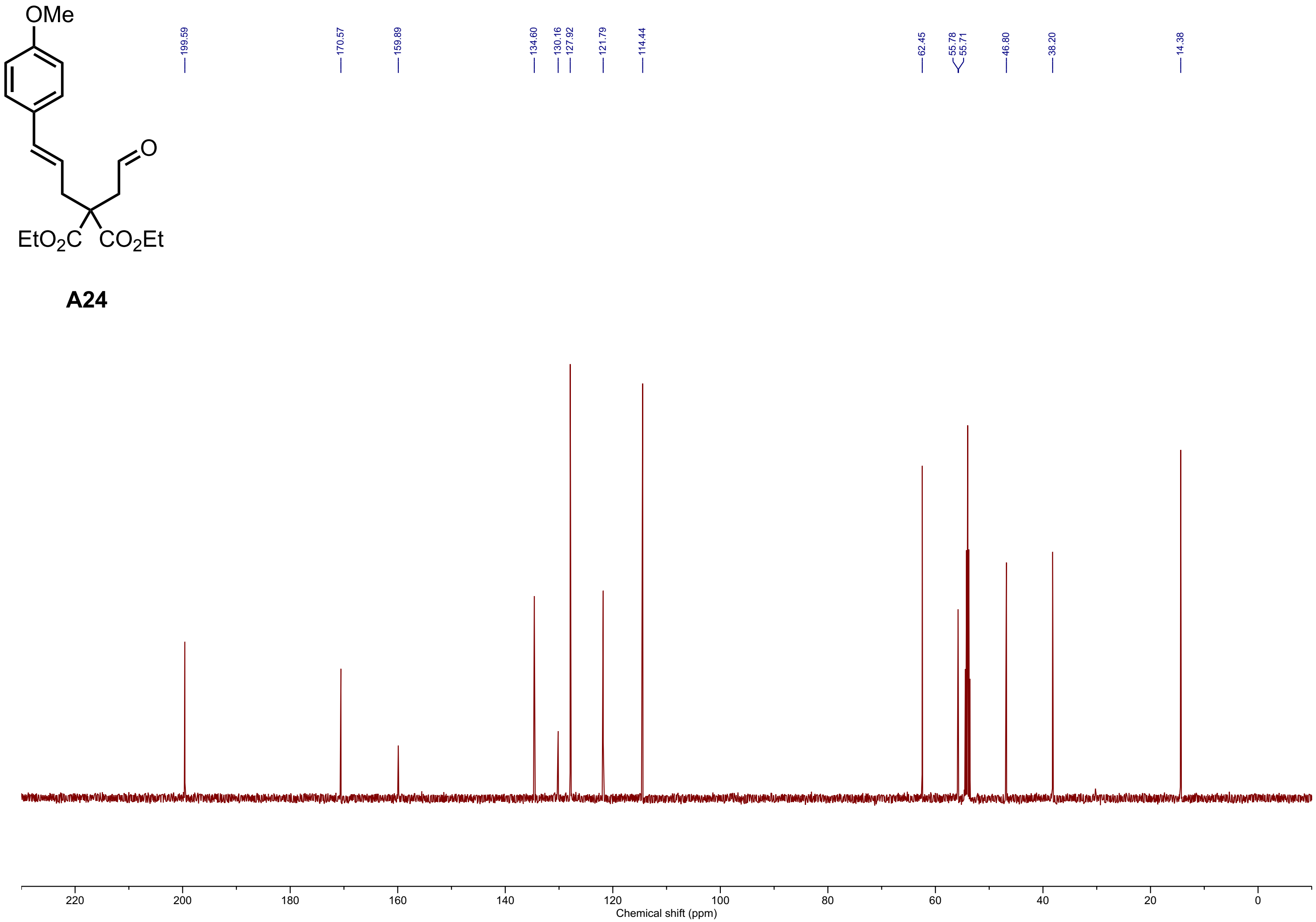

Supplementary Figure 41. ^{13}C NMR (126 MHz, CD_2Cl_2) of **A24**.

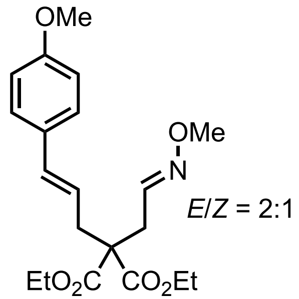

S24

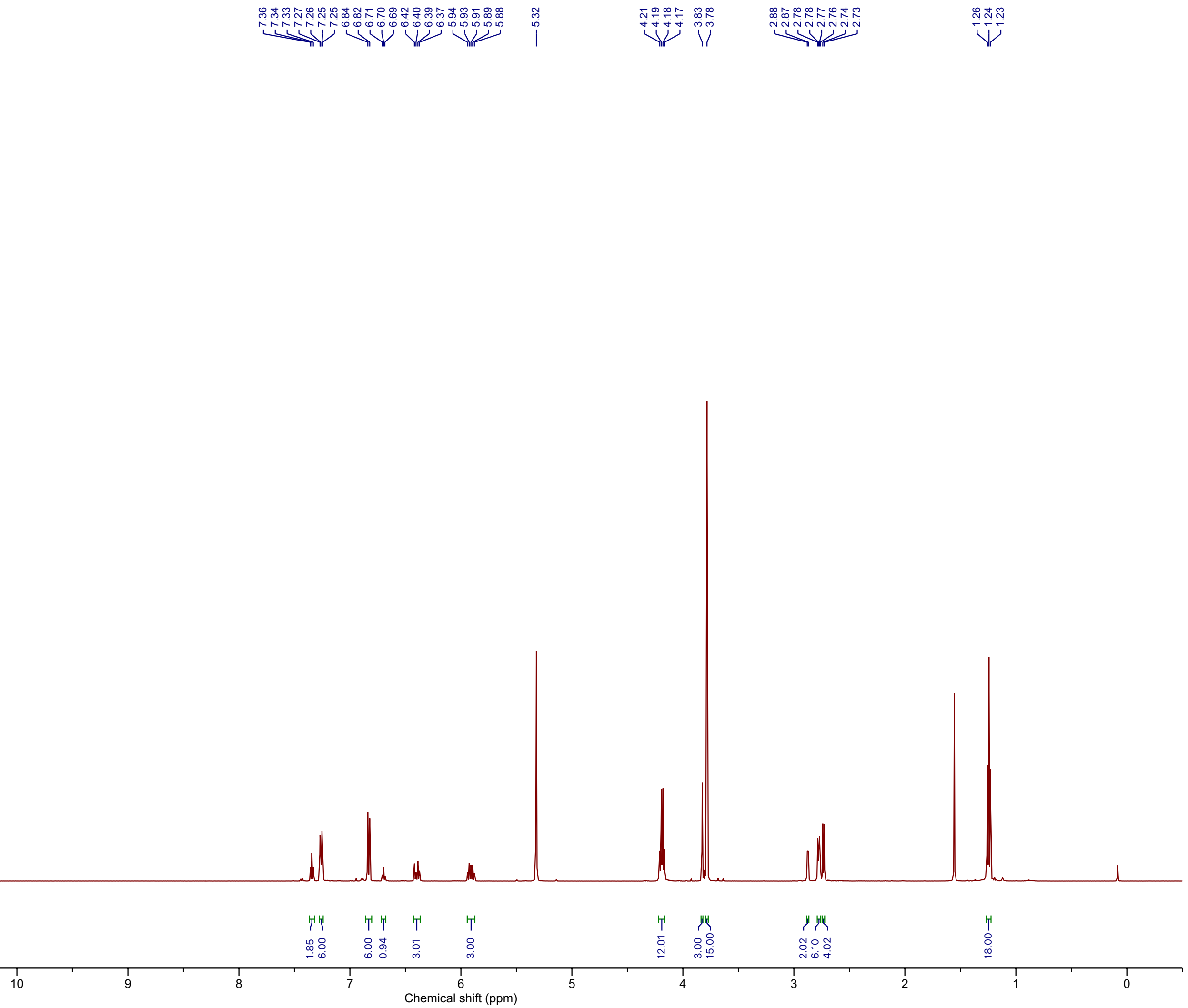

Supplementary Figure 42. ^{1}H NMR (500 MHz, CD_2Cl_2) of **S24**.

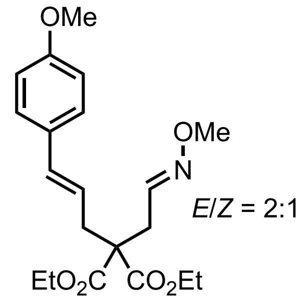

S24

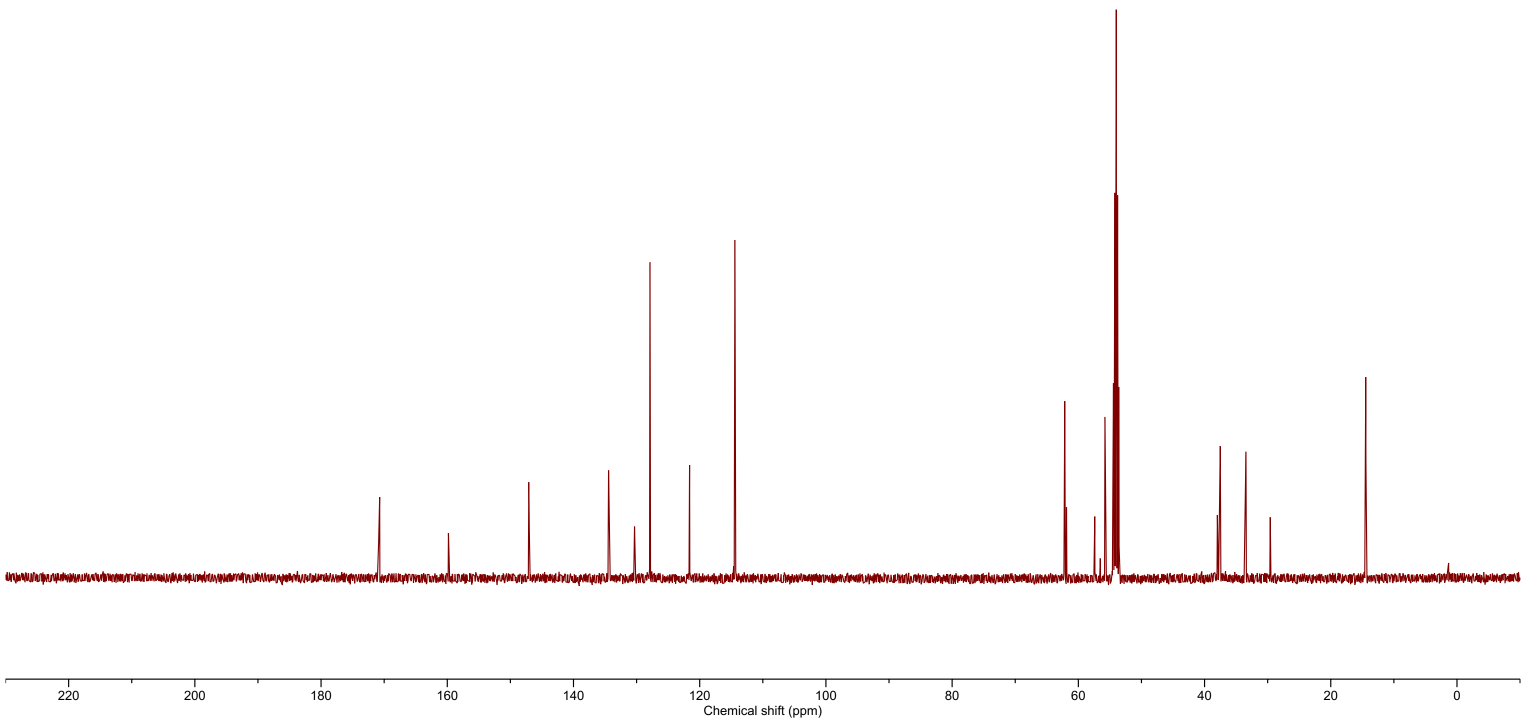

Supplementary Figure 43. ^{13}C NMR (126 MHz, CD_2Cl_2) of **S24**.

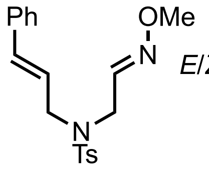

E/*Z* = 1.2:1

S25

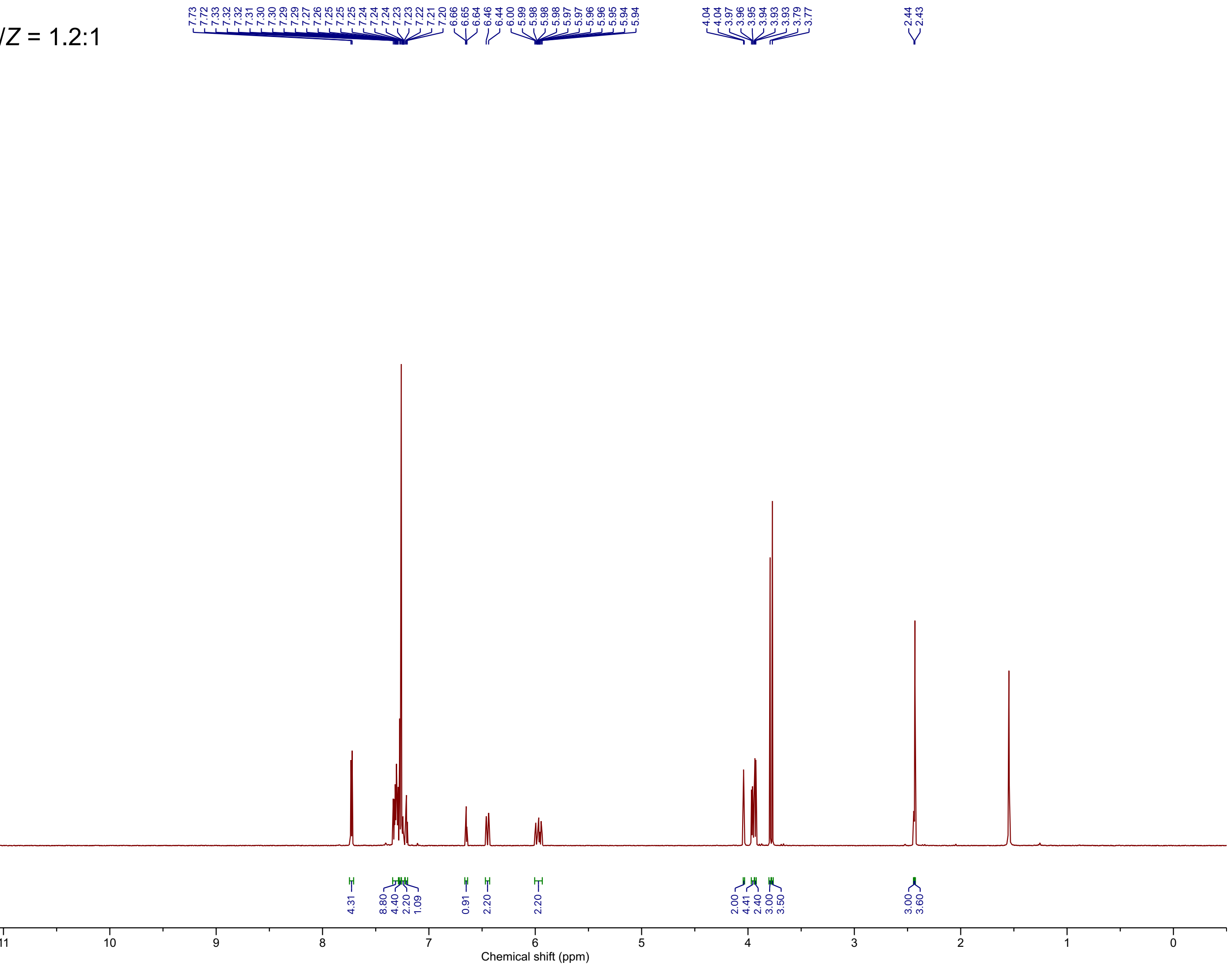

Supplementary Figure 44. ^{1}H NMR (700 MHz, $CDCl_3$) of **S25**.

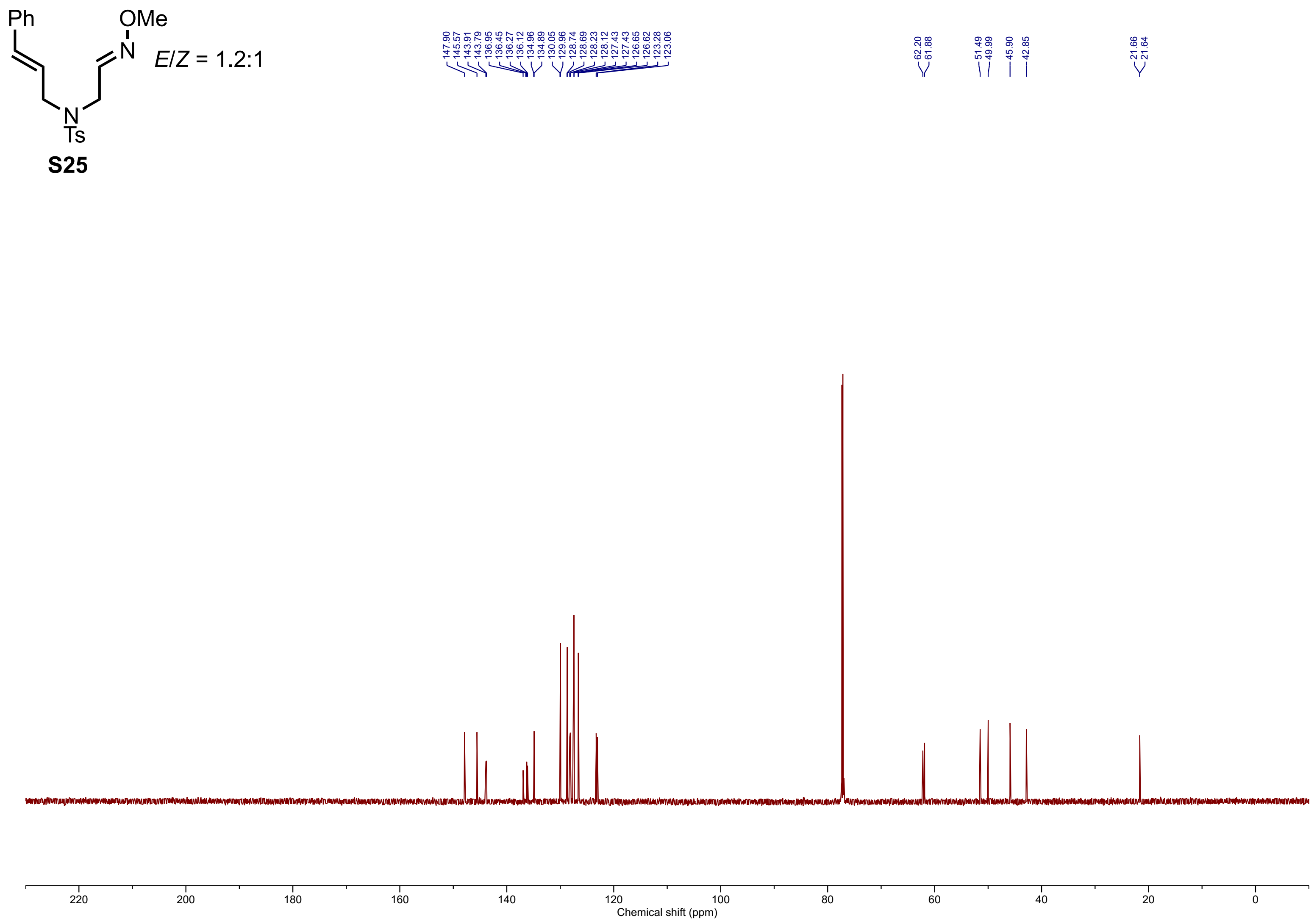

S25

Supplementary Figure 45. ^{13}C NMR (176 MHz, $CDCl_3$) of **S25**.

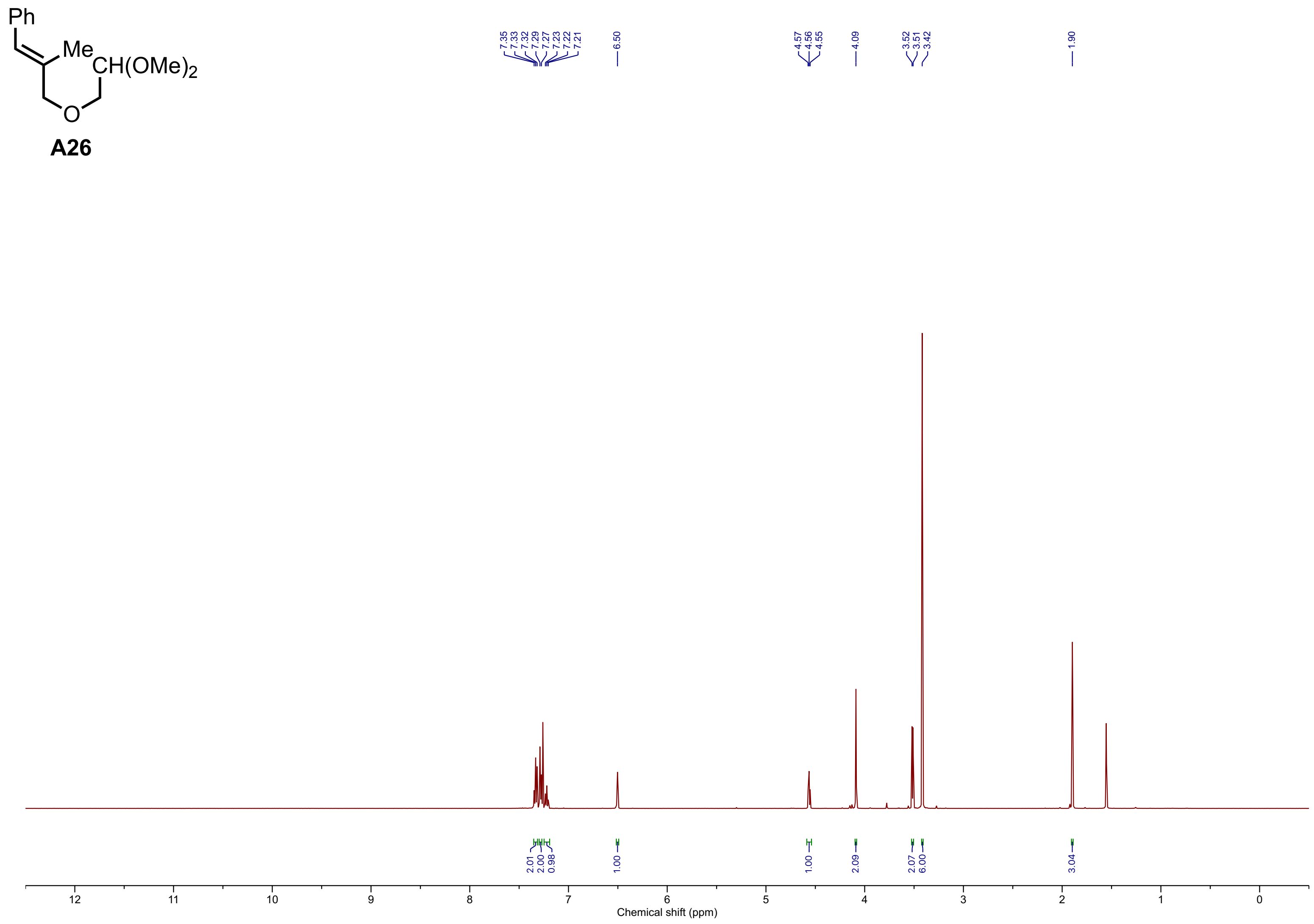

Supplementary Figure 46. ^{1}H NMR (500 MHz, $CDCl_3$) of **A26**.

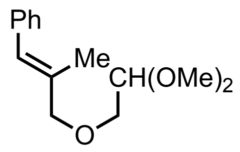

A26

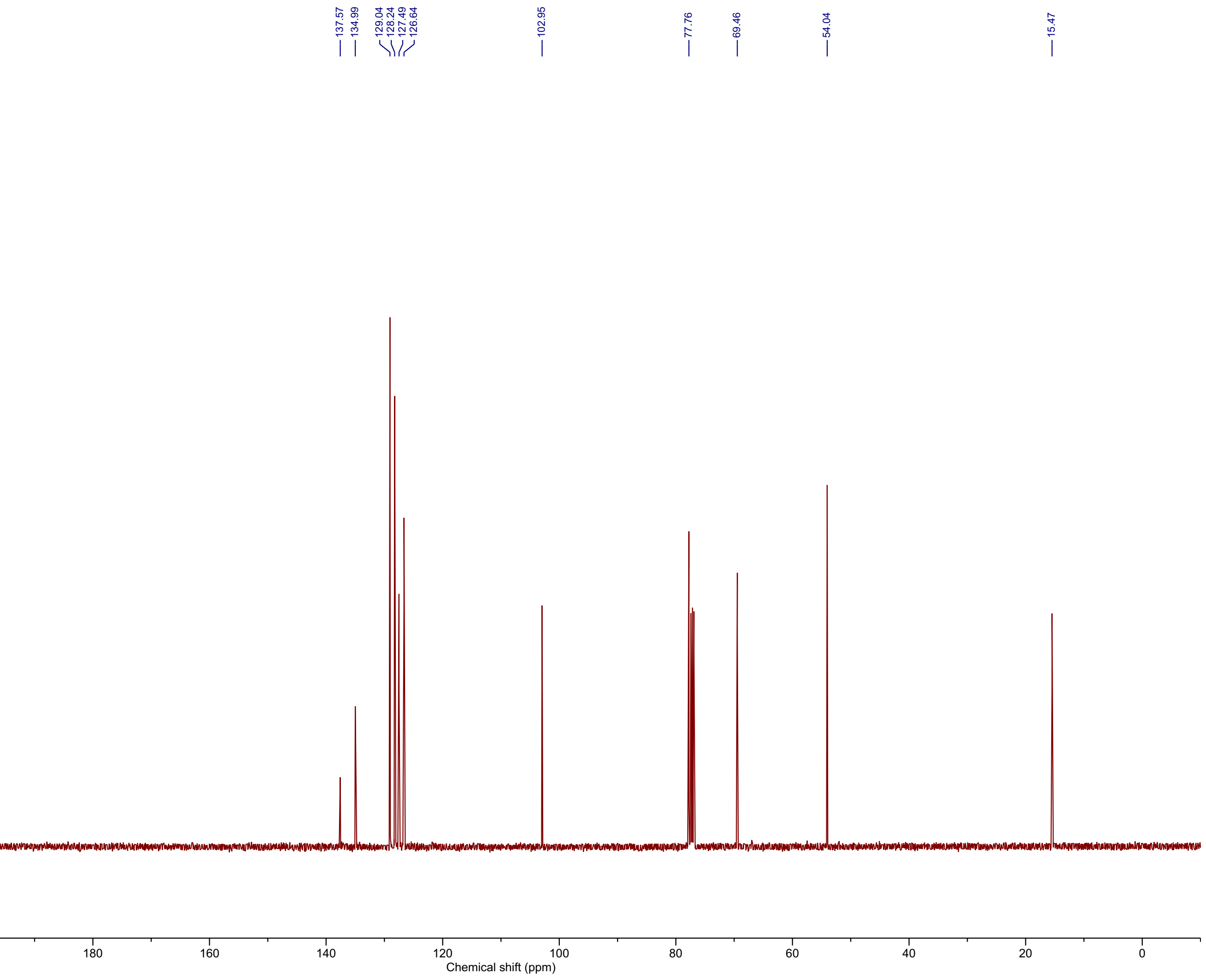

Supplementary Figure 47. ^{13}C NMR (126 MHz, $CDCl_3$) of **A26**.

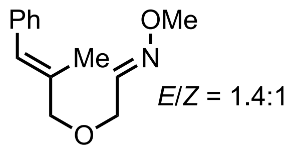

S26

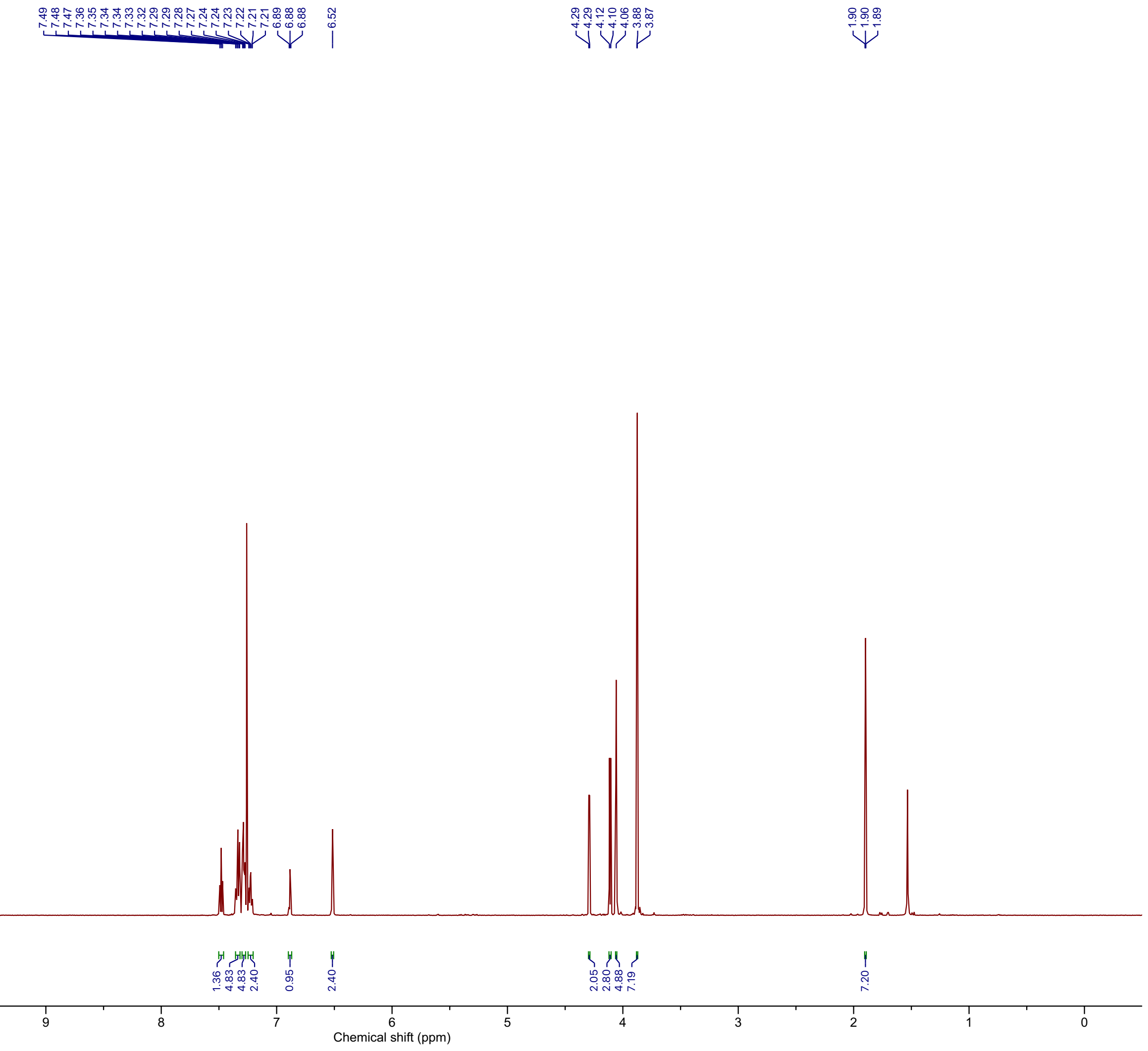

Supplementary Figure 48. ^{1}H NMR (500 MHz, $CDCl_3$) of **S26**.

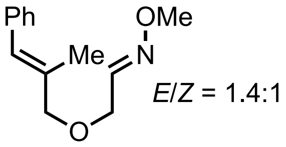

S26

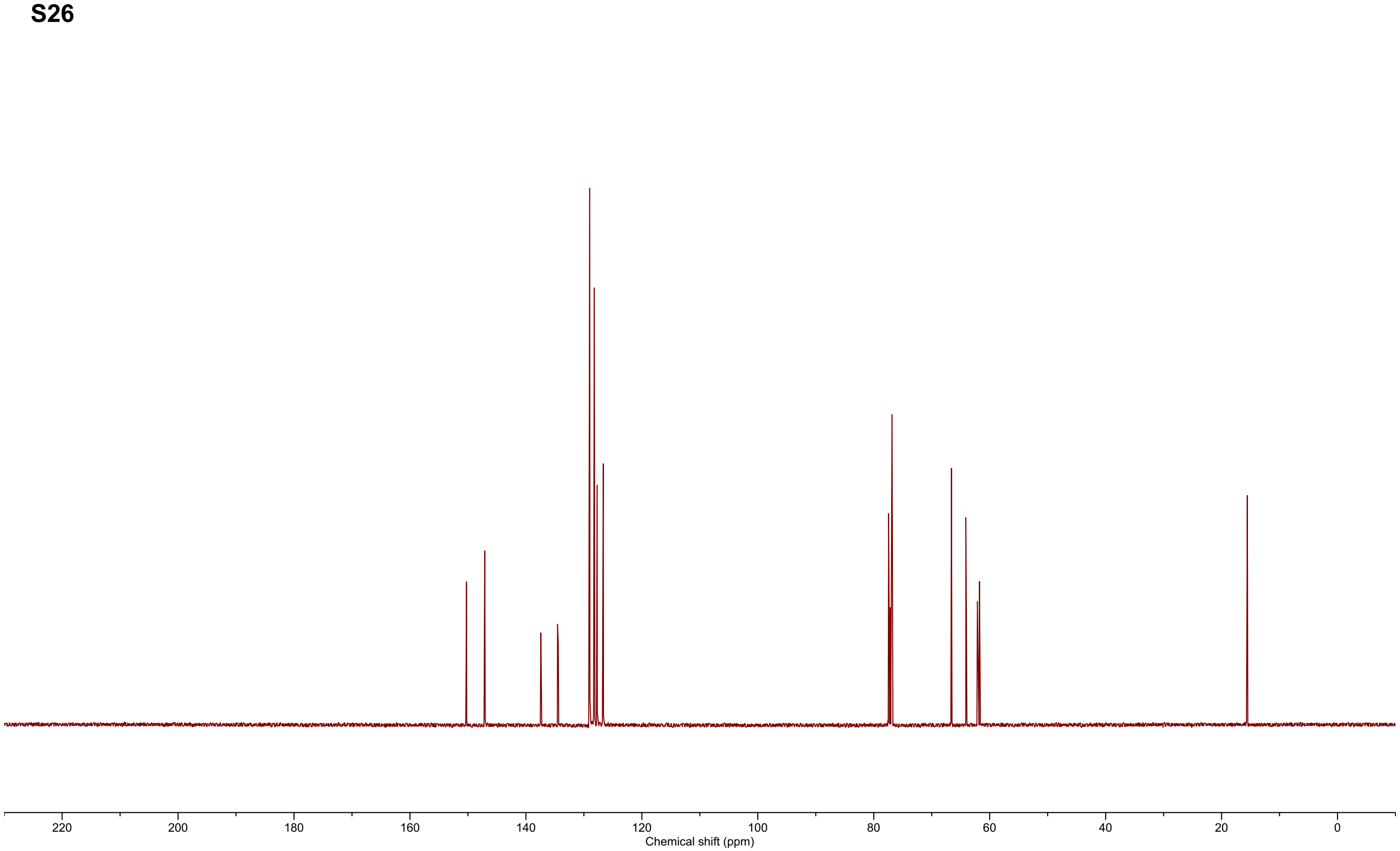

Supplementary Figure 49. ^{13}C NMR (176 MHz, $CDCl_3$) of **S26**.

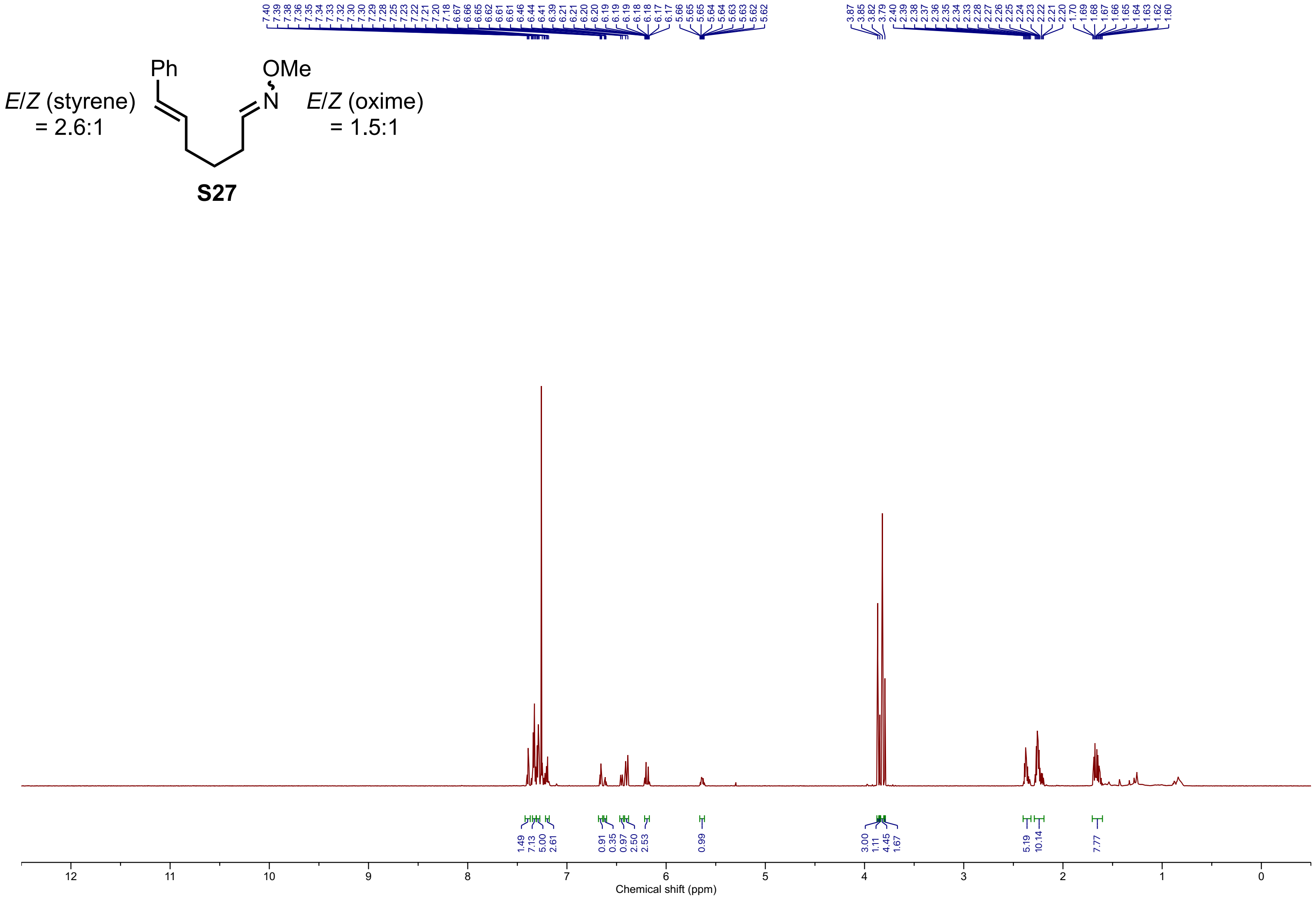

Supplementary Figure 50. ^{1}H NMR (700 MHz, $CDCl_3$) of **S27**.

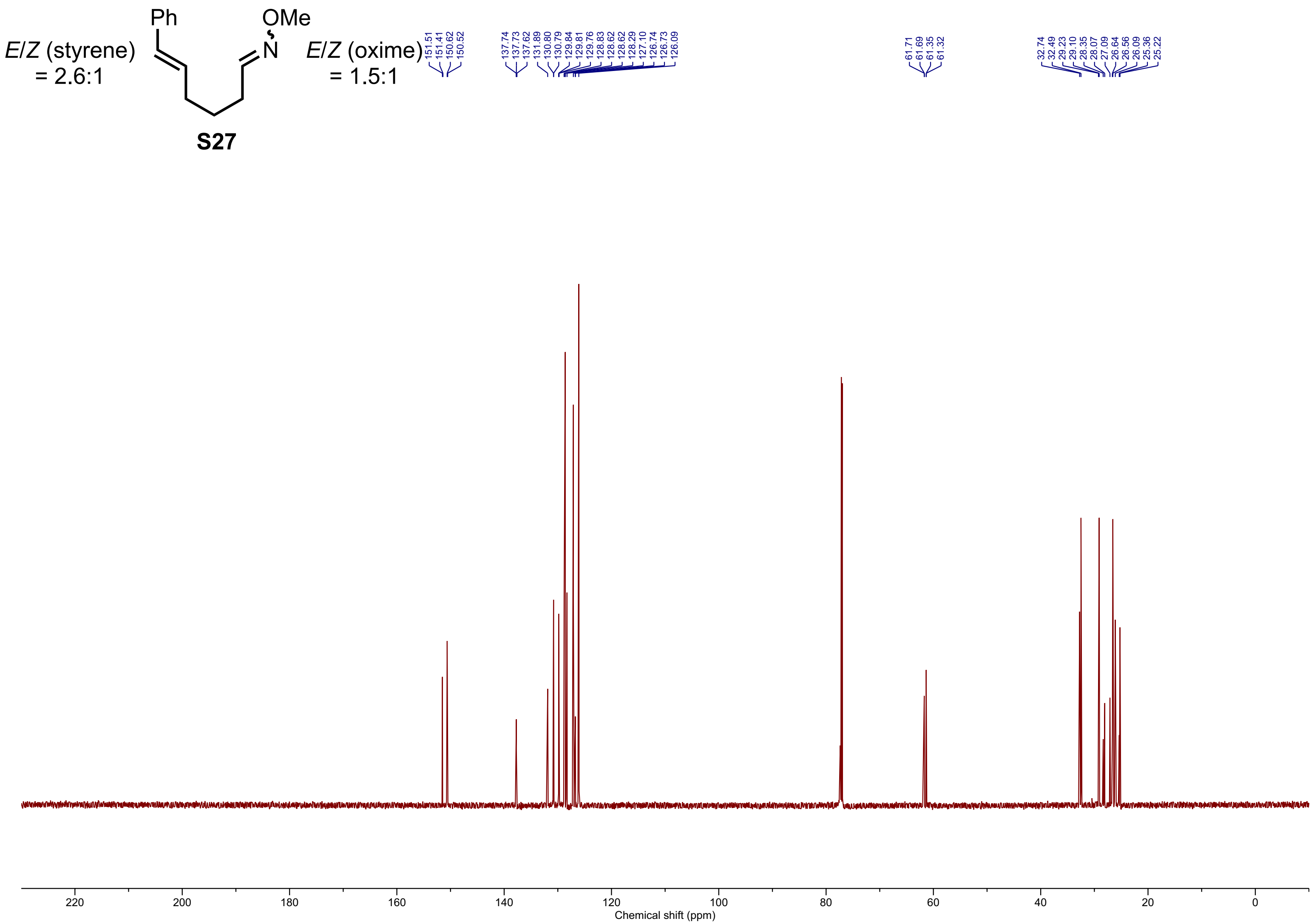

Supplementary Figure 51. ^{13}C NMR (176 MHz, $CDCl_3$) of **S27**.

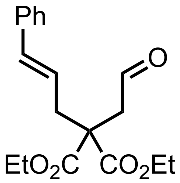

A28

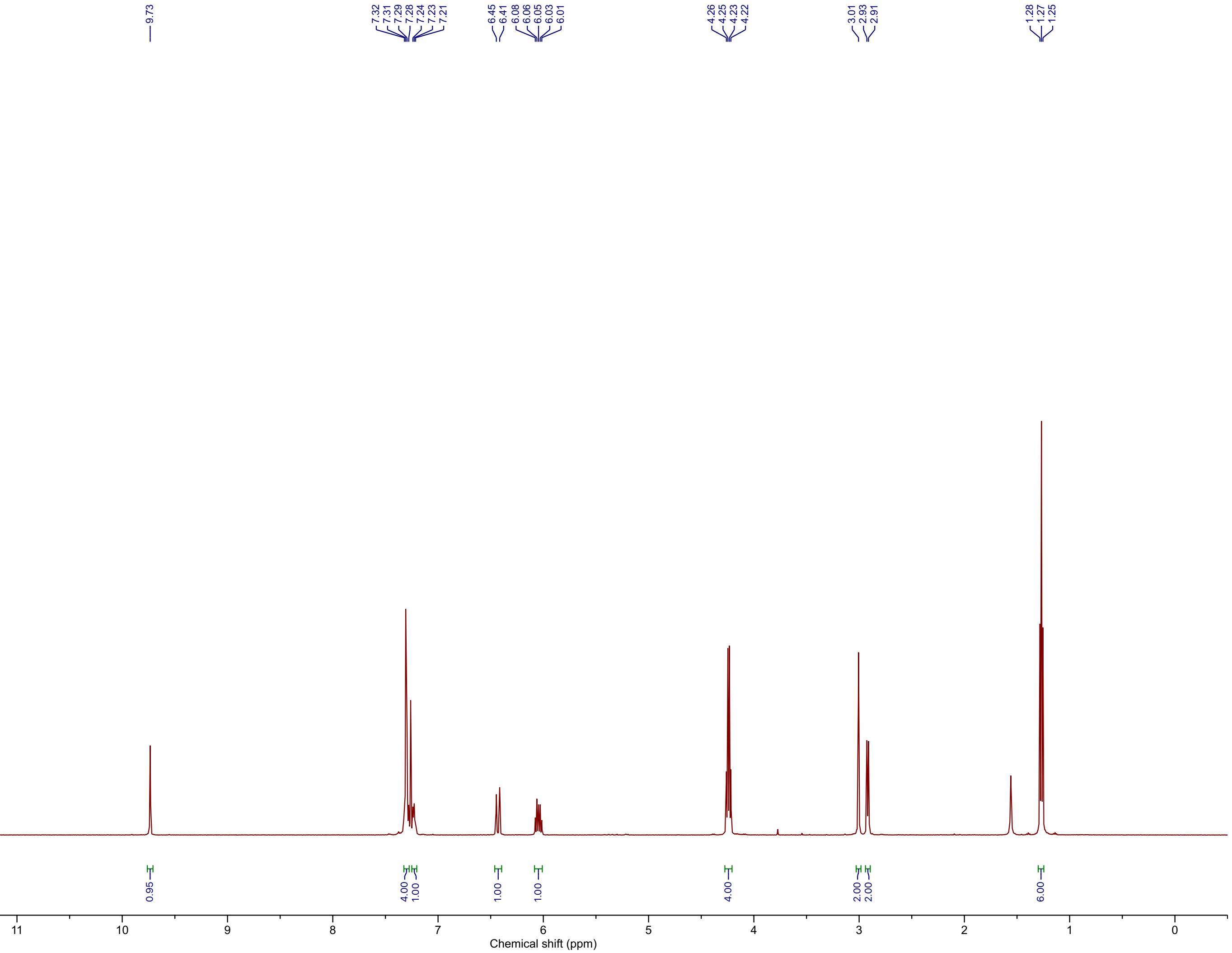

Supplementary Figure 52. ^{1}H NMR (500 MHz, $CDCl_3$) of **A28**.

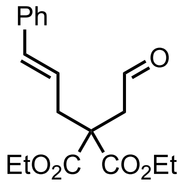

A28

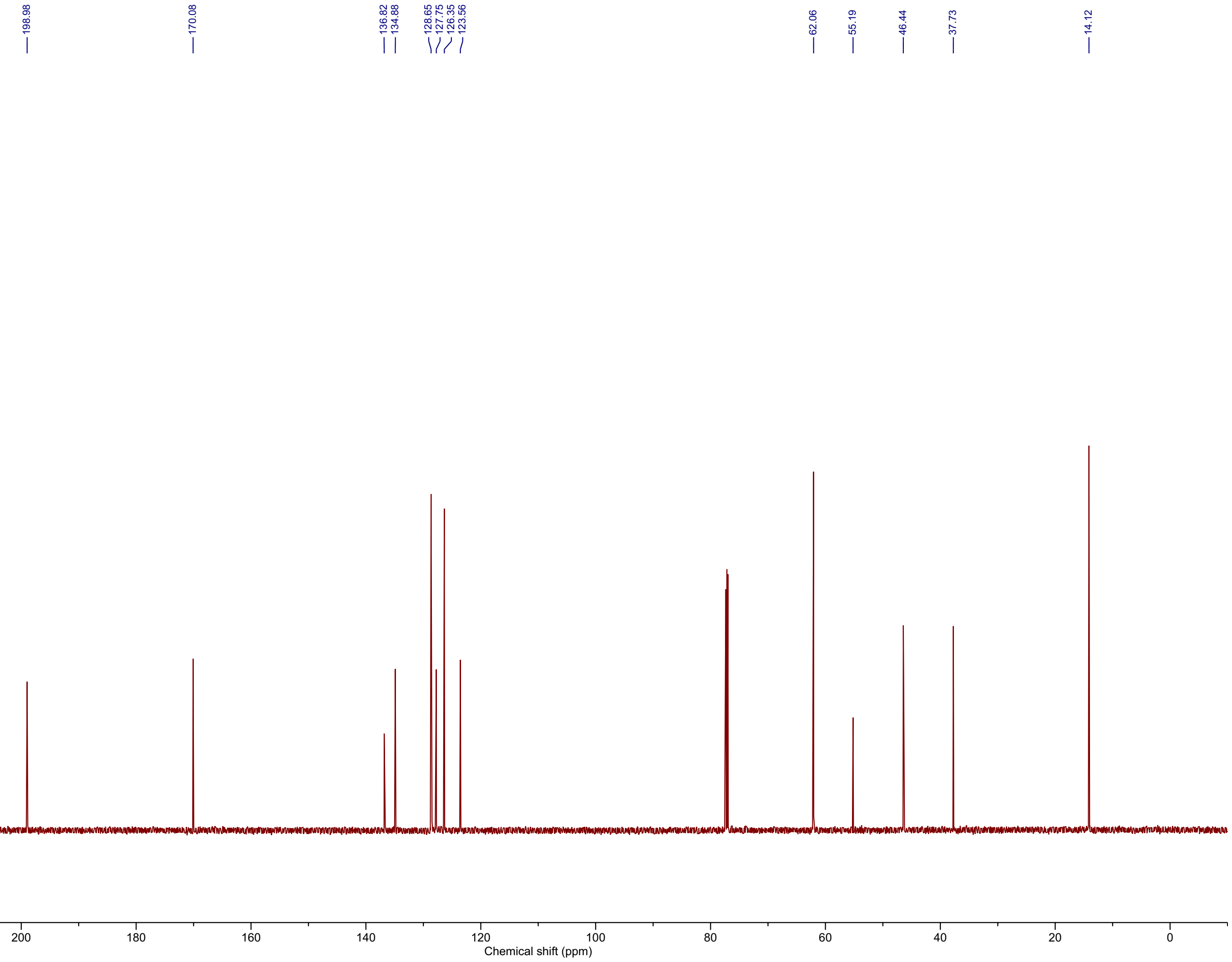

Supplementary Figure 53. ^{13}C NMR (176 MHz, $CDCl_3$) of **A28**.

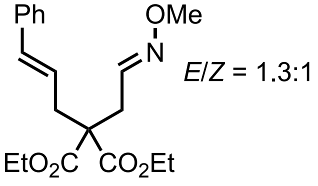

S28

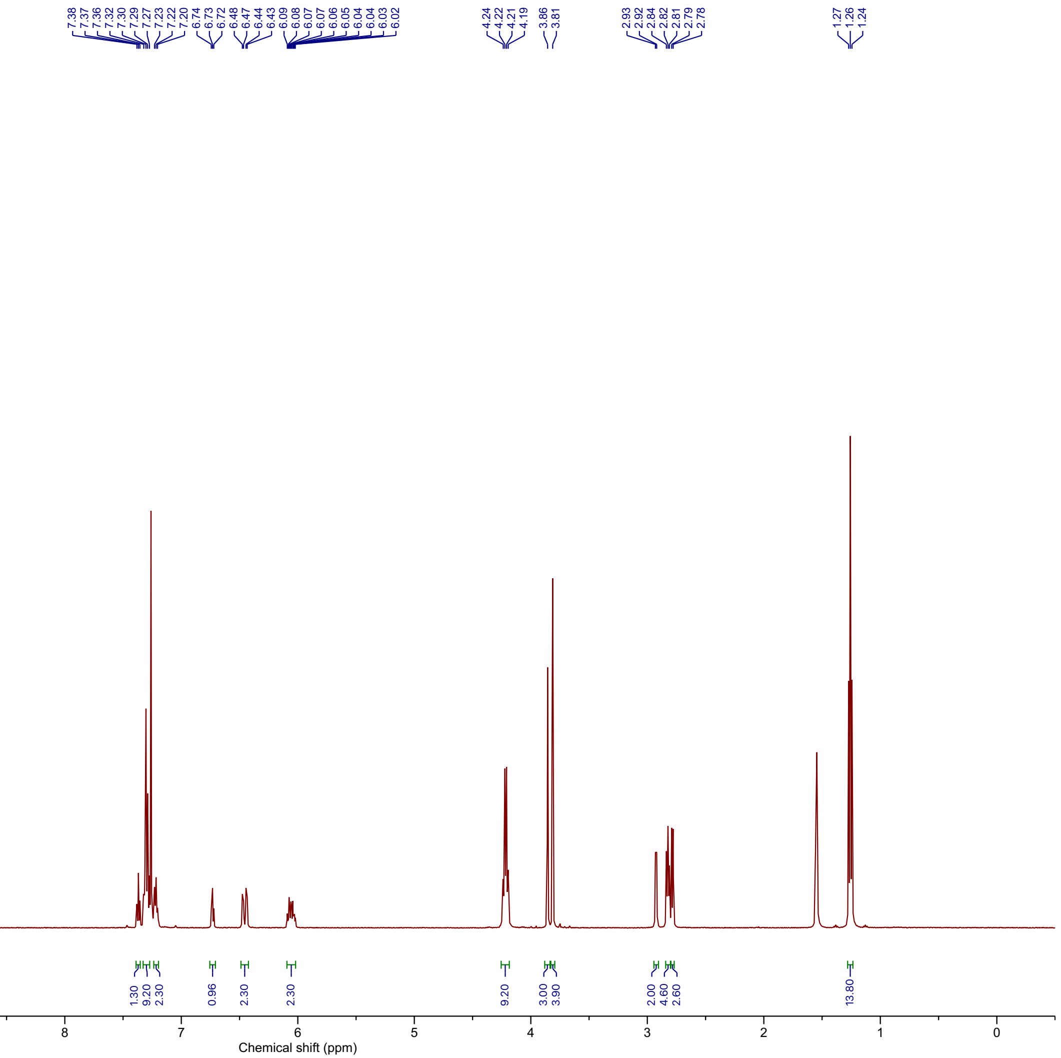

Supplementary Figure 54. ^{1}H NMR (500 MHz, $CDCl_3$) of **S28**.

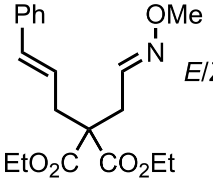

E/*Z* = 1.3:1

S28

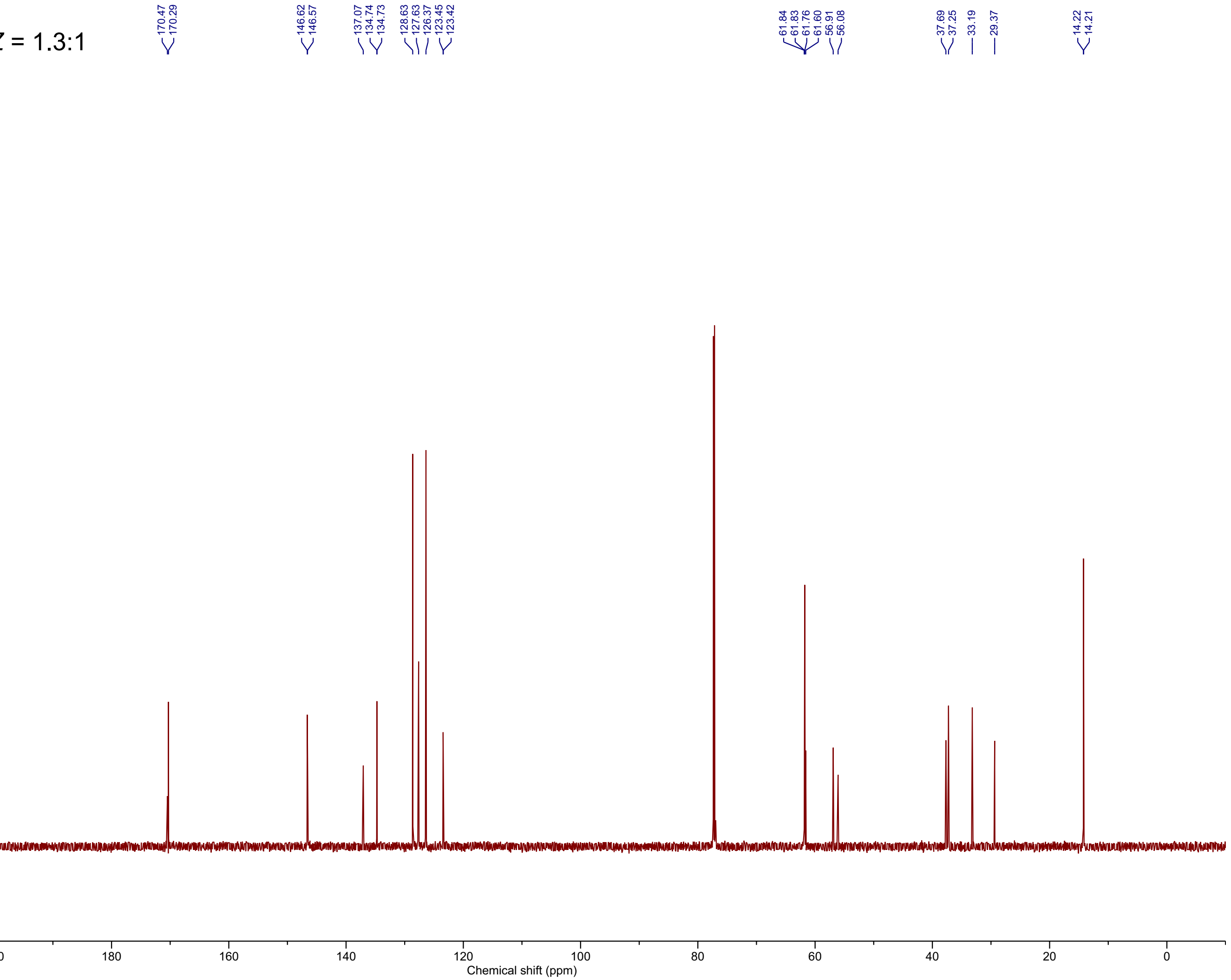

Supplementary Figure 55. ^{13}C NMR (176 MHz, $CDCl_3$) of **S28**.

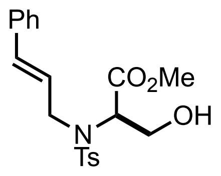

A1-29

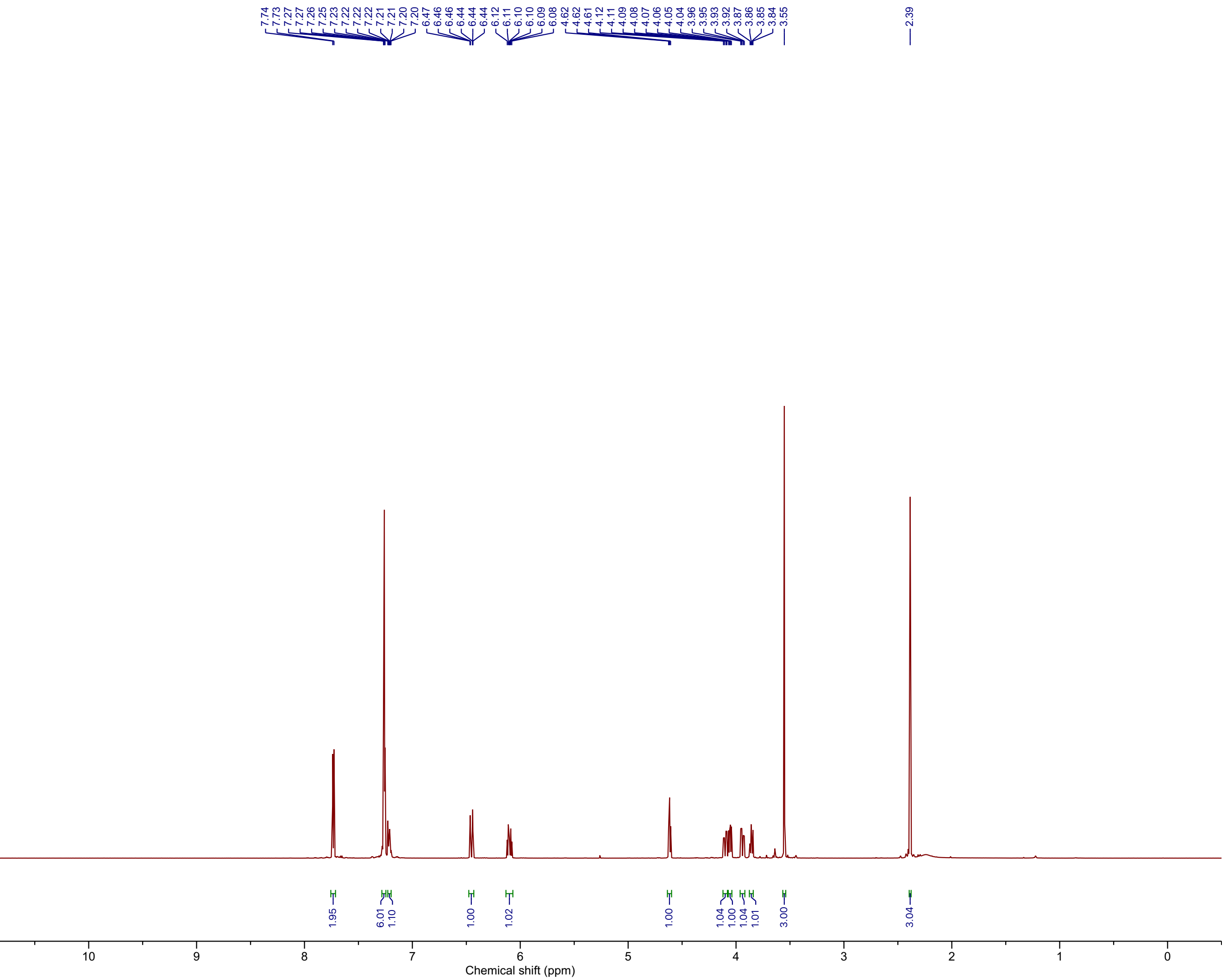

Supplementary Figure 56. ^{1}H NMR (700 MHz, $CDCl_3$) of **A1-29**.

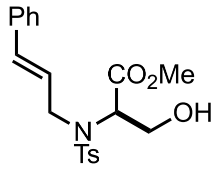

A1-29

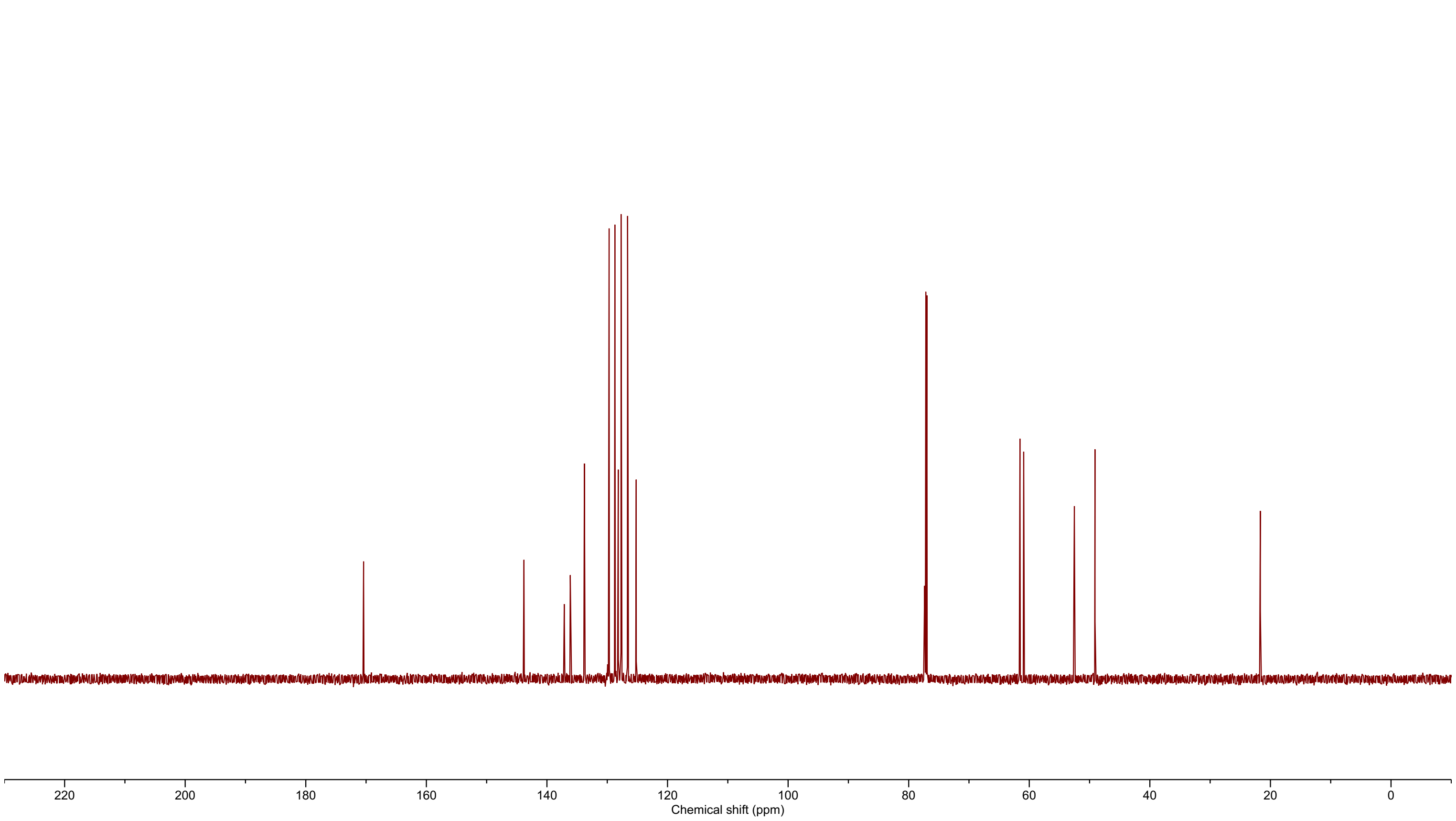

Supplementary Figure 57. ^{13}C NMR (176 MHz, $CDCl_3$) of **A1-29**.

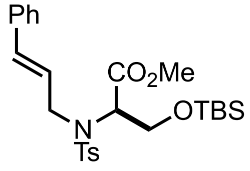

A2-29

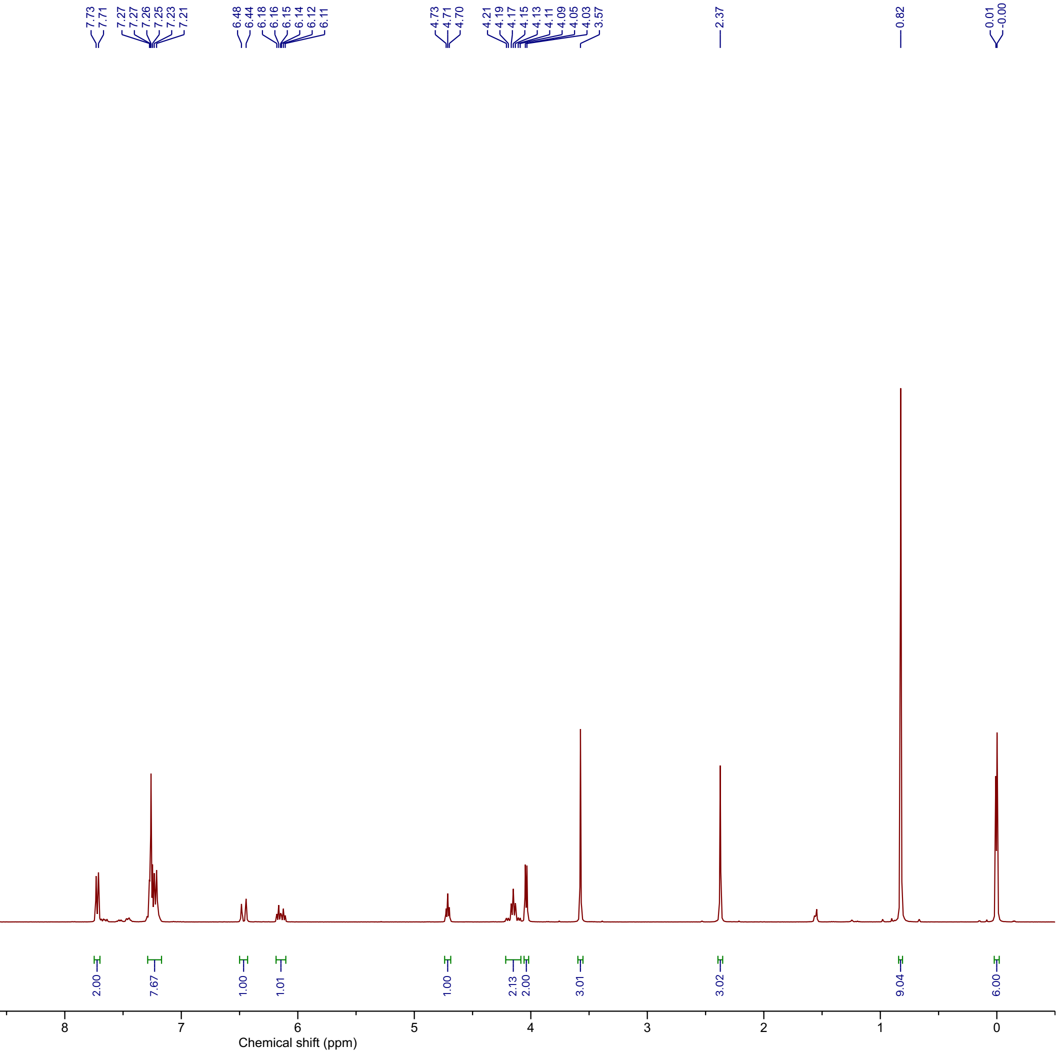

Supplementary Figure 58. ^{1}H NMR (400 MHz, CDCl$_3$) of **A2-29**.

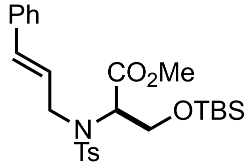

A2-29

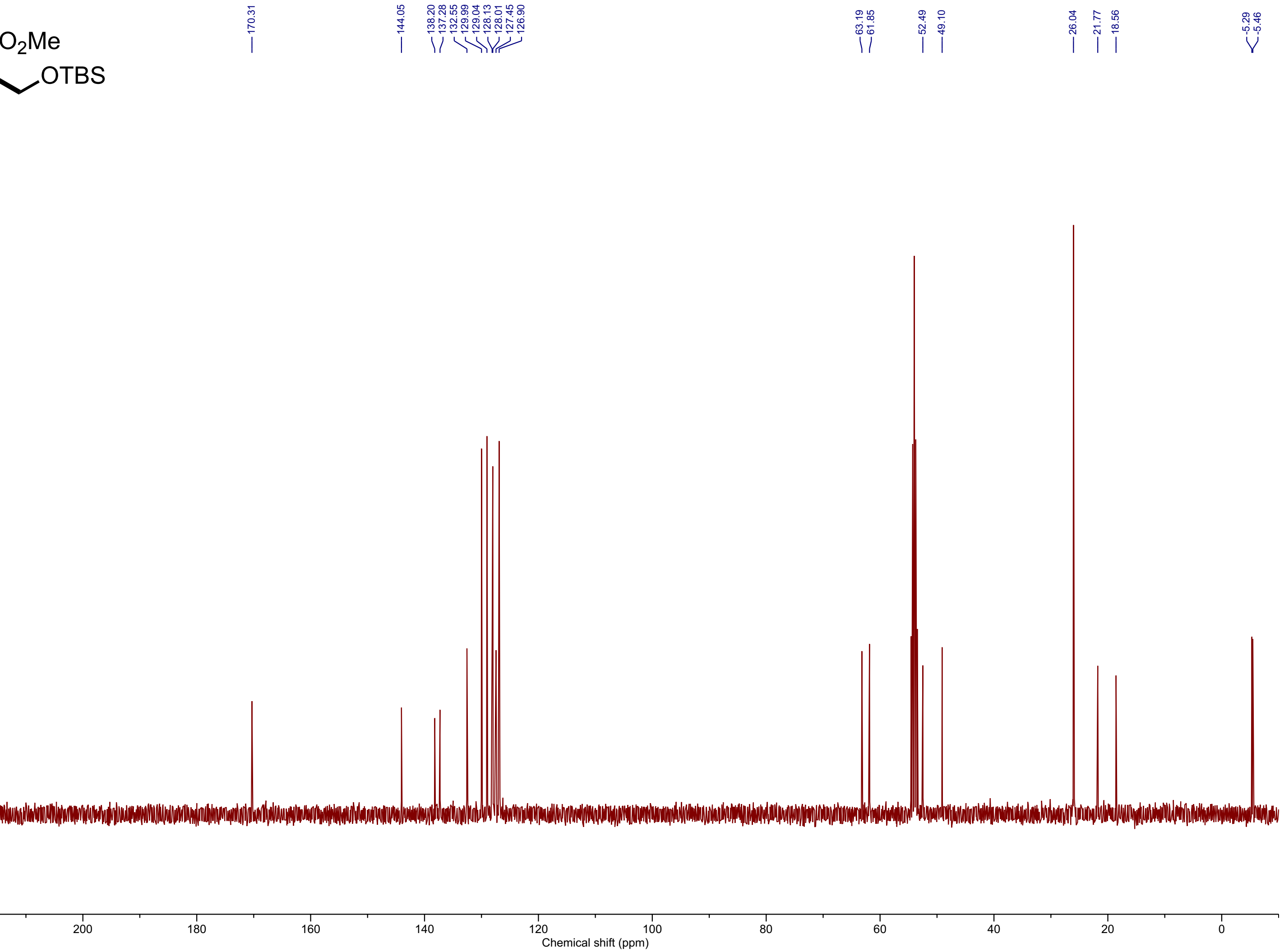

Supplementary Figure 59. ^{13}C NMR (100 MHz, CD_2Cl_2) of **A2-29**.

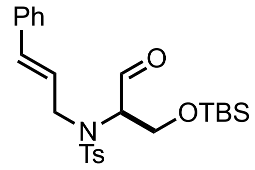

A3-29

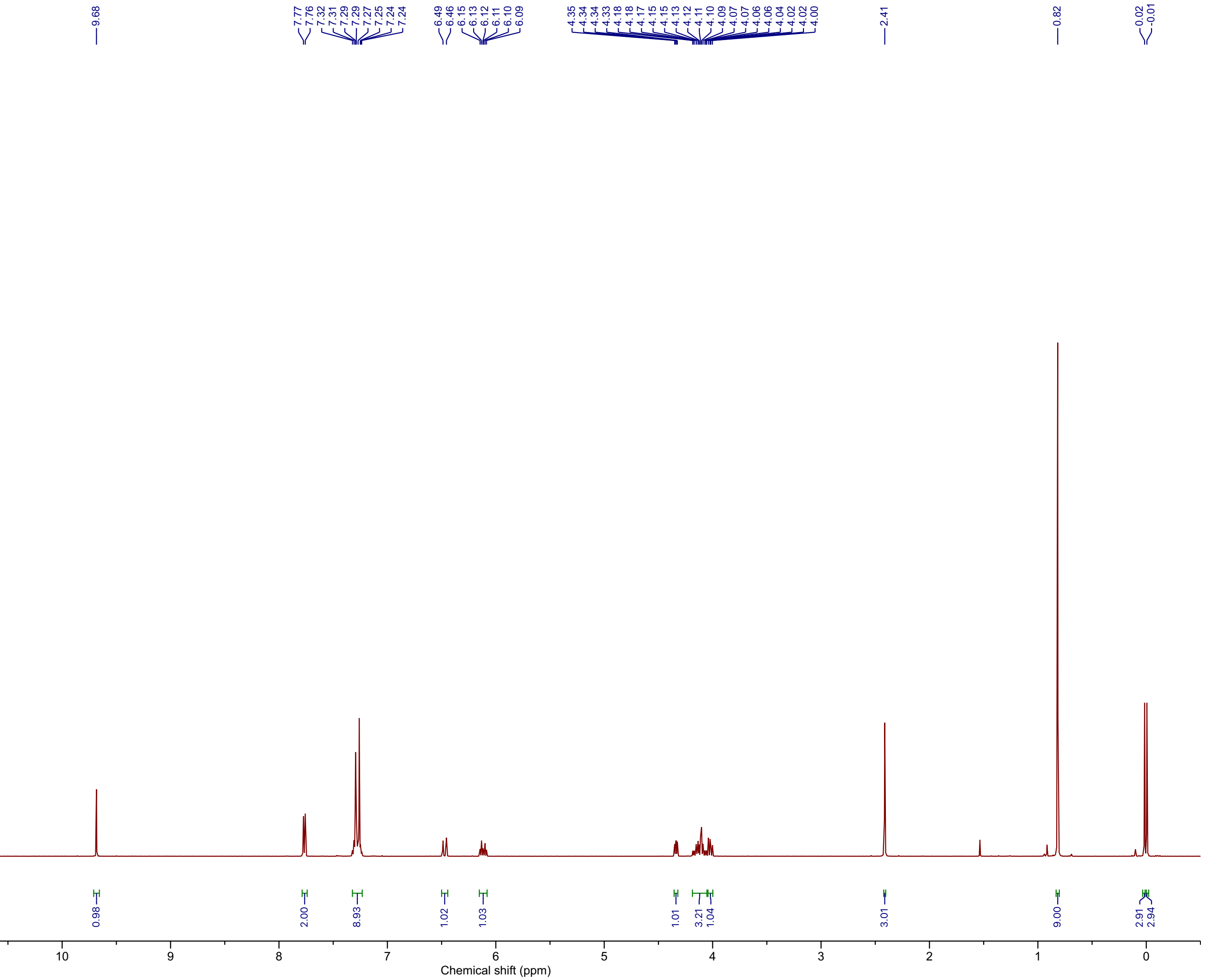

Supplementary Figure 60. ^{1}H NMR (500 MHz, $CDCl_3$) of **A3-29**.

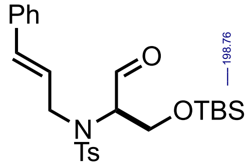

A3-29

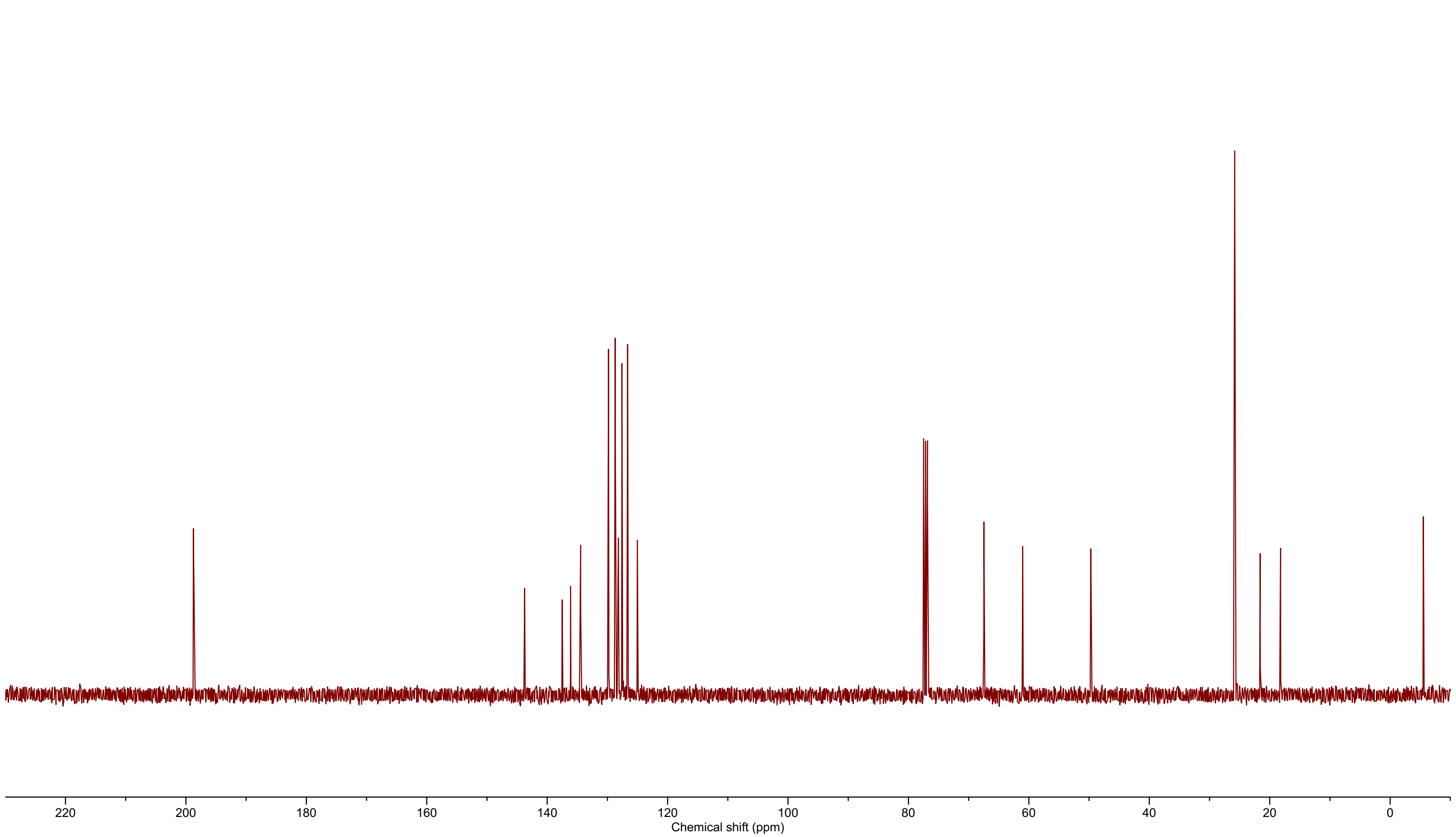

Supplementary Figure 61. ^{13}C NMR (100 MHz, $CDCl_3$) of **A3-29**.

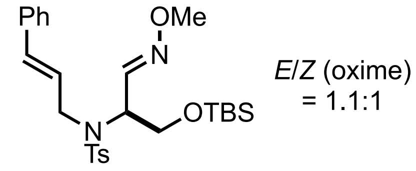

S29

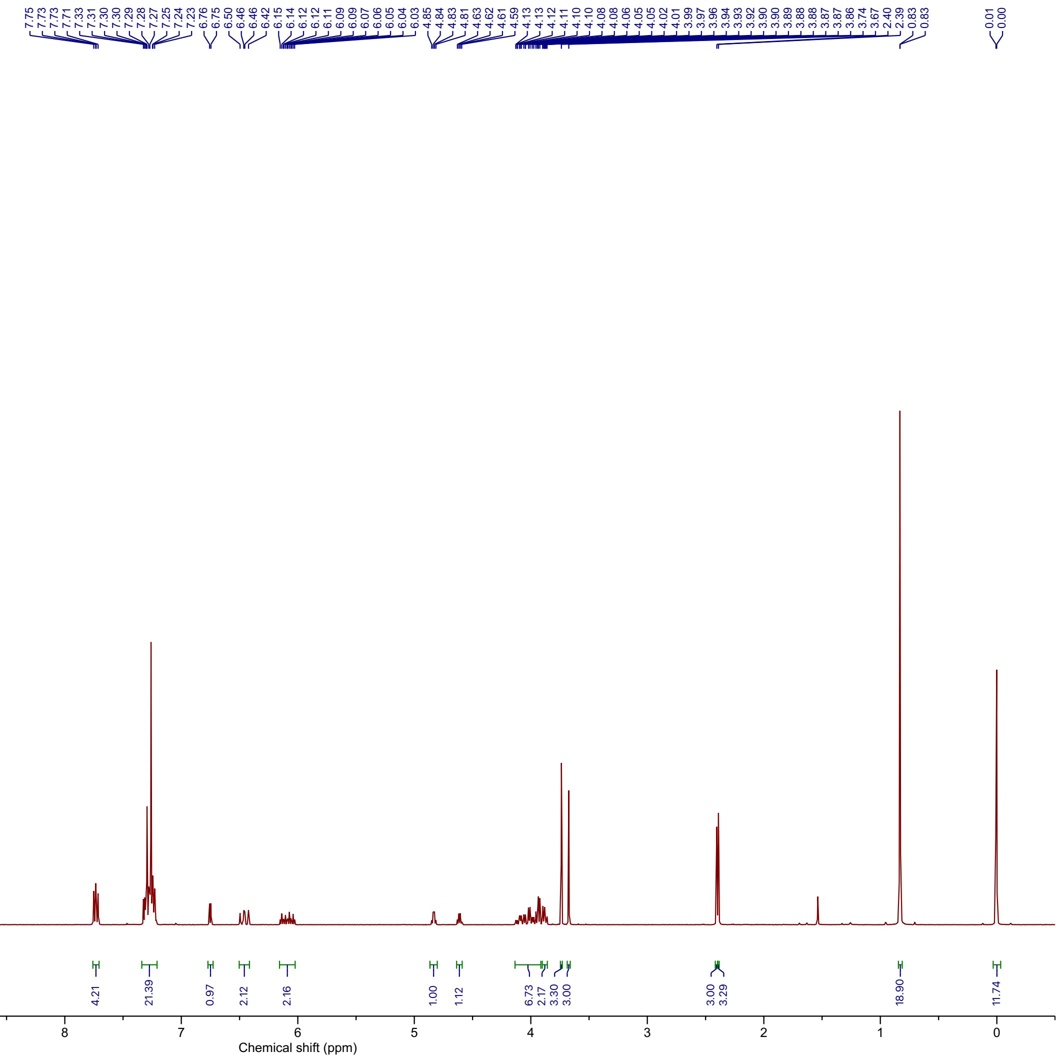

Supplementary Figure 62. ^{1}H NMR (500 MHz, $CDCl_3$) of **S29**.

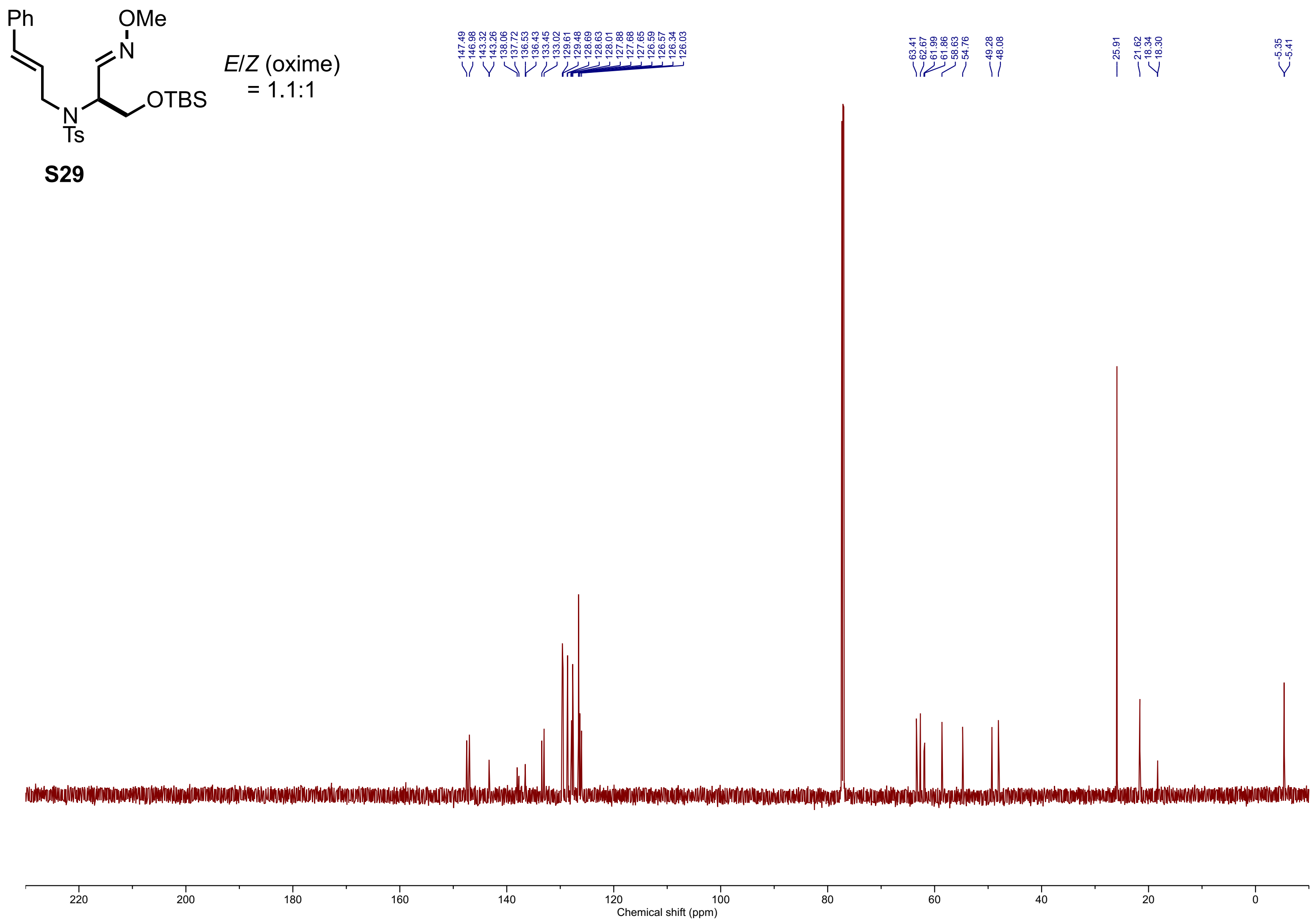

Supplementary Figure 63. ^{13}C NMR (176 MHz, $CDCl_3$) of **S29**.

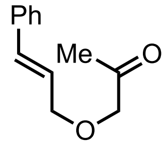

A30

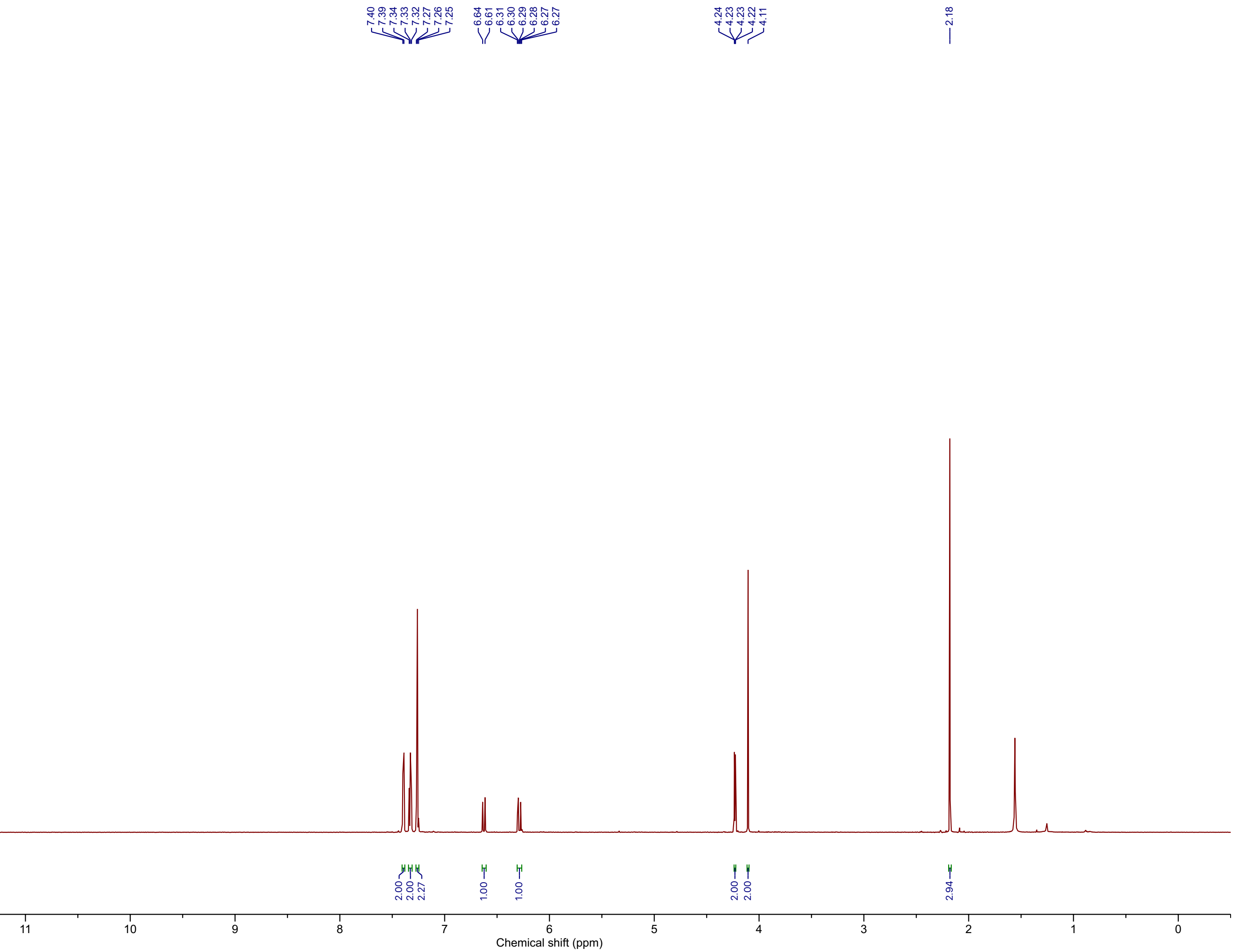

Supplementary Figure 64. ^{1}H NMR (700 MHz, $CDCl_3$) of **A30**.

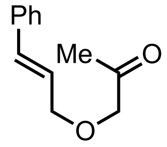

A30

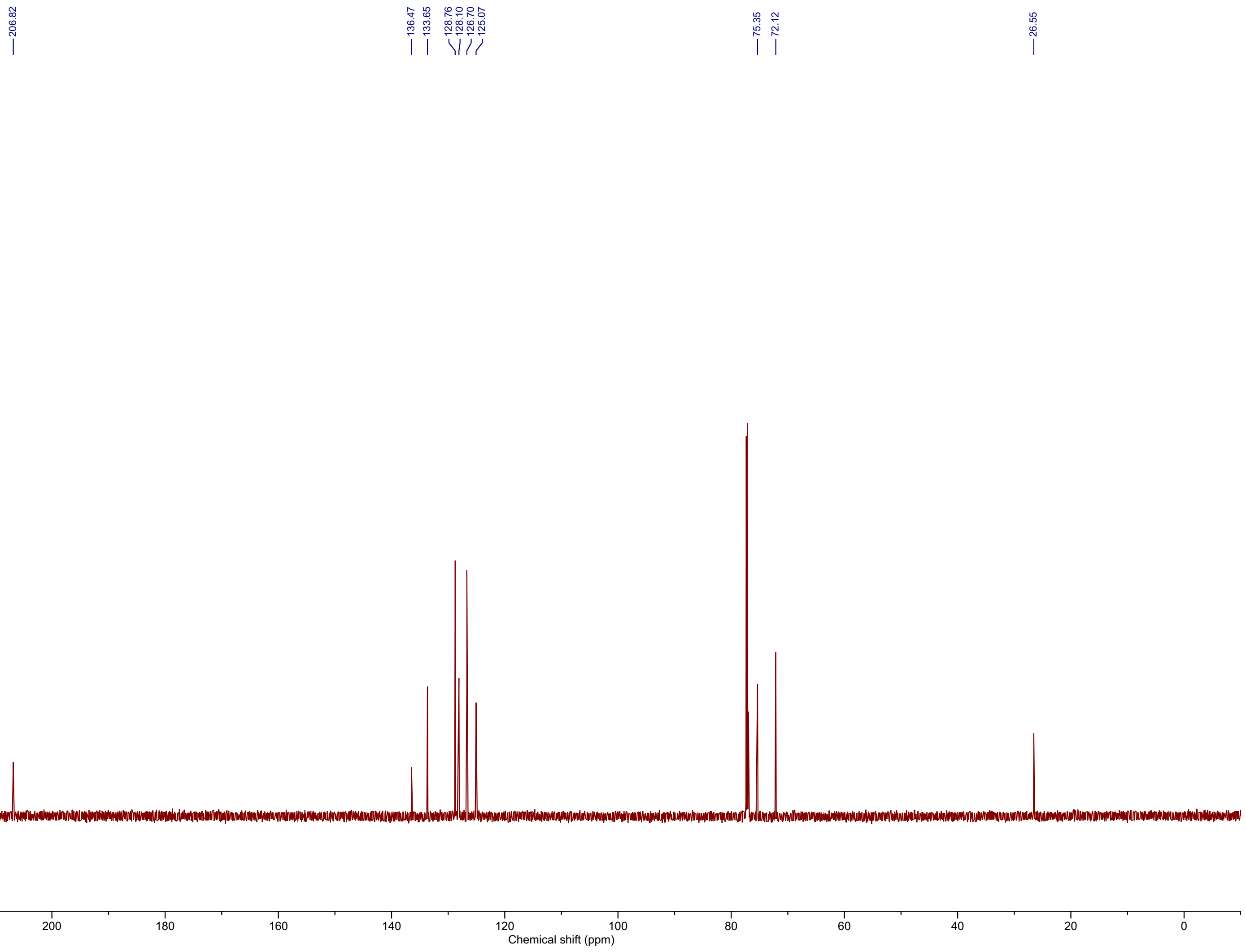

Supplementary Figure 65. ^{13}C NMR (176 MHz, $CDCl_3$) of **A30**.

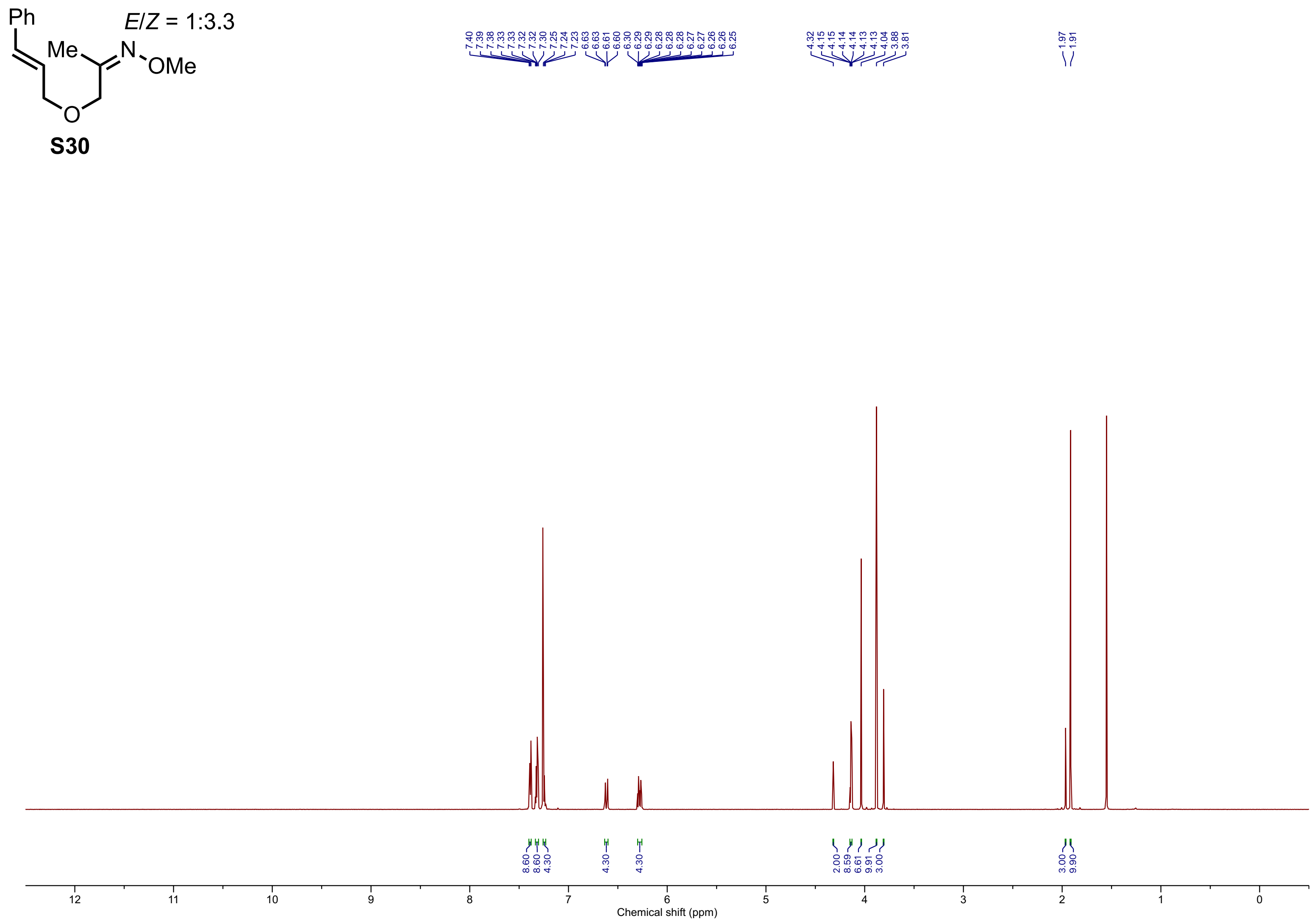

Supplementary Figure 66. ^{1}H NMR (700 MHz, $CDCl_3$) of **S30**.

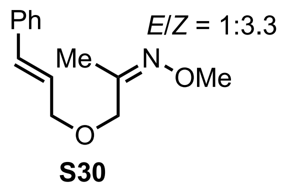

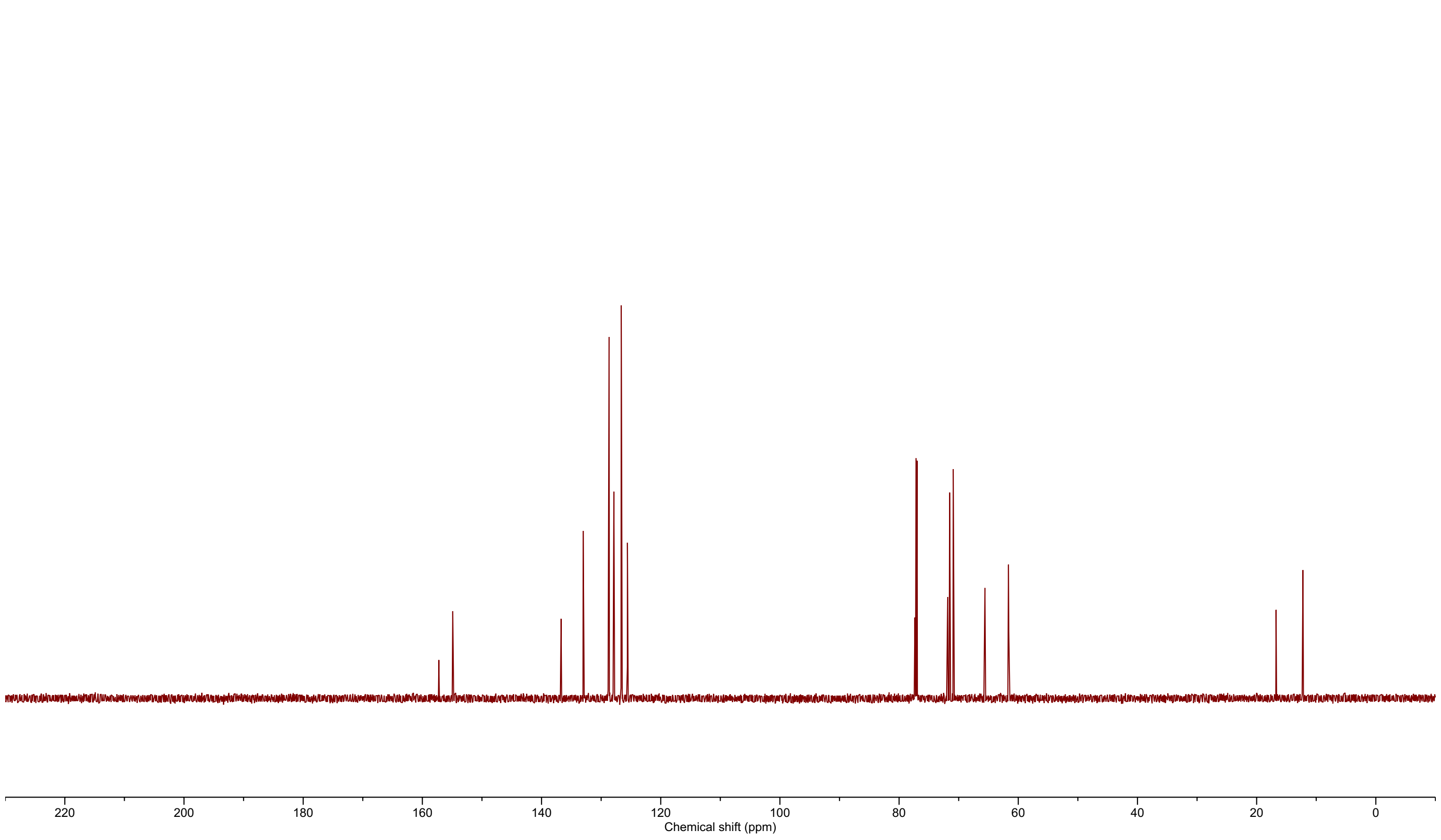

Supplementary Figure 67. ^{13}C NMR (176 MHz, $CDCl_3$) of **S30**.

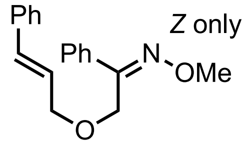

S31

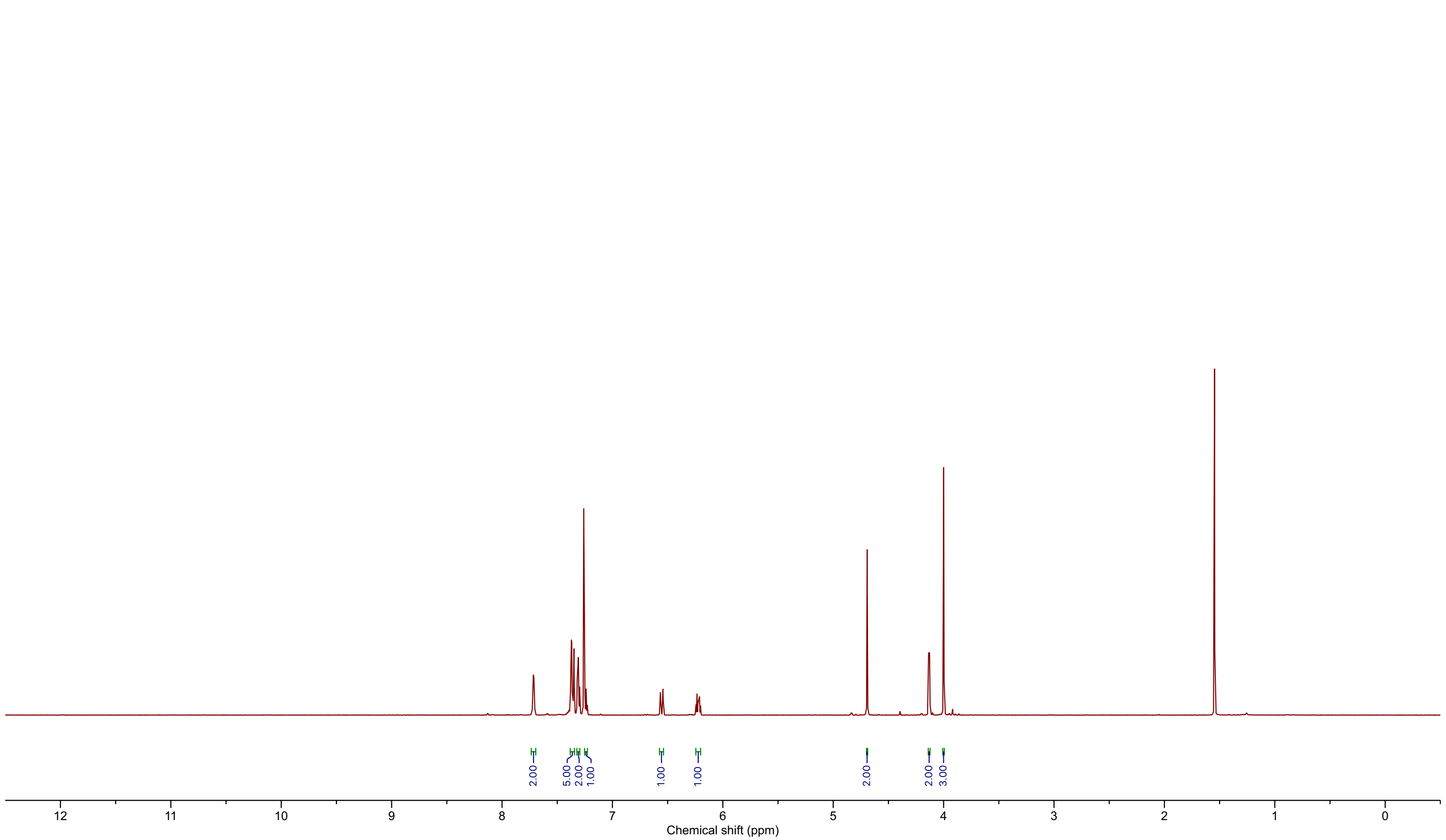

Supplementary Figure 68. ^{1}H NMR (700 MHz, $CDCl_3$) of **S31**.

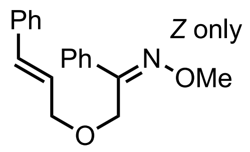

S31

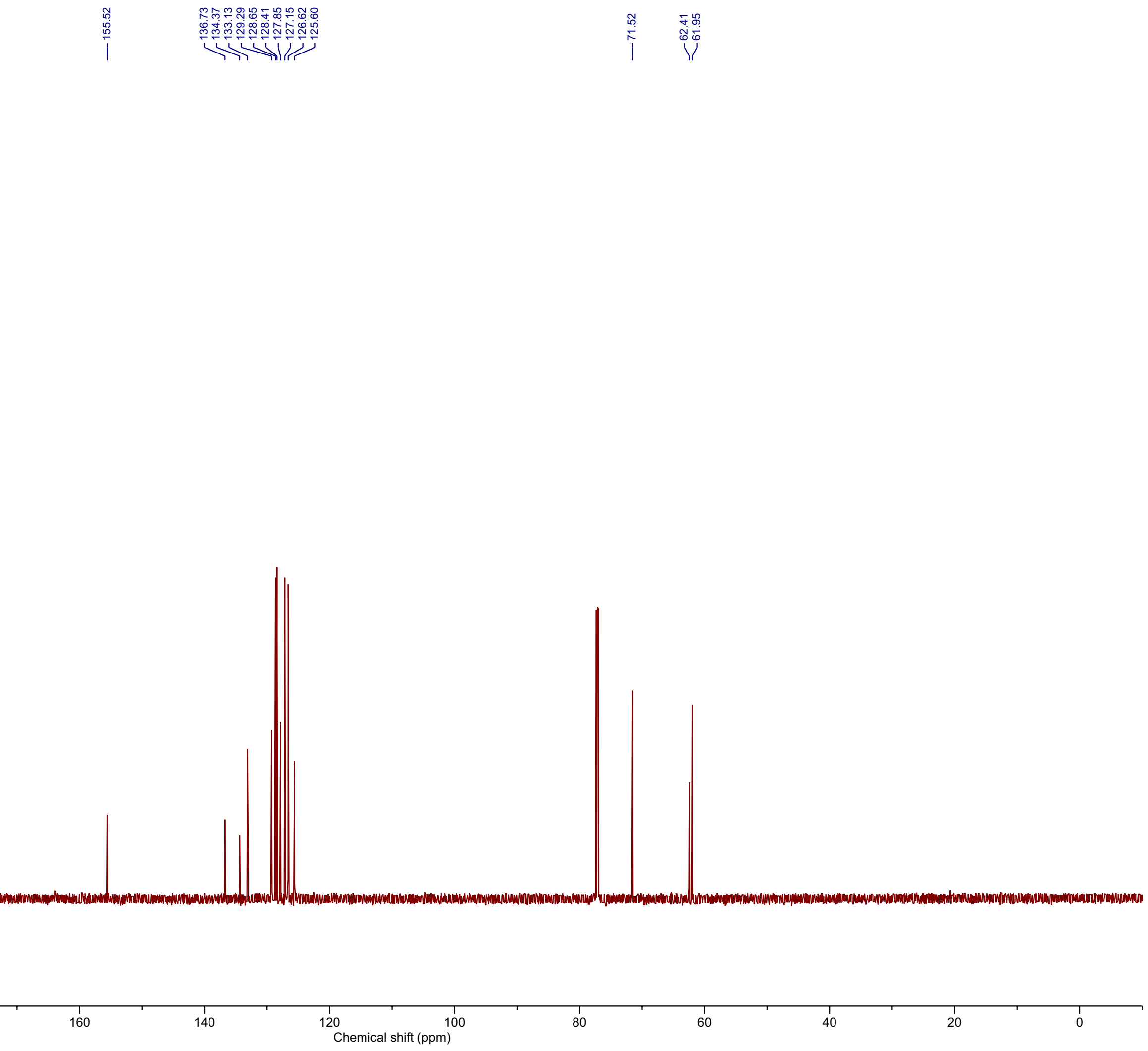

Supplementary Figure 69. ^{13}C NMR (176 MHz, $CDCl_3$) of **S31**.

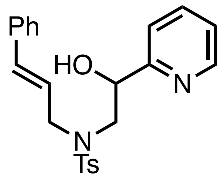

A32

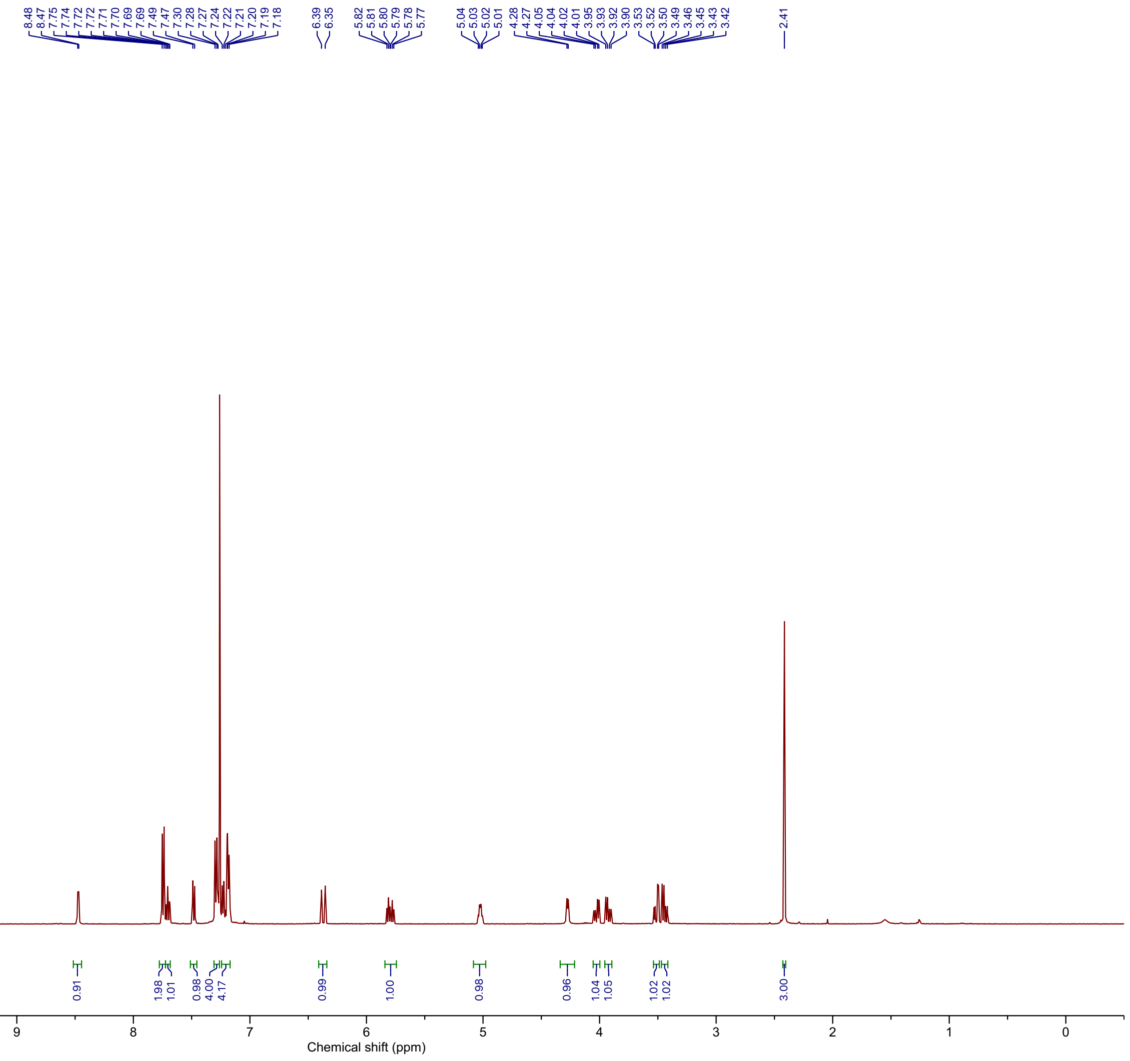

Supplementary Figure 70. ^{1}H NMR (500 MHz, $CDCl_3$) of **A32**.

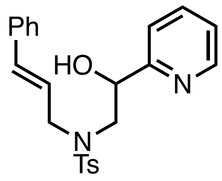

A32

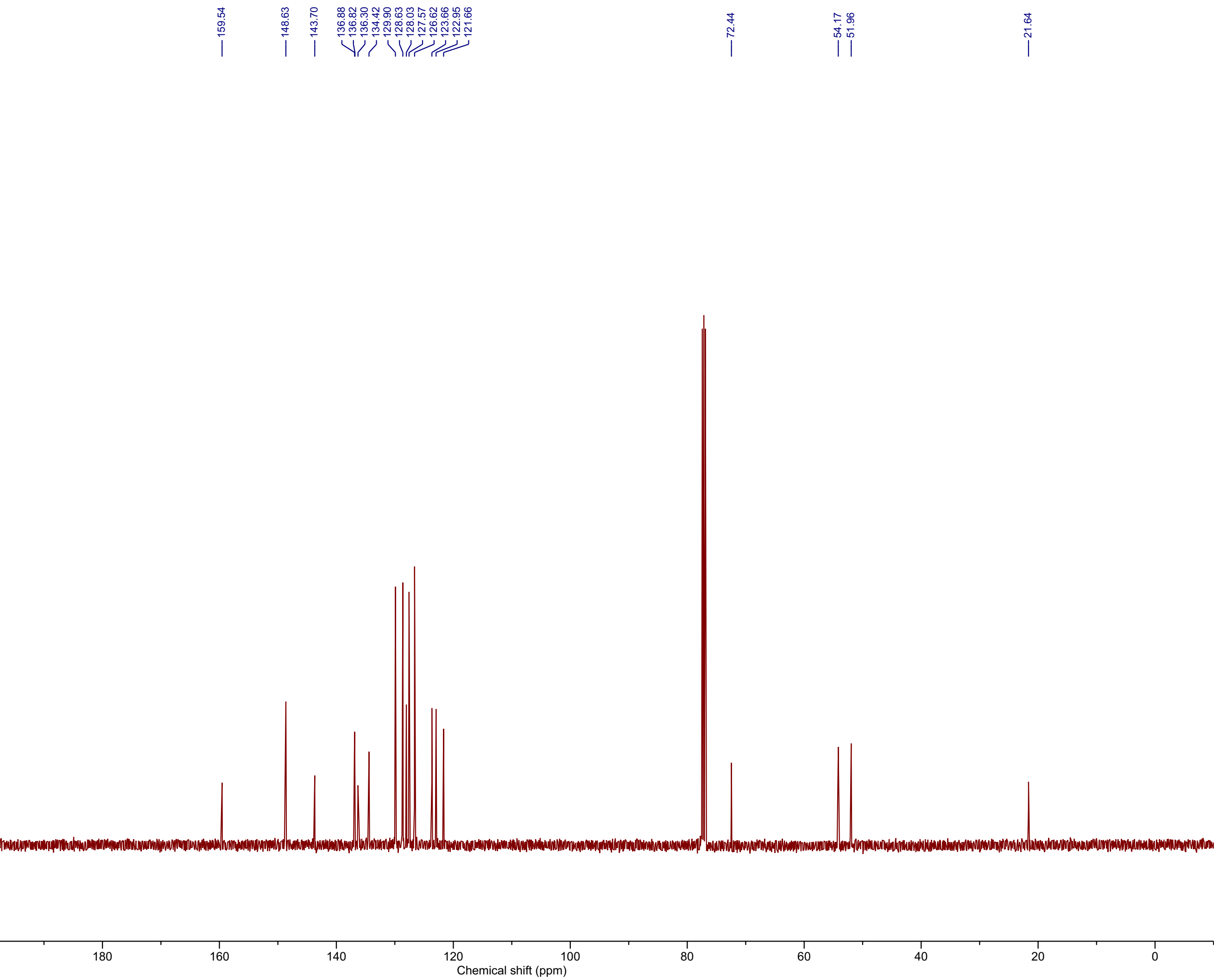

Supplementary Figure 71. ^{13}C NMR (126 MHz, $CDCl_3$) of **A32**.

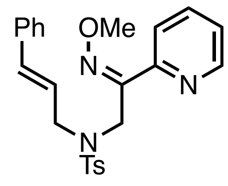

S32

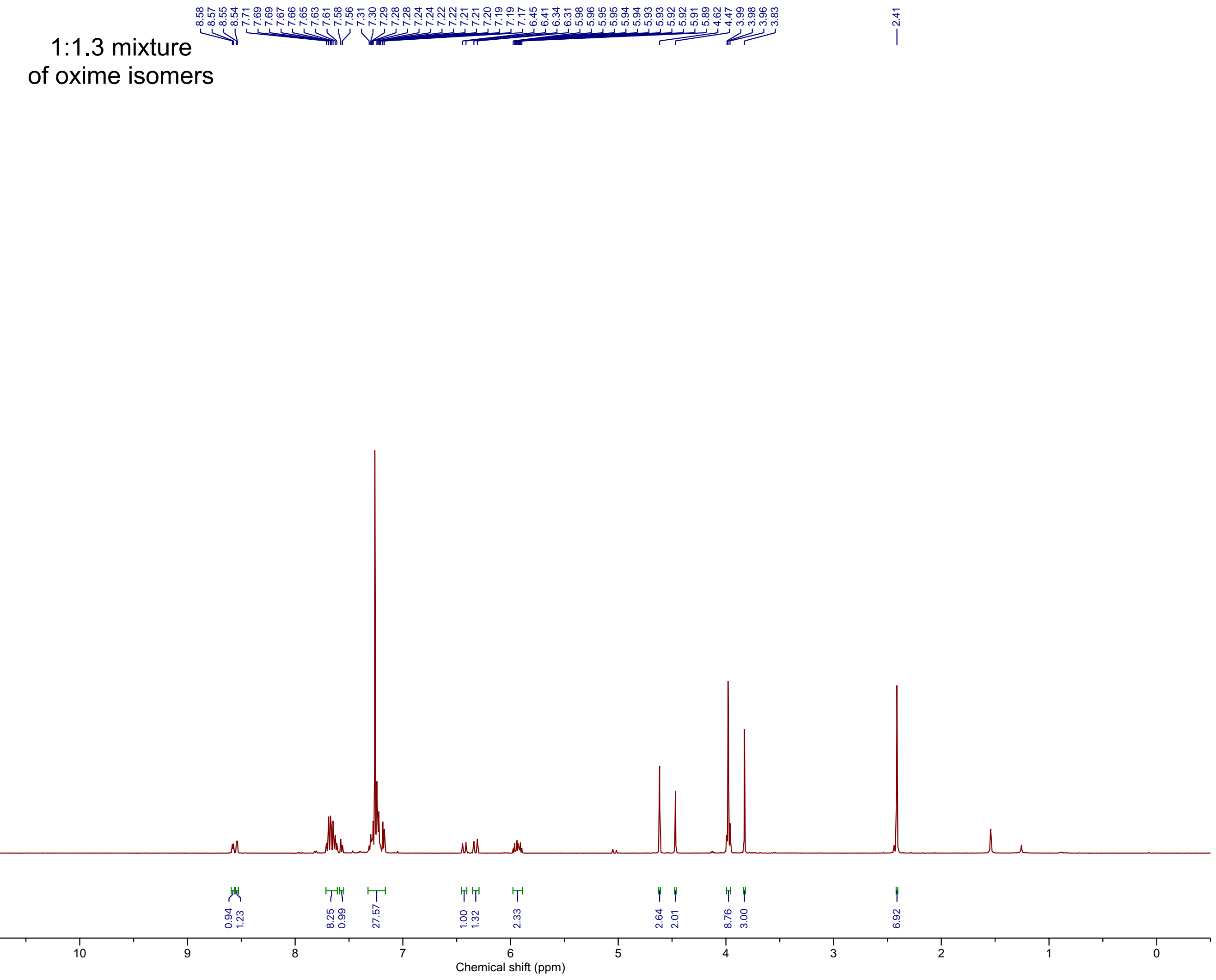

Supplementary Figure 72. ^{1}H NMR (500 MHz, $CDCl_3$) of **S32**.

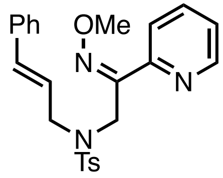

S32

1:1.3 mixture
of oxime isomers

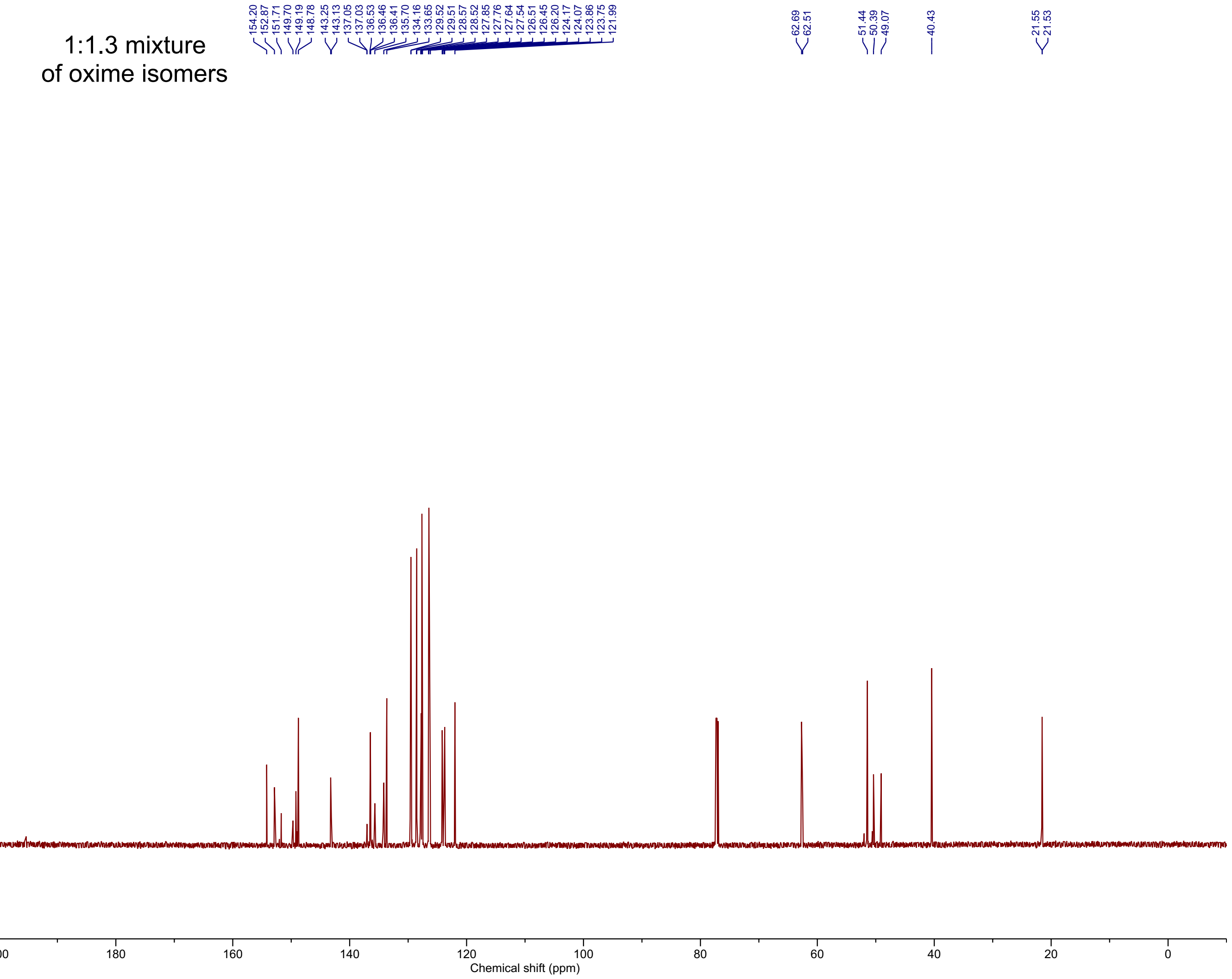

Supplementary Figure 73. ^{13}C NMR (176 MHz, $CDCl_3$) of **S32**.

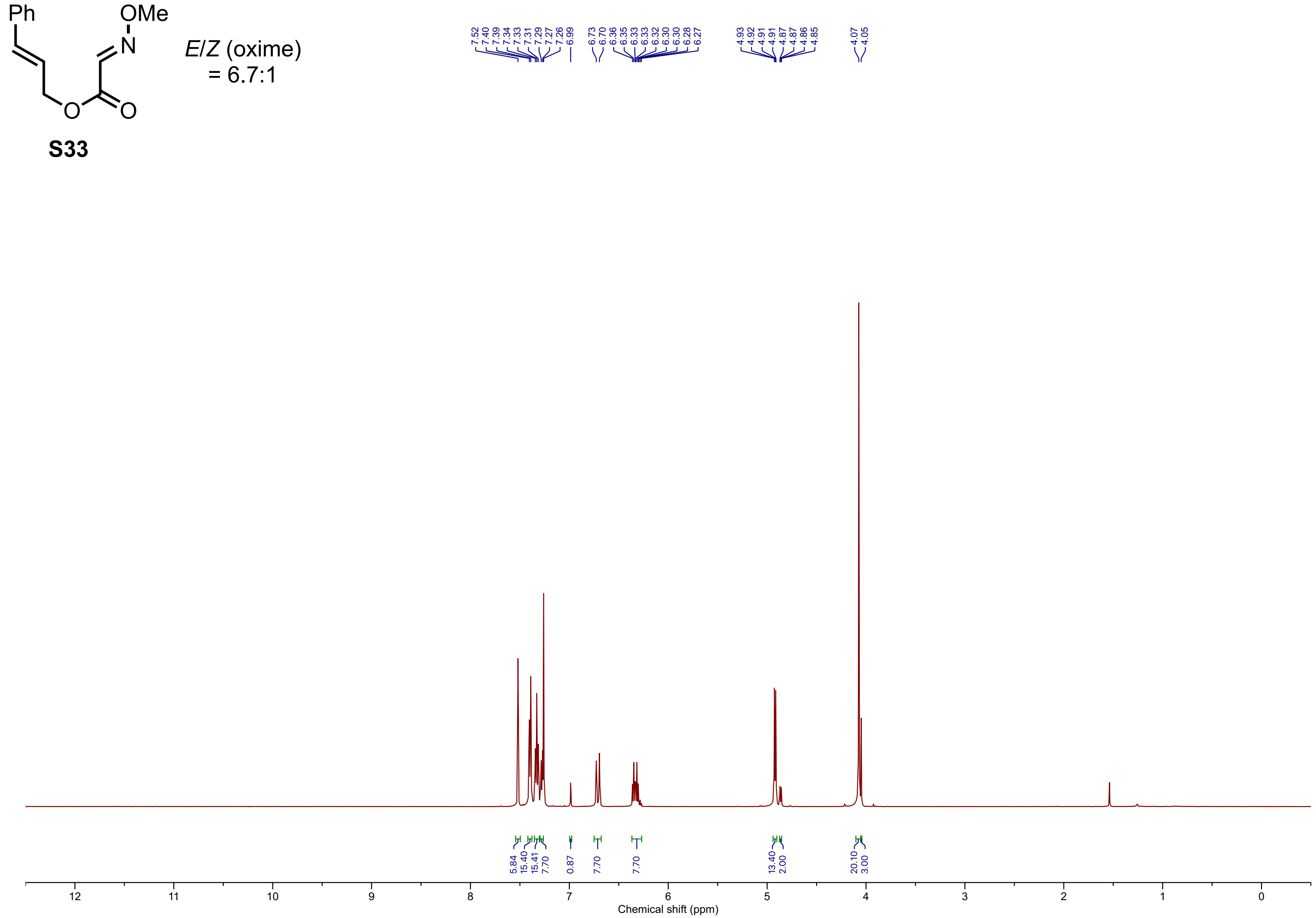

Supplementary Figure 74. ^{1}H NMR (500 MHz, $CDCl_3$) of **S33**.

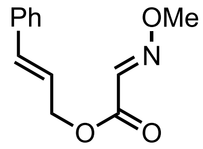

S33

E/*Z* (oxime)
= 6.7:1

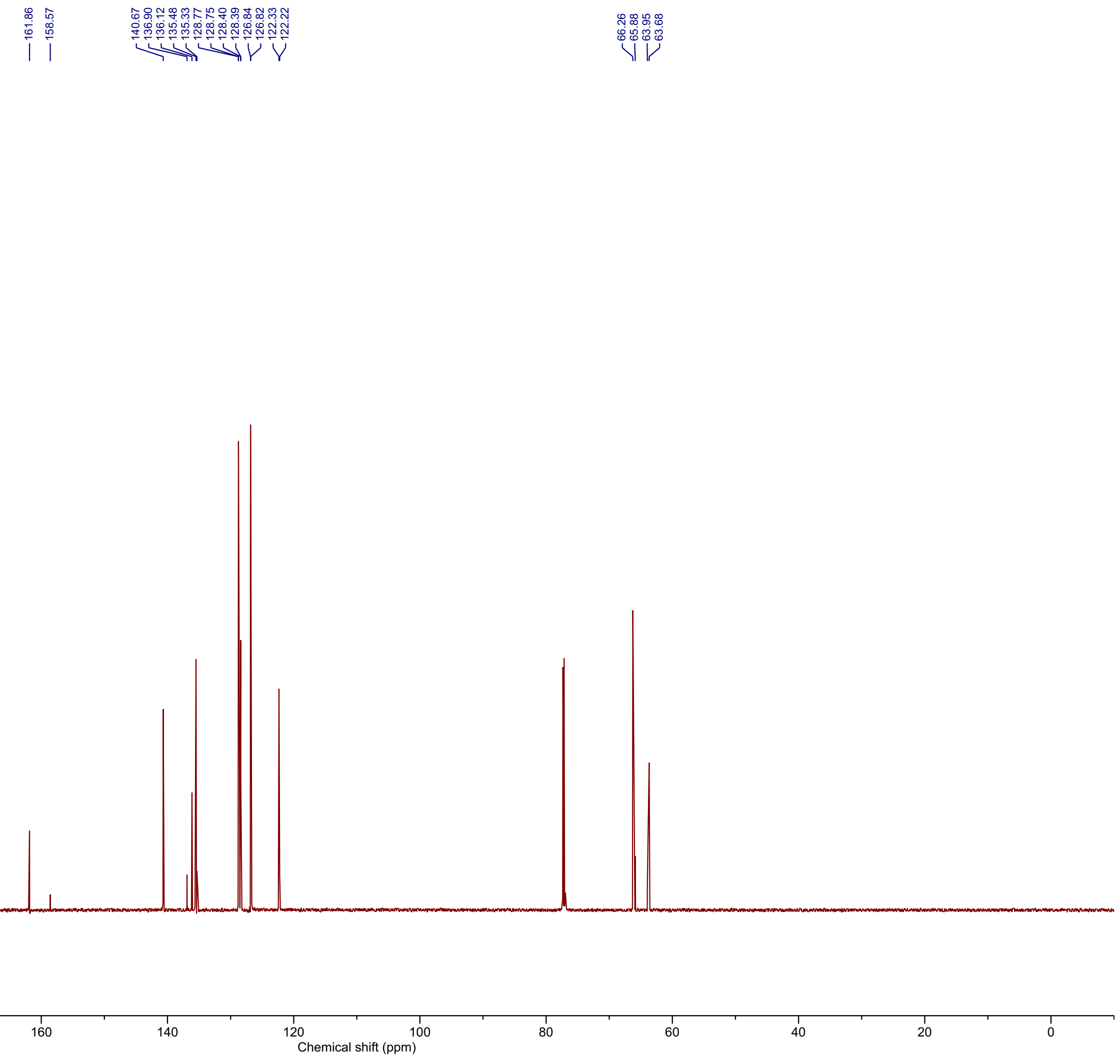

Supplementary Figure 75. ^{13}C NMR (176 MHz, $CDCl_3$) of **S33**.

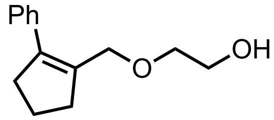

A2-34

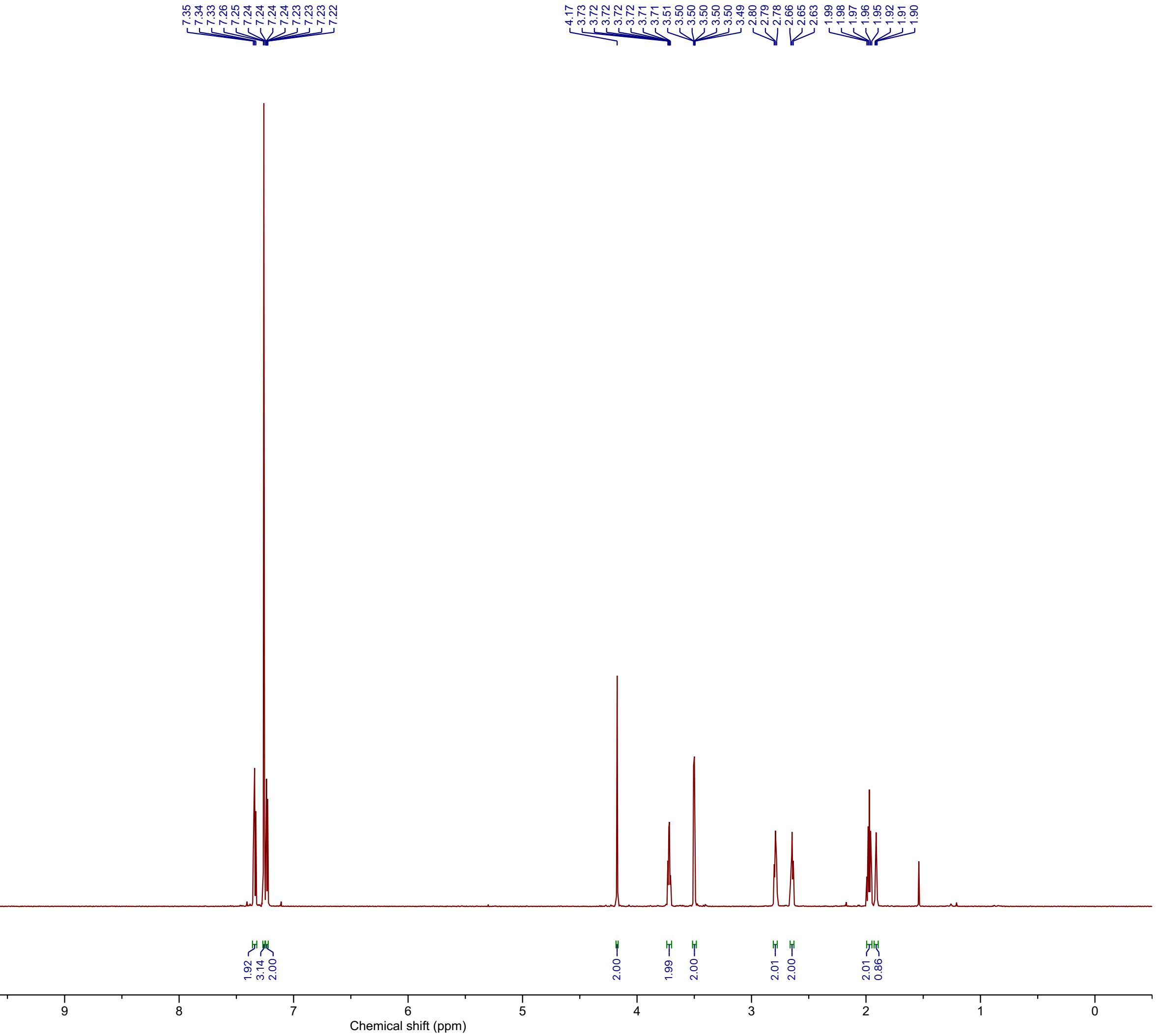

Supplementary Figure 76. ^{1}H NMR (700 MHz, $CDCl_3$) of **A2-34**.

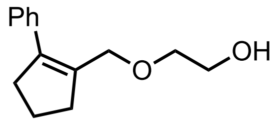

A2-34

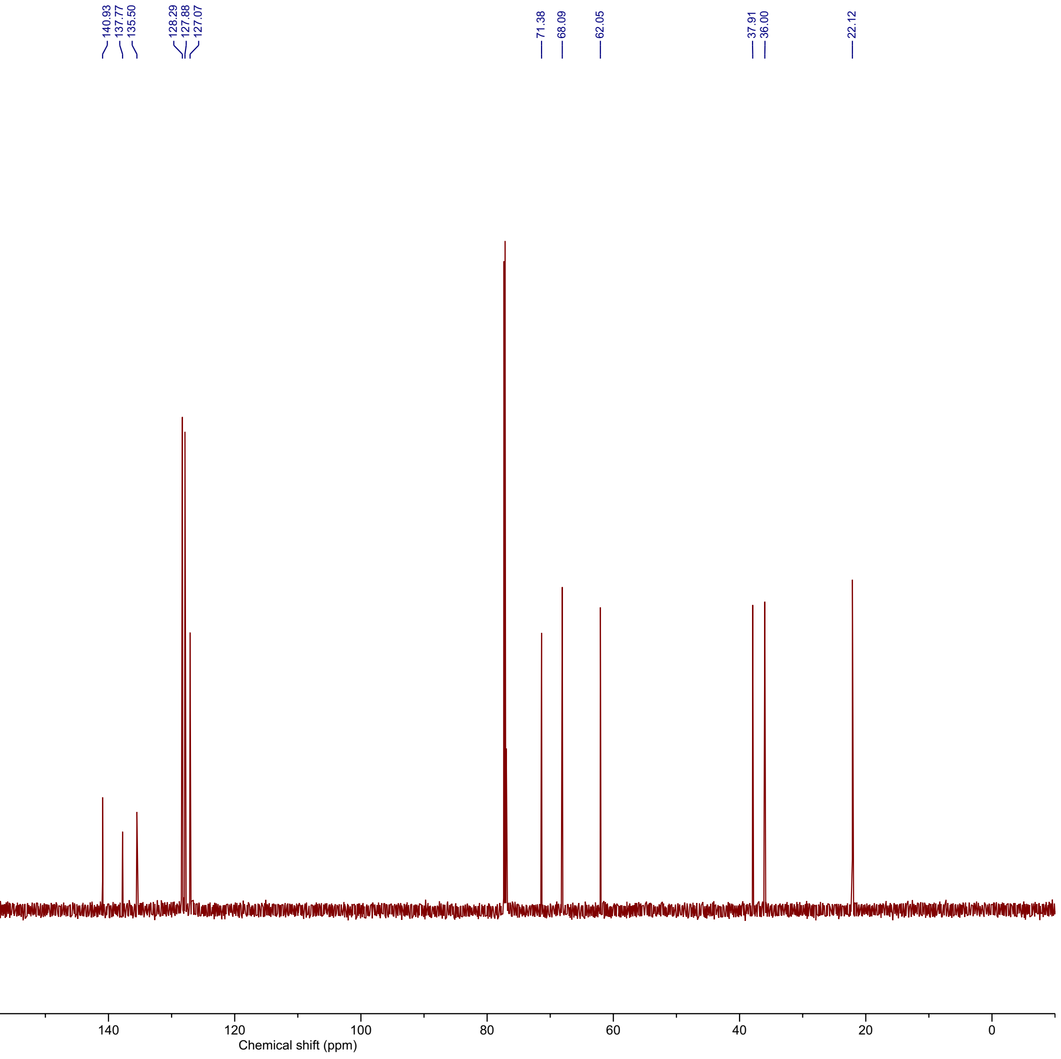

Supplementary Figure 77. ^{13}C NMR (176 MHz, $CDCl_3$) of **A2-34**.

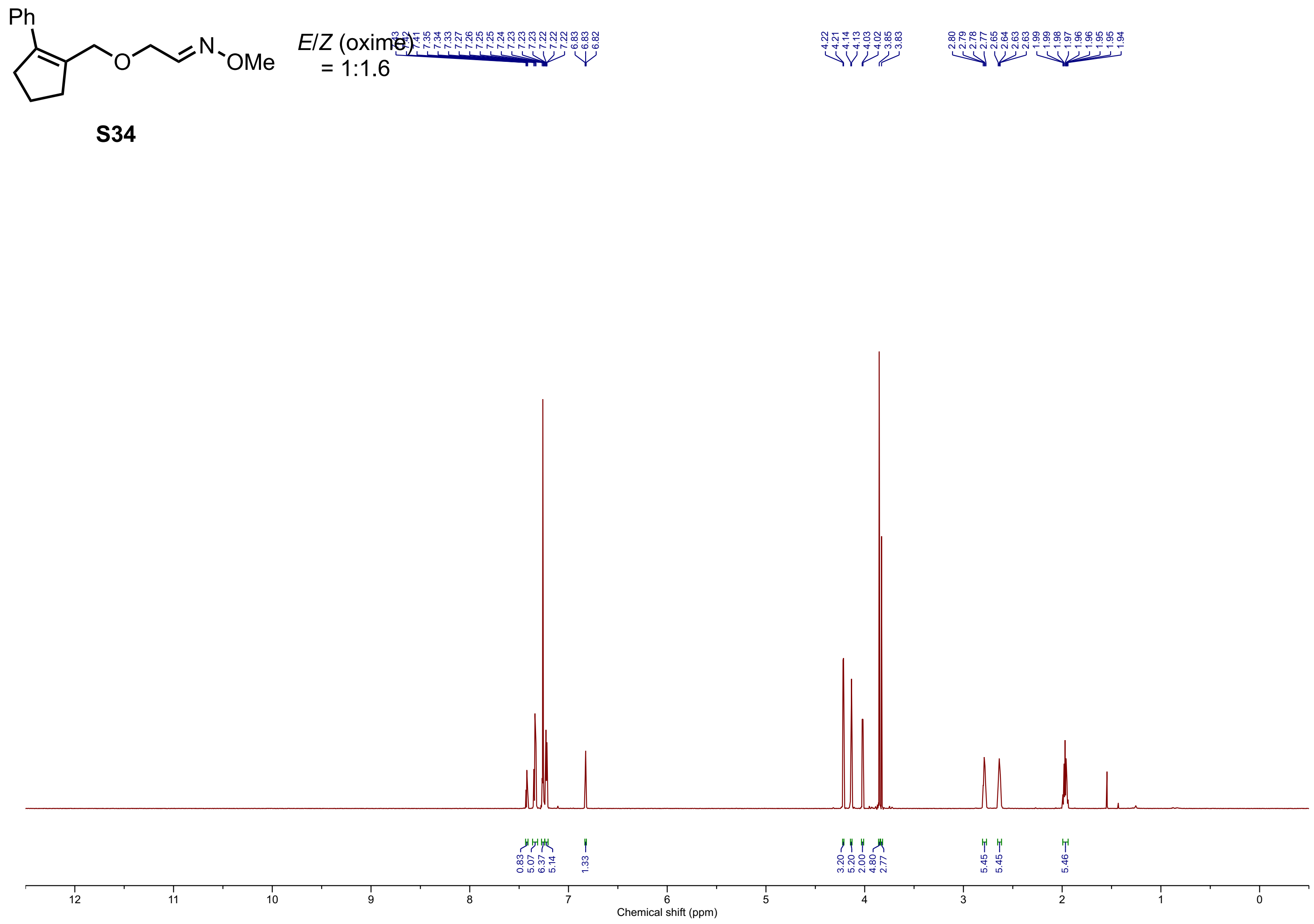

S34

Supplementary Figure 78. ^{1}H NMR (700 MHz, $CDCl_3$) of **S34**.

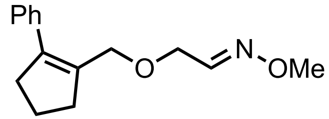

S34

E/*Z* (oxime) = 1:1.6

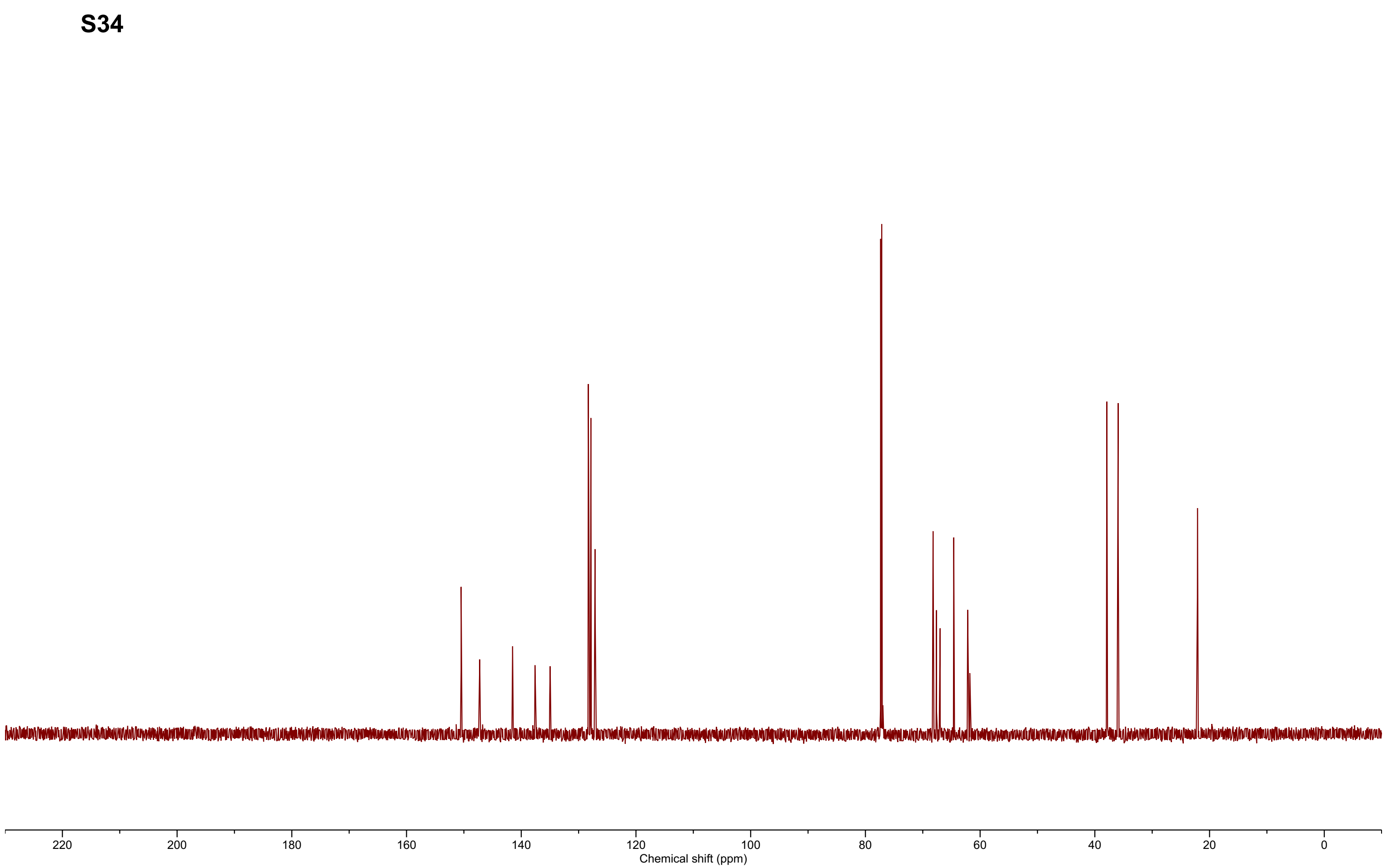

Supplementary Figure 79. ^{13}C NMR (176 MHz, $CDCl_3$) of **S34**.

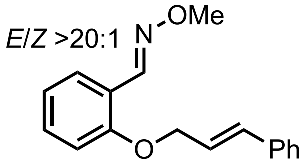

S35

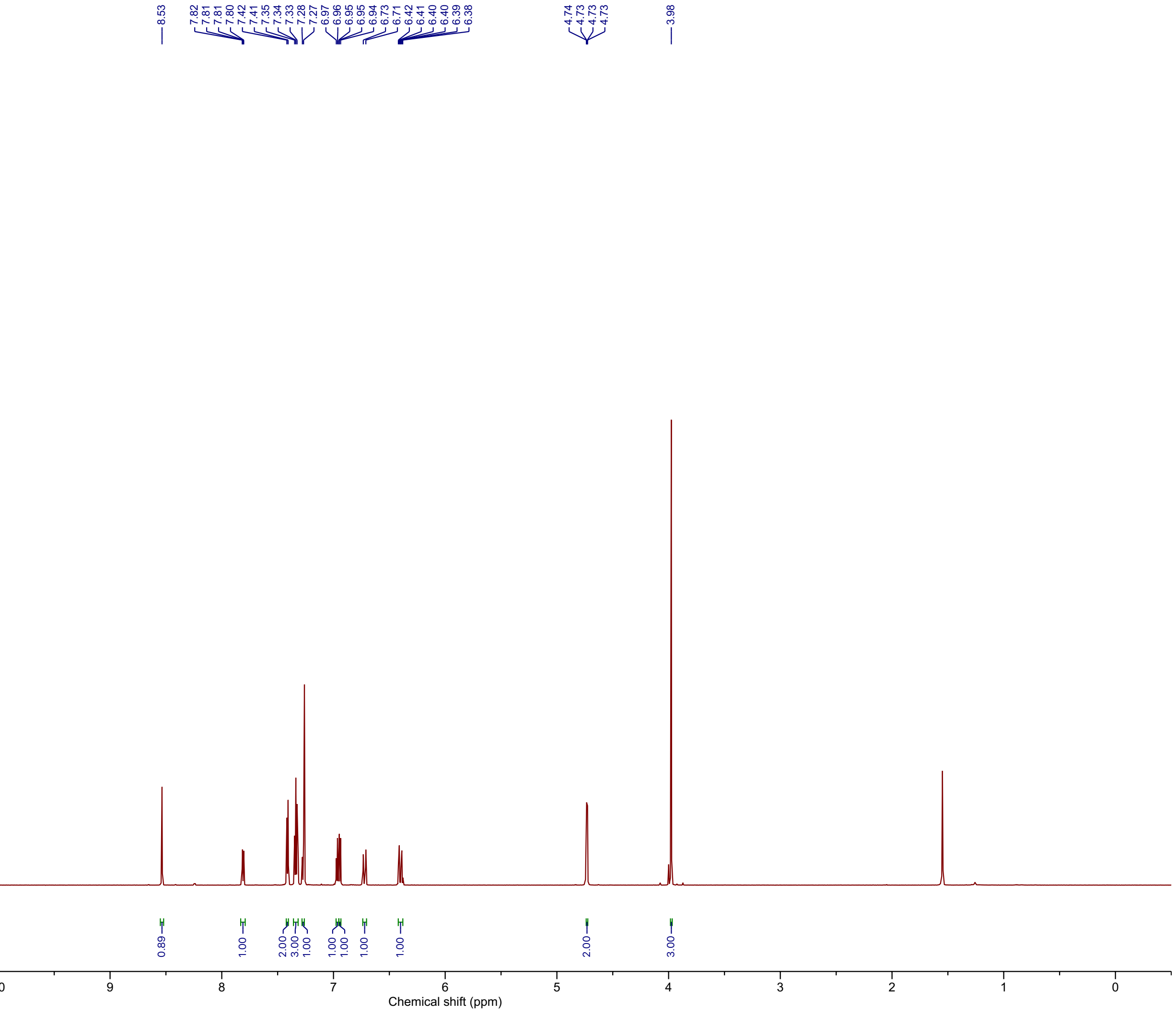

Supplementary Figure 80. ^{1}H NMR (700 MHz, $CDCl_3$) of **S35**.

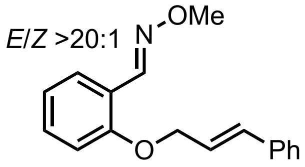

S35

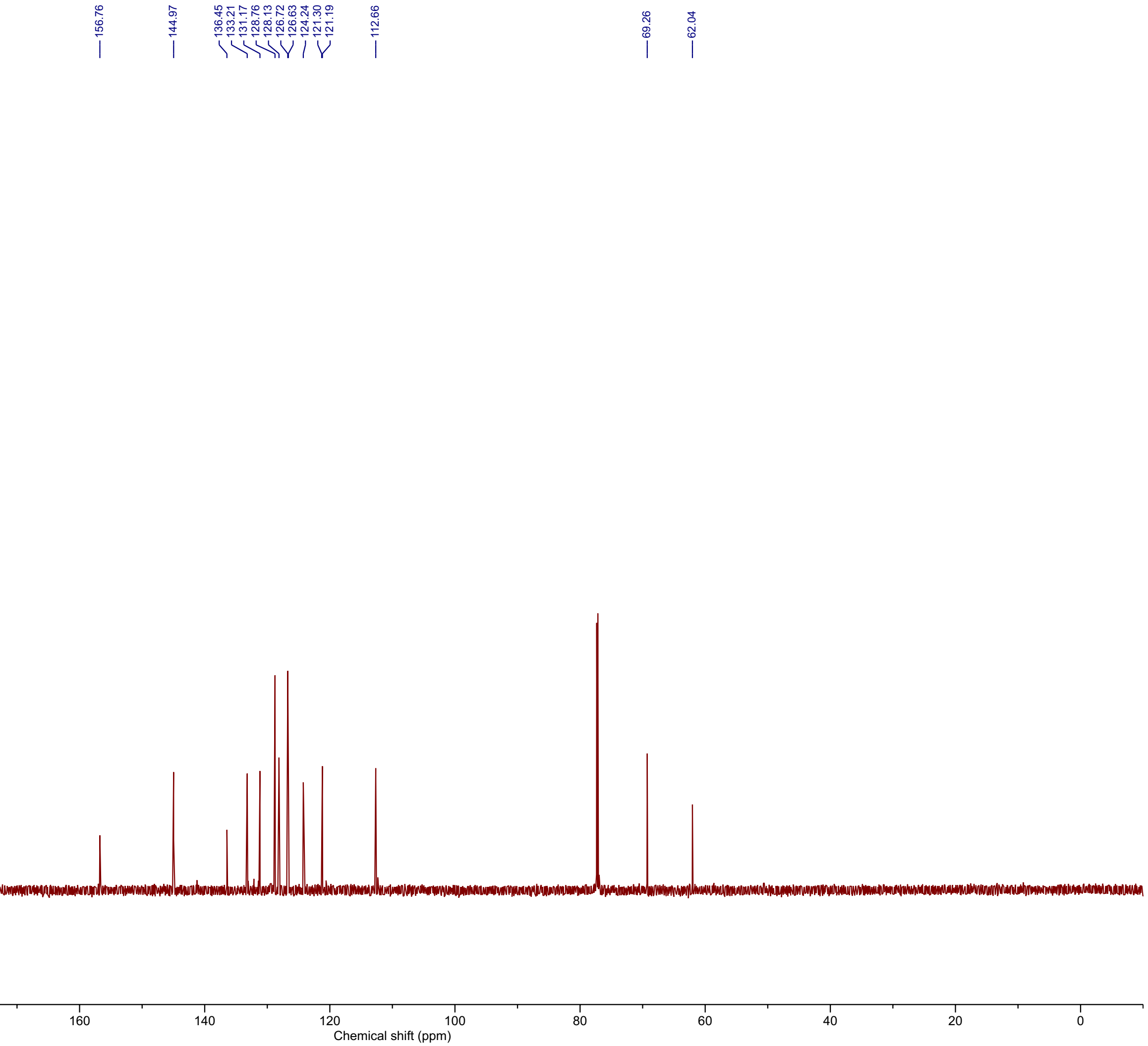

Supplementary Figure 81. ^{13}C NMR (176 MHz, $CDCl_3$) of **S35**.

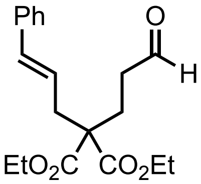

A36

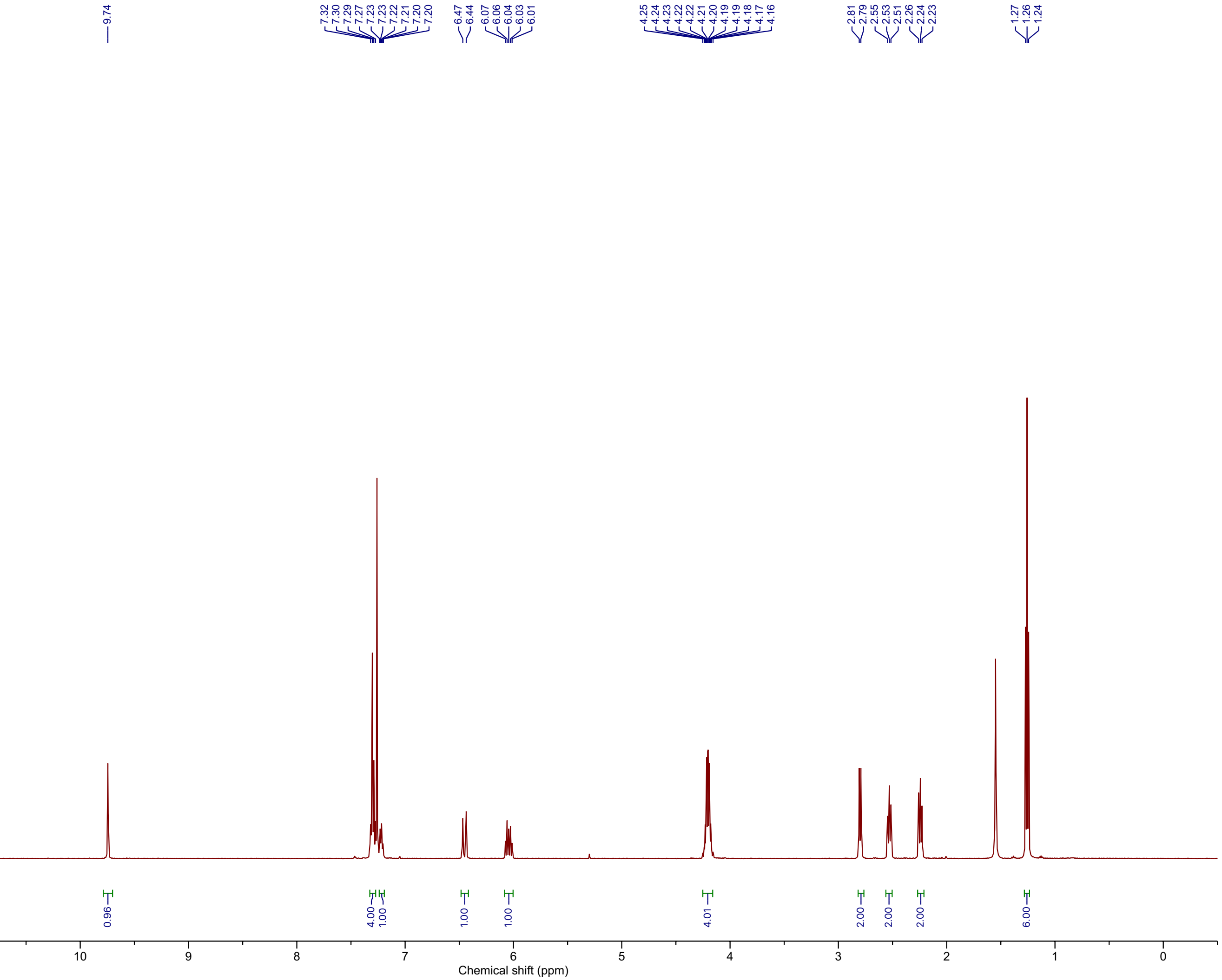

Supplementary Figure 82. ^{1}H NMR (500 MHz, $CDCl_3$) of **A36**.

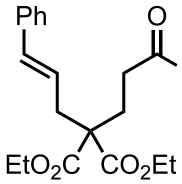

A36

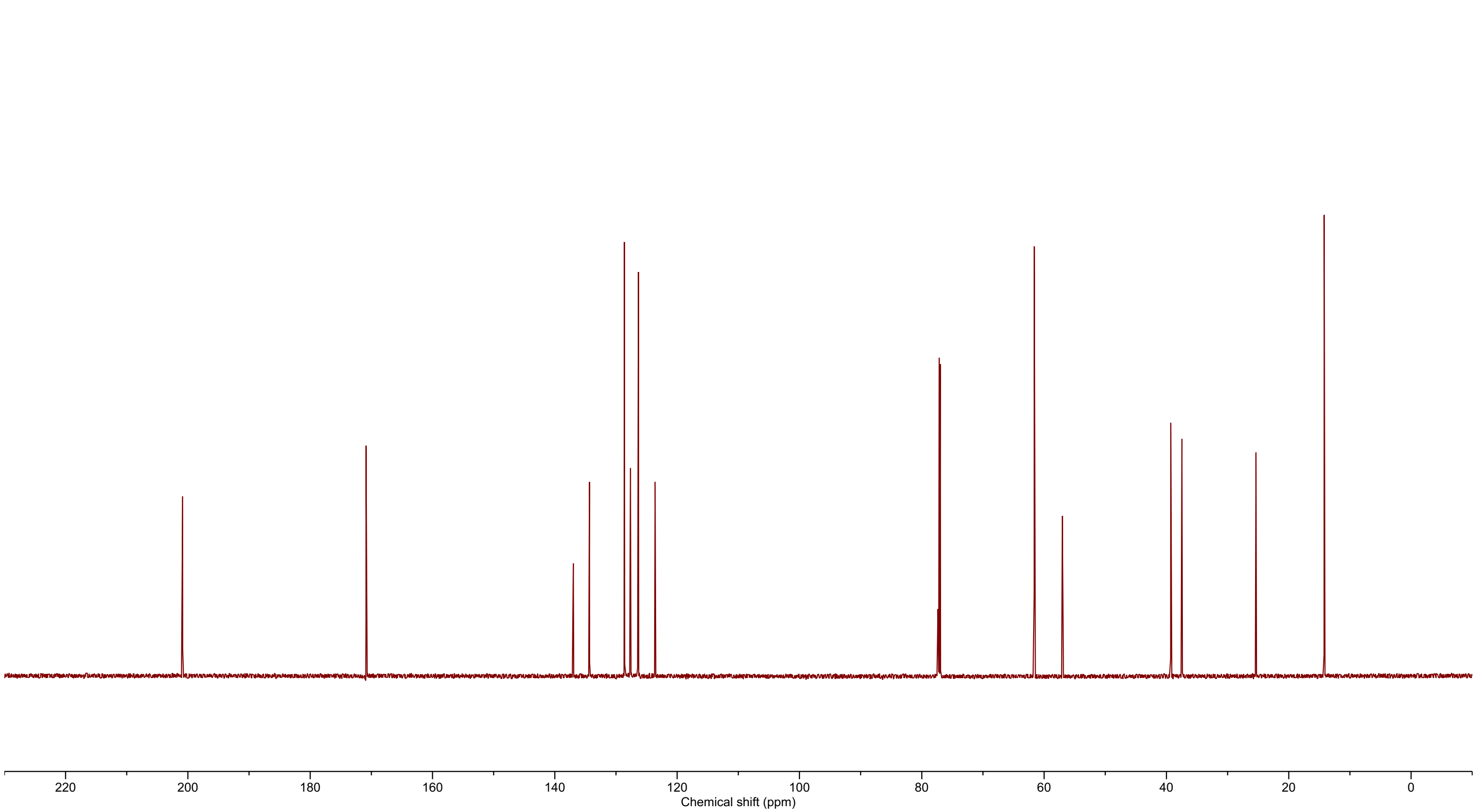

Supplementary Figure 83. ^{13}C NMR (176 MHz, $CDCl_3$) of **A36**.

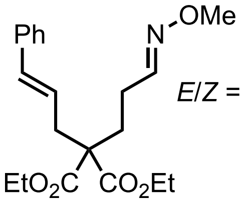

E/*Z* = 1.2:1

S36

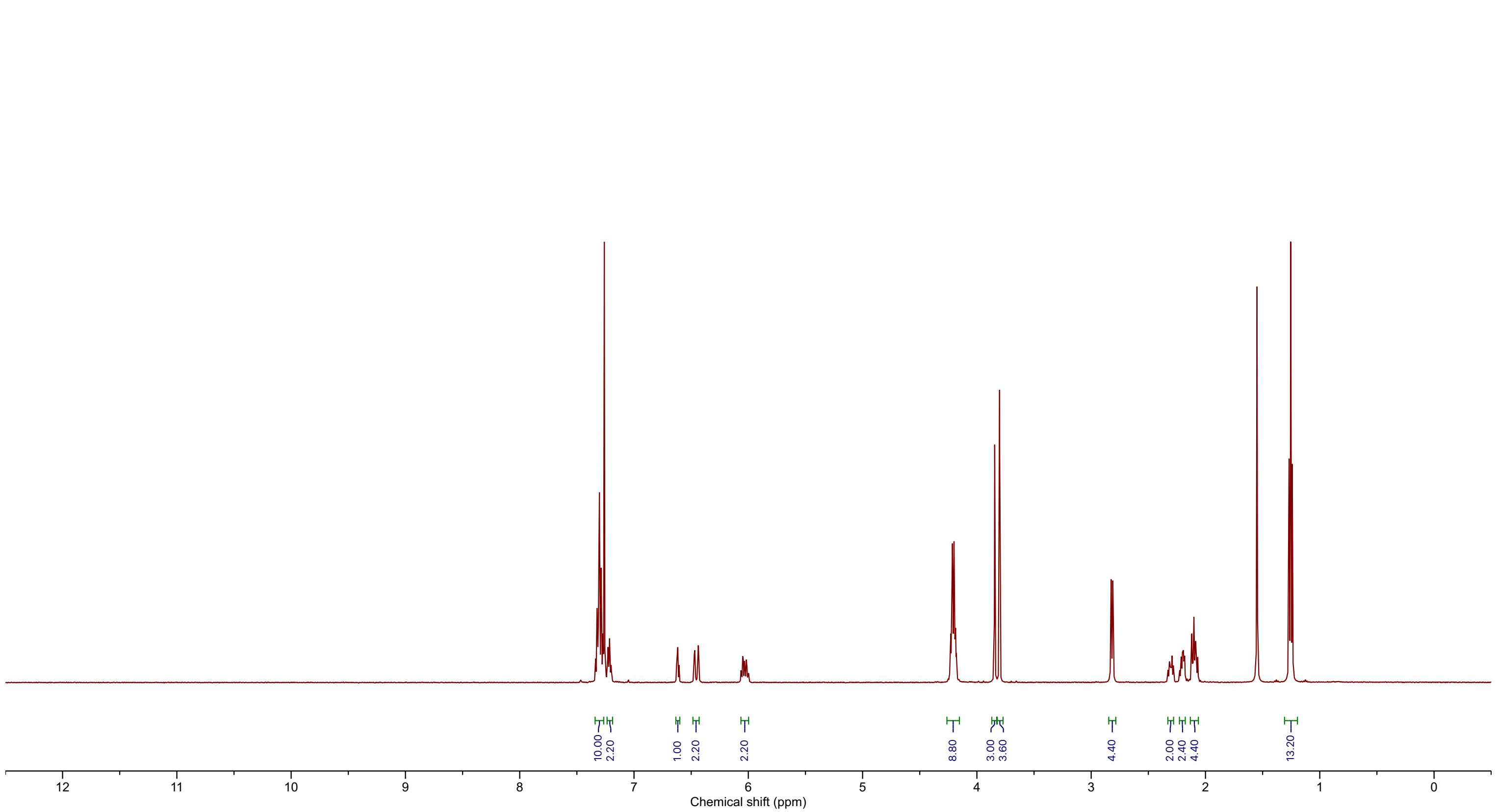

Supplementary Figure 84. ^{1}H NMR (500 MHz, $CDCl_3$) of **S36**.

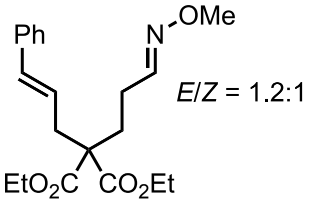

S36

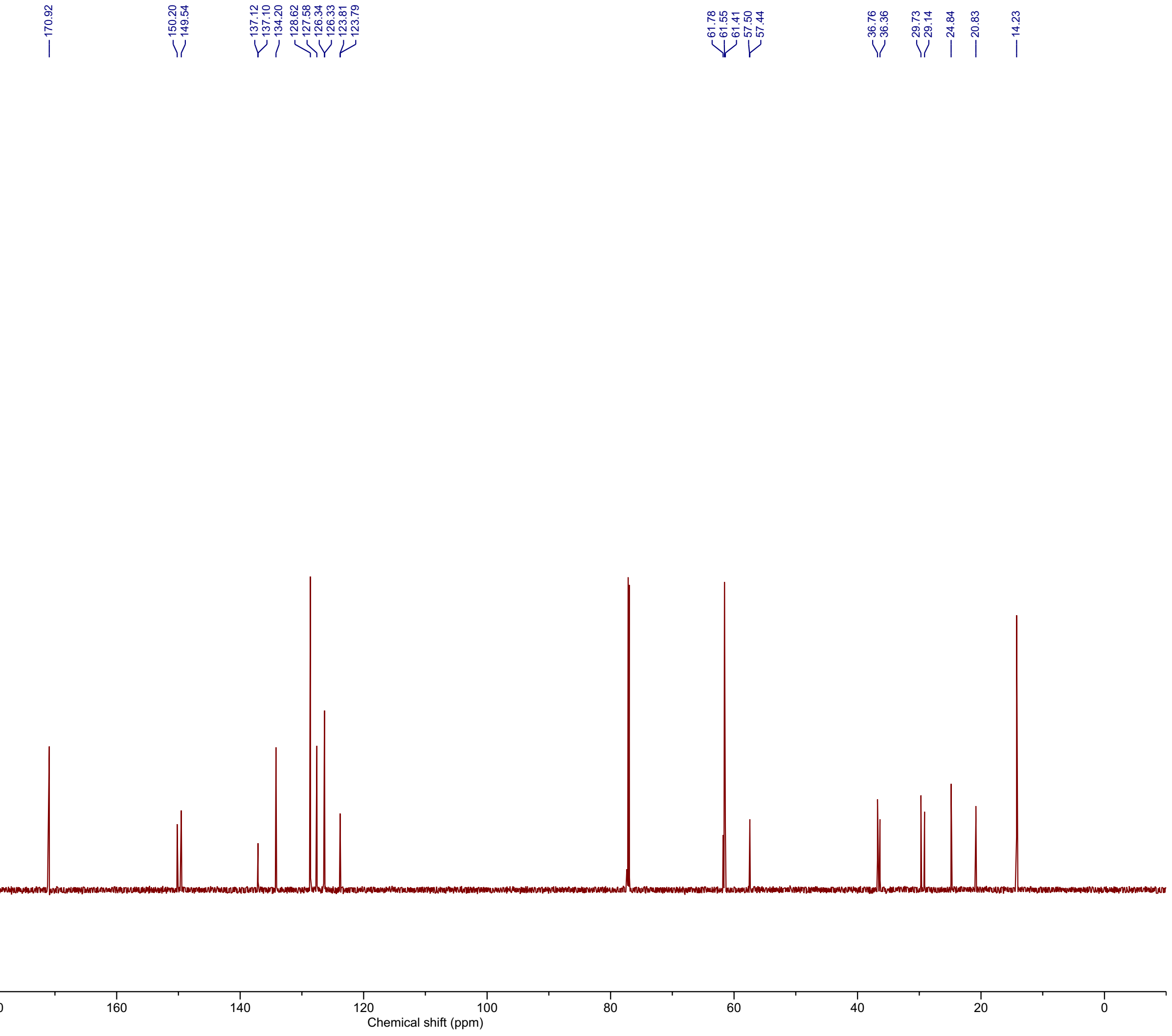

Supplementary Figure 85. ^{13}C NMR (176 MHz, $CDCl_3$) of **S36**.

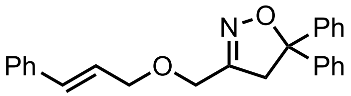

38

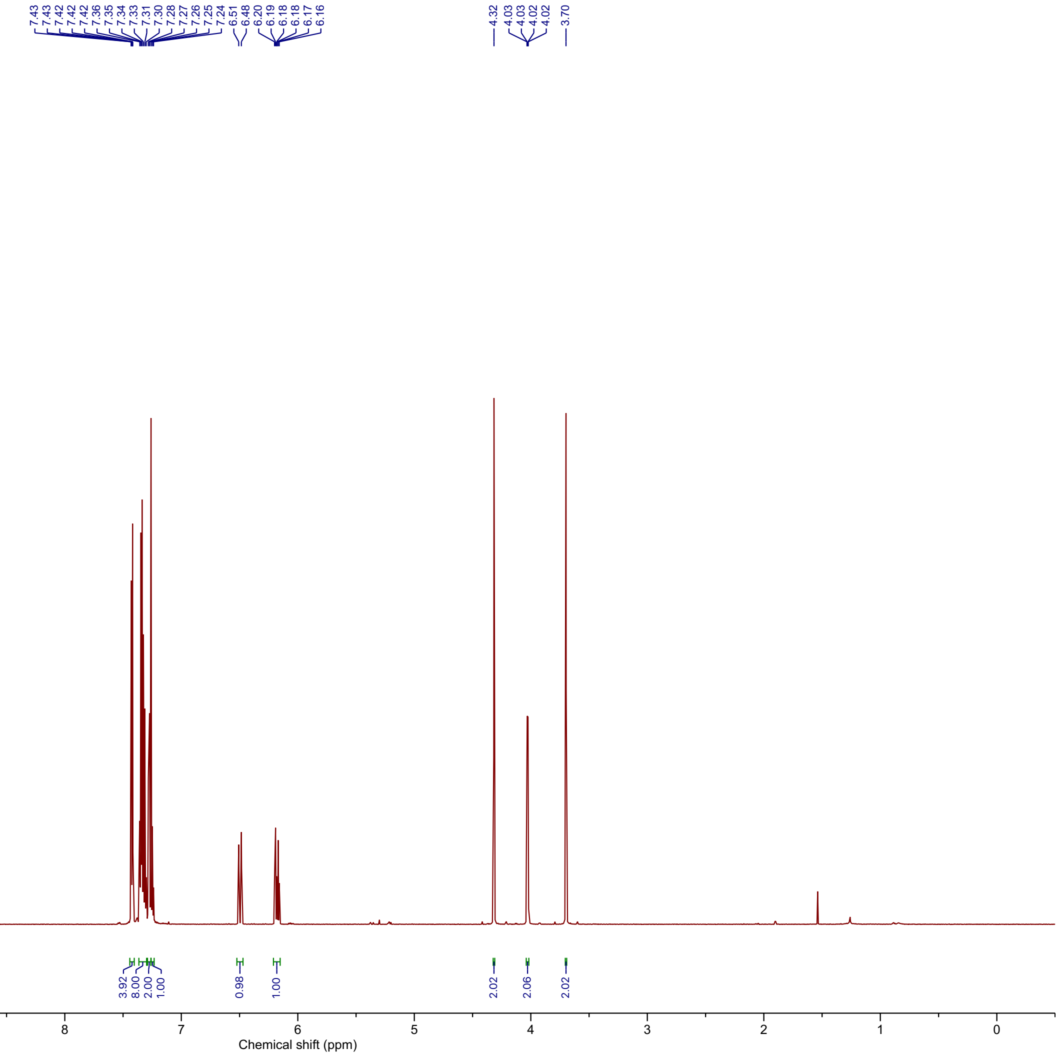

Supplementary Figure 86. ^{1}H NMR (700 MHz, $CDCl_3$) of **38**.

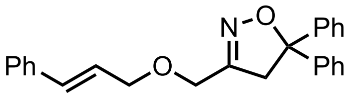

38

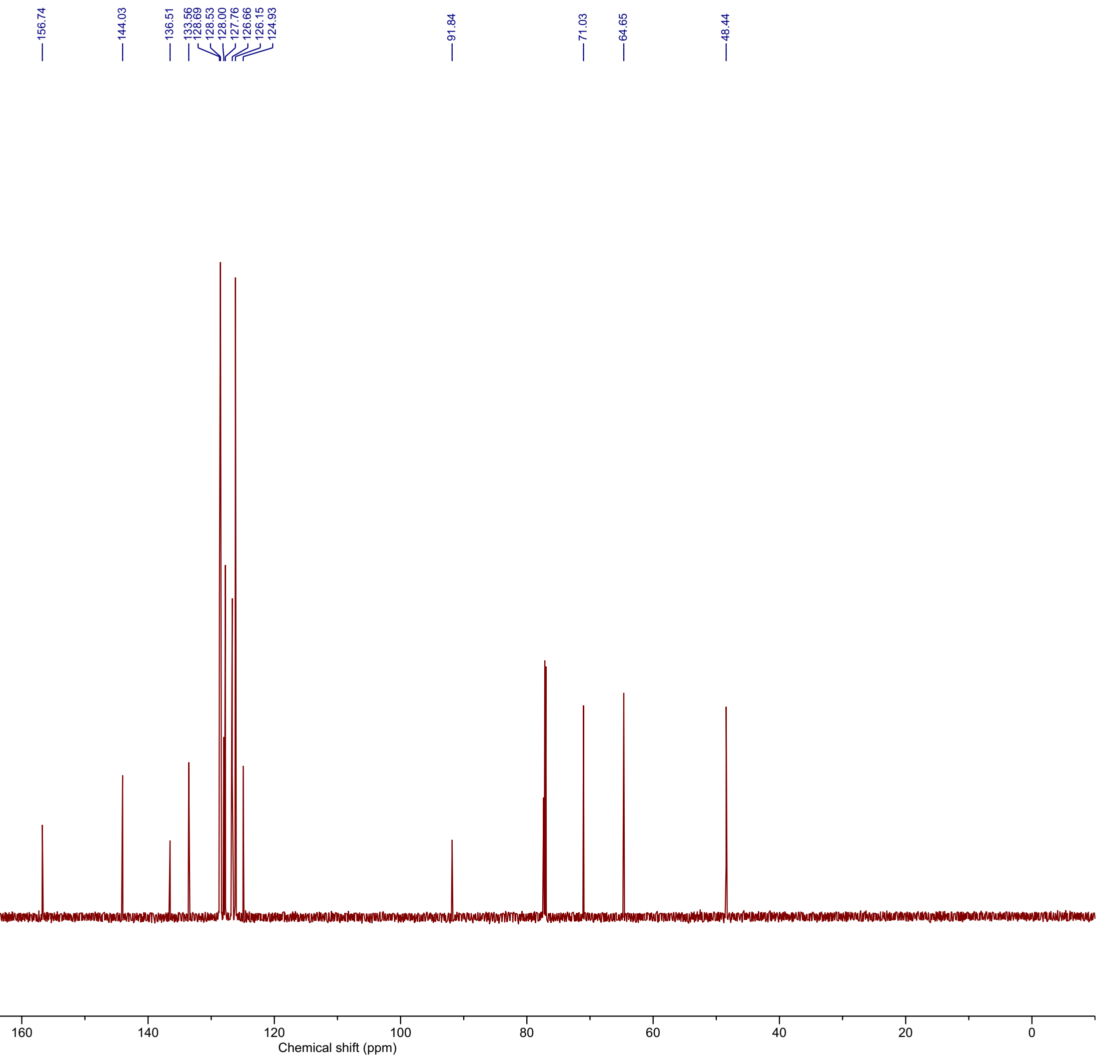

Supplementary Figure 87. ^{13}C NMR (176 MHz, $CDCl_3$) of **38**.

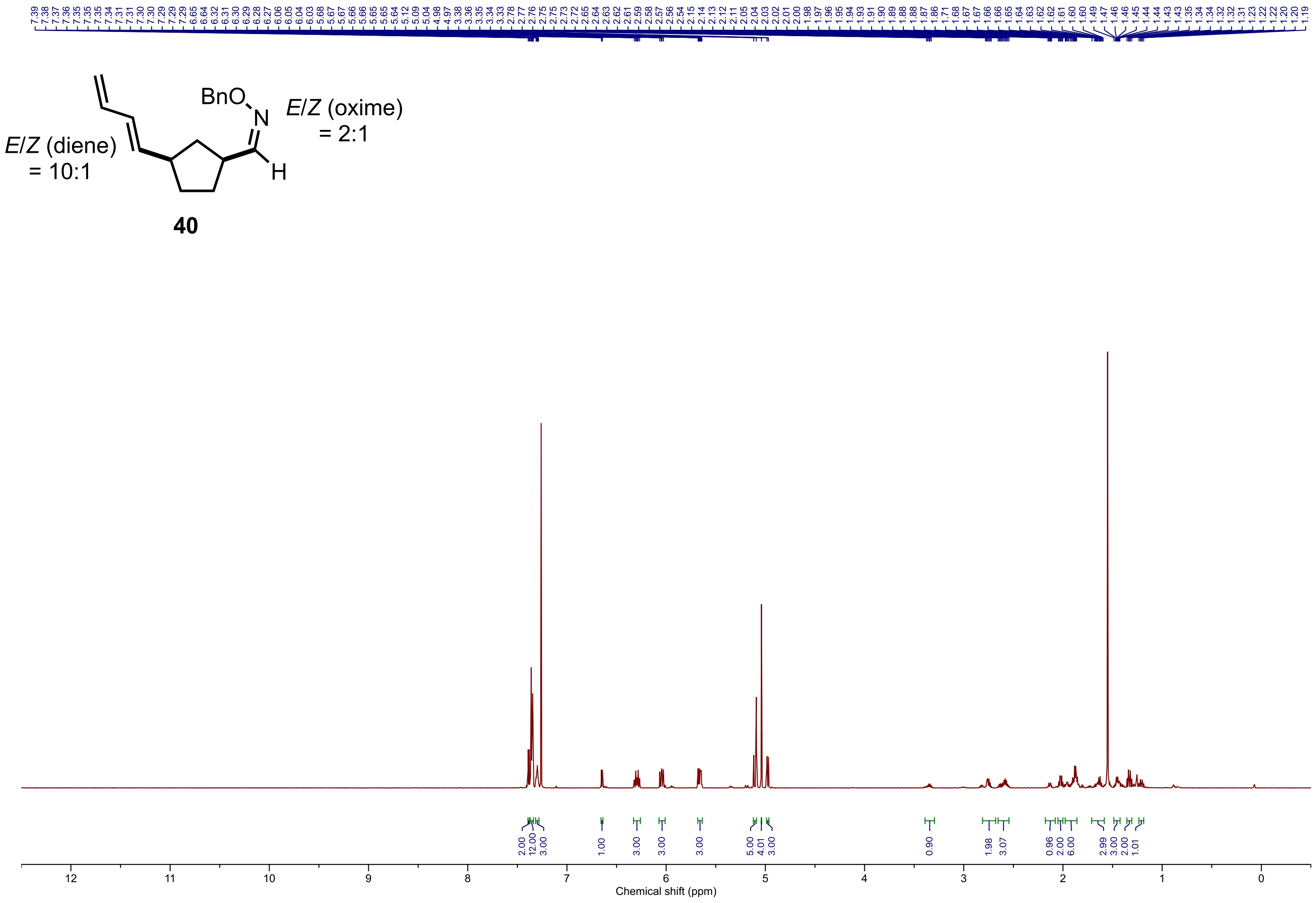

Supplementary Figure 88. ^{1}H NMR (700 MHz, $CDCl_3$) of **40**.

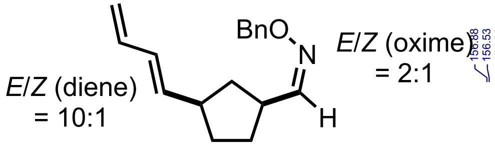

40

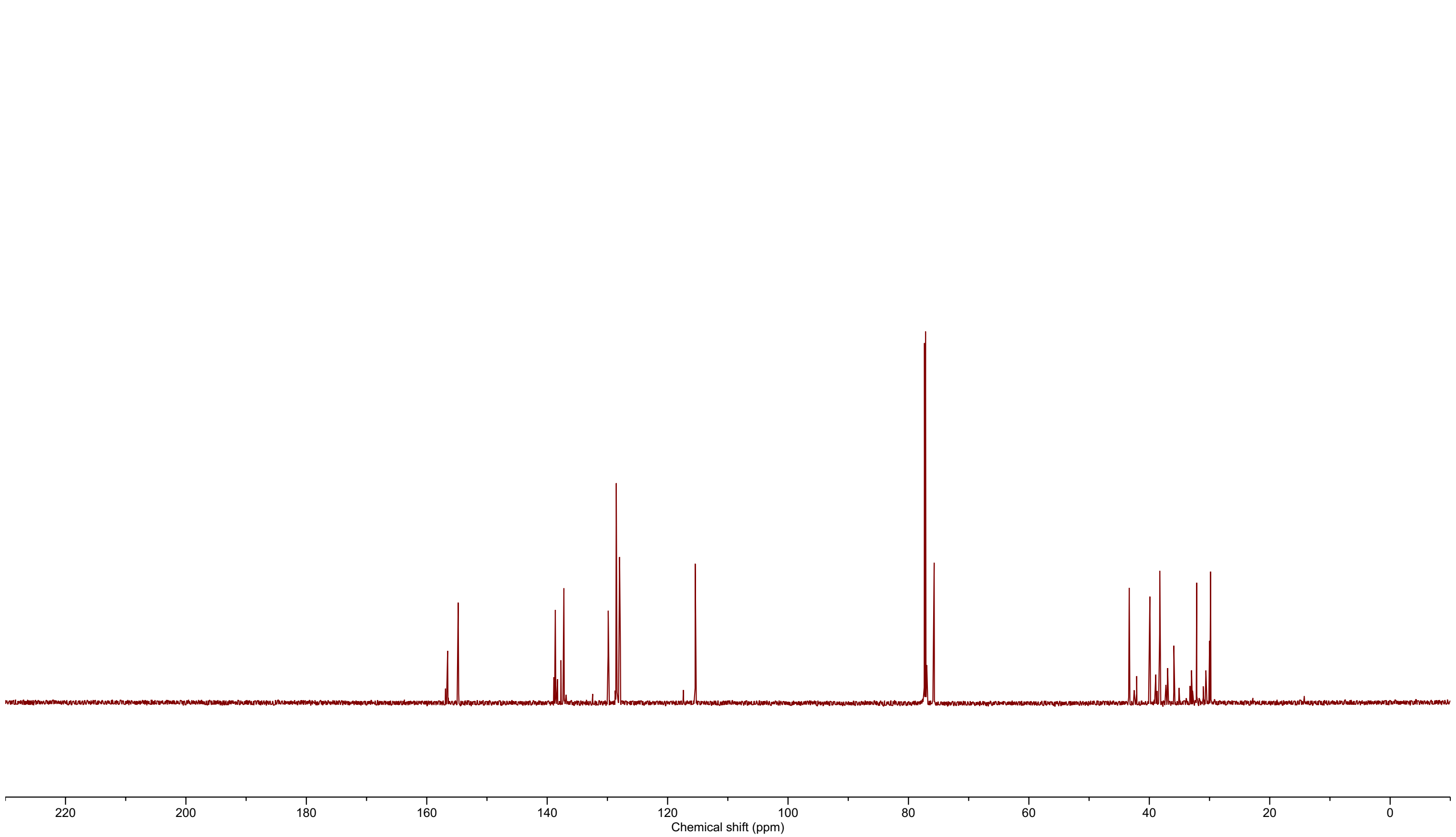

Supplementary Figure 89. ^{13}C NMR (176 MHz, $CDCl_3$) of **40**.

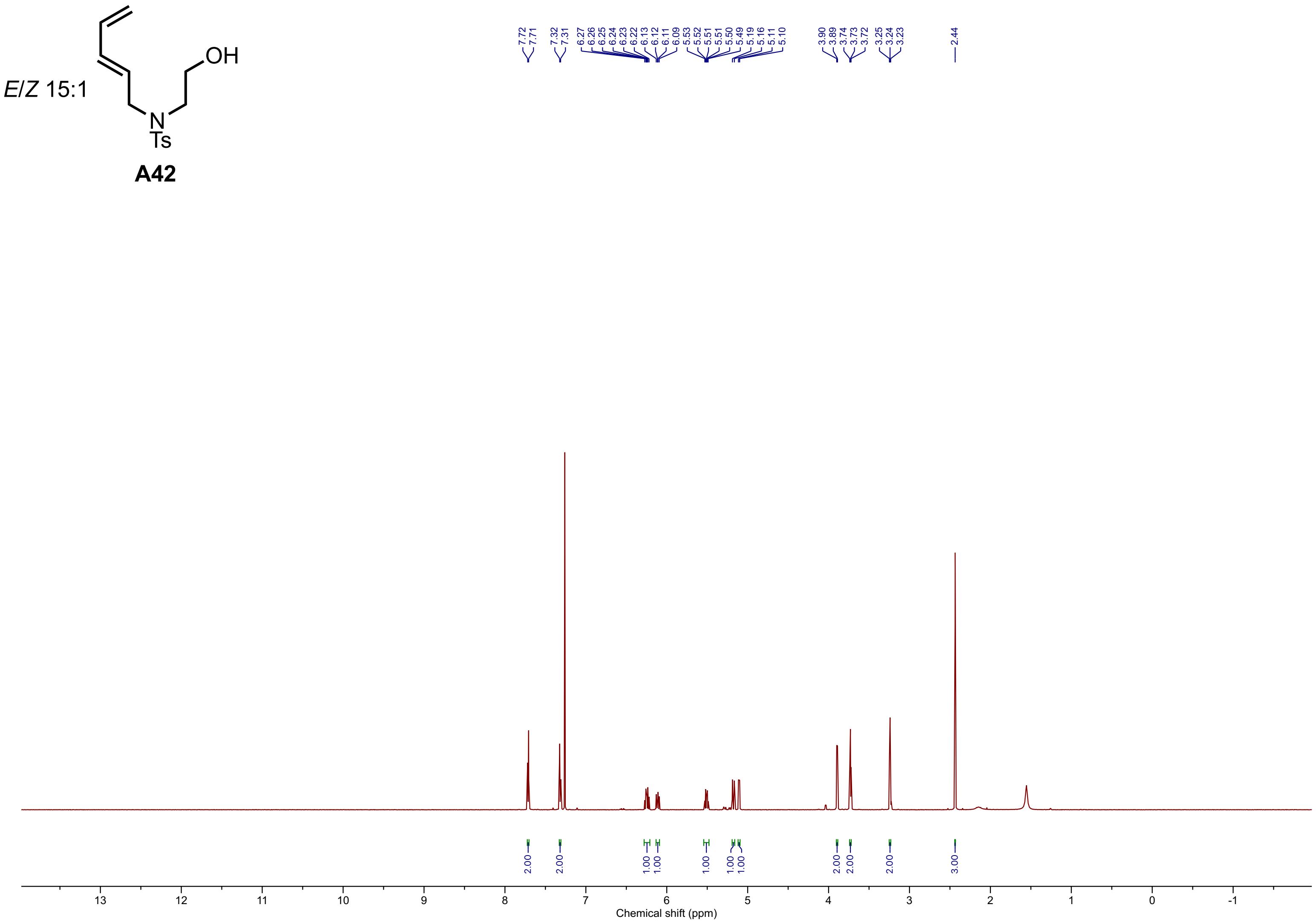

Supplementary Figure 90. ^{1}H NMR (700 MHz, $CDCl_3$) of **A42**.

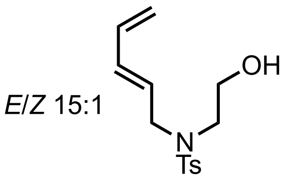

A42

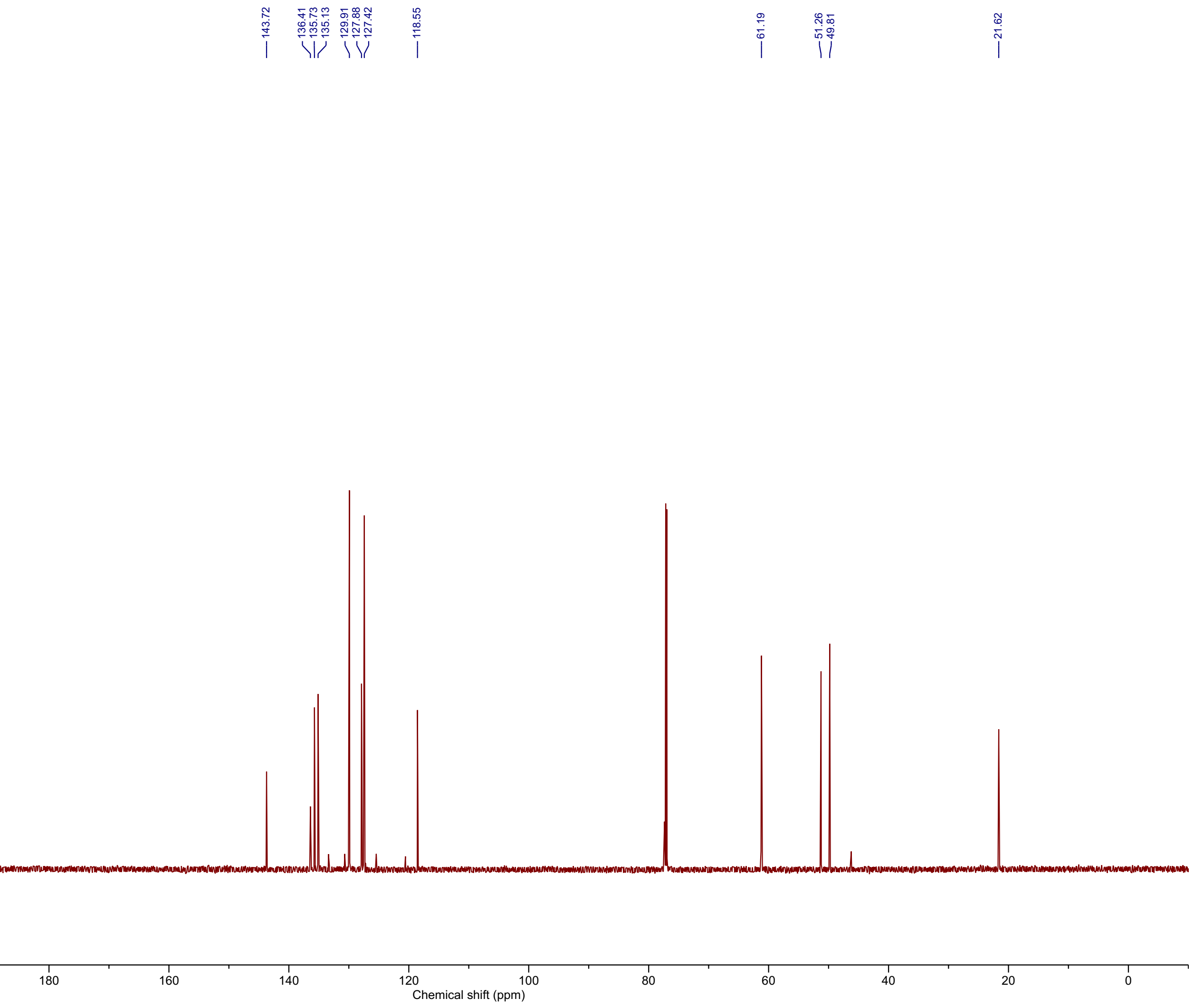

Supplementary Figure 91. ^{13}C NMR (176 MHz, $CDCl_3$) of **A42**.

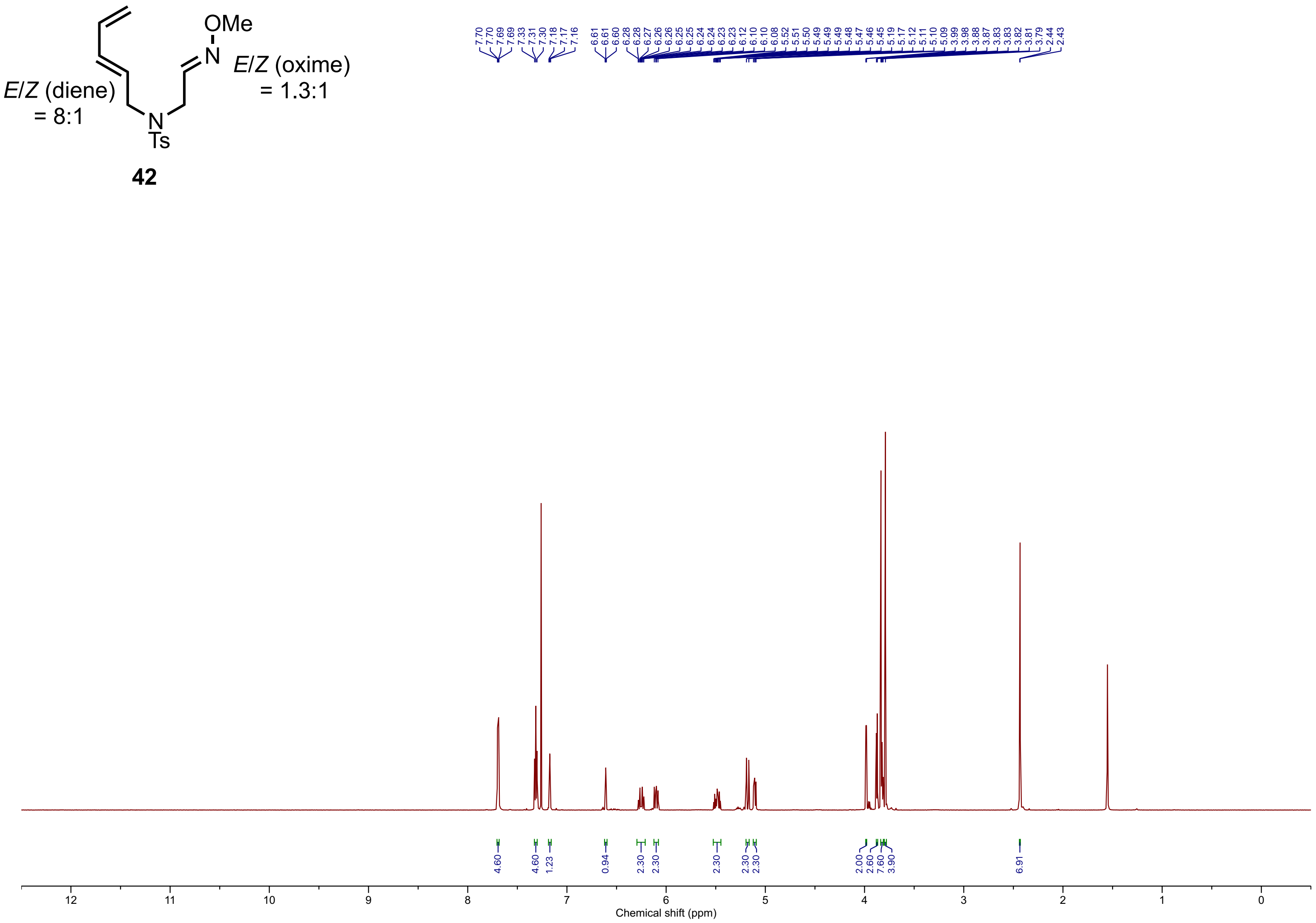

Supplementary Figure 92. ^{1}H NMR (700 MHz, $CDCl_3$) of **42**.

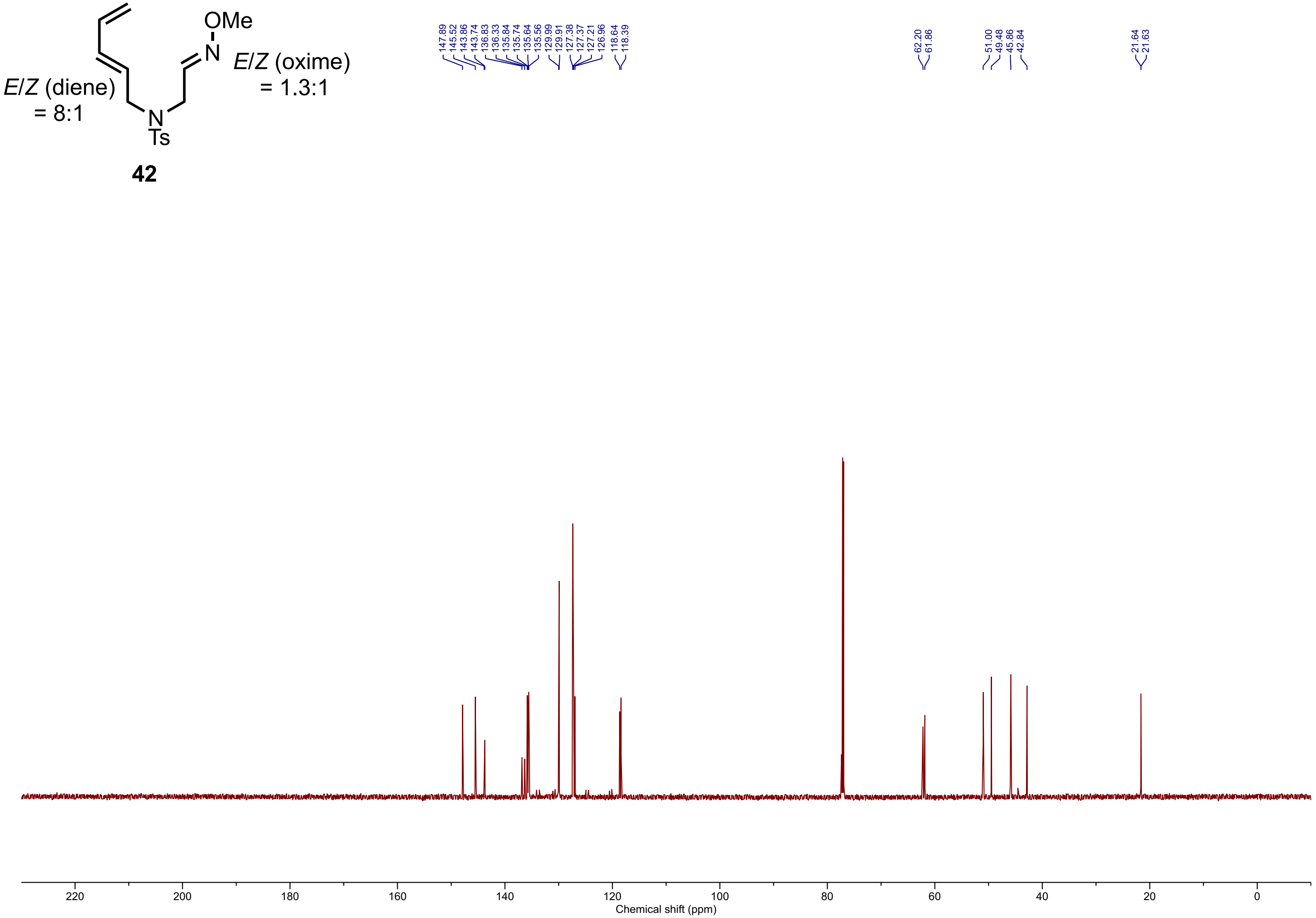

Supplementary Figure 93. ^{13}C NMR (176 MHz, $CDCl_3$) of **42**.

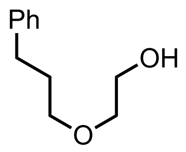

A45

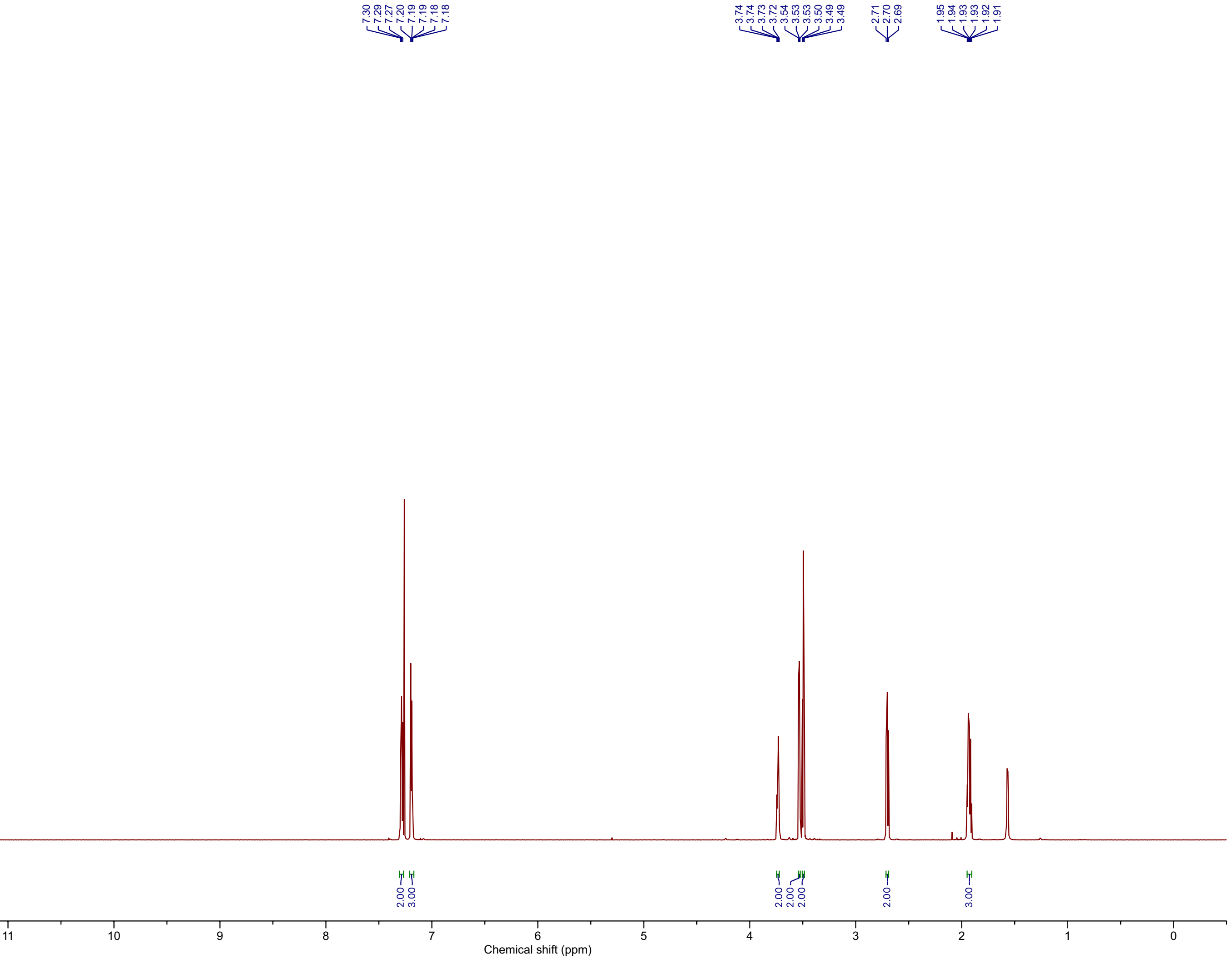

Supplementary Figure 94. ^{1}H NMR (700 MHz, $CDCl_3$) of **A45**.

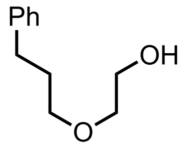

A45

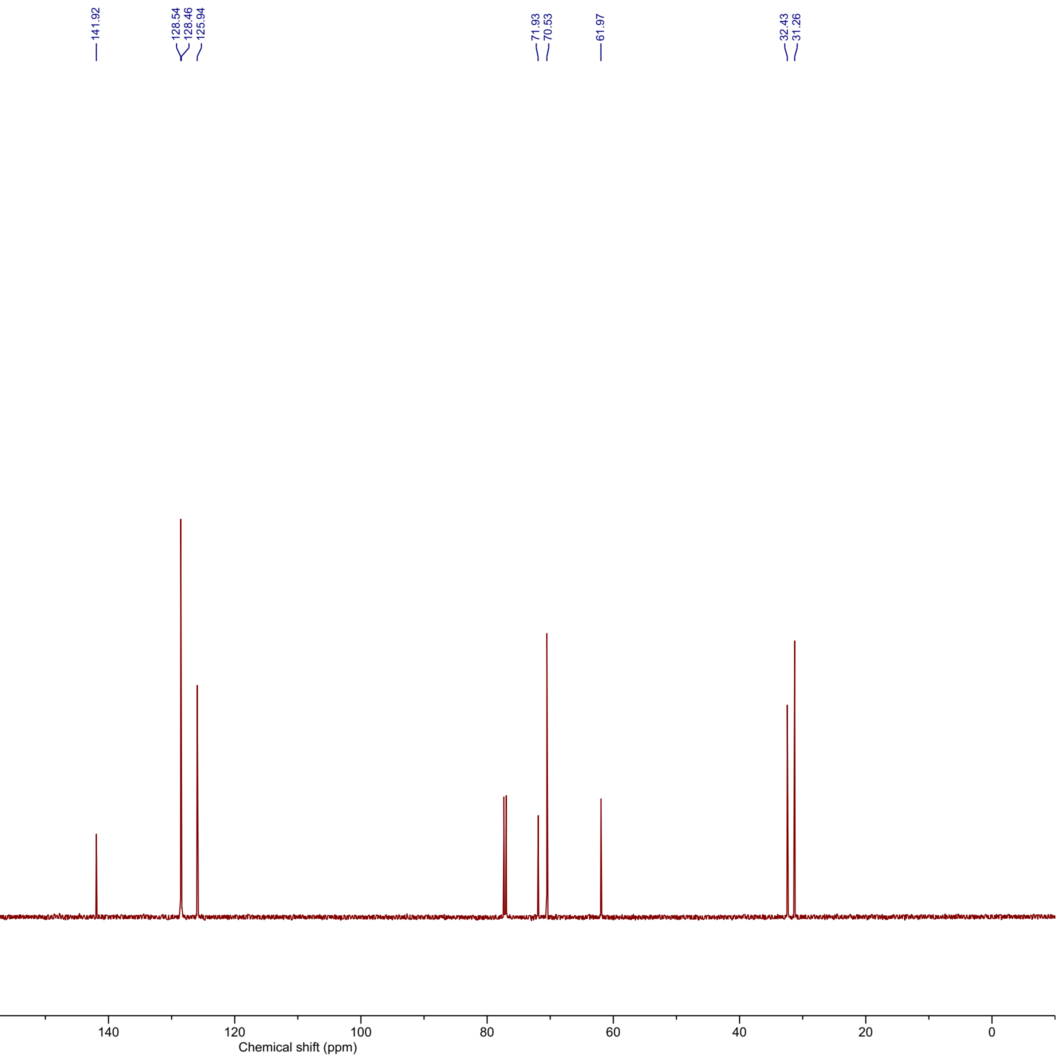

Supplementary Figure 95. ^{13}C NMR (176 MHz, $CDCl_3$) of **A45**.

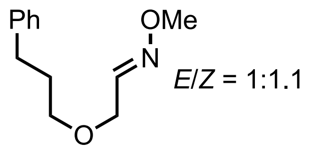

45

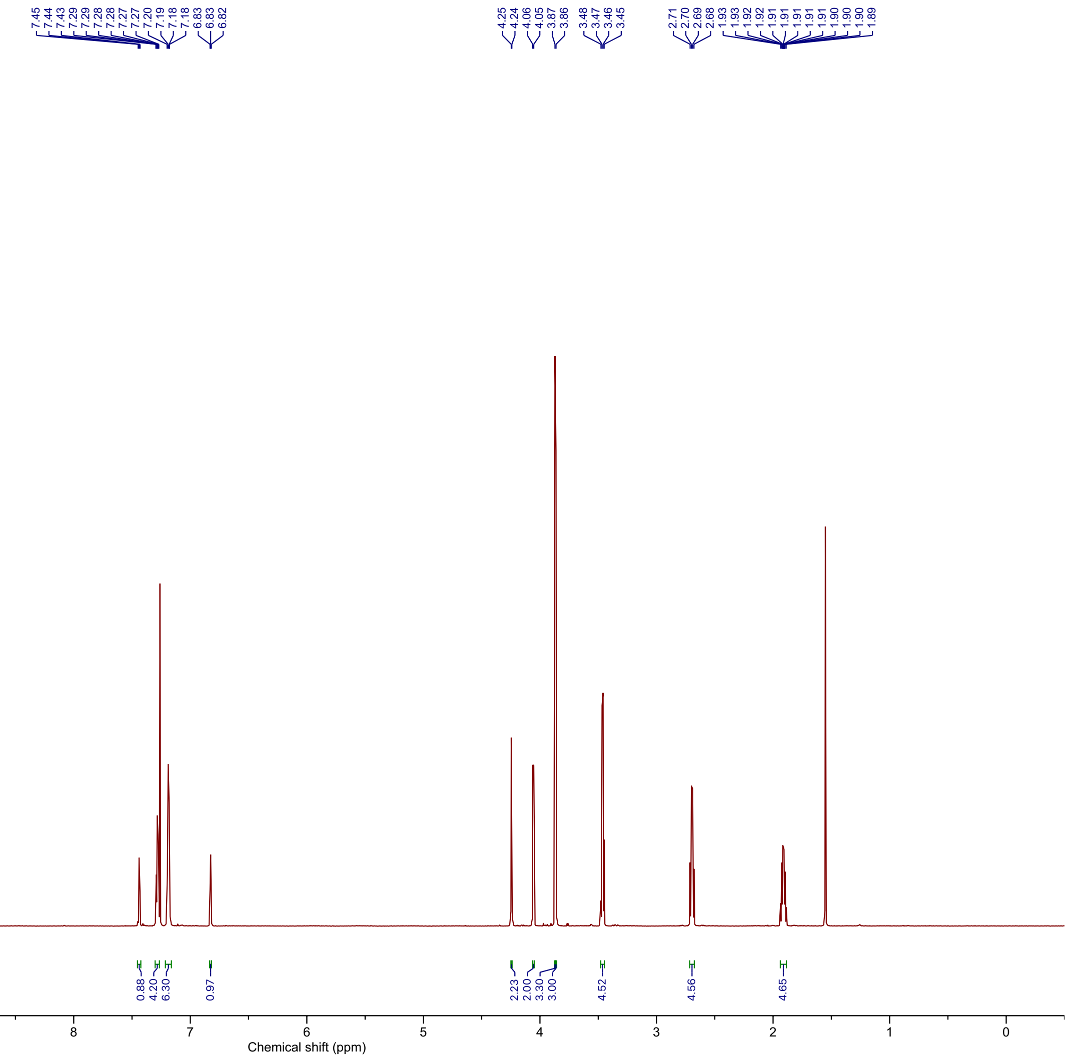

Supplementary Figure 96. ^{1}H NMR (700 MHz, $CDCl_3$) of **45**.

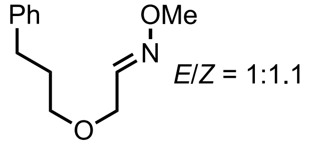

45

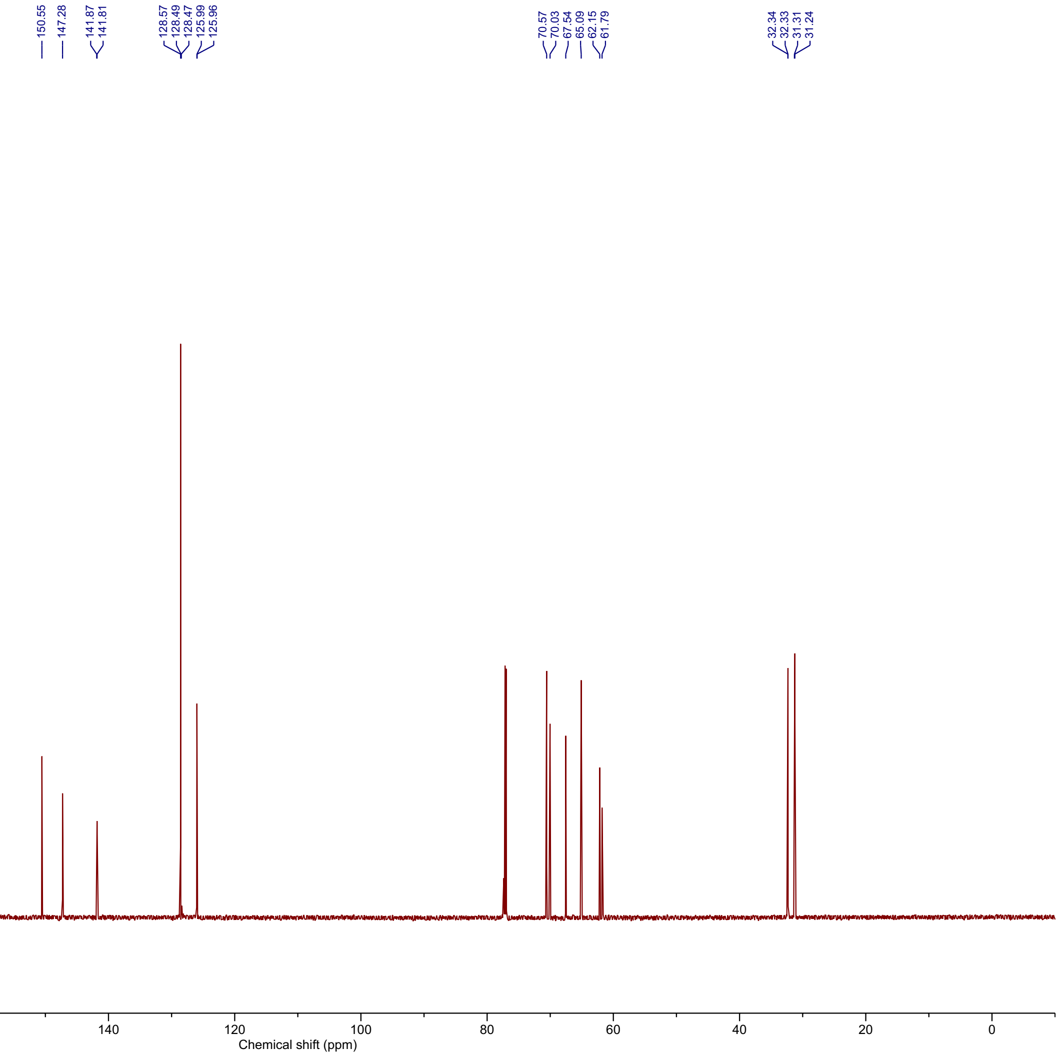

Supplementary Figure 97. ^{13}C NMR (176 MHz, $CDCl_3$) of **45**.

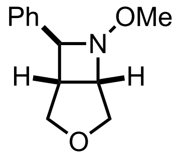

16

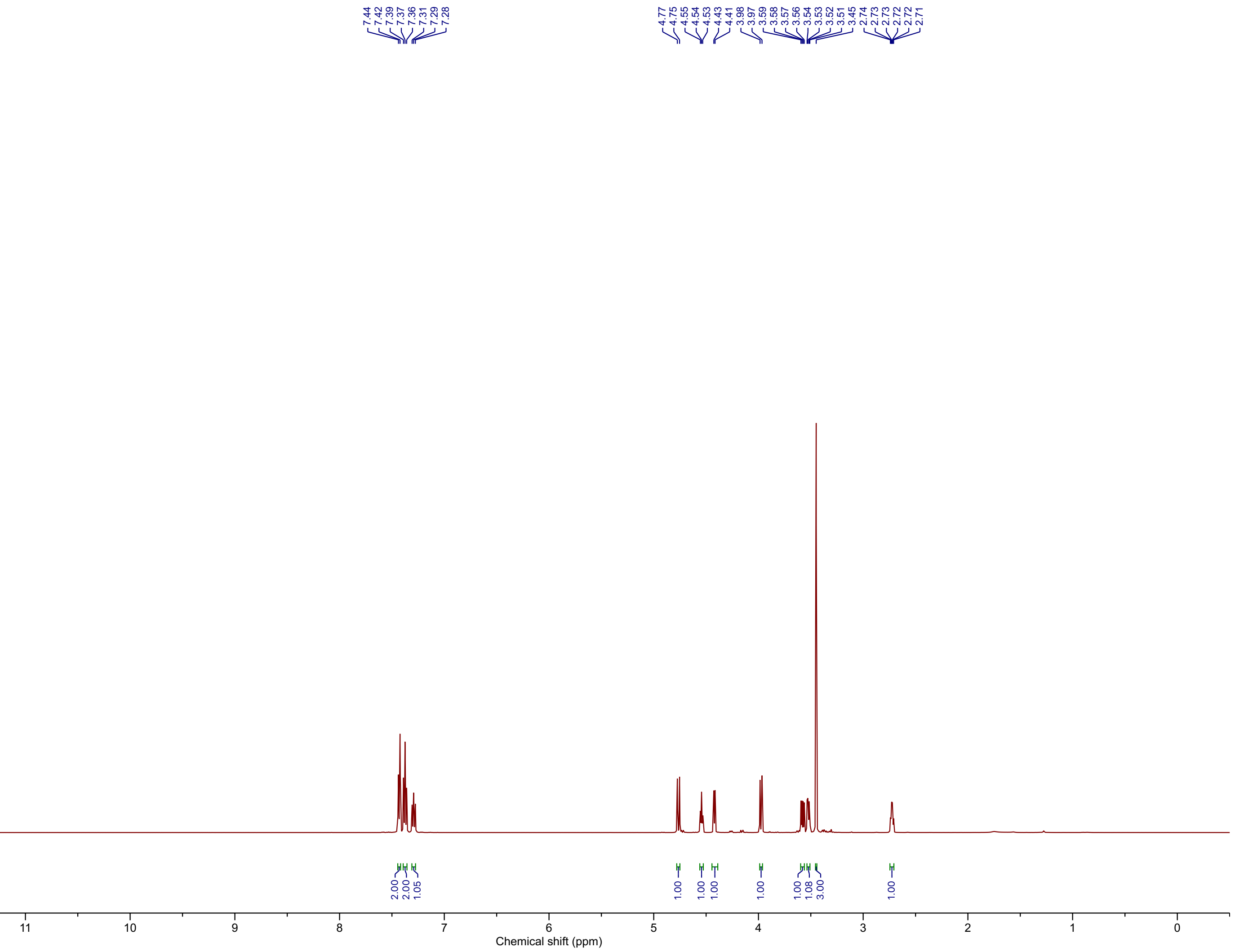

Supplementary Figure 98. ^{1}H NMR (500 MHz, $CDCl_3$) of **16**.

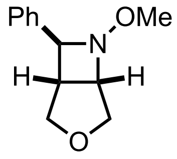

16

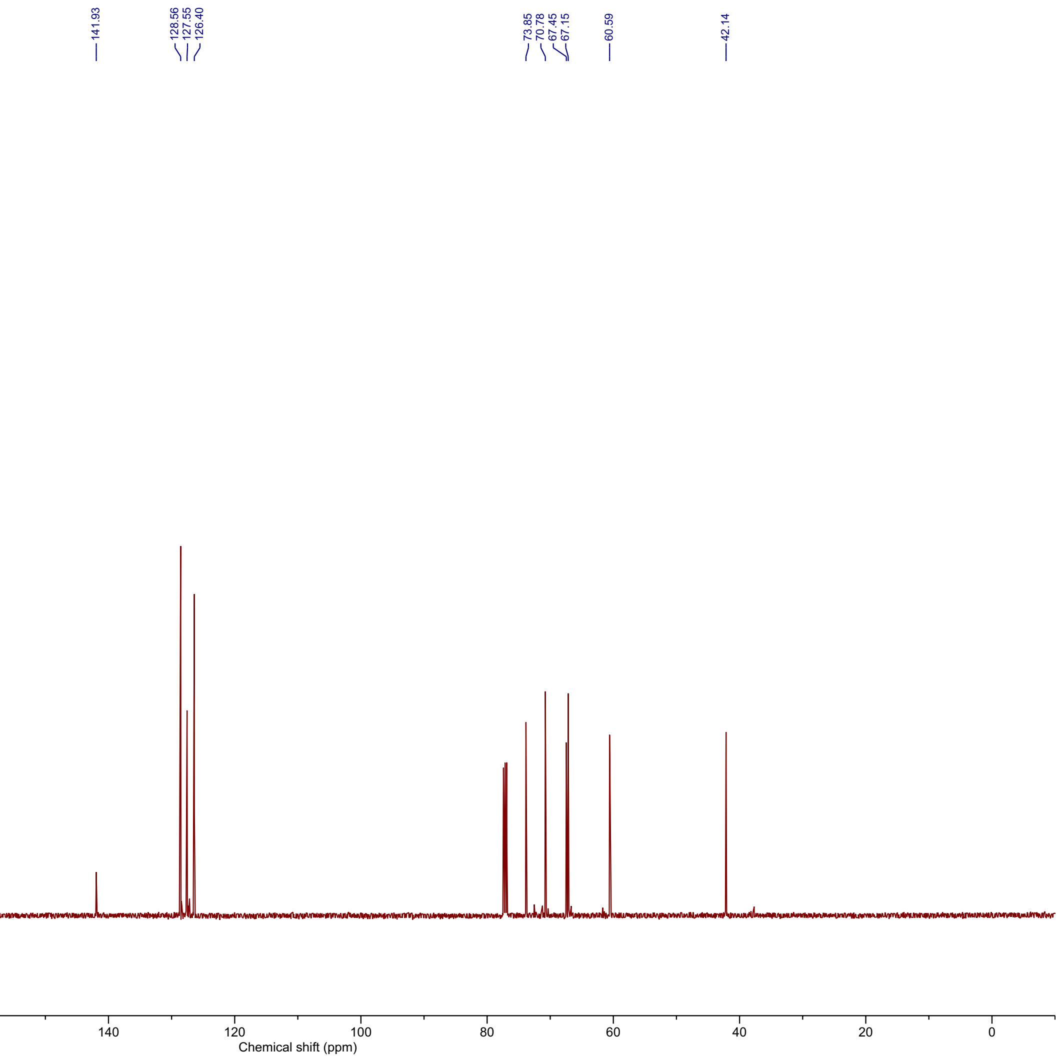

Supplementary Figure 99. ^{13}C NMR (126 MHz, $CDCl_3$) of **16**.

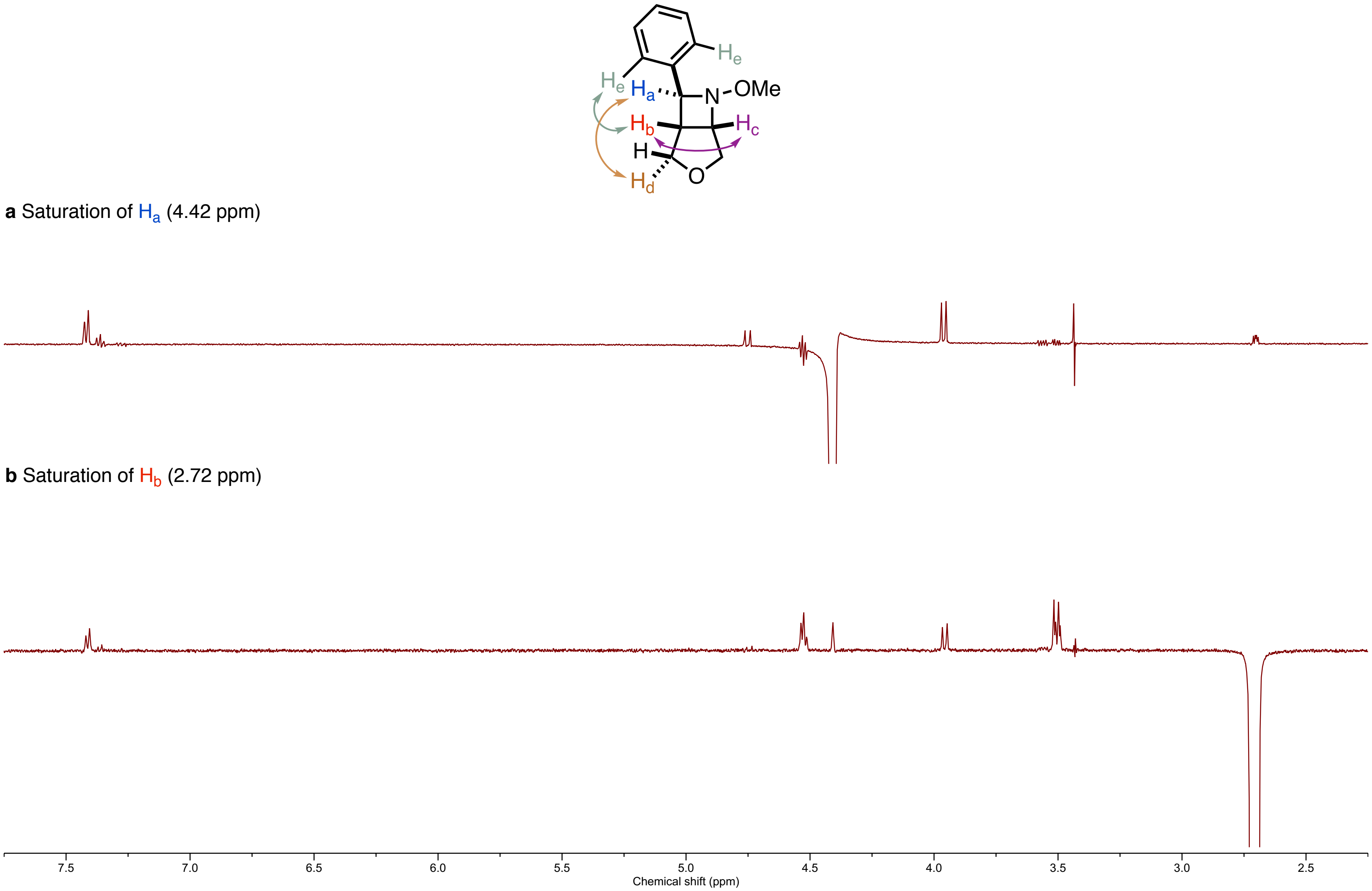

Supplementary Figure 100. ^{1}H NMR NOE of **16**. **a** Saturation of H_a at 4.42 ppm; **b** Saturation of H_b at 2.72 ppm.

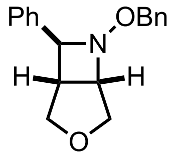

19a

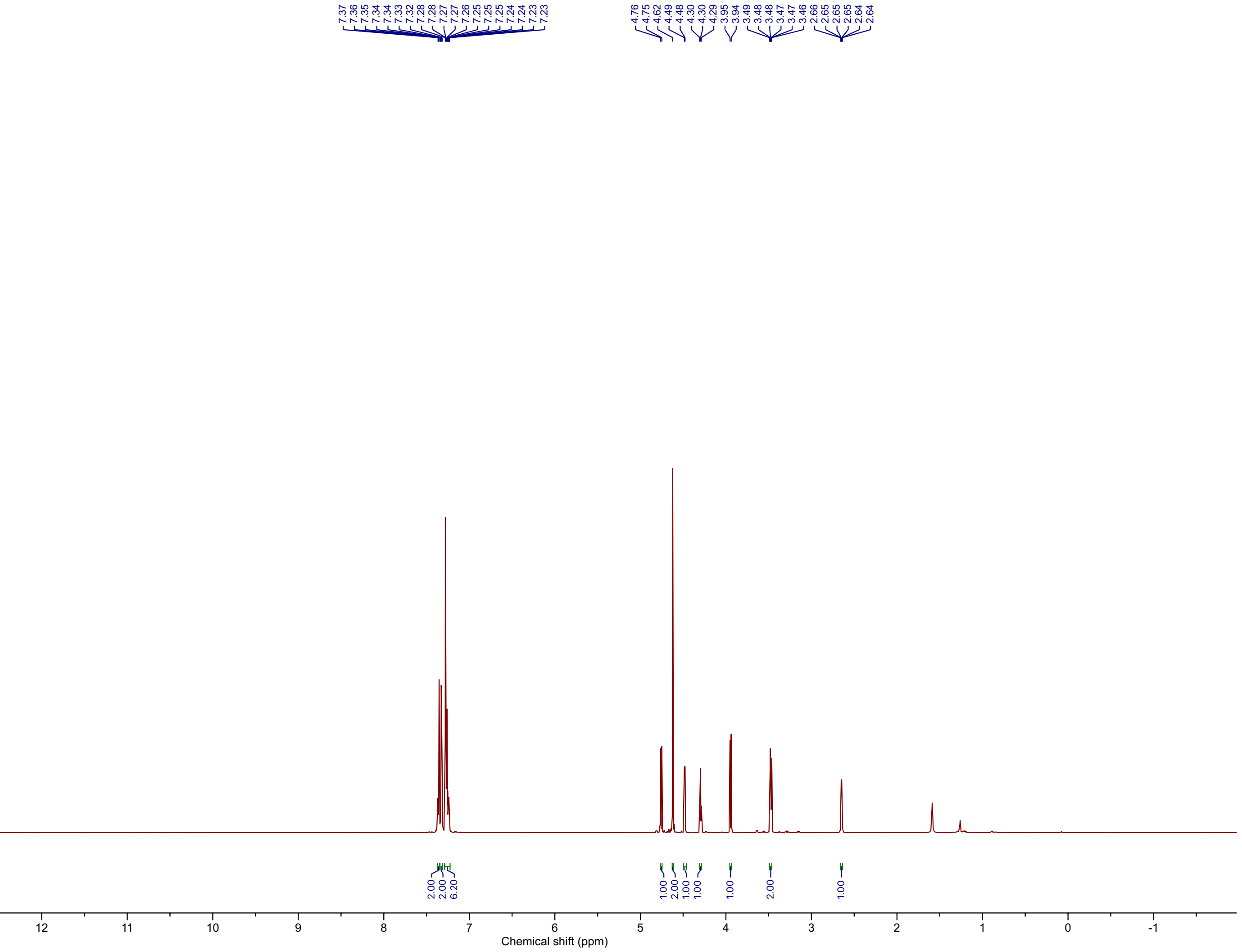

Supplementary Figure 101. ^{1}H NMR (700 MHz, $CDCl_3$) of **19a**.

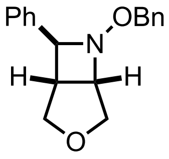

19a

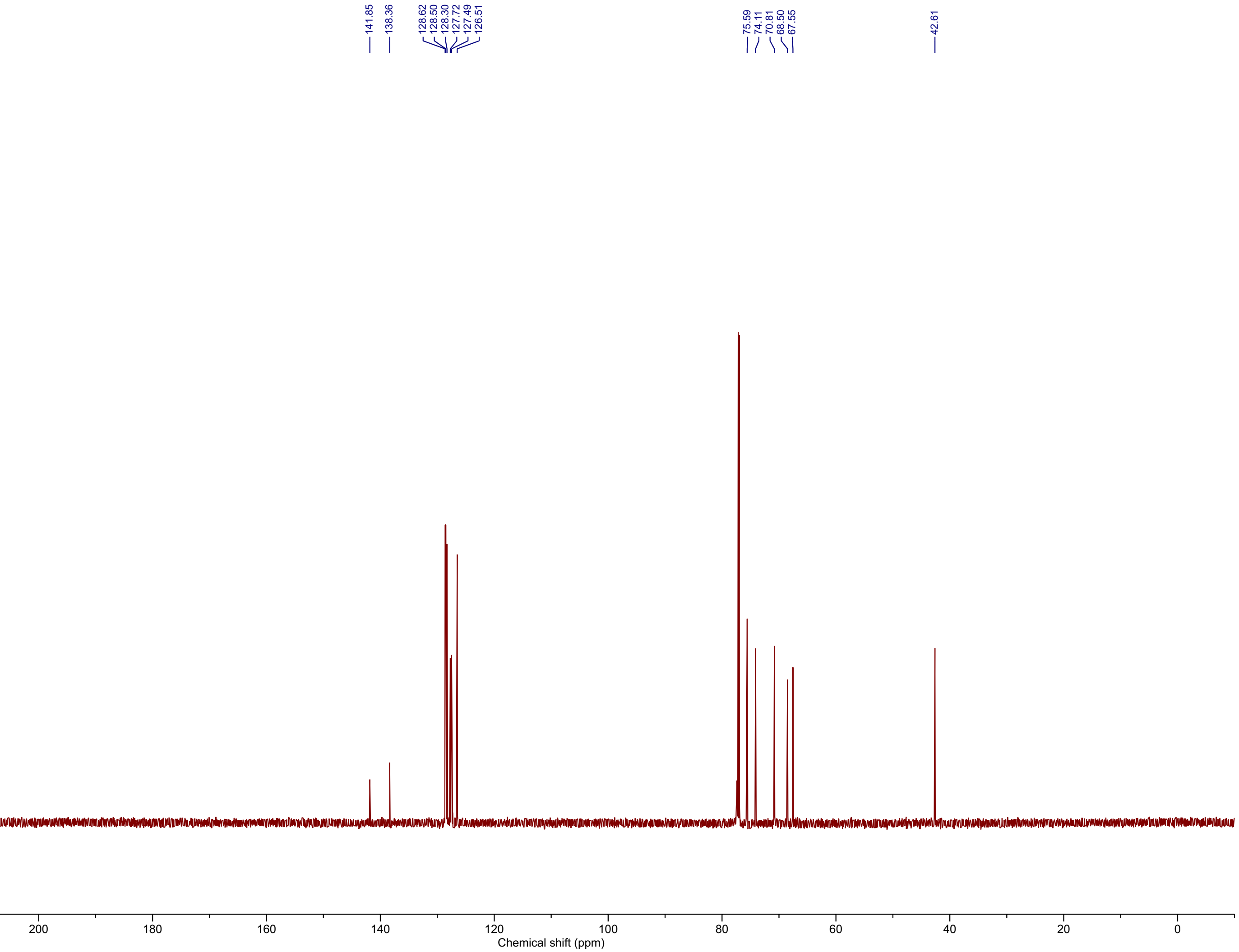

Supplementary Figure 102. ^{13}C NMR (176 MHz, $CDCl_3$) of **19a**.

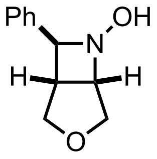

19b

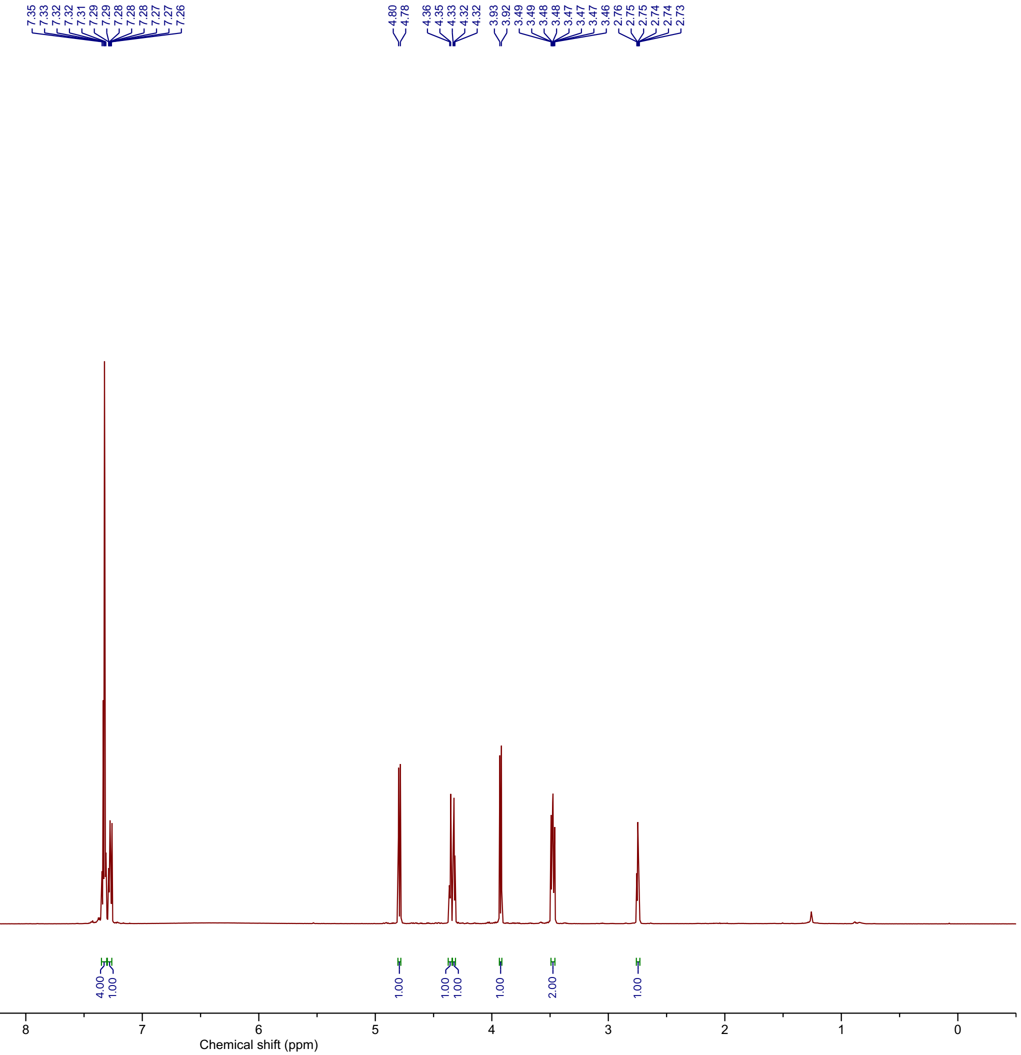

Supplementary Figure 103. ^{1}H NMR (700 MHz, $CDCl_3$) of **19b**.

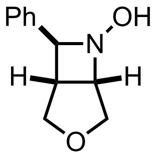

19b

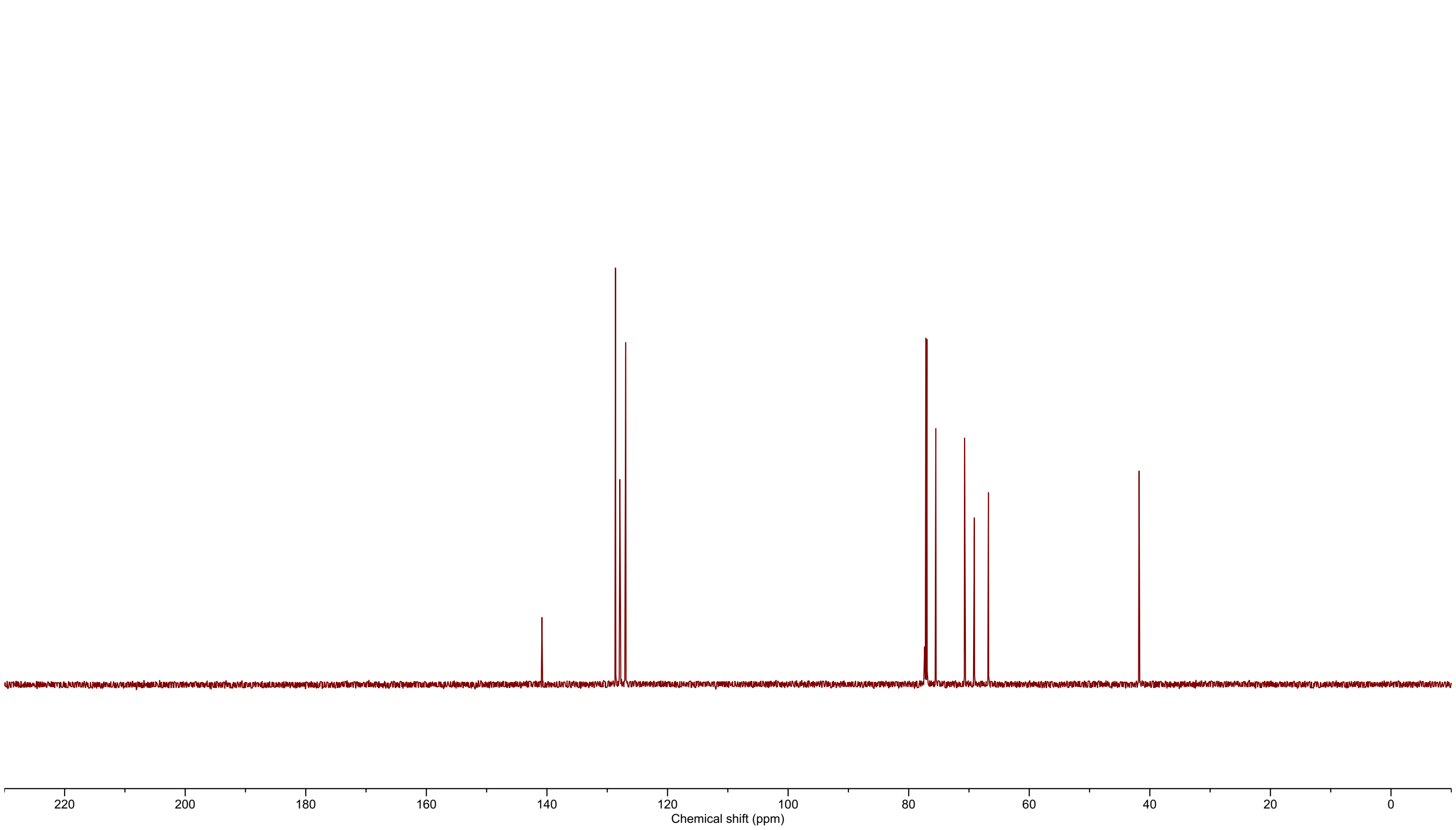

Supplementary Figure 104. ^{13}C NMR (176 MHz, $CDCl_3$) of **19b**.

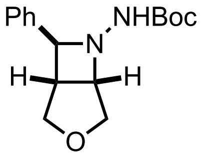

19c

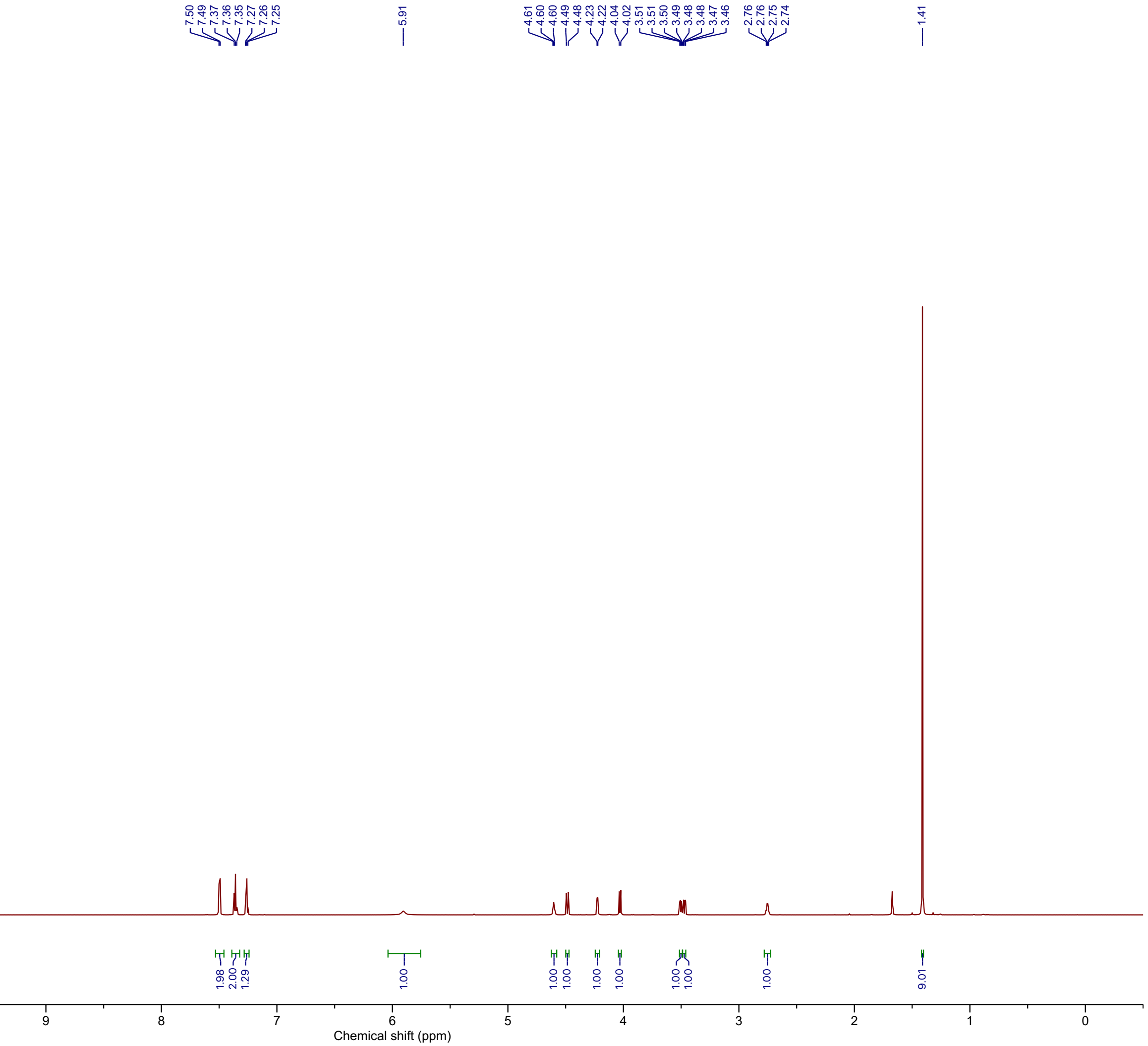

Supplementary Figure 105. ^{1}H NMR (700 MHz, $CDCl_3$) of **19c**.

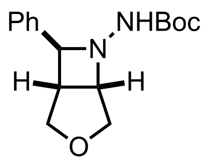

19c

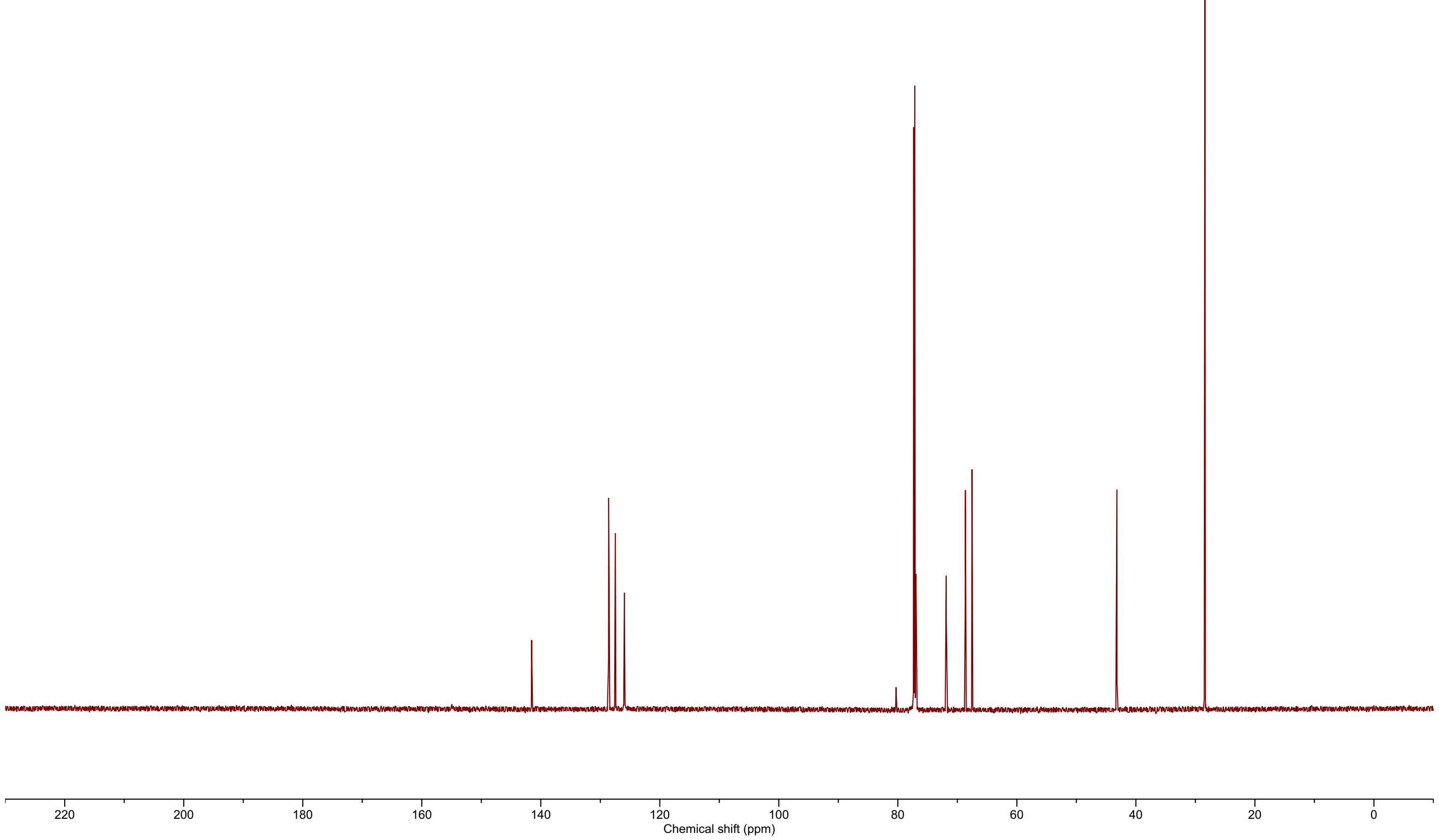

Supplementary Figure 106. ^{13}C NMR (176 MHz, $CDCl_3$) of **19c**.

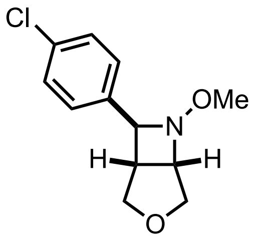

20

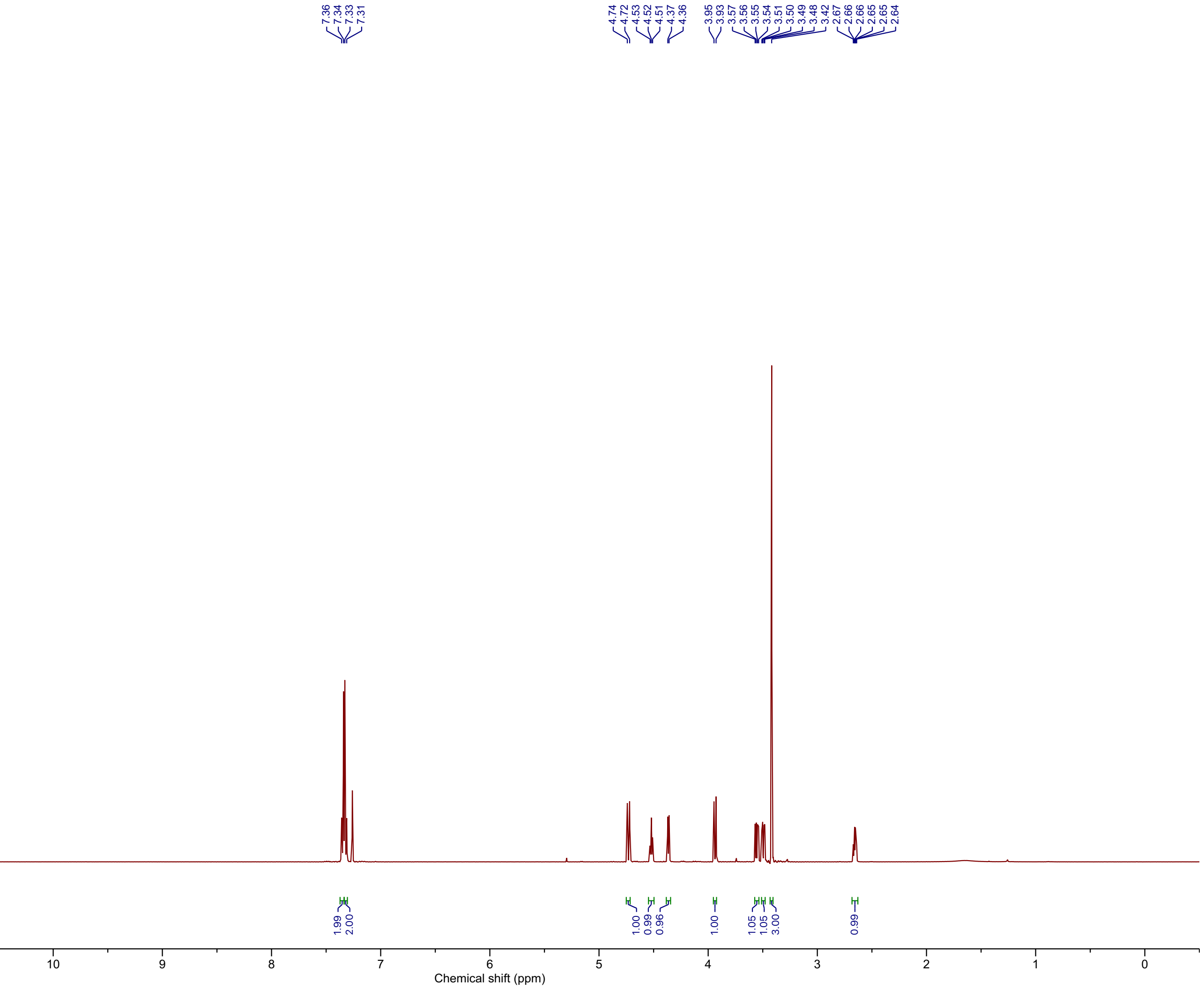

Supplementary Figure 107. ^{1}H NMR (500 MHz, $CDCl_3$) of **20**.

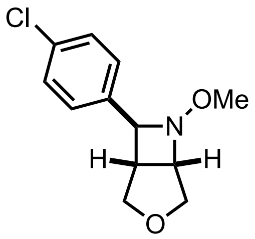

20

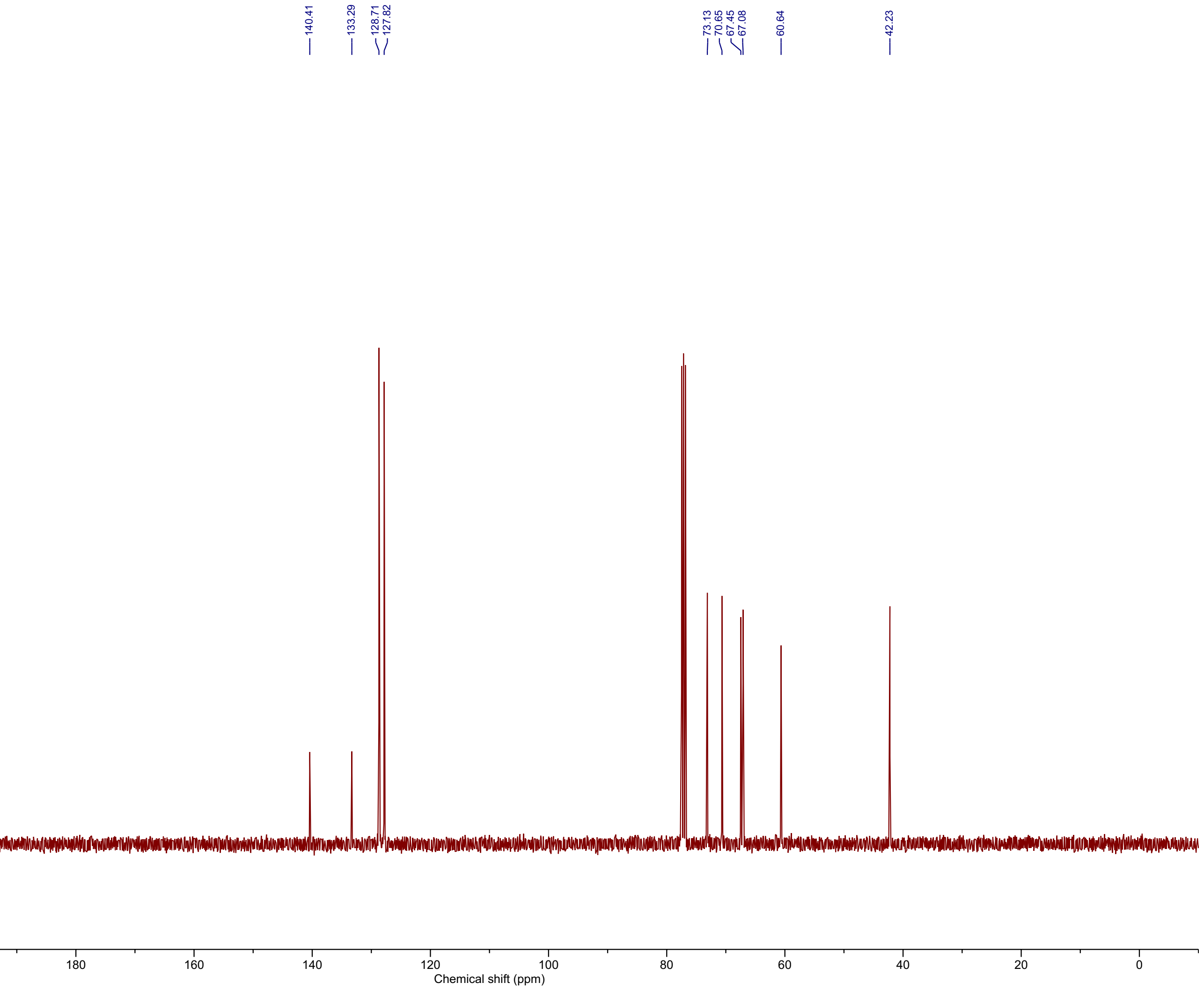

Supplementary Figure 108. ^{13}C NMR (100 MHz, $CDCl_3$) of **20**.

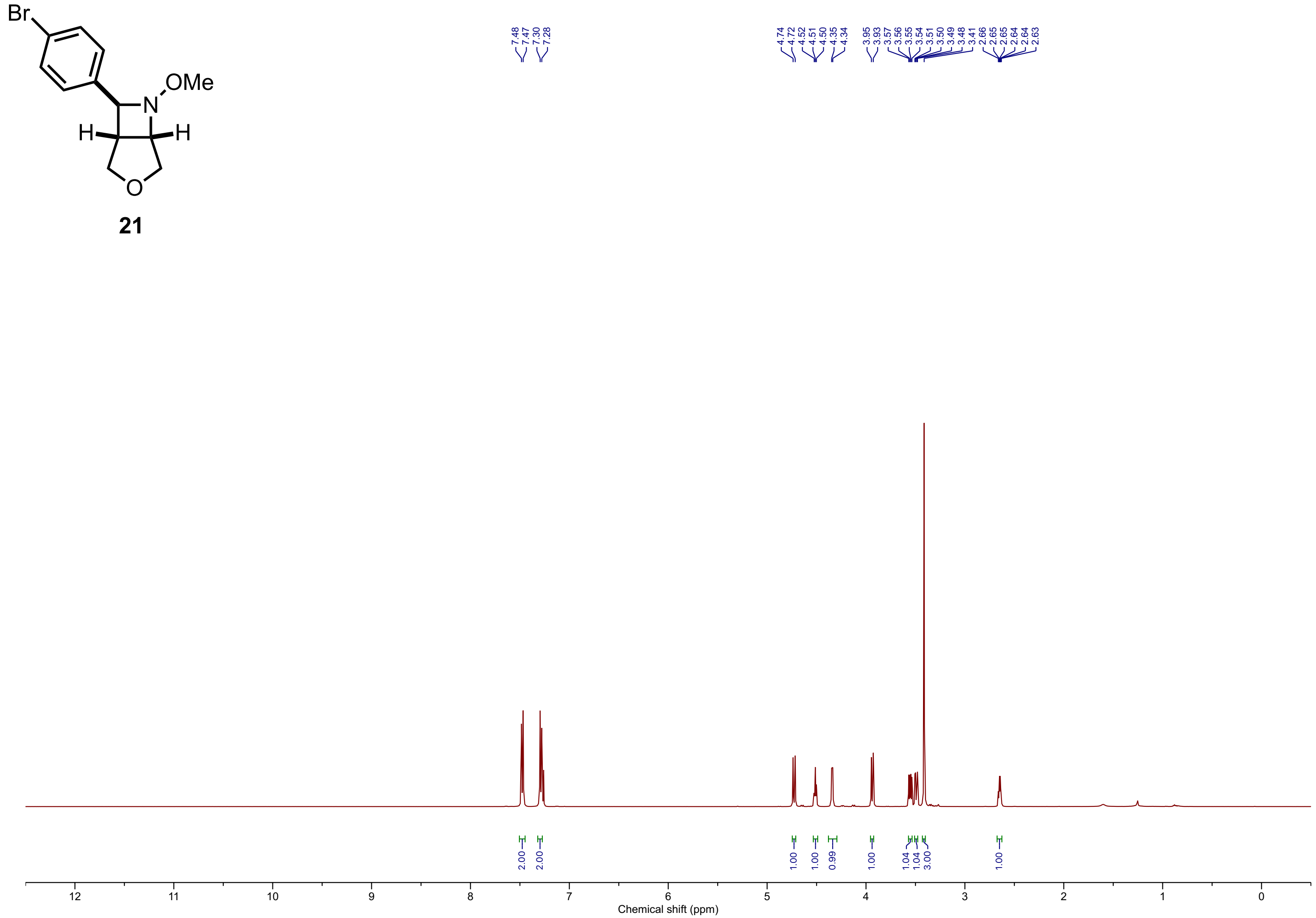

Supplementary Figure 109. ^{1}H NMR (500 MHz, $CDCl_3$) of **21**.

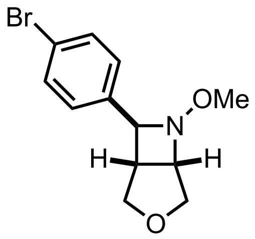

21

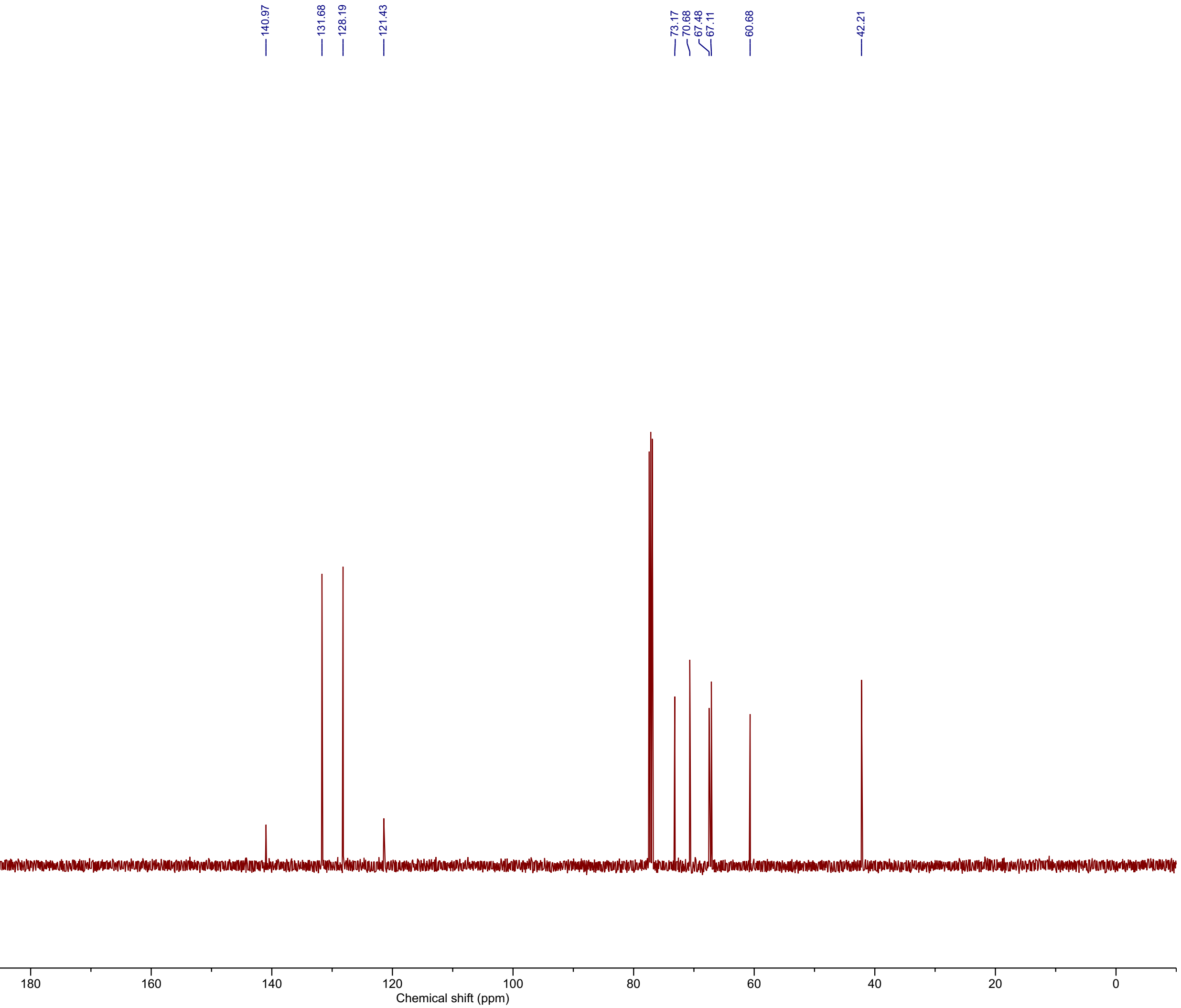

Supplementary Figure 110. ^{13}C NMR (126 MHz, $CDCl_3$) of **21**.

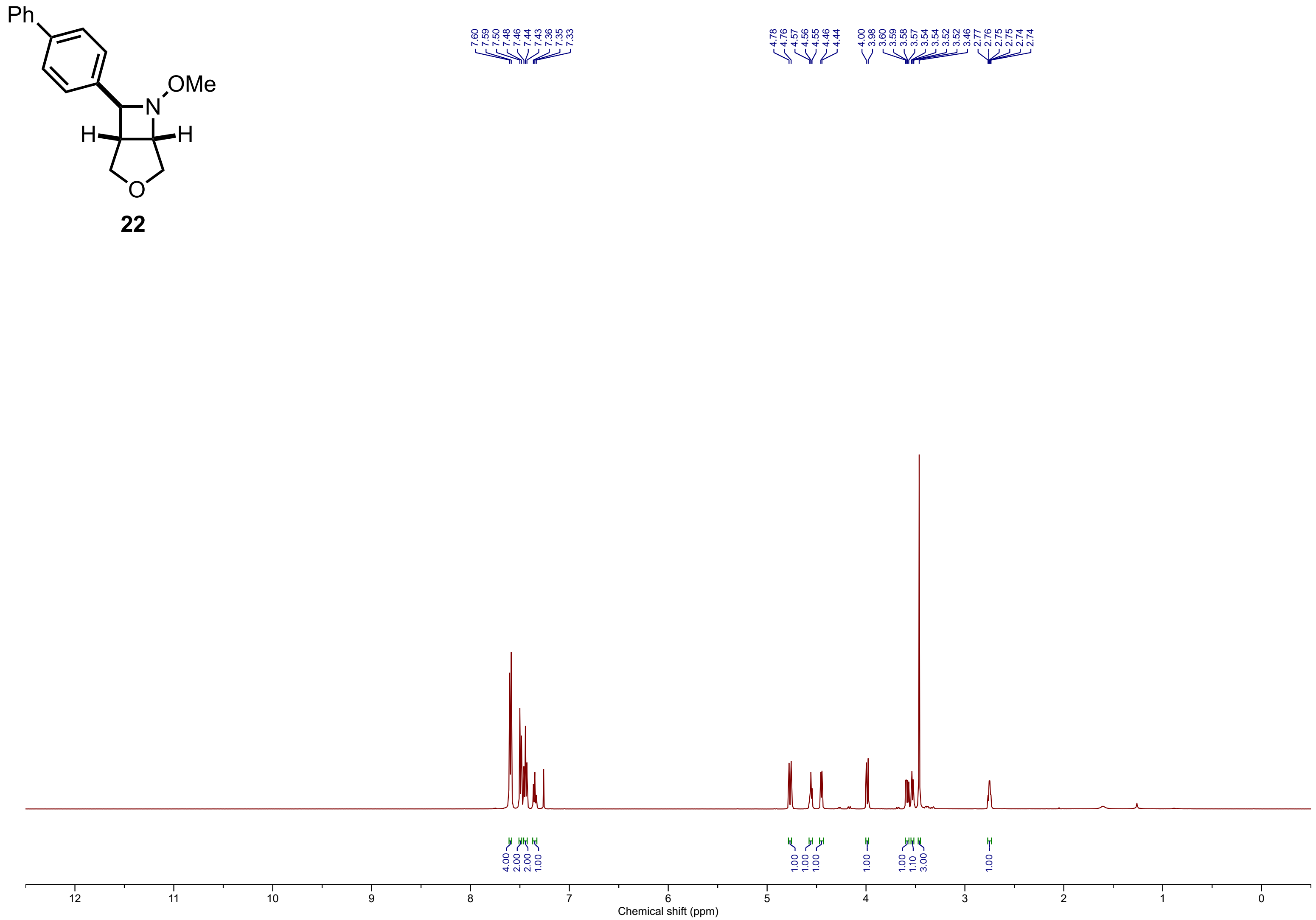

Supplementary Figure 111. ^{1}H NMR (500 MHz, $CDCl_3$) of **22**.

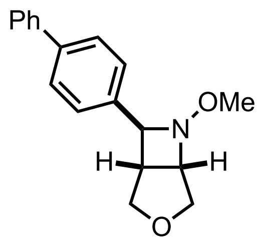

22

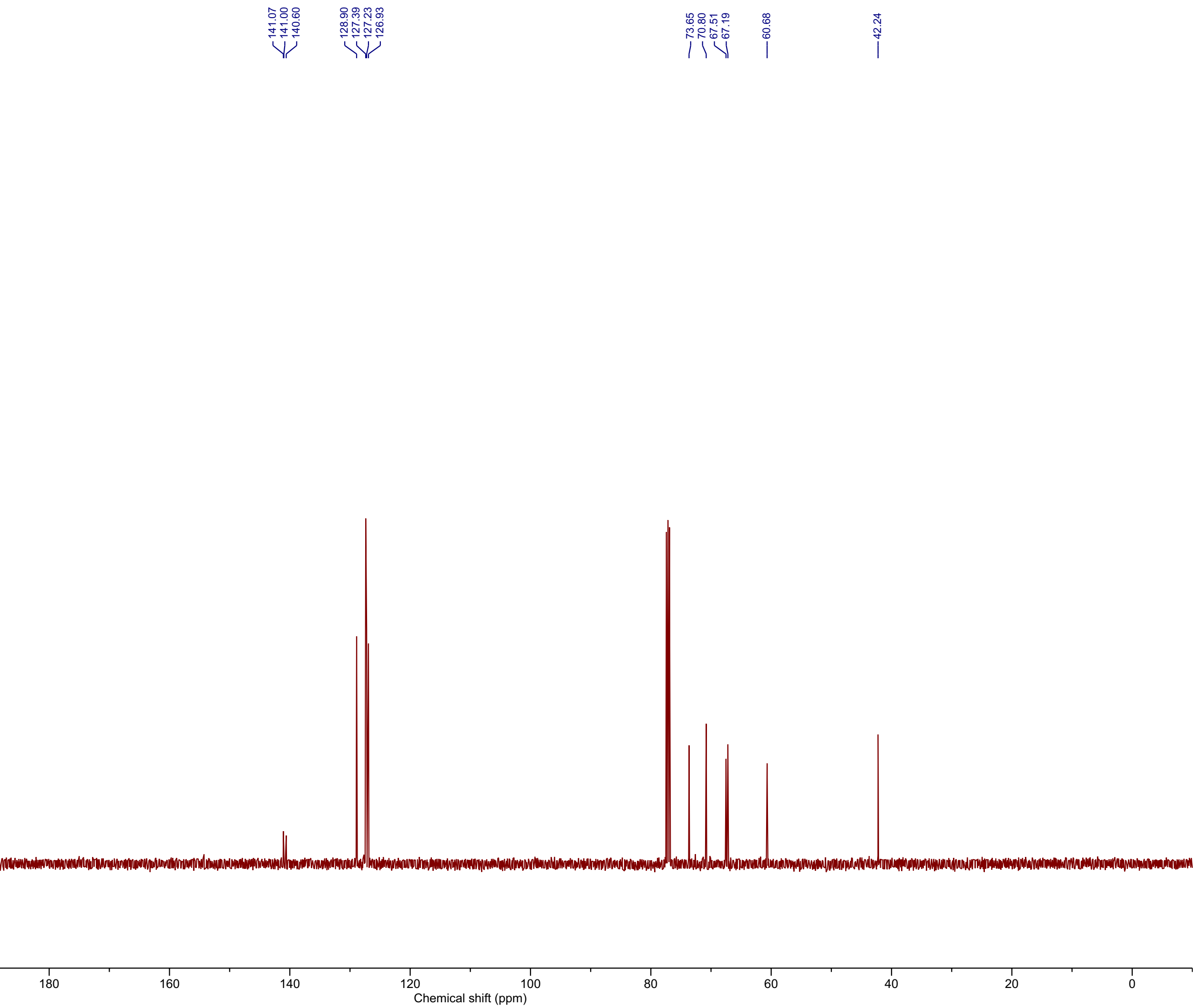

Supplementary Figure 112. ^{13}C NMR (126 MHz, $CDCl_3$) of **22**.

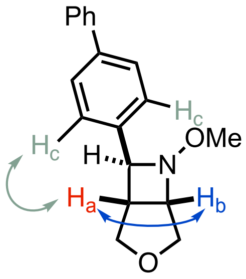

a Saturation of Ha (2.75 ppm)

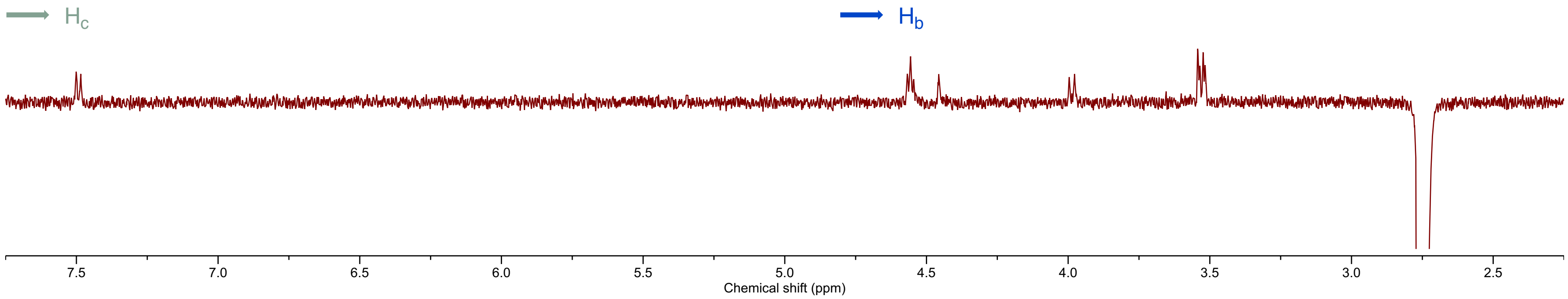

^{1}H NMR NOE (500 MHz, $CDCl_3$)

Supplementary Figure 113. ^{1}H NMR NOE of **22**. **a** Saturation of H_a at 2.75 ppm.

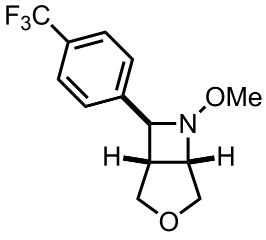

23

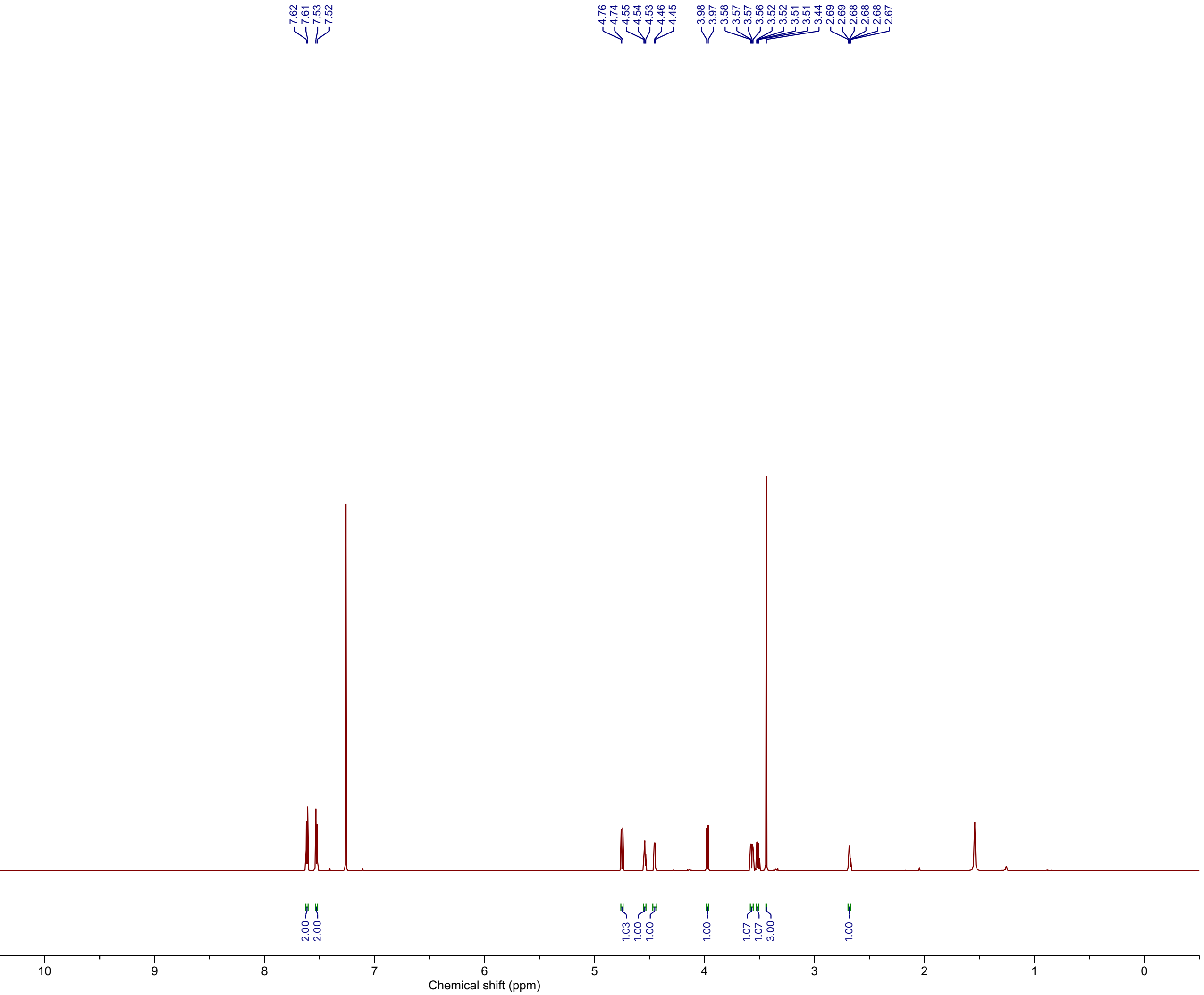

Supplementary Figure 114. ^{1}H NMR (700 MHz, $CDCl_3$) of **23**.

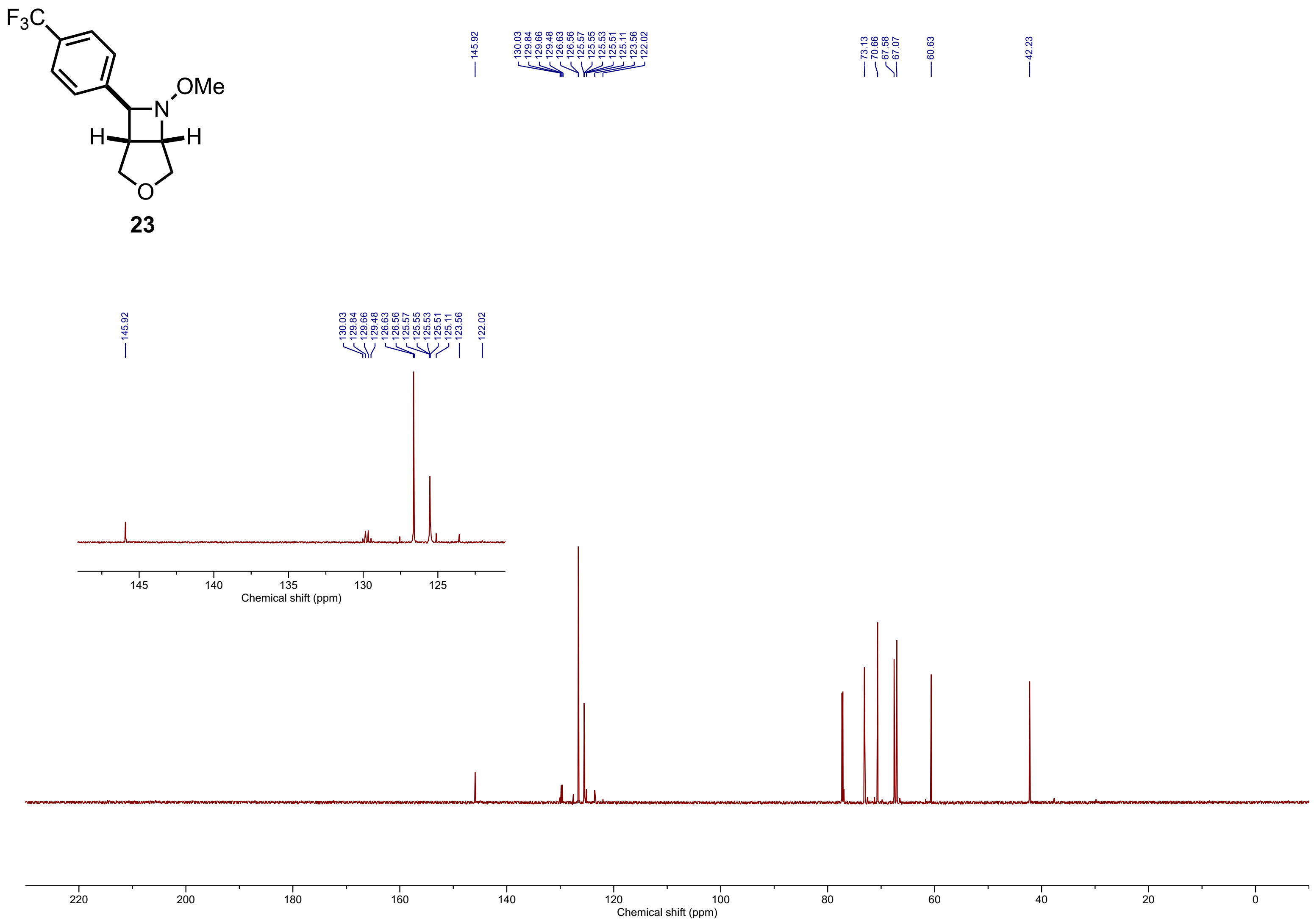

23

Supplementary Figure 115. ^{13}C NMR (176 MHz, $CDCl_3$) of **23**.

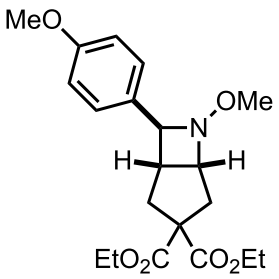

24

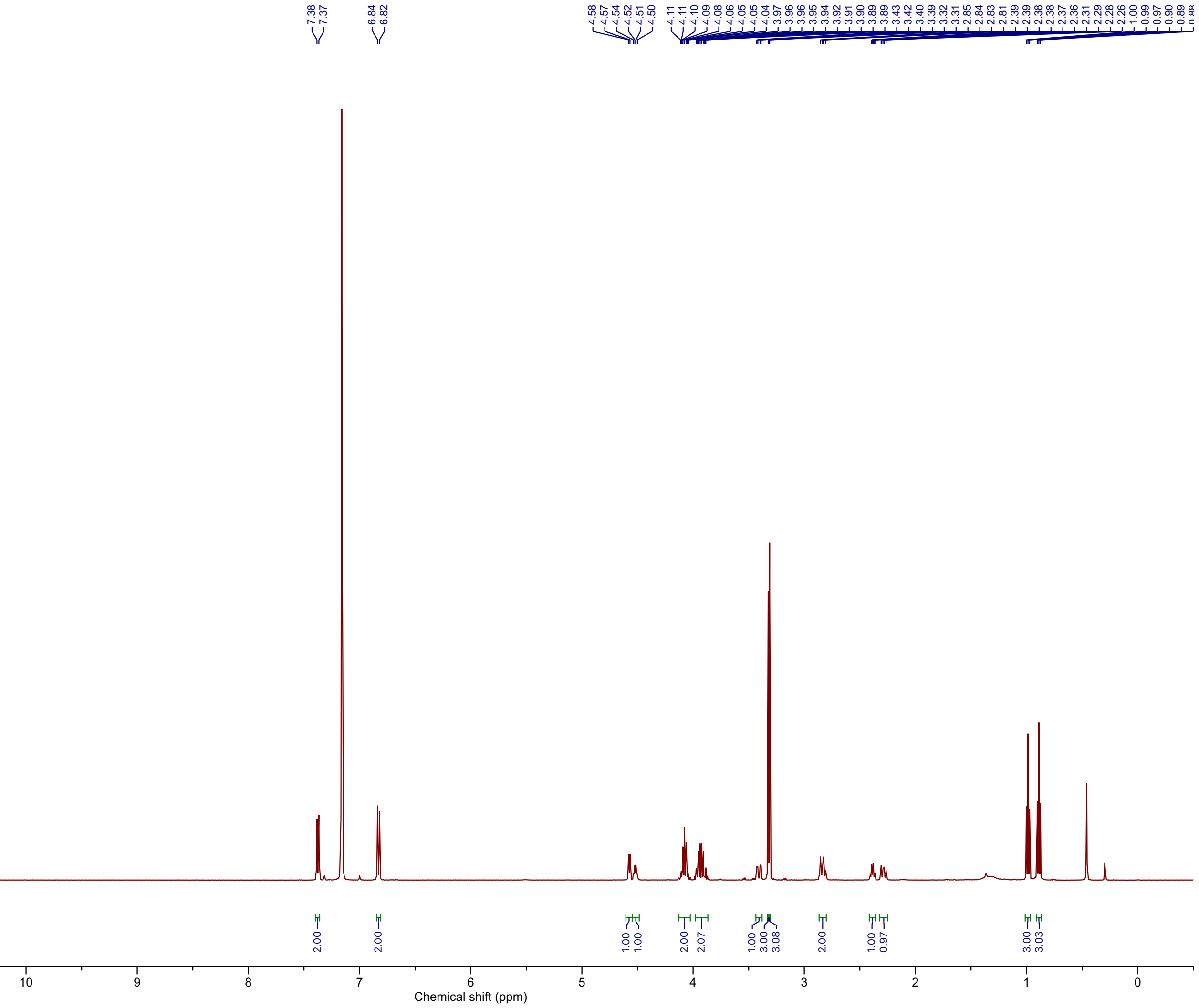

Supplementary Figure 116. ^{1}H NMR (400 MHz, C_6D_6) of **24**.

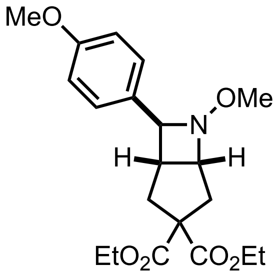

24

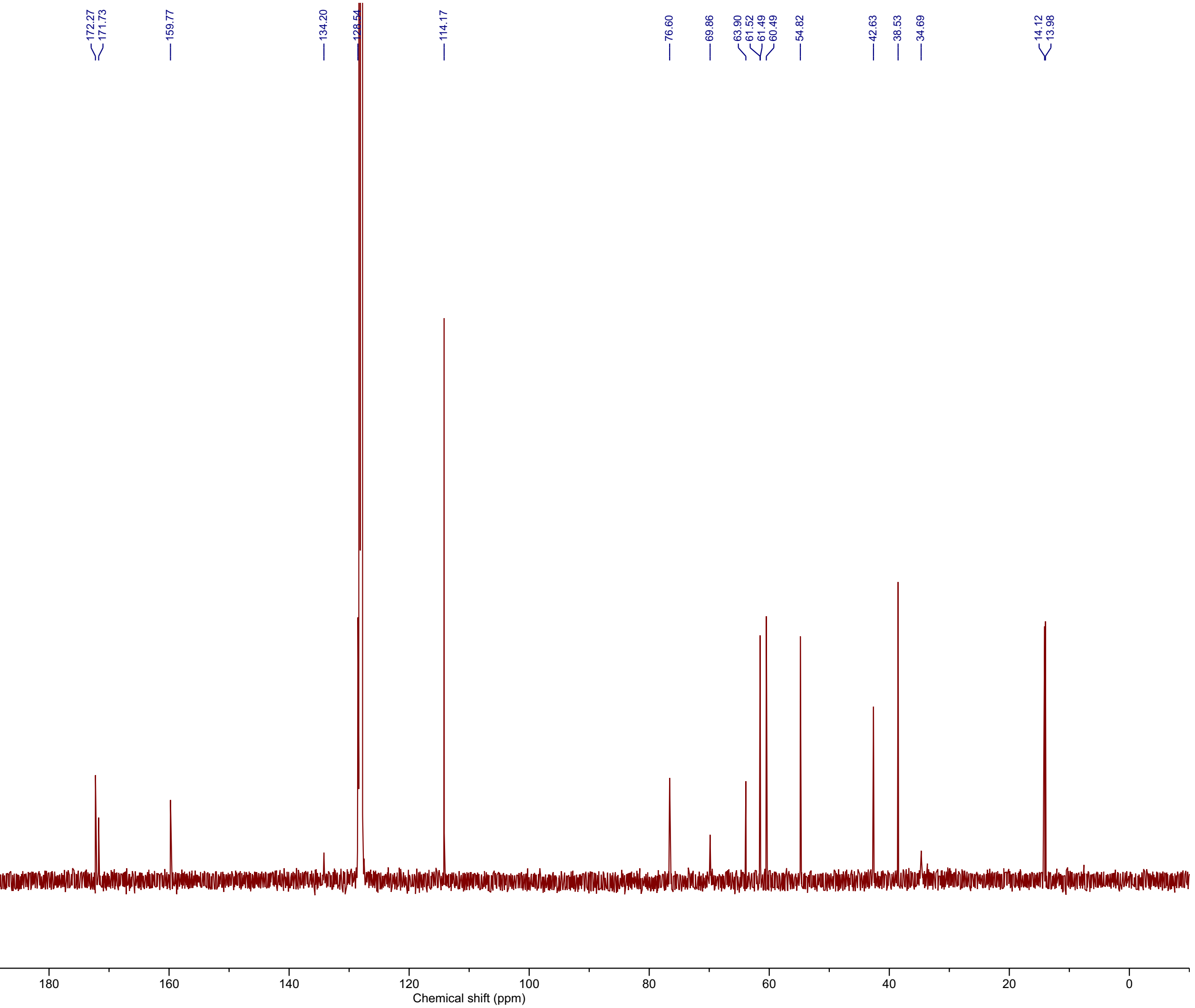

Supplementary Figure 117. ^{13}C NMR (126 MHz, C_6D_6) of **24**.

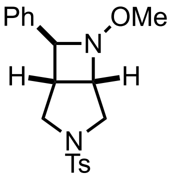

25

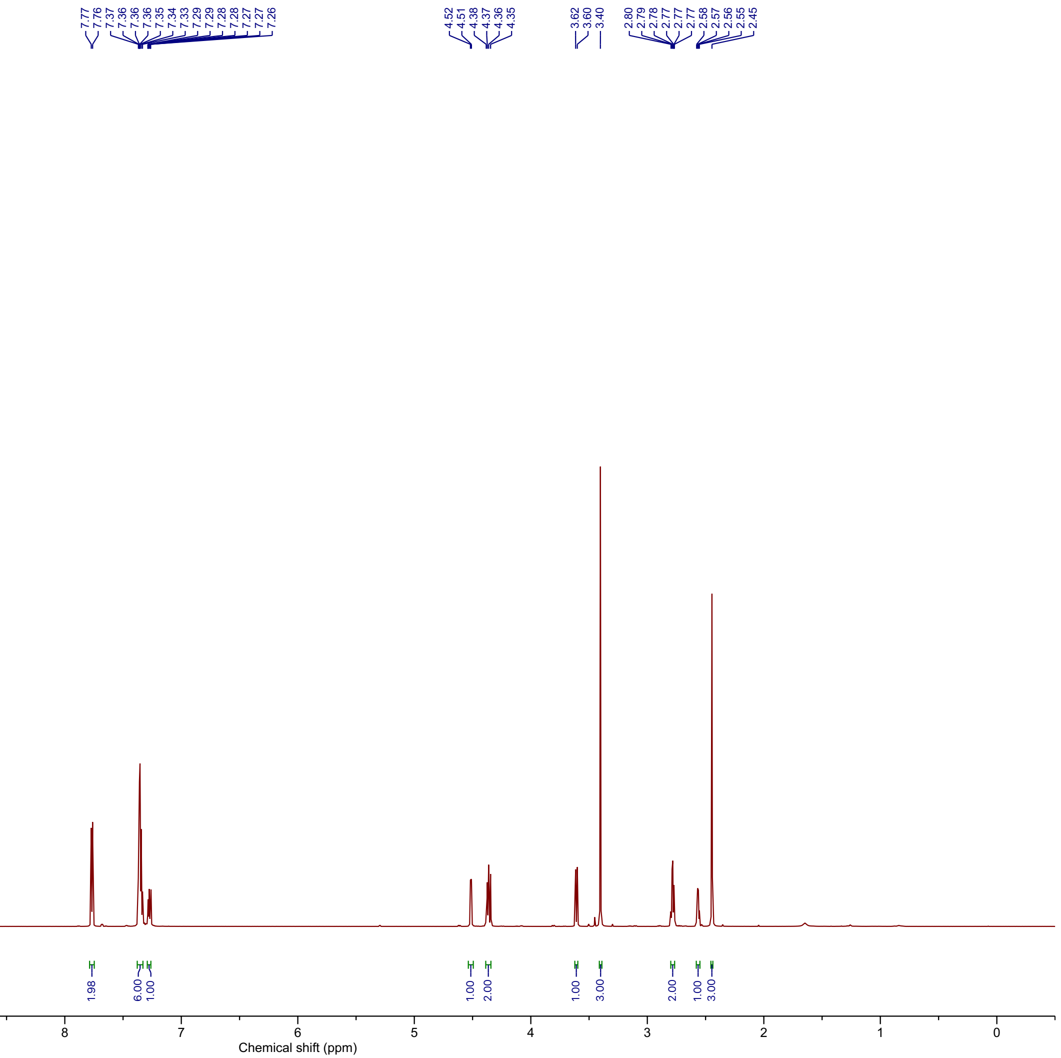

Supplementary Figure 118. ^{1}H NMR (700 MHz, $CDCl_3$) of **25**.

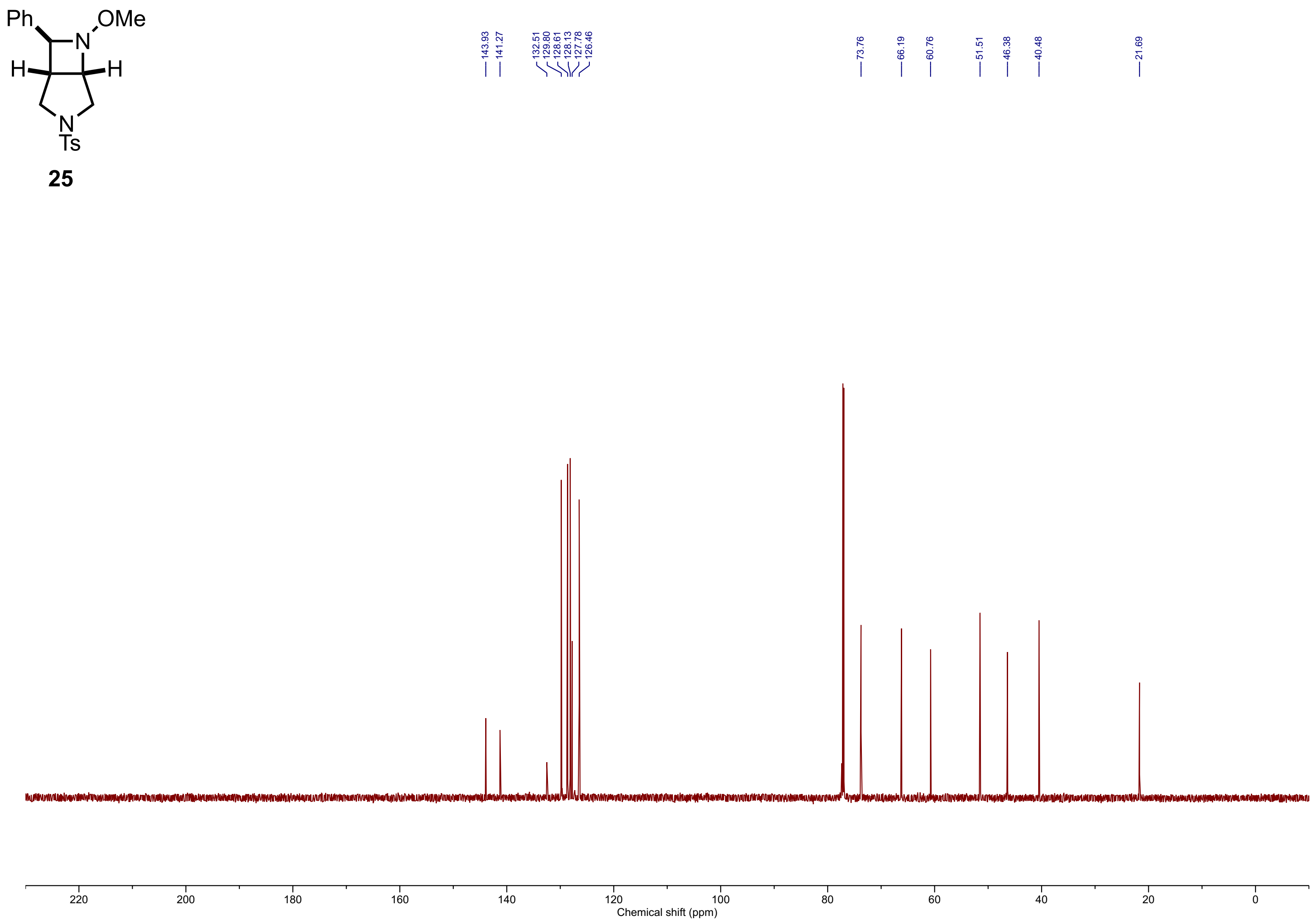

Supplementary Figure 119. ^{13}C NMR (176 MHz, $CDCl_3$) of **25**.

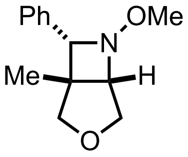

major
diastereomer

26

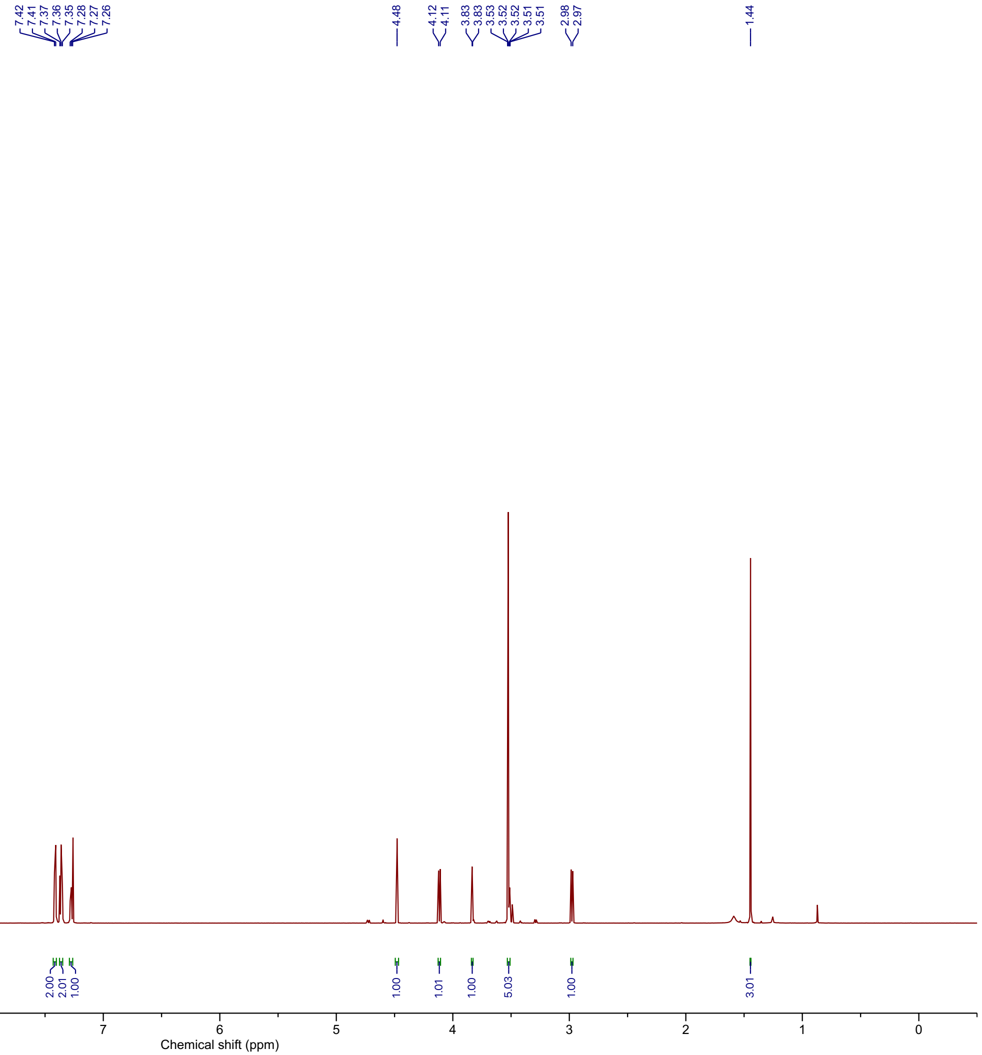

Supplementary Figure 120. ^{1}H NMR (700 MHz, $CDCl_3$) of **26** (major diastereomer)..

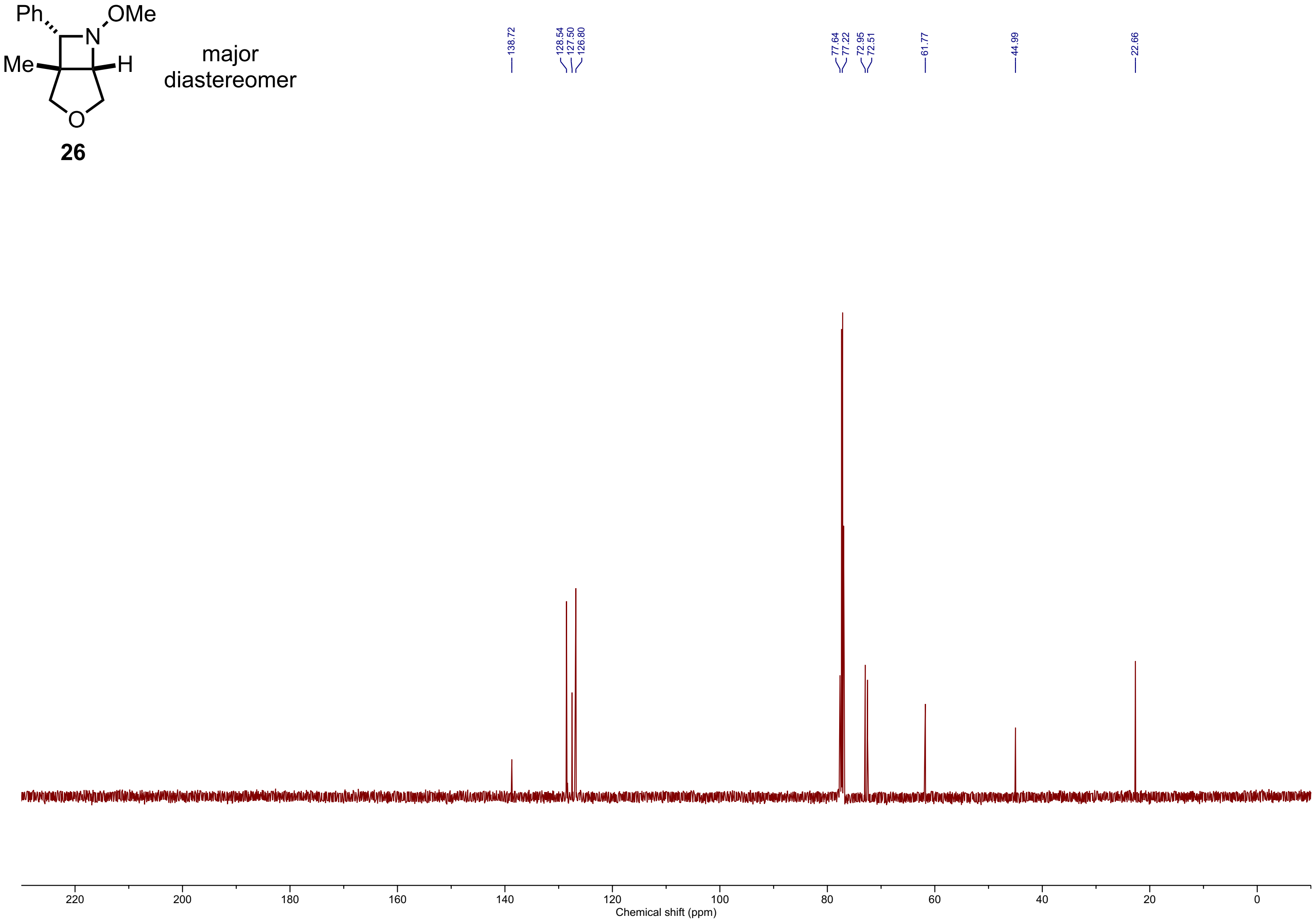

Supplementary Figure 121. ^{13}C NMR (176 MHz, $CDCl_3$) of **26** (major diastereomer).

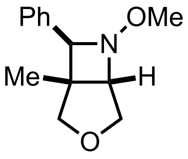

minor diastereomer

26

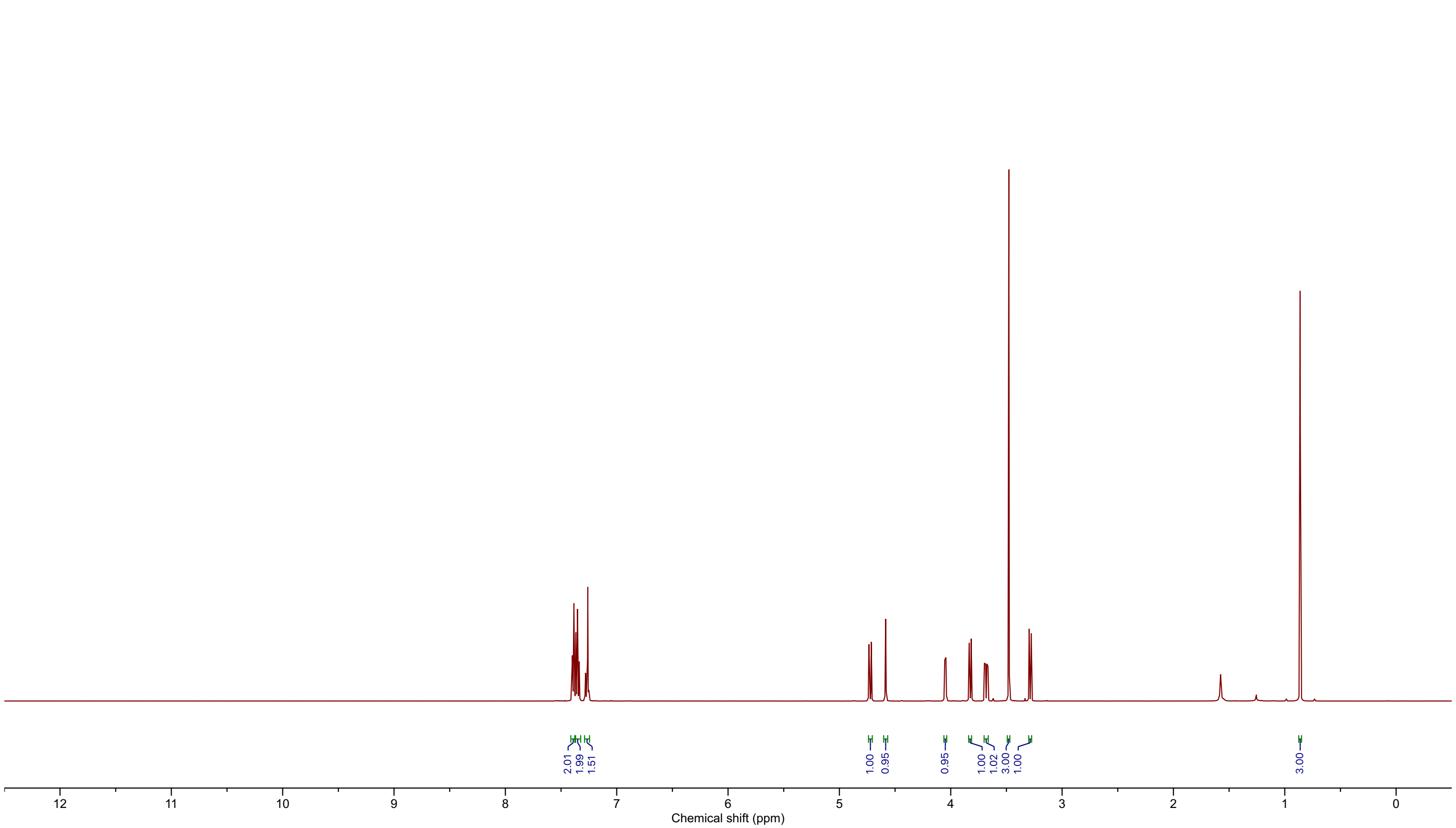

Supplementary Figure 122. ^{1}H NMR (500 MHz, $CDCl_3$) of **26** (minor diastereomer).

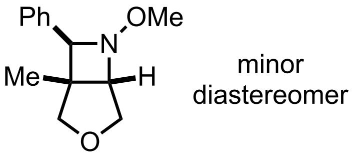

26

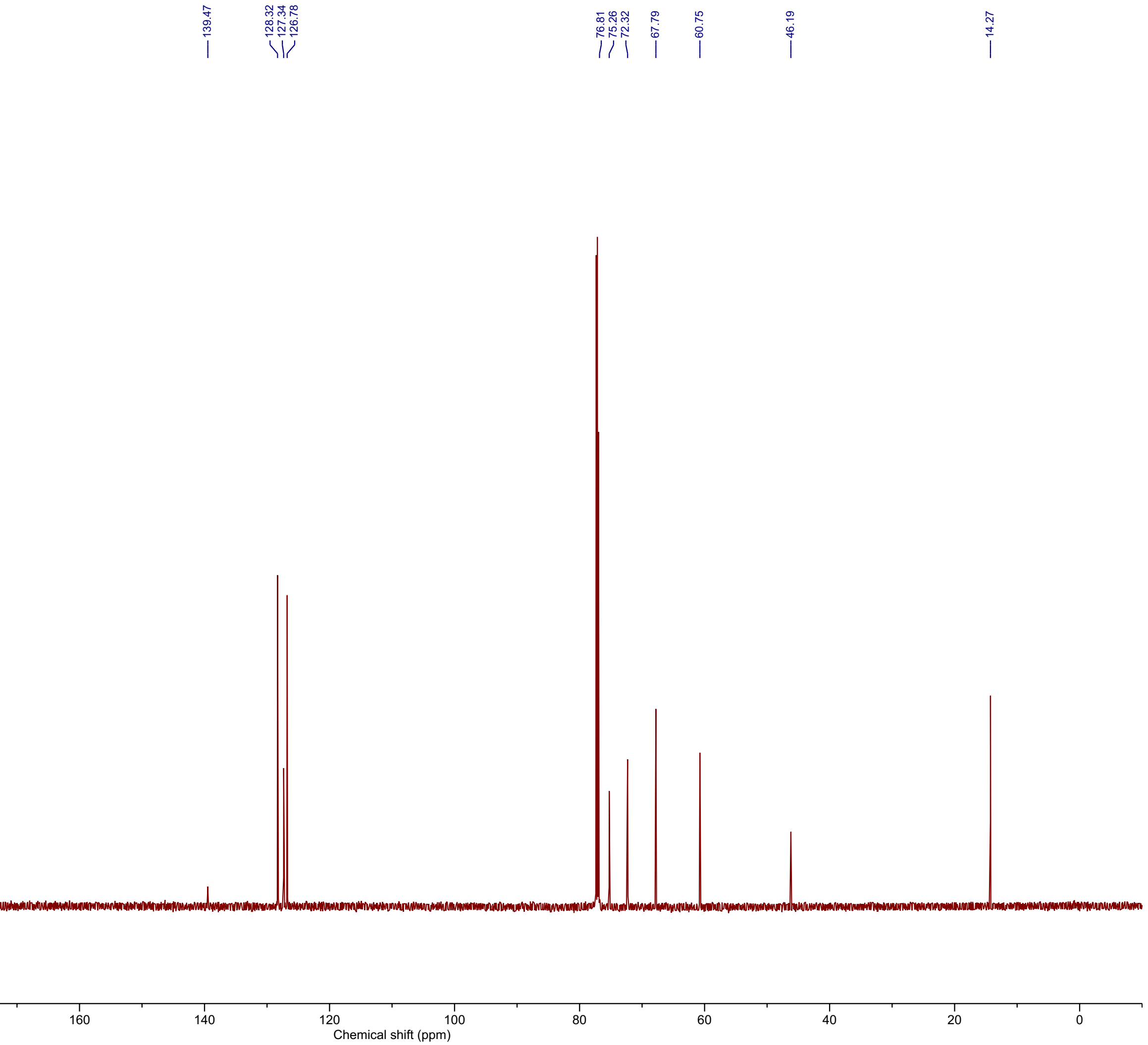

Supplementary Figure 123. ^{13}C NMR (176 MHz, $CDCl_3$) of **26** (minor diastereomer).

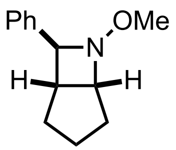

27

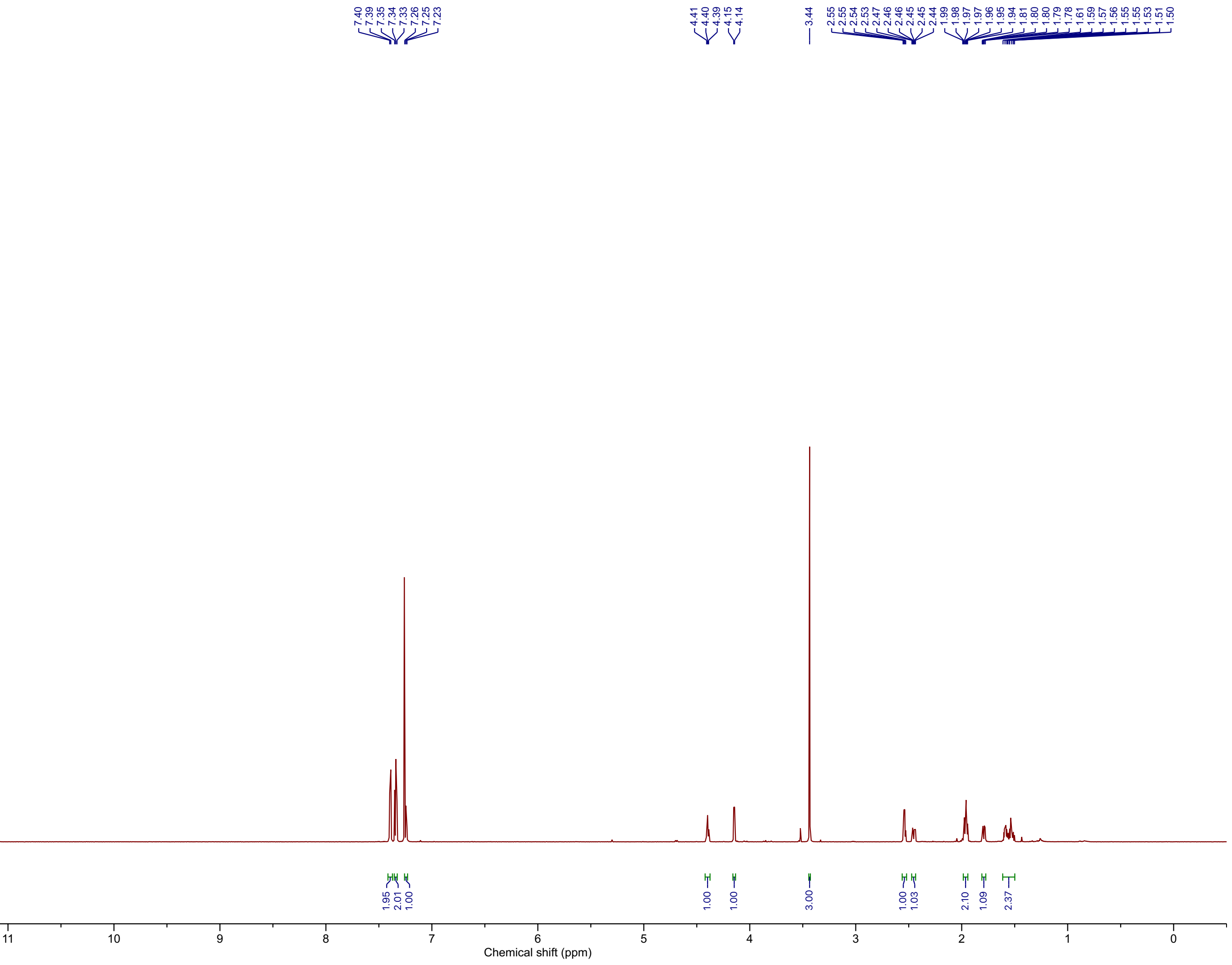

Supplementary Figure 124. ^{1}H NMR (700 MHz, $CDCl_3$) of **27**.

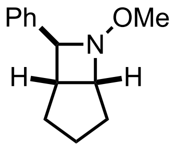

27

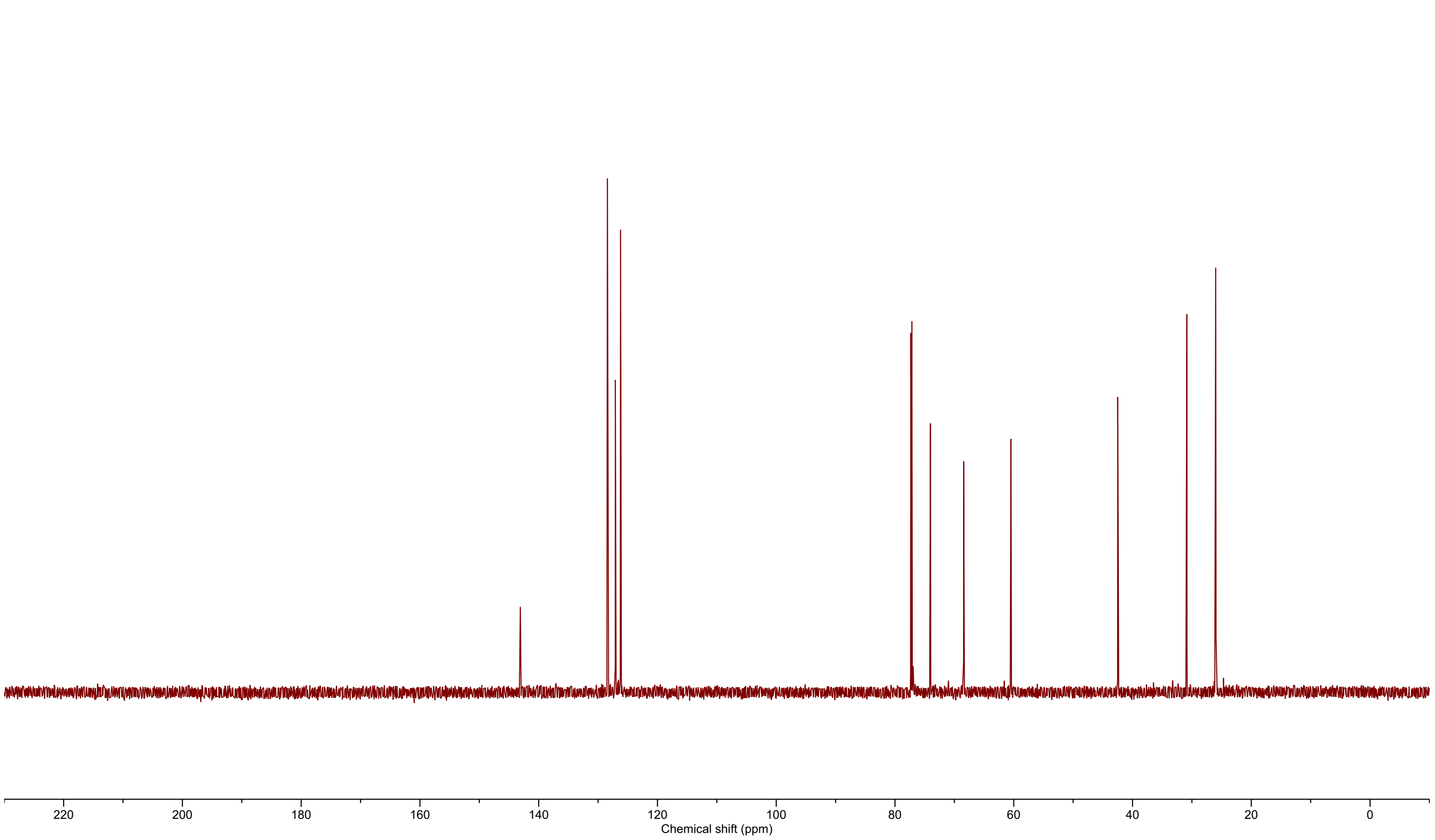

Supplementary Figure 125. ^{13}C NMR (176 MHz, $CDCl_3$) of **27**.

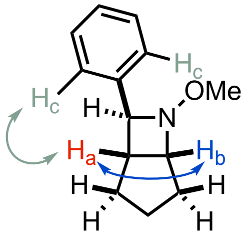

a Saturation of Ha (2.55 ppm)

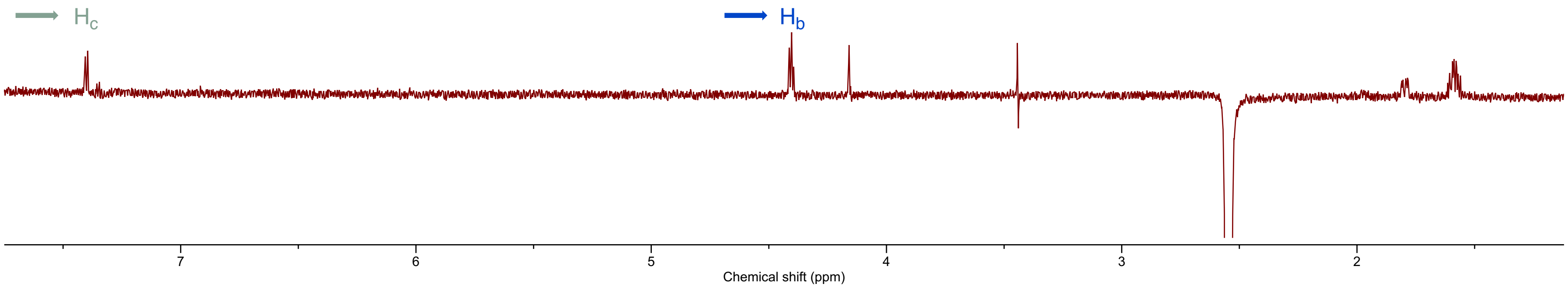

^{1}H NMR NOE (700 MHz, $CDCl_3$)

Supplementary Figure 126. ^{1}H NMR NOE of **27**. **a** Saturation of H_a at 2.55 ppm.

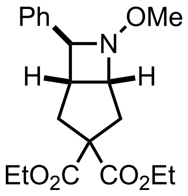

28

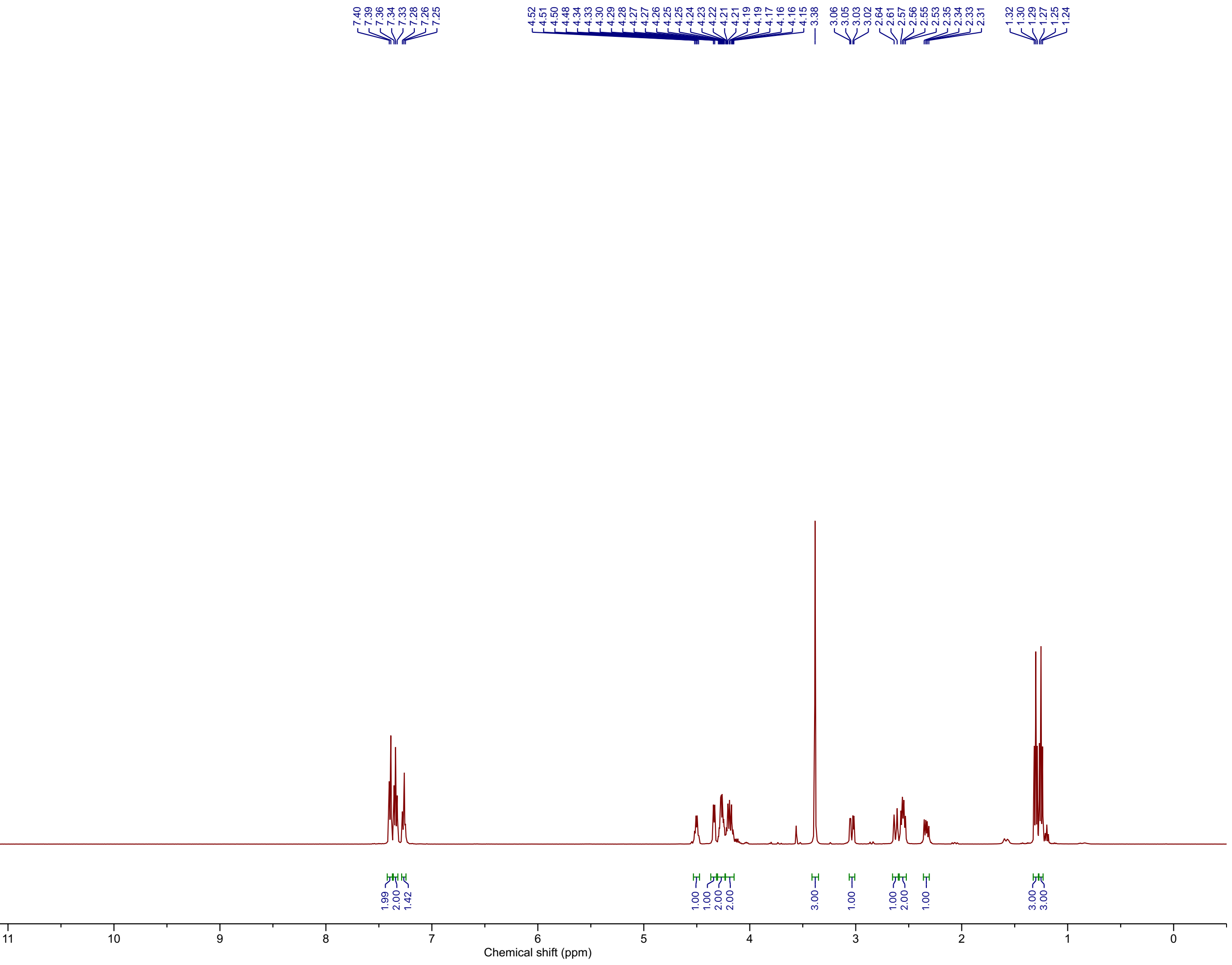

Supplementary Figure 127. ^{1}H NMR (500 MHz, $CDCl_3$) of **28**.

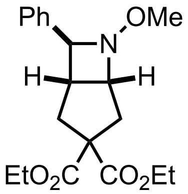

28

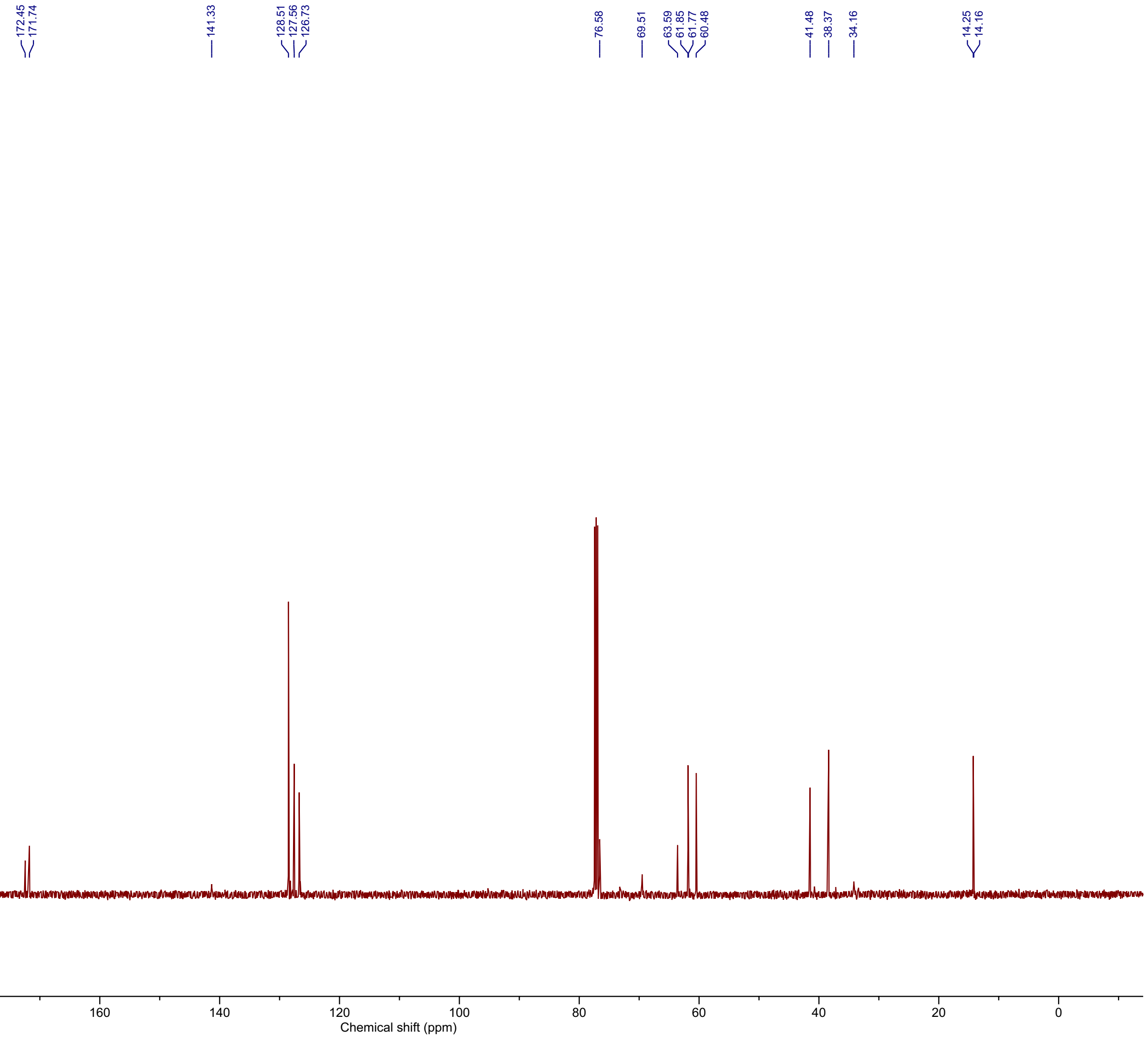

Supplementary Figure 128. ^{13}C NMR (126 MHz, $CDCl_3$) of **28**.

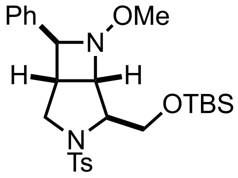

29

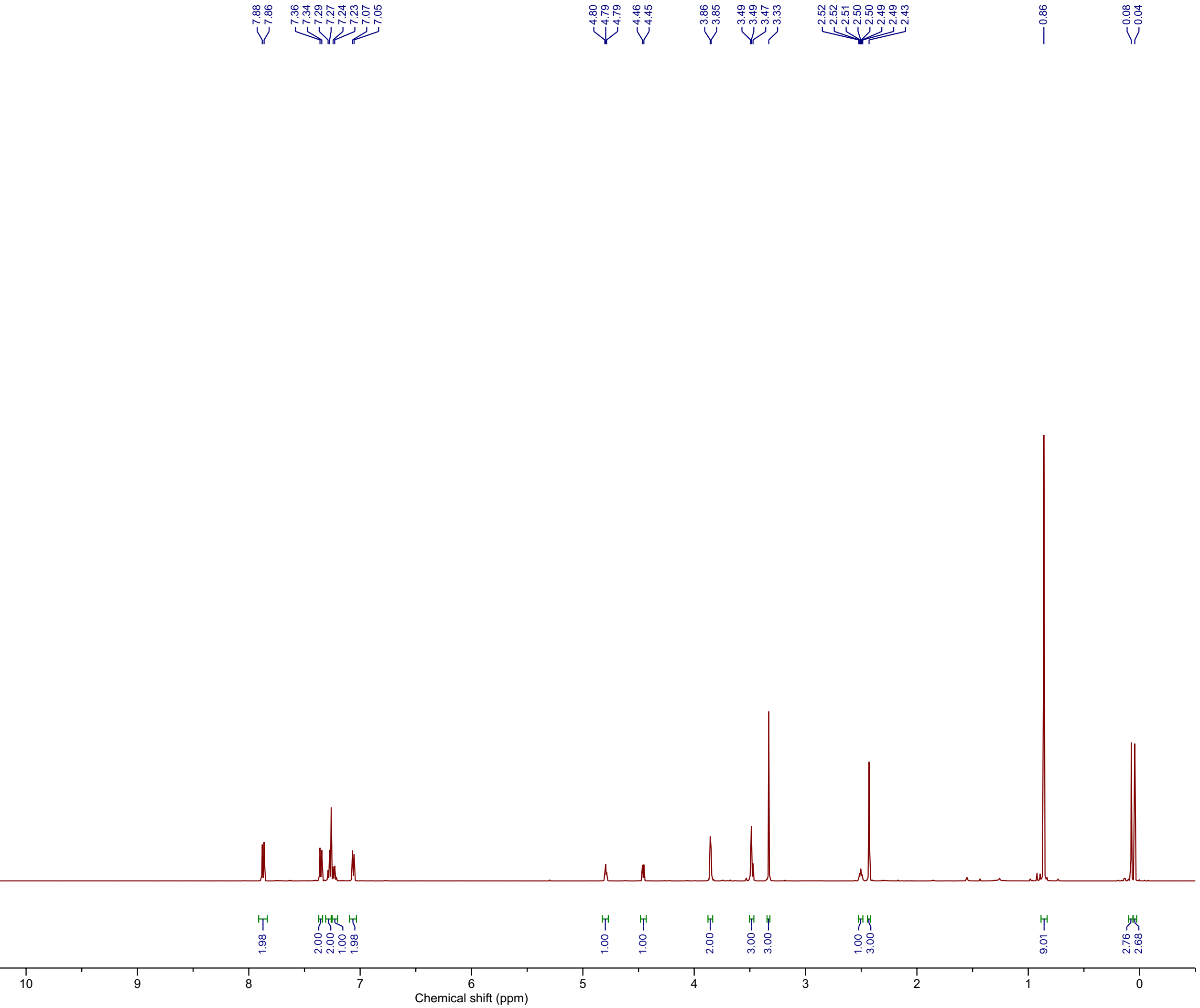

Supplementary Figure 129. ^{1}H NMR (500 MHz, $CDCl_3$) of **29**.

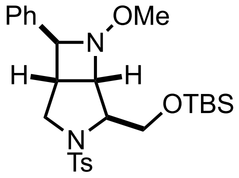

29

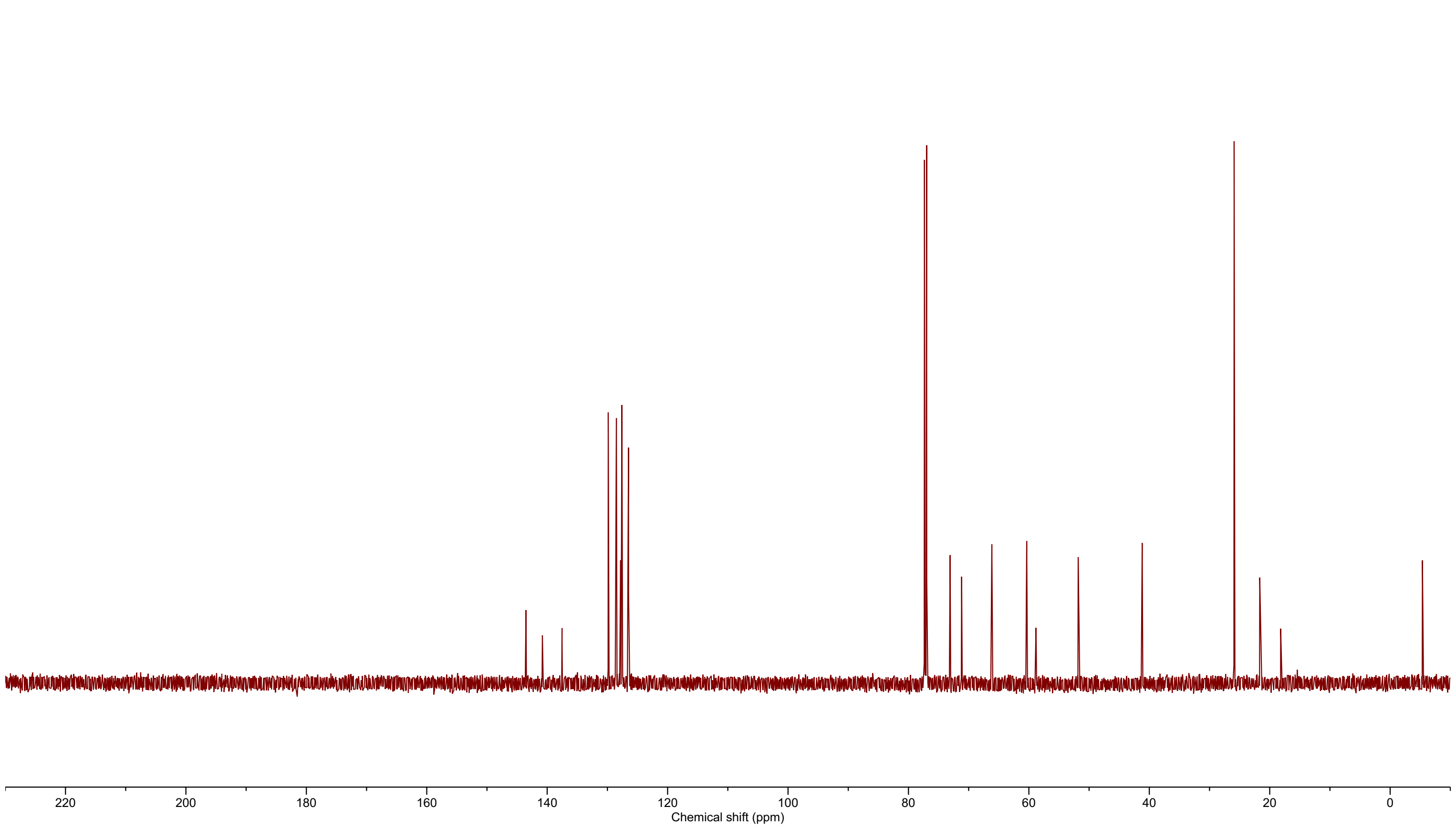

Supplementary Figure 130. ^{13}C NMR (176 MHz, $CDCl_3$) of **29**.

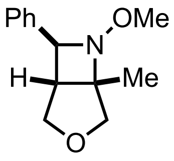

30

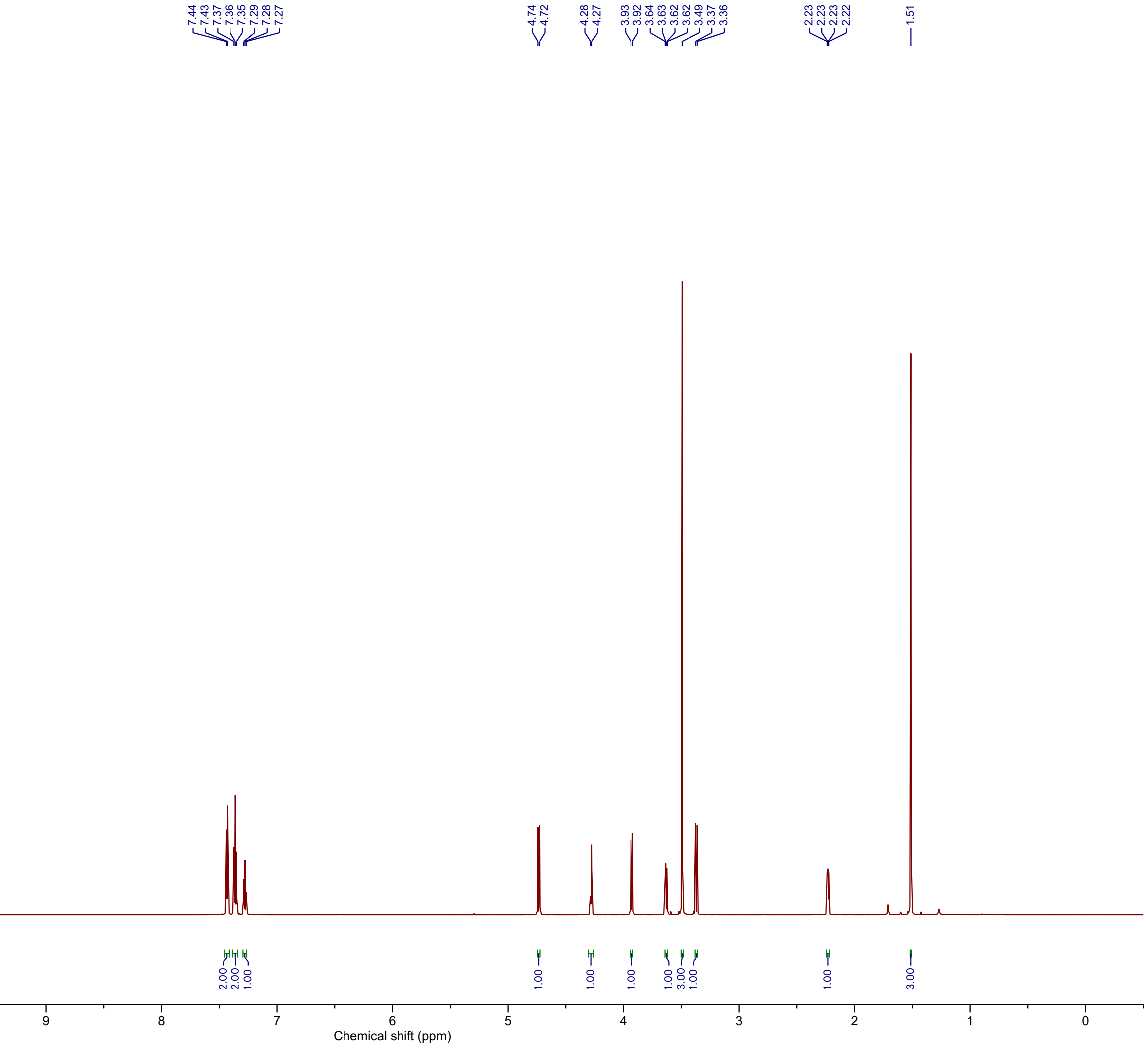

Supplementary Figure 131. ^{1}H NMR (700 MHz, $CDCl_3$) of **30**.

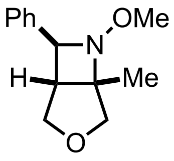

30

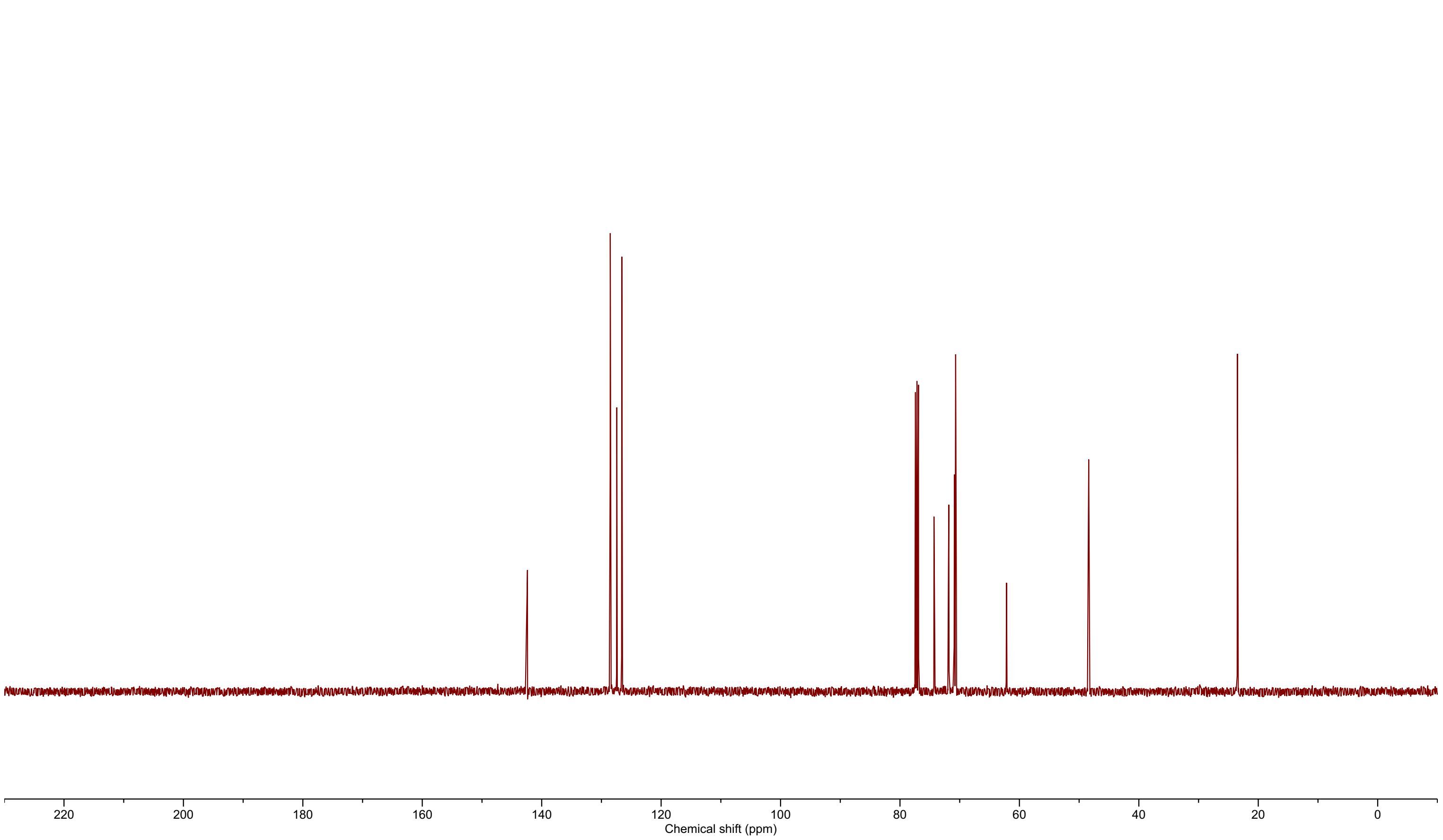

Supplementary Figure 132. ^{13}C NMR (126 MHz, $CDCl_3$) of **30**.

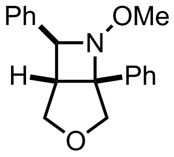

31

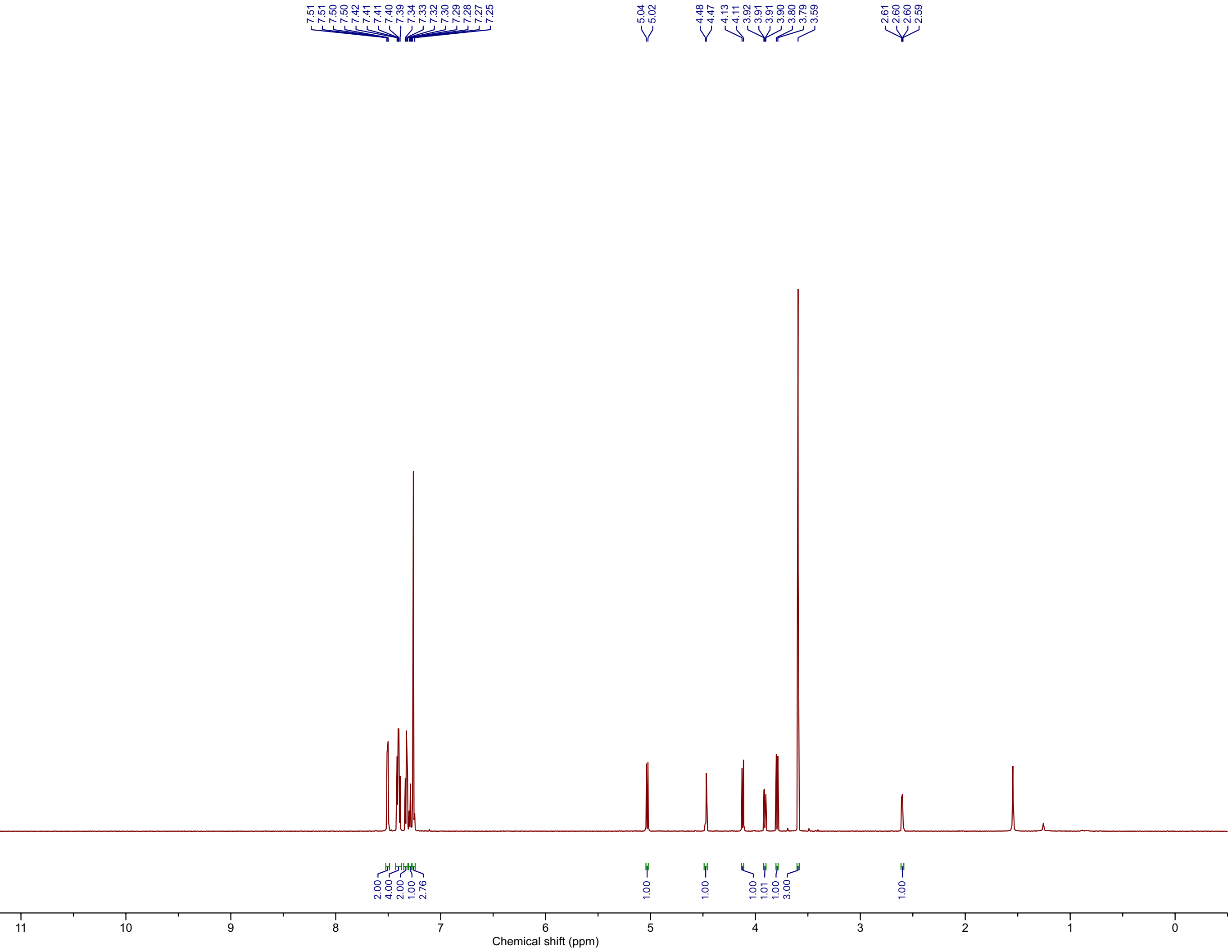

Supplementary Figure 133. ^{1}H NMR (700 MHz, $CDCl_3$) of **31**.

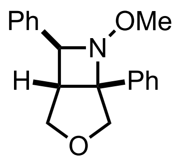

31

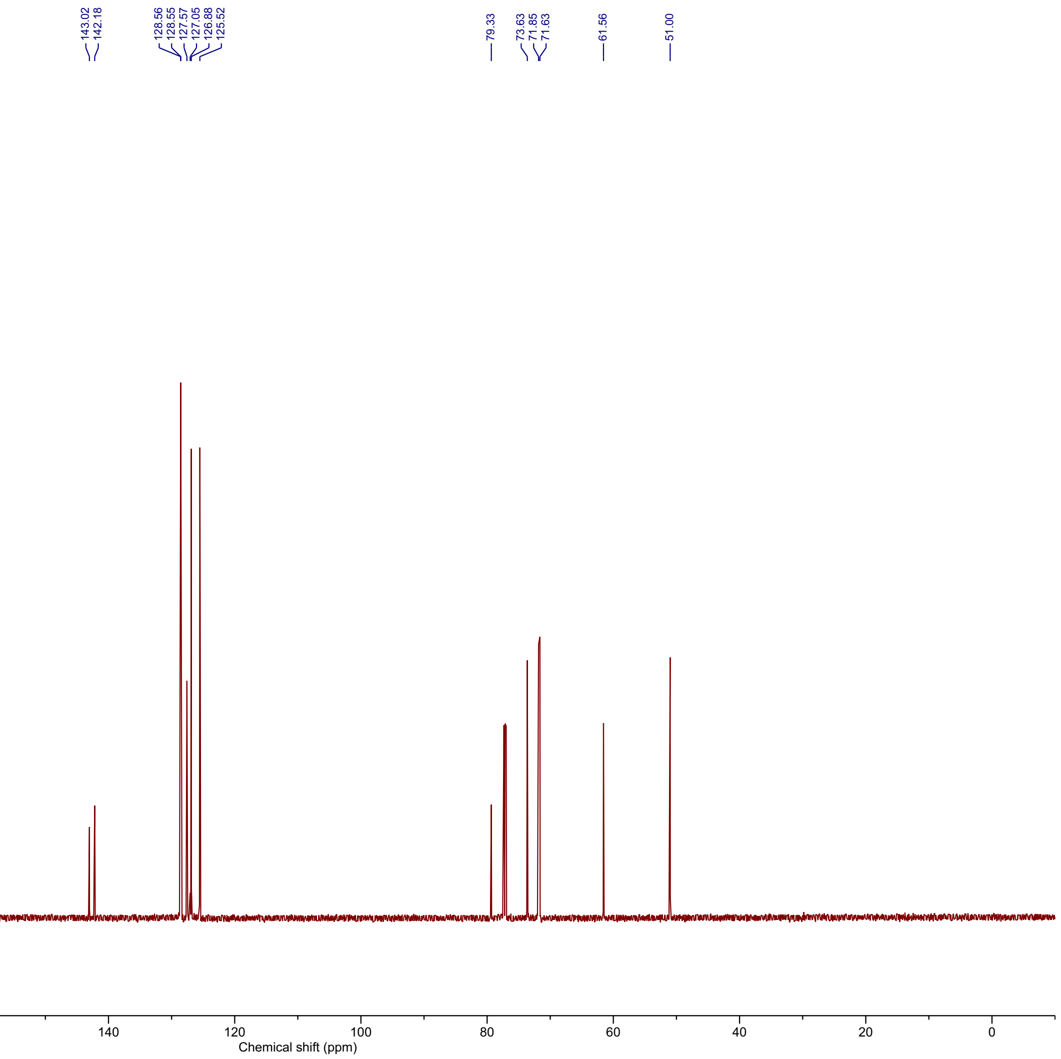

Supplementary Figure 134. ^{13}C NMR (176 MHz, $CDCl_3$) of **31**.

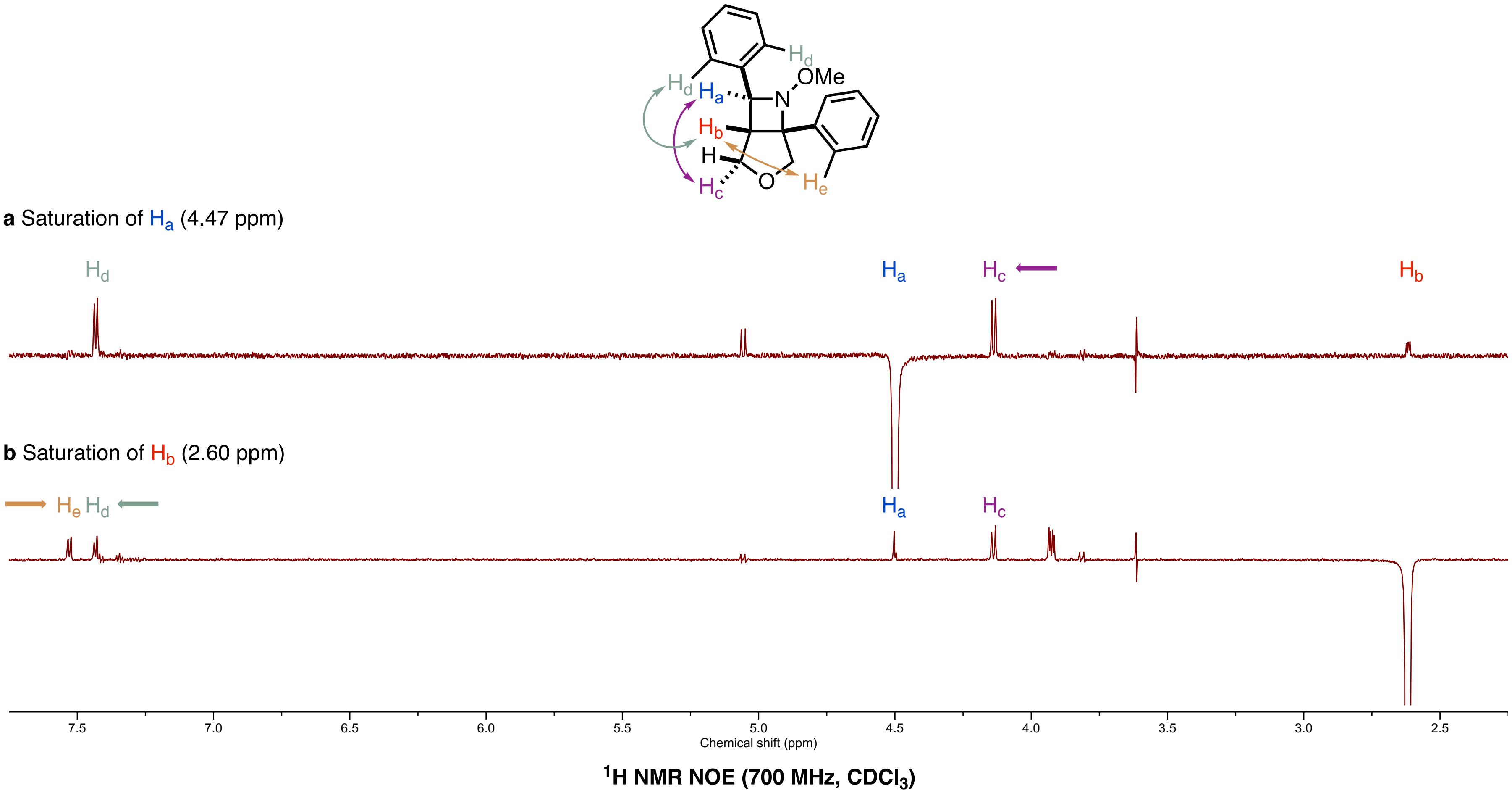

Supplementary Figure 135. ^{1}H NMR NOE of **31**. **a** Saturation of H_a at 4.47 ppm **b** Saturation of H_b at 2.60 ppm.

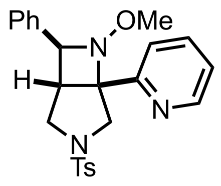

32

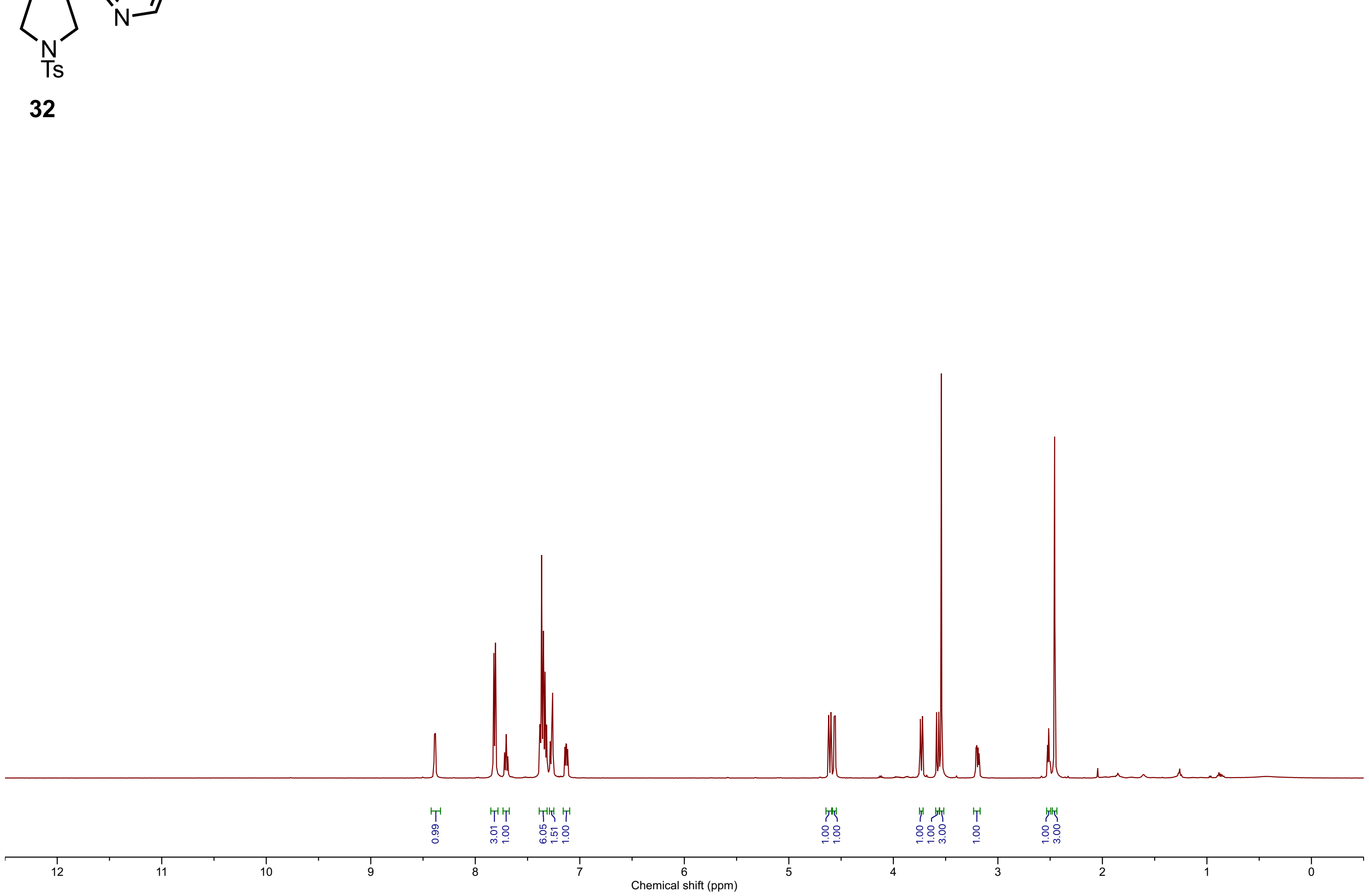

Supplementary Figure 136. ^{1}H NMR (500 MHz, $CDCl_3$) of **32**.

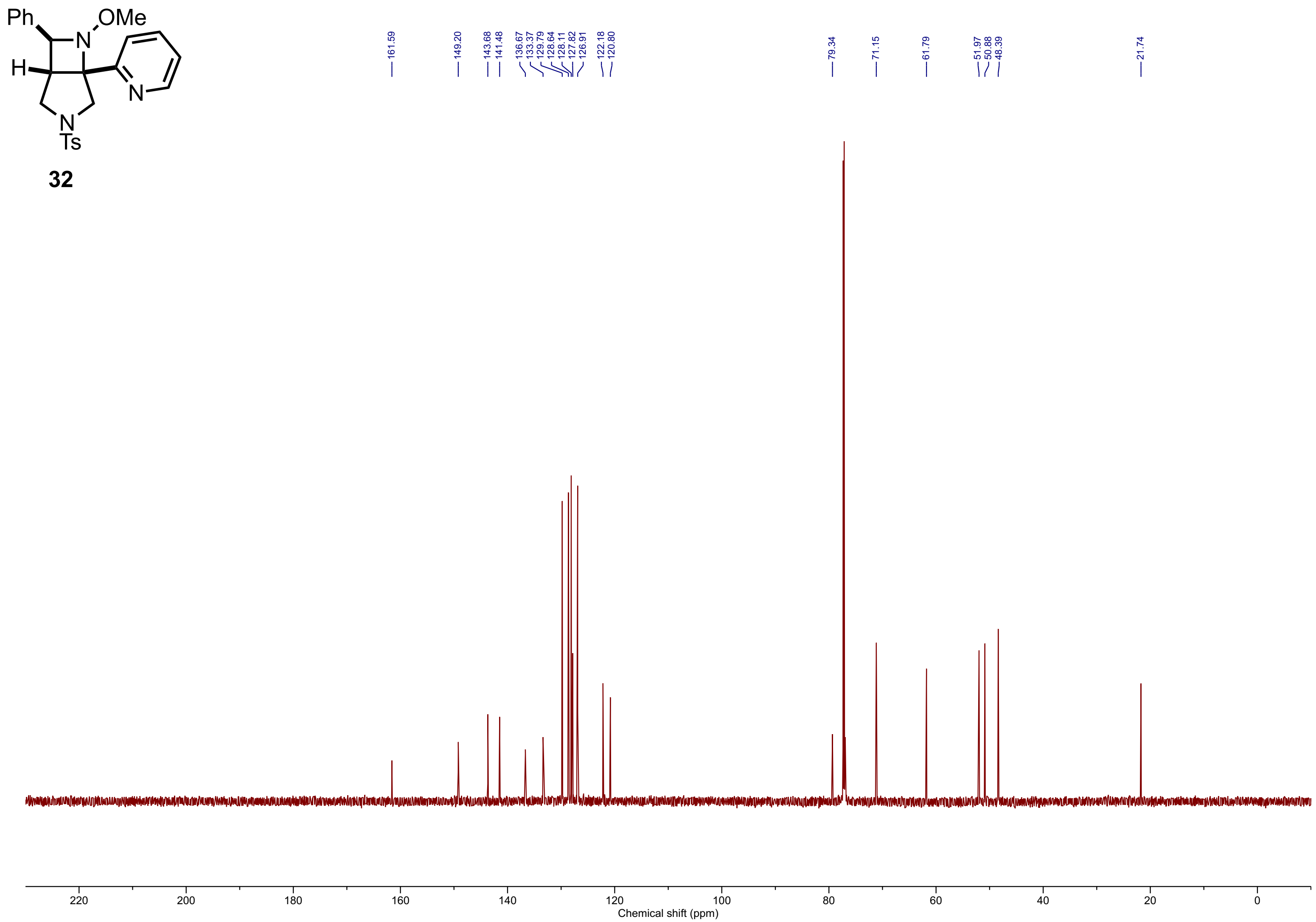

Supplementary Figure 137. ^{13}C NMR (176 MHz, $CDCl_3$) of **32**.

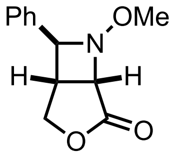

33

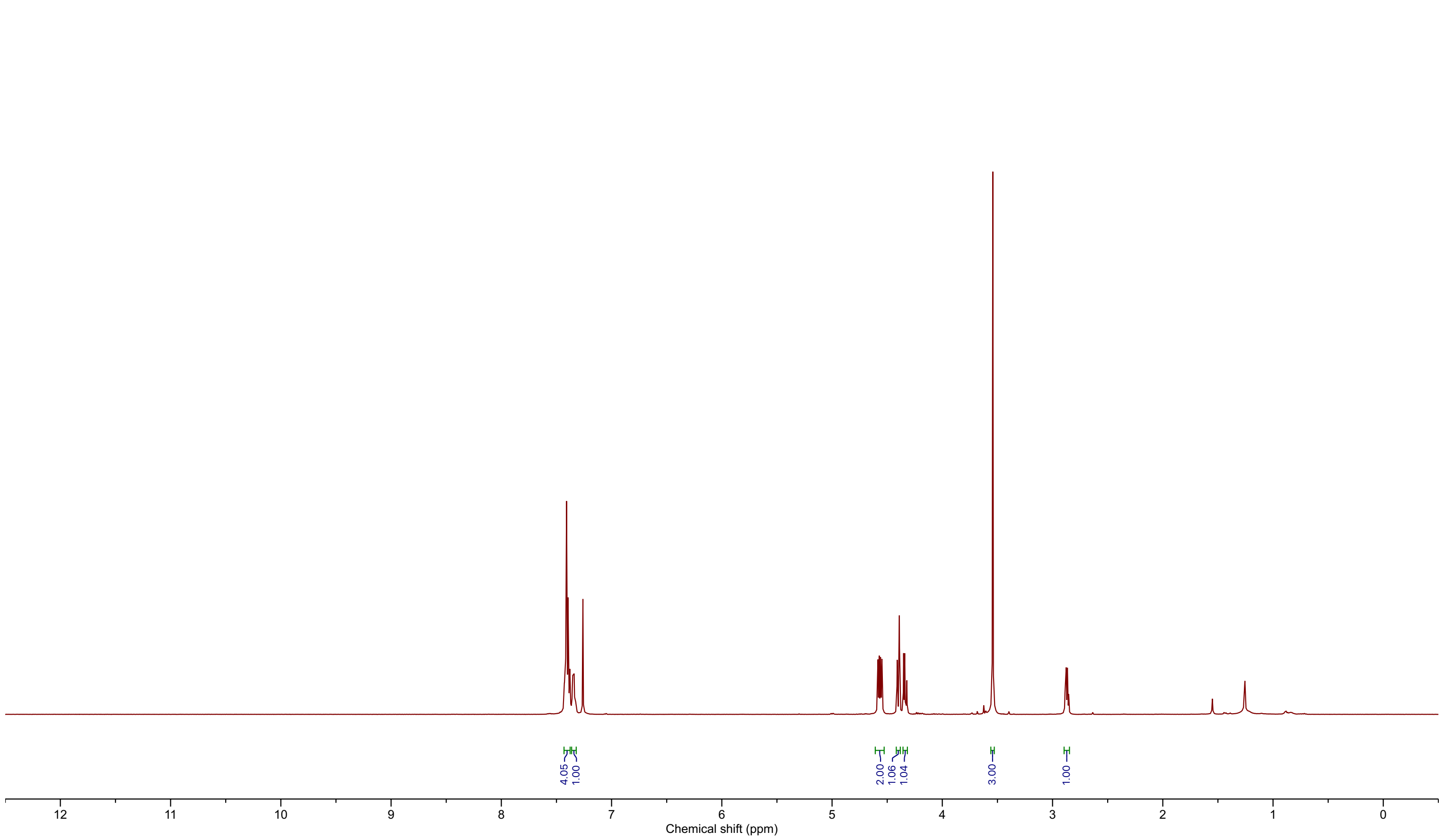

Supplementary Figure 138. ^{1}H NMR (500 MHz, $CDCl_3$) of **33**.

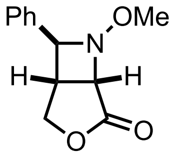

33

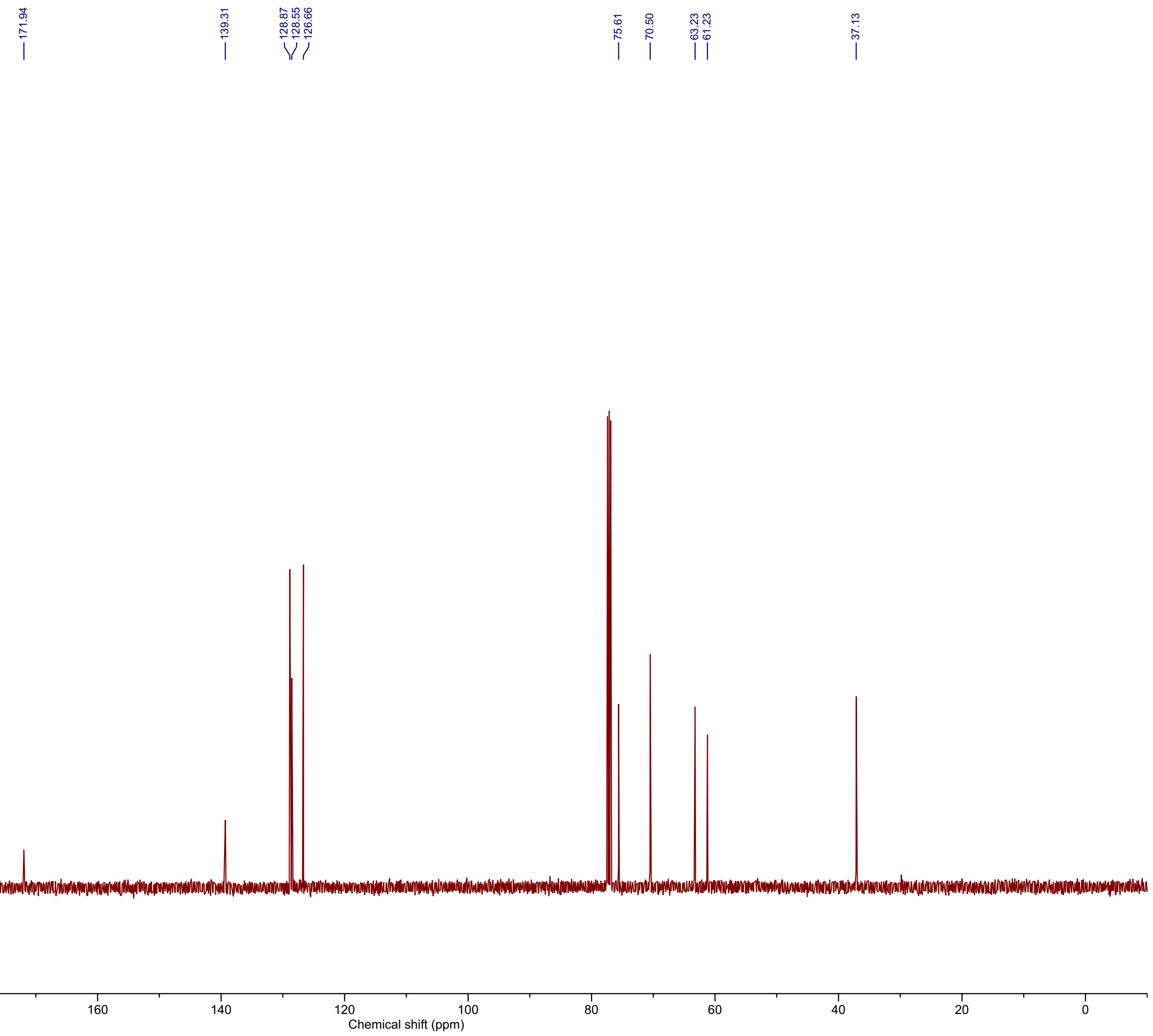

Supplementary Figure 139. ^{13}C NMR (126 MHz, $CDCl_3$) of **33**.

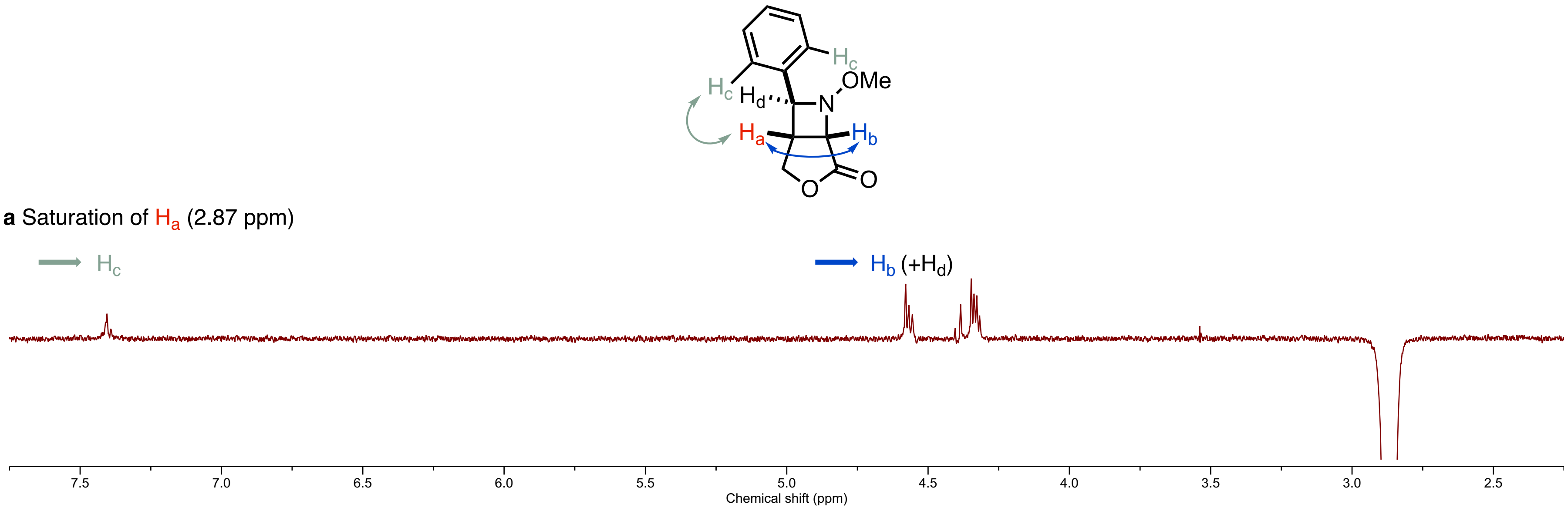

^{1}H NMR NOE (700 MHz, $CDCl_3$)

Supplementary Figure 140. ^{1}H NMR NOE of **33**. **a** Saturation of H_a at 2.87 ppm.

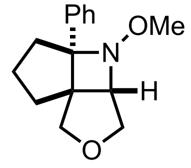

34

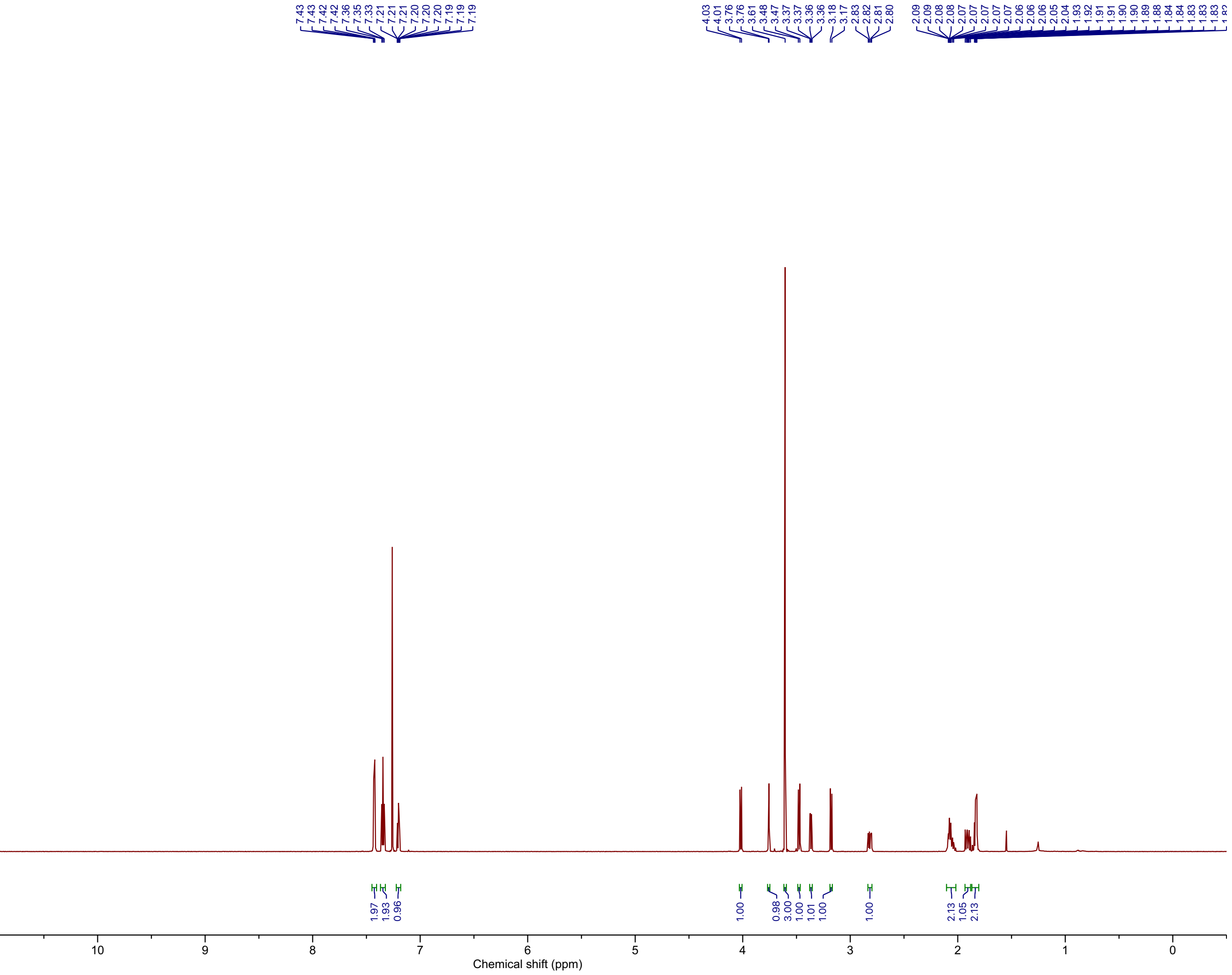

Supplementary Figure 141. ^{1}H NMR (700 MHz, $CDCl_3$) of **34**.

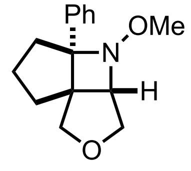

34

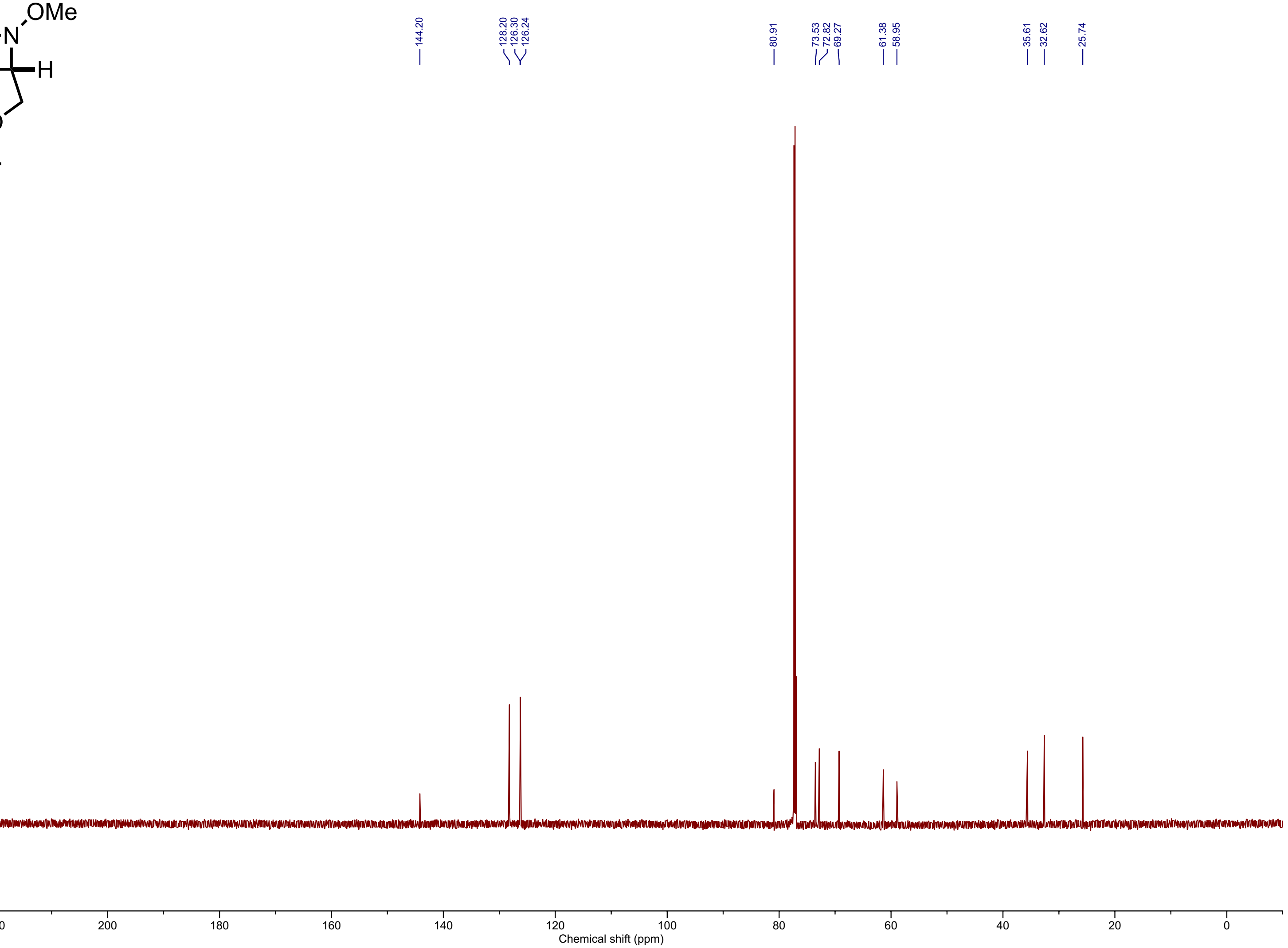

Supplementary Figure 142. ^{13}C NMR (176 MHz, $CDCl_3$) of **34**.

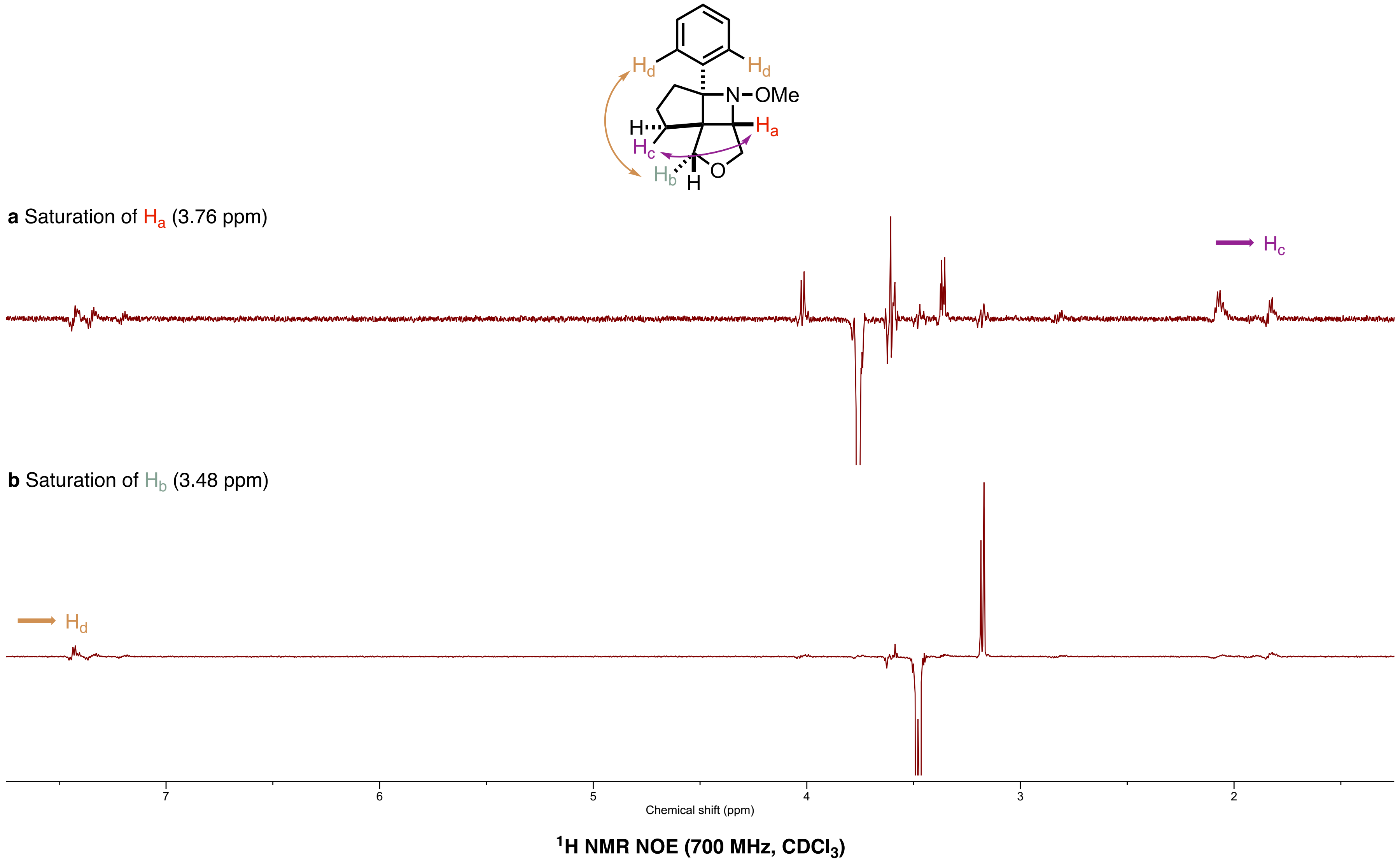

Supplementary Figure 143. ^{1}H NMR NOE of **34**. **a** Saturation of H_a at 3.76 ppm; **b** Saturation of H_b at 3.48 ppm.

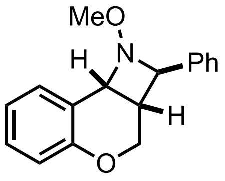

35

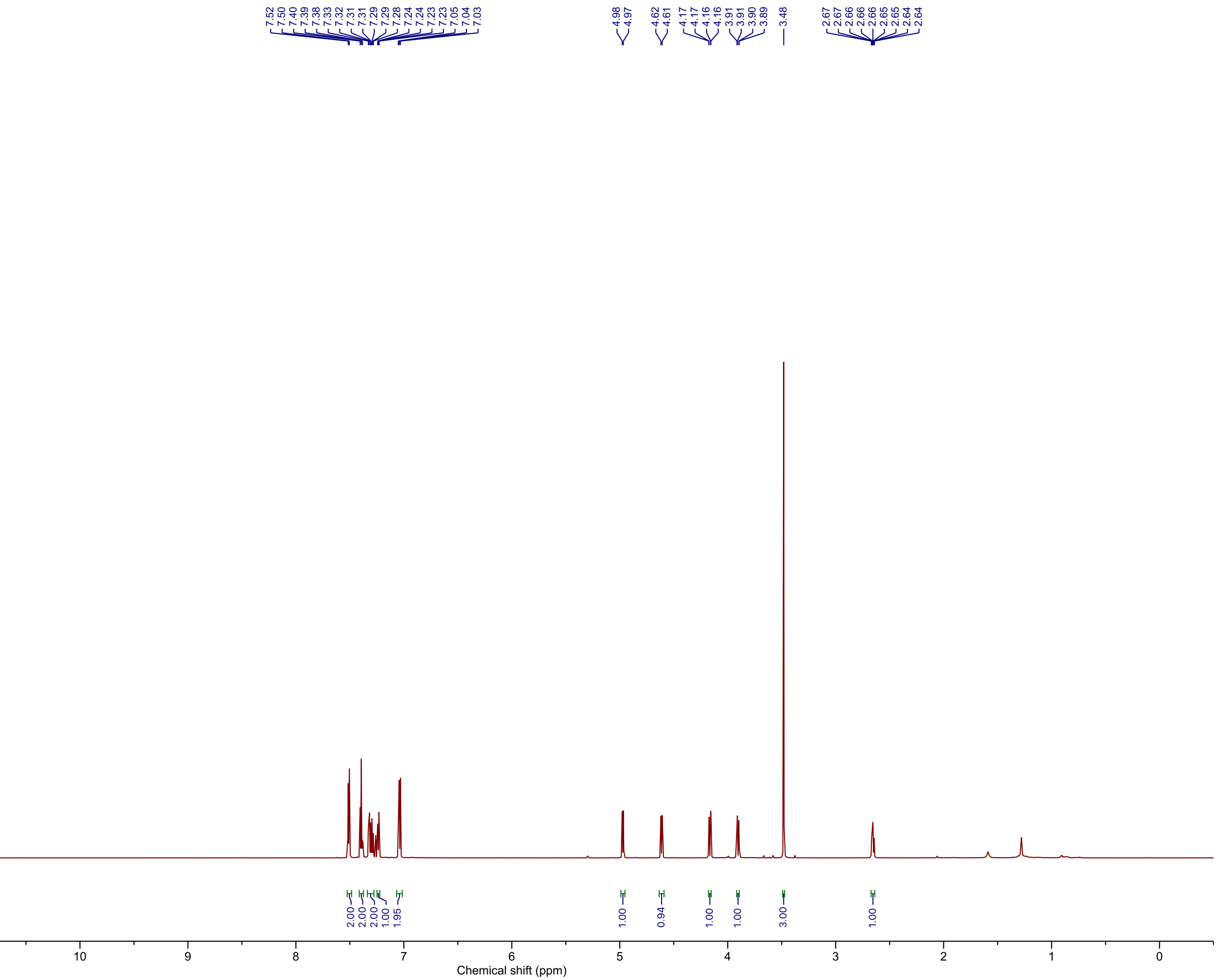

Supplementary Figure 144. ^{1}H NMR (700 MHz, $CDCl_3$) of **35**.

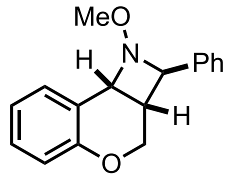

35

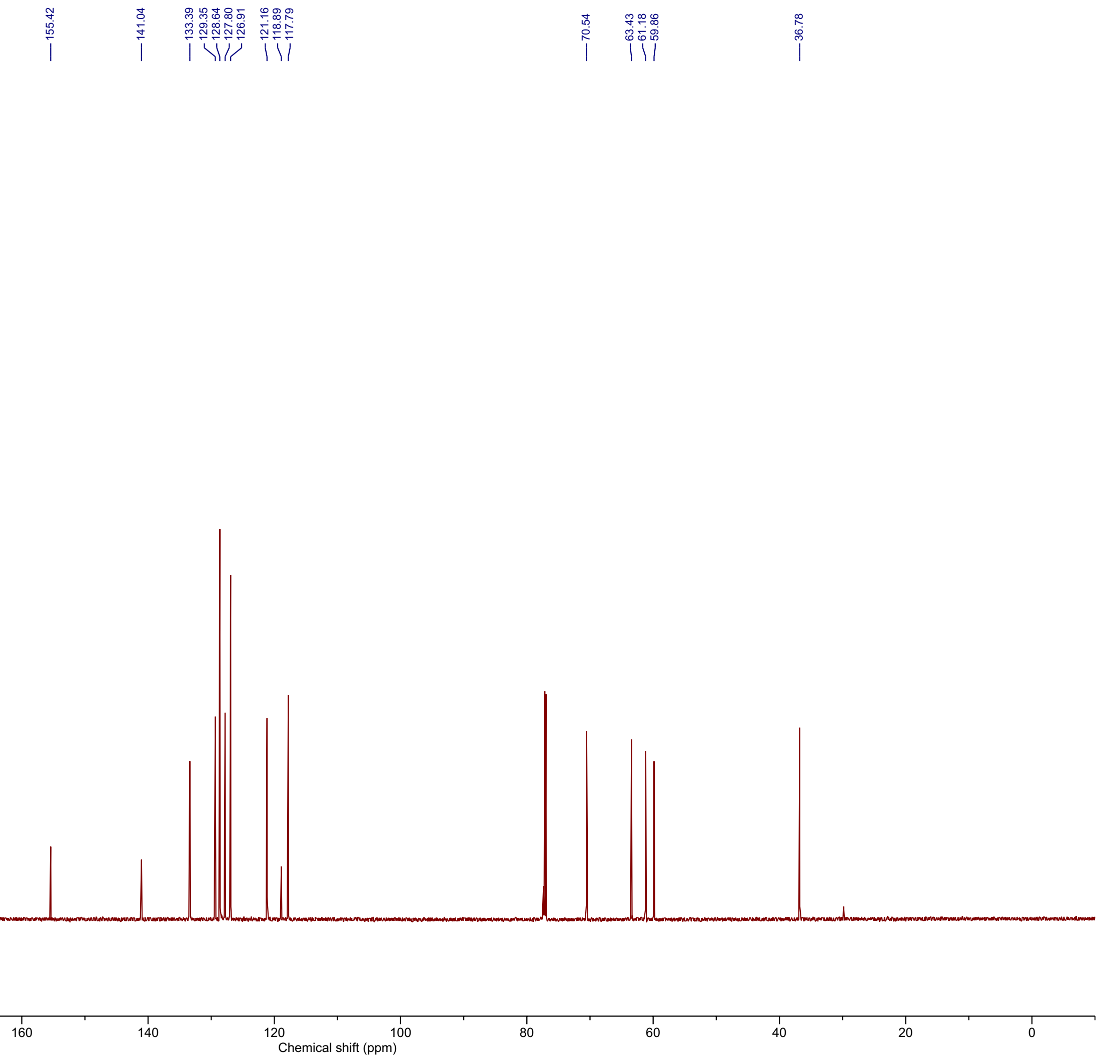

Supplementary Figure 145. ^{13}C NMR (176 MHz, $CDCl_3$) of **35**.

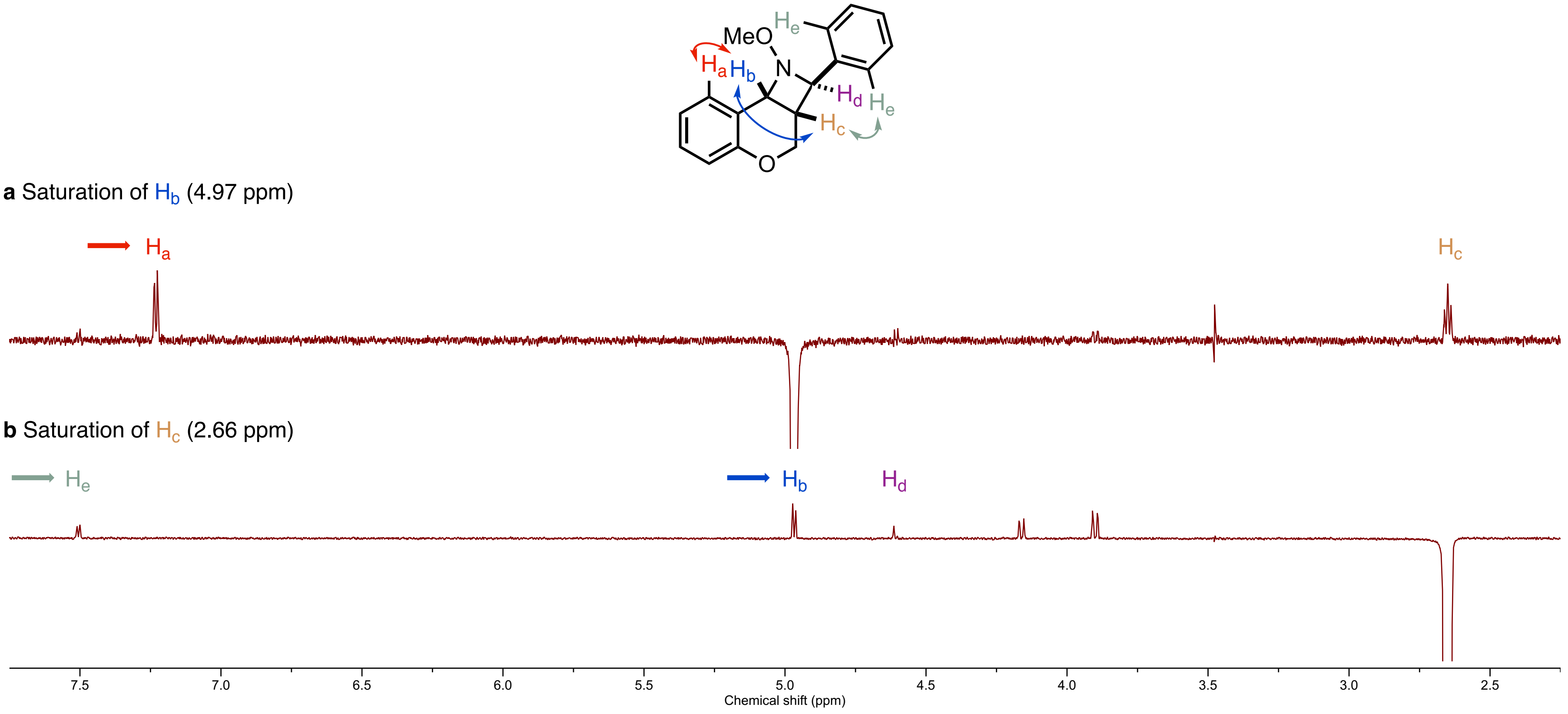

^{1}H NMR NOE (700 MHz, $CDCl_3$)

Supplementary Figure 146. ^{1}H NMR NOE of **35**. **a** Saturation of H_b at 4.97 ppm; **b** Saturation of H_c at 2.66 ppm.

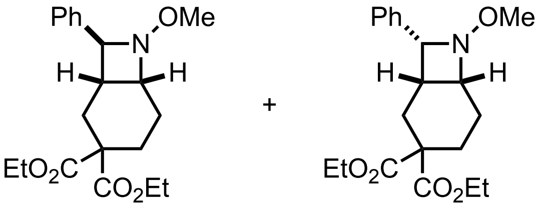

5 : 1

36 (major) **36** (minor)

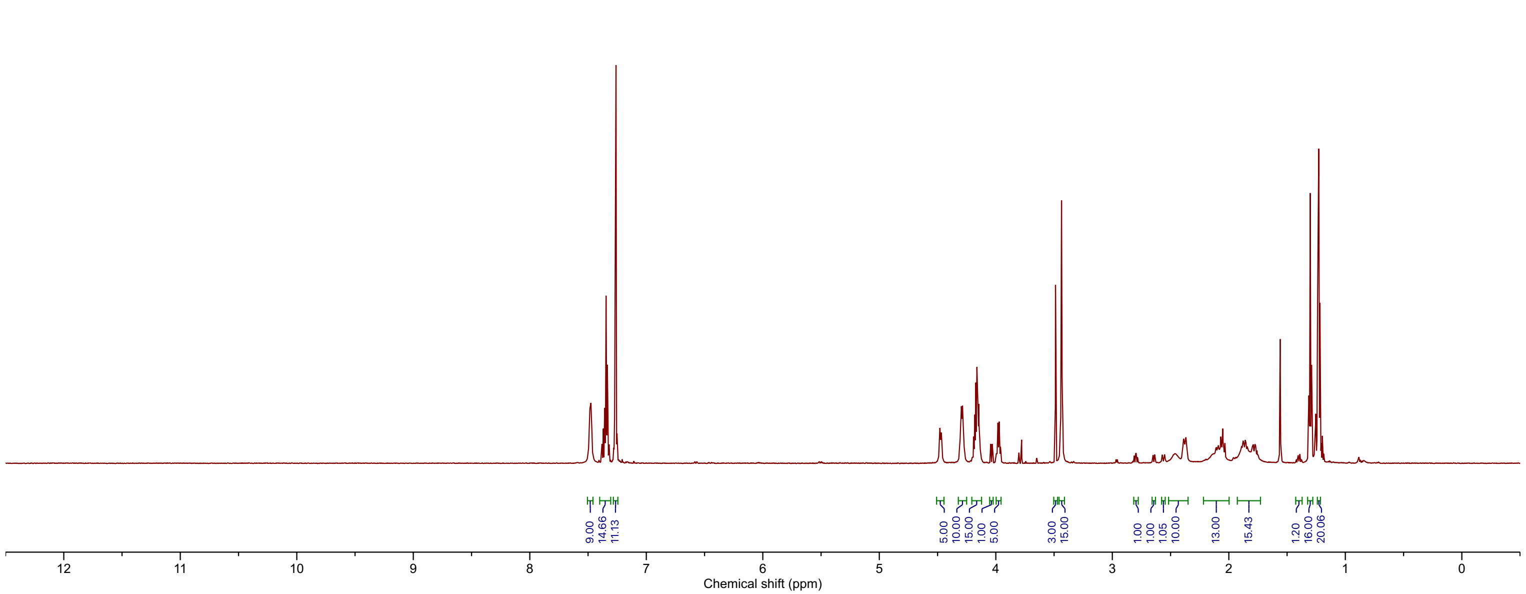

Supplementary Figure 147. ^{1}H NMR (700 MHz, $CDCl_3$) of **36**.

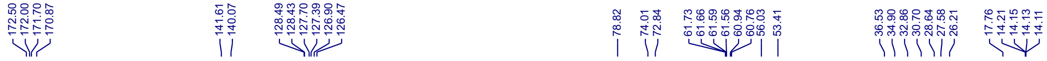

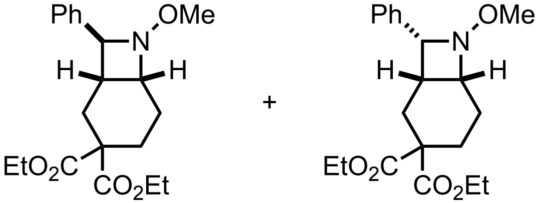

5 : 1

36 (major) **36** (minor)

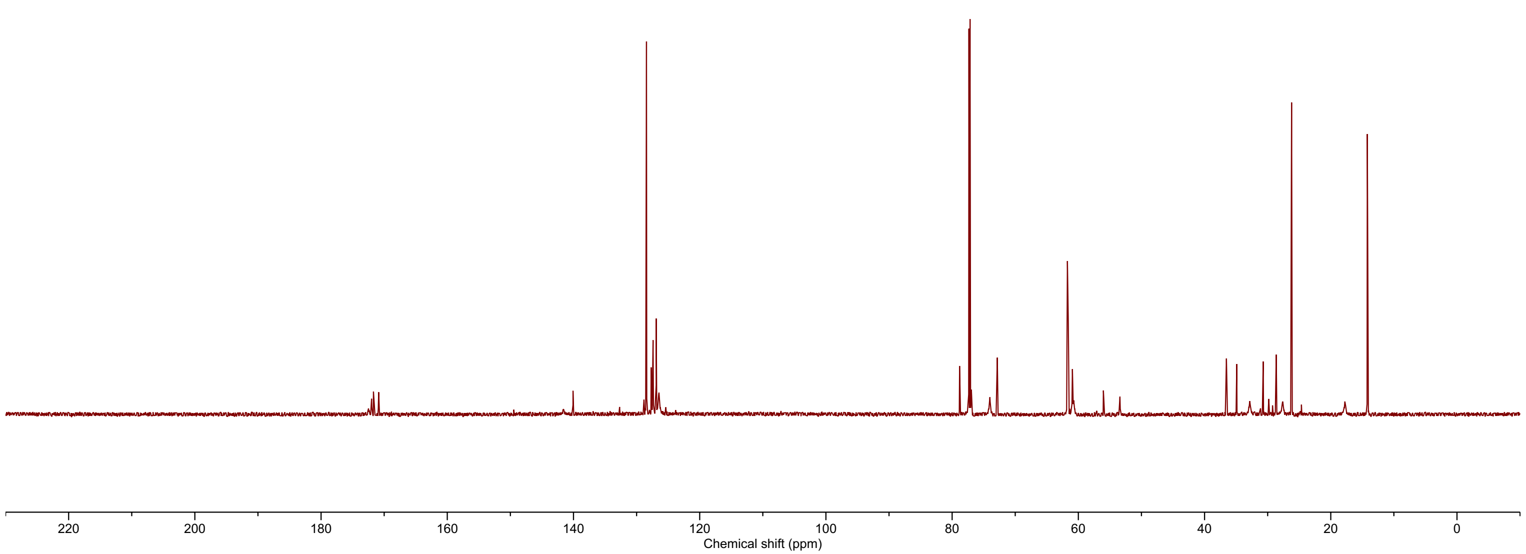

Supplementary Figure 148. ^{13}C NMR (176 MHz, $CDCl_3$) of **36**.

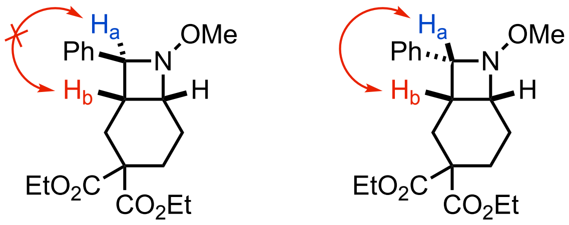

major diastereomer

minor diastereomer

a Saturation of H_a of major diastereomer (4.47 ppm)

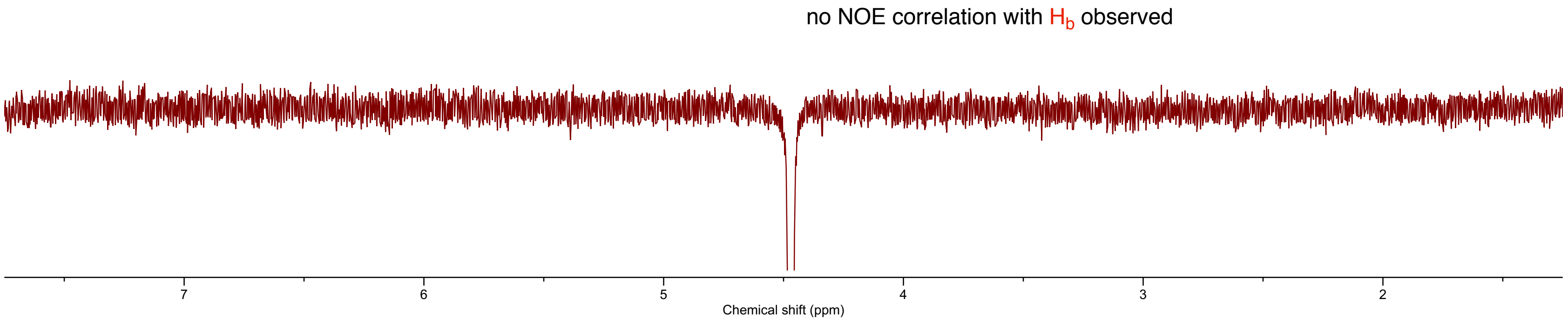

b Saturation of H_a of minor diastereomer (4.04 ppm)

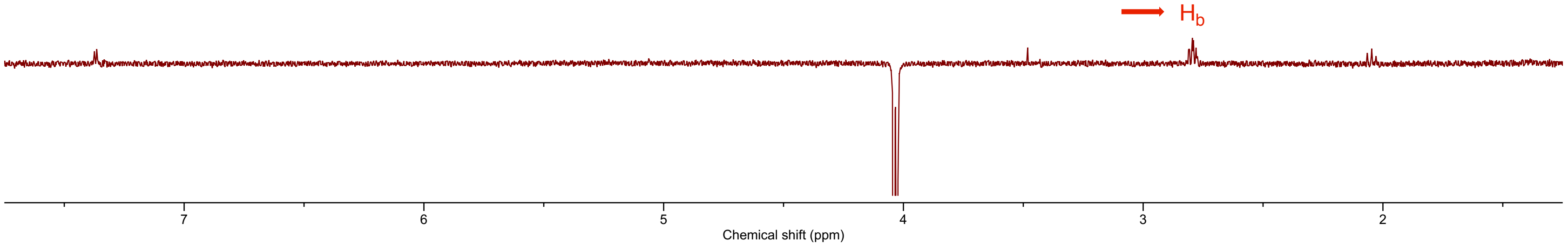

Supplementary Figure 149. ^{1}H NMR NOE of **36**. **a** Saturation of H_a (major diastereomer) at 4.47 ppm; **b** Saturation of H_a (minor diastereomer) at 4.04 ppm.

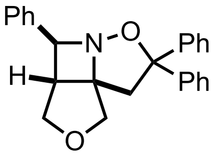

39

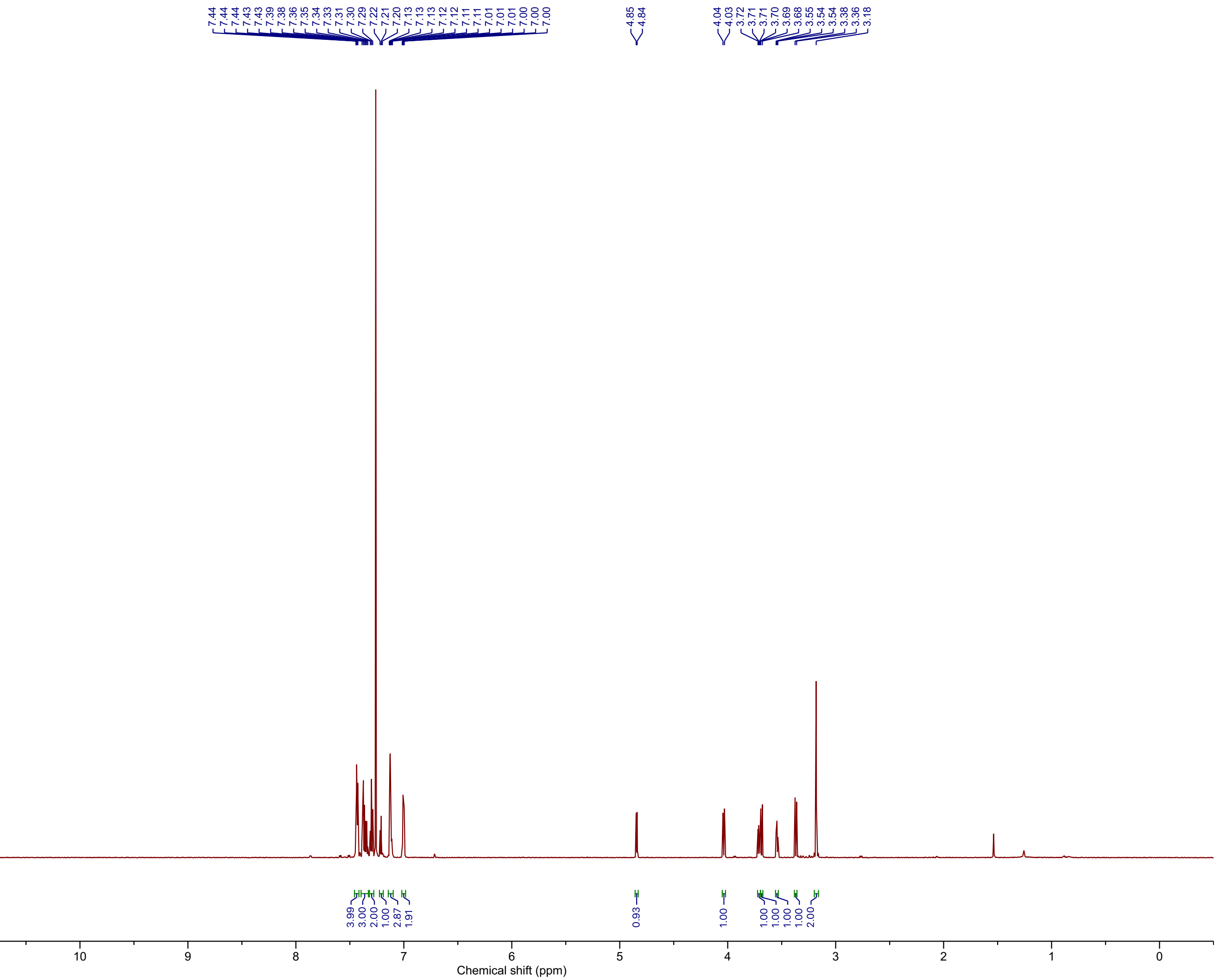

Supplementary Figure 150. ^{1}H NMR (700 MHz, $CDCl_3$) of **39**.

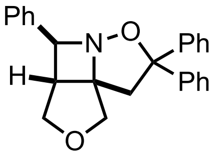

39

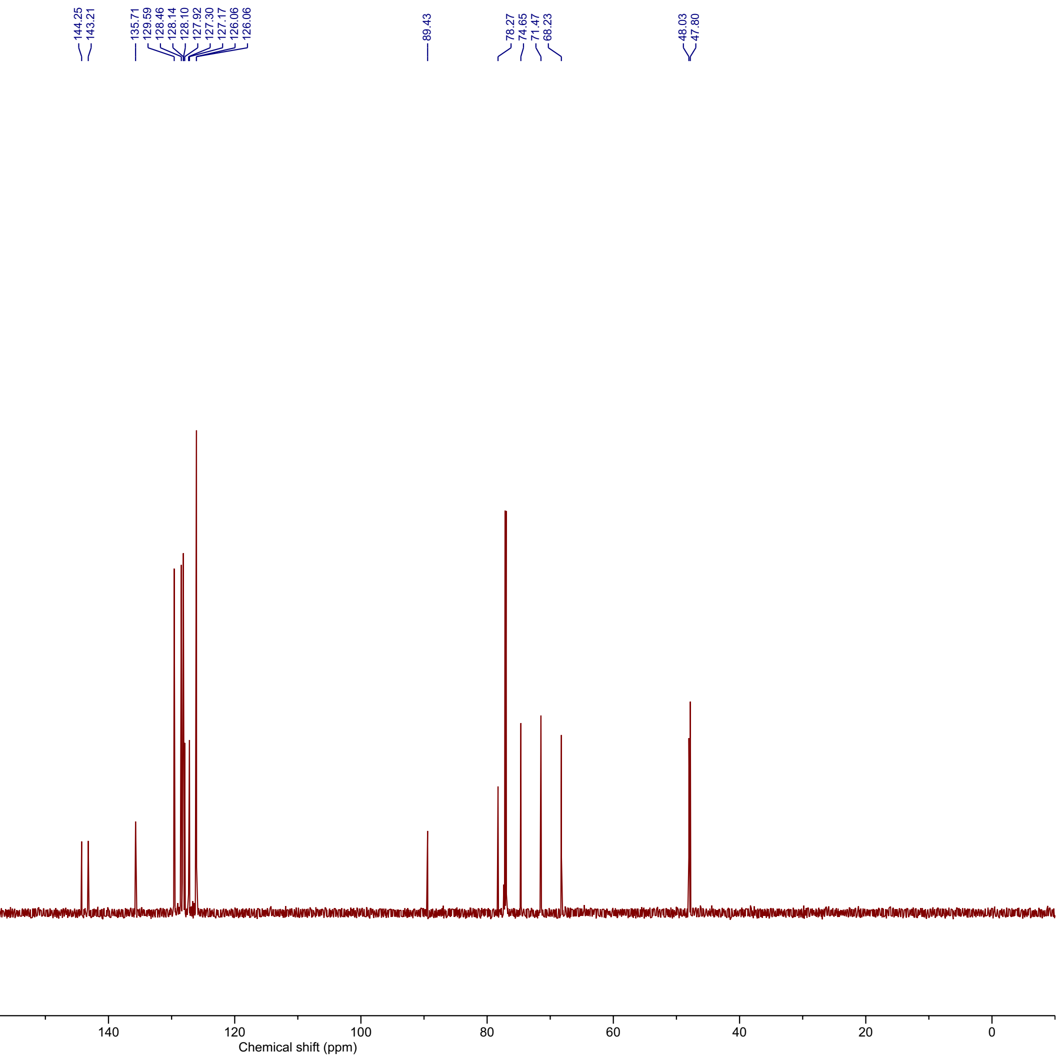

Supplementary Figure 151. ^{13}C NMR (176 MHz, $CDCl_3$) of **39**.

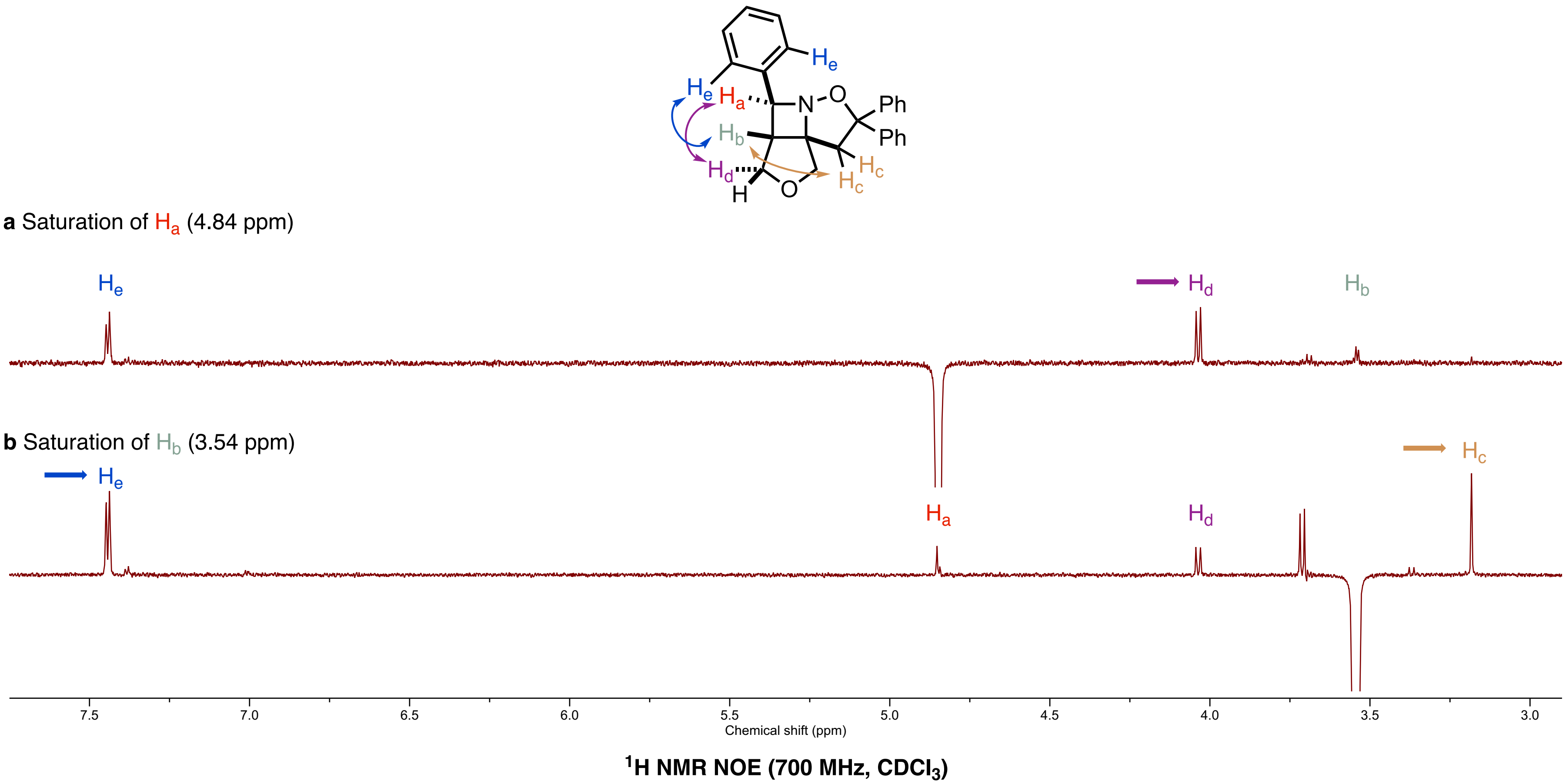

Supplementary Figure 152. ^{1}H NMR NOE of **39**. **a** Saturation of H_a at 4.84 ppm; **b** Saturation of H_b at 3.54 ppm.

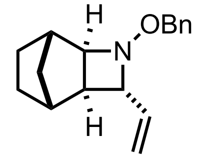

41

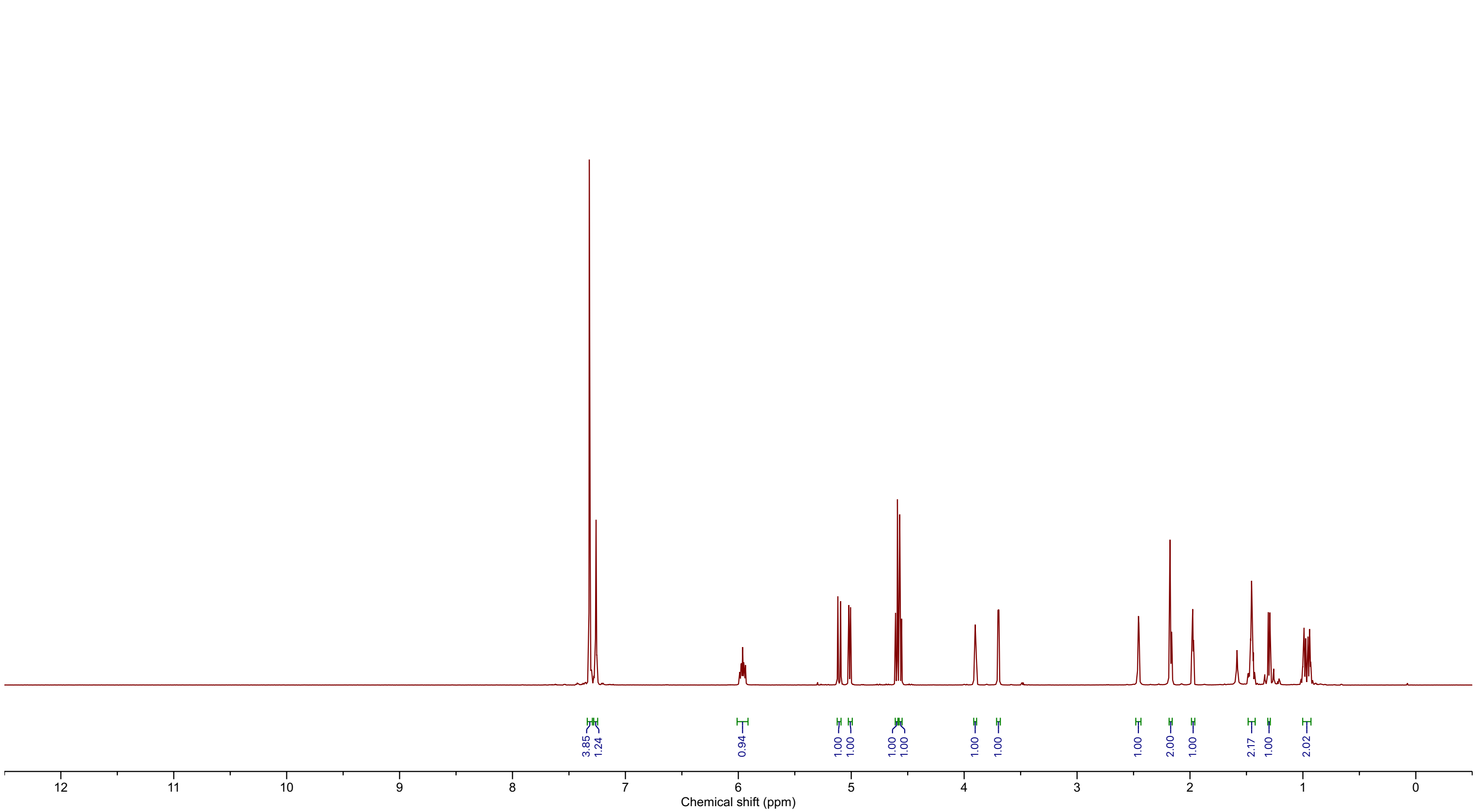

Supplementary Figure 153. ^{1}H NMR (700 MHz, $CDCl_3$) of **41**.

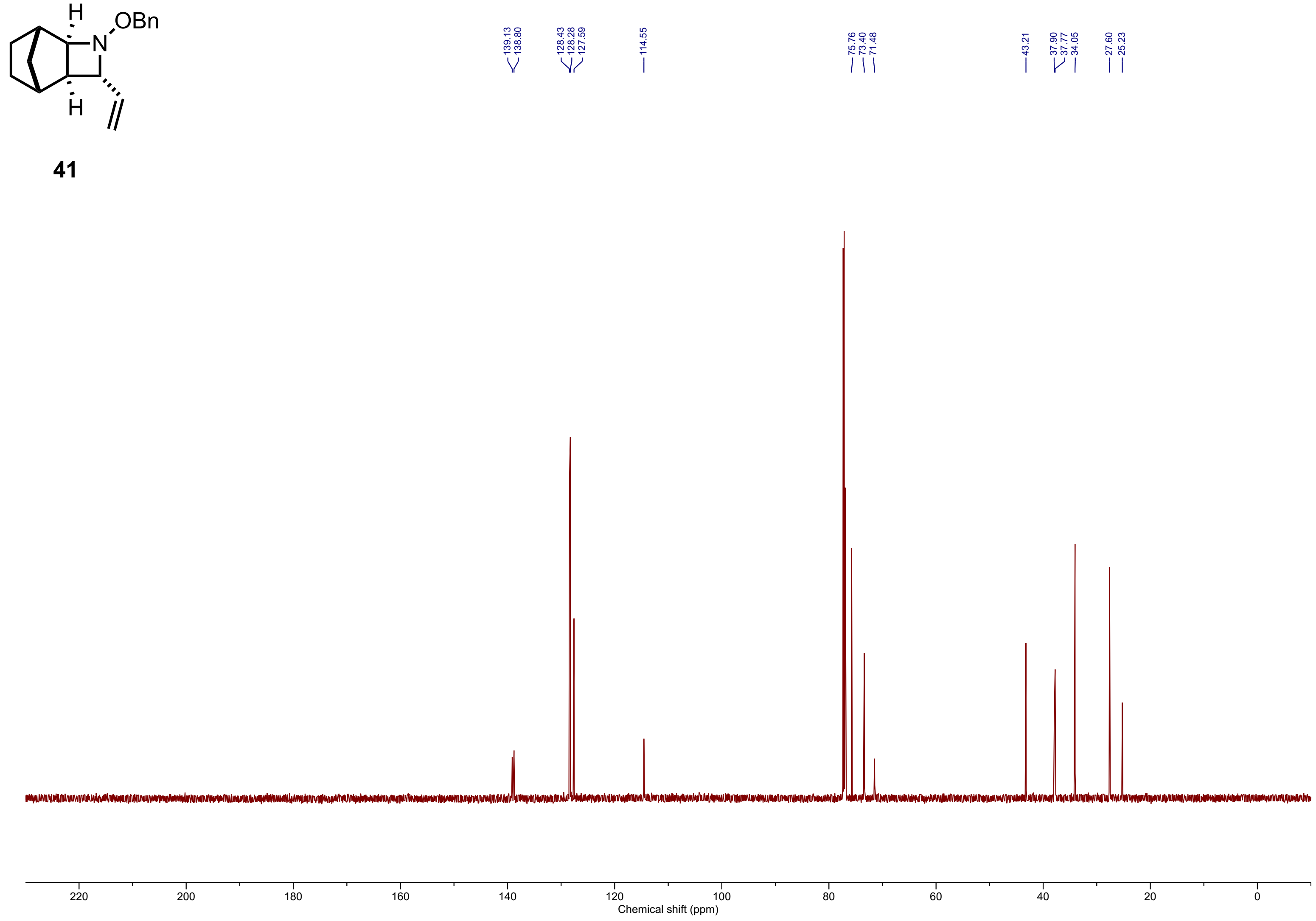

41

Supplementary Figure 154. ^{13}C NMR (176 MHz, $CDCl_3$) of **41**.

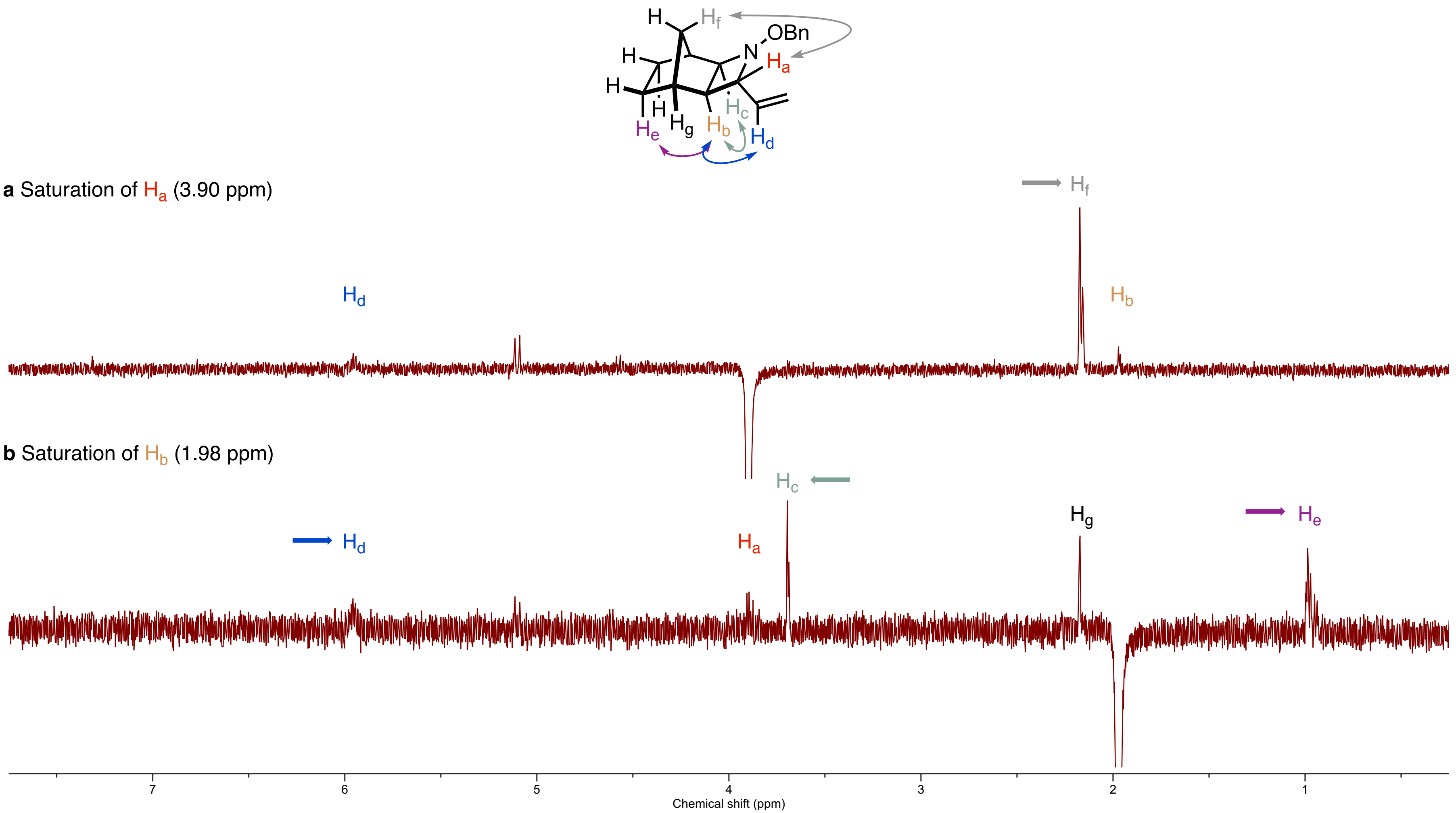

^{1}H NMR NOE (700 MHz, $CDCl_3$)

Supplementary Figure 155. ^{1}H NMR NOE of **41**. **a** Saturation of H_a at 3.90 ppm; **b** Saturation of H_b at 1.98 ppm.

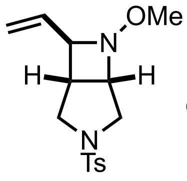

major
diastereomer

43

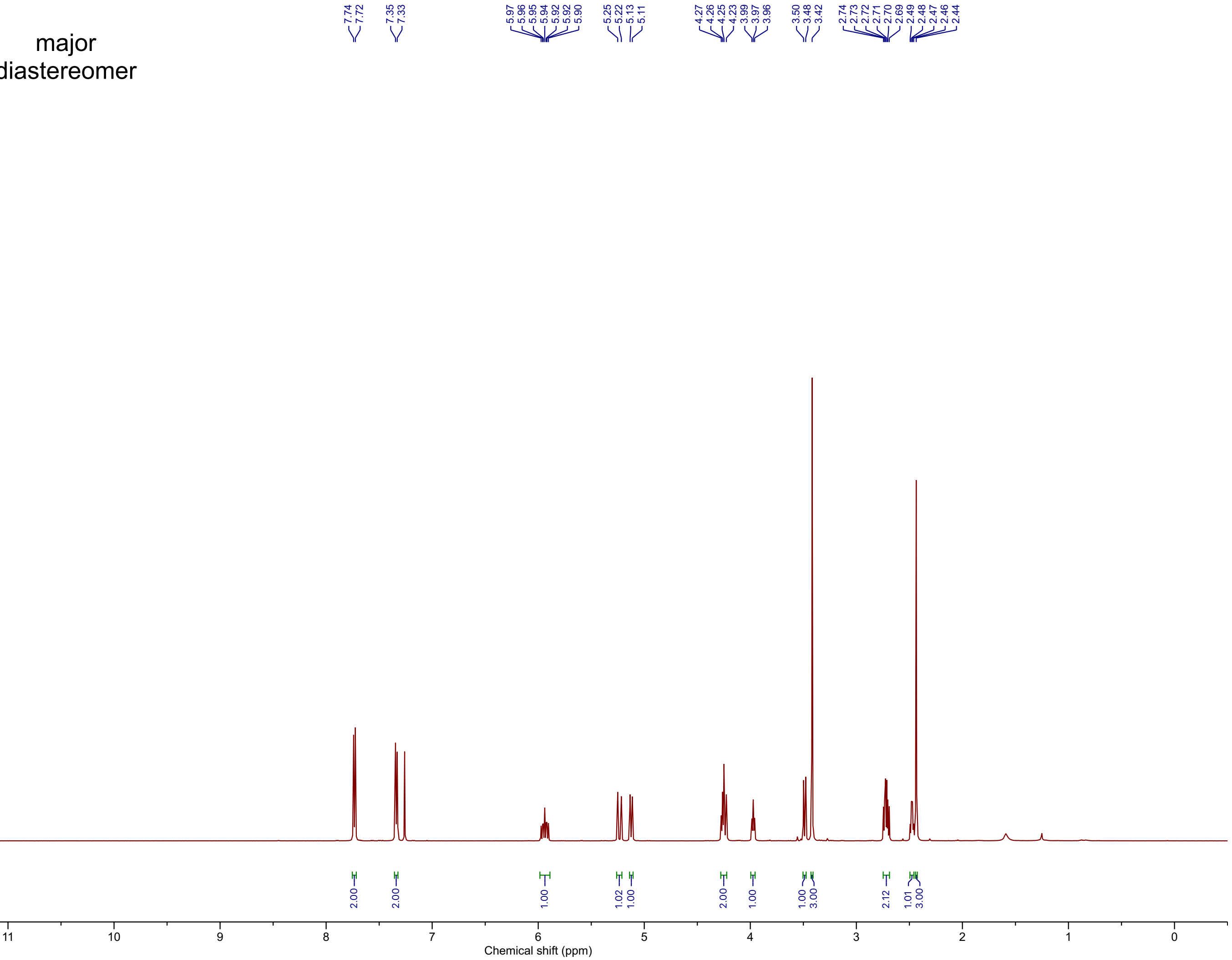

Supplementary Figure 156. ^{1}H NMR (500 MHz, $CDCl_3$) of **43** (major diastereomer).

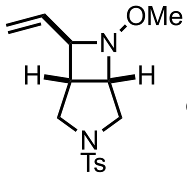

43

major diastereomer

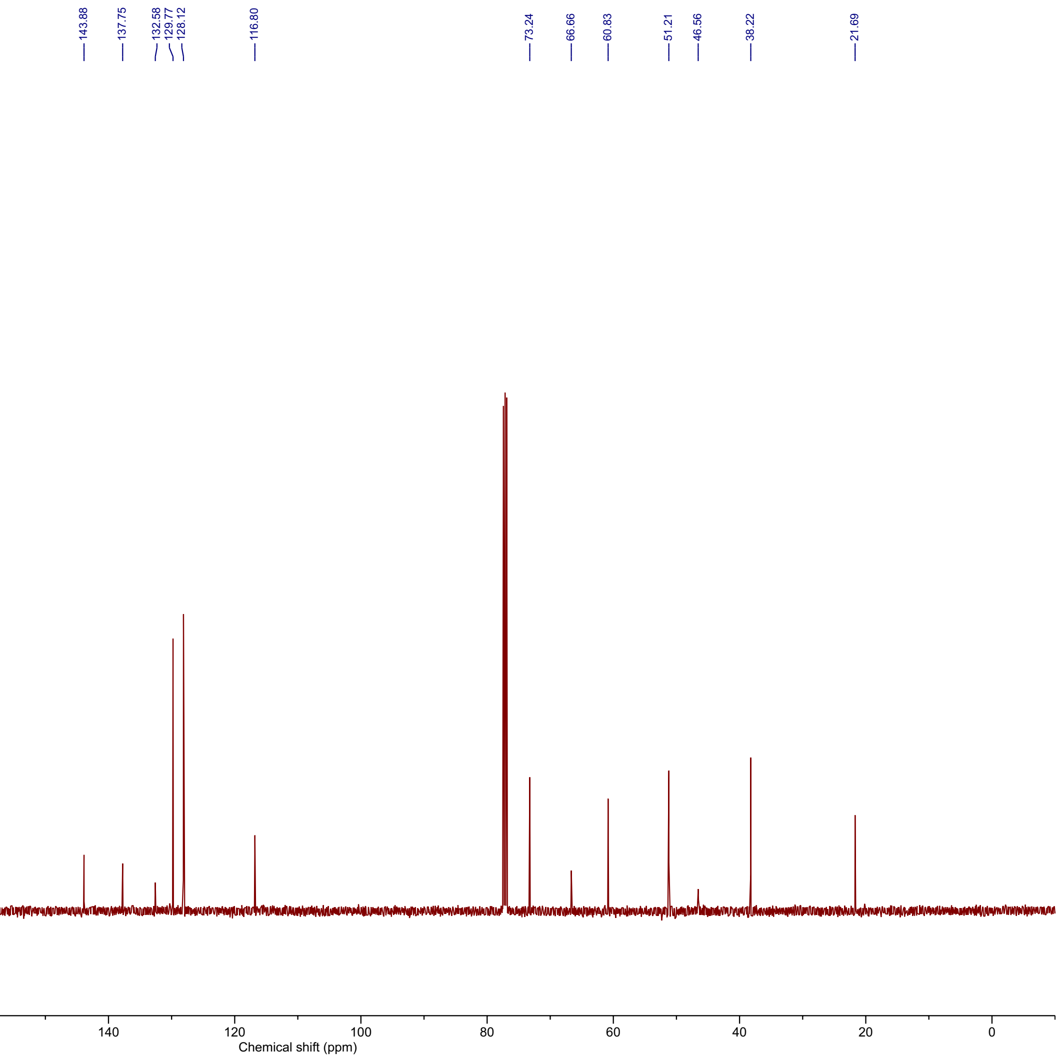

Supplementary Figure 157. ^{13}C NMR (126 MHz, $CDCl_3$) of **43** (major diastereomer).

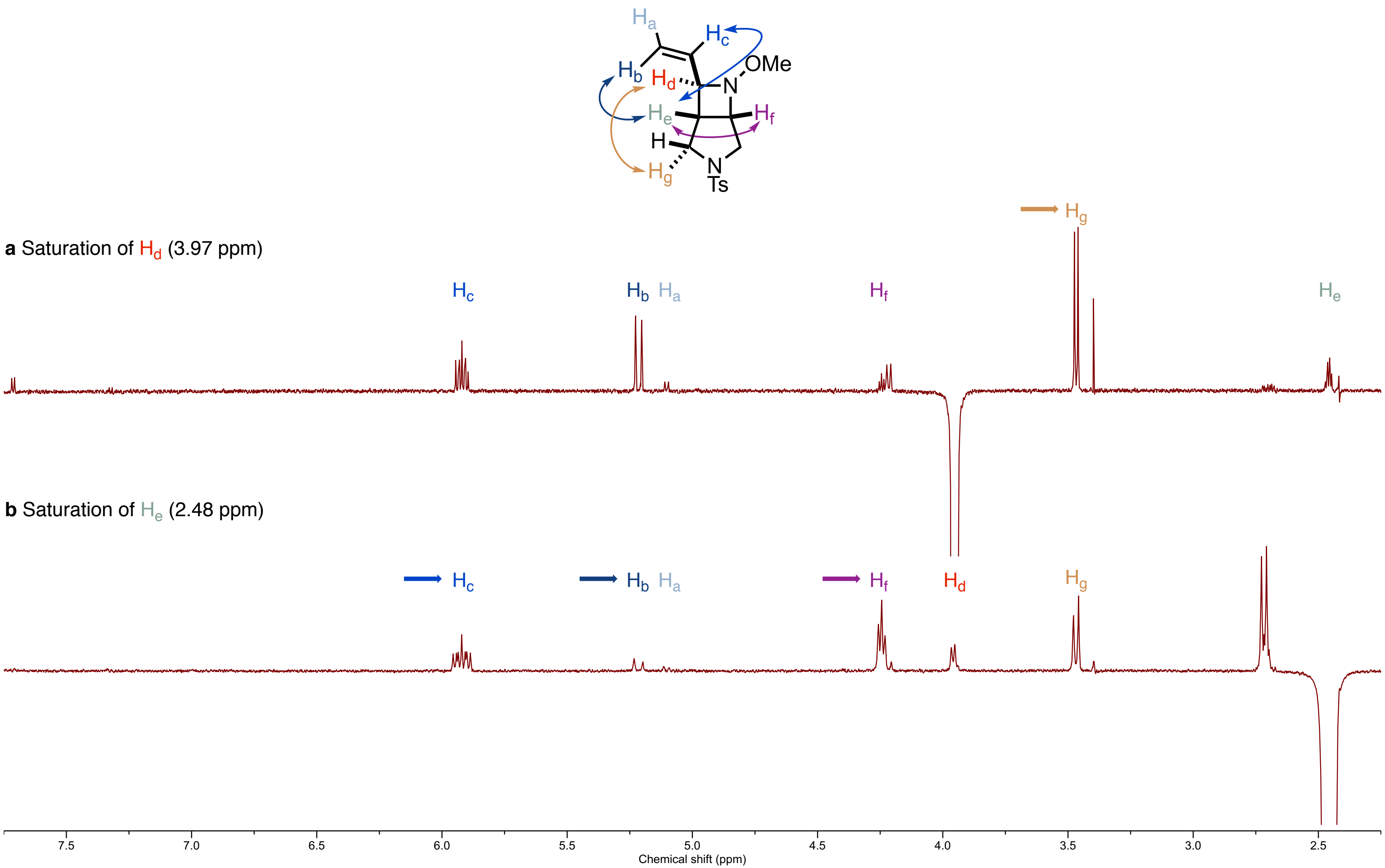

^{1}H NMR NOE (500 MHz, $CDCl_3$)

Supplementary Figure 158. ^{1}H NMR NOE of **43** (major diastereomer). **a** Saturation of H_d at 3.97 ppm **b** Saturation of H_e at 2.48 ppm.

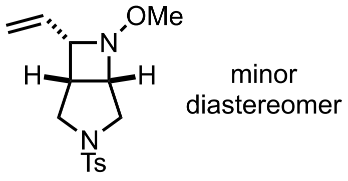

43

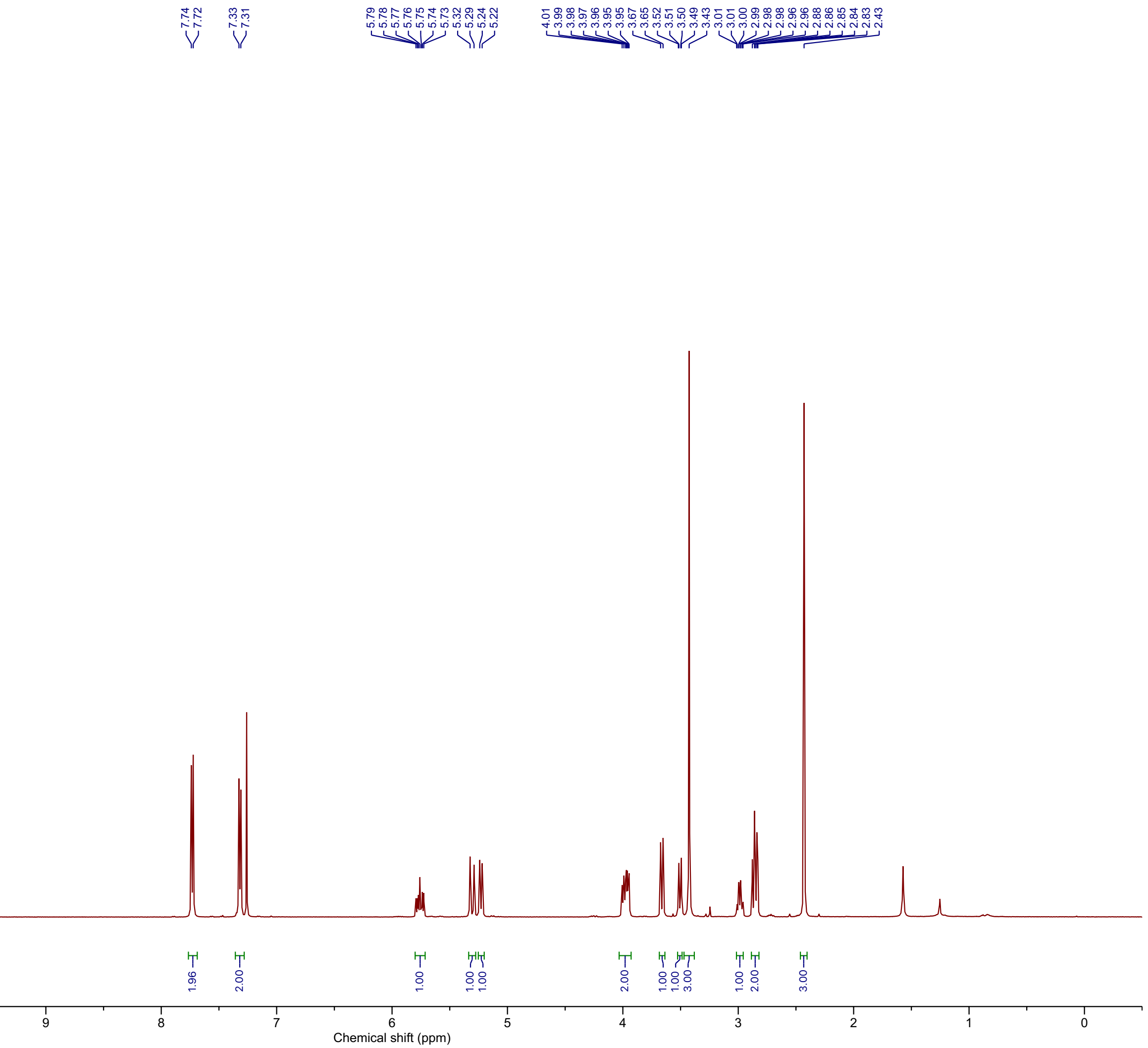

Supplementary Figure 159. ^{1}H NMR (500 MHz, $CDCl_3$) of **43** (minor diastereomer).

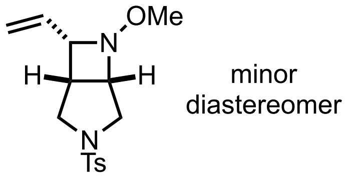

43

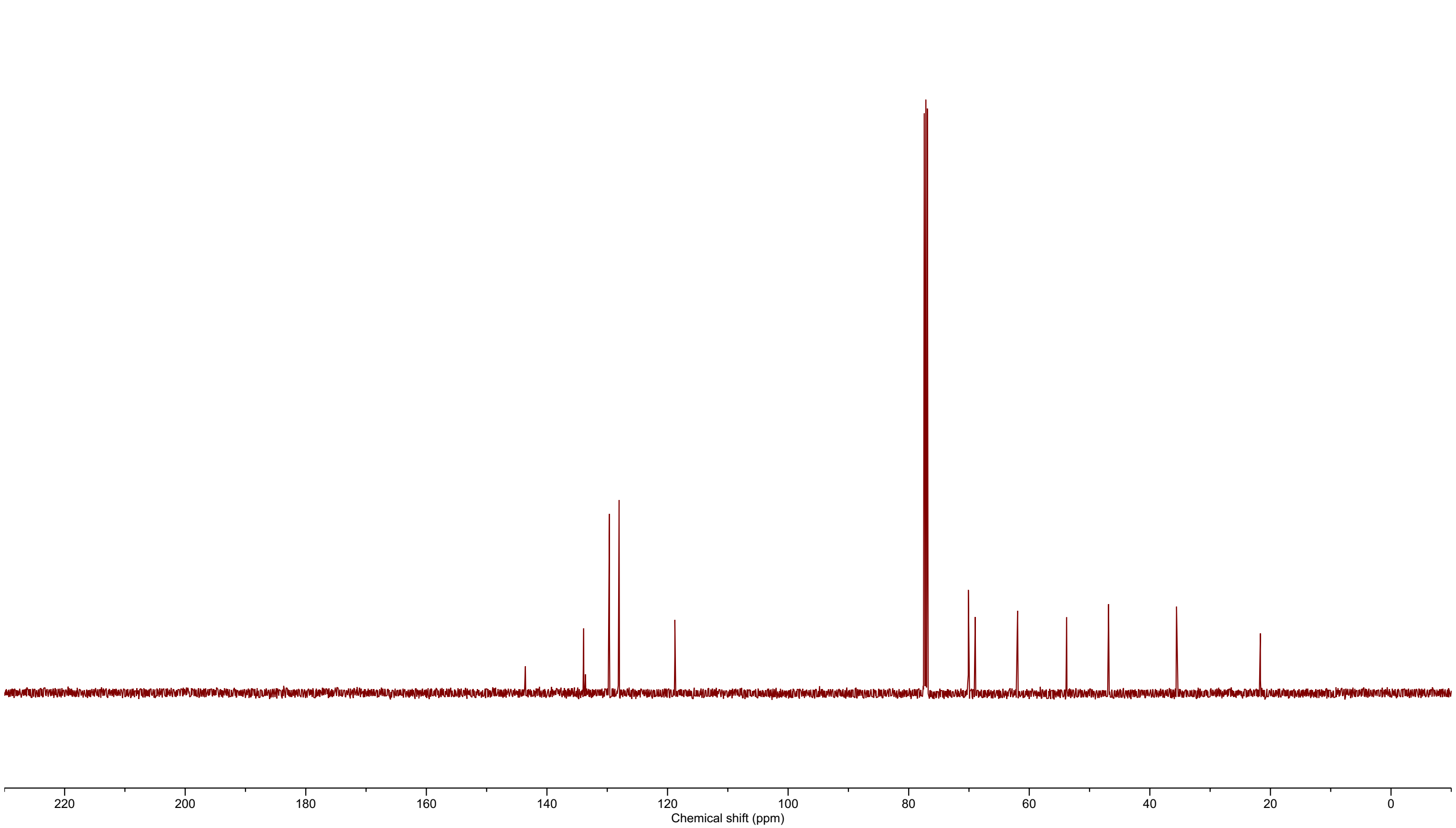

Supplementary Figure 160. ^{13}C NMR (126 MHz, $CDCl_3$) of **43** (minor diastereomer).

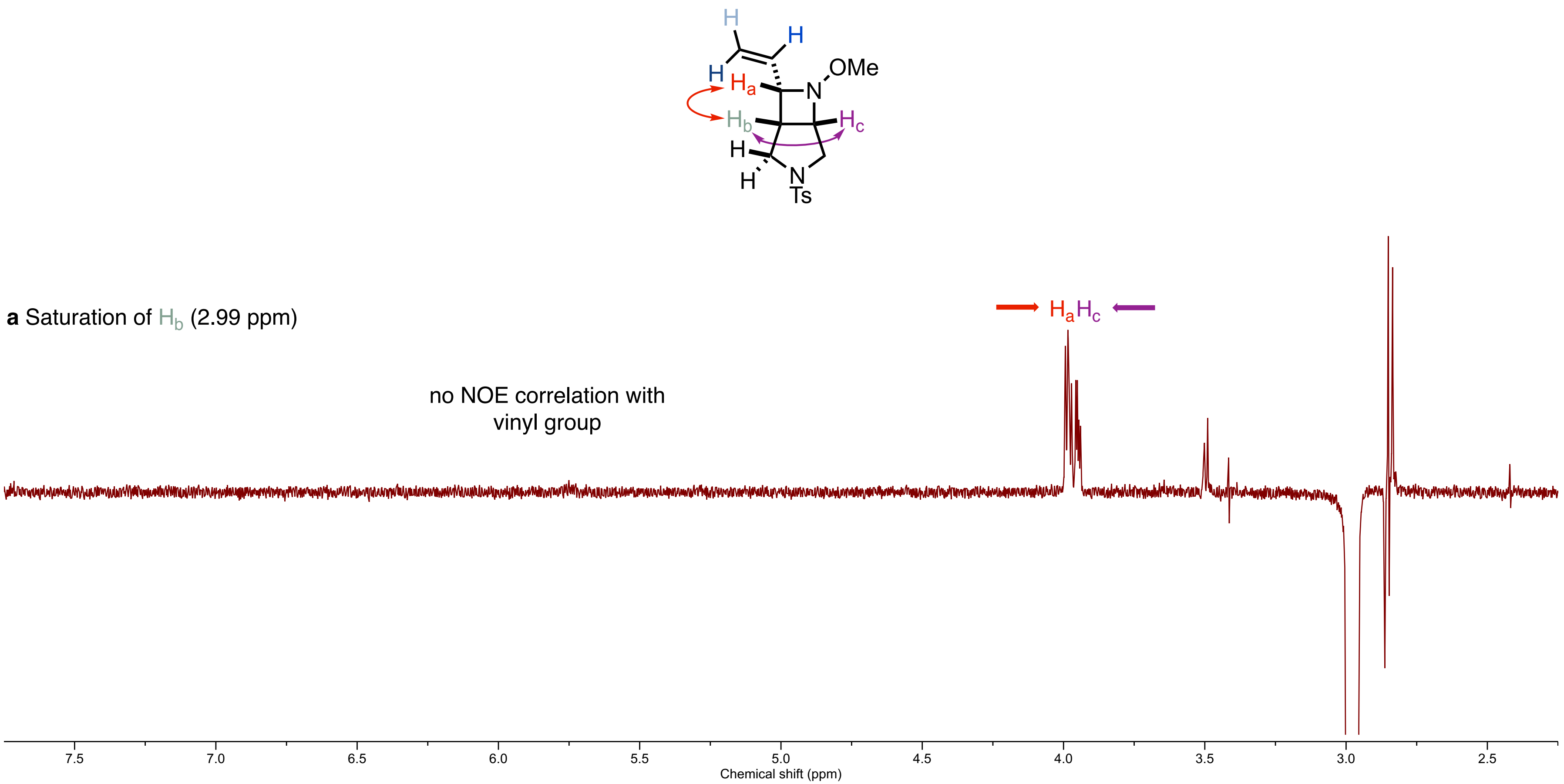

^{1}H NMR NOE (500 MHz, CDCl$_3$)

Supplementary Figure 161. ^{1}H NMR NOE of **43** (minor diastereomer). **a** Saturation of H_b at 2.99 ppm.

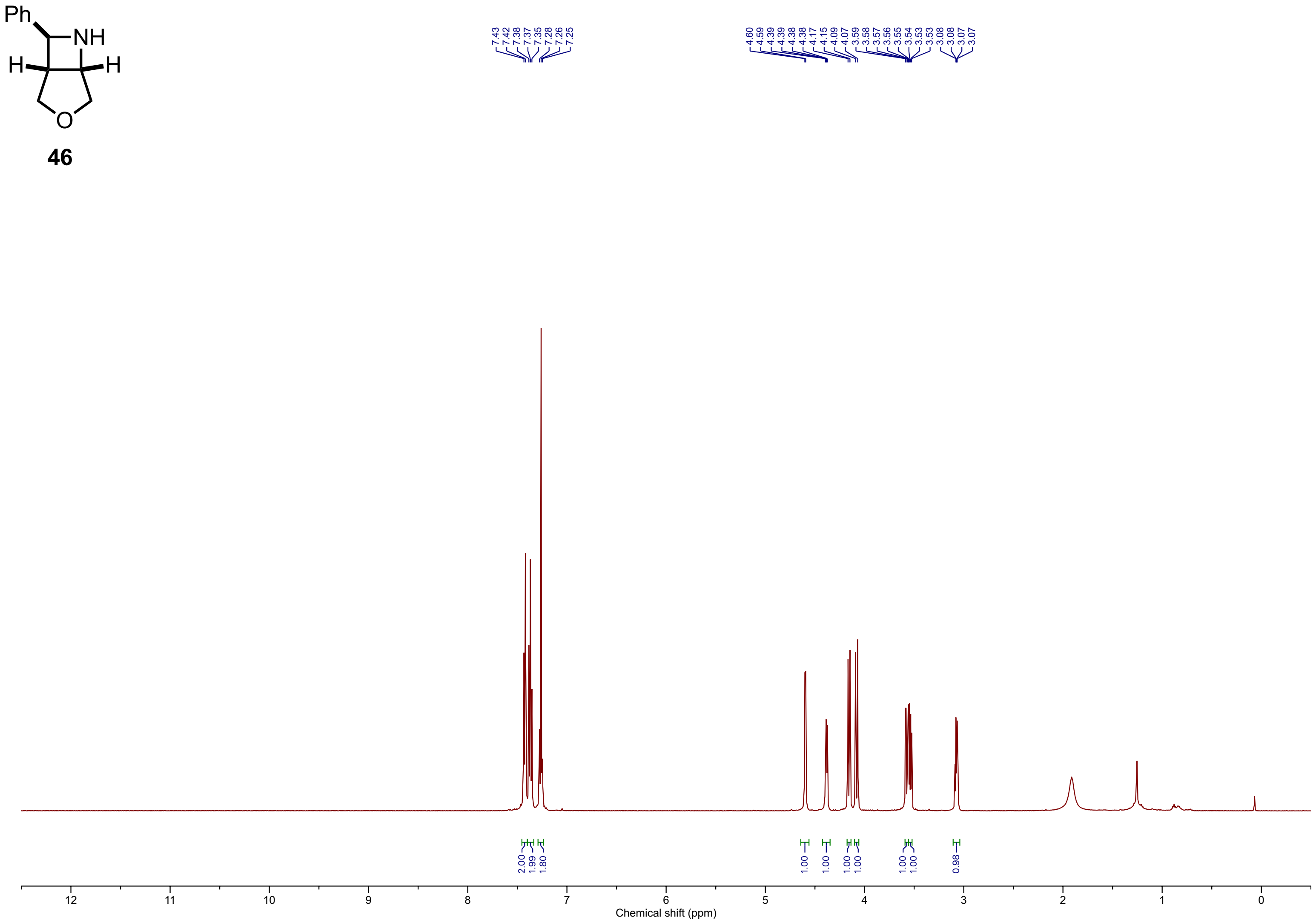

46

Supplementary Figure 162. ^{1}H NMR (500 MHz, $CDCl_3$) of **46**.

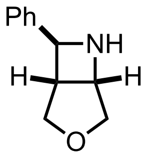

46

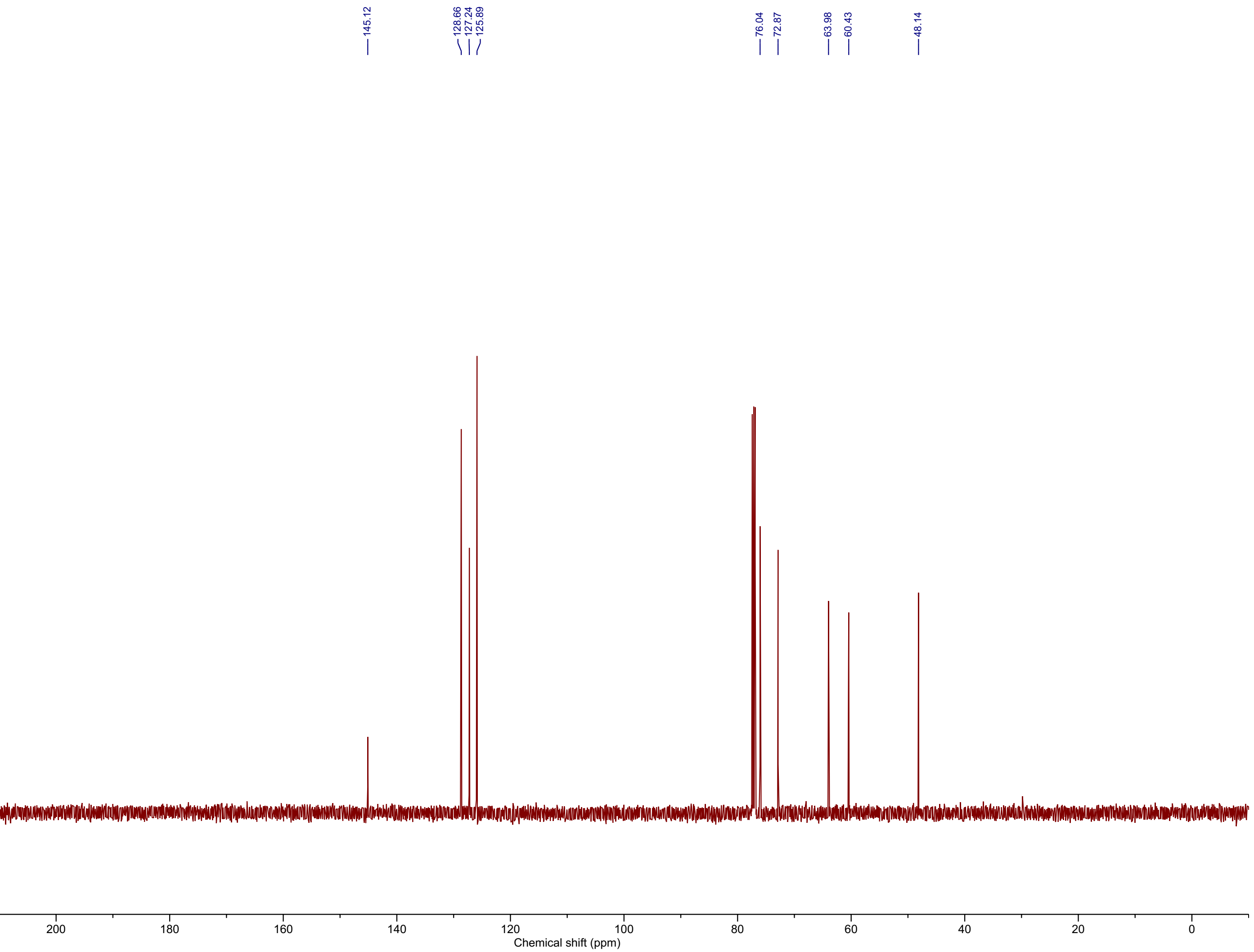

Supplementary Figure 163. ^{13}C NMR (126 MHz, $CDCl_3$) of **46**.

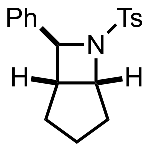

47

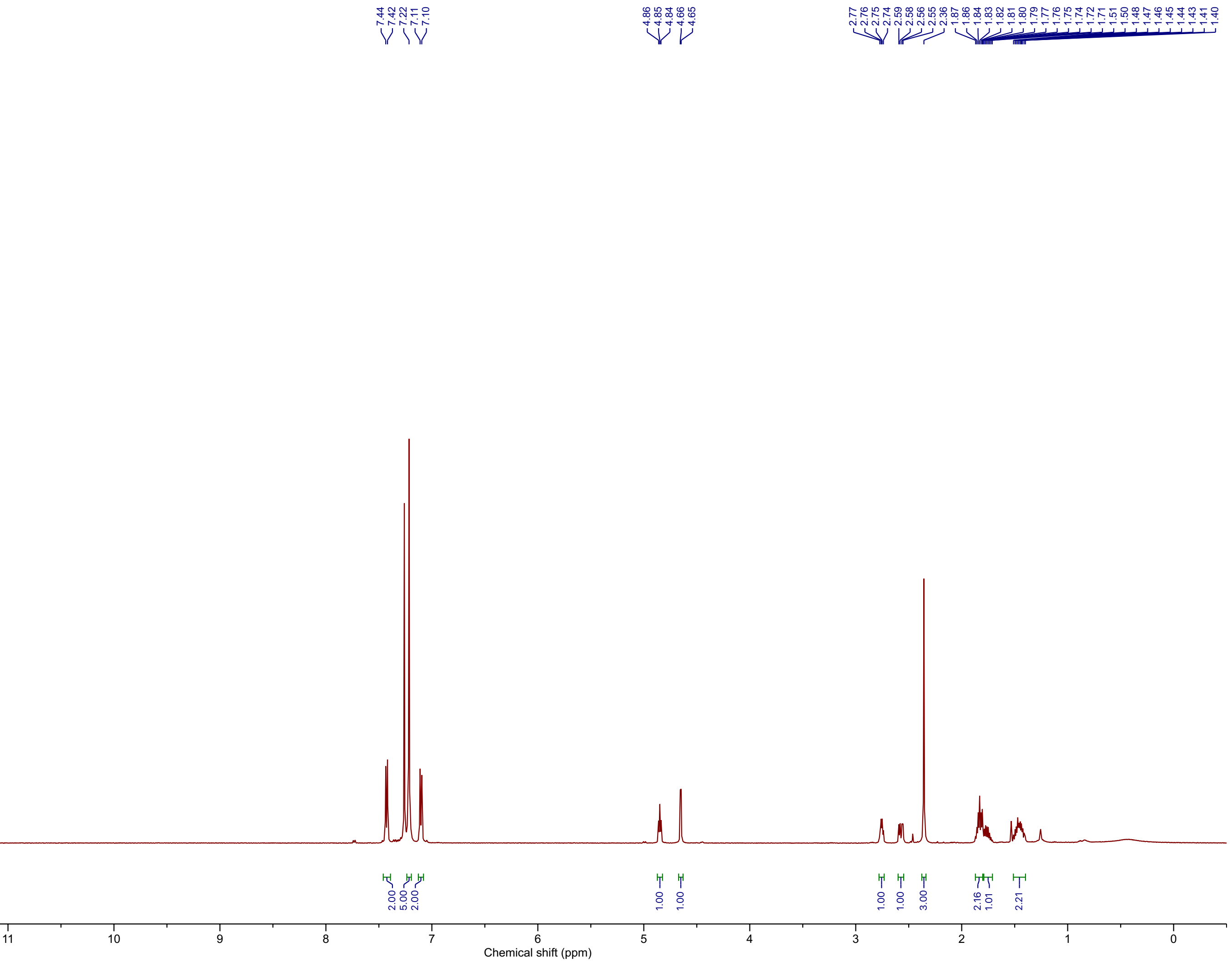

Supplementary Figure 164. ^{1}H NMR (700 MHz, $CDCl_3$) of **47**.

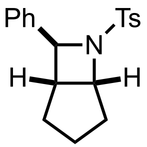

47

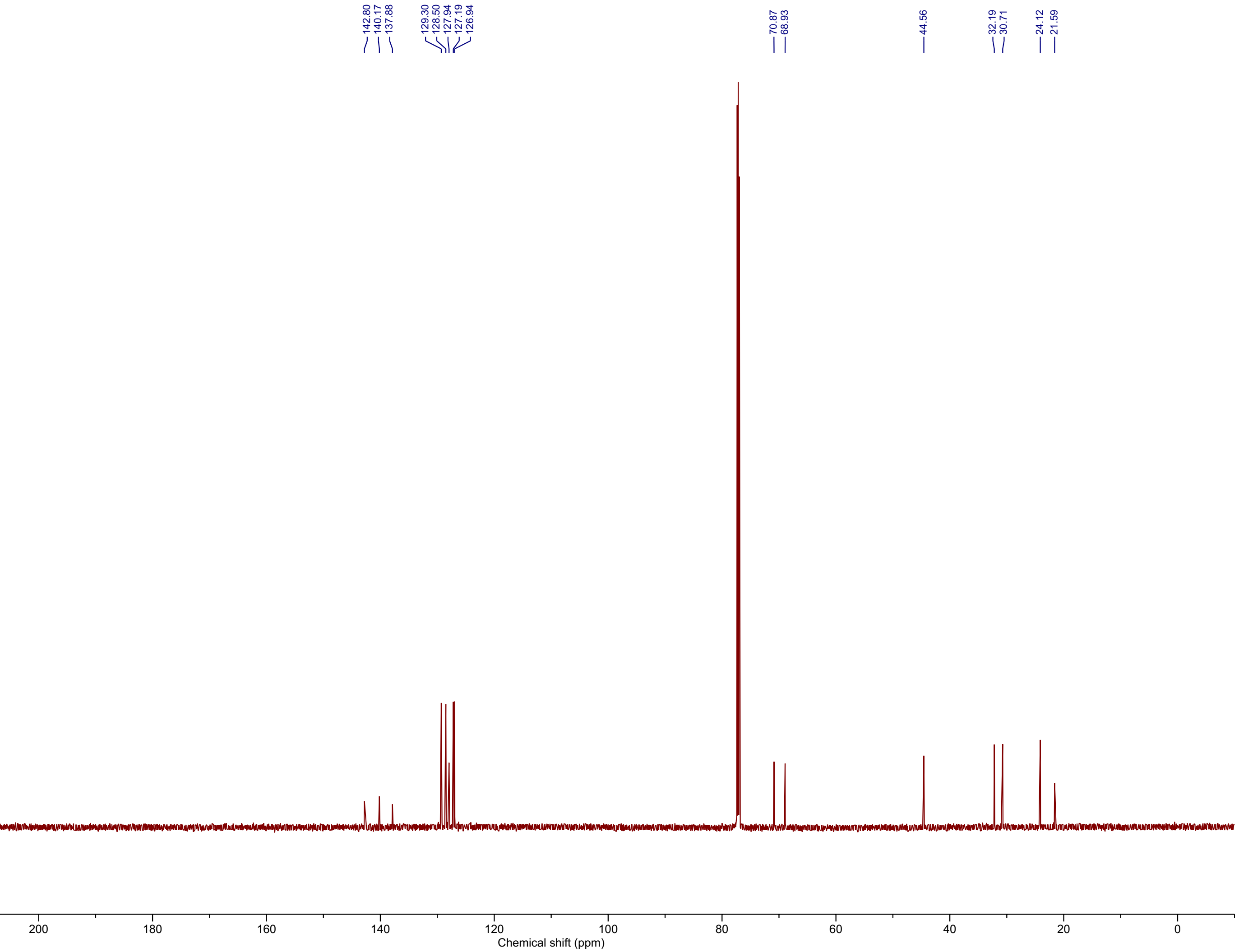

Supplementary Figure 165. ^{13}C NMR (176 MHz, $CDCl_3$) of **47**.

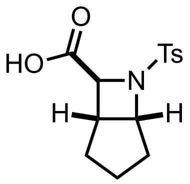

48

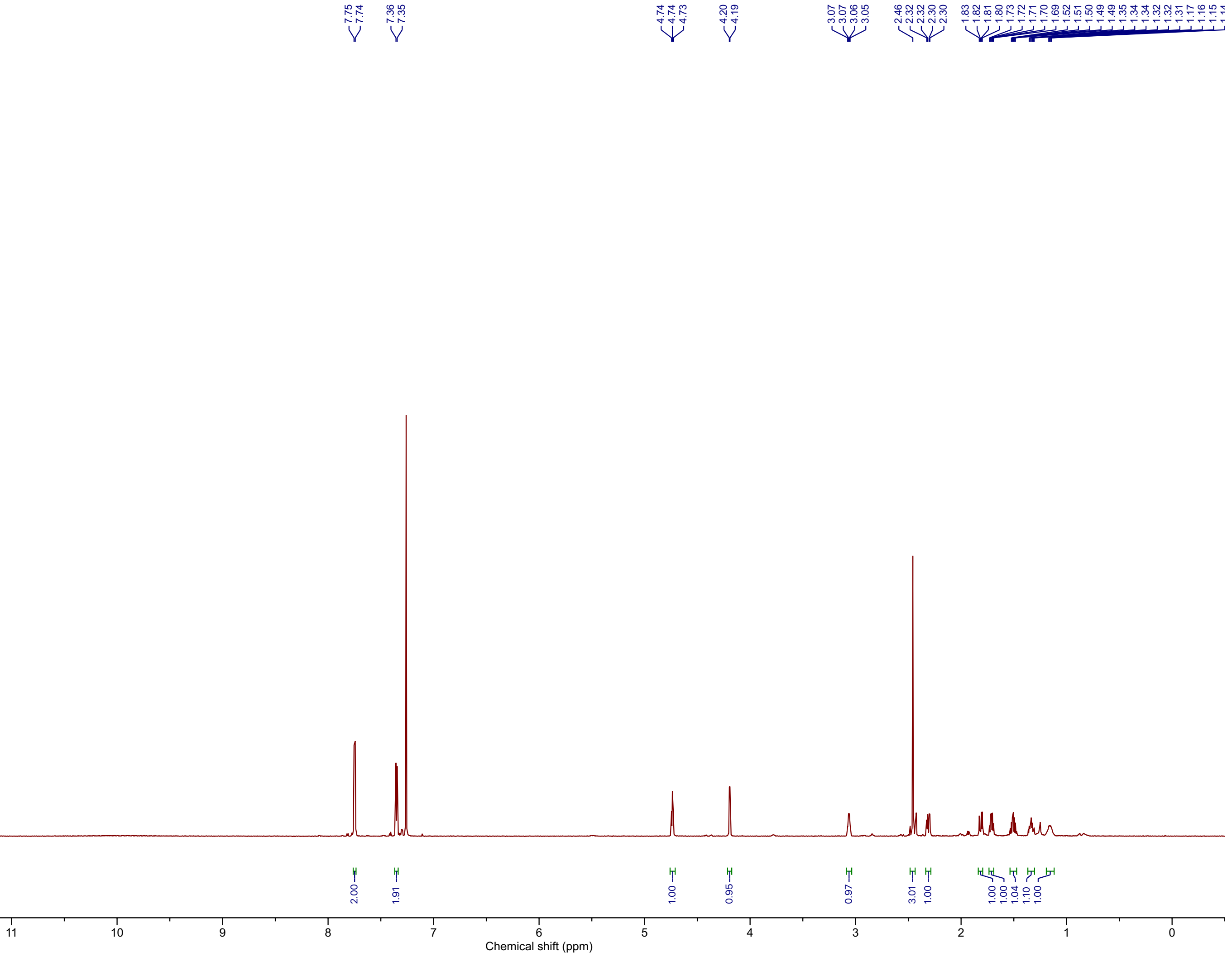

Supplementary Figure 166. ^{1}H NMR (700 MHz, $CDCl_3$) of **48**.

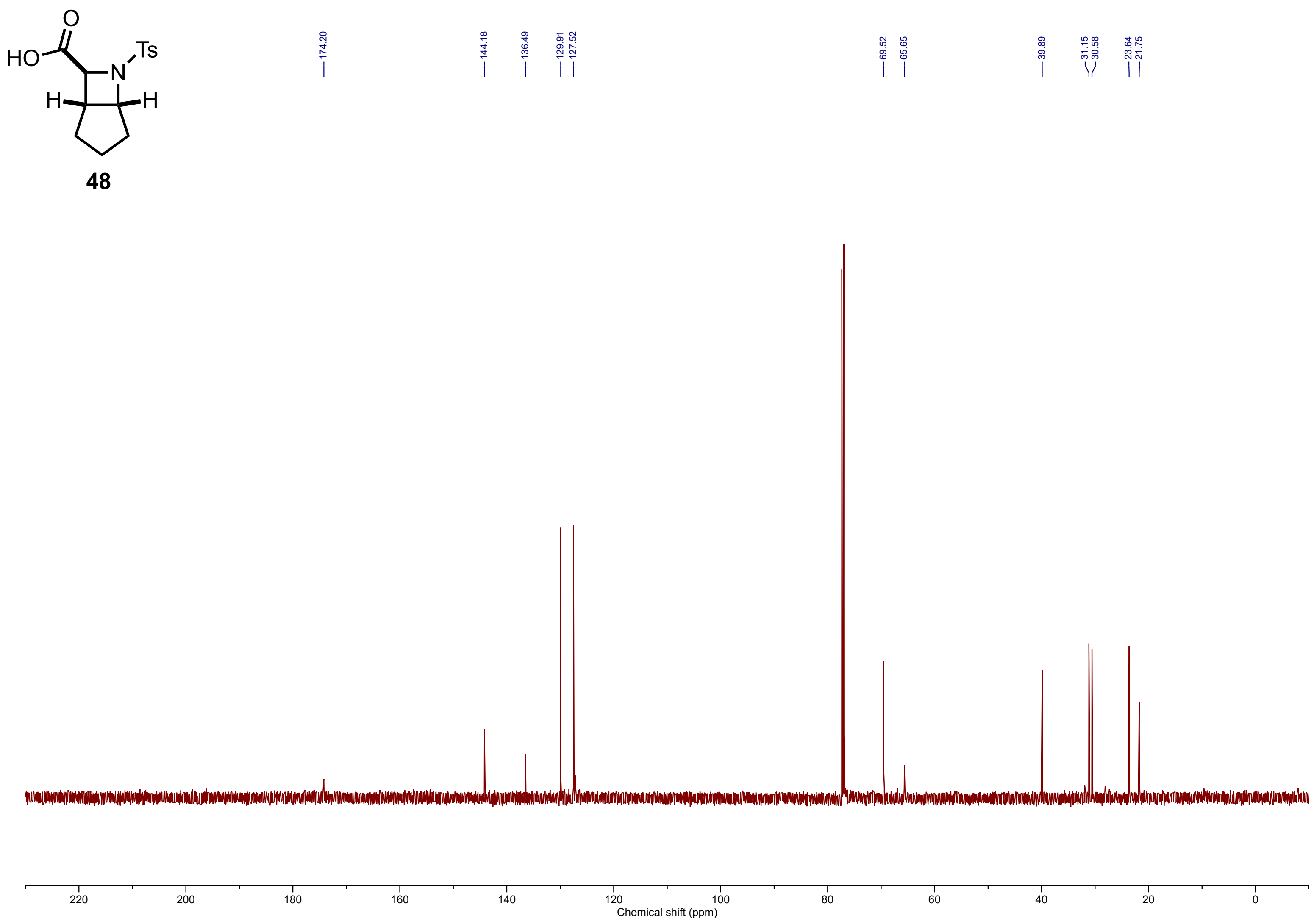

Supplementary Figure 167. ^{13}C NMR (176 MHz, $CDCl_3$) of **48**.

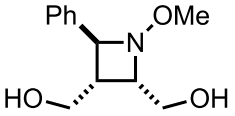

49

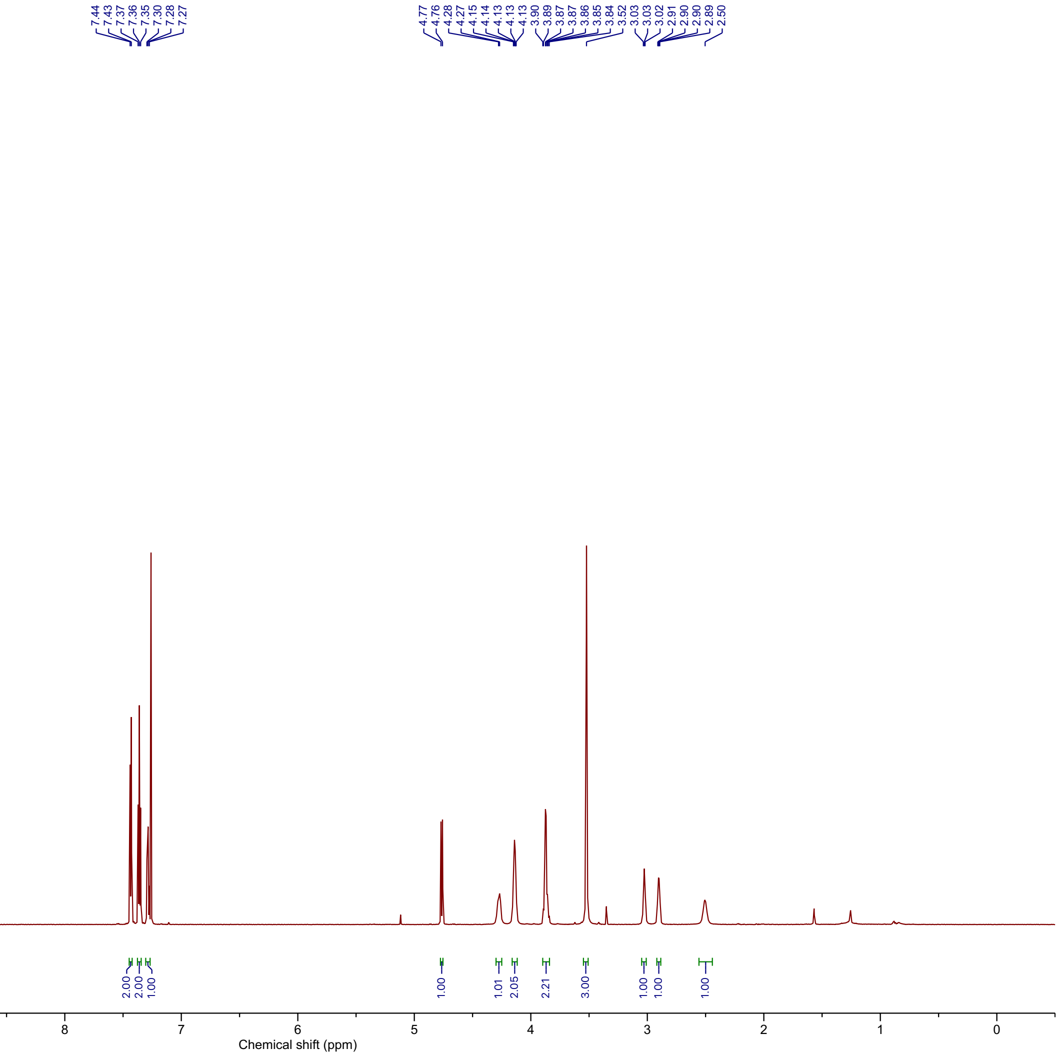

Supplementary Figure 168. ^{1}H NMR (700 MHz, $CDCl_3$) of **49**.

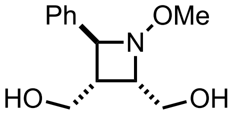

49

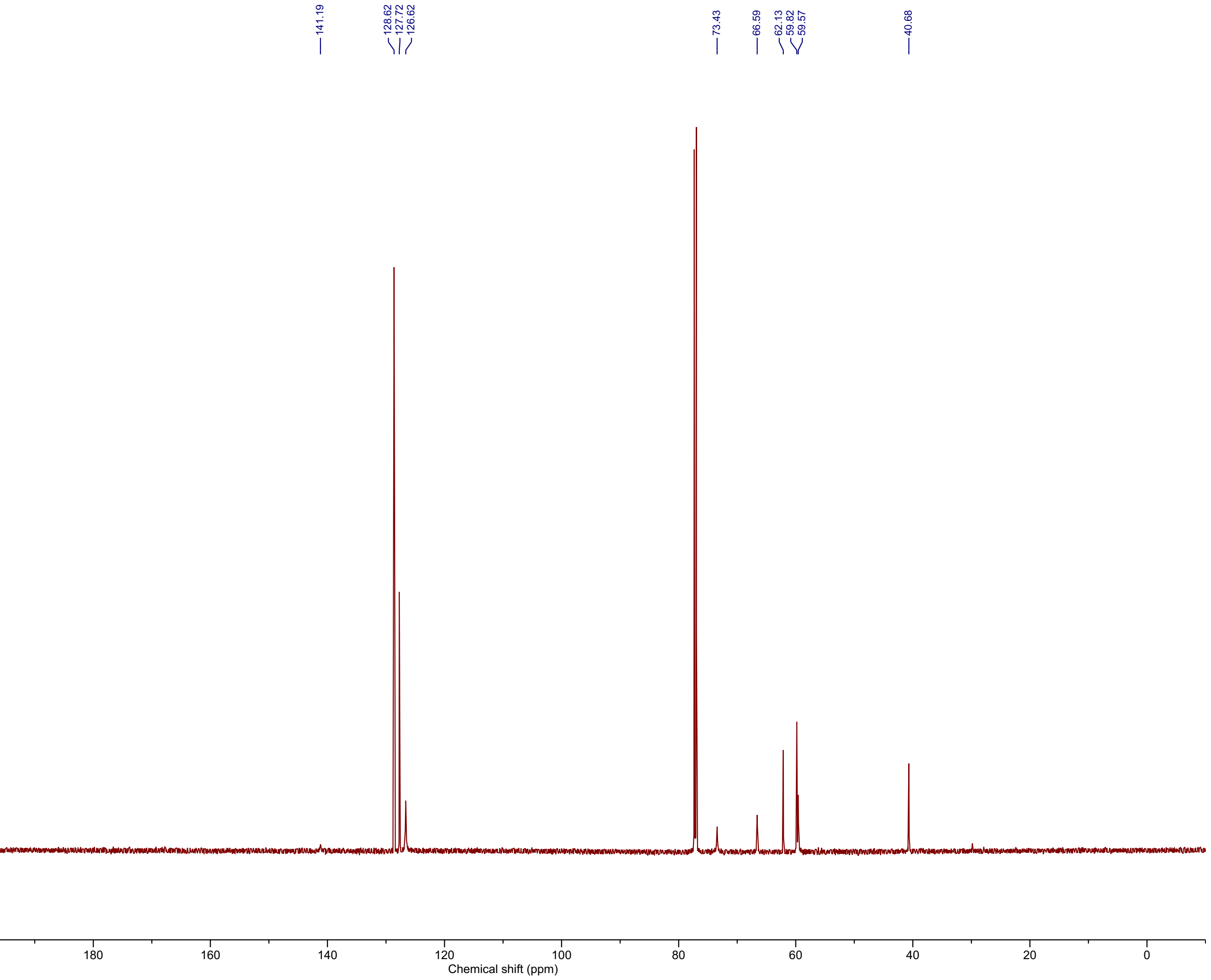

Supplementary Figure 169. ^{13}C NMR (176 MHz, $CDCl_3$) of **49**.

Supplementary References

1. Monos, T. M., Sun, A. C., McAtee, R. C., Devery, J. J. & Stephenson, C. R. J. Microwave-Assisted Synthesis of Heteroleptic Ir(III) + Polypyridyl Complexes. *J. Org. Chem.* **81**, 6988–6994 (2016).
2. Frigerio, M., Santagostino, M. & Sputore, S. A user-friendly entry to 2-iodoxybenzoic acid (IBX). *J. Org. Chem.* **64**, 4537–4538 (1999).
3. Roth, H. G., Romero, N. A. & Nicewicz, D. A. Experimental and Calculated Electrochemical Potentials of Common Organic Molecules for Applications to Single-Electron Redox Chemistry. *Synlett* **27**, 714–723 (2016).
4. Prier, C. K., Rankic, D. & MacMillan, D. W. C. Visible light photoredox catalysis with transition metal complexes: Applications in organic synthesis. *Chem. Rev.* **113**, 5322–63 (2013).
5. Strieth-Kalthoff, F., James, M. J., Teders, M., Pitzer, L. & Glorius, F. Energy transfer catalysis mediated by visible light: principles, applications, directions. *Chem. Soc. Rev.* **47**, 7190–7202 (2018).
6. Ni, T., Caldwell, R. A. & Melton, L. A. The relaxed and spectroscopic energies of olefin triplets. *J. Am. Chem. Soc.* **111**, 457–464 (1989).
7. Huo, X. *et al.* Stereoselective and Site-Specific Allylic Alkylation of Amino Acids and Small Peptides via a Pd/Cu Dual Catalysis. *J. Am. Chem. Soc.* **139**, 9819–9822 (2017).
8. Guo, R., Yang, H. & Tang, P. Silver-catalyzed Meerwein arylation: Intermolecular and intramolecular fluoroarylation of styrenes. *Chem. Commun.* **51**, 8829–8832 (2015).
9. Yu, Z., Liu, L. & Zhang, J. Triflic Acid-Catalyzed Enynes Cyclization: A New Strategy beyond Electrophilic π-Activation. *Chem. - A Eur. J.* **22**, 8488–8492 (2016).
10. Tamura, O. *et al.* Intramolecular cycloaddition of O-tert-butyldimethylsilyloximes in the presence of BF3-OEt2. *J. Org. Chem.* **70**, 10720–10725 (2005).
11. West, T. H., Daniels, D. S. B., Slawin, A. M. Z. & Smith, A. D. An isothiourea-catalyzed asymmetric [2,3]-rearrangement of allylic ammonium ylides. *J. Am. Chem. Soc.* **136**, 4476–4479 (2014).
12. Kablaoui, N. M. & Buchwald, S. L. Development of a method for the reductive cyclization of enones by a titanium catalyst. *J. Am. Chem. Soc.* **118**, 3182–3191 (1996).
13. Chen, Z. & Sun, J. Enantio- and diastereoselective assembly of tetrahydrofuran and tetrahydropyran skeletons with all-carbon-substituted quaternary stereocenters. *Angew. Chemie - Int. Ed.* **52**, 13593–13596 (2013).
14. Venning, A. R. O. *et al.* Palladium-Catalyzed Carbocyclizations of Unactivated Alkyl Bromides with Alkenes Involving Auto-tandem Catalysis. *J. Am. Chem. Soc.* **139**, 11595–11600 (2017).
15. Logan, A. W. J., Parker, J. S., Hallside, M. S. & Burton, J. W. Manganese(III) acetate mediated oxidative radical cyclizations. Toward vicinal all-carbon quaternary stereocenters. *Org. Lett.* **14**, 2940–2943 (2012).
16. Bosque, I., Bagdatli, E., Foubelo, F. & Gonzalez-Gomez, J. C. Regio- and stereoselective aminopentadienylation of carbonyl compounds. *J. Org. Chem.* **79**, 1796–1804 (2014).
17. Li, G. Y. & Che, C. M. Highly selective intra- and intermolecular coupling reactions of diazo compounds to form cis-alkenes using a ruthenium porphyrin catalyst. *Org. Lett.* **6**, 1621–1623 (2004).

18. Mahesh, M., Murphy, J. A. & Wessel, H. P. Novel deoxygenation reaction of epoxides by indium. *J. Org. Chem.* **70**, 4118–4123 (2005).
19. Tamura, O. *et al.* Intramolecular cycloaddition of O-tert-butyldimethylsilyloximes in the presence of BF3-OEt2. *J. Org. Chem.* **70**, 10720–10725 (2005).
20. Musacchio, A. J., Nguyen, L. Q., Beard, G. H. & Knowles, R. R. Catalytic olefin hydroamination with aminium radical cations: A photoredox method for direct C-N bond formation. *J. Am. Chem. Soc.* **136**, 12217–12220 (2014).
21. Miege, F., Meyer, C. & Cossy, J. Rhodium-catalyzed cycloisomerization involving cyclopropenes: Efficient stereoselective synthesis of medium-sized heterocyclic scaffolds. *Angew. Chemie - Int. Ed.* **50**, 5932–5937 (2011).
22. Kimura, M., Mori, M. & Tamaru, Y. Palladium-catalyzed 1,3-diol fragmentation: Synthesis of ??-dienyl aldehydes. *Chem. Commun.* 4504–4506 (2007). doi:10.1039/b708526e
23. Miyata, O. *et al.* Radical cyclization in heterocycle synthesis. Part 13: Sulfanyl radical addition-cyclization of oxime ethers and hydrazones connected with alkenes for synthesis of cyclic β-amino acids. *Tetrahedron* **58**, 4459–4479 (2002).
24. Desrat, S. & Van De Weghe, P. Intramolecular imino Diels - Alder reaction: Progress toward the synthesis of uncialamycin. *J. Org. Chem.* **74**, 6728–6734 (2009).
25. Cremonesi, G., Croce, P. D., Fontana, F., Fiorelli, C. & Rosa, C. La. Stereoselective synthesis of β,ε-dihydroxy-α-amino acids by ring opening of 4,5-dihydroisoxazolyl derivatives. *Tetrahedron Asymmetry* **19**, 2850–2855 (2008).
26. Enders, D. & Gries, J. Asymmetric synthesis of substituted azetidine type α- and β-amino acids. *Synthesis (Stuttg).* 3508–3516 (2005). doi:10.1055/s-2005-918421
